# Neutrophil to lymphocyte ratio and cancer prognosis: an umbrella review of systematic reviews and meta-analyses of observational studies

**DOI:** 10.1186/s12916-020-01817-1

**Published:** 2020-11-20

**Authors:** Meghan A. Cupp, Margarita Cariolou, Ioanna Tzoulaki, Dagfinn Aune, Evangelos Evangelou, Antonio J. Berlanga-Taylor

**Affiliations:** 1 Department of Epidemiology & Biostatistics, MRC Centre for Environment and Health, School of Public Health, Faculty of Medicine, Imperial College London, St Mary’s Campus, Norfolk Place, London, W21PG UK; 2 Department of Hygiene and Epidemiology, University of Ioannina Medical School, Ioannina, Greece; 3 Department of Nutrition, Bjørknes University College, Oslo, Norway; 4 Department of Endocrinology, Morbid Obesity and Preventive Medicine, Oslo University Hospital, Oslo, Norway

**Keywords:** Cancer, Neutrophils, Neutrophil to lymphocyte ratio, Tumour-associated neutrophils, Prognosis, Umbrella review

## Abstract

**Background:**

Although neutrophils have been linked to the progression of cancer, uncertainty exists around their association with cancer outcomes, depending on the site, outcome and treatments considered. We aimed to evaluate the strength and validity of evidence on the association between either the neutrophil to lymphocyte ratio (NLR) or tumour-associated neutrophils (TAN) and cancer prognosis.

**Methods:**

We searched MEDLINE, Embase and Cochrane Database of Systematic Reviews from inception to 29 May 2020 for systematic reviews and meta-analyses of observational studies on neutrophil counts (here NLR or TAN) and specific cancer outcomes related to disease progression or survival. The available evidence was graded as strong, highly suggestive, suggestive, weak or uncertain through the application of pre-set GRADE criteria.

**Results:**

A total of 204 meta-analyses from 86 studies investigating the association between either NLR or TAN and cancer outcomes met the criteria for inclusion. All but one meta-analyses found a hazard ratio (HR) which increased risk (HR > 1). We did not find sufficient meta-analyses to evaluate TAN and cancer outcomes (*N* = 9). When assessed for magnitude of effect, significance and bias related to heterogeneity and small study effects, 18 (9%) associations between NLR and outcomes in composite cancer endpoints (combined analysis), cancers treated with immunotherapy and some site specific cancers (urinary, nasopharyngeal, gastric, breast, endometrial, soft tissue sarcoma and hepatocellular cancers) were supported by strong evidence.

**Conclusion:**

In total, 60 (29%) meta-analyses presented strong or highly suggestive evidence. Although the NLR and TAN hold clinical promise in their association with poor cancer prognosis, further research is required to provide robust evidence, assess causality and test clinical utility.

**Trial registration:**

PROSPERO CRD42017069131.

## Background

Cancer is the second leading cause of mortality worldwide [1], contributing to over 8.7 million deaths globally in 2015 [2]. Cancer incidence is increasing due, in part, to higher morbidity from chronic diseases and epidemiological transitions in developing countries [3]. This increase highlights the importance of identifying prognostic indicators associated with cancer progression such as the neutrophil to lymphocyte ratio (NLR) [4]. The link between inflammation and cancer was first observed by Rudolf Virchow, who detected leukocytes within tumours and hypothesised that inflammation increased cellular proliferation [5]. Since this discovery in the nineteenth century, inflammation has been recognised as one of the six biological capabilities of tumour development and a hallmark of cancer [6], with links to cancer initiation, progression and metastasis [7]. The paradoxical role of neutrophils in both the prevention and facilitation of tumour progression has generated significant research interest around neutrophils in the tumour microenvironment [8].

The NLR has emerged as a potential biomarker of cancer prognosis and is of particular clinical interest due to its accessibility and the ease of calculating the ratio from patients’ routine blood cell counts [9]. The NLR was first recognised for its association with systemic inflammation in the critically ill and meta-analyses on the association between elevated NLR and poor prognosis have reported a wide range of effect sizes, depending on the site of cancer [9, 10]. The close association between inflammation and cancer progression hints at the potential of elevated tumour-associated neutrophils (TAN), or neutrophils which infiltrate tumours [11], as a prognostic biomarker [8, 12, 13]. Many systematic reviews and meta-analyses have explored the association between neutrophils and cancer prognosis. However, the myriad of different cancer sites, stages, treatments, survival outcomes and cut-off values for classifying a “high” NLR complicates the interpretation of this body of evidence.

It is currently unclear how the association between NLR and poor prognosis varies depending on the site of cancer or the treatment considered. Umbrella reviews allow for the analysis of such broad subject areas to examine the strength and credibility of associations using the results of published systematic reviews and meta-analyses [14, 15]. Umbrella review methods assess the strength and consistency of the literature to evaluate bias and identify which associations are supported by strong evidence [14]. Here we carried out an umbrella review of systematic reviews and meta-analyses with the aim of comprehensively evaluating the validity and strength of reported associations between NLR or TAN and cancer prognosis and identify potential biases in relevant literature.

## Methods

### Literature search

Searches were conducted in MEDLINE, Embase and the Cochrane Database (Additional File 1: Appendix A) and aimed to include all systematic reviews and meta-analyses published in English from inception up to 29 May 2020. Measures of neutrophil counts included NLR and TAN (intratumoural, peritumoural and stromal neutrophils). Overall survival (OS), cancer-specific survival (CSS), progression-free survival (PFS), disease-free survival (DFS) and reoccurrence-free survival (RFS) were considered as cancer outcomes. Articles were initially screened by title and abstract to determine eligibility for full text screening and inclusion using RefWorks web-based bibliography and database manager [16].

### Inclusion and exclusion criteria

Included studies were systematic reviews and meta-analyses of individual observational studies in humans with any cancer diagnosis and NLR or TAN measurements taken around the time of diagnosis. Systematic reviews which did not include a meta-analysis were excluded. Meta-analyses were excluded if they did not assess a cancer outcome in our inclusion criteria, included more than one outcome in a single analysis, did not specify the cancer site or included multiple cancer sites in a single analysis without clarifying whether there was a shared feature (e.g. analyses that combined cancers and classified them as “other cancers” without further details). However, meta-analyses which assessed multiple cancers in a single analysis based on a shared feature were included and classified as “composite cancer endpoints”. Meta-analyses were also excluded if they did not provide sufficient detail for replication, such as the hazard ratio (HR), 95% confidence interval and total sample size of each individual study included in a meta-analysis. If a single systematic review included multiple meta-analyses, all meta-analyses were individually assessed for eligibility.

When more than one meta-analysis was identified for a single association at a specific site, they were assessed for concordance in the direction, magnitude and significance of their effect estimates. If the duplicate meta-analyses identified agreed in significance, magnitude and direction of effect, the meta-analysis with the greatest number of component studies was included. Where disagreement was found, both duplicate meta-analyses were excluded unless the disagreement arose from an underpowered meta-analysis (less than five component studies), in which case the meta-analysis with the greatest number of component studies was used.

### Data extraction

Data extraction forms were generated to record information from each meta-analysis and the included individual studies. First author, year of publication, outcome measure, biomarker and cancer diagnosis were extracted from each meta-analysis. For each included individual study within a meta-analysis, the first author, year of publication, total population, epidemiological design, HR and 95% confidence interval were extracted, along with analysis method and NLR cut-off where available.

### Data analysis

The weighted inverse variance method was used with restricted maximum likelihood (REML) estimations to reproduce all included meta-analyses in R with the “meta” package [17] and “metagen” command [18]. For each cancer site-specific biomarker and outcome pair, the summary effect size and 95% confidence interval were calculated using fixed and random effects models with adjustment by the Knapp-Hartung modification. To take into account heterogeneity between studies, a random effects model was used to compute summary effect size estimates [19, 20]. Estimates from the fixed effects model are also presented.

Each included meta-analysis was reproduced to yield both fixed and random effects estimates. Reproduced random or fixed effect estimates which did not match the results of the original review results were assessed for absolute and percent difference. Meta-analyses with a difference in HR of 0.01 were attributed to rounding errors. Studies with larger discrepancies were investigated to determine the source of disagreement. Where there were issues with reproducibility, the calculated values of the random effects model were used to assess the evidence for the association.

We calculated 95% prediction intervals (PI) in order to assess the impact of uncertainty around the summary effect size estimate and between-study variance (*Tau*) [21]. Prediction intervals account for the uncertainty caused by heterogeneity when estimating the distribution of true effect sizes in an association and yield an interval which predicts the effect size of future studies investigating the same association [21]. In studies with large amounts of heterogeneity or an effect size close to the null value, the prediction interval may be wide enough to include the null value (HR = 1). This suggests the true effect size in a single meta-analysis may be a HR of 1 or < 1. We further assessed heterogeneity with Cochran’s Q test and quantified using the *I*^2^ statistic [22]. We considered Cochran’s Q test to detect a departure from homogeny in the effect sizes of individual studies when *P* < 0.10 [22]. Due to common limitations associated with Cochran’s Q test, the *I*^2^ statistic was also used to quantify the percentage of variation which can be attributed to heterogeneity [22]. The 95% confidence intervals around each *I*^2^ value were included to evaluate the uncertainty around estimates of heterogeneity [23]. However, large measures of variation due to heterogeneity, representing true heterogeneity or inconsistency due to bias [24], were primarily assessed through prediction intervals. To further assess heterogeneity, we used sub-group analysis and meta-regression and as detailed below.

To further explore heterogeneity and determine the impact of adjustment with additional prognostic factors, sub-group analyses of the statistical models used in each study were conducted. Meta-analyses which reported the analysis method (univariate or multivariate) of their component studies were included. The Q test for subgroup differences was used to test for significant differences in the random effects model effect size between subgroups in each meta-analysis (*P* < 0.05).

Meta-regression was used to assess NLR cut-offs as a source of heterogeneity in all component studies that reported them [25]. We used a random-effects meta-regression model with REML estimates to account for residual heterogeneity and the Knapp-Hartung method to adjust CIs and test statistics [26].

Small study effects and funnel plot asymmetry were quantified through the arcsine-Thompson test described by Rücker et al. [27] and the command “metabias” from the R package “meta” [17, 27]. The arcsine-Thompson has greater power than similar tests of small study effects when heterogeneity is present; however, it may be overly conservative when no heterogeneity is present [27]. A low significance value in the arcsine-Thompson test (*P* < 0.10) was used to indicate presence of small study effects which could reflect publication and other selective reporting biases. Further assessment was carried out to determine if the summary effect size estimate of each meta-analysis was greater than the point estimate of the largest included study, indicating potential small study effects [28]. A meta-analysis was judged to have evidence of small study effects if either one of these criteria was met.

The test for excess significance (TES) was used to determine if the number of observed significant results differed significantly from the expected number, indicating reporting bias [29]. TES results can reveal reporting bias if the number of observed studies with significant results in each meta-analysis is significantly larger than the expected number using a two-tailed binomial probability test (*P* < 0.10) [30]. The expected number of significant results in each meta-analysis was calculated as the sum of the statistical power estimate, or the probability that each component study will find a positive result [29, 30]. The estimated power for each component study was calculated in Stata 14 [31], using the “power cox” command to calculate the power of each test given its sample size, effect size and significance level. The estimation of power for each component study also requires an estimation of the true effect size, so the effect size of the largest study was used to give an estimation of true effect with the smallest standard error. Estimates from both fixed and random effects models were included for sensitivity analysis. The “binom.test” command in R was used to assess the significance of differences in the number of observed versus expected significant studies using an exact binomial test .

Credibility ceilings were utilised for sensitivity analysis and to test the robustness of meta-analyses, considering studies of biomarkers often underestimate biases due to large sample sizes and observational study design [32, 33]. Credibility ceiling calculations inflate the variance of each study included in a meta-analysis to account for the probability *c* that the true effect size is in the opposite direction of effect of the observed point estimate [33]. Inflated variances were calculated in Stata 14 [31, 32]. The summary effect size and heterogeneity of each meta-analysis was assessed with ceiling values ranging from 5 to 20%.

Associations between neutrophil counts (here NLR or TAN) and cancer prognosis were categorised into strong, highly suggestive, suggestive, weak or uncertain through assessment of the strength and validity of the evidence for each meta-analysis, according to pre-defined Grading of Recommendations, Assessment, Development and Evaluations (GRADE) criteria outlined in Additional File 2: Supplementary Fig. 1 [34]*.* In order for an association to be considered strong, the meta-analysis must yield a *P* value of less than 10^− 6^ in the random effects model [35], include more than 1000 individuals, show significance at *P* < 0.05 in the largest included study, find no heterogeneity (*P* > 0.10) through the Q test, detect less than 50% variance due to heterogeneity through the *I*^2^ statistic, yield a prediction interval excluding the null value (HR = 1), display no evidence of small study effects or excess significance, and the association must maintain significance at *P* < 0.05 with the application of a credibility ceiling of 10%. The number of studies in each meta-analysis was also included as eligibility criterion for strong evidence since a sample size greater than three is required for reliable assessment of heterogeneity and small study effects [22, 36, 37]. Associations categorised as “highly suggestive” were eligible to be upgraded to “strong” if they presented a HR > 2 and a lower 95% CI > 1.6 [38]. To assess the potential impact of limitations around the measures assessing heterogeneity on the GRADE criteria, we performed a sensitivity analysis in which *I*^2^ and Cochran’s Q test criteria were removed for associations classified as strong. Additionally, we applied a simplified method to rank associations based only on their effect size estimate (HR) and precision (standard error from PI or CI intervals) to aid interpretation.

Studies with meta-analyses categorised as providing either highly suggestive or strong evidence underwent quality assessment through AMSTAR 2, a tool for assessing the methodological quality of systematic reviews for both health interventions and observational research [39, 40]. Furthermore, assessment of the component studies included in each meta-analysis providing strong evidence was carried out with the Quality in Prognostic Studies (QUIPS) tool [41]. Studies were assessed by two reviewers (MAC and MC) and consensus reached on any disagreements in quality.

Statistical analyses were carried out in R [18], including the packages “meta” version 4.8-4 and “ggplot2” version 2.2.1, and in Stata 14 [17, 31, 42].

### Role of the funding source

Funders had no role in data collection, analysis, interpretation or writing of the report. All authors had access to all the data in the study.

### Patient involvement

No patients were involved in development of our umbrella review design nor were they asked to advise on interpretation. No ethical approval was required for this review since it relied entirely on anonymised, published data.

## Results

### Characteristics of included meta-analyses

**Fig. 1:**
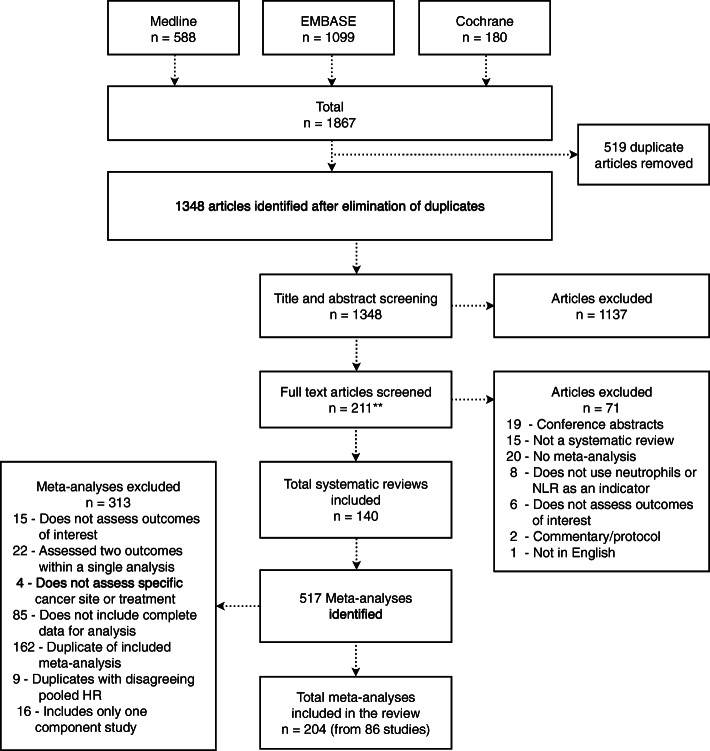
Flowchart of systematic review and meta-analysis selection

The 140 published articles meeting the criteria for inclusion contained 517 meta-analyses (Additional File 1: Appendix B). The 204 meta-analyses meeting the eligibility criteria arose from 86 of these articles, published between 2014 and 2020 (Fig. 1) [43–128]. These meta-analyses included individual studies which presented NLR (*N* = 195) or TAN (*N* = 9) categorically as either a high or low value. Included meta-analyses summarised effect size estimates from 1978 individual studies, with OS as the most frequently assessed outcome (*N* = 90). In 135 meta-analyses (66%), total sample size exceeded 1000 individuals and each meta-analysis had a median of seven studies. However, 134 meta-analyses (66%) included less than ten studies and 25 (12%) included only two studies. The characteristics of included meta-analyses are summarised in Additional File 3: Supplementary Table 1.

A total of 171 duplicate meta-analyses were excluded. Nine meta-analyses assessing three associations were excluded due to disagreement in significance between duplicates. A further 162 duplicate meta-analyses that agreed in significance, magnitude and direction of effect were excluded for 69 associations and only the meta-analysis with the largest number of studies was included for each association (Additional File 1: Appendix C).

### Summary effect size

All estimated summary effect sizes for both fixed and random effects estimates are shown in Additional File 2: Supplementary Figs. 2–205.

Using a threshold of *P* < 0.05 for statistical significance, 188 of the 204 meta-analyses (92%) were significant with random effects. At a more stringent threshold of *P* < 10^− 6^, the number of statistically significant meta-analyses for random effects dropped to 93 (46%) (Additional File 3: Supplementary Table 1). The 93 meta-analyses with significance at *P* < 10^− 6^ assessed both NLR and TAN as biomarkers of poor prognosis. Ninety of these 93 meta-analyses (97%) assessed NLR as a biomarker of poor prognosis in melanoma, neurologic, gynaecologic, pancreatic, gastrointestinal and oesophageal, colorectal, hepatocellular and biliary, respiratory and oral, urinary, head and neck, soft tissue sarcoma (STS), treatment with immunotherapy, and composite cancer endpoints. TAN (intratumoural neutrophils) were assessed as a biomarker of poor prognosis in three of the 93 meta-analyses (3%), including urinary and composite cancer endpoints.

**Fig. 2:**
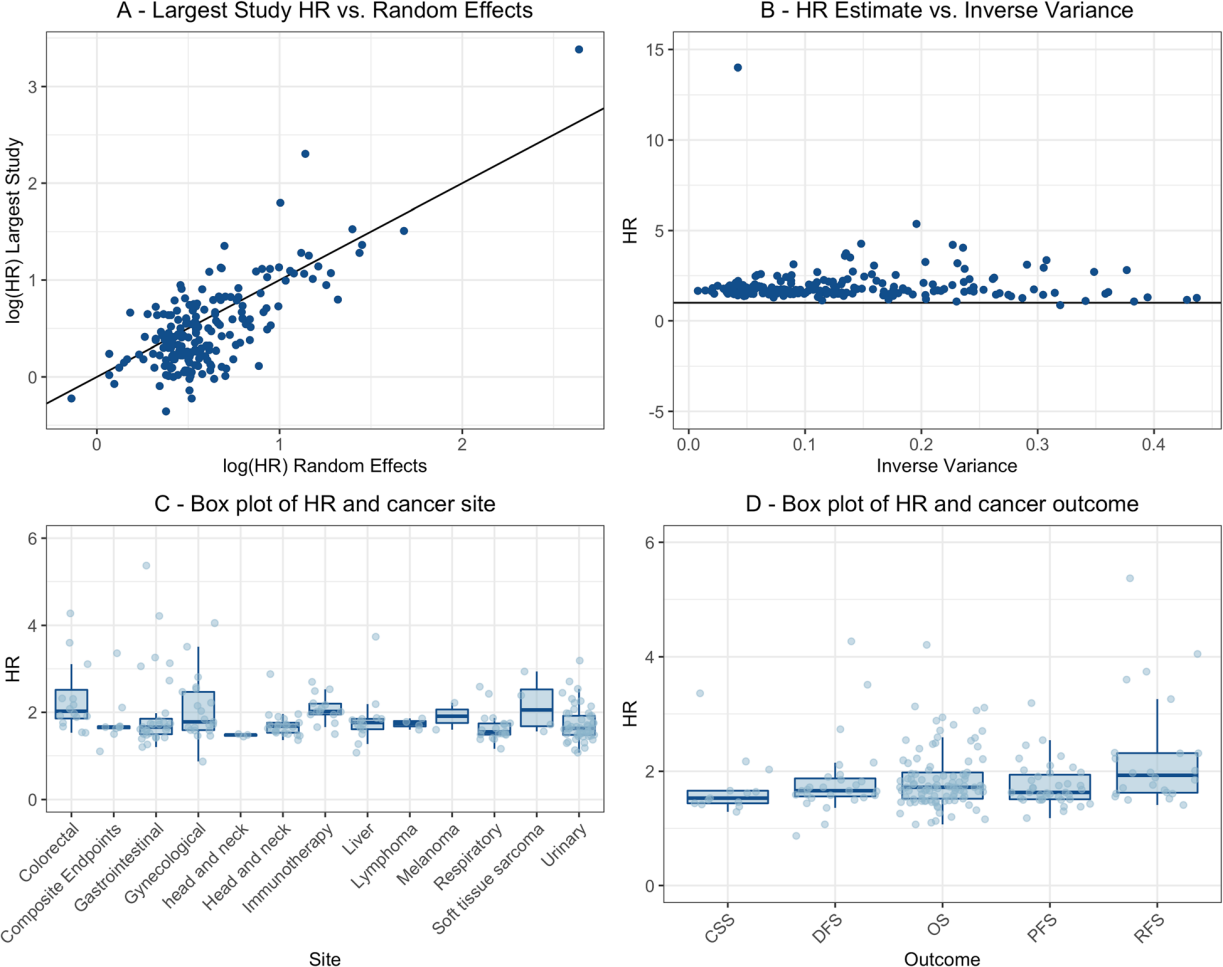
Assessment of consistency in meta-analyses. **a** Log (HR) of largest study versus log (HR) of random effects estimates for each meta-analysis. The *Y*-axis labelled “log (HR) Largest Study” represents the log of the HR of the largest component study. The *X*-axis labelled “log (HR) Random Effects” represents the log of the HR of the random effects estimate calculated in each meta-analysis. **b** Random effects estimates versus inverse variance. The *Y*-axis labelled “Random Effects Estimates Hazard Ratio” represents the HR of random effects estimate for each meta-analysis. The *X*-axis labelled “Inverse Variance” represents the inverse of the variance for each meta-analysis. **c, d** Box plots of random effects HR estimates for each meta-analysis by cancer site and outcome. The *Y*-axis labelled “HR” details the effect size for each meta-analysis describing an association between NLR or TAN and cancer prognosis for each site grouping. The *X*-axis labelled “Site” in **c** represents each site group meta-analyses have been sorted into. The composite endpoints subgroup is defined as a grouping of cancer diagnosis unrelated to site, stage or treatment. The *X*-axis labelled “Outcome” in **d** represents the prognostic outcome assessed in each meta-analysis. The outlier of HR = 14 for NLR and OS in rectal cancer has been excluded from these figures

In 48 meta-analyses (24%), the largest component study was not statistically significant at *P* < 0.05. However, 42 (88%) of these meta-analyses still had a statistically significant summary random effects estimate. In three meta-analyses [68, 70, 94], the largest component study had an effect size in the opposite direction to the random effects estimate (HR < 1). The effect size estimates of the largest component studies tended to be more conservative than the random effect estimates, with 136 meta-analyses (67%) yielding a HR which was greater than the point estimate of the largest component study. However, there was correlation between the log (HR) of the summary random effects and the largest component study for each meta-analysis, indicating consistency in the results (Fig. 2a).

In order to determine the impact of study size on the magnitude of the summary effect size, random effects estimates were plotted against the inverse variance of the pooled effect size from each meta-analysis. When compared to meta-analyses with large variances, those with smaller variances produced more conservative estimates, displaying a smaller range of HR estimates and a slight tendency toward a null value (HR = 1). Meta-analyses with large variance displayed a wide range of random effects HR and included an increased number of HR estimates greater than two (Fig. 2b).

### Reproducibility

In 87 of the 204 included meta-analyses (43%), the pooled effect size was reproduced with an absolute difference between the calculated and reported HR outside of the range which can be attributed to rounding errors (> 0.01). Twenty-eight of these 87 meta-analyses were within 2% of the reported HR, 35 were between 2% and 5% of the reported HR, 14 were between 5% and 10% and ten meta-analyses reported an HR with over a 10% difference from the calculated HR (Additional File 1: Appendix D).

### Heterogeneity between studies

Prediction intervals were not calculated for 25 (12%) meta-analyses which had included only two individual studies (Additional File 3: Supplementary Table 1). The prediction intervals of 131 meta-analyses (64%) included the null value of HR = 1. Of 179 meta-analyses (87%) including at least three individual studies, 47 had prediction intervals which excluded the null value (HR = 1). The 25 meta-analyses (12%) including exactly three individual studies yielded very wide prediction intervals, all of which included the null value of HR = 1. For completeness, we also calculated *I*^2^ values and Cochran’s Q test although they must be interpreted with caution due to low power, consistent direction of effect and moderate magnitude of effects (Additional File 1: Appendix E).

### Subgroup analyses of adjustment of effect estimates

In the subgroup analyses of the 48 meta-analyses (24%) which reported on the analysis methods utilised by component studies, a significant difference was found between the multivariate and univariate groups in only six (13%) meta-analyses (Additional File 1*: Appendix F* and Additional File 2: Supplementary Figs. 2–*205*). In general, adjustment of HRs resulted in minor modification in the strength of association with no consistent pattern. In most cases, summary estimates obtained with the random effects model showed slightly stronger or weaker associations with no change in the direction of effect and no major differences in the width of the 95% CI. However, we note that the majority of studies had 10 or fewer component studies. In studies where both univariate and multivariate meta-analyses included at least five component studies, 95% PIs tended to be wider for multivariate studies, with lower bounds close to the null.

Study authors of the included meta-analyses often reported that HRs derived from multivariate models were preferentially included. Additional factors that were typically adjusted for included gender, age, smoking, body mass index, co-morbidities, C-reactive protein, primary tumour location, stage and grade. These could constitute a core set which future studies could consider adjusting for at a minimum. Concurrent infections, other inflammatory markers (such as fibrinogen and erythrocyte sedimentation rate), ethnicity and current medications were not typically reported and could modify the estimates.

### Meta-regressions of NLR threshold values

156 meta-analyses (76%) reported sufficient information on NLR cut-offs to undergo meta-regression analysis (Additional File 1: Appendix G). Of these, 97 (62%) yielded a positive association between NLR and effect size, however only 14 (11%) showed significance. In the remaining 59 meta-analyses (38%) with a negative association, only three (5%) showed significance. We observed extreme R^2^ in many cases, likely due to small sample sizes [129]. In 82 meta-analyses (53%), the addition of NLR cut-off did not account for any of the heterogeneity observed, indicated by an R^2^ value of 0%. In 15 (20%) of the remaining 74 meta-analyses, the addition of NLR cut-off values accounted for 100% of the heterogeneity, with a mean and standard deviation of 50.15 ± 39.94%.

Results of the meta-regressions should be interpreted with caution since only 58 meta-analyses (37%) included the recommended threshold of ten or more component studies to be considered reliable [25]. Of these 58 meta-analyses, 42 (72%) suggested a positive association between NLR and effect size, with only four (10%) showing significance. The 16 meta-analyses (28%) suggesting a negative association did not show significance. Bubble plots of the regression of log HR on NLR cut-off are available in Additional File 2: Supplementary Figs. 2–*205*. Although few showed significance, most meta-regressions yielded a positive association between NLR cut-off and effect size, suggesting the dose-response relationship should be explored further in future studies. Ideally, continuous data should be reported instead of non-standardised thresholds*.*

### Small study effects

179 meta-analyses (88%) included two or more studies and were eligible for further assessment through the arcsine-Thompson test for publication bias [27]. Eighty-eight (49%) of these 179 meta-analyses yielded significant *P*-values (*P* < 0.10), indicating potential small study effects. The presence of small study effects was also assessed through comparison of the random effects model effect estimate and the effect estimate of the largest component study. In 68 meta-analyses (33%), the summary effects estimate from the largest component study was larger than the point estimate of the model and were considered to have evidence of small study effects. Taking both criteria into account, 136 (67%) of the 204 included meta-analyses were judged to have evidence of small study effects *(*Additional File 1: Appendix E).

### Excess significance

Forty-two meta-analyses (21%) showed evidence of excess significance bias according to the TES when the effect size of the largest component study was utilised as an estimate of true effect size (Additional File 1: Appendix H). When the fixed summary effect sizes were utilised as an estimation of true effect size, 50 meta-analyses (25%) showed evidence of excess significance. Four meta-analyses (2%) showed evidence when the random summary effect sizes were used.

### Credibility ceilings

The summary effect size estimates and significance of each meta-analysis matched that of the random effects model at a credibility ceiling of 0%, with 188 of the 204 meta-analyses being significant at *P* < 0.05 (93%) (Table 1).

**Table 1:** Credibility ceiling results

All meta-analyses	Ceiling 0%	Ceiling 5%	Ceiling 10%	Ceiling 15%	Ceiling 20%
***X = 1, n (%)***					
Number of meta-analyses with effect size > 1.0	204 (100)	204 (100)	204 (100)	204 (100)	201 (99)
Number of meta-analyses with nominal statistical significance	188 (92)	178 (87)	139 (68)	84 (41)	51 (25)

At a ceiling of 5%, 178 maintained significance (87%) and 139 (68%), 84 (41%) and 51 (25%) maintained significance at ceilings of 10%, 15% and 20%, respectively. All of the meta-analyses remained consistent in direction of effect (HR > 1) up to a ceiling of 15% and three (1.5%) yielded an effect estimate in the opposite direction (HR < 1) with a ceiling of 20%. The *I*^2^ value of each meta-analysis decreased with each increase in ceiling value.

### Grading the evidence

Each included meta-analysis was evaluated to determine if the association of interest was supported by strong, highly suggestive, suggestive or weak evidence (Additional File 1: Appendix I). In 16 meta-analyses (8%), no significance was detected at a threshold of *P* < 0.05. The remaining 188 meta-analyses (92%) provided at least weak evidence of an association (*P* < 0.05) (Table 2).

**Table 2:** Grading of evidence

Evidence	Criteria	Increased risk of poor prognosis
Strong (*N* = 18)	*P* < 10–6* with random effects; > 1000 individuals included; > 3 studies included; largest study significant at *P* < 0.05; Q test significant at *P* < 0.10; *I*^2^ less than 50%, prediction interval does not include null value (HR = 1); small study effects not detected; excess significance not detected	Bladder (UTR)—RFS [NLR], Breast—OS [NLR], Breast (SR) —OS [NLR], CLM—OS [NLR], CLM (SR) —OS [NLR], Endometrial—OS [NLR], Gastric (SR)—OS [NLR], Immunotherapy (Immune checkpoint inhibitors)—OS [NLR], Melanoma (Immune checkpoint inhibitors)—OS [NLR], Nasopharyngeal—OS [NLR], Nasopharyngeal—PFS [NLR], Non-muscle-invasive bladder—PFS [NLR], NSCLC (PD-1 inhibitor)—OS [NLR], Prostate CR—OS [NLR], Renal (TKI)—OS [NLR], STS—DFS [NLR]
	Upgraded to “strong” from “highly suggestive” if HR > 2, lower 95%CI > 1.6	Advanced Cancer (Anti-VEGFR)—OS [NLR], Composite endpoints—DFS [NLR], Bladder (UTR)—RFS [NLR], Breast—OS [NLR], Breast (SR)—OS [NLR], CLM—OS [NLR], CLM (SR)—OS [NLR], Endometrial—OS [NLR], Gastric (SR)—OS [NLR], Immunotherapy (Immune checkpoint inhibitors)—OS [NLR], Melanoma (Immune checkpoint inhibitors)—OS [NLR], Nasopharyngeal—OS [NLR], Nasopharyngeal—PFS [NLR], Non muscle Invasive Bladder—PFS [NLR], NSCLC (PD-1 inhibitor)—OS [NLR], Prostate CR—OS [NLR], Renal (TKI)—OS [NLR]
Highly suggestive (*N* = 42)	*P* < 10–6* with random effects; > 1000 individuals included; largest study significant at *P* < 0.05	Advanced Cancer (Immunotherapy)—PFS [NLR], Composite endpoints—PFS [NLR], Biliary Tract—OS [NLR], Bladder—RFS [NLR], Breast (Metastasis)—DFS [NLR], Breast (Triple Negative and Her2 Positive)—DFS [NLR], Colorectal—OS [NLR], CLM—RFS [NLR], Gastric—OS [NLR], Gynaecologic—OS [NLR], Head and Neck—OS [NLR], Hepatocellular—OS [NLR], Hepatocellular—DFS [NLR], Hepatocellular—RFS [NLR], Hepatocellular (Sorfenib)—OS [NLR], Hepatocellular (SR)—RFS [NLR], Hepatocellular (SR)—OS [NLR], Hepatocellular (SR)—DFS [NLR], Hepatocellular (TACE)—OS [NLR], Advanced Cancer (Immunotherapy)—OS [NLR], Immunotherapy (Immune checkpoint inhibitors)—PFS [NLR], Lung (both)—OS [NLR], NAC—OS [NLR], NSCLC—PFS [NLR], NSCLC (PD-1 inhibitor)—PFS [NLR], NSCLC (ST)—PFS [NLR], Oesophageal—OS [NLR], Oesophageal (DCRT)—OS [NLR], Oesophageal (Surgery)—OS [NLR], Oesophageal (Surgery+/−Chemo)—OS [NLR], Oesophageal SCC—OS [NLR], Oral cavity—OS [NLR], Oral cavity—DFS [NLR], Pancreatic—OS [NLR], Pancreatic (SR)—OS [NLR], Prostate—PFS [NLR], Prostate (Metastatic)—OS [NLR], Prostate CR (Enzalutamide)—OS [NLR], Renal—OS [NLR], Renal Advanced—OS [NLR], STS—OS [NLR], Urothelial (Chemotherapy)—OS [NLR]
Suggestive (*N* = 51)	*P* < 10–4* with random effects; > 1000 individuals included	Composite endpoints—OS [NLR], Composite endpoints—OS [IN], Composite endpoints Operable—OS [NLR], Composite endpoints Operable—CSS [NLR], Bladder—OS [NLR], Bladder (UTR)—PFS [NLR], Bladder and Upper Urinary—PFS [NLR], Bladder and Upper Urinary—CSS [NLR], Breast—DFS [NLR], Breast (SR)—DFS [NLR], Breast (Triple Negative and Her2 Positive)—OS [NLR], Cervical—OS [NLR], Cervical—PFS [NLR], Colorectal—DFS [NLR], Colorectal—PFS [NLR], Colorectal (SR)—OS [NLR], CLM (Non-surgical)—OS [NLR], Endometrial—PFS [NLR], Glioma—OS [NLR], Head and neck—DFS [NLR], Head and Neck (No Surgery)—OS [NLR], Head and Neck (SCC)—CSS [NLR], Head and Neck (SCC)—OS [NLR], Head and Neck (SCC)—DFS [NLR], Hepatocellular (Transplant)—OS [NLR], Larynx—DFS [NLR], Larynx—OS [NLR], Lung (Surgery)—OS [NLR], Melanoma—OS [NLR], Multiple Myeloma—OS [NLR], Nasopharyngeal—CSS [NLR], Non muscle Invasive Bladder—RFS [NLR], NSCLC—OS [NLR], NSCLC (Chemotherapy)—OS [NLR], NSCLC (Immunotherapy)—OS [NLR], NSCLC (ST)—OS [NLR], Oesophageal (No Surgery)—OS [NLR], Oral SCC—OS [NLR], Ovarian—PFS [NLR], Ovarian—OS [NLR], Prostate—OS [NLR], Prostate CR—PFS [NLR], Prostate CR (Abitaterone)—OS [NLR], Rectal—OS [NLR], Renal—PFS [NLR], Urinary—OS [NLR], Urothelial (Nephroureterectomy)—OS [NLR], Urothelial (Nephroureterectomy)—PFS [NLR], Urothelial (Radical Cystectomy)—CSS [NLR], UTUC (Nephroureterectomy)—CSS [NLR], UTUC (Radical Cystectomy)—CSS [NLR],
Weak (*N* = 77)	*P* < 0.05* with random effects	Composite endpoints—CSS [IN], Biliary Tract—RFS [NLR], Bladder (Metastasis)—OS [NLR], Bladder (Radical Cystectomy)—OS [NLR], Breast—CSS [NLR], Breast (Metastasis)—OS [NLR], Breast (SR)—RFS [NLR], Breast (Triple-negative)—OS [NLR], Breast (Triple-negative)—DFS [NLR], Colorectal (PC)—DFS [NLR], Colorectal (SR)—DFS [NLR], Colorectal (SR)—RFS [NLR], CLM (Non-surgical)—RFS [NLR], CLM (SR)—RFS [NLR], Gastric—DFS [NLR], Gastric—PFS [NLR], Gastric (SR)—DFS [NLR], GNT—OS [NLR], GNT—RFS [NLR], Head and Neck—RFS [NLR], Head and neck—PFS [NLR], Head and Neck—OS [IN], Head and Neck (SCC)—PFS [NLR], Head and Neck (SR)—OS [NLR], Hepatocellular (MT)—OS [NLR], Hepatocellular (RFA)—OS [NLR], Hepatocellular (Transplant)—RFS [NLR], Hepatocellular and ICC—OS [IN], Hypopharynx—OS [NLR], Ipilimumuab—OS [NLR], Ipilimumuab—PFS [NLR], Large B—OS [NLR], Large B—PFS [NLR], Lung (both)—PFS [NLR], Melanoma—PFS [NLR], Melanoma (Immune checkpoint inhibitors)—PFS [NLR], MPM—OS [NLR], Multiple Myeloma—PFS [NLR], NAC—RFS [NLR], NAC—CSS [NLR], Nivolumab—OS [NLR], Nivolumab—PFS [NLR], Non muscle Invasive Bladder (High Risk)—RFS [NLR], Non muscle Invasive Bladder (High Risk)—PFS [NLR], NSCLC (Chemotherapy)—PFS [NLR], NSCLC (Immune Checkpoint Inhibitors)—PFS [NLR], NSCLC (Nivoumab)—OS [NLR], NSCLC (Nivoumab)—PFS [NLR], NSCLC (TT)—OS [NLR], NSCLC (TT)—PFS [NLR], Oesophageal—CSS [NLR], Oesophageal—RFS [NLR], Oesophageal—DFS [NLR], Oesophageal—PFS [NLR], Oesophageal (Neo + Surgery)—OS [NLR], Oral SCC—DFS [NLR], Oropharynx—DFS [NLR], Pancreatic—DFS [NLR], Pancreatic—CSS [NLR], Pancreatic Neuroendocrine Tumour—OS [NLR], Pancreatic NeuroendocrineTumour—RFS [NLR], Rectal—DFS [NLR], Rectal—RFS [NLR], Renal—RFS [NLR], Renal—OS [IN], Renal (TKI)—PFS [NLR], Renal Advanced—PFS [NLR], Renal Localised—RFS [NLR], STS (Synovial Sarcoma)—OS [NLR], STS (Liposarcoma)—OS [NLR], Upper Urinary—RFS [NLR], Urothelial—OS [NLR], Urothelial—RFS [NLR], Urothelial (Chemotherapy)—PFS [NLR], Urothelial (Radical Cystectomy)—OS [NLR], Urothelial (Radical Cystectomy)—PFS [NLR], Prostate Localised—RFS [NLR]
Uncertain (*N* = 16)	Not significant at *P* < 0.05* with random effects	Composite endpoints—OS [PN], Composite endpoints—OS [SN], Bladder (Radical Cystectomy and NAC)—OS [NLR], Breast (No Metastasis)—OS [NLR], Breast (No Metastasis)—DFS [NLR], Gastric—OS [IN], Gastrointestinal Stromal—OS [NLR], Hepatocellular (RFA)—DFS [NLR], NSCLC—OS [IN], Prostate Localised—OS [NLR], Prostate CR (Abitaterone)—PFS [NLR], Prostate CR (Enzalutamide)—PFS [NLR], Renal—CSS [NLR], Renal Localised—OS [NLR], Thyroid—DFS [NLR], Urothelial—DFS [NLR]

*NLR* neutrophil to lymphocyte ratio, *IN* intratumoural neutrophils, *PN* peritumoural neutrophils, *SN* stromal neutrophils, *OS* overall survival, *DFS* disease-free survival, *PFS* progression-free survival, *RFS* reoccurrence-free survival, *CSS* cancer-specific survival, *VEGFR* vascular endothelial growth factor receptor, *PC* palliative chemotherapy, *SBR* surgical bowel resection, *NAC* neoadjuvant chemotherapy, *STS* soft tissue sarcoma, *UTR* urothelial transurethral resection, *SR* surgical resection, *GNT* gastroenteropancreatic neuroendocrine tumours, *SCC* squamous cell carcinoma, *ST* systematic therapy, *MT* mixed therapy, *TT* targeted therapy, *CLM* colorectal liver metastasis, *NS* non-surgical, *ICC* intrahepatic cholangiocarcinoma, *RFA* radiofrequency ablation, *TACE* trans-arterial chemoembolization, *MPM* malignant pleural mesothelioma, *NSCLC* non-small cell lung cancer, *NHC* neck and head cancer, *UTUC* upper tract urothelial carcinoma, *DCRT* definitive chemoradiotherapy, *CR* castration resistant, *TKI* tyrosine kinase inhibitor

*Composite cancer endpoints are defined as a grouping of cancer diagnosis unrelated to site, stage or treatment unless otherwise specified

No meta-analyses presented evidence of elevated neutrophils and improved cancer prognosis (HR < 1)

Strong evidence was presented in 18 meta-analyses (9%) for associations between NLR and poor cancer prognosis. Seven of these associations met the grade criteria for strong evidence, including PFS in non-muscle-invasive bladder cancer (*N* = 6; HR 2.26, 95%CI 1.59–3.22), OS and PFS in nasopharyngeal cancer (*N* = 10; HR 1.48, 95%CI 1.29–1.69 and *N* = 5; HR 1.50, 95%CI 1.30–1.73), OS in castration-resistant prostate cancer (*N* = 9; HR 1.56, 95%CI 1.42–1.72), RFS in bladder cancer with urothelial transurethral resection (UTR) (*N* = 5; HR 2.22, 95%CI 1.81–2.74), OS in endometrial cancer (*N* = 9, HR 2.22, 95%CI 1.76–2.79) and DFS in soft tissue sarcoma (STS) (*N* = 7, HR 1.72, 95%CI 1.43–2.08) and the other 11 were upgraded from highly suggestive (Additional File 1*: Appendix J*), including DFS in composite cancer endpoints (*N* = 20; HR 2.11, 95%CI 1.71–2.60), OS in advanced cancer with anti-vascular endothelial growth factor receptors (VEGFR) (*N* = 14; HR 2.02, 95%CI 1.61–2.53), OS in cancer with immune checkpoint inhibitors (ICI) (*N* = 18; HR 2.21, 95%CI 1.70–2.88), OS in gastric cancer with surgical resection (SR) (*N* = 7; HR 3.13, 95%CI 1.99–4.92), OS in colorectal liver metastasis (CLM) (*N* = 7; HR 2.17, 95%CI 1.83–2.57), OS in CLM with SR (*N* = 5; HR 2.08, 95%CI 1.73–2.49), OS in breast cancer (*N* = 13, HR 2.54, 95%CI 1.96–3.30), OS in renal cancer with tyrosine kinase inhibitors (TKIs) (*N* = 7; HR 2.14, 95%CI 1.66–2.76), OS in melanoma with ICI (*N* = 9, HR 2.49, 95%CI 1.72–3.61), OS in non-small cell lung cancer (NSCLC) with PD-1 inhibitors (*N* = 13, HR 2.59, 95%CI 2.10–3.20) and OS in breast cancer with SR (*N* = 12, HR 2.47, 95%CI 1.71–3.56).

Forty-two meta-analyses (21%) presented associations supported by highly suggestive evidence, including associations between increased NLR and poor prognosis in composite cancer endpoints, cancers treated with immunotherapy, gastric, colorectal, CLM, pancreatic, gynaecologic, breast, hepatocellular, biliary, NSCLC, lung, head and neck, oral, renal, advanced renal cancer, upper urinary and bladder, STS and bladder. The most commonly assessed outcome for highly suggestive associations was OS (*N* = 26), followed by PFS (*N* = 7), DFS (*N* = 5) and RFS (*N* = 4).

Fifty-one meta-analyses (25%) provided suggestive evidence for an association between high NLR (*N* = 50) or TAN (*N* = 1) and poor cancer prognosis, and 77 meta-analyses (38%) provided weak evidence for an association between high NLR or TAN and poor cancer prognosis. The association between intratumoural neutrophils and overall survival in composite cancer endpoints was classified as suggestive, but there was weak evidence supporting associations with peritumoural neutrophils or stromal neutrophils. Details of the grading and upgrading for each meta-analysis are included in Additional File 1: Appendices I and J.

### Sensitivity analysis of evidence classification

The sensitivity analysis of GRADE criteria resulted in only one association between NLR and OS in gastric cancer being reclassified from highly suggestive to strong when *I*^2^ and Cochran’s Q test criteria were removed (Additional File 1: Appendix K). To aid interpretation, we also ranked studies by precision (inverse of the standard error of 95% PI or 95% CI) and then by effect size (Additional File 1: Appendix L). We observed a moderate but highly significant positive correlation between rankings (Kendall’s rank correlation tau = 0.31, *P* value = 1.32e−07 for GRADE and SE 95% PI rank). Although GRADE criteria are limited by rigid thresholds, these analyses suggest they are relatively robust and considerably improve the classification of evidence for quality and strength of recommendations.

### Quality assessment

The 38 meta-analyses categorised as providing either highly suggestive (*N* = 26) or strong evidence (*N* = 12) arose from 30 individually published studies. Of the 26 highly suggestive meta-analyses, four were from studies ranked as critically low quality (15%), five as low quality (19%), seventeen as moderate quality (65%) and one as high quality (4%). The twelve meta-analyses categorised as providing strong evidence were ranked as moderate quality (*N* = 8, 67%), low quality (*N* = 3, 25%) and critically low quality (*N* = 1, 8%) (Additional File 1: Appendix M). From the 86 review studies included, we found that 10 (12%) did not assess the risk of bias in component studies, 7 (8%) used QUIPS and the remainder used various other tools (Additional File 1: Appendix N*,* Table 1). We assessed the risk of bias in the 42 unique component studies included from the seven review studies graded as strong and considered most (*N* = 37) to be at low risk of bias (Additional File 1: Appendix N*,* Table 2).

## Discussion

A total of 204 associations between elevated NLR or TAN and cancer outcomes were reviewed to assess the strength of the evidence supporting them. Twelve associations were supported by strong evidence. Although the studies included showed strong consistency in direction of effect and moderate effect sizes, we detected poor reproducibility of findings overall as well as evidence of heterogeneity and small-study effects.

### Risk of elevated neutrophil to lymphocyte ratio

Previous studies have documented the prognostic role of neutrophils, particularly the NLR, and their link with poor outcomes for many cancer sites [10]. We found that 92% of the included meta-analyses had a significant HR through random effects estimates (*P* < 0.05). However, a cautious interpretation is required due to the presence of heterogeneity and small study effect biases. Associations supported by strong evidence included elevated NLR in urinary (prostate and non-muscle-invasive bladder) and nasopharyngeal cancers, amongst others. Future research should assess the association between the NLR and prognosis in oral and respiratory cancers by environmental exposures, such as smoking status, to ensure these do not confound associations.

CLM represents a unique case where metastasis has already occurred and may present a link between elevated NLR and poor prognosis in metastasised cancers. It is interesting to note that the associations between NLR and OS and PFS in colorectal cancer also included patients with different stages of metastasis according to the tumour node metastasis (TNM) system. These associations were supported by highly suggestive and suggestive evidence, respectively, and emerged from a study of moderate quality. To determine the impact of advanced cancer stages, metastasis and subtypes of cancer on the association between the NLR and cancer outcomes, future studies should consider these additional factors carefully when assessing the prognostic potential of the NLR.

### Risk of tumour-associated neutrophils

Although previous studies suggest a link between TAN and the progression of cancer in the tumour microenvironment [11, 13, 130], the evidence for their association was mostly classified as weak or uncertain. The significance of these associations may have been limited by small sample size, and it is important to note that all meta-analyses considering TAN arose from a single publication and may be subject to the same limitations [55]. The association between TAN and cancer outcomes holds potential for a strong association due to the large effect size observed in this review and the plausibility of the biological mechanism behind the relationship [8]. However, a recently published individual study on this association found that high levels of TAN had a protective effect in cancer [131], indicating that additional research is required.

### Strengths

A key strength of this study comes from the use of umbrella review methodology which only includes meta-analyses as evidence for quantitative data analyses [14]. The use of meta-analyses ensures that effect size estimates are a balanced representation of the available evidence, as demonstrated by the sensitivity analysis of the association between elevated NLR and OS in rectal cancer from Dong et al. (Additional File 1: Appendix O) [114]. When an extreme outlier detected in this meta-analysis was removed from the analysis, the random effects estimate was not considerably altered due to the small weighting given to studies with large variances.

Although there are considerable differences between the included meta-analyses in terms of cancer site, stage and treatment, all but one of the HR estimates reported in these meta-analyses were in the same direction of effect. This finding suggests consistency in the relationship between neutrophil biomarkers and poor outcomes across a wide spectrum of cancer diagnoses.

### Limitations

Only 39% of the identified meta-analyses were eligible for inclusion and 16% of the identified meta-analyses were excluded because they did not include sufficient data to be reproduced. Furthermore, we were unable to reproduce 43% of the included studies within 0.01 of the reported HR, highlighting issues with transparency and reproducibility of findings in epidemiologic research [132]. Umbrella reviews also fail to include evidence published in individual studies after the last published meta-analyses. However, all the included meta-analyses in our review were recently published, with the oldest published in 2014, so the exclusion of individual studies in our case may be minimal. This exclusion of individual studies is of greatest concern in the association between TAN and cancer outcomes, due to the availability of a single systematic review, which yielded all nine meta-analysis considered.

Umbrella reviews are reliant on the quality of the included meta-analyses. This methodological limitation is of concern since 42% of the studies which yielded meta-analyses with highly suggestive or strong evidence were ranked as low or critically low quality by AMSTAR 2 (Additional File 1: Appendix M). The application of AMSTAR 2 for the purpose of ranking the quality of systematic reviews of prognostic studies may also present a limitation in itself, as there are currently no tools designed specifically for this. There is also some concern over consistency, since meta-analyses aggregated the results of individual studies which categorised patients’ NLR or TAN levels as high or low using different cut-off values (Additional File 1: Appendix G) and utilised different analysis methods which adjusted for a range of confounders (Additional File 1: Appendix F). Due to heterogeneity in these values, it is not possible to establish a dose-response relationship between NLR and cancer prognosis.

The assessment of heterogeneity using Cochran’s Q test and the *I*^2^ statistic is problematic with varying sizes of component studies. Although some studies recommend the interpretation of the *I*^2^ value with 95% confidence intervals [36], we did not utilise them as grading criteria since 168 of our included meta-analyses (82%) include less than 15 studies [36]. Cochran’s Q test also has weak power when there are few studies and excess power in detecting heterogeneity when studies are numerous, both of which are complications in this study [22]. The sensitivity analysis removing *I*^2^ and Cochran’s Q test was conducted to assess the potential impact of these limitations, and the resulting reclassification of only a single association suggests the GRADE criteria are relatively robust.

### Causal association

This umbrella review does not address causality directly and cannot determine whether the association between neutrophils and poor prognosis in cancer is causal or due to confounding or reverse causation [133, 134]. Previous studies have highlighted the paradoxical role of neutrophils in both tumour progression and suppression [13]. Our study suggests that the overall effect of high NLR could be tumourigenic in certain cases, but further work is required to assess this.

Our study supports the relationship between elevated NLR and poor outcomes in cancer in terms of effect size and consistency of findings. We cannot address temporality as the studies included measured biomarkers before the initiation of treatment but after diagnosis. The biological mechanisms behind inflammation and cancer progression could suggest temporality, as elevated NLR and TAN are proposed to promote increased cell proliferation, angiogenesis and risk of metastasis as contributors to poor prognosis [5, 13, 135].

### Clinical significance and future research

Future research should focus on strengthening the current evidence base for specific cancers which displayed suggestive and highly suggestive associations, addressing heterogeneity and small-study issues. Unveiling a causal association between neutrophils and cancer outcomes could lead to cancer treatments which involve neutrophils as a therapeutic target and validate the NLR as a prognostic biomarker in cancer. A causal association between neutrophils and poor prognosis could give further insight into experimental therapy which lowers neutrophil counts in the body to improve outcomes in cancer [136–139]. Sub-group analysis suggested that the magnitude and direction of effect of NLR on cancer outcomes was robust to adjustment for additional prognostic factors. Regardless, consensus on a minimum set of covariates to adjust for is needed. Furthermore, the dose response relationship between NLR and prognosis remains unclear. Within the included papers, only two of the 86 conducted a dose response analysis [80, 140]. Future work should consider whether the association is linear or has threshold effects. In light of the variation observed in meta-regressions of NLR cut-off and effect size, identification of a clinically relevant NLR cut-off could be specific to different cancer sites and may be affected by treatment. Future systematic reviews should consider performing individual patient data (IPD) meta-analyses to allow for the assessment of NLR values on a continuous scale.

## Conclusion

The quantitative evidence presented suggests an association between elevated NLR and poor outcomes in cancer patients across a wide spectrum of diagnoses, stages of disease and courses of treatment. The evidence is strongest for associations between NLR and OS in prostate, non-muscle-invasive bladder and nasopharyngeal cancer, amongst others. Overall however, and in particular for associations between TAN and poor prognosis in cancer patients, evidence is limited by study quality, heterogeneity and small-study effects. Further research is required to overcome the limitations of the existing evidence.

## Supplementary information

**Additional file 1.** Appendix A. Search strategy. Appendix B. 517 extracted meta-analyses with exclusions identified. Appendix C. Assessment of duplicate meta-analyses. Appendix D. Description of 87 meta-analyses with poor reproducibility. Appendix E. Heterogeneity and small-study effects in included meta-analyses. Appendix F: Results of subgroup analysis of adjusted versus unadjusted effect estimates. Appendix G. Meta-regression results of NLR cut-off values. Appendix H. Excess significance in included meta-analyses. Appendix I: Grading of evidence (GRADE criteria). Appendix J: Effect size upgrading of GRADE. Appendix K. Sensitivity analysis of GRADE. Appendix L. Ranking based on precision versus effect size. Appendix M. AMSTAR 2 Quality Rating of Meta-analyses with Highly Suggestive or Strong Evidence. Appendix N. Assessment of quality of component studies. Appendix O: Sensitivity analysis of overall survival in rectal cancer.

**Additional file 2.** Supplementary Figs. 1–205.

**Additional file 3.** Supplementary Table 1.

## Data Availability

The datasets generated and analysed during the current study are available in the “Neutrophils-and-Cancer-Prognosis” repository, at https://github.com/megcupp. All authors had full access to all of the data (including statistical reports and tables) in the study and can take responsibility for the integrity of the data and the accuracy of the data analysis. The corresponding author had final responsibility for the decision to submit for publication.

## Abbreviations

NLR: Neutrophil to lymphocyte ratio
TAN: Tumour-associated neutrophils
HR: Hazard ratio
OS: Overall survival
CSS: Cancer-specific survival
PFS: Progression-free survival
DFS: Disease-free survival
RFS: Reoccurrence-free survival
REML: Restricted maximum likelihood
PI: Prediction intervals
TES: Test for excess significance
GRADE: Grading of Recommendations, Assessment, Development and Evaluations
QUIPS: Quality in Prognostic Studies
ICI: Immune checkpoint inhibitors
UTR: Urothelial transurethral resection
VEGFR: Anti-vascular endothelial growth factor receptors
SR: Surgical resection
CLM: Colorectal liver metastasis
TKI: Tyrosine kinase inhibitor
NSCLC: Non-small cell lung cancer
TNM: Tumour node metastasis
