# Supplementary material for: Neutrophil to lymphocyte ratio and cancer prognosis: an umbrella review of systematic reviews and meta-analyses of observational studies
Source: BMC Med. 2020 Nov 20;18:360. doi: 10.1186/s12916-020-01817-1 (PMC7678319; doi:10.1186/s12916-020-01817-1)
Supplement: Supplementary file 1 — Additional file 1. Appendix A. Search strategy. Appendix B. 517 extracted meta-analyses with exclusions identified. Appendix C. Assessment of duplicate meta-analyses. Appendix D. Description of 87 meta-analyses with poor reproducibility. Appendix E. Heterogeneity and small-study effects in included meta-analyses. Appendix F: Results of subgroup analysis of adjusted versus unadjusted effect estimates. Appendix G. Meta-regression results of NLR cut-off values. Appendix H. Excess significance in included meta-analyses. Appendix I: Grading of evidence (GRADE criteria). Appendix J: Effect size upgrading of GRADE. Appendix K. Sensitivity analysis of GRADE. Appendix L. Ranking based on precision versus effect size. Appendix M. AMSTAR 2 Quality Rating of Meta-analyses with Highly Suggestive or Strong Evidence. Appendix N. Assessment of quality of component studies. Appendix O: Sensitivity analysis of overall survival in rectal cancer. [file 12916_2020_1817_MOESM1_ESM.pdf]

Appendix A: Search Strategy

Concept 1
Neutrophils, Neutrophil to Lymphocyte Ratio

Concept 2
Cancer, Carcinoma, Neoplasia, Tumour, Neoplasm

Concept 3
Systematic reviews

EMBASE CLASSIC+EMBASE
1947 to 2020 May 29:
Searched on 30 May 2020 Using search filter "Embase systematic review strategy" from BMJ Clinical Evidence.[1]
Number of results: 1099.

1	(neutrophil* or NLR).mp.
2	exp neutrophil/ or exp neutrophil count/
3	1 or 2
4	(cancer or carcinoma or neoplasia or tumor or tumour or neoplasm*).mp.
5	exp neoplasm/
6	4 or 5
7	3 and 6
8	exp review/
9	(literature adj3 review$).ti,ab.
10	exp meta analysis/
11	exp "Systematic Review"/
12	8 or 9 or 10 or 11
13	(medline or medlars or embase or pubmed or cinahl or amed or psychlit or psyclit or psychinfo or psycinfo or scisearch or cochrane).ti,ab.
14	RETRACTED ARTICLE/
15	13 or 14
16	12 and 15
17	(systematic$ adj2 (review$ or overview)).ti,ab.
18	(meta?anal$ or meta anal$ or meta-anal$ or metaanal$ or metanal$).ti,ab.
19	16 or 17 or 18
20	7 and 19

OVID MEDLINE® EPUB AHEAD OF PRINT, IN-PROCESS & OTHER NON-INDEXED CITATIONS, OVID MEDLINE® DAILY AND OVID MEDLINE®

1946 to 2020 May 29:
Searched on 30 May 2020 Using search filter "Embase systematic review strategy" from BMJ Clinical Evidence.
Number of results: 588.

1	(neutrophil* or NLR).mp.
2	exp Neutrophils/
3	1 or 2
4	(cancer or carcinoma or neoplasia or tumor or tumour or neoplasm*).mp.
5	exp Neoplasms/
6	4 or 5
7	3 and 6
8	(review or review,tutorial or review, academic).pt.
9	(medline or medlars or embase or pubmed or cochrane).tw,sh.
10	(scisearch or psychinfo or psycinfo).tw,sh.
11	(psychlit or psyclit).tw,sh.
12	cinahl.tw,sh.
13	((hand adj2 search$) or (manual$ adj2 search$)).tw,sh.
14	(electronic database$ or bibliographic database$ or computeri?ed database$ or online database$).tw,sh.
15	(pooling or pooled or mantel haenszel).tw,sh.
16	(peto or dersimonian or der simonian or fixed effect).tw,sh.
17	(retraction of publication or retracted publication).pt.
18	9 or 10 or 11 or 12 or 13 or 14 or 15 or 16 or 17
19	8 and 18
20	meta-analysis.pt.
21	exp Meta-Analysis/
22	(meta-analys$ or meta analys$ or metaanalys$).tw,sh.
23	(systematic$ adj5 review$).tw,sh.
24	(systematic$ adj5 overview$).tw,sh.
25	(quantitativ$ adj5 review$).tw,sh.
26	(quantitativ$ adj5 overview$).tw,sh.
27	(quantitativ$ adj5 synthesis$).tw,sh.
28	(methodologic$ adj5 review$).tw,sh.
29	(methodologic$ adj5 overview$).tw,sh.
30	(integrative research review$ or research integration).tw.
31	20 or 21 or 22 or 23 or 24 or 25 or 26 or 27 or 28 or 29 or 30
32	19 or 31
33	7 and 32

COCHRANE
Issue 5 or 12, May 2020 (Only reviewing results from Cochrane Database of Systematic Reviews)

Searched on 30 May 2020. Number of results: 180.

1	neutrophil* or NLR
2	MeSH descriptor: [Neutrophils] explode all trees
3	#1 or #2
4	cancer or carcinoma or neoplasia or tumor or tumour or neoplasm*
5	MeSH descriptor: [Neoplasms] explode all trees
6	#4 or #5
7	#3 and #6

References

1. BMJ Clinical Evidence. Study Design Search Filters [Internet]. *BMJ*. Available from: http://clinicalevidence.bmj.com/x/set/static/ebm/learn/665076.html

Appendix B: 517 extracted meta-analyses with exclusions identified

Author	Year	Biomarker	Cancer site	meta-analysis metric	N	Sample size (HNLR/LNLR)	Pooled HR	Notes
Boissier R	2017	NLR	Renal Localised	OS	3	1168 (403/651)*	1·46 (0·85-2·50)	
Boissier R	2017	NLR	Renal Advanced	OS	7	1304 (331/337)*	1·55 (1·36-1·76)	
Boissier R	2017	NLR	Renal Localised	RFS	6	1962 (487/817)*	1·63 (1·15-2·29)	
Boissier R	2017	NLR	Renal Advanced	PFS	3	306 (143/163)	3·19 (2·23-4·57)	
Bowen RC	2017	NLR	Hepatocellular	OS	NA	8337	1·40 (1·30-1·52)	Does not include complete data for analysis
Bowen RC	2017	NLR	Hepatocellular	DFS	NA	3802	1·71 (1·40-2·09)	Does not include complete data for analysis
Bowen RC	2017	NLR	Hepatocellular	PFS	NA	266	1·36 (1·01-1·84)	Does not include complete data for analysis
Bowen RC	2017	NLR	Pancreatic	OS	NA	4381	1·90 (1·43-2·52)	Does not include complete data for analysis
Bowen RC	2017	NLR	Pancreatic	CSS	NA	261	2·53 (1·64-3·91)	Does not include complete data for analysis
Bowen RC	2017	NLR	Pancreatic	DFS	NA	460	2·19 (0·51-9·34)	Does not include complete data for analysis
Bowen RC	2017	NLR	Gastric	OS	NA	10559	1·47 (1·25-1·72)	Does not include complete data for analysis
Bowen RC	2017	NLR	Gastric	DFS	NA	806	1·42 (1·11-1·81)	Does not include complete data for analysis
Bowen RC	2017	NLR	Gastric	PFS	NA	268	1·48 (1·15-1·89)	Does not include complete data for analysis
Bowen RC	2017	NLR	Gastric	CSS	NA	389	1·53 (1·11-2·11)	Does not include complete data for analysis
Bowen RC	2017	NLR	Colorectal	OS	NA	9481	2·04 (1·79-2·23)	Does not include complete data for analysis
Bowen RC	2017	NLR	Colorectal	DFS	NA	7131	1·75 (1·44-2·12)	Does not include complete data for analysis
Bowen RC	2017	NLR	Colorectal	PFS	NA	541	2·02 (1·43-2·84)	Does not include complete data for analysis
Bowen RC	2017	NLR	Colorectal	CSS	NA	2639	2·23 (1·71-2·92)	Does not include complete data for analysis
Bowen RC	2017	NLR	Cholangiocarcinoma	OS	NA	1187	1·53 (1·14-2·05)	Does not include complete data for analysis
Bowen RC	2017	NLR	Cholangiocarcinoma	DFS	NA	102	2·26 (1·24-4·11)	Does not include complete data for analysis
Bowen RC	2017	NLR	Oesophageal	PFS	NA	138	1·80 (1·05-3·08)	Does not include complete data for analysis
Bowen RC	2017	NLR	Oesophageal	CSS	NA	1297	1·32 (1·03-1·70)	Does not include complete data for analysis
Bowen RC	2017	NLR	Oesophageal	DFS	NA	1580	1·67 (1·18-2·38)	Does not include complete data for analysis
Bowen RC	2017	NLR	Oesophageal	OS	NA	2939	1·61 (1·32-1·95)	Does not include complete data for analysis
Bowen RC	2017	NLR	Gastrointestinal	OS	NA	36884	1·63 (1·53-1·73)	Does not include complete data for analysis
Bowen RC	2017	NLR	Gastrointestinal	DFS	NA	14201	1·70 (1·52-1·91)	Does not include complete data for analysis
Bowen RC	2017	NLR	Gastrointestinal	PFS	NA	1213	1·64 (1·36-1·97)	Does not include complete data for analysis
Bowen RC	2017	NLR	Gastrointestinal	CSS	NA	4586	1·83 (1·50-2·23)	Does not include complete data for analysis

Bowen RC	2017	NLR	Gastrointestinal Stromal Tumour	DFS	NA	300	2·16 (1·19-3·94)	Does not include complete data for analysis
Cao J	2016	NLR	Prostate	OS	16	15298	1·41 (1·26-1·55)	
Cao J	2016	NLR	Prostate Castration Resistant	PFS	6	1629	1·42 (1·23-1·61)	
Cao J	2016	NLR	Prostate Localised	RFS	7	11745	1·38 (1·01-1·75)	
Chen	2017	NLR	MPM	OS	11	1378	1·48 (1·16-1·89)	
Chen G	2018	NLR	Ovarian Cancer	OS	11	3702	1·41 (1·11-1·79)	
Chen G	2018	NLR	Ovarian Cancer	PFS	8	2991	1·52 (1·19-1·95)	
Chen J	2015	NLR	Gastric	OS	9	3723 (1357/2366)	2·16 (1·86-2·51)	
Chen J	2015	NLR	Gastric	PFS	2	282 (113/169)	2·78 (1·95-3·96)	
Chen J	2015	NLR	Breast	OS	5	162172	2·28 (1·08-4·80)	
Chen J	2015	NLR	Breast	DFS	4	2764	1·38 (1·09-1·74)	
Chen J	2018	NLR	Breast (Metastasis)	OS	2	858	2·81 (2·13-3·71)	
Chen J	2018	NLR	Breast (No Metastasis)	OS	2	488	1·45 (0·37-5·66)	
Chen J	2018	NLR	Breast (Metastasis)	DFS	3	1003	2·16 (1·67-2·80)	
Chen J	2018	NLR	Breast (No Metastasis)	DFS	2	466	1·34 (0·50-3·58)	
Chen S	2017	NLR	Ovarian Cancer	OS	11	2017*	1·51 (1·03-2·23)	
Chen S	2017	NLR	Ovarian Cancer	PFS	7	851*	1·55 (1·15-2·08)	
Cheng H	2015	NLR	Pancreatic	OS	9	2035 (958/1077)	1·59 (1·41-1·79)	
Ding Y	2018	NLR	Melanoma	OS	10	3207	2·23 (1·64-3·04)	
Ding Y	2018	NLR	Melanoma	PFS	6	752	2·19 (1·78-2·69)	
Dolan R	2017	NLR	Operable Cancer	OS	120	68603 (18667/49936)	1·73 (1·56-1·91)	
Dolan R	2017	NLR	Operable Cancer	CSS	41	21175 (4617/16558)	1·32 (1·24-1·41)	
Dolan R	2017	NLR	Operable Colorectal	OS	8	4204 (825/3379)	1·8 (1·37-2·37)	
Dong YW	2016	NLR	Rectal	OS	6	857	13·41 (4·90-36·72)	
Dong YW	2016	NLR	Rectal	DFS	3	403	4·37 (2·33-8·19)	
Dong YW	2016	NLR	Rectal	RFS	3	433	3·64 (1·88-7·05)	
Ethier JL	2017	NLR	Gynecologic	OS	27	10530	1·65 (1·44-1·89)	
Ethier JL	2017	NLR	Gynecologic	EFS	18	5641	1·57 (1·35-1·82)	Does not assess outcomes of interest
Ethier JL	2017	NLR	Breast	OS	13	8015	2·56 (1·96-3·35)	
Ethier JL	2017	NLR	Breast	DFS	9	4864	1·74 (1·47-2·07)	
Gu X	2016	NLR	Prostate	OS	11	14250	1·38 (1·22-1·56)	

Gu X	2016	NLR	Prostate	RFS/PFS	9	12541	1·24 (1·05-1·46)	Assessed two outcomes within a single analysis
Gu XB	2015	NLR	NSCLC	OS	13	3544	1·70 (1·39-2·09)	
Gu XB	2015	NLR	NSCLC	PFS	10	2623	1·63 (1·27-2·09)	
Hu K	2015	NLR	Renal	OS	13	3034	1·82 (1·51-2·19)	
Hu K	2015	NLR	Renal	RFS/PFS	10	2101	2·18 (1·75-2·71)	Assessed two outcomes within a single analysis
Huang QT	2017	NLR	Cervical	OS	7	2699	1·88 (1·30-2·73)	
Huang QT	2017	NLR	Cervical	PFS	5	2607	1·65 (1·18-2·29)	
Huang QT	2017	NLR	Ovarian	OS	12	3935	1·69 (1·29-2·22)	
Huang QT	2017	NLR	Ovarian	PFS	8	2152	1·63 (1·27-2·09)	
Huang Y	2017	NLR	Oesophageal	OS	9	2513	1·31 (1·16-1·48)	
Huang Y	2017	NLR	Oesophageal	PFS	2	253	1·22 (0·90-1·66)	
Huang Y	2017	NLR	Oesophageal	DFS	2	535	1·27 (1·01-1·59)	
Jiang T	2018	NLR	Anti-VEGFR	OS	14	2918	2·04 (1·61-2·60)	
Jiang T	2018	NLR	Immune checkpoint blockage	OS	4	1291	2·16 (1·86-2·51)	
Jiang T	2018	NLR	Other	OS	2	261	1·63 (0·89-2·99)	Does not assess specific cancer site or treatment
Jiang T	2018	NLR	Advanced with immunotherapy	OS	23	4355	1·98 (1·66-2·36)	
Jiang T	2018	NLR	Advanced with immunotherapy	PFS	16	2318	1·78 (1·48-2·15)	
Li MX	2014	NLR	Colorectal	OS	16	6291 (2042/4134)*	1·81 (1·50-1·20)	
Li MX	2014	NLR	Colorectal	PFS	10	2718 (617/1856)*	2·10 (1·55-2·84)	
Li X	2018	NLR	Neoadjuvant Chemotherapy	OS	18	3282	1·58 (1·34-1·86	
Li X	2018	NLR	Neoadjuvant Chemotherapy	CSS	5	971	2·22 (1·32-3·74)	
Li X	2018	NLR	Neoadjuvant Chemotherapy	DFS	11	1719	1·32 (1·10-1·59)	
Li X	2018	NLR	Neoadjuvant Chemotherapy	RFS	7	775	1·90 (1·50-2·40)	
Li X	2017	NLR	Upper Urinary and Bladder	OS	17	4432	1·72 (1·45-2·05)	
Li X	2017	NLR	Upper Urinary and Bladder	PFS	19	8182	1·68 (1·44-1·96)	
Li X	2017	NLR	Upper Urinary and Bladder	CSS	17	7645	1·64 (1·39-1·93)	
Li Y	2016	NLR	Soft Tissue Sarcoma	OS	3	494	3·75 (1·24-11·37)	

Li Y	2016	NLR	Soft Tissue Sarcoma	DFS/PFS	3	514	2·43 (0·84-7·05)	Assessed two outcomes within a single analysis
Liu G	2018	NLR	Soft Tissue Sarcoma	OS	13	2658*	1·59 (1·28-1·97)	
Liu G	2018	NLR	Soft Tissue Sarcoma	DFS/PFS	NA	NA	1·28 (1·12-1·47)	Assessed two outcomes within a single analysis
Liu X	2017	NLR	Breast	OS	11	6836	1·87 (1·41-2·48)	
Liu X	2017	NLR	Breast	DFS	12	5523	1·72 (1·30-2·27)	
Liu X	2017	NLR	Breast	CSS	4	4186	2·09 (1·04-4·21)	
Liu X	2017	NLR	Triple-negative breast cancer	OS	4	608	2·58 (1·63-4·06)	
Liu X	2017	NLR	Triple-negative breast cancer	DFS	4	461	3·51 (1·97-6·24)	
Lucca I	2015	NLR	Urothelial Carcinoma	OS	4	5452	1·11 (1·01-1·22)	
Lucca I	2015	NLR	Urothelial Carcinoma	CSS	5	5641	1·21 (1·07-1·37)	
Luo XF	2018	NLR	Gastrointestinal stromal	OS	2	278	1·74 (0·63-4·84)	
Luo XF	2018	NLR	Gastrointestinal stromal	RFS/DFS	7	NA	2·18 (1·30-3·67)	Assessed two outcomes within a single analysis
Luo Y	2015	NLR	Upper Urinary	CSS	4	1218	2·52 (1·41-4·52)	
Luo Y	2015	NLR	Upper Urinary	MFS	1	234	2·47 (1·16-5·29)	Does not assess outcomes of interest
Luo Y	2015	NLR	Upper Urinary	PFS	1	460	1·70 (1·14-2·56)	Includes only one component study
Luo Y	2015	NLR	Upper Urinary	RFS	2	802	1·47 (1·11-1·95)	
Luo Y	2015	NLR	Upper Urinary	OS	1	182	2·48 (1·31-4·70)	Includes only one component study
Luo Y	2015	NLR	Renal	CSS	4	1728	1·38 (0·96-1·99)	
Luo Y	2015	NLR	Renal	MFS	2	1505	1·60 (1·29-1·98)	Does not assess outcomes of interest
Luo Y	2015	NLR	Renal	PFS	7	753	1·85 (1·24-2·77)	
Luo Y	2015	NLR	Renal	RFS	3	700	1·97 (1·37-2·84)	
Luo Y	2015	NLR	Renal	OS	14	3373	1·79 (1·61-2·00)	
Luo Y	2015	NLR	Bladder	CSS	5	1927	1·70 (1·45-1·99)	
Luo Y	2015	NLR	Bladder	PFS	1	107	3·52 (1·33-9·33)	Includes only one component study
Luo Y	2015	NLR	Bladder	RFS	2	531	1·55 (1·21-2·00)	
Luo Y	2015	NLR	Bladder	OS	4	1810	1·68 (1·45-1·94)	
Luo Y	2015	NLR	Prostate	PFS	2	881	1·29 (1·04-1·59)	
Luo Y	2015	NLR	Prostate	OS	5	2310	1·44 (1·28-1·62)	
Malietzis G	2014	NLR	Colorectal	OS	13	3455 (1044/2411)*	2·08 (1·64-2·64)	
Malietzis G	2014	NLR	Colorectal with Surgical Bowel Resection	DFS	7	2408 (797/1611)*	2·10 (1·50-2·93)	

Malietzis G	2014	NLR	Colorectal with Palliative Chemotherapy	DFS	3	515 (148/367)	1·53 (1·23-1·90)	
Marchioni M	2017	NLR	Urothelial Carcinoma	OS	2	397	1·97 (1·27-3·04)	
Marchioni M	2017	NLR	Urothelial Carcinoma	RFS	4	1313	1·52 (1·19-1·96)	
Marchioni M	2017	NLR	Urothelial Carcinoma	CSS	6	1710	1·25 (0·29-5·41)	
Mascarella M	2018	NLR	Hypopharynx	OS	3	305	2·36 (1·54-3·61)	
Mascarella M	2018	NLR	Larynx	OS	6	1017	1·55 (1·26-1·92)	
Mascarella M	2018	NLR	Nasopharyngeal	OS	3	1890	1·66 (1·35-2·04)	
Mascarella M	2018	NLR	Oral cavity	OS	3	1899	1·56 (1·53-2·07)	
Mascarella M	2018	NLR	Head and Neck	OS	24	6917	1·78 (1·53-2·07)	
Mei Z	2017	NLR	Multiple	OS	65	18262*	1·70 (1·57-1·84)	
Mei Z	2017	NLR	Multiple	PFS	23	5607	1·61 (1·42-1·82)	
Mei Z	2017	NLR	Hepatocellular	OS	2	524	1·91 (1·55-2·37)	
Mei Z	2017	NLR	Pancreatic	OS	9	1983	2·04 (1·50-2·75)	
Mei Z	2017	NLR	Gastric	OS	4	1715	1·69 (1·41-2·03)	
Mei Z	2017	NLR	Colorectal	OS	15	4787	1·89 (1·56-2·30)	
Mei Z	2017	NLR	Colorectal	PFS	5	549	1·91 (1·31-2·79)	
Mei Z	2017	NLR	NSCLC	OS	7	1386	1·20 (1·08-1·34)	
Mei Z	2017	NLR	NSCLC	PFS	5	1140	1·38 (1·06-1·80)	
Mei Z	2017	NLR	Renal	OS	9	1689	1·91 (1·58-2·32)	
Mei Z	2017	NLR	Renal	PFS	5	930	1·49 (1·18-1·89)	
Mei Z	2017	NLR	Prostate	OS	5	2019	1·43 (1·26-1·62)	
Mei Z	2017	NLR	Other	OS	12	NA	1·53 (1·41-1·67)	Does not assess specific cancer site or treatment
Mei Z	2017	NLR	Other	PFS	8	NA	166 (1·44-1·90)	Does not assess specific cancer site or treatment
Mellor K	2018	NLR	potentially curative gastrectomy	OS	5	2787 (1338/1449)	2·31 (1·40-3·83)	
Mellor K	2018	NLR	potentially curative gastrectomy	DFS	3	820 (156/664)	2·72 (1·14-6·54)	
Min GT	2018	NLR	HCC	OS	45	10491 (2594/5312)*	1·52 (1·39-1·67)	
Min GT	2018	NLR	HCC	DFS	17	3881 (919/1729)*	1·71 (1·39-2·11)	
Min GT	2018	NLR	HCC	RFS	16	5037 (1470/3188)*	1·84 (1·48-2·30)	
Mowbray N	2018	NLR	Pancreatic (Resection)	OS	8	1519	1·77 (1·45-2·15)	
Mu S	2018	NLR	Large B	OS	10	NA	1·83 (1·24-2·70)	
Mu S	2018	NLR	Large B	PFS	9	NA	1·59 (1·12-2·25)	

Na N	2016	NLR	Renal	OS	8	1220	1·93 (1·35-2·77)	
Na N	2016	NLR	Renal	PFS	8	1115*	2·12 (1·42-3·17)	
Oh D	2018	NLR	Pancreatic (Chemotherapy)	OS	13	NA	2·04 (1·58-2·64)	Does not include complete data for analysis
Oh D	2018	NLR	Pancreatic (Chemradiation)	OS	2	NA	0·92 (0·27-3·16)	Does not include complete data for analysis
Oh D	2018	NLR	Pancreatic (Mixed Treatment)	OS	18	NA	1·67 (1·39-2·01)	Does not include complete data for analysis
Oh D	2018	NLR	Pancreatic	OS	35	NA	1·74 (1·50-2·01)	Does not include complete data for analysis
Paramanathan A	2014	NLR	Colorectal Liver Metastasis Resection of Solid Tumours	OS	3	814	2·28 (1·77-2·93)	
Paramanathan A	2014	NLR	Hepatocellular Resection of Solid Tumours	OS	4	566	3·52 (2·34-5·28)	
Paramanathan A	2014	NLR	Gastric Resection of Solid Tumours	OS	7	2334	1·97 (1·41-2·76)	
Paramanathan A	2014	NLR	Colorectal Solid Tumours	OS	6	1961	1·65 (1·21-2·26)	
Paramanathan A	2014	NLR	Oesophageal Solid Tumours	OS	3	559	1·48 (0·91-2·42)	
Paramanathan A	2014	NLR	Ovarian Solid Tumours	OS	2	321	2·02 (0·18-22·57)	
Paramanathan A	2014	NLR	Multiple	OS	36	8626	1·92 (1·64-2·24)	
Paramanathan A	2014	NLR	Multiple	DFS	20	13065	1·99 (1·80-2·20)	
Paramanathan A	2014	NLR	Other Solid Tumours	OS	11	2071*	1·71 (1·35-2·15)	Does not assess specific cancer site or treatment
Peng B	2015	NLR	NSCLC	OS	11	2274	1·43 (1·25-1·64)	
Peng B	2015	NLR	NSCLC	PFS	5	1052	1·37 (1·07-1·74)	
Qi X	2016	NLR	Hepatocellular	OS	38	9127 (3025/5922)	1·80 (1·59-2·04)	
Qi X	2016	NLR	Hepatocellular	RFS/DFS	20	4093 (1289/2804)	2·23 (1·80-2·79)	Assessed two outcomes within a single analysis
Sacdalan D	2018	NLR	Gentiourinary	PFS	1	NA	NA	Includes only one component study
Sacdalan D	2018	NLR	Gentiourinary	OS	1	NA	NA	Includes only one component study
Sacdalan D	2018	NLR	NSCLC (immune inhibitor)	PFS	2	227	1·56 (1·14-2·13)	
Sacdalan D	2018	NLR	NSCLC (immune inhibitor)	OS	3	342	1·63 (0·92-2·88)	
Sacdalan D	2018	NLR	Melanoma (immune inhibitor)	PFS	2	281	1·73 (1·33-2·25)	
Sacdalan D	2018	NLR	Melanoma (immune inhibitor)	OS	3	339	2·18 (1·66-2·84)	
Sacdalan D	2018	NLR	Composite endpoints (immune inhibitor)	PFS	5	592	1·66 (1·38-2·01)	

Sacdalan D	2018	NLR	Composite endpoints (immune inhibitor)	OS	7	765	1·92 (1·29-2·87)	
Sacdalan D	2018	NLR	Ipilimumab	PFS	1	NA	NA	Includes only one component study
Sacdalan D	2018	NLR	Ipilimumab	OS	2	255	2·05 (1·54-2·74)	
Sacdalan D	2018	NLR	Nivolumab	PFS	1	NA	NA	Includes only one component study
Sacdalan D	2018	NLR	Nivolumab	OS	2	290	1·43 (0·77-2·69)	
Sacdalan D	2018	NLR	ICI	PFS	3	220	1·76 (1·25-2·47)	
Sacdalan D	2018	NLR	ICI	OS	3	220	2·52 (1·58-4·02)	
Semeniuk-Wojtas A	2018	NLR	Renal (TKI)	OS	7	NA	2·14 (1·67-2·73)	
Semeniuk-Wojtas A	2018	NLR	Renal (TKI)	PFS	6	NA	2·01 (1·27-3·18)	
Shen M	2014	Intratumoral Neutrophils	Multiple	OS	16	3335	1·66 (1·37-2·01)	
Shen M	2014	Peritumoral Neutrophils	Multiple	OS	2	535	1·66 (0·64-4·32)	
Shen M	2014	Stromal Neutrophils	Multiple	OS	2	535	1·10 (0·76-1·61)	
Shen M	2014	Intratumoral Neutrophils	Multiple	CSS	3	437	3·36 (2·08-5·42)	
Shen M	2014	Intratumoral Neutrophils	Multiple	RFS/DFS	10	2139	1·68 (1·36-2·07)	Assessed two outcomes within a single analysis
Shen M	2014	Peritumoral Neutrophils	Multiple	RFS/DFS	3	636	1·80 (0·96-3·37)	Assessed two outcomes within a single analysis
Shen M	2014	Stromal Neutrophils	Multiple	RFS/DFS	3	636	1·27 (0·75-2·16)	Assessed two outcomes within a single analysis
Shen M	2014	Intratumoral Neutrophils	HCC and ICC	OS	4	846	1·80 (1·3-2·43)	
Shen M	2014	Intratumoral Neutrophils	HCC and ICC	RFS/DFS	4	846	1·58 (1·33-1·88)	Assessed two outcomes within a single analysis
Shen M	2014	Intratumoral Neutrophils	Gastric	OS	2	388	1·20 (0·50-2·89)	
Shen M	2014	Intratumoral Neutrophils	NSCLC	OS	2	967	1·16 (1·00-1·35)	
Shen M	2014	Intratumoral Neutrophils	Renal	OS	2	206	2·69 (1·89-3·83)	
Shen M	2014	Intratumoral Neutrophils	NHC	OS	3	220	1·69 (1·10-2·60)	
Shen M	2014	Intratumoral Neutrophils	Multiple	RFS/DFS	10	2139	1·68 (1·36-2·07)	Assessed two outcomes within a single analysis
Shen M	2014	Peritumoral Neutrophils	Multiple	RFS/DFS	3	636	1·80 (0·96-3·37)	Assessed two outcomes within a single analysis

Shen M	2014	Stromal Neutrophils	Multiple	RFS/DFS	3	636	1·27 (0·75-2·16)	Assessed two outcomes within a single analysis
Shen M	2014	Intratumoral Neutrophils	HCC and ICC	RFS/DFS	4	846	1·58 (1·33-1·88)	Assessed two outcomes within a single analysis
Su L	2017	NLR	Nasopharyngeal	OS	4	2101	1·77 (1·41-2·23)	
Su L	2017	NLR	Nasopharyngeal	PFS	4	1934	1·67 (1·36-2·06)	
Su L	2017	NLR	Nasopharyngeal	CSS	3	2320	1·42 (1·21-1·67)	
Su L	2017	NLR	Nasopharyngeal	LRFS	2	425	1·64 (1·15-2·34)	Does not assess outcomes of interest
Sun J	2016	NLR	Gastric	OS	19	5431 (2186/3245)	1·98 (1·75-2·24)	
Sun J	2016	NLR	Gastric	DFS	7	976 (472/504)	1·58 (1·32-1·88)	
Sun XD	2016	NLR	Hepatocellular with liver transplantation	OS	7	1243 (264/979)	2·71 (1·91-3·83)	
Sun XD	2016	NLR	Hepatocellular with liver transplantation	DFS	10	1637 (372/1265)	3·61 (2·23-5·84)	
Szor D	2018	NLR	Gastric (Resected)	OS	7	3223 (1018/2205)	2·89 (2·41-3·47)	
Takenaka Y	2017	NLR	Nasopharyngeal	OS	6	2890	1·51 (1·27-1·78)	
Takenaka Y	2017	NLR	Nasopharyngeal	DSS	3	NA	1·44 (1·22-1·71)	Does not assess outcomes of interest
Takenaka Y	2017	NLR	Nasophayrngeal	PFS	3	1872	1·53 (1·22-1·90)	
Takenaka Y	2017	NLR	Nasopharyngeal	DMFS	NA	NA	NA	Does not assess outcomes of interest
Takenaka Y	2018	NLR	Head and Neck	OS	17	3371	1·69 (1·47-1·93)	
Takenaka Y	2018	NLR	Head and Neck	DSS	5	972	1·88 (1·20-2·95)	Does not assess outcomes of interest
Tan DW	2016	NLR	Cholangiocarcinoma	OS	12	2093	1·45 (1·30-1·62)	
Tan Q	2018	NLR	Immune checkpoint inhibitors	OS	18	1863	2·26 (1·68-3·03)	
Tan Q	2018	NLR	Immune checkpoint inhibitors	PFS	11	1274	1·81 (1·36-2·41)	
Tang H	2016	NLR	Colorectal Liver Metastasis	OS	7	1491	2·17 (1·82-2·58)	
Tang H	2016	NLR	Colorectal Liver Metastasis	RFS	6	1093	1·96 (1·64-2·35)	
Tang H	2016	NLR	Colorectal Liver Metastasis Surgical Resection	OS	5	1309	2·08 (1·73-2·49)	
Tang H	2016	NLR	Colorectal Liver Metastasis Surgical Resection	RFS	4	903	1·89 (1·34-2·66)	
Tang H	2016	NLR	Colorectal Liver Metastasis Non-surgical	OS	2	182	3·11 (1·83-5·49)	
Tang H	2016	NLR	Colorectal Liver Metastasis Non-surgical	RFS	2	190	2·33 (1·27-3·95)	

Tang H	2017	NLR	Biliary Tract	OS	14	3217 (1295/1710)*	1·51 (1·36-1·67)	
Tang H	2017	NLR	Biliary Tract	RFS	5	637 (303/244)	1·46 (1·18-1·81)	
Tang L	2016	NLR	Prostate	OS	13	NA	1·63 (1·41-1·88)	Does not include complete data for analysis
Tang L	2016	NLR	Prostate	RFS	10	NA	1·36 (1·13-1·64)	Does not include complete data for analysis
Tang L	2016	NLR	Prostate Localised	OS	2	NA	1·44 (0·75-2·75)	Does not include complete data for analysis
Tang L	2016	NLR	Prostate Localised	PFS	7	NA	1·33 (1·05-1·69)	Does not include complete data for analysis
Tang L	2016	NLR	Prostate Locally Advanced	OS	10	NA	1·51 (1·40-1·63)	Does not include complete data for analysis
Tang L	2016	NLR	Prostate Locally Advanced	PFS	3	NA	1·39 (1·17-1·67)	Does not include complete data for analysis
Tang X	2017	NLR	Bladder	OS	11	3093	1·19 (1·07-1·31)	
Tang X	2017	NLR	Bladder	CSS	8	2766	1·40 (1·17-1·69)	
Tang X	2017	NLR	Bladder	RFS	8	4850	1·58 (1·24-2·03)	
Tang X	2017	NLR	Bladder	PFS	6	3327	1·33 (1·19-1·49)	
Te	2014	NLR	Pancreatic	OS	4	466	2·27 (1·01-5·14)	
Templeton AJ	2014	NLR	Multiple	OS	79	33432	1·81 (1·67-1·97)	Does not include complete data for analysis
Templeton AJ	2014	NLR	Multiple	CSS	10	14407	1·61 (1·36-1·91)	Does not include complete data for analysis
Templeton AJ	2014	NLR	Multiple	PFS	16	7822	1·63 (1·39-1·91)	Does not include complete data for analysis
Templeton AJ	2014	NLR	Multiple	DFS	28	7203	2·27 (1·85-1·79)	Does not include complete data for analysis
Templeton AJ	2014	NLR	Hepatocellular	OS	11	8849	1·43 (1·23-1·66)	Does not include complete data for analysis
Templeton AJ	2014	NLR	Hepatocellular	DFS	NA		4·49 (1·87-10·8)	Does not include complete data for analysis
Templeton AJ	2014	NLR	Colorectal	OS	22	8849	1·91 (1·53-2·39)	Does not include complete data for analysis
Templeton AJ	2014	NLR	Colorectal	CSS	NA		1·37 (0·89-2·12)	Does not include complete data for analysis
Templeton AJ	2014	NLR	Colorectal	PFS	NA		1·32 (1·16-1·91)	Does not include complete data for analysis
Templeton AJ	2014	NLR	Colorectal	DFS	NA		2·00 (1·53-2·62)	Does not include complete data for analysis
Templeton AJ	2014	NLR	Gastroesophageal	OS	14	4548	1·66 (1·46-1·88)	Does not include complete data for analysis
Templeton AJ	2014	NLR	Gastroesophageal	CSS	NA		1·27 (0·83-1·96)	Does not include complete data for analysis
Templeton AJ	2014	NLR	Gastroesophageal	DFS	NA		2·01 (1·51-2·69)	Does not include complete data for analysis
Templeton AJ	2014	NLR	Cholangiocarinoma	OS	3	973	1·43 (1·25-1·63)	Does not include complete data for analysis
Templeton AJ	2014	NLR	NSCLC	OS	7	1591	1·66 (1·40-1·96)	Does not include complete data for analysis
Templeton AJ	2014	NLR	NSCLC	PFS	NA		1·63 (1·33-1·99)	Does not include complete data for analysis
Templeton AJ	2014	NLR	Renal	OS	8	1704	2·22 (1·72-2·88)	Does not include complete data for analysis
Templeton AJ	2014	NLR	Renal	PFS	NA		1·64 (0·99-2·74)	Does not include complete data for analysis
Templeton AJ	2014	NLR	Renal	DFS	NA		2·61 (1·62-4·18)	Does not include complete data for analysis

Templeton AJ	2014	NLR	Mesothelioma	OS	7	693	2·35 (1·89-2·92)	Does not include complete data for analysis
Templeton AJ	2014	NLR	Other	OS	36	10715	1·57 (1·39-1·82)	Does not include complete data for analysis
Templeton AJ	2014	NLR	Other	OS	28	7675	1·79 (1·63-1·97)	Does not include complete data for analysis
Templeton AJ	2014	NLR	Other	OS	36	22169	1·80 (1·63-1·99)	Does not include complete data for analysis
Templeton AJ	2014	NLR	Other	CSS	NA		1·78 (1·47-2·15)	Does not include complete data for analysis
Templeton AJ	2014	NLR	Other	PFS	NA		2·08 (1·61-2·70)	Does not include complete data for analysis
Templeton AJ	2014	NLR	Other	DFS	NA		1·46 (1·15-1·87)	Does not include complete data for analysis
Tham T	2018	NLR	Head and Neck	OS	11	1972 (1137/835)	1·51 (1·32-1·73)	
Tham T	2018	NLR	Head and Neck	DSS	5	3184	1·50 (1·23-1·83)	Does not assess outcomes of interest
Tsai PL	2016	NLR	Colorectal	OS	4	1844	2·03 (1·56-2·63)	
Tsai PL	2016	NLR	Colorectal	DFS	2	1480	1·67 (1·19-2·35)	
Vartolomei M	2018	NLR	Urothelial Carcinoma	OS	3	822	1·64 (1·23-2·17)	
Vartolomei M	2018	NLR	Urothelial Carcinoma	RFS	6	3563	1·60 (1·16-2·20)	
Vartolomei M	2018	NLR	Urothelial Carcinoma	CSS	7	4132	1·73 (1·23-2·44)	
Vartolomei MD	2018	NLR	Non muscle-Invasive Bladder Cancer	RFS	6	2298	1·78 (1·32-2·40)	
Vartolomei MD	2018	NLR	Non muscle-Invasive Bladder Cancer	PFS	6	2298	2·14 (1·59-2·87)	
Vartolomei MD	2018	NLR	Non muscle-Invasive Bladder Cancer (High Risk)	RFS	4	599	2·31 (1·27-4·22)	
Vartolomei MD	2018	NLR	Non muscle-Invasive Bladder Cancer (High Risk)	PFS	3	510	2·54 (1·37-4·71)	
Wang Y	2018	NLR	Hepatocellular Resection	OS	13	4225 (1336/2723)*	1·52 (1·37-1·69)	
Wang Y	2018	NLR	Hepatocellular Resection	RFS	10	3404 (940/2298)*	1·64 (1·44-1·87)	
Wang Y	2018	NLR	Hepatocellular Resection	DFS	7	1272 (581/691)	1·50 (1·35-1·67)	
Wang Y	2018	NLR	Oral Squamous Cell Carcinoma	OS	6	1360	1·56 (1·28-1·90)	
Wang Y	2018	NLR	Oral Squamous Cell Carcinoma	DFS	6	1161	1·93 (1·47-2·54)	
Wei B	2016	NLR	Breast (some with Metastasis)	OS	10	6812	2·03 (1·41-2·93)	
Wei B	2016	NLR	Breast (some with Metastasis)	DFS	8	5314	1·46 (1·12-1·90)	
Wei Y	2014	NLR	Urinary	OS	13	3391	1·81 (1·48-2·21)	
Wei Y	2014	NLR	Urinary	RFS/CSS	9	2060	2·07 (1·65-2·60)	Assessed two outcomes within a single analysis

Wei Y	2014	NLR	Urothelial Carcinoma	RFS/CSS	3	456	2·58 (1·66-4·01)	Assessed two outcomes within a single analysis
Wei Y	2014	NLR	Renal	OS	9	1764	1·90 (1·47-2·45)	
Wei Y	2014	NLR	Renal	RFS/CSS	4	1347	1·83 (1·35-2·48)	Assessed two outcomes within a single analysis
Wei Y	2014	NLR	Bladder	RFS/CSS	2	257	2·20 (1·27-3·80)	Assessed two outcomes within a single analysis
Wei Y	2014	NLR	Prostate	OS	2	377	1·33 (0·99-1·80)	
Wei Y	2014	NLR	Urinary	RFS/CSS	9	2060	2·07 (1·65-2·60)	Assessed two outcomes within a single analysis
Wu S	2018	NLR	Bladder	OS	9	4151	1·27 (1·12-1·43)	
Wu S	2018	NLR	Bladder	RFS	9	8075	1·41 (1·23-1·60)	
Wu S	2018	NLR	Bladder	PFS	7	6771	1·75 (1·36-2·15)	
Wu S	2018	NLR	Bladder (Metastasis)	OS	4	862	1·63 (1·34-1·91)	
Wu S	2018	NLR	Bladder	CSS/DSS	NA	NA	1·27 (1·19-1·35)	Does not include complete data for analysis
Wu J	2017	NLR	Cervical	OS	12	3661	1·38 (1·20-1·58)	
Wu J	2017	NLR	Cervical	PFS	10	2452	1·65 (1·31-2·07)	
Xiao WK	2014	NLR	Hepatocellular Transplant	OS	5	788 (123/665)	3·42 (2·41-4·85)	
Xiao WK	2014	NLR	Hepatocellular Mixed treatment	OS	3	522 (213/309)	1·85 (1·40-2·44)	
Xiao WK	2014		Hepatocellular TACE	OS	3	360 (102/258)	2·52 (1·64-3·86)	
Xiao WK	2014	NLR	Hepatocellular Resection	OS	2	1054 (264/790)	3·33 (2·23-4·98)	
Xiao WK	2014	NLR	Hepatocellular RFA	OS	2	370 (172/198)	1·28 (1·10-1·48)	
Xiao WK	2014	NLR	Hepatocellular Transplant	DFS	5	788 (123/665)	5·9 (3·99-8·70)	
Xiao WK	2014	NLR	Hepatocellular RFA	DFS	2	1054 (264/790)	2·10 (2·06-2·14)	
Xiao WK	2014	NLR	Hepatocellular Resection	DFS	2	370 (172/198)	1·07 (0·82-1·40)	
Xin-Ji Z	2015	NLR	Gastric	OS	17	5623	1·65 (1·46-1·83)	
Xin-Ji Z	2015	NLR	Gastric	DFS	4	1736	1·61 (1·28-1·94)	
Xu W	2018	NLR	Hepatocellular (TACE)	OS	6	NA	1·53 (1·29-1·78)	
Xu W	2018	NLR	Hepatocellular (TACE)	PFS	NA	NA	NA	Does not include complete data for analysis
Xu ZG	2018	NLR	Hepatocellular (Transplant)	OS	8	1230 (352/878)	2·22 (1·34-3·68)	
Xu ZG	2018	NLR	Hepatocellular (Transplant)	RFS	4	83 (251/372)*	3·77 (2·01-7·06)	
Xu ZG	2018	NLR	Hepatocellular (Transplant)	DFS	8	1104 (176/715)*	2·51 (1·22-5·15)	

Xue TC	2014	NLR	Hepatocellular	OS	21	3874 (1295/2504)*	2·10 (1·74-2·54)	
Xue TC	2014	NLR	Hepatocellular	DFS	17	3184 (1025/2159)	2·47 (1·86-3·30)	
Yang HB	2016	NLR	Lung (both)	OS	18	6206*	1·23 (1·17-1·29)	
Yang HB	2016	NLR	Lung (both)	PFS	7	1186*	1·18 (1·08-1·29)	
Yang JJ	2015	NLR	Pancreatic	OS	8	959 (268/691)	2·61 (1·68-4·06)	
Yang JJ	2015	NLR	Pancreatic	CSS	3	845 (387/458)	1·66 (1·08-2·57)	
Yang X	2015	NLR	Oesophageal	OS	6	1633	1·54 (1·32-1·80)	
Yang X	2015	NLR	Oesophageal	DFS	2	385	1·74 (1·25-2·43)	
Yang Z	2017	NLR	Ovarian	OS	10	2320	1·72 (1·18-2·51)	
Yang Z	2017	NLR	Ovarian	PFS	7	1863	1·80 (1·22-2·65)	
Yin X	2016	NLR	Prostate Localised	OS	2	8765	1·45 (0·77-2·71)	
Yin X	2016	NLR	Prostate Castration Resistant	OS	9	2765	1·57 (1·41-1·74)	
Yin X	2016	NLR	Prostate Localised	RFS	4	9553	1·34 (0·89-2·02)	
Yin X	2016	NLR	Prostate Castration Resistant	PFS	4	1010	1·97 (1·28-3·04)	
Yin Y	2015	NLR	Lung (both)	OS	17	2990*	1·51 (1·32-1·72)	
Yodying H	2016	NLR	Oesophageal	OS	7	1540	1·40 (1·08-1·81)	
Yodying H	2016	NLR	Oesophageal	DFS	3	476	1·54 (0·79-2·98)	
Yu B	2017	NLR	Nasopharyngeal	OS	3	691	1·71 (1·37-2·13)	
Yu B	2017	NLR	Nasopharyngeal	PFS	2	462	1·57 (1·19-2·09)	
Yu B	2017	NLR	Nasopharyngeal	DSS	2	2258	1·41 (1·19-1·66)	Does not assess outcomes of interest
Yu B	2017	NLR	Nasopharyngeal	DMFS	2	883	1·91 (1·35-2·71)	Does not assess outcomes of interest
Yin J	2017	NLR	Nasopharyngeal	OS	5	2464	1·74 (1·45-2·10)	
Yin J	2017	NLR	Nasopharyngeal	PFS	5	4130	1·48 (1·30-1·69)	
Yu Y	2017	NLR	Lung (both)	OS	17	6070	1·46 (1·30-1·64)	
Yu Y	2017	NLR	Lung (both)	PFS	8	1581	1·42 (1·15-1·75)	
Yu Y	2017	NLR	Lung (both) with Surgery	OS	7	2877	1·50 (1·21-1·84)	
Yu Y	2018	NLR	Head and Neck	OS	14	4122	1·84 (1·53-2·23)	
Yu Y	2018	NLR	Head and Neck	PFS	5	1997	2·17 (1·20-3·92)	
Yu Y	2018	NLR	Head and Neck	DFS	4	780	1·99 (1·46-2·71)	
Yu Y	2018	NLR	Head and Neck	RFS	4	658	1·63 (1·09-2·45)	
Yu Y	2018	NLR	Head and Neck	MFS	NA	NA	NA	Does not assess outcomes of interest
Zhan H	2018	NLR	Melanoma	OS	10	4437	1·56 (1·28-1·90)	

Zhan H	2018	NLR	Melanoma	DFS/PFS	6	NA	1·86 (1·24-2·80)	Assessed two outcomes within a single analysis
Zhang J	2017	NLR	Colorectal	OS	13	4628	1·92 (1·57-2·34)	
Zhang J	2017	NLR	Colorectal	DFS	5	2178	1·66 (1·31-2·11)	
Zhang J	2017	NLR	Glioma	OS	6	1021	1·48 (1·25-1·76)	
Zhang X	2014	NLR	Gastric	OS	10	2952 (1768/1184)	1·83 (1·62-2·07)	
Zhang X	2014	NLR	Gastric	PFS	2	314 (158/156)	1·54 (1·22-1·95)	
Zhang X	2014	NLR	Gastric	DFS	3	719 (194/525)	1·58 (1·12-2·21)	
Zhao QT	2015	NLR	Lung (both)	OS	21	6942	1·51 (1·33-1·71)	
Zhao QT	2015	NLR	Lung (both)	PFS	9	1748	1·33 (1·07-1·67)	
Zhao Z	2018	NLR	Ovarian Cancer	OS	12	3261	1·70 (1·35-2·15)	
Zhao Z	2018	NLR	Ovarian Cancer	PFS	11	2713	1·77 (1·48-2·12)	
Zheng	2017	NLR	Hepatocellular	OS	21	4777	1·54 (1·34-1·76)	
Zheng	2017	NLR	Hepatocellular	RFS	12	2792	1·45 (1·16-1·82)	
Zheng DC	2016	NLR	Colorectal	OS	21	7927	1·92 (1·59-2·32)	
Zheng DC	2016	NLR	Colorectal	PFS	14	3662	1·92 (1·51-2·44)	
Zhou Q	2017	NLR	Ovarian Cancer	OS	16	4910	1·50 (1·27-1·77)	
Zhou Q	2017	NLR	Ovarian Cancer	PFS	12	3884	1·53 (1·28-1·84)	
Zhou Y	2018	NLR	Gastroenteropancreatic Neuroendocrine Tumors	OS	4	422	3·05 (1·97-4·76)	
Zhou Y	2018	NLR	Gastroenteropancreatic Neuroendocrine Tumors	*RFS*	3	300	3·30 (2·04-5·32)	
Zhou Y	2018	NLR	Pancreatic Neutroendocrine Tumour	OS	2	223	4·21 (1·95-9·13)	
Zhou Y	2018	NLR	Pancreatic Neutroendocrine Tumour	RFS	2	223	5·37 (2·14-13·47)	
Zhou Y	2018	NLR	Pancreatic	OS	43	NA	1·81 (1·59-2·05)	
Zhou Y	2018	NLR	Pancreatic	DFS	8	8147 (2862/4100)	1·66 (1·17-2·35)	
Zhu Y	2018	NLR	Ovarian	OS	8	1915	2·21 (1·95-2·52)	
Zhu Y	2018	NLR	Ovarian	PFS	5	1069	2·22 (1·92-2·57)	
Li H	2019	NLR	Colorectal Surgical Resection	OS	11	4832	1.66 (1.36–2.02)	
Li H	2019	NLR	Colorectal Surgical Resection	DFS	9	4237	1.54 (1.18–2.02)	
Li H	2019	NLR	Colorectal Surgical Resection	RFS	3	643	2.31 (1.68–3.17)	

Li H	2019	NLR	Colorectal Surgical Resection	DSS	3	x	2.27 (1.75–2.96)	Does not assess outcomes of interest
Liu X	2020	NLR	Adenocarcinoma of the esophagogastric junction (AEG)	OS/CC/DSS	5	x	1.55 (1.10-2.18)	Assessed two outcomes within a single analysis
Duan J	2018	NLR	Breast Surgical Resection	OS	12	6233	2.45 (1.69–3.54)	
Duan J	2018	NLR	Breast Surgical Resection	DFS	15	7820	1.54 (1.28–1.87)	
Duan J	2018	NLR	Breast Surgical Resection	RFS	2	319	4.05 (1.94–8.47)	
Guo W	2019	NLR	Breast (triple negative and Her2 positive)	OS	28	14128	1.78 (1.49-2.13)	
Guo W	2019	NLR	Breast (triple negative and Her2 positive)	DFS	27	11369	1.6 (1.42-1.96)	
Ni L	2020	NLR	Endometrial carcinoma	OS	9	2942	2.22 (1.77-2.78)	
Ni L	2020	NLR	Endometrial carcinoma	PFS	5	1798	1.81 (1.35-2.41)	
Yin X	2019	NLR	Ovarian	OS	10	2400	1.34 (1.16–1.54)	
Yin X	2019	NLR	Ovarian	PFS	10	2400	1.36 (1.17–1.57)	
Liu L	2019	NLR	Hepatocellular carcinoma treated with Sorfenib	OS	11	2324	1.76 (1.44-2.15)	
Li S	2020	NLR	Hepatocellular (TACE)	OS	16	4023	1.81 (1.66-1.97)	
Choi N	2019	NLR	Radiotherapy for Solid Tumours, Head and Neck, Rectal, Lung cancer	OS (all)	38	x	1.9 (1.66-2.17)	Does not include complete data for analysis
Choi N	2019	NLR	Radiotherapy for Solid Tumours, Head and Neck, Rectal, Lung cancer	OS (radiation alone)	9	x	1.71 (1.44-2.04)	Does not include complete data for analysis
Choi N	2019	NLR	Radiotherapy for Solid Tumours, Head and Neck, Rectal, Lung cancer	OS (Head and Neck)	16	x	1.91 (1.49-2.20)	Does not include complete data for analysis
Choi N	2019	NLR	Radiotherapy for Solid Tumours, Head and Neck, Rectal, Lung cancer	OS (Rectal)	6	x	2.45 (1.47-4.08)	Does not include complete data for analysis
Choi N	2019	NLR	Radiotherapy for Solid Tumours, Head and Neck, Rectal, Lung cancer	OS (Lung)	6	x	1.46 (1.24-1.72)	Does not include complete data for analysis

Choi N	2019	NLR	Radiotherapy for Solid Tumours, Head and Neck, Rectal, Lung cancer	DFS	11	x	1.54 (1.23-1.94)	Does not include complete data for analysis
Choi N	2019	NLR	Radiotherapy for Solid Tumours, Head and Neck, Rectal, Lung cancer	PFS	15	x	2.12 (1.64-2.75)	Does not include complete data for analysis
Li LQ	2020	NLR	soft tissue sarcoma	OS	12	1865	2.08 (1.60-2.69)	
Li LQ	2020	NLR	soft tissue sarcoma	DFS	7	1239	1.46 (1.21-1.77)	
Li LQ	2020	NLR	soft tissue sarcoma (synovial sarcoma)	OS	3	x	2.39 (1.89-3.02)	
Li LQ	2020	NLR	soft tissue sarcoma (liposarcoma)	OS	2	x	2.94 (1.81-4.77)	
Li LQ	2020	NLR	soft tissue sarcoma	OS (clear cell sarcoma)	1	x	3.06 (1.26-7.4)	Includes only one component study
Li LQ	2020	NLR	soft tissue sarcoma	OS (leiomayosarcoma)	1	x	x	Includes only one component study
Li LQ	2020	NLR	soft tissue sarcoma	OS (angiosarcoma)	1	x	2.15 (1.29-3.59)	Includes only one component study
Li LQ	2020	NLR	soft tissue sarcoma	OS (undifferentiated pleomorphic sarcoma)	1	x	2.17 (1.29-3.59)	Includes only one component study
Li LQ	2020	NLR	soft tissue sarcoma	OS (rhabdomyosarcoma)	1	22	4.76 (1.02-22.24)	Includes only one component study
Li LQ	2020	NLR	soft tissue sarcoma	DSS	7	1452	1.72 (1.43-2.08)	Does not assess outcomes of interest
Lei Y	2019	NLR	Glioma	OS	16	2275	1.43 (1.27-1.62)	
Zeng Q	2018	NLR	Multiple Myeloma	PFS	4	1334	1.74 (1.11-2.73)	
Zeng Q	2018	NLR	Multiple Myeloma	OS	8	1886	1.73 (1.23-2.44)	
Wang D	2020	NLR	Glioma	OS	15	2798	1.38 (1.09-1.74)	
Xie X	2019	NLR	Immune checkpoint inhibitors	PFS	11	1114	1.74 (1.34-2.27)	
Xie X	2019	NLR	NSCLC with Immune checkpoint inhibitors	PFS	6	609	1.39 (1.09-1.78)	
Xie X	2019	NLR	Melanoma with Immune checkpoint inhibitors	PFS	4	467	2.07 (1.78-2.41)	
Xie X	2019	NLR	Immune checkpoint inhibitors (nivolumab)	PFS	8	744	1.99 (1.72-2.30)	
Xie X	2019	NLR	Immune checkpoint inhibitors (ipilimumuab)	PFS	3	370	2.12 (1.65-2.71)	

Xie X	2019	NLR	Immune checkpoint inhibitors (nivolumab+ipilimumuab)	OS	1	x	x	Includes only one component study
Xie X	2019	NLR	Immune checkpoint inhibitors (nivolumab)	OS	6	442	2.58 (1.92-3.48)	
Xie X	2019	NLR	Immune checkpoint inhibitors (ipilimumuab)	OS	7	798	2.55 (1.47-4.42)	
Xie X	2019	NLR	NSCLC with Immune checkpoint inhibitors	OS	4	307	2.62 (1.40-4.65)	
Xie X	2019	NLR	Melanoma with Immune checkpoint inhibitors	OS	9	1104	2.51 (1.56-4.02)	
Xie X	2019	NLR	Immune checkpoint inhibitors	OS	14	1449	2.61 (1.77-3.86)	
Xue L	2019	NLR	Neoadjuvant chemotherapy	OS	3	701	2.781 (0.54-14.32)	
Xue L	2019	NLR	Neoadjuvant chemotherapy	DFS	5	1242	1.18 (0.78-1.78)	
Zhan H	2018	NLR	Melanoma	DFS/PFS	6	x	1.86 (1.24-2.80)	Assessed two outcomes within a single analysis
Guo J	2018	NLR	Prostate	OS	13	4917	1.58 (1.41-1.78)	
Guo J	2018	NLR	Prostate metastatic	OS	8	2152	1.66 (1.35-2.03)	
Guo J	2018	NLR	Prostate mixed treatment	OS	3	x	2.16 (1.33-3.50)	Does not include complete data for analysis
Guo J	2018	NLR	Prostate	PFS	7	1088	1.95 (1.53-2.49)	
Hu G	2019	NLR	Bladder with Radical Cystectomy	OS	8	10363	1.14 (1.05-1.24)	
Hu G	2019	NLR	Bladder with Radical Cystectomy and NAC	OS	4	447	1.06 (0.99-1.15)	
Hu G	2019	NLR	Bladder	OS	18	11945	1.23 (1.13-1.33)	
Mori K	2020	NLR	UTUC treated with nephroureterectomy	CSS	12	6350	1.66 (1.34-2.06)	
Mori K	2020	NLR	UCB treated with radical cystectomy	CSS	12	11158	1.2 (1.11-1.29)	
Nunno V	2019	NLR	Renal	OS	21	x	1.8 (1.61–2.00)	Does not include complete data for analysis
Nunno V	2019	NLR	Renal non-metastatic	OS	5	x	1.57 (1.27-1.94)	Does not include complete data for analysis
Nunno V	2019	NLR	Renal metastatic	OS	13	x	2.05 (1.74-2.41)	Does not include complete data for analysis
Nunno V	2019	NLR	Renal	PFS	15	x	1.69 (1.42–2.01)	Does not include complete data for analysis
Nunno V	2019	NLR	Renal non-metastatic	DFS	8	x	1.52 (1.23-1.87)	Does not include complete data for analysis
Nunno V	2019	NLR	Renal metastatic	PFS	7	x	1.95 (1.43-2.64)	Does not include complete data for analysis
Su S	2019	NLR	Renal	OS	3	1174	1.05 (0.71-1.53)	

Su S	2019	NLR	Renal	DFS	1	x	2.02 (1.03-3.94)	Includes only one component study
Su S	2019	NLR	Renal	CSS	1	x	2.67 (1.06-6.71)	Includes only one component study
Su S	2019	NLR	Urothelial Carcinoma	OS	7	2554	1.29 (1.03-1.61)	
Su S	2019	NLR	Urothelial Carcinoma	DFS	2	400	1.44 (0.89-2.34)	
Su S	2019	NLR	Urothelial Carcinoma	CSS	5	1067	1.51 (0.89-2.34)	
Su S	2019	NLR	Prostate	OS	2	2230	1.35 (1.20-1.51)	
Shen J	2019	NLR	Renal metastatic	OS	12	x	2.17 (1.68–2.79)	Does not include complete data for analysis
Shen J	2019	NLR	Renal metastatic	PFS	x	x	1.78 (0.91–3.49)	Does not include complete data for analysis
Suh J	2019	NLR	Urothelial transurethral resection of bladder tumor	RFS	5	1129	2.32 (1.77-3.05)	
Suh J	2019	NLR	Urothelial transurethral resection of bladder tumor	PFS	4	1040	2.45 (1.49-4.02)	
Suh J	2019	NLR	Urothelial Carcinoma with radical cystectomy	OS	10	7049	1.18 (1.08-1.30)	
Suh J	2019	NLR	Urothelial Carcinoma with radical cystectomy	PFS	5	5935	1.12 (1.03-1.22)	
Suh J	2019	NLR	Urothelial Carcinoma with radical cystectomy	CSS	9	6974	1.35 (1.18-1.55)	
Suh J	2019	NLR	Urothelial Carcinoma with Chemo	OS	5	1664	1.44 (1.28-1.62)	
Suh J	2019	NLR	Urothelial Carcinoma with Chemo	PFS	2	500	1.3 (1.02-1.64)	
Suh J	2019	NLR	Urothelial Carcinoma with nephroureterectomy	PFS	8	4411	1.63 (1.22-2.18)	
Suh J	2019	NLR	Urothelial Carcinoma with nephroureterectomy	CSS	8	4893	1.68 (1.23-2.31)	
Suh J	2019	NLR	Urothelial Carcinoma with nephroureterectomy	OS	3	1103	1.72 (1.31-2.25)	
Guan Y	2020	NLR	Prostate (castration resistant) with abitaterone	OS	10	1626	1.63 (1.43-1.85)	
Guan Y	2020	NLR	Prostate (castration resistant) with enzalutamide	OS	5	1518	1.48 (1.27-1.72)	
Guan Y	2020	NLR	Prostate (castration resistant) with abitaterone	PFS	2	198	1.62 (0.91-3.26)	

Guan Y	2020	NLR	Prostate (castration resistant) with enzalutamide	PFS	2	427	1.55 (.96-2.45)	
Yang S	2019	NLR	Nasopharyngeal	OS	10	7031	1.46 (1.30-1.63)	
Yang S	2019	NLR	Nasopharyngeal	PFS	4	2012	1.67 (1.36-2.07)	
Ishibashi Y	2020	NLR	Oesophageal with SR	OS	9	4128	1.47 (1.32-1.63)	
Lu Y	2020	NLR	NSCLC	OS	21	5141	1.4 (1.26–1.55)	
Lu Y	2020	NLR	NSCLC	PFS	3	701	1.55 (1.27–1.88)	
Sun Y	2018	NLR	Oesophageal squamous cell carcinom	OC/CSS	x	x	x	Does not assess outcomes of interest
Sun Y	2018	NLR	Oesophageal squamous cell carcinom	EFS	x	x	x	Does not assess outcomes of interest
Yang Y	2018	NLR	Oral cavity	OS	9	2373	1.61 (1.39-1.86)	
Yang Y	2018	NLR	Oral cavity	DFS	10	1920	1.73 (1.44-2.07)	
Yang L	2019	NLR	head and neck squamous cell carcinoma	OS	23	6834	1.68 (1.39-2.03)	
Yang L	2019	NLR	squamous cell carcinoma of oral cavity	OS	5	3234	1.59 (1.36-1.85)	
Yang L	2019	NLR	oropharynx	OS	3	x	2.7 (1.74-4.18)	Does not include complete data for analysis
Yang L	2019	NLR	hypopharynx	OS	3	310	2.88 (2.06-4.03)	
Yang L	2019	NLR	larynx	OS	4	826	1.65 (1.24-2.19)	
Yang L	2019	NLR	head and neck squamous cell carcinoma	DFS	9	2406	1.76 (1.42-2.17)	
Yang L	2019	NLR	oropharynx	DFS	3	x	1.69 (1.24-2.30)	
Yang L	2019	NLR	larynx	DFS	3	1260	1.36 (1.13-1.63)	
Yang L	2019	NLR	head and neck squamous cell carcinoma	PFS	4	461	1.53 (1.09-2.14)	
Yang L	2019	NLR	head and neck squamous cell carcinoma	CSS	5	1854	1.45 (1.23-1.71)	
Zhang X	2018	NLR	Oesophageal	OS	26	8985	1.39 (1.23–1.54)	
Zhang X	2018	NLR	Oesophageal Squamous cell carcinoma	OS	18	7403	1.343 (1.145-1.541)	
Zhang X	2018	NLR	Oesophageal adenocarcinoma	OS	1	327	x	Includes only one component study
Zhang X	2018	NLR	Oesophageal mixed treatment	OS	7	x	1.391 (1.120-1.662)	Does not include complete data for analysis
Zhang X	2018	NLR	Oesophageal Surgery	OS	22	7119	1.349 (1.188-1.509)	
Zhang X	2018	NLR	Oesophageal no Surgery	OS	4	1046	1.666 (1.151-2.181)	

Zhang X	2018	NLR	Oesophageal	DFS	10	2837	1.41 (1.12-1.69)	
Zhang X	2018	NLR	Oesophageal	PFS	4	908	1.4 (1.15-1.65)	
Zhang X	2018	NLR	Oesophageal	RFS	3	677	1.51 (1.11-1.91)	
Zhang X	2018	NLR	Oesophageal	CSS	7	1885	1.38 (1.07-1.69)	
Wang Z	2019	NLR	NSCLC with systematic therapy	OS	24	3694	1.63 (1.43-1.84)	
Wang Z	2019	NLR	NSCLC chemo	OS	9	1276	1.73 (1.26-2.36)	
Wang Z	2019	NLR	NSCLC immuno	OS	8	1396	2.5 (1.60-3.89)	
Wang Z	2019	NLR	NSCLC targeted	OS	5	422	1.92 (1.14-3.24)	
Wang Z	2019	NLR	NSCLC targeted	PFS	5	619	1.53 (1.04-2.25)	
Wang Z	2019	NLR	NSCLC immuno	PFS	4	585	1.53 (1.27-1.84)	
Wang Z	2019	NLR	NSCLC chemo	PFS	5	875	1.74 (1.39-2.17)	
Wang Z	2019	NLR	NSCLC with systematic therapy	PFS	16	2679	1.45 (1.28-1.66)	
Tham T	2018	NLR	Head and neck	DFS	11	1692	1.66 (1.30-2.11)	
Tham T	2018	NLR	Head and neck	PFS	6	2173	1.43 (1.09-1.87)	
Tham T	2018	NLR	Head and neck Surgery	OS	3	707	1.76 (1.16-2.68)	
Tham T	2018	NLR	Head and neck no Surgery	OS	9	3285	1.48 (1.21-1.80)	
Tham T	2018	NLR	Oral cavity, oropharyngeal, and hypopharyngeal	OS	7	x	1.83 (1.43-2.35)	Does not include complete data for analysis
Tham T	2018	NLR	Nasopharyngeal	OS	5	2565	1.28 (1.01-1.62)	
Tham T	2018	NLR	Head and neck other	OS	x	x	x	Does not include complete data for analysis
Tham T	2018	NLR	Larynx	OS	4	821	1.6 (1.27-2.03)	
Tham T	2018	NLR	Head and neck	OS	26	6769	1.85 (1.58-2.17)	
Pirozzolo G	2019	NLR	Oesophageal	OS	20	6457	1.68 (1.46-1.93)	
Pirozzolo G	2019	NLR	Oesophageal Surgery+/- chemo	OS	14	3580	1.69 (1.39-2.06)	
Pirozzolo G	2019	NLR	Oesophageal Neo+surgery	OS	3	1314	1.52 (1.00-2.31)	
Pirozzolo G	2019	NLR	Oesophageal DCRT	OS	3	1563	1.74 (1.55-1.95)	
Pirozzolo G	2019	NLR	Oesophageal SCC	OS	15	x	1.61 (1.36-1.90)	Does not include complete data for analysis
Pirozzolo G	2019	NLR	Oesophageal	DFS	8	2077	1.75 (1.35-2.26)	
Pirozzolo G	2019	NLR	Oesophageal	PFS	2	1046	1.66 (1.43-1.93)	
Jiang T	2019	NLR	NSCLC treated with PD-1 inhibitor	OS	13	2018	2.07 (1.70-2.44)	

Jiang T	2019	NLR	NSCLC treated with PD-1 inhibitor	PFS	12	1614	1.59 (1.36-1.82)	
Kamarajah S	2020	NLR	Oesophageal with oesophagectomy	OS	12	x	1.47 (1.30-1.66)	Does not include complete data for analysis
Feng J	2020	NLR	Thyroid	DFS	7	6343	1.79 (0.97-3.33)	
Cho J	2018	NLR	head and neck squamous cell carcinoma	OS	24	5650	1.96 (1.66–2.31)	
Cho J	2018	NLR	head and neck squamous cell carcinoma	DFS	12	2079	1.9 (1.41–2.54)	
Cao D	2018	NLR	NSCLC treated with Nivoumab	OS	8	666	1.75 (1.33–2.30)	
Cao D	2018	NLR	NSCLC treated with Nivoumab	PFS	10	900	1.44 (1.18–1.77)	

N=number of component studies with in review, (NA) Not Applicable, (HR), Hazard ratio, (NLR) Neutrophil to Lymphocyte Ratio, (HNLR) High Neutrophil to Lymphocyte Ratio, (LNLR) Low Neutrophil to Lymphocyte Ratio, (IN) Intratumoural Neutrophils, (PN) Peritumoural Neutrophils, (SN) Stromal Neutrophils, (OS) Overall Survival, (DFS) Disease-Free Survival, (PFS) Progression-Free Survival, (RFS) Reoccurrence-Free Survival, (EFS) Event Free Survival, (CSS) Cancer Specific Survival, (DSS) Disease Specific Survival, (MFS) Metastasis-Free Survival, (DMFS) Distant Metastasis Free Survival, (VEGFR) Vascular Endothelial Growth Factor Receptor, (ICI) Immune Checkpoint Inhibitor, (PC) Palliative Chemotherapy, (SBR) Surgical Bowel Resection, (SR) Surgical Resection, (CLM) Colorectal Liver Metastasis, (NS) Non-surgical, (HCC) Hepatocellular Carcinoma, (ICC) Intrahepatic Cholangiocarcinoma, (RFA) Radiofrequency Ablation, (TACE) Trans-Arterial Chemoembolization, (MPM) Malignant Pleural Mesothelioma, (NSCLC) Non-Small Cell Lung Cancer, (NHC), Neck and Head Cancer, (CR) Castration Resistant, (TKI) Tyrosine Kinase Inhibitor.

*Composite endpoints are defined as a grouping of cancer diagnosis unrelated to site, stage or treatment unless otherwise specified.
†review reported incomplete data on sample size.

Appendix C: Assessment of duplicate meta-analyses

Author	Year	Biomarker	Cancer site	meta-analysis metric	N	Sample size	Pooled HR	Assessing magnitude	Notes	Count included pairs	Count removed
Luo Y	2015	NLR	Bladder	CSS	5	1927	1·70 (1·45-1·99)	1·482352941	AGREE	1	3
Luo Y	2015	NLR	Upper Urinary	CSS	4	1218	2·52 (1·41-4·52)	1			
Li X	2017	NLR	Upper Urinary and Bladder	CSS	17	7645	1·64 (1·39-1·93)	1·536585366			
Tang X	2017	NLR	Bladder	CSS	8	2766	1·40 (1·17-1·69)	1·8			
Lucca I	2015	NLR	Urothelial Carcinoma	CSS	5	5641	1·21 (1·07-1·37)	1·429752066	DO NOT AGREE	0	4
Marchioni M	2017	NLR	Urothelial Carcinoma	CSS	6	1710	1·25 (0.29-5·41)	1·384			
Vartolomei M	2018	NLR	Urothelial Carcinoma	CSS	7	4132	1·73 (1·23-2·44)	1			
Su S	2019	NLR	Urothelial Carcinoma	CSS	5	1067	1.51 (0.89-2.34)	1.145695364			
Chen J	2015	NLR	Breast	DFS	4	2764	1·38 (1·09-1·74)	1·260869565	AGREE	1	3
Ethier JL	2017	NLR	Breast	DFS	9	4864	1·74 (1·47-2·07)	1			
Wei B	2016	NLR	Breast (some with Metastasis)	DFS	8	5314	1·46 (1·12-1·90)	1·191780822			
Liu X	2017	NLR	Breast	DFS	12	5523	1·72 (1·30-2·27)	1·011627907			
Tsai PL	2016	NLR	Colorectal	DFS	2	1480	1·67 (1·19-2·35)	1·006024096	AGREE	1	1
Zhang J	2017	NLR	Colorectal	DFS	5	2178	1·66 (1·31-2·11)	1			
Sun J	2016	NLR	Gastric	DFS	7	976	1·58 (1·32-1·88)	1·018987342	AGREE	1	2
Xin-Ji Z	2015	NLR	Gastric	DFS	4	1736	1·61 (1·28-1·94)	1			
Zhang X	2014	NLR	Gastric	DFS	3	719	1·58 (1·12-2·21)	1·018987342			
Sun XD	2016	NLR	HCC Transplant	DFS	10	1637	3·61 (2·23-5·84)	1·63888889	DO NOT AGREE	0	3
Xiao WK	2014	NLR	Hepatocellular Transplant	DFS	5	788 (123/665)	5·9 (3·99-8·70)				
Xu ZG	2018	NLR	Hepatocellular Transplant	DFS	8	1104 (176/715)*	2·51 (1·22-5·15)	2·35059761			
Huang Y	2017	NLR	Oesophageal	DFS	2	535	1·27 (1·01-1·59)	1.377952756	AGREE	1	4
Yang X	2015	NLR	Oesophageal	DFS	2	385	1·74 (1·25-2·43)	1.005747126			
Yodying H	2016	NLR	Oesophageal	DFS	3	476	1·54 (0.79-2·98)	*Dissagreement in significance disregarded due to small sample size (<5)*			

Zhang X	2018	NLR	Oesophageal	DFS	10	2837	1.41 (1.12-1.69)	1.241134752			
Pirozzolo G	2019	NLR	Oesophageal	DFS	8	2077	1.75 (1.35-2.26)	1			
Chen J	2015	NLR	Breast	OS	5	162172	2·28 (1·08-4·80)	1·122807018			
Ethier JL	2017	NLR	Breast	OS	13	8015	2·56 (1·96-3·35)	1			
Wei B	2016	NLR	Breast (some with Metastasis)	OS	10	6812	2·03 (1·41-2·93)	1·261083744	AGREE	1	3
Liu X	2017	NLR	Breast	OS	11	6836	1·87 (1·41-2·48)	1·368983957			
Huang QT	2017	NLR	Cervical	OS	7	2699	1·88 (1·30-2·73)	1·362318841	AGREE	1	1
Wu J	2017	NLR	Cervical	OS	12	3661	1·38 (1·20-1·58)				
Li MX	2014	NLR	Colorectal	OS	16	629*	1·81 (1·50-1·20)	1·149171271			
Malietzis G	2014	NLR	Colorectal	OS	13	3455*	2·08 (1·64-2·64)	1			
Mei Z	2017	NLR	Colorectal	OS	15	4787	1·89 (1·56-2·30)	1·100529101			
Tsai PL	2016	NLR	Colorectal	OS	4	1844	2·03 (1·56-2·63)	1·024630542			
Zhang J	2017	NLR	Colorectal	OS	13	4628	1·92 (1·57-2·34)	1·083333333	AGREE	1	7
Zheng DC	2016	NLR	Colorectal	OS	21	7927	1·92 (1·59-2·32)	1·083333333			
Paramanathan A	2014	NLR	Colorectal Solid Tumours	OS	6	1961	1·65 (1·21-2·26)	1·260606061			
Dolan R	2017	NLR	Operable Colorectal	OS	8	4204 (825/3379)	1·8 (1·37-2·37)	1·155555556			
Chen J	2015	NLR	Gastric	OS	9	3723	2·16 (1·86-2·51)	1			
Mei Z	2017	NLR	Gastric	OS	4	1715	1·69 (1·41-2·03)	1·278106509			
Sun J	2016	NLR	Gastric	OS	19	5431	1·98 (1·75-2·24)	1·090909091	AGREE	1	4
Xin-Ji Z	2015	NLR	Gastric	OS	17	5623	1·65 (1·46-1·83)	1·309090909			
Zhang X	2014	NLR	Gastric	OS	10	2952	1·83 (1·62-2·07)	1·180327869			
Mei Z	2017	NLR	HCC	OS	2	524	1·91 (1·55-2·37)	1·09947644			
Qi X	2016	NLR	HCC	OS	38	9127	1·80 (1·59-2·04)	1·166666667			
Xue TC	2014	NLR	HCC	OS	21	3874*	2·10 (1·74-2·54)	1	AGREE	1	4
Min GT	2018	NLR	HCC	OS	45	10491 (2594/5312)*	1·52 (1·39-1·67)	1·381578947			
Zheng	2017	NLR	HCC	OS	21	4777	1·54 (1·34-1·76)	1·363636364			
Yang HB	2016	NLR	Lung (both)	OS	18	6206*	1·23 (1·17-1·29)	1·227642276			
Yin Y	2015	NLR	Lung (both)	OS	17	2990*	1·51 (1·32-1·72)	1	AGREE	1	3
Zhao QT	2015	NLR	Lung (both)	OS	21	6942	1·51 (1·33-1·71)	1			
Yu Y	2017	NLR	Lung (both)	OS	17	6070	1·46 (1·30-1·64)	1·034246575			
Su L	2017	NLR	Nasopharyngeal	OS	4	2101	1·77 (1·41-2·23)	1			
Yu B	2017	NLR	Nasopharyngeal	OS	3	691	1·71 (1·37-2·13)	1·035087719			
Mascarella M	2018	NLR	Nasopharyngeal	OS	3	1890	1·66 (1·35-2·04)	1·06626506	AGREE	1	6
Takenaka Y	2017	NLR	Nasopharyngeal	OS	6	2890	1·51 (1·27-1·78)	1·17218543			
Yin J	2017	NLR	Nasopharyngeal	OS	5	2464	1·74 (1·45-2·10)	1·017241379			
Yang S	2019	NLR	Nasopharyngeal	OS	10	7031	1.46 (1.30-1.63)	1.212328767			

Tham T	2018	NLR	Nasopharyngeal	OS	5	2565	1.28 (1.01-1.62)	1.3828125			
Gu XB	2015	NLR	NSCLC	OS	13	3544	1·70 (1·39-2·09)	1	AGREE	1	3
Mei Z	2017	NLR	NSCLC	OS	7	1386	1·20 (1·08-1·34)	1·416666667			
Peng B	2015	NLR	NSCLC	OS	11	2274	1·43 (1·25-1·64)	1·188811189			
Lu Y	2020	NLR	NSCLC	OS	21	5141	1.4 (1.26–1.55)	1.214285714			
Huang Y	2017	NLR	Oesophageal	OS	9	2513	1·31 (1·16-1·48)	1.282442748	AGREE	1	5
Yang X	2015	NLR	Oesophageal	OS	6	1633	1·54 (1·32-1·80)	1.090909091			
Yodying H	2016	NLR	Oesophageal	OS	7	1540	1·40 (1·08-1·81)	1.2			
Paramanathan A	2014	NLR	Oesophageal Solid Tumours	OS	3	559	1·48 (0.91-2·42)	*Dissagreement in significance disregarded due to small sample size (<5)*			
Zhang X	2018	NLR	Oesophageal	OS	26	8985	1.39 (1.23–1.54)	1.208633094			
Pirozzolo G	2019	NLR	Oesophageal	OS	20	6457	1.68 (1.46-1.93)	1			
Huang QT	2017	NLR	Ovarian	OS	12	3935	1·69 (1·29-2·22)	1·307692308	AGREE	1	8
Yang Z	2017	NLR	Ovarian	OS	10	2320	1·72 (1·18-2·51)	1·284883721			
Paramanathan A	2014	NLR	Ovarian Solid Tumours	OS	2	321	2·02 (0.18-22·57)	*Dissagreement in significance disregarded due to small sample size (<5)*			
Chen S	2017	NLR	Ovarian	OS	11	2017*	1·51 (1·03-2·23)	1·463576159			
Chen G	2018	NLR	Ovarian	OS	11	3702	1·41 (1·11-1·79)	1·567375887			
Zhao Z	2018	NLR	Ovarian	OS	12	3261	1·70 (1·35-2·15)	1·3			
Zhou Q	2017	NLR	Ovarian	OS	16	4910	1·50 (1·27-1·77)	1·473333333			
Zhu Y	2018	NLR	Ovarian	OS	8	1915	2·21 (1·95-2·52)	1			
Yin X	2019	NLR	Ovarian	OS	10	2400	1.34 (1.16–1.54)	1.649253731			
Cheng H	2015	NLR	Pancreatic	OS	9	2035	1·59 (1·41-1·79)	1·641509434	AGREE	1	4
Mei Z	2017	NLR	Pancreatic	OS	9	1983	2·04 (1·50-2·75)	1·279411765			
Te	2014	NLR	Pancreatic	OS	4	466	2·27 (1·01-5·14)	1·149779736			
Yang JJ	2015	NLR	Pancreatic	OS	8	959	2·61 (1·68-4·06)	1			
Zhou Y	2018	NLR	Pancreatic	OS	43	NA	1·81 (1·59-2·05)	1·44198895			
Cao J	2016	NLR	Prostate	OS	16	15298	1·41 (1·26-1·55)	1.120567376	AGREE	1	6
Gu X	2016	NLR	Prostate	OS	11	14250	1·38 (1·22-1·56)	1.144927536			
Luo Y	2015	NLR	Prostate	OS	5	2310	1·44 (1·28-1·62)	1.097222222			
Mei Z	2017	NLR	Prostate	OS	5	2019	1·43 (1·26-1·62)	1.104895105			
Wei Y	2014	NLR	Prostate	OS	2	377	1·33 (0.99-1·80)	*Dissagreement in significance disregarded due to small sample size (<5)*			
Guo J	2018	NLR	Prostate	OS	13	4917	1.58 (1.41-1.78)	1			
Su S	2019	NLR	Prostate	OS	2	2230	1.35 (1.20-1.51)	1.17037037			
Hu K	2015	NLR	Renal	OS	13	3034	1·82 (1·51-2·19)	1·06043956	AGREE	1	5

Luo Y	2015	NLR	Renal	OS	14	3373	1·79 (1·61-2·00)	1·078212291			
Mei Z	2017	NLR	Renal	OS	9	1689	1·91 (1·58-2·32)	1·010471204			
Na N	2016	NLR	Renal	OS	8	1220	1·93 (1·35-2·77)	1			
Wei Y	2014	NLR	Renal	OS	9	1764	1·90 (1·47-2·45)	1·015789474			
Su S	2019	NLR	Renal	OS	3	1174	1.05 (0.71-1.53)	*Dissagreement in significance disregarded due to small sample size (<5)*			
Paramanathan A	2014	NLR	All Solid Tumours	OS	36	8626	1·92 (1·64-2·24)	1	AGREE	1	1
Mei Z	2017	NLR	Composite endpoints	OS	65	18262*	1·70 (1·57-1·84)	1·129411765			
Li X	2017	NLR	Upper Urinary and Bladder	OS	17	4432	1·72 (1·45-2·05)	1			
Luo Y	2015	NLR	Bladder	OS	4	1810	1·68 (1·45-1·94)	1·023809520			
Tang X	2017	NLR	Bladder	OS	11	3093	1·19 (1·07-1·31)	1·445378151	AGREE	1	4
Wu S	2018	NLR	Bladder	OS	9	4151	1·27 (1·12-1·43)	1·354330709			
Hu G	2019	NLR	Bladder	OS	18	11945	1.23 (1.13-1.33)	1.398373984			
Lucca I	2015	NLR	Urothelial Carcinoma	OS	4	5452	1·11 (1·01-1·22)	1·774774775			
Marchioni M	2017	NLR	Urothelial Carcinoma	OS	2	397	1·97 (1·27-3·04)	1	AGREE	1	3
Vartolomei M	2018	NLR	Urothelial Carcinoma	OS	3	822	1·64 (1·23-2·17)	1·201219512			
Su S	2019	NLR	Urothelial Carcinoma	OS	7	2554	1.29 (1.03-1.61)	1.527131783			
Huang QT	2017	NLR	Cervical	PFS	5	2607	1·65 (1·18-2·29)	1	AGREE	1	1
Wu J	2017	NLR	Cervical	PFS	10	2452	1·65 (1·31-2·07)	1			
Li MX	2014	NLR	Colorectal	PFS	10	2718*	2·10 (1·55-2·84)	1			
Mei Z	2017	NLR	Colorectal	PFS	5	549	1·91 (1·31-2·79)	1·09947644	AGREE	1	2
Zheng DC	2016	NLR	Colorectal	PFS	14	3662	1·92 (1·51-2·44)	1·09375			
Chen J	2015	NLR	Gastric	PFS	2	282	2·78 (1·95-3·96)	1	AGREE	1	1
Zhang X	2014	NLR	Gastric	PFS	2	314	1·54 (1·22-1·95)	1·805194805			
Yang HB	2016	NLR	Lung (both)	PFS	7	1186*	1·18 (1·08-1·29)	1·203389831			
Zhao QT	2015	NLR	Lung (both)	PFS	9	1748	1·33 (1·07-1·67)	1·067669173	AGREE	1	2
Yu Y	2017	NLR	Lung (both)	PFS	8	1581	1·42 (1·15-1·75)	1			
Su L	2017	NLR	Nasopharyngeal	PFS	4	1934	1·67 (1·36-2·06)	1			
Yu B	2017	NLR	Nasopharyngeal	PFS	2	462	1·57 (1·19-2·09)	1·063694268	AGREE	1	4
Takenaka Y	2017	NLR	Nasopharngeal	PFS	3	1872	1·53 (1·22-1·90)	1·091503268			
Yin J	2017	NLR	Nasopharngeal	PFS	5	4130	1·48 (1·30-1·69)	1·128378378			

Yang S	2019	NLR	Nasopharyngeal	PFS	4	2012	1.67 (1.36-2.07)	1			
Gu XB	2015	NLR	NSCLC	PFS	10	2623	1·63 (1·27-2·09)	1	AGREE	1	3
Mei Z	2017	NLR	NSCLC	PFS	5	1140	1·38 (1·06-1·80)	1·18115942			
Peng B	2015	NLR	NSCLC	PFS	5	1052	1·37 (1·07-1·74)	1·189781022			
Lu Y	2020	NLR	NSCLC	PFS	3	701	1.55 (1.27–1.88)	1.051612903			
Huang QT	2017	NLR	Ovarian	PFS	8	2152	1·63 (1·27-2·09)	1·36196319	AGREE	1	7
Yang Z	2017	NLR	Ovarian	PFS	7	1863	1·80 (1·22-2·65)	1·233333333			
Chen G	2018	NLR	Ovarian	PFS	8	2991	1·52 (1·19-1·95)	1·460526316			
Chen S	2017	NLR	Ovarian	PFS	7	851*	1·55 (1·15-2·08)	1·432258065			
Zhao Z	2018	NLR	Ovarian	PFS	11	2713	1·77 (1·48-2·12)	1·254237288			
Zhou Q	2017	NLR	Ovarian	PFS	12	3884	1·53 (1·28-1·84)	1·450980392			
Zhu Y	2018	NLR	Ovarian	PFS	5	1069	2·22 (1·92-2·57)	1			
Yin X	2019	NLR	Ovarian	PFS	10	2400	1.36 (1.17–1.57)	1.632352941			
Luo Y	2015	NLR	Renal	PFS	7	753	1·85 (1·24-2·77)	1·145945946	AGREE	1	2
Mei Z	2017	NLR	Renal	PFS	5	930	1·49 (1·18-1·89)	1·422818792			
Na N	2016	NLR	Renal	PFS	8	1115*	2·12 (1·42-3·17)	1			
Li X	2017	NLR	Upper Urinary and Bladder	PFS	19	8182	1·68 (1·44-1·96)	0.007936508	AGREE	1	2
Tang X	2017	NLR	Bladder	PFS	6	3327	1·33 (1·19-1·49)	0.01025641			
Wu S	2018	NLR	Bladder	PFS	7	6771	1·75 (1·36-2·15)	1			
Cao J	2016	NLR	Prostate Castration Resistant	PFS	6	1629	1·42 (1·23-1·61)	1·387323944	AGREE	1	1
Yin X	2016	NLR	Prostate Castration Resistant	PFS	4	1010	1·97 (1·28-3·04)	1			
Xiao WK	2014	NLR	HCC Resection	OS	2	1054	3·33 (2·23-4·98)	1·057057057	AGREE	1	2
Paramanathan A	2014	NLR	HCC Resection of Solid Tumours	OS	4	566	3·52 (2·34-5·28)	*Dissagreement in magnitude disregarded due to small sample size (<5)*			
Wang Y	2018	NLR	Hepatocellular Resection	OS	13	4225 (1336/2723)*	1·52 (1·37-1·69)	2·315789474			
Paramanathan A	2014	NLR	Colorectal Liver Metastasis Resection of Solid Tumours	OS	3	814	2·28 (1·77-2·93)	1	AGREE	1	1

Tang H	2016	NLR	Colorectal Liver Metastasis Surgical Resection	OS	5	1309	2·08 (1·73-2·49)	1·096153846			
Cao J	2016	NLR	Prostate Localised	RFS	7	11745	1·38 (1·01-1·75)	1	AGREE	1	1
Yin X	2016	NLR	Prostate Localised	RFS	4	9553	1·34 (0.89-2·02)	*Dissagreement in significance disregarded due to small sample size (<5)*			
Paramanathan A	2014	NLR	Gastric Resection of Solid Tumours	OS	7	2334	1·97 (1·41-2·76)	1·467005076	AGREE	1	2
Mellor K	2018	NLR	potentially curative gastrectomy	OS	5	2787 (1338/1449)	2·31 (1·40-3·83)	1·251082250			
Szor D	2018	NLR	Gastric Resection	OS	7	3223 (1018/2205)	2·89 (2·41-3·47)	1			
Vartolomei M	2018	NLR	Urothelial Carcinoma	RFS	6	3563	1·60 (1·16-2·20)	1	AGREE	1	1
Marchioni M	2017	NLR	Urothelial Carcinoma	RFS	4	1313	1·52 (1·19-1·96)	1·052631579			
Xiao WK	2014	NLR	Hepatocellular Transplant	OS	5	788 (123/665)	3·42 (2·41-4·85)	1	AGREE	1	2
Sun XD	2016	NLR	Hepatocellular Transplant	OS	7	1243 (264/979)	2·71 (1·91-3·83)	1·258302583			
Xu ZG	2018	NLR	Hepatocellular Transplant	OS	8	1230 (352/878)	2·22 (1·34-3·68)	1·540540541			
Ding Y	2018	NLR	Melanoma	OS	10	3207	2·23 (1·64-3·04)	1	AGREE	1	1
Zhan H	2018	NLR	Melanoma	OS	10	4437	1·56 (1·28-1·90)	1·429487179			
Li Y	2016	NLR	Soft Tissue Sarcoma	OS	3	494	3·75 (1·24-11·37)	*Dissagreement in magnitude disregarded due to small sample size (<5)*	AGREE	1	2
Liu G	2018	NLR	Soft Tissue Sarcoma	OS	13	2658*	1·59 (1·28-1·97)	2·358490566			
Li LQ	2020	NLR	Soft Tissue Sarcoma	OS	12	1865	2.08 (1.60-2.69)	1.802884615			
Mascarella M	2018	NLR	Head and Neck	OS	24	6917	1·78 (1·53-2·07)	1.039325843	AGREE	1	4
Takenaka Y	2018	NLR	Head and Neck	OS	17	3371	1·69 (1·47-1·93)	1.094674556			
Tham T	2018	NLR	Head and Neck	OS	11	1972 (1137/835)	1·51 (1·32-1·73)	1.225165563			
Yu Y	2018	NLR	Head and Neck	OS	14	4122	1·84 (1·53-2·23)	1.005434783			
Tham T	2018	NLR	Head and Neck	OS	26	6769	1.85 (1.58-2.17)	1			

Xiao WK	2014	NLR	Hepatocellular Resection	DFS	2	370 (172/198)	1·07 (0.82-1·40)	*Dissagreement in significance disregarded due to small sample size (<5)*	AGREE	1	1
Wang Y	2018	NLR	Hepatocellular Resection	DFS	7	1272 (581/691)	1·50 (1·35-1·67)	1			
Jiang T	2018	NLR	Immune checkpoint blockage	OS	4	1291	2·16 (1·86-2·51)	1.208333333	AGREE	1	4
Tan Q	2018	NLR	Immune checkpoint inhibitors	OS	18	1863	2·26 (1·68-3·03)	1.154867257			
Sacdalan D	2018	NLR	Immune checkpoint inhibitors	OS	3	220	2·52 (1·58-4·02)	1.035714286			
Sacdalan D	2018	NLR	Composite endpoints (immune inhibitor)	OS	7	765	1·92 (1·29-2·87)	1.359375			
Xie X	2019	NLR	Immune checkpoint inhibitors	OS	14	1449	2.61 (1.77-3.86)	1			
Min GT	2018	NLR	Hepatocellular	DFS	17	3881 (919/1729)*	1·71 (1·39-2·11)	1·444444444	AGREE	1	1
Xue TC	2014	NLR	Hepatocellular	DFS	17	3184 (1025/2159)	2·47 (1·86-3·30)	1			
Min GT	2018	NLR	Hepatocellular	RFS	16	5037 (1470/3188)*	1·84 (1·48-2·30)	1	AGREE	1	1
Zheng	2017	NLR	Hepatocellular	RFS	12	2792	1·45 (1·16-1·82)	0.008210181			
Luo Y	2015	NLR	Bladder	RFS	2	531	1·55 (1·21-2·00)	1·019354839	AGREE	1	2
Tang X	2017	NLR	Bladder	RFS	8	4850	1·58 (1·24-2·03)	1			
Wu S	2018	NLR	Bladder	RFS	9	8075	1·41 (1·23-1·60)	1·120567376			
Tang H	2017	NLR	Biliary Tract	OS	14	3217 (1295/1710)*	1·51 (1·36-1·67)	1	AGREE	1	1
Tan DW	2016	NLR	Cholangiocarcinoma	OS	12	2093	1·45 (1·30-1·62)	1·04137931			
Tan Q	2018	NLR	Immune checkpoint inhibitors	PFS	11	1274	1·81 (1·36-2·41)	1	AGREE	1	3
Sacdalan D	2018	NLR	Immune checkpoint inhibitors	PFS	3	220	1·76 (1·25-2·47)	1·028409091			

Sacdalan D	2018	NLR	Composite endpoints (immune inhibitor)	PFS	5	592	1·66 (1·38-2·01)	1·090361446			
Xie X	2019	NLR	Immune checkpoint inhibitors	PFS	11	1114	1.74 (1.34-2.27)	1.040229885			
Li H	2019	NLR	Colorectal Surgical Resection	DFS	9	4237	1.54 (1.18–2.02)	1.363636364	AGREE	1	1
Malietzis G	2014	NLR	Colorectal with Surgical Bowel Resection	DFS	7	2408 (797/1611)*	2·10 (1·50-2·93)	1			
Zhang J	2017	NLR	Glioma	OS	6	1021	1·48 (1·25-1·76)	1	AGREE	1	2
Lei Y	2019	NLR	Glioma	OS	16	2275	1.43 (1.27-1.62)	1.034965035			
Wang D	2020	NLR	Glioma	OS	15	2798	1.38 (1.09-1.74)	1.072463768			
Yu Y	2018	NLR	Head and Neck	DFS	4	780	1·99 (1·46-2·71)	1	AGREE	1	1
Tham T	2018	NLR	Head and neck	DFS	11	1692	1.66 (1.30-2.11)	1.198795181			
Yu Y	2018	NLR	Head and Neck	PFS	5	1997	2·17 (1·20-3·92)	1	AGREE	1	1
Tham T	2018	NLR	Head and neck	PFS	6	2173	1.43 (1.09-1.87)	1.517482517			
Yang L	2019	NLR	head and neck squamous cell carcinoma	DFS	9	2406	1.76 (1.42-2.17)	1.079545455	AGREE	1	1
Cho J	2018	NLR	head and neck squamous cell carcinoma	DFS	12	2079	1.9 (1.41–2.54)	1			
Yang L	2019	NLR	head and neck squamous cell carcinoma	OS	23	6834	1.68 (1.39-2.03)	1.166666667	AGREE	1	1
Cho J	2018	NLR	head and neck squamous cell carcinoma	OS	24	5650	1.96 (1.66–2.31)	1			
Xu W	2018	NLR	Hepatocellular (TACE)	OS	6	NA	1·53 (1·29-1·78)	1.183006536	AGREE	1	2
Li S	2020	NLR	Hepatocellular (TACE)	OS	16	4023	1.81 (1.66-1.97)	1			
Xiao WK	2014		Hepatocellular TACE	OS	3	360 (102/258)	2·52 (1·64-3·86)	1			

Mascarella M	2018	NLR	Hypopharynx	OS	3	305	2·36 (1·54-3·61)	1.220338983	AGREE	1	1
Yang L	2019	NLR	Hypopharynx	OS	3	310	2.88 (2.06-4.03)	1			
Sacdalan D	2018	NLR	Ipilimumab	OS	2	255	2·05 (1·54-2·74)	1.243902439	AGREE	1	1
Xie X	2019	NLR	Ipilimumuab	OS	7	798	2.55 (1.47-4.42)	1			
Mascarella M	2018	NLR	Larynx	OS	6	1017	1·55 (1·26-1·92)	1.064516129	AGREE	1	2
Yang L	2019	NLR	Larynx	OS	4	826	1.65 (1.24-2.19)	1			
Tham T	2018	NLR	Larynx	OS	4	821	1.6 (1.27-2.03)	1.03125			
Sacdalan D	2018	NLR	Melanoma (immune inhibitor)	OS	3	339	2·18 (1·66-2·84)	1.151376147	AGREE	1	1
Xie X	2019	NLR	Melanoma with Immune checkpoint inhibitors	OS	9	1104	2.51 (1.56-4.02)	1			
Sacdalan D	2018	NLR	Melanoma (immune inhibitor)	PFS	2	281	1·73 (1·33-2·25)	1.196531792	AGREE	1	1
Xie X	2019	NLR	Melanoma with Immune checkpoint inhibitors	PFS	4	467	2.07 (1.78-2.41)	1			
Li X	2018	NLR	Neoadjuvant Chemotherapy	DFS	11	1719	1·32 (1·10-1·59)	1	DO NOT AGREE	0	2
Xue L	2019	NLR	Neoadjuvant chemotherapy	DFS	5	1242	1.18 (0.78-1.78)	1.118644068			
Li X	2018	NLR	Neoadjuvant Chemotherapy	OS	18	3282	1·58 (1·34-1·86	1.760126582	AGREE	1	1
Xue L	2019	NLR	Neoadjuvant chemotherapy	OS	3	701	2.781 (0.54-14.32)	*Dissagreement in significance disregarded due to small sample size (<5)*			
Xie X	2019	NLR	Nivolumab	OS	6	442	2.58 (1.92-3.48)	1	AGREE	1	1
Sacdalan D	2018	NLR	Nivolumab	OS	2	290	1·43 (0·77-2·69)	*Dissagreement in significance disregarded due to small sample size (<5)*			
Sacdalan D	2018	NLR	NSCLC (immune inhibitor)	OS	3	342	1·63 (0·92-2·88)	*Dissagreement in significance disregarded due to small sample size (<5)*	AGREE	1	2

Xie X	2019	NLR	NSCLC with Immune checkpoint inhibitors	OS	4	307	2.62 (1.40-4.65)	1			
Wang Z	2019	NLR	NSCLC immuno	OS	8	1396	2.5 (1.60-3.89)	1.048			
Sacdalan D	2018	NLR	NSCLC (immune inhibitor)	PFS	2	227	1·56 (1·14-2·13)	1	AGREE	1	2
Xie X	2019	NLR	NSCLC with Immune checkpoint inhibitors	PFS	6	609	1.39 (1.09-1.78)	1.122302158			
Wang Z	2019	NLR	NSCLC immuno	PFS	4	585	1.53 (1.27-1.84)	1.019607843			
Zhang X	2018	NLR	Oesophageal	PFS	4	908	1.4 (1.15-1.65)	1.185714286	AGREE	1	2
Pirozzolo G	2019	NLR	Oesophageal	PFS	2	1046	1.66 (1.43-1.93)	1			
Huang Y	2017	NLR	Oesophageal	PFS	2	253	1·22 (0·90-1·66)	*Dissagreement in significance disregarded due to small sample size (<5)*			
Zhang X	2018	NLR	Oesophageal Surgery	OS	22	7119	1.349 (1.188-1.509)	1.089696071	AGREE	1	1
Ishibashi Y	2020	NLR	Oesophageal with SR	OS	9	4128	1.47 (1.32-1.63)	1			
Mascarella M	2018	NLR	Oral cavity	OS	3	1899	1·56 (1·53-2·07)	1.032051282	AGREE	1	1
Yang Y	2018	NLR	Oral cavity	OS	9	2373	1.61 (1.39-1.86)	1			
Luo Y	2015	NLR	Prostate	PFS	2	881	1·29 (1·04-1·59)	1.511627907	AGREE	1	1
Guo J	2018	NLR	Prostate	PFS	7	1088	1.95 (1.53-2.49)	1			
Yang L	2019	NLR	Oral cavity squamous cell carcinoma	OS	5	3234	1.59 (1.36-1.85)	1	AGREE	1	1
Wang Y	2018	NLR	Oral Squamous Cell Carcinoma	OS	6	1360	1·56 (1·28-1·90)	1.019230769			
Suh J	2019	NLR	Urothelial Carcinoma with nephroureterectomy	CSS	8	4893	1.68 (1.23-2.31)	1	AGREE	1	1
Mori K	2020	NLR	UTUC treated with nephroureterectomy	CSS	12	6350	1.66 (1.34-2.06)	1.012048193			

N=number of component studies with in review, (NA) Not Applicable, (HR) Hazard ratio, (NLR) Neutrophil to Lymphocyte Ratio, (IN) Intratumoural Neutrophils, (PN) Peritumoural Neutrophils, (SN) Stromal Neutrophils, (OS) Overall Survival, (DFS) Disease-Free Survival, (PFS) Progression-Free Survival, (RFS) Reoccurrence-Free Survival, (CSS) Cancer Specific Survival, (SBR) Surgical Bowel Resection, (SR) Surgical Resection, (CLM) Colorectal Liver Metastasis, (NS) Non-surgical, (HCC) Hepatocellular Carcinoma, (TACE) Trans-Arterial Chemoembolization, (MPM) Malignant Pleural Mesothelioma, (NSCLC) Non-Small Cell Lung Cancer, (NHC) Neck and Head Cancer.

(*) Composite endpoints are defined as a grouping of cancer diagnosis unrelated to site, stage or treatment unless otherwise specified.
(†) Review reported incomplete data on sample size

Appendix D: Description of 87 meta-analyses with poor reproducibility

Author	Year	Biomarker	Cancer site	meta-analysis metric	N	Sample size (HNLR/LNLR)	Reported Effect size and 95% CI			% difference	Calculated Effect Size and 95% CI			
							HR	Lower Limit	Upper Limit		Random or Fixed	HR	Lower Limit	Upper Limit
Jiang T	2018	NLR	Advanced Cancer (Anti-VEGFR)	OS	14	2918	2.04	1.61	2.6	0.99%	R	2.02	1.61	2.53
Jiang T	2018	NLR	Advanced Cancer (Immunotherapy)	OS	23	4355	1.98	1.66	2.36	1.53%	R	1.95	1.67	2.29
Mei Z	2017	NLR	Composite endpoints	OS	65	18262*	1.7	1.57	1.84	1.18%	R	1.68	1.56	1.82
Paramanathan A	2014	NLR	Composite endpoints	DFS	20	13065	1.99	1.8	2.2	5.85%	R	2.11	1.71	2.6
Dolan R	2017	NLR	Composite endpoints Operable	OS	120	68603 (18667/49936)	1.73	1.56	1.91	4.13%	R	1.66	1.55	1.77
Dolan R	2017	NLR	Composite endpoints Operable	CSS	41	21175 (4617/16558)	1.32	1.24	1.41	12.77%	R	1.5	1.35	1.67
Tang H	2017	NLR	Biliary Tract	RFS	5	637 (303/244)	1.46	1.18	1.81	3.48%	R	1.41	1.19	1.68
Wu S	2018	NLR	Bladder	RFS	9	8075	1.41	1.23	1.6	7.51%	F	1.52	1.29	1.79
Li X	2017	NLR	Bladder and Upper Urinary	CSS	17	7645	1.64	1.39	1.93	1.23%	R	1.62	1.39	1.89
Ethier JL	2017	NLR	Breast	OS	13	8015	2.56	1.96	3.35	0.78%	R	2.54	1.96	3.3
Liu X	2017	NLR	Breast	DFS	12	5523	1.72	1.3	2.27	4.15%	R	1.65	1.3	2.1
Liu X	2017	NLR	Breast	CSS	4	4186	2.09	1.04	4.21	2.91%	R	2.03	1.08	3.82
Chen J	2018	NLR	Breast (No Metastasis)	DFS	2	466	1.34	0.5	3.58	42.53%	R	0.87	0.56	1.35
Wu J	2017	NLR	Cervical	OS	12	3661	1.375	1.2	1.58	6.00%	R	1.46	1.21	1.76
Wu J	2017	NLR	Cervical	PFS	10	2452	1.65	1.31	2.07	3.57%	R	1.71	1.32	2.22
Szor D	2018	NLR	Gastric (Surgical Resection)	OS	7	3223 (1018/2205)	2.89	2.41	3.47	7.97%	F	3.13	1.99	4.92
Zhou Y	2018	NLR	Gastroenteropancreatic Neuroendocrine Tumors	*RFS*	3	300	3.3	2.04	5.32	1.22%	R	3.26	2.01	5.29
Ethier JL	2017	NLR	Gynecologic	OS	27	10530	1.65	1.44	1.89	4.15%	R	1.72	1.46	2.03
Min GT	2018	NLR	Hepatocellular	OS	44	10491 (2594/5312)*	1.52	1.39	1.67	7.59%	R	1.64	1.41	1.9
Min GT	2018	NLR	Hepatocellular	DFS	17	3881 (919/1729)*	1.71	1.39	2.11	6.23%	R	1.82	1.4	2.37
Xu ZG	2018	NLR	Hepatocellular (Transplant)	OS	8	1230 (352/878)	2.22	1.34	3.68	1.36%	R	2.19	1.4	3.43
Xu ZG	2018	NLR	Hepatocellular (Transplant)	RFS	4	83 (251/372)*	3.77	2.01	7.06	0.80%	R	3.74	2.05	6.83

Sun XD	2016	NLR	Hepatocellular (Transplant)	DFS	10	1637	3.61	2.23	5.84	1.65%	R	3.67	2.19	6.13
Tan Q	2018	NLR	Immune checkpoint inhibitors	OS	18	1863	2.26	1.68	3.03	2.24%	R	2.21	1.7	2.88
Tan Q	2018	NLR	Immune checkpoint inhibitors	PFS	11	1274	1.81	1.36	2.41	3.37%	R	1.75	1.39	2.21
Mu S	2018	NLR	Large B	OS	10	NA†	1.83	1.24	2.69	1.63%	R	1.86	1.2	2.88
Mascarella M	2018	NLR	Larynx	OS	6	1017	1.55	1.26	1.92	1.28%	R	1.57	1.25	1.98
Zhao QT	2015	NLR	Lung (both)	OS	21	6942	1.51	1.33	1.71	2.61%	R	1.55	1.33	1.8
Zhao QT	2015	NLR	Lung (both)	PFS	9	1748	1.33	1.07	1.67	3.69%	R	1.38	1.02	1.87
Zhan H	2018	NLR	Melanoma	OS	10	4437	1.56	1.28	1.9	2.53%	R	1.6	1.26	2.02
Li X	2018	NLR	Neoadjuvant Chemotherapy	OS	18	3282	1.58	1.34	1.86	4.94%	R	1.66	1.36	2.04
Li X	2018	NLR	Neoadjuvant Chemotherapy	CSS	5	971	2.22	1.32	3.74	2.28%	R	2.17	1.35	3.47
Vartolomei MD	2018	NLR	Non muscle-invasive Bladder	RFS	6	2298	1.78	1.32	2.4	1.13%	R	1.76	1.33	2.32
Gu XB	2015	NLR	NSCLC	PFS	10	2623	1.63	1.27	2.09	2.48%	R	1.59	1.29	1.95
Zhou Q	2017	NLR	Ovarian	PFS	12	3884	1.53	1.28	1.84	3.22%	R	1.58	1.26	1.98
Mowbray N	2018	NLR	Pancreatic (Surgical Resection)	OS	8	1519	1.77	1.45	2.15	2.87%	R	1.72	1.48	2
Cao J	2016	NLR	Prostate Castration Resistant	PFS	6	1629	1.42	1.23	1.61	18.53%	F	1.71	1.29	2.26
Dong YW	2016	NLR	Rectal	OS	6	857	13.41	4.9	36.72	4.30%	R	14	3.65	53.75
Dong YW	2016	NLR	Rectal	DFS	3	403	4.37	2.33	8.19	2.31%	R	4.27	1.93	9.45
Dong YW	2016	NLR	Rectal	RFS	3	433	3.64	1.88	7.05	1.10%	R	3.6	1.48	8.78
Na N	2016	NLR	Renal	PFS	8	1115*	2.12	1.42	3.17	2.39%	R	2.07	1.47	2.92
Shen M	2014	Intratumoral Neutrophils	Renal	OS	2	206	2.69	1.89	3.83	0.74%	R	2.71	1.9	3.86
Semeniuk-Wojtas A	2018	NLR	Renal (TKI)	PFS	6	NA†	2.01	1.27	3.18	1.50%	R	1.98	1.31	3.01
Boissier R	2017	NLR	Renal Localised	RFS	6	1962 (487/817)*	1.63	1.15	2.29	2.42%	R	1.67	1.13	2.45
Wei Y	2014	NLR	Urinary	OS	13	3391	1.81	1.48	2.21	1.10%	R	1.83	1.48	2.26
Liu G	2018	NLR	Soft Tissue Sarcoma	OS	13	2658*	1.59	1.28	1.97	1.90%	R	1.56	1.3	1.88
Zeng Q	2018	NLR	Multiple Myeloma	OS	8	1886	1.73	1.23	2.44	1.75%	R	1.7	1.28	2.27

Zeng Q	2018	NLR	Multiple Myeloma	PFS	4	1334	1.74	1.11	2.73	2.27%	R	1.78	1.07	2.96
Xie X	2019	NLR	Nivolumab	OS	6	442	2.58	1.92	3.48	1.03%	F	2.55	1.89	3.46
Xie X	2019	NLR	Nivolumab	PFS	8	744	1.54	1.19	1.99	2.63%	R	1.5	1.2	1.88
Xie X	2019	NLR	Ipilimumuab	OS	7	798	2.55	1.47	4.42	0.79%	R	2.53	1.58	4.05
Zhang X	2018	NLR	Oesophageal	OS	26	8985	1.39	1.23	1.54	1.82%	F	1.36	1.30	1.44
Zhang X	2018	NLR	Oesophageal	CSS	7	1885	1.38	1.07	1.69	2.86%	R	1.42	1.12	1.8
Zhang X	2018	NLR	Oesophageal	RFS	3	677	1.51	1.11	1.91	6.41%	R	1.61	1.16	2.25
Pirozzolo G	2019	NLR	Oesophageal (Neo+Surgery)	OS	3	1314	1.52	1	2.31	18.71%	R	1.26	1.09	1.46
Zhang X	2018	NLR	Oesophageal (No Surgery)	OS	4	1046	1.666	1.151	2.181	2.27%	F	1.70	1.45	2.01
Zhang X	2018	NLR	Oesophageal Squamous Cell Carcinoma	OS	18	7403	1.343	1.145	1.541	3.79%	F	1.39	1.30	1.49
Zhang X	2018	NLR	Oesophageal	DFS	10	2837	1.41	1.12	1.69	13.22%	F	1.24	1.14	1.34
Zhang X	2018	NLR	Oesophageal	PFS	4	908	1.4	1.15	1.65	1.78%	F	1.43	1.18	1.73
Duan J	2018	NLR	Breast (Surgical Resection)	DFS	15	7820	1.54	1.28	1.87	2.56%	R	1.58	1.27	1.96
Duan J	2018	NLR	Breast (Surgical Resection)	OS	12	6233	2.45	1.69	3.54	0.81%	R	2.47	1.71	3.56
Liu L	2019	NLR	Hepatocellular (Sorfenib)	OS	11	2324	1.76	1.44	2.15	1.72%	R	1.73	1.45	2.07
Lei Y	***2019***	**NLR**	Glioma	**OS**	**16**	**2275**	1.43	1.27	1.62	6.10%	R	1.52	1.28	1.81
Wang Z	2019	NLR	NSCLC (Targeted Therapy)	OS	5	422	1.92	1.14	3.24	3.17%	R	1.86	1.18	2.91
Wang Z	2019	NLR	NSCLC (Targeted Therapy)	PFS	5	619	1.53	1.04	2.25	1.32%	R	1.51	1.06	2.13
Cao D	2018	NLR	NSCLC (Nivoumab)	OS	8	666	1.75	1.33	2.3	1.73%	R	1.72	1.33	2.23
Cao D	2018	NLR	NSCLC (Nivoumab)	PFS	10	900	1.44	1.18	1.77	4.75%	R	1.51	1.15	2
Jiang T	2019	NLR	NSCLC (PD-1 inhibitor)	OS	13	2018	2.07	1.7	2.44	14.36%	F	2.39	2.03	2.81
Jiang T	2019	NLR	NSCLC (PD-1 inhibitor)	PFS	12	1614	1.59	1.36	1.82	2.48%	F	1.63	1.42	1.87
Wang Z	2019	NLR	NSCLC (Systematic Therapy)	OS	24	3694	1.63	1.43	1.84	13.71%	R	1.87	1.53	2.29
Wang Z	2019	NLR	NSCLC (Systematic Therapy)	PFS	16	2679	1.45	1.28	1.66	3.39%	R	1.5	1.29	1.74
Tham T	2018	NLR	Head and Neck	OS	26	6769	1.85	1.58	2.17	7.28%	R	1.72	1.51	1.96
Yang Y	2018	NLR	Oral cavity	OS	9	2373	1.61	1.39	1.86	6.02%	R	1.71	1.42	2.06
Xie X	2019	NLR	Melanoma (Immune checkpoint inhibitors)	OS	9	1104	2.51	1.56	4.02	0.80%	R	2.49	1.72	3.61
Su S	2019	NLR	Urothelial Carcinoma	OS	7	2554	1.29	1.03	1.61	2.30%	R	1.32	1.03	1.69
Mori K	2020	NLR	UTUC (Nephroureterectomy)	CSS	12	6350	1.66	1.34	2.06	1.21%	R	1.64	1.34	2.02
Mori K	2020	NLR	UTUC (Radical Cystectomy)	CSS	12	11158	1.2	1.11	1.29	7.23%	R	1.29	1.13	1.47
Suh J	2019	NLR	Urothelial Carcinoma (Radical Cystectomy)	CSS	9	6974	1.35	1.18	1.55	12.50%	R	1.53	1.18	1.97

Suh J	2019	NLR	Urothelial Carcinoma (Radical Cystectomy)	OS	10	7049	1.18	1.08	1.3	12.70%	F	1.04	1.02	1.06
Suh J	2019	NLR	Urothelial Carcinoma (Radical Cystectomy)	PFS	5	5935	1.12	1.03	1.22	5.78%	F	1.06	1.03	1.09
Suh J	2019	NLR	Bladder (Urothelial Transurethral Resection)	RFS	5	1129	2.32	1.77	3.05	4.41%	R	2.22	1.81	2.74
Hu G	2019	NLR	Bladder	OS	18	11945	1.23	1.13	1.33	10.77%	R	1.37	1.18	1.6
Feng J	2020	NLR	Thyroid	DFS	7	6343	1.79	0.97	3.33	3.30%	R	1.85	0.93	3.65
Zhou Q	2017	NLR	Ovarian	OS	16	4910	1.5	1.27	1.77	9.52%	R	1.65	1.29	2.11
Cao J	2016	NLR	Prostate	OS	16	15298	1.4	1.25	1.55	3.15%	F	1.36	1.28	1.43
Cao J	2016	NLR	Prostate Localised	RFS	7	11745	1.38	1.01	1.75	9.01%	F	1.26	1.13	1.40
Wang Z	2019	NLR	NSCLC (Immunotherapy)	OS	8	1396	2.5	1.6	3.89	2.99%	R	2.43	1.64	3.59

N=number of component studies with in review, (NA) Not Applicable, (F) Fixed Effects, (R) Random Effects, (HR) Hazard ratio, (NLR) Neutrophil to lymphocyte ratio, (IN) Intratumoural neutrophils, (PN) Peritumoural neutrophils, (SN) Stromal Neutrophils, (OS) Overall Survival, (DFS) Disease-Free Survival, (PFS) Progression-Free Survival, (RFS) Reoccurrence-Free Survival, (CSS) Cancer Specific Survival, (VEGFR) Vascular Endothelial Growth Factor Receptor, (PC) Palliative Chemotherapy, (SBR) Surgical Bowel Resection, (NAC) Neoadjuvant Chemotherapy, (STS) Soft Tissue Sarcoma, (UTR) Urothelial Transurethral Resection, (SR) Surgical Resection, (GNT) Gastroenteropancreatic Neuroendocrine Tumors, (SCC) Squamous Cell Carcinoma, (ST) Systematic Therapy, (MT) Mixed Therapy, (TT) Targeted Therapy, (CLM) Colorectal Liver Metastasis, (NS) Non-surgical, (ICC) Intrahepatic Cholangiocarcinoma, (RFA) Radiofrequency Ablation, (TACE) Trans-Arterial Chemoembolization, (MPM) Malignant Pleural Mesothelioma, (NSCLC) Non-Small Cell Lung Cancer, (NHC), Neck and Head Cancer, (UTUC) Upper Tract Urothelial Carcinoma, (DCRT) Definitive Chemoradiotherapy, (CR) Castration Resistant, (TKI) Tyrosine Kinase Inhibitor.

*All-cancers are defined as a grouping of cancer diagnosis unrelated to site, stage or treatment unless otherwise specified.

†review reported incomplete data on sample size.

Appendix E: Heterogeneity and small study effects in included meta-analyses

							Heterogeneity							Small Study Effects	
**Author**	**Year**	**Biomarker**	**Cancer site**	**meta-analysis metric**	**N**	**Sample size (HNLR/LNLR)**	**Q**	**Q p-value**	**Tau^2**	**SE Tau^2**	**I^2**	**I^2 Upper CI**	**I^2 Lower CI**	**ARC-Thompson p-value**	**Largest > Random**
Jiang T	2018	NLR	Advanced Cancer (Anti-VEGFR)	OS	14	2918	105.08	< 0.001	0.365	0.072	88%	81%	92%	0.016	T
Jiang T	2018	NLR	Advanced Cancer (Immunotherapy)	PFS	16	2318	107.08	< 0.001	0.305	0.051	86%	79%	91%	0.002	F
Mei Z	2017	NLR	Composite endpoints	OS	65	18262*	627.98	< 0.001	0.236	0.016	90%	88%	92%	< 0.001	F
Mei Z	2017	NLR	Composite endpoints	PFS	23	5607	192.17	< 0.001	0.254	0.029	89%	84%	92%	0.001	T
Shen M	2014	IN	Composite endpoints	OS	16	3335	50.67	< 0.001	0.318	0.057	70%	51%	82%	0.033	F
Shen M	2014	IN	Composite endpoints	CSS	2	437	0.05	0.824	< 0.001	0.179	0%	NA	NA	NA	F
Paramanathan A	2014	NLR	Composite endpoints	DFS	20	13065	654.72	< 0.001	0.441	0.072	97%	96%	98%	< 0.001	F
Shen M	2014	PN	Composite endpoints	OS	2	535	7.90	0.005	0.647	0.677	87%	51%	97%	NA	F
Shen M	2014	SN	Composite endpoints	OS	2	535	2.31	0.129	0.206	0.106	57%	0%	90%	NA	F
Dolan R	2017	NLR	Composite endpoints Operable	OS	120	68603 (18667/49936)	7563.80	< 0.001	0.269	0.015	98%	98%	99%	< 0.001	F
Dolan R	2017	NLR	Composite endpoints Operable	CSS	41	21175 (4617/16558)	201.35	< 0.001	0.262	0.025	80%	74%	85%	< 0.001	F
Tang H	2017	NLR	Biliary Tract	OS	14	3217 (1295/1710)*	15.14	0.298	0.095	0.016	14%	0%	53%	0.154	F
Tang H	2017	NLR	Biliary Tract	RFS	5	637 (303/244)	6.85	0.144	0.080	0.027	42%	0%	78%	0.093	F
Hu G	2019	NLR	Bladder	OS	18	11945	76.43	< 0.001	0.258	0.034	78%	65%	86%	< 0.001	F
Wu S	2018	NLR	Bladder	RFS	9	8075	27.70	0.001	0.189	0.029	71%	43%	85%	0.005	F
Wu S	2018	NLR	Bladder (Metastasis)	OS	4	862	0.49	0.922	< 0.001	0.024	0%	0%	6%	0.976	T
Hu G	2019	NLR	Bladder (Radical Cystectomy and NAC)	OS	4	447	3.70	0.296	0.048	0.006	19%	0%	88%	0.475	F
Hu G	2019	NLR	Bladder (Radical Cystectomy)	OS	8	10363	25.63	0.001	0.075	0.006	73%	44%	87%	0.003	F
Suh J	2019	NLR	Bladder (UTR)	PFS	4	1040	1.71	0.635	< 0.001	0.210	0%	0%	73%	0.717	F
Suh J	2019	NLR	Bladder (UTR)	RFS	5	1129	5.03	0.284	0.002	0.046	20%	0%	66%	0.180	F
Li X	2017	NLR	Bladder and Upper Urinary	PFS	19	8182	32.66	0.018	0.197	0.033	45%	6%	68%	< 0.001	F
Li X	2017	NLR	Bladder and Upper Urinary	CSS	17	7645	46.40	< 0.001	0.219	0.033	66%	42%	79%	0.001	F
Ethier JL	2017	NLR	Breast	OS	13	8015	26.42	0.009	0.316	0.086	55%	15%	76%	0.001	F
Liu X	2017	NLR	Breast	DFS	12	5523	46.52	< 0.001	0.281	0.067	76%	59%	86%	< 0.001	F

Liu X	2017	NLR	Breast	CSS	4	4186	13.25	0.004	0.529	0.343	77%	38%	92%	0.071	F
Chen J	2018	NLR	Breast (Metastasis)	OS	2	858	0.01	0.923	< 0.001	0.141	0%	NA	NA	NA	F
Chen J	2018	NLR	Breast (Metastasis)	DFS	3	1003	0.05	0.976	< 0.001	0.090	0%	0%	0%	0.862	T
Chen J	2018	NLR	Breast (No Metastasis)	OS	2	488	13.14	< 0.001	0.947	1.373	92%	74%	98%	NA	F
Chen J	2018	NLR	Breast (No Metastasis)	DFS	2	466	0.60	0.439	< 0.001	0.260	0%	NA	NA	NA	F
Duan J	2018	NLR	Breast (SR)	DFS	15	7820	91.73	< 0.001	0.322	0.064	85%	76%	90%	0.046	T
Duan J	2018	NLR	Breast (SR)	OS	12	6233	25.37	0.008	0.446	0.167	57%	17%	77%	0.219	T
Duan J	2018	NLR	Breast (SR)	RFS	2	319	0.04	0.839	< 0.001	0.509	0%	NA	NA	NA	T
Guo W	2019	NLR	Breast (Triple Negative and Her2 Positive)	DFS	27	11369	52.37	0.002	0.200	0.026	50%	23%	68%	0.015	F
Guo W	2019	NLR	Breast (Triple Negative and Her2 Positive)	OS	28	14128	97.63	< 0.001	0.353	0.057	72%	60%	81%	0.005	F
Liu X	2017	NLR	Breast (Triple-negative)	OS	4	608	2.48	0.479	< 0.001	0.185	0%	0%	81%	0.262	T
Liu X	2017	NLR	Breast (Triple-negative)	DFS	4	461	1.83	0.609	< 0.001	0.310	0%	0%	75%	0.357	F
Wu J	2017	NLR	Cervical	OS	12	3661	26.42	0.006	0.236	0.044	58%	21%	78%	0.016	F
Wu J	2017	NLR	Cervical	PFS	10	2452	93.66	< 0.001	0.364	0.081	90%	84%	94%	0.192	F
Zhang J	2017	NLR	Colorectal	DFS	5	2178	5.63	0.229	0.217	0.092	29%	0%	72%	0.253	F
Zheng DC	2016	NLR	Colorectal	OS	21	7927	58.60	< 0.001	0.322	0.059	66%	46%	78%	0.010	F
Zheng DC	2016	NLR	Colorectal	PFS	14	3662	27.99	0.009	0.341	0.086	54%	15%	75%	0.041	T
Malietzis G	2014	NLR	Colorectal (PC)	DFS	3	515 (148/367)	0.36	0.834	< 0.001	0.036	0%	0%	43%	0.686	F
Li H	2019	NLR	Colorectal (SR)	DFS	9	4237	16.81	0.032	0.276	0.078	52%	0%	78%	0.027	F
Li H	2019	NLR	Colorectal (SR)	OS	11	4832	21.98	0.015	0.238	0.048	54%	10%	77%	0.034	F
Li H	2019	NLR	Colorectal (SR)	RFS	3	643	1.72	0.424	< 0.001	0.081	0%	0%	88%	0.638	F
Tang H	2016	NLR	CLM	OS	7	1491	4.16	0.655	< 0.001	0.028	0%	0%	58%	0.246	T
Tang H	2016	NLR	CLM	RFS	6	1093	9.17	0.102	0.234	0.078	45%	0%	78%	0.455	T
Tang H	2016	NLR	CLM (Non-surgical)	OS	2	182	0.14	0.710	< 0.001	0.233	0%	NA	NA	NA	F
Tang H	2016	NLR	CLM (Non-surgical)	RFS	2	190	1.43	0.232	0.250	0.296	30%	NA	NA	NA	F
Tang H	2016	NLR	CLM (SR)	OS	5	1309	2.02	0.731	< 0.001	0.029	0%	0%	59%	0.713	T
Tang H	2016	NLR	CLM (SR)	RFS	4	903	7.29	0.063	0.259	0.099	59%	0%	86%	0.576	T
Ni L	2020	NLR	Endometrial	OS	9	2942	4.29	0.830	< 0.001	0.053	0%	0%	34%	0.261	F

Ni L	2020	NLR	Endometrial	PFS	5	1798	5.95	0.203	0.187	0.104	33%	0%	74%	0.284	F
Sun J	2016	NLR	Gastric	OS	19	5431	38.17	0.004	0.155	0.020	53%	21%	72%	0.105	F
Sun J	2016	NLR	Gastric	DFS	7	976	3.23	0.780	< 0.001	0.029	0%	0%	46%	0.887	T
Shen M	2014	IN	Gastric	OS	2	388	6.42	0.011	0.584	0.571	84%	36%	96%	NA	T
Zhang X	2014	NLR	Gastric	PFS	2	314	1.19	0.275	0.129	0.146	16%	NA	NA	NA	F
Szor D	2018	NLR	Gastric (SR)	OS	7	3223 (1018/2205)	39.86	< 0.001	0.542	0.215	85%	71%	92%	0.737	T
Mellor K	2018	NLR	Gastric (SR)	DFS	3	820 (156/664)	10.03	0.007	0.657	0.554	80%	37%	94%	0.197	T
Zhou Y	2018	NLR	GNT	OS	4	422	2.01	0.571	< 0.001	0.182	0%	0%	77%	0.299	T
Zhou Y	2018	NLR	GNT	RFS	3	300	1.45	0.484	0.024	0.273	0%	0%	86%	0.466	F
Luo XF	2018	NLR	Gastrointestinal Stromal	OS	2	278	6.02	0.014	0.682	0.789	83%	31%	96%	NA	F
Lei Y	2019	NLR	Glioma	OS	16	2275	88.11	< 0.001	0.283	0.046	83%	74%	89%	< 0.001	F
Ethier JL	2017	NLR	Gynecologic	OS	27	10530	88.64	< 0.001	0.312	0.047	71%	57%	80%	< 0.001	F
Yu Y	2018	NLR	Head and Neck	RFS	4	658	9.57	0.023	0.307	0.131	69%	9%	89%	0.182	T
Tham T	2018	NLR	Head and Neck	OS	26	6769	36.40	0.066	0.172	0.028	31%	0%	58%	< 0.001	F
Tham T	2018	NLR	Head and neck	PFS	6	2173	10.92	0.053	0.221	0.059	54%	0%	82%	0.276	T
Tham T	2018	NLR	Head and neck	DFS	11	1692	15.52	0.114	0.237	0.069	36%	0%	68%	0.032	F
Shen M	2014	IN	Head and Neck	OS	3	220	1.88	0.391	0.025	0.150	0%	0%	89%	0.419	T
Tham T	2018	NLR	Head and Neck (No Surgery)	OS	9	3285	10.40	0.238	0.146	0.045	23%	0%	64%	0.052	T
Yang L	2019	NLR	Head and Neck (SCC)	CSS	5	1854	7.53	0.110	0.001	0.023	47%	0%	81%	0.124	F
Yang L	2019	NLR	Head and Neck (SCC)	PFS	4	461	6.15	0.104	0.228	0.087	51%	0%	84%	0.177	T
Cho J	2018	NLR	Head and Neck (SCC)	OS	24	5650	44.01	0.005	0.259	0.044	48%	16%	68%	0.001	F
Cho J	2018	NLR	Head and Neck (SCC)	DFS	12	2079	35.54	< 0.001	0.387	0.108	69%	44%	83%	0.003	F
Tham T	2018	NLR	Head and Neck (SR)	OS	3	707	4.88	0.087	0.277	0.136	59%	0%	88%	0.272	F
Min GT	2018	NLR	Hepatocellular	OS	44	10491 (2594/5312)*	224.97	< 0.001	0.429	0.053	81%	75%	85%	< 0.001	F
Min GT	2018	NLR	Hepatocellular	DFS	17	3881 (919/1729)*	81.01	< 0.001	0.447	0.102	80%	69%	87%	< 0.001	F
Min GT	2018	NLR	Hepatocellular	RFS	16	5037 (1470/3188)*	93.69	< 0.001	0.374	0.074	84%	75%	90%	0.001	F
Xiao WK	2014	NLR	Hepatocellular (MT)	OS	3	522 (213/309)	1.97	0.374	0.084	0.079	0%	0%	89%	0.394	F
Xiao WK	2014	NLR	Hepatocellular (RFA)	OS	2	370 (172/198)	0.94	0.333	< 0.001	0.055	0%	NA	NA	NA	T
Xiao WK	2014	NLR	Hepatocellular (RFA)	DFS	2	1054 (264/790)	0.94	0.333	< 0.001	0.055	0%	NA	NA	NA	T
Liu L	2019	NLR	Hepatocellular (Sorfenib)	OS	11	2324	30.93	0.001	0.210	0.039	68%	39%	83%	0.001	T

Wang Y	2018	NLR	Hepatocellular (SR)	RFS	10	3404 (940/2298)*	11.61	0.236	0.145	0.032	22%	0%	62%	0.079	F
Wang Y	2018	NLR	Hepatocellular (SR)	OS	13	4225 (1336/2723)*	17.35	0.137	0.134	0.024	31%	0%	64%	0.010	F
Wang Y	2018	NLR	Hepatocellular (SR)	DFS	7	1272 (581/691)	8.51	0.203	0.001	0.011	30%	0%	70%	0.052	F
Li S	2020	NLR	Hepatocellular (TACE)	OS	16	4023	28.47	0.019	0.142	0.019	47%	6%	71%	0.031	F
Xu ZG	2018	NLR	Hepatocellular (Transplant)	OS	8	1230 (352/878)	48.51	< 0.001	0.564	0.222	86%	74%	92%	0.008	F
Xu ZG	2018	NLR	Hepatocellular (Transplant)	RFS	4	83 (251/372)*	9.01	0.029	0.481	0.309	67%	3%	89%	0.262	F
Shen M	2014	IN	Hepatocellular and ICC	OS	4	846	7.02	0.071	0.234	0.077	57%	0%	86%	0.742	T
Yang L	2019	NLR	Hypopharynx	OS	3	310	0.95	0.622	< 0.001	0.087	0%	0%	78%	0.589	T
Jiang T	2018	NLR	Immunotherapy (Advanced Cancer)	OS	23	4355	118.18	< 0.001	0.298	0.043	81%	73%	87%	0.009	T
Tan Q	2018	NLR	Immunotherapy (Immune checkpoint inhibitors)	OS	18	1863	127.64	< 0.001	0.448	0.106	87%	80%	91%	< 0.001	F
Tan Q	2018	NLR	Immunotherapy (Immune checkpoint inhibitors)	PFS	11	1274	67.67	< 0.001	0.303	0.066	85%	75%	91%	< 0.001	F
Xie X	2019	NLR	Ipilimumuab	OS	7	798	136.13	< 0.001	0.593	0.232	96%	93%	97%	0.046	F
Xie X	2019	NLR	Ipilimumuab	PFS	3	370	6.67	0.036	0.328	0.161	70%	0%	91%	0.461	F
Mu S	2018	NLR	Large B	OS	10	NA†	31.24	< 0.001	0.584	0.229	71%	45%	85%	0.131	F
Mu S	2018	NLR	Large B	PFS	9	NA†	25.97	0.001	0.473	0.161	69%	38%	85%	0.085	F
Yang L	2019	NLR	Larynx	DFS	3	1260	1.84	0.399	0.022	0.036	0%	0%	89%	0.415	F
Mascarella M	2018	NLR	Larynx	OS	6	1017	5.45	0.364	0.111	0.046	8%	0%	77%	0.268	F
Zhao QT	2015	NLR	Lung (both)	OS	21	6942	102.04	< 0.001	0.289	0.039	80%	71%	87%	0.001	T
Zhao QT	2015	NLR	Lung (both)	PFS	9	1748	39.37	< 0.001	0.408	0.105	80%	62%	89%	0.205	T
Yu Y	2017	NLR	Lung (Surgery)	OS	7	2877	36.28	< 0.001	0.198	0.034	83%	67%	92%	0.017	T
Zhan H	2018	NLR	Melanoma	OS	10	4437	97.28	< 0.001	0.325	0.065	91%	85%	94%	0.013	F
Ding Y	2018	NLR	Melanoma	PFS	6	752	3.94	0.558	0.090	0.048	0%	0%	68%	0.241	F
Xie X	2019	NLR	Melanoma (Immune checkpoint inhibitors)	OS	9	1104	151.41	< 0.001	0.519	0.162	95%	92%	97%	0.050	F
Xie X	2019	NLR	Melanoma (Immune checkpoint inhibitors)	PFS	4	467	4.65	0.199	0.131	0.036	35%	0%	78%	0.716	F
Chen	2017	NLR	MPM	OS	11	1378	39.67	< 0.001	0.348	0.082	77%	58%	88%	0.719	F

Zeng Q	2018	NLR	Multiple Myeloma	OS	8	1886	36.22	< 0.001	0.301	0.085	81%	63%	90%	0.286	T
Zeng Q	2018	NLR	Multiple Myeloma	PFS	4	1334	24.94	< 0.001	0.474	0.221	88%	72%	95%	0.039	T
Yang S	2019	NLR	Nasopharyngeal	OS	10	7031	9.90	0.358	0.085	0.019	9%	0%	66%	0.171	F
Yin J	2017	NLR	Nasopharyngeal	PFS	5	4130	3.67	0.452	0.052	0.020	0%	0%	77%	0.183	F
Su L	2017	NLR	Nasopharyngeal	CSS	3	2320	1.47	0.480	0.063	0.040	0%	0%	86%	0.487	F
Li X	2018	NLR	NAC	OS	18	3282	74.20	< 0.001	0.332	0.062	77%	64%	85%	< 0.001	T
Li X	2018	NLR	NAC	RFS	7	775	9.43	0.151	0.251	0.102	36%	0%	73%	0.104	T
Li X	2018	NLR	NAC	CSS	5	971	21.41	< 0.001	0.409	0.198	81%	57%	92%	0.126	T
Xie X	2019	NLR	Nivolumab	OS	6	442	7.98	0.158	0.287	0.156	37%	0%	75%	0.345	F
Xie X	2019	NLR	Nivolumab	PFS	8	744	52.44	< 0.001	0.261	0.055	87%	76%	93%	0.068	F
Vartolomei MD	2018	NLR	Non muscle Invasive Bladder	RFS	6	2298	14.03	0.015	0.242	0.073	64%	14%	85%	0.282	F
Vartolomei MD	2018	NLR	Non muscle Invasive Bladder	PFS	6	2298	4.30	0.506	0.172	0.118	0%	0%	71%	0.149	F
Vartolomei MD	2018	NLR	Non muscle Invasive Bladder (High Risk)	RFS	4	599	7.12	0.068	0.451	0.303	58%	0%	86%	0.163	F
Vartolomei MD	2018	NLR	Non muscle Invasive Bladder (High Risk)	PFS	3	510	1.77	0.413	< 0.001	0.305	0%	0%	88%	0.751	T
Lu Y	2020	NLR	NSCLC	OS	21	5141	54.83	< 0.001	0.207	0.022	64%	42%	77%	0.002	F
Gu XB	2015	NLR	NSCLC	PFS	10	2623	48.87	< 0.001	0.251	0.050	82%	67%	90%	< 0.001	F
Shen M	2014	IN	NSCLC	OS	2	967	0.00	0.960	< 0.001	0.021	0%	NA	NA	NA	T
Wang Z	2019	NLR	NSCLC (Chemotherapy)	OS	9	1276	53.18	< 0.001	0.441	0.129	85%	73%	92%	0.049	F
Wang Z	2019	NLR	NSCLC (Chemotherapy)	PFS	5	875	5.44	0.245	0.150	0.049	26%	0%	71%	0.146	F
Xie X	2019	NLR	NSCLC (Immune Checkpoint Inhibitors)	PFS	6	609	17.62	0.003	0.208	0.048	72%	34%	88%	0.018	T
Wang Z	2019	NLR	NSCLC (Immunotherapy)	OS	8	1396	34.59	< 0.001	0.45	0.17	80%	61%	90%	0.001185886	F
Cao D	2018	NLR	NSCLC (Nivoumab)	OS	8	666	55.59	< 0.001	0.288	0.069	87%	77%	93%	0.015	T
Cao D	2018	NLR	NSCLC (Nivoumab)	PFS	10	900	64.86	< 0.001	0.401	0.093	86%	76%	92%	0.008	F
Jiang T	2019	NLR	NSCLC (PD-1 inhibitor)	OS	13	2018	15.38	0.222	0.202	0.056	22%	0%	59%	0.006	F
Jiang T	2019	NLR	NSCLC (PD-1 inhibitor)	PFS	12	1614	3.53	0.982	< 0.001	0.022	0%	0%	0%	0.742	T
Wang Z	2019	NLR	NSCLC (ST)	OS	24	3694	134.01	< 0.001	0.413	0.072	83%	75%	88%	< 0.001	F
Wang Z	2019	NLR	NSCLC (ST)	PFS	16	2679	66.69	< 0.001	0.229	0.032	78%	64%	86%	< 0.001	F
Wang Z	2019	NLR	NSCLC (TT)	OS	5	422	17.59	0.001	0.402	0.185	77%	45%	91%	0.025	F

Wang Z	2019	NLR	NSCLC (TT)	PFS	5	619	16.06	0.003	0.311	0.110	75%	39%	90%	0.034	F
Zhang X	2018	NLR	Oesophageal	OS	26	8985	97.15	< 0.001	0.267	0.031	74%	62%	82%	0.039	T
Zhang X	2018	NLR	Oesophageal	CSS	7	1885	14.46	0.025	0.235	0.058	59%	4%	82%	0.877	T
Zhang X	2018	NLR	Oesophageal	RFS	3	677	3.36	0.187	0.179	0.089	40%	0%	82%	0.675	F
Zhang X	2018	NLR	Oesophageal	DFS	10	2837	65.99	< 0.001	0.394	0.093	86%	77%	92%	0.049	F
Zhang X	2018	NLR	Oesophageal	PFS	4	908	2.45	0.484	0.058	0.034	0%	0%	81%	0.568	T
Pirozzolo G	2019	NLR	Oesophageal (DCRT)	OS	3	1563	1.15	0.562	< 0.001	0.012	0%	0%	82%	0.513	F
Pirozzolo G	2019	NLR	Oesophageal (Neo+Surgery)	OS	3	1314	1.41	0.493	0.001	0.016	0%	0%	85%	0.451	F
Zhang X	2018	NLR	Oesophageal (No Surgery)	OS	4	1046	6.75	0.080	0.220	0.075	56%	0%	85%	0.549	T
Zhang X	2018	NLR	Oesophageal (Surgery)	OS	22	7119	82.49	< 0.001	0.289	0.039	76%	63%	84%	0.076	T
Pirozzolo G	2019	NLR	Oesophageal (Surgery+/-Chemo)	OS	14	3580	42.61	< 0.001	0.330	0.064	69%	47%	82%	0.225	F
Zhang X	2018	NLR	Oesophageal SCC	OS	18	7403	56.55	< 0.001	0.217	0.026	68%	49%	80%	0.331	T
Yang Y	2018	NLR	Oral cavity	OS	9	2373	13.01	0.112	0.172	0.038	38%	0%	72%	0.747	T
Yang Y	2018	NLR	Oral cavity	DFS	10	1920	8.51	0.484	0.002	0.034	0%	0%	60%	0.045	T
Wang Y	2018	NLR	Oral SCC	OS	6	1360	4.38	0.496	0.021	0.040	0%	0%	71%	0.327	F
Wang Y	2018	NLR	Oral SCC	DFS	6	1161	3.46	0.629	0.001	0.083	0%	0%	63%	0.203	F
Yang L	2019	NLR	Oropharynx	DFS	3	NA†	0.39	0.822	< 0.001	0.078	0%	0%	47%	0.670	T
Zhou Q	2017	NLR	Ovarian	PFS	12	3884	75.78	< 0.001	0.330	0.065	85%	76%	91%	0.004	F
Zhou Q	2017	NLR	Ovarian	OS	16	4910	75.30	< 0.001	0.412	0.088	80%	68%	87%	< 0.001	F
Zhou Y	2018	NLR	Pancreatic	OS	43	NA†	232.24	< 0.001	0.345	0.038	82%	76%	86%	< 0.001	F
Zhou Y	2018	NLR	Pancreatic	DFS	8	1236	21.33	0.003	0.417	0.143	67%	31%	84%	0.370	F
Yang JJ	2015	NLR	Pancreatic	CSS	3	845 (387/458)	8.92	0.012	0.321	0.139	78%	27%	93%	0.424	F
Mowbray N	2018	NLR	Pancreatic (SR)	OS	8	1519	10.15	0.180	0.001	0.023	31%	0%	69%	0.054	F
Zhou Y	2018	NLR	Pancreatic Neuroendocrine Tumour	OS	2	223	0.24	0.623	< 0.001	0.459	0%	NA	NA	NA	F
Zhou Y	2018	NLR	Pancreatic Neuroendocrine Tumour	RFS	2	223	0.09	0.766	< 0.001	0.650	0%	NA	NA	NA	F
Guo J	2018	NLR	Prostate	PFS	7	1088	8.22	0.222	0.196	0.084	27%	0%	68%	0.178	T
Cao J	2016	NLR	Prostate	OS	16	15298	50.93	< 0.001	0.174	0.019	69%	48%	81%	0.002	F
Guo J	2018	NLR	Prostate (Metastatic)	OS	8	2152	13.40	0.099	0.176	0.040	40%	0%	73%	0.023	F
Cao J	2016	NLR	Prostate CR	PFS	6	1629	9.52	0.090	0.243	0.072	47%	0%	79%	0.163	F
Yin X	2016	NLR	Prostate CR	OS	9	2765	8.56	0.380	< 0.001	0.009	7%	0%	67%	0.340	F

Yin X	2016	NLR	Prostate Localised	OS	2	8765	4.24	0.039	0.406	0.305	76%	0%	95%	NA	F
Guan Y	2020	NLR	Prostate CR (Abitaterone)	OS	10	1626	16.74	0.053	0.204	0.045	46%	0%	74%	0.069	F
Guan Y	2020	NLR	Prostate CR (Abitaterone)	PFS	2	198	3.54	0.060	0.429	0.362	72%	0%	94%	NA	T
Guan Y	2020	NLR	Prostate CR (Enzalutamide)	OS	5	1518	10.23	0.037	0.148	0.025	61%	0%	85%	0.110	F
Guan Y	2020	NLR	Prostate CR (Enzalutamide)	PFS	2	427	9.97	0.002	0.314	0.155	90%	63%	97%	NA	T
Cao J	2016	NLR	Prostate Localised	RFS	7	11745	62.96	< 0.001	0.49	0.18	90%	83%	95%	0.351065463	F
Dong YW	2016	NLR	Rectal	OS	6	857	1.78	0.879	< 0.001	1.822	0%	0%	29%	0.298	T
Dong YW	2016	NLR	Rectal	DFS	3	403	0.91	0.635	< 0.001	1.135	0%	0%	77%	0.538	F
Dong YW	2016	NLR	Rectal	RFS	3	433	0.76	0.684	< 0.001	0.710	0%	0%	73%	0.545	F
Na N	2016	NLR	Renal	PFS	8	1115*	58.02	< 0.001	0.398	0.125	88%	78%	93%	0.046	F
Luo Y	2015	NLR	Renal	OS	14	3373	18.39	0.104	0.185	0.034	35%	0%	66%	0.017	F
Luo Y	2015	NLR	Renal	RFS	3	700	2.02	0.364	0.079	0.116	1%	0%	90%	0.392	T
Luo Y	2015	NLR	Renal	CSS	4	1728	21.92	< 0.001	0.309	0.100	86%	67%	94%	0.534	T
Shen M	2014	IN	Renal	OS	2	206	0.67	0.411	< 0.001	0.093	0%	NA	NA	NA	T
Semeniuk-Wojtas A	2018	NLR	Renal (TKI)	OS	7	NA†	10.29	0.113	0.220	0.065	42%	0%	75%	0.385	F
Semeniuk-Wojtas A	2018	NLR	Renal (TKI)	PFS	6	NA†	48.32	< 0.001	0.439	0.166	90%	80%	95%	0.120	F
Boissier R	2017	NLR	Renal Advanced	OS	7	1304 (331/337)*	19.39	0.004	0.299	0.082	69%	32%	86%	0.009	T
Boissier R	2017	NLR	Renal Advanced	PFS	3	306 (143/163)	0.50	0.779	< 0.001	0.102	0%	0%	58%	0.981	T
Boissier R	2017	NLR	Renal Localised	OS	3	1168 (403/651)*	7.84	0.020	0.398	0.234	74%	15%	92%	0.219	F
Boissier R	2017	NLR	Renal Localised	RFS	6	1962 (487/817)*	28.36	< 0.001	0.405	0.144	82%	63%	92%	0.007	F
Li LQ	2020	NLR	STS	DFS	7	1239	4.64	0.590	< 0.001	0.035	0%	0%	62%	0.125	F
Liu G	2018	NLR	STS	OS	13	2658*	49.41	< 0.001	0.243	0.044	76%	58%	86%	0.018	T
Li LQ	2020	NLR	STS (Synovial Sarcoma)	OS	3	NA†	1.35	0.510	< 0.001	0.147	0%	0%	85%	0.464	T
Li LQ	2020	NLR	STS (Liposarcoma)	OS	2	NA†	0.00	0.952	< 0.001	0.225	0%	NA	NA	NA	F
Feng J	2020	NLR	Thyroid	DFS	7	6343	17.61	0.007	0.710	0.473	66%	24%	85%	0.207	T
Luo Y	2015	NLR	Upper Urinary	RFS	2	802	1.13	0.287	0.105	0.133	12%	NA	NA	NA	F
Wei Y	2014	NLR	Urinary	OS	13	3391	28.41	0.005	0.279	0.058	58%	22%	77%	0.001	F
Su S	2019	NLR	Urothelial	OS	7	2554	13.04	0.042	0.241	0.063	54%	0%	80%	0.844	T
Su S	2019	NLR	Urothelial	DFS	2	400	2.43	0.119	0.270	0.176	59%	0%	90%	NA	T
Vartolomei M	2018	NLR	Urothelial	RFS	6	3563	14.13	0.015	0.299	0.100	65%	15%	85%	0.021	F
Suh J	2019	NLR	Urothelial (Chemotherapy)	OS	5	1664	5.69	0.224	0.089	0.015	30%	0%	73%	0.131	T

Suh J	2019	NLR	Urothelial (Chemotherapy)	PFS	2	500	2.75	0.097	0.139	0.043	64%	0%	92%	NA	T
Suh J	2019	NLR	Urothelial (Nephroureterectomy)	OS	3	1103	1.54	0.463	< 0.001	0.067	0%	0%	86%	0.544	F
Suh J	2019	NLR	Urothelial (Nephroureterectomy)	PFS	8	4411	17.85	0.013	0.294	0.089	61%	15%	82%	0.011	F
Suh J	2019	NLR	Urothelial (Radical Cystectomy)	CSS	9	6974	69.19	< 0.001	0.333	0.073	88%	80%	93%	0.001	F
Suh J	2019	NLR	Urothelial (Radical Cystectomy)	OS	10	7049	55.80	< 0.001	0.380	0.086	84%	72%	91%	< 0.001	F
Suh J	2019	NLR	Urothelial (Radical Cystectomy)	PFS	5	5935	21.73	< 0.001	0.149	0.021	82%	57%	92%	0.027	T
Mori K	2020	NLR	UTUC (Nephroureterectomy)	CSS	12	6350	22.36	0.022	0.220	0.050	51%	5%	75%	0.104	F
Mori K	2020	NLR	UTUC (Radical Cystectomy)	CSS	12	11158	50.31	< 0.001	0.175	0.020	78%	62%	87%	< 0.001	F

=number of component studies with in review, (NA) Not applicable, (T) True, (F) False, (HR), Hazard ratio, (NLR) Neutrophil to lymphocyte ratio, (IN) Intratumoural neutrophils, (PN) Peritumoural neutrophils, (SN) Stromal Neutrophils,)S) Overall Survival, (DFS) Disease-Free Survival, (PFS) Progression-Free Survival, (RFS) Reoccurrence-Free Survival, (CSS) Cancer Specific Survival, (VEGFR) Vascular Endothelial Growth Factor Receptor, (PC) Palliative Chemotherapy, BR) Surgical Bowel Resection, (NAC) Neoadjuvant Chemotherapy, (STS) Soft Tissue Sarcoma, (UTR) Urothelial Transurethral Resection, (SR) Surgical Resection, (GNT) Gastroenteropancreatic Neuroendocrine Tumors, (SCC) Squamous ell Carcinoma, (ST) Systematic Therapy, (MT) Mixed Therapy, (TT) Targeted Therapy, (CLM) Colorectal Liver Metastasis, (NS) Non-surgical, (ICC) Intrahepatic Cholangiocarcinoma, (RFA) Radiofrequency Ablation, (TACE) Trans-Arterial hemoembolization, (MPM) Malignant Pleural Mesothelioma, (NSCLC) Non-Small Cell Lung Cancer, (NHC), Neck and Head Cancer, (UTUC) Upper Tract Urothelial Carcinoma, (DCRT) Definitive Chemoradiotherapy, (CR) Castration esistant, (TKI) Tyrosine Kinase Inhibitor.

All-cancers are defined as a grouping of cancer diagnosis unrelated to site, stage or treatment unless otherwise specified.

eview reported incomplete data on sample size.

Appendix F: Results of subgroup analyses of adjusted versus unadjusted effect estimates

Author	Year	Biomarker	Outcome Measure	Cancer Site	Adjustment	N	Random Effects			Fixed Effects			95% Prediction Interval		Test for subgroup differences (random)	
							HR	LL	UL	HR	LL	UL	LL	UL	Q	p-val
Paramanathan A	2014	NLR	DFS	All Cancers	univariate	2	1.93	1.71	2.18	1.93	1.71	2.18	NA	NA	0.69	0.4059
					multivariate	18	2.16	1.71	2.72	1.29	1.27	1.30	0.78	5.97		
Dolan R	2017	NLR	OS	All Cancers Operable	univariate	20	1.64	1.43	1.87	1.26	1.20	1.31	1.02	2.63	0.05	0.82
					multivariate	100	1.67	1.55	1.79	1.16	1.15	1.17	0.95	2.92		
Dolan R	2017	NLR	CSS	All Cancers Operable	univariate	7	1.16	1.07	1.27	1.16	1.07	1.27	1.04	1.31	15.55	0.0001
					multivariate	34	1.58	1.40	1.79	1.06	1.04	1.07	0.87	2.87		
Tan Q	2018	NLR	OS	ICI	univariate	5	2.56	1.45	4.52	2.01	1.51	2.69	0.44	15.08	0.30	0.585
					multivariate	13	2.14	1.57	2.91	1.05	1.00	1.10	0.73	6.27		
Tan Q	2018	NLR	PFS	ICI	univariate	5	1.90	1.39	2.60	1.76	1.42	2.19	0.79	4.54	0.36	0.5465
					multivariate	6	1.65	1.20	2.28	1.04	0.99	1.09	0.59	4.63		
Zheng DC	2016	NLR	PFS	Colorectal	univariate	3	1.49	0.91	2.45	1.51	1.08	2.12	0.01	225.93	1.25	0.2644
					multivariate	11	2.08	1.53	2.81	1.75	1.50	2.05	0.83	5.20		
Zhou Y	2018	NLR	OS	Pancreas	univariate	2	1.61	0.68	3.85	1.73	1.38	2.16	NA	NA	0.07	0.7918
					multivariate	41	1.82	1.59	2.07	1.34	1.28	1.40	0.90	3.66		
Mowbray N	2018	NLR	OS	Pancreas resect	univariate	2	1.48	1.03	2.12	1.48	1.04	2.10	NA	NA	0.86	0.3526
					multivariate	6	1.79	1.50	2.13	1.78	1.50	2.10	1.35	2.37		
Wu J	2017	NLR	OS	Cervical	univariate	4	1.47	1.17	1.84	1.47	1.17	1.84	0.90	2.41	0.02	0.9003
					multivariate	8	1.51	1.11	2.03	1.20	1.15	1.25	0.59	3.85		
Wu J	2017	NLR	PFS	Cervical	univariate	4	1.63	1.28	2.08	1.63	1.28	2.08	0.96	2.78	0.10	0.7517
					multivariate	6	1.76	1.19	2.62	1.18	1.13	1.23	0.43	7.21		
Zhou Q	2017	NLR	PFS	Ovarian	univariate	2	1.51	0.69	3.30	1.05	1.02	1.08	NA	NA	0.02	0.8934
					multivariate	10	1.60	1.26	2.02	1.37	1.26	1.48	0.76	3.34		
Liu X	2017	NLR	DFS	Breast	univariate	2	1.40	0.88	2.24	1.40	0.88	2.24	NA	NA	0.70	0.4025
					multivariate	10	1.78	1.32	2.39	1.05	1.00	1.10	0.75	4.22		
Tang H	2017	NLR	OS	BT	univariate	2	1.40	1.09	1.80	1.40	1.09	1.80	NA	NA	0.44	0.508
					multivariate	12	1.54	1.36	1.75	1.50	1.36	1.66	1.16	2.05		
Tang H	2017	NLR	RFS	BT	univariate	2	1.30	1.07	1.58	1.30	1.07	1.58	NA	NA	1.53	0.2158
					multivariate	3	1.78	1.13	2.82	1.55	1.21	1.99	0.01	271.58		

Zhao QT	2015	NLR	PFS	Lung	univariate	2	0.96	0.34	2.72	0.78	0.59	1.02	NA	NA	0.60	0.437
					multivariate	7	1.46	1.21	1.77	1.26	1.19	1.34	0.87	2.47		
Zhao QT	2015	NLR	OS	Lung	univariate	4	1.22	0.74	2.04	1.34	1.19	1.50	0.11	13.51	1.05	0.3046
					multivariate	17	1.61	1.40	1.86	1.19	1.14	1.24	0.99	2.64		
Su L	2017	NLR	CSS	Nasopharyngeal	univariate	1	1.72	0.78	3.79	1.72	0.78	3.79	NA	NA	0.20	0.6586
					multivariate	2	1.43	1.16	1.76	1.41	1.19	1.66	NA	NA		
Yu Y	2018	NLR	RFS	Head and Neck	univariate	1	2.62	1.23	5.58	2.62	1.23	5.58	NA	NA	1.78	0.1825
					multivariate	3	1.47	0.99	2.18	1.30	1.09	1.54	0.02	110.5		
Wang Y	2018	NLR	OS	OSCC	univariate	2	1.49	0.86	2.59	1.47	0.95	2.28	NA	NA	0.07	0.7941
					multivariate	4	1.62	1.25	2.10	1.58	1.26	1.98	0.78	3.39		
Wang Y	2018	NLR	DFS	OSCC	univariate	2	2.56	1.19	5.54	2.56	1.19	5.54	NA	NA	0.58	0.4453
					multivariate	4	1.86	1.38	2.49	1.86	1.38	2.49	0.97	3.55		
Zhan H	2018	NLR	OS	Melanoma	univariate	1	1.81	0.69	4.75	1.81	0.69	4.75	NA	NA	0.06	0.7988
					multivariate	9	1.59	1.24	2.03	1.10	1.06	1.14	0.69	3.69		
Li X	2017	NLR	CSS	UUB	univariate	5	1.63	1.38	1.93	1.62	1.39	1.90	1.18	2.26	0.02	0.8965
					multivariate	12	1.66	1.34	2.06	1.27	1.18	1.38	0.85	3.23		
Xiao WK	2014	NLR	OS	HCC MT	univariate	1	2.77	1.29	5.96	2.77	1.29	5.96	NA	NA	1.24	0.266
					multivariate	2	1.74	1.29	2.34	1.74	1.29	2.34	NA	NA		
Li X	2017	NLR	PFS	UUB	univariate	6	1.64	1.36	1.97	1.64	1.36	1.97	1.26	2.12	0.25	0.6197
					multivariate	13	1.76	1.42	2.18	1.42	1.27	1.60	0.94	3.28		
Cao J	2016	NLR	PFS	Prostate CR	univariate	4	1.40	1.23	1.61	1.40	1.23	1.61	1.04	1.89	7.85	0.0051
					multivariate	2	2.67	1.74	4.11	2.67	1.74	4.11	NA	NA		
Luo Y	2015	NLR	OS	Renal	univariate	1	1.78	1.45	2.19	1.78	1.45	2.19	NA	NA	0.53	0.4648
					multivariate	12	1.98	1.63	2.41	1.80	1.59	2.04	1.16	3.37		
Luo Y	2015	NLR	CSS	Renal	univariate	1	2.06	1.57	2.70	2.06	1.57	2.70	NA	NA	12.16	0.0005
					multivariate	3	1.12	0.91	1.38	1.07	0.94	1.21	0.15	8.57		
Luo Y	2015	NLR	RFS	Renal	univariate	2	2.03	1.06	3.88	1.90	1.23	2.93	NA	NA	0.02	0.8985
					multivariate	1	2.16	1.09	4.27	2.16	1.09	4.27	NA	NA		
Wu S	2018	NLR	OS	Bladder (Metastatic)	univariate	3	1.68	1.39	2.03	1.68	1.39	2.03	0.49	5.79	0.30	0.5854
					multivariate	1	1.49	1.02	2.18	1.49	1.02	2.18	NA	NA		
Zheng DC	2016	NLR	OS	Colorectal	univariate	2	2.53	1.40	4.57	2.53	1.40	4.57	NA	NA	0.86	0.3532
					multivariate	19	1.88	1.54	2.30	1.57	1.43	1.72	0.91	3.91		
Zhang X	2018	NLR	OS	Oesophageal	univariate	2	1.98	0.97	4.02	1.61	1.26	2.04	NA	NA	0.61	0.4348

					multivariate	24	1.48	1.29	1.70	1.35	1.28	1.43	0.83	2.64		
Chen	2017	NLR	OS	MPM	univariate	2	1.27	0.48	3.39	1.64	1.12	2.39	NA	NA	0.10	0.7564
					multivariate	8	1.50	1.12	2.01	1.25	1.15	1.36	0.58	3.89		
Zhang X	2018	NLR	OS	Oesophageal (Surgery)	univariate	2	1.98	0.97	4.02	1.61	1.26	2.04	NA	NA	0.68	0.4085
					multivariate	19	1.45	1.24	1.71	1.32	1.24	1.40	0.76	2.77		
Zhang X	2018	NLR	CSS	Oesophageal	univariate	2	1.53	0.79	2.98	1.63	1.29	2.05	NA	NA	0.12	0.7247
					multivariate	5	1.35	1.06	1.71	1.33	1.10	1.62	0.74	2.46		
Zhang X	2018	NLR	OS	Oesophageal Squamous Cell Carcinoma	univariate	2	1.98	0.97	4.02	1.61	1.26	2.04	NA	NA	1.05	0.3057
					multivariate	17	1.35	1.18	1.55	1.38	1.28	1.48	0.83	2.21		
Lei Y	2019	NLR	OS	Glioma	univariate	3	1.69	1.08	2.64	1.74	1.42	2.14	0.01	305.73	0.29	0.5872
					multivariate	13	1.48	1.22	1.79	1.03	1.00	1.05	0.78	2.81		
Lu Y	2020	NLR	OS	NSCLC	univariate	1	0.86	0.64	1.15	0.86	0.64	1.15	NA	NA	10.58	0.0011
					multivariate	20	1.44	1.29	1.61	1.32	1.25	1.40	0.97	2.15		
Tham T	2018	NLR	OS	Head and Neck	univariate	2	2.01	1.28	3.16	2.01	1.28	3.16	NA	NA	0.46	0.4955
					multivariate	24	1.71	1.49	1.96	1.62	1.46	1.79	1.15	2.55		
Tham T	2018	NLR	DFS	Head and neck	univariate	2	1.81	1.25	2.62	1.81	1.25	2.62	NA	NA	0.16	0.6904
					multivariate	9	1.64	1.22	2.21	1.43	1.18	1.74	0.77	3.49		
Cho J	2018	NLR	OS	Head and Neck (Squamous Cell Carcinoma)	univariate	6	2.33	1.76	3.07	2.32	1.80	3.00	1.42	3.80	1.71	0.1913
					multivariate	18	1.86	1.55	2.24	1.67	1.49	1.88	1.03	3.37		
Cho J	2018	NLR	DFS	Head and Neck (Squamous Cell Carcinoma)	univariate	3	3.16	1.18	8.45	2.34	1.52	3.62	0.00	199856.61	1.44	0.2307
					multivariate	9	1.69	1.26	2.25	1.36	1.19	1.56	0.72	3.93		
Yang L	2019	NLR	CSS	Head and Neck (Squamous Cell Carcinoma)	univariate	2	1.40	0.97	2.01	1.40	0.97	2.01	NA	NA	0.53	0.4656
					multivariate	3	1.80	1.01	3.19	1.48	1.23	1.78	0.00	1651.83		
Yang L	2019	NLR	OS	Hypopharynx	univariate	1	2.99	1.91	4.67	2.99	1.91	4.67	NA	NA	0.06	0.8037
					multivariate	2	2.75	1.66	4.54	2.75	1.66	4.54	NA	NA		
Yang L	2019	NLR	DFS	Oropharynx	univariate	1	1.72	1.04	2.85	1.72	1.04	2.85	NA	NA	0.01	0.9181
					multivariate	2	1.66	1.12	2.48	1.66	1.12	2.48	NA	NA		
Guan Y	2020	NLR	OS	Prostate CR (Abitaterone)	univariate	3	1.70	1.11	2.59	1.51	1.25	1.83	0.01	205.11	0.02	0.8863
					multivariate	7	1.75	1.46	2.10	1.75	1.46	2.10	1.38	2.22		
Guan Y	2020	NLR	OS	Prostate CR (Enzalutamide)	univariate	2	2.07	1.59	2.69	2.07	1.59	2.69	NA	NA	9.26	0.0023
					multivariate	3	1.35	1.24	1.46	1.35	1.24	1.46	0.80	2.26		
Guan Y	2020	NLR	PFS	Prostate CR (Enzalutamide)	univariate	1	1.98	1.53	2.56	1.98	1.53	2.56	NA	NA	9.97	0.0016
					multivariate	1	1.24	1.08	1.42	1.24	1.08	1.42	NA	NA		

Feng J	2020	NLR	DFS	Thyroid	univariate	5	1.47	0.67	3.23	1.17	0.98	1.39	0.11	20.43	2.02	0.1555
					multivariate	2	3.51	1.42	8.69	3.51	1.42	8.69	NA	NA		

N=number of component studies with in review, (LL) Lower Limited, (UL) Upper Limit, (NA) Not applicable, (T) True, (F) False, (HR), Hazard ratio, (NLR) Neutrophil to lymphocyte ratio, (IN) Intratumoural neutrophils, (PN) Peritumoural neutrophils, (SN) Stromal Neutrophils, (OS) Overall Survival, (DFS) Disease-Free Survival, (PFS) Progression-Free Survival, (RFS) Reoccurrence-Free Survival, (CSS) Cancer Specific Survival, (VEGFR) Vascular Endothelial Growth Factor Receptor, (PC) Palliative Chemotherapy, (SBR) Surgical Bowel Resection, (NAC) Neoadjuvant Chemotherapy, (STS) Soft Tissue Sarcoma, (UTR) Urothelial Transurethral Resection, (SR) Surgical Resection, (GNT) Gastroenteropancreatic Neuroendocrine Tumors, (SCC) Squamous Cell Carcinoma, (ST) Systematic Therapy, (MT) Mixed Therapy, (TT) Targeted Therapy, (CLM) Colorectal Liver Metastasis, (NS) Non-surgical, (ICC) Intrahepatic Cholangiocarcinoma, (RFA) Radiofrequency Ablation, (TACE) Trans-Arterial Chemoembolization, (MPM) Malignant Pleural Mesothelioma, (NSCLC) Non-Small Cell Lung Cancer, (NHC), Neck and Head Cancer, (UTUC) Upper Tract Urothelial Carcinoma, (DCRT) Definitive Chemoradiotherapy, (CR) Castration Resistant, (TKI) Tyrosine Kinase Inhibitor.

*All-cancers are defined as a grouping of cancer diagnosis unrelated to site, stage or treatment unless otherwise specified.
†review reported incomplete data on sample size.

Appendix G: Meta-regression results of NLR cut-off values

Author	Year	Site	Outcome	N	Regression coefficients				Tau^2	I^2	H^2	R^2	Test for residual heterogeneity		Test of Moderators	
					Intercept	p-val	NLR	p-val					QE	p-val	QM	p-val
Jiang T	2018	Advanced Cancer (Anti-VEGFR)	OS	14	-0.789	0.205	0.471	0.017	0.087	79%	4.681	34%	72.859	<0.001	5.737	0.017
Jiang T	2018	Advanced Cancer (Immunotherapy)	OS	23	0.123	0.700	0.157	0.082	0.074	72%	3.550	16%	87.993	<0.001	3.032	0.082
Jiang T	2018	Advanced Cancer (Immunotherapy)	PFS	16	0.796	0.060	-0.063	0.606	0.104	88%	8.373	0%	103.940	<0.001	0.265	0.606
Mei Z	2017	Composite endpoints	OS	62	0.384	0.021	0.036	0.413	0.058	82%	5.573	0%	437.578	<0.001	0.669	0.413
Mei Z	2017	Composite endpoints	PFS	23	0.107	0.691	0.112	0.150	0.060	88%	8.205	7%	191.392	<0.001	2.072	0.150
Paramanathan A	2014	Composite endpoints	DFS	20	0.329	0.411	0.105	0.282	0.196	99%	196.092	0%	525.841	<0.001	1.156	0.282
Hu G	2019	Bladder	OS	17	0.559	0.119	-0.077	0.539	0.091	96%	24.628	0%	68.824	<0.001	0.378	0.539
Wu S	2018	Bladder	RFS	8	1.142	0.216	-0.266	0.440	0.047	78%	4.512	0%	22.656	0.001	0.597	0.440
Hu G	2019	Bladder (Radical Cystectomy and NAC)	OS	4	-0.081	0.409	0.039	0.137	0	0%	1.000	100%	1.488	0.475	2.209	0.137
Hu G	2019	Bladder (Radical Cystectomy)	OS	8	0.401	0.676	-0.070	0.852	0.045	93%	13.910	0%	20.803	0.002	0.035	0.852
Suh J	2019	Bladder (UTR)	PFS	4	-0.576	0.870	0.631	0.675	0	0%	1	0%	1.535	0.464	0.176	0.675
Suh J	2019	Bladder (UTR)	RFS	5	-0.259	0.798	0.452	0.294	0	0%	1.000	94%	3.926	0.270	1.102	0.294
Ethier JL	2017	Breast	OS	13	1.582	0.001	-0.197	0.141	0.089	46%	1.841	11%	20.834	0.035	2.164	0.141
Liu X	2017	Breast	DFS	11	0.550	0.048	-0.010	0.921	0.003	3%	1.030	0%	11.678	0.232	0.010	0.921
Chen J	2018	Breast (Metastatic)	DFS	3	0.587	0.576	0.052	0.865	0	0%	1.000	NA	0.019	0.889	0.029	0.865
Duan J	2018	Breast (SR)	DFS	11	1.191	0.028	-0.212	0.263	0.079	53%	2.127	0%	18.630	0.029	1.254	0.263
Duan J	2018	Breast (SR)	OS	10	1.977	0.065	-0.348	0.322	0.248	61%	2.579	0%	19.344	0.013	0.980	0.322
Guo W	2019	Breast (Triple Negative and Her2 Positive)	DFS	27	0.474	0.048	-0.002	0.982	0.044	49%	1.944	0%	51.454	0.001	0.001	0.982
Guo W	2019	Breast (Triple Negative and Her2 Positive)	OS	28	0.435	0.222	0.051	0.677	0.131	70%	3.350	0	88.623	<0.001	0.173	0.677
Liu X	2017	Breast (Triple Negative)	OS	4	1.656	0.039	-0.229	0.355	0	0%	1.000	NA	1.624	0.444	0.856	0.355
Liu X	2017	Breast (Triple Negative)	DFS	4	-0.079	0.963	0.615	0.434	0	0%	1.000	NA	1.216	0.544	0.613	0.434
Tang H	2017	Biliary Tract	OS	14	0.415	0.064	0.002	0.981	0.011	25%	1.334	0	15.114	0.235	0.001	0.981
Tang H	2017	Biliary Tract	RFS	5	-0.275	0.513	0.207	0.123	0.011	25%	1.336	0	4.413	0.220	2.374	0.123
Wu J	2017	Cervical	OS	12	-0.351	0.565	0.305	0.223	0.063	60%	2.501	0	25.436	0.005	1.482	0.223
Wu J	2017	Cervical	PFS	10	0.525	0.501	0.007	0.982	0.151	87%	7.494	0	86.656	<0.001	0.001	0.982
Tang H	2016	CLM	OS	7	0.723	0.237	0.011	0.929	0.002	3%	1.034	NA	4.153	0.528	0.008	0.929
Tang H	2016	CLM	RFS	6	-0.300	0.423	0.224	0.008	0	0%	1.000	100%	2.025	0.731	7.147	0.008
Tang H	2016	CLM (SR)	OS	5	0.773	0.202	-0.009	0.945	0	0%	1.000	NA	2.019	0.568	0.005	0.945
Tang H	2016	CLM (SR)	RFS	4	-0.408	0.331	0.240	0.010	0	0%	1.000	100%	0.604	0.740	6.683	0.010

Zheng DC	2016	Colorectal	OS	21	0.024	0.954	0.142	0.129	0.079	59%	2.441	24%	40.665	0.003	2.299	0.129
Zheng DC	2016	Colorectal	PFS	14	0.287	0.758	0.081	0.688	0.142	61%	2.545	0%	27.803	0.006	0.161	0.688
Malietzis G	2014	Colorectal (Chemotherapy)	DFS	3	0.312	0.502	0.029	0.801	0	0%	1.000	NA	0.300	0.584	0.063	0.801
Li H	2019	Colorectal (SR)	DFS	9	0.840	0.091	-0.110	0.405	0.107	57%	2.330	0%	16.256	0.023	0.693	0.405
Li H	2019	Colorectal (SR)	OS	11	0.470	0.204	0.014	0.892	0.071	59%	2.445	0%	21.924	0.009	0.018	0.892
Li H	2019	Colorectal (SR)	RFS	3	1.486	0.040	-0.168	0.358	0	0%	1	0%	0.869	0.351	0.846	0.358
Ni L	2020	Endometrial	OS	9	0.817	0.163	-0.008	0.971	0	0%	1	0	4.291	0.746	0.001	0.971
Ni L	2020	Endometrial	PFS	5	0.927	0.459	-0.123	0.811	0.122	48%	1.937	0%	5.831	0.120	0.057	0.811
Sun J	2016	Gastric	OS	19	0.346	0.090	0.110	0.094	0.016	40%	1.679	0.33	29.531	0.030	2.808	0.094
Sun J	2016	Gastric	DFS	7	0.767	0.077	-0.111	0.465	0	0%	1.000	NA	2.693	0.747	0.533	0.465
Szor D	2018	Gastric (SR)	OS	7	0.263	0.723	0.293	0.214	0.262	80%	5.012	11%	32.467	<0.001	1.544	0.214
Mellor K	2018	Gastric (SR)	DFS	3	2.070	0.259	-0.278	0.542	0.651	86%	7.244	0%	7.244	0.007	0.372	0.542
Lei Y	2019	Glioma	OS	16	0.820	0.008	-0.098	0.176	0.070	85%	6.51147	13%	69.901	<0.001	1.835	0.176
Zhang J	2017	Glioma	OS	6	1.102	0	-0.147	0.017	0.002	4%	1.039	95%	2.787	0.594	5.733	0.017
Zhou Y	2018	GNT	OS	4	0.648	0.352	0.181	0.475	0	0%	1.000	NA	1.496	0.473	0.510	0.475
Zhou Y	2018	GNT	RFS	3	1.461	0.496	-0.095	0.925	0.093	30%	1.424	0%	1.424	0.233	0.009	0.925
Ethier JL	2017	Gynecologic	OS	26	0.818	0.079	-0.096	0.548	0.095	81%	5.229	4%	87.001	<0.001	0.360	0.548
Xiao WK	2014	HCC (MT)	OS	3	0.145	0.710	0.141	0.194	0	0%	1.000	100%	0.282	0.596	1.684	0.194
Wang Y	2018	HCC (SR)	RFS	9	0.727	0.041	-0.081	0.528	0.019	29%	1.415	0%	8.497	0.291	0.399	0.528
Xu ZG	2018	HCC (Transplant)	OS	8	0.003	0.997	0.193	0.356	0.321	81%	5.208	0%	36.397	<0.001	0.853	0.356
Xu ZG	2018	HCC (Transplant)	RFS	4	-0.151	0.926	0.373	0.356	0.246	69%	3.204	0%	7.106	0.029	0.850	0.356
Yu Y	2018	Head and Neck	RFS	4	-1.127	0.023	0.424	0.004	0	0%	1.000	100%	1.120	0.571	8.446	0.004
Tham T	2018	Head and Neck	OS	24	-0.025	0.905	0.176	0.008	0.011	14%	1.161	62%	24.905	0.302	7.037	0.008
Tham T	2018	Head and neck	PFS	5	0.186	0.930	0.067	0.930	0.077	67%	3.052	0%	9.158	0.027	0.008	0.930
Tham T	2018	Head and neck	DFS	10	0.127	0.770	0.120	0.332	0.068	41%	1.683	3%	13.679	0.091	0.941	0.332
Tham T	2018	Head and Neck (No Surgery)	OS	8	0.027	0.938	0.103	0.356	0.007	9%	1.094	0%	5.264	0.510	0.851	0.356
Yang L	2019	Head and Neck (SCC)	CSS	4	0.216	0.472	0.052	0.683	0	0%	1.000	0%	0.603	0.740	0.167	0.683
Yang L	2019	Head and Neck (SCC)	PFS	3	7.800	0.706	-2.491	0.719	0.081	73%	3.755	0%	3.755	0.053	0.129	0.719
Cho J	2018	Head and Neck (SCC)	OS	24	0.313	0.250	0.108	0.161	0.072	50%	1.997	0%	41.800	0.007	1.969	0.161
Cho J	2018	Head and Neck (SCC)	DFS	12	0.093	0.828	0.167	0.176	0.143	70%	3.301	5%	34.232	<0.001	1.834	0.176
Tham T	2018	Head and Neck (Surgery)	OS	3	4.055	0.018	-1.457	0.034	0	0%	1	100%	0.433	0.510	4.472	0.034
Min GT	2018	Hepatocellular	OS	44	0.140	0.532	0.106	0.097	0.178	93%	15.368	3%	214.542	<0.001	2.751	0.097
Min GT	2018	Hepatocellular	DFS	17	0.141	0.701	0.145	0.194	0.180	87%	7.708	10%	77.786	<0.001	1.688	0.194

Min GT	2018	Hepatocellular	RFS	16	-0.315	0.341	0.324	0.004	0.087	74%	3.809	38%	44.743	<0.001	8.134	0.004
Liu L	2019	Hepatocellular (Sorfenib)	OS	11	-0.079	0.864	0.196	0.180	0.032	45%	1.819	26%	16.506	0.057	1.800	0.180
Wang Y	2018	Hepatocellular (SR)	OS	13	0.532	0.131	-0.020	0.881	0.023	36%	1.562	0%	17.045	0.107	0.022	0.881
Wang Y	2018	Hepatocellular (SR)	DFS	7	0.245	0.367	0.063	0.536	0.001	5%	1.058	0%	8.160	0.148	0.383	0.536
Li S	2020	Hepatocellular (TACE)	OS	16	0.626	0.001	-0.003	0.961	0.025	43%	1.768	0%	28.035	0.014	0.002	0.961
Yang L	2019	Hypopharynx	OS	3	0.835	0.261	0.080	0.758	0	0%	1	0%	0.853	0.356	0.095	0.758
Tan Q	2018	Immunotherapy (Immune checkpoint inhibitors)	OS	18	0.729	0.409	0.016	0.934	0.215	78%	4.550	0%	86.955	<0.001	0.007	0.934
Tan Q	2018	Immunotherapy (Immune checkpoint inhibitors)	PFS	11	-0.189	0.827	0.161	0.386	0.085	68%	3.139	7%	31.583	<0.001	0.753	0.386
Xie X	2019	Ipilimumuab	OS	6	-3.358	0.131	0.923	0.051	0.243	82%	5.696	43%	19.012	0.001	3.794	0.051
Yang L	2019	Larynx	DFS	3	-0.924	0.315	0.721	0.178	0	0%	1	100%	0.027	0.870	1.810	0.178
Mascarella M	2018	Larynx	OS	6	0.419	0.746	0.025	0.958	0.034	30%	1.420	0%	5.444	0.245	0.003	0.958
Zhao QT	2015	Lung	OS	21	0.492	0.150	-0.013	0.873	0.090	88%	8.251	0%	101.987	<0.001	0.025	0.873
Zhao QT	2015	Lung	PFS	9	1.480	0.044	-0.280	0.107	0.140	80%	5.064	16%	36.750	<0.001	2.593	0.107
Yu Y	2017	Lung Surgery	OS	7	0.464	0.196	-0.017	0.873	0.050	79%	4.770	0%	26.900	<0.001	0.026	0.873
Zhan H	2018	Melanoma	OS	10	-0.091	0.831	0.156	0.178	0.098	90%	10.282	7%	91.782	<0.001	1.812	0.178
Ding Y	2018	Melanoma	PFS	6	1.044	0.104	-0.053	0.701	0.011	13%	1.146	0	3.721	0.445	0.148	0.701
Xie X	2019	Melanoma (Immune checkpoint inhibitors)	OS	8	-3.395	0.056	0.916	0.015	0.149	72%	3.622	52%	19.482	0.003	5.930	0.015
Chen	2017	MPM	OS	10	-0.050	0.937	0.108	0.475	0.130	82%	5.520	0%	36.158	<0.001	0.510	0.475
Zeng Q	2018	Multiple Myeloma	OS	8	0.054	0.915	0.195	0.325	0.088	65%	2.847	3%	20.715	0.002	0.969	0.325
Zeng Q	2018	Multiple Myeloma	PFS	4	0.078	0.930	0.207	0.542	0.278	94%	15.956	0%	16.072	<0.001	0.371	0.542
Li X	2018	NAC	OS	18	-0.127	0.756	0.198	0.121	0.086	82%	5.693	22%	58.956	<0.001	2.410	0.121
Li X	2018	NAC	RFS	7	0.379	0.761	0.133	0.784	0.103	47%	1.882	0%	9.038	0.108	0.075	0.784
Li X	2018	NAC	CSS	5	0.574	0.481	0.077	0.755	0.250	66%	2.924	0%	9.322	0.025	0.097	0.755
Yin J	2017	Nasopharngeal	PFS	5	0.454	0.146	-0.009	0.920	0.012	27%	1.374	0%	3.651	0.302	0.010	0.920
Yang S	2019	Nasopharyngeal	OS	10	0.030	0.908	0.115	0.179	0	0%	1.000	100%	8.099	0.424	1.804	0.179
Su L	2017	Nasopharyngeal	CSS	3	-0.226	0.657	0.213	0.251	0	0%	1.000	100%	0.149	0.699	1.319	0.251
Xie X	2019	Nivolumab	OS	6	-1.433	0.268	0.501	0.061	0.039	20%	1.257	53%	4.197	0.380	3.521	0.061
Xie X	2019	Nivolumab	PFS	8	-0.829	0	0.294	0	0	0%	1.000	100%	4.432	0.618	48.012	0
Vartolomei MD	2018	Non muscle Invasive Bladder	RFS	6	-0.614	0.567	0.483	0.263	0.074	64%	2.780	0%	14.030	0.007	1.251	0.263
Vartolomei MD	2018	Non muscle Invasive Bladder	PFS	6	-0.180	0.861	0.403	0.320	0.037	18%	1.222	0%	3.282	0.512	0.990	0.320
Vartolomei MD	2018	Non muscle Invasive Bladder (High Risk)	RFS	4	3.429	0.409	-0.986	0.536	0.318	68%	3.172	0%	6.952	0.031	0.383	0.536
Vartolomei MD	2018	Non-muscle Invasive Bladder (High Risk)	PFS	3	-3.141	0.397	1.574	0.271	0	0%	1.000	NA	0.554	0.457	1.214	0.271
Lu Y	2020	NSCLC	OS	21	0.604	0.016	-0.070	0.287	0.041	70%	3.346	4%	52.946	<0.001	1.133	0.287
Gu XB	2015	NSCLC	PFS	10	0.448	0.410	0.006	0.964	0.074	67%	2.995	0%	28.495	<0.001	0.002	0.964
Wang Z	2019	NSCLC (Chemotherapy)	OS	8	1.576	0.009	-0.279	0.102	0.128	69%	3.206	27%	15.889	0.014	2.671	0.102

Wang Z	2019	NSCLC (Chemotherapy)	PFS	5	2.193	0.053	-0.558	0.141	0.005	10%	1.117	77%	2.914	0.405	2.162	0.141
Xie X	2019	NSCLC (Immune Checkpoint Inhibitors)	PFS	6	-0.676	0.001	0.243	0	0	0%	1.000	100%	2.392	0.664	15.231	0
Wang Z	2019	NSCLC (Immunotherapy)	OS	7	0.969	0.546	-0.030	0.922	0.214	76%	4.107	0	24.418	0	0.010	0.922
Cao D	2018	NSCLC (Nivoumab)	OS	8	-0.484	0	0.204	0	0	0%	1.000	100%	8.870	0.181	46.698	0
Cao D	2018	NSCLC (Nivoumab)	PFS	10	-0.390	0.674	0.185	0.376	0	93%	15.012	0%	56.851	<0.001	0.783	0.376
Jiang T	2019	NSCLC (PD-1 inhibitor)	OS	13	-0.235	0.541	0.273	0.003	0	0%	1.000	100%	6.690	0.824	8.686	0.003
Jiang T	2019	NSCLC (PD-1 inhibitor)	PFS	12	0.374	0.346	0.027	0.768	0	0%	1.000	0%	3.447	0.969	0.087	0.768
Wang Z	2019	NSCLC (ST)	OS	22	0.432	0.216	0.049	0.562	0.151	91%	10.731	0%	109.798	<0.001	0.337	0.562
Wang Z	2019	NSCLC (ST)	PFS	15	0.350	0.196	0.011	0.880	0.055	80%	4.994	0%	53.719	<0.001	0.023	0.880
Wang Z	2019	NSCLC (TT)	OS	5	-0.590	0.099	0.345	0.005	0.022	17%	1.201	86%	2.967	0.397	7.915	0.005
Wang Z	2019	NSCLC (TT)	PFS	5	-1.163	0	0.565	0	0	0%	1.000	100%	1.916	0.590	14.148	0
Zhang X	2018	Oesophageal	OS	26	0.206	0.294	0.064	0.280	0.068	75%	4.055	5%	83.809	<0.001	1.168	0.280
Zhang X	2018	Oesophageal	CSS	7	0.082	0.859	0.096	0.540	0.060	58%	2.389	0%	12.236	0.032	0.375	0.540
Zhang X	2018	Oesophageal	RFS	3	1.440	0.008	-0.352	0.071	0	0%	1.000	100%	0.108	0.742	3.249	0.071
Zhang X	2018	Oesophageal	DFS	10	-0.251	0.566	0.202	0.094	0.114	81%	5.317	27%	37.388	<0.001	2.798	0.094
Zhang X	2018	Oesophageal	PFS	4	0.300	0.486	0.018	0.867	0	27%	1.374	0%	2.267	0.322	0.028	0.867
Zhang X	2018	Oesophageal (No Surgery)	OS	4	0.278	0.616	0.071	0.607	0.070	58%	2.398	0%	4.809	0.090	0.264	0.607
Zhang X	2018	Oesophageal (Surgery)	OS	21	0.243	0.305	0.052	0.492	0.086	79%	4.841	0%	76.621	<0.001	0.471	0.492
Zhang X	2018	Oesophageal SCC	OS	19	0.378	0.087	-0.016	0.817	0.053	67%	3.046	0%	56.070	<0.001	0.053	0.817
Yang Y	2018	Oral cavity	OS	9	-0.316	0.618	0.348	0.174	0	27%	1.366	38%	9.188	0.239	1.851	0.174
Yang Y	2018	Oral cavity	DFS	10	0.513	0.320	0.013	0.949	0	0%	1.000	100%	8.501	0.386	0.004	0.949
Wang Y	2018	Oral SCC	OS	5	-1.195	0.198	0.679	0.076	0	0%	1.000	100%	1.231	0.746	3.143	0.076
Wang Y	2018	Oral SCC	DFS	6	0.527	0.411	0.052	0.831	0	0%	1.000	33%	3.418	0.490	0.046	0.831
Zhou Q	2017	Ovarian	PFS	12	-0.315	0.515	0.234	0.104	0.099	83%	5.926	9%	62.107	<0.001	2.648	0.104
Zhou Q	2017	Ovarian	OS	16	-0.300	0.597	0.236	0.154	0.152	84%	6.427	10%	73.819	<0.001	2.027	0.154
Zhou Y	2018	Pancreatic	OS	43	0.311	0.142	0.075	0.169	0.112	80%	4.994	6%	176.759	<0.001	1.893	0.169
Zhou Y	2018	Pancreatic	DFS	8	1.641	0.064	-0.250	0.189	0.143	63%	2.682	18%	15.204	0.019	1.723	0.189
Yang JJ	2015	Pancreatic	CSS	3	-0.204	0.680	0.182	0.141	0.051	50%	1.994	51%	1.994	0.158	2.163	0.141
Mowbray N	2018	Pancreatic (SR)	OS	8	0.369	0.083	0.060	0.384	0	0%	1.000	0%	9.395	0.153	0.759	0.384
Guo J	2018	Prostate	PFS	7	0.948	0.103	-0.073	0.641	0.064	37%	1.588	0%	7.911	0.161	0.218	0.641
Guo J	2018	Prostate (Metastatic)	OS	9	0.949	0.024	-0.126	0.290	0.035	43%	1.758	0%	12.582	0.083	1.118	0.290
Yin X	2016	Prostate CR	OS	8	0.763	0.002	-0.088	0.194	0.001	2%	1.021	0%	6.864	0.334	1.689	0.194
Guan Y	2020	Prostate CR (Abitaterone)	OS	10	0.375	0.357	0.053	0.632	0.046	48%	1.906	0%	14.941	0.060	0.230	0.632
Guan Y	2020	Prostate CR (Enzalutamide)	OS	5	1.003	0.351	-0.210	0.579	0	78%	4.600	0%	9.366	0.025	0.309	0.579
Dong YW	2016	Rectal	OS	6	0.458	0.862	0.733	0.390	0	0%	1.000	NA	1.042	0.903	0.739	0.390
Dong YW	2016	Rectal	DFS	3	-2.146	0.573	1.256	0.341	0	0%	1.000	NA	0.004	0.953	0.905	0.341
Dong YW	2016	Rectal	RFS	3	0.683	0.734	0.170	0.760	0	0%	1.000	NA	0.667	0.414	0.094	0.760
Na N	2016	Renal	PFS	7	0.954	0.531	-0.090	0.867	0.197	82%	5.558	0%	47.577	<0.001	0.028	0.867
Luo Y	2015	Renal	OS	13	0.607	0.196	0.016	0.906	0.041	46%	1.865	0%	18.121	0.079	0.014	0.906
Luo Y	2015	Renal	CSS	4	-1.165	0	0.476	0	0	0%	1.000	100%	0.594	0.743	21.326	<0.001
Luo Y	2015	Renal	RFS	3	1.425	0.068	-0.184	0.325	0.003	2%	1.020	53%	1.020	0.313	0.967	0.325
Semeniuk-Wojtas A	2018	Renal (TKI)	OS	7	0.020	0.975	0.243	0.238	0.040	35%	1.535	17%	6.487	0.262	1.392	0.238
Semeniuk-Wojtas A	2018	Renal (TKI)	PFS	6	1.101	0.513	-0.145	0.810	0.250	85%	6.485	0%	41.803	<0.001	0.058	0.810
Boissier R	2017	Renal Advanced	OS	6	-0.395	0.729	0.381	0.315	0.065	55%	2.198	0%	9.634	0.047	1.011	0.315

Boissier R	2017	Renal Advanced	PFS	3	-7.802	0.811	2.979	0.784	0	0%	1.000	NA	0.424	0.515	0.075	0.784
Boissier R	2017	Renal Localised	OS	3	1.311	0.449	-0.218	0.603	0.306	87%	7.693	0%	7.693	0.006	0.270	0.603
Boissier R	2017	Renal Localised	RFS	6	1.264	0.106	-0.221	0.321	0.174	81%	5.225	0%	18.851	0.001	0.985	0.321
Li LQ	2020	STS	DFS	7	0.323	0.158	0.089	0.291	0	0%	1.000	0%	3.531	0.619	1.113	0.291
Liu G	2018	STS	OS	13	0.749	0.044	-0.117	0.394	0	66%	2.911	7%	45.486	<0.001	0.725	0.394
Feng J	2020	Thyroid	DFS	7	3.210	0.153	-1.249	0.244	0.618	73%	3.725	0%	17.568	0.004	1.356	0.244
Wei Y	2014	Urinary	OS	12	1.023	0.010	-0.102	0.345	0.079	52%	2.074	0%	20.471	0.025	0.894	0.345
Su S	2019	Urothelial	OS	7	1.332	0.004	-0.578	0.015	0	0%	1.000	100%	7.176	0.208	5.864	0.015
Vartolomei MD	2018	Urothelial	RFS	6	1.725	0.403	-0.444	0.549	0.116	68%	3.096	0%	13.830	0.008	0.359	0.549
Suh J	2019	Urothelial (Chemotherapy)	OS	5	0.439	0.197	-0.016	0.863	0.013	48%	1.925	0%	5.661	0.129	0.030	0.863
Suh J	2019	Urothelial (Nephroureterectomy)	OS	3	1.816	0.271	-0.422	0.439	0	0%	1.000	0%	0.940	0.332	0.600	0.439
Suh J	2019	Urothelial (Nephroureterectomy)	PFS	8	1.151	0.069	-0.230	0.285	0.095	65%	2.852	0%	17.185	0.009	1.143	0.285
Suh J	2019	Urothelial (Radical Cystectomy)	CSS	8	0.627	0.316	-0.042	0.833	0.122	89%	9.288	0%	57.670	<0.001	0.045	0.833
Suh J	2019	Urothelial (Radical Cystectomy)	OS	8	0.677	0.352	-0.034	0.881	0.185	91%	10.918	0%	48.548	<0.001	0.022	0.881
Suh J	2019	Urothelial (Radical Cystectomy)	PFS	4	0.964	0.538	-0.225	0.666	0.110	85%	6.504	0%	11.251	0.004	0.186	0.666

N=number of component studies with in review, (NA) Not applicable, (T) True, (F) False, (HR) Hazard ratio, (NLR) Neutrophil to lymphocyte ratio, (IN) Intratumoural neutrophils, (PN) Peritumoural neutrophils, (SN) Stromal Neutrophils, (OS) Overall Survival, (DFS) Disease-Free Survival, (PFS) Progression-Free Survival, (RFS) Reoccurrence-Free Survival, (CSS) Cancer Specific Survival, (VEGFR) Vascular Endothelial Growth Factor Receptor, (PC) Palliative Chemotherapy, (SBR) Surgical Bowel Resection, (NAC) Neoadjuvant Chemotherapy, (STS) Soft Tissue Sarcoma, (UTR) Urothelial Transurethral Resection, (SR) Surgical Resection, (GNT) Gastroenteropancreatic Neuroendocrine Tumors, (SCC) Squamous Cell Carcinoma, (ST) Systematic Therapy, (MT) Mixed Therapy, (TT) Targeted Therapy, (CLM) Colorectal Liver Metastasis, (NS) Non-surgical, (ICC) Intrahepatic Cholangiocarcinoma, (RFA) Radiofrequency Ablation, (TACE) Trans-Arterial Chemoembolization, (MPM) Malignant Pleural Mesothelioma, (NSCLC) Non-Small Cell Lung Cancer, (NHC), Neck and Head Cancer, (UTUC) Upper Tract Urothelial Carcinoma, (DCRT) Definitive Chemoradiotherapy, (CR) Castration Resistant, (TKI) Tyrosine Kinase Inhibitor.

*All-cancers are defined as a grouping of cancer diagnosis unrelated to site, stage or treatment unless otherwise specified.
†review reported incomplete data on sample size.

Appendix H: Excess significance in included meta-analyses

Author	Year	Biomarker	Cancer site	meta-analysis metric	N	Sample size	Largest Study Effect Size			Fixed Effects Size			Random Effects Size			Largest Study O > E	Bias (Largest)
							O	E	P-val	O	E	P-val	O	E	P-val		
Jiang T	2018	NLR	Advanced Cancer (Anti-VEGFR)	OS	14	2918	13	13	NA	13	9	0.0252	13	13	NA	NA	No
Jiang T	2018	NLR	Advanced Cancer (Immunotherapy)	PFS	16	2318	13	10	0.1947	13	4	3.78E-06	13	13	NA	TRUE	None Detected
Mei Z	2017	NLR	Composite endpoints	OS	65	18262	59	26	<0.0001	59	8	2.20E-16	59	57	0.572	TRUE	Yes
Mei Z	2017	NLR	Composite endpoints	PFS	23	5607	19	21	NA	19	9	3.61E-05	19	20	NA	FALSE	No
Shen M	2014	IN	Composite endpoints	OS	16	3335	10	3	0.0001	10	11	NA	10	13	NA	TRUE	Yes
Shen M	2014	IN	Composite endpoints	CSS	2	437	2	2	NA	2	2	NA	2	2	NA	NA	No
Paramanathan A	2014	NLR	Composite endpoints	DFS	20	13065	17	8	8.39E-05	17	12	0.0224	17	20	NA	TRUE	Yes
Shen M	2014	PN	Composite endpoints	OS	2	535	1	0	<0.0001	1	1	NA	1	2	NA	TRUE	Yes
Shen M	2014	PN	Composite endpoints	OS	2	535	0	0	NA	0	0	NA	0	0	NA	NA	No (NM)
Dolan R	2017	NLR	Composite endpoints Operable	OS	120	68603	84	67	0.0017	84	48	3.58E-11	84	116	NA	TRUE	Yes
Dolan R	2017	NLR	Composite endpoints Operable	CSS	41	21175	28	7	5.44E-13	28	4	2.20E-16	28	37	NA	TRUE	Yes
Tang H	2017	NLR	Biliary Tract	OS	14	3217	10	5	0.0092	10	9	0.7819	10	9	0.7819	TRUE	Yes
Tang H	2017	NLR	Biliary Tract	RFS	5	637	3	1	0.0579	3	2	0.3952	3	2	0.3952	TRUE	Yes
Hu G	2019	NLR	Bladder	OS	18	11945	13	3	2.84E-07	13	2	1.96E-09	13	11	0.4695	TRUE	Yes
Wu S	2018	NLR	Bladder	RFS	9	8075	9	5	0.0057	9	6	0.0343	9	8	0.6103	TRUE	Yes
Wu S	2018	NLR	Bladder (Metastasis)	OS	4	862	3	4	NA	3	4	NA	3	4	NA	FALSE	No
Hu G	2019	NLR	Bladder (Radical Cystectomy and NAC)	OS	4	447	1	0	< 2.2e-16	1	0	< 2.2e-16	1	0	< 2.2e-16	TRUE	No (NM)
Hu G	2019	NLR	Bladder (Radical Cystectomy)	OS	8	10363	7	3	0.0056	7	1	3.40E-06	7	3	0.006	TRUE	Yes
Suh J	2019	NLR	Bladder (UTR)	PFS	4	1040	2	4	< 2.2e-16	2	4	< 2.2e-16	2	4	< 2.2e-16	FALSE	No
Suh J	2019	NLR	Bladder (UTR)	RFS	5	1129	5	5	NA	5	5	NA	5	5	NA	NA	No
Li X	2017	NLR	Bladder and Upper Urinary	PFS	19	8182	12	1	1.61E-11	12	15	NA	12	17	NA	TRUE	Yes
Li X	2017	NLR	Bladder and Upper Urinary	CSS	17	7645	13	2	1.24E-09	13	12	0.7916	13	16	NA	TRUE	Yes
Ethier JL	2017	NLR	Breast	OS	13	8015	13	12	0.6173	13	13	NA	13	13	NA	TRUE	None Detected
Liu X	2017	NLR	Breast	DFS	12	5523	7	9	NA	7	1	1.52E-05	7	10	NA	FALSE	No
Liu X	2017	NLR	Breast	CSS	4	4186	2	1	0.2617	2	2	NA	2	4	NA	TRUE	None Detected
Chen J	2018	NLR	Breast (Metastasis)	OS	2	858	2	2	NA	2	2	NA	2	2	NA	NA	No
Chen J	2018	NLR	Breast (Metastasis)	DFS	3	1003	2	3	NA	2	3	NA	2	3	NA	FALSE	No

Chen J	2018	NLR	Breast (No Metastasis)	OS	2	488	1	1	NA	1	2	NA	1	2	NA	NA	No
Chen J	2018	NLR	Breast (No Metastasis)	DFS	2	466	0	1	NA	0	0	NA	0	0	NA	FALSE	No
Duan J	2018	NLR	Breast (SR)	DFS	15	7820	9	15	< 2.2e-16	9	3	0.0008	9	14	0.0003	FALSE	No
Duan J	2018	NLR	Breast (SR)	OS	12	6233	10	12	< 2.2e-16	10	12	< 2.2e-16	10	12	< 2.2e-16	FALSE	No
Duan J	2018	NLR	Breast (SR)	RFS	2	319	2	2	NA	2	2	NA	2	2	NA	NA	No
Guo W	2019	NLR	Breast (Triple Negative and Her2 Positive)	DFS	27	11369	15	22	0.0018	15	23	0.0002	15	24	1.23E-05	FALSE	No
Guo W	2019	NLR	Breast (Triple Negative and Her2 Positive)	OS	28	14128	18	1	< 2.2e-16	18	22	0.1022	18	25	0.0004	TRUE	None Detected
Liu X	2017	NLR	Breast (Triple-negative)	OS	4	608	3	4	NA	3	4	NA	3	4	NA	FALSE	No
Liu X	2017	NLR	Breast (Triple-negative)	DFS	4	461	3	4	NA	3	4	NA	3	4	NA	FALSE	No
Wu J	2017	NLR	Cervical	OS	12	3661	5	4	0.5496	5	4	0.5496	5	7	NA	TRUE	None Detected
Wu J	2017	NLR	Cervical	PFS	10	2452	7	1	9.12E-06	7	2	0.0009	7	7	NA	TRUE	Yes
Zhang J	2017	NLR	Colorectal	DFS	5	2178	2	3	NA	2	5	NA	2	5	NA	FALSE	No
Zheng DC	2016	NLR	Colorectal	OS	21	7927	13	11	0.5133	13	17	NA	13	20	NA	TRUE	None Detected
Zheng DC	2016	NLR	Colorectal	PFS	14	3662	9	13	NA	9	12	NA	9	13	NA	FALSE	No
Malietzis G	2014	NLR	Colorectal (PC)	DFS	3	515	2	2	NA	2	2	NA	2	2	NA	NA	No
Li H	2019	NLR	Colorectal (SR)	DFS	9	4237	5	5	NA	5	8	0.01211	5	9	< 2.2e-16	NA	No
Li H	2019	NLR	Colorectal (SR)	OS	11	4832	7	7	NA	7	10	0.01336	7	10	0.0134	NA	No
Li H	2019	NLR	Colorectal (SR)	RFS	3	643	3	3	NA	3	3	NA	3	3	NA	NA	No
Tang H	2016	NLR	CLM	OS	7	1491	7	7	NA	7	7	NA	7	7	NA	NA	No
Tang H	2016	NLR	CLM	RFS	6	1093	4	6	NA	4	6	NA	4	6	NA	FALSE	No
Tang H	2016	NLR	CLM (Non-surgical)	OS	2	182	2	2	NA	2	2	NA	2	2	NA	NA	No
Tang H	2016	NLR	CLM (Non-surgical)	RFS	2	190	1	4	NA	1	4	NA	1	4	NA	FALSE	No
Tang H	2016	NLR	CLM (SR)	OS	5	1309	5	5	NA	5	5	NA	5	5	NA	NA	No
Tang H	2016	NLR	CLM (SR)	RFS	4	903	3	1	0.0508	3	2	0.625	3	2	0.625	TRUE	Yes
Ni L	2020	NLR	Endometrial	OS	9	2942	6	9	< 2.2e-16	6	9	< 2.2e-16	6	9	< 2.2e-16	FALSE	No
Ni L	2020	NLR	Endometrial	PFS	5	1798	3	5	< 2.2e-16	3	5	< 2.2e-16	3	5	< 2.2e-16	FALSE	No
Sun J	2016	NLR	Gastric	OS	19	5431	16	14	0.4353	16	18	NA	16	18	NA	TRUE	None Detected
Sun J	2016	NLR	Gastric	DFS	7	976	4	5	NA	4	5	NA	4	5	NA	FALSE	No
Shen M	2014	IN	Gastric	OS	2	388	1	2	NA	1	0	< 2.2e-16	1	1	NA	FALSE	No
Zhang X	2014	NLR	Gastric	PFS	2	314	2	1	0.5	2	1	0.5	2	1	0.5	TRUE	None Detected
Szor D	2018	NLR	Gastric (SR)	OS	7	3223	6	7	NA	6	7	NA	6	7	NA	FALSE	No

Mellor K	2018	NLR	Gastric (SR)	DFS	3	820	1	3	NA	1	3	NA	1	3	NA	FALSE	No
Zhou Y	2018	NLR	GNT	OS	4	422	4	4	NA	4	4	NA	4	4	NA	NA	No
Zhou Y	2018	NLR	GNT	RFS	3	300	3	3	NA	3	3	NA	3	3	NA	NA	No
Luo XF	2018	NLR	Gastrointestinal Stromal	OS	2	278	2	0	< 0.00001	2	0	< 2.2e-16	2	2	NA	TRUE	Yes
Lei Y	2019	NLR	Glioma	OS	16	2275	12	NA	NA	12	1	5.10E-12	12	10	0.4397	FALSE	No
Ethier JL	2017	NLR	Gynecologic	OS	27	10530	17	15	0.5622	17	12	0.0794	17	23	NA	TRUE	None Detected
Yu Y	2018	NLR	Head and Neck	RFS	4	658	2	3	NA	2	2	NA	2	3	NA	FALSE	No
Tham T	2018	NLR	Head and Neck	OS	26	6769	19	21	0.3201	19	22	0.1058	19	23	0.0246	FALSE	No
Tham T	2018	NLR	Head and neck	PFS	6	2173	4	5	0.2632	4	4	NA	4	4	NA	FALSE	No
Tham T	2018	NLR	Head and neck	DFS	11	1692	5	0	< 2.2e-16	5	7	0.2232	5	9	0.0072	TRUE	No (NM)
Shen M	2014	IN	Head and Neck	OS	3	220	1	2	NA	1	2	NA	1	2	NA	FALSE	No
Tham T	2018	NLR	Head and Neck (No Surgery)	OS	9	3285	3	8	0.0001	3	7	0.0054	3	7	0.0054	FALSE	No
Yang L	2019	NLR	Head and Neck (SCC)	CSS	5	1854	3	3	NA	3	4	0.2627	3	4	0.2627	NA	No
Yang L	2019	NLR	Head and Neck (SCC)	PFS	4	461	2	4	< 2.2e-16	2	2	NA	2	2	NA	FALSE	No
Cho J	2018	NLR	Head and Neck (SCC)	OS	24	5650	17	20	0.1038	17	21	0.0239	17	22	0.0027	FALSE	No
Cho J	2018	NLR	Head and Neck (SCC)	DFS	12	2079	7	0	< 2.2e-16	7	7	NA	7	11	0.0019	TRUE	None Detected
Tham T	2018	NLR	Head and Neck (SR)	OS	3	707	3	2	0.5556	3	2	0.5556	3	3	NA	TRUE	None Detected
Min GT	2018	NLR	Hepatocellular	OS	44	10491	33	20	0.0001	33	14	5.16E-09	33	36	NA	TRUE	Yes
Min GT	2018	NLR	Hepatocellular	DFS	17	3881	12	9	0.2235	12	5	0.0005	12	15	NA	TRUE	None Detected
Min GT	2018	NLR	Hepatocellular	RFS	16	5037	12	9	0.2063	12	3	1.61E-06	12	3	1.61E-06	TRUE	None Detected
Xiao WK	2014	NLR	Hepatocellular (MT)	OS	3	522	3	2	0.5556	3	3	NA	3	3	NA	TRUE	None Detected
Xiao WK	2014	NLR	Hepatocellular (RFA)	OS	2	370	2	0	2.20E-16	2	0	2.20E-16	2	0	2.20E-16	TRUE	Yes
Xiao WK	2014	NLR	Hepatocellular (RFA)	DFS	2	1054	0	0	NA	0	0	NA	0	0	NA	NA	No
Liu L	2019	NLR	Hepatocellular (Sorfenib)	OS	11	2324	10	10	NA	10	6	0.0154	10	9	0.7013	NA	No
Wang Y	2018	NLR	Hepatocellular (SR)	RFS	10	3404	7	6	0.7492	7	9	NA	7	10	NA	TRUE	None Detected
Wang Y	2018	NLR	Hepatocellular (SR)	OS	13	4225	10	7	0.1618	10	11	0.4367	10	11	0.4367	TRUE	None Detected
Wang Y	2018	NLR	Hepatocellular (SR)	DFS	7	1272	7	4	0.0226	7	5	0.2031	7	5	0.2031	TRUE	Yes
Li S	2020	NLR	Hepatocellular (TACE)	OS	16	4023	15	9	0.0017	15	14	0.7109	15	15	NA	TRUE	Yes
Xu ZG	2018	NLR	Hepatocellular (Transplant)	OS	8	1230	6	4	0.2891	6	5	0.7184	6	8	NA	TRUE	None Detected
Xu ZG	2018	NLR	Hepatocellular (Transplant)	RFS	4	83	4	4	NA	4	4	NA	4	4	NA	NA	No
Shen M	2014	IN	Hepatocellular and ICC	OS	4	846	3	4	NA	3	4	NA	3	4	NA	FALSE	No

Yang L	2019	NLR	Hypopharynx	OS	3	310	3	3	NA	3	3	NA	3	3	NA	NA	No
Jiang T	2018	NLR	Immunotherapy (Advanced Cancer)	OS	23	4355	20	22	NA	20	16	0.0733	20	21	NA	FALSE	No
Tan Q	2018	NLR	Immunotherapy (Immune checkpoint inhibitors)	OS	18	1863	15	15	NA	15	1	< 2.2e-16	15	16	NA	NA	No
Tan Q	2018	NLR	Immunotherapy (Immune checkpoint inhibitors)	PFS	11	1274	9	5	0.0289	9	1	1.97E-08	9	8	0.738	TRUE	Yes
Xie X	2019	NLR	Ipilimumuab	OS	7	798	7	6	0.6034	7	1	1.21E-06	7	7	NA	TRUE	None Detected
Xie X	2019	NLR	Ipilimumuab	PFS	3	370	2	0	< 2.2e-16	2	3	< 2.2e-16	2	3	< 2.2e-16	TRUE	No (NM)
Mu S	2018	NLR	Large B	OS	10	NA†	6	2	0.0064	6	8	NA	6	8	NA	TRUE	Yes
Mu S	2018	NLR	Large B	PFS	9	NA†	4	7	NA	4	6	NA	4	7	NA	FALSE	No
Yang L	2019	NLR	Larynx	DFS	3	1260	2	2	NA	2	2	NA	2	2	NA	NA	No
Mascarella M	2018	NLR	Larynx	OS	6	1017	4	2	0.1001	4	4	NA	4	4	NA	TRUE	None Detected
Zhao QT	2015	NLR	Lung (both)	OS	21	6942	18	17	0.7831	18	7	1.10E-06	18	16	0.443	TRUE	None Detected
Zhao QT	2015	NLR	Lung (both)	PFS	9	1748	6	7	NA	6	3	0.0684	6	5	0.7398	FALSE	No
Yu Y	2017	NLR	Lung (Surgery)	OS	7	2877	7	6	0.6034	7	3	0.0027	7	6	0.6034	TRUE	None Detected
Zhan H	2018	NLR	Melanoma	OS	10	4437	8	5	0.1094	8	2	7.79E-05	8	9	NA	TRUE	None Detected
Ding Y	2018	NLR	Melanoma	PFS	6	752	5	5	NA	5	6	NA	5	6	NA	NA	No
Xie X	2019	NLR	Melanoma (Immune checkpoint inhibitors)	OS	9	1104	9	8	0.6103	9	1	2.58E-09	9	9	NA	TRUE	None Detected
Xie X	2019	NLR	Melanoma (Immune checkpoint inhibitors)	PFS	4	467	4	3	0.5781	4	4	NA	4	4	NA	TRUE	None Detected
Chen	2017	NLR	MPM	OS	11	1378	7	0	2.20E-16	7	3	0.0126	7	6	NA	TRUE	Yes
Zeng Q	2018	NLR	Multiple Myeloma	OS	8	1886	6	8	< 2.2e-16	6	1	8.52E-05	6	7	0.2637	FALSE	No
Zeng Q	2018	NLR	Multiple Myeloma	PFS	4	1334	4	4	NA	4	0	< 2.2e-16	4	4	NA	NA	No
Yang S	2019	NLR	Nasopharyngeal	OS	10	7031	7	8	0.4296	7	9	0.0702	7	9	0.0702	FALSE	No
Yin J	2017	NLR	Nasopharyngeal	PFS	5	4130	4	4	NA	4	5	NA	4	5	NA	NA	No
Su L	2017	NLR	Nasopharyngeal	CSS	3	2320	2	2	NA	2	2	NA	2	2	NA	NA	No
Li X	2018	NLR	NAC	OS	18	3282	13	17	NA	13	3	2.84E-07	13	14	NA	FALSE	No
Li X	2018	NLR	NAC	RFS	7	775	6	7	NA	6	6	NA	6	6	NA	FALSE	No
Li X	2018	NLR	NAC	CSS	5	971	5	5	NA	5	3	0.1648	5	5	NA	NA	No
Xie X	2019	NLR	Nivolumab	OS	6	442	5	5	NA	5	5	NA	5	6	< 2.2e-16	NA	No
Xie X	2019	NLR	Nivolumab	PFS	8	744	6	4	0.2891	6	1	8.52E-05	6	4	0.2891	TRUE	None Detected
Vartolomei MD	2018	NLR	Non muscle Invasive Bladder	RFS	6	2298	5	3	0.2188	5	5	NA	5	6	NA	TRUE	None Detected
Vartolomei MD	2018	NLR	Non muscle Invasive Bladder	PFS	6	2298	4	5	NA	4	6	NA	4	6	NA	FALSE	No

Vartolomei MD	2018	NLR	Non muscle Invasive Bladder (High Risk)	RFS	4	599	3	2	0.625	3	3	NA	3	4	NA	TRUE	None Detected
Vartolomei MD	2018	NLR	Non muscle Invasive Bladder (High Risk)	PFS	3	510	2	3	NA	2	3	NA	2	3	NA	FALSE	No
Lu Y	2020	NLR	NSCLC	OS	21	5141	15	2	1.49E-11	15	10	0.0466	15	14	0.8179	TRUE	Yes
Gu XB	2015	NLR	NSCLC	PFS	10	2623	7	5	0.3438	7	1	9.12E-06	7	8	NA	TRUE	None Detected
Shen M	2014	IN	NSCLC	OS	2	967	0	1	NA	0	1	NA	0	1	NA	FALSE	No
Wang Z	2019	NLR	NSCLC (Chemotherapy)	OS	9	1276	5	7	0.1178	5	1	0.0014	5	8	0.0121	FALSE	No
Wang Z	2019	NLR	NSCLC (Chemotherapy)	PFS	5	875	4	2	0.0870	4	4	NA	4	4	NA	TRUE	Yes
Xie X	2019	NLR	NSCLC (Immune Checkpoint Inhibitors)	PFS	6	609	4	3	0.6875	4	0	< 2.2e-16	4	2	0.1001	TRUE	None Detected
Wang Z	2019	NLR	NSCLC (Immunotherapy)	OS	8	1396	7	1	3.4E-06	7	4	0.0703	7	8	< 2.2e-16	TRUE	Yes
Cao D	2018	NLR	NSCLC (Nivoumab)	OS	8	666	7	6	0.6885	7	1	3.40E-06	7	5	0.2725	TRUE	None Detected
Cao D	2018	NLR	NSCLC (Nivoumab)	PFS	10	900	8	4	0.0183	8	1	3.74E-07	8	5	0.1094	TRUE	Yes
Jiang T	2019	NLR	NSCLC (PD-1 inhibitor)	OS	13	2018	13	9	0.0133	13	12	0.6173	13	13	NA	TRUE	Yes
Jiang T	2019	NLR	NSCLC (PD-1 inhibitor)	PFS	12	1614	8	9	0.5096	8	8	NA	8	8	NA	FALSE	No
Wang Z	2019	NLR	NSCLC (ST)	OS	24	3694	19	2	< 2.2e-16	19	2	< 2.2e-16	19	20	0.5825	TRUE	None Detected
Wang Z	2019	NLR	NSCLC (ST)	PFS	16	2679	11	8	0.2101	11	1	1.85E-10	11	11	NA	TRUE	None Detected
Wang Z	2019	NLR	NSCLC (TT)	OS	5	422	5	0	< 2.2e-16	5	0	< 2.2e-16	5	4	0.5904	TRUE	No (NM)
Wang Z	2019	NLR	NSCLC (TT)	PFS	5	619	3	1	0.0579	3	0	< 2.2e-16	3	3	NA	TRUE	Yes
Zhang X	2018	NLR	Oesophageal	OS	26	8985	16	25	2.13E-08	16	18	0.3999	16	22	0.0036	FALSE	No
Zhang X	2018	NLR	Oesophageal	CSS	7	1885	3	6	0.0102	3	6	0.0102	3	5	0.1083	FALSE	No
Zhang X	2018	NLR	Oesophageal	RFS	3	677	2	2	NA	2	3	< 2.2e-16	2	3	< 2.2e-16	NA	No
Zhang X	2018	NLR	Oesophageal	DFS	10	2837	5	5	NA	5	4	0.5342	5	9	0.0016	NA	No
Zhang X	2018	NLR	Oesophageal	PFS	4	908	2	3	0.2617	2	2	NA	2	2	NA	FALSE	No
Pirozzolo G	2019	NLR	Oesophageal (DCRT)	OS	3	1563	3	3	NA	3	3	NA	3	3	NA	NA	No
Pirozzolo G	2019	NLR	Oesophageal (Neo+Surgery)	OS	3	1314	2	2	NA	2	2	NA	2	2	NA	NA	No
Zhang X	2018	NLR	Oesophageal (No Surgery)	OS	4	1046	3	4	< 2.2e-16	3	4	< 2.2e-16	3	4	< 2.2e-16	FALSE	No
Zhang X	2018	NLR	Oesophageal (Surgery)	OS	22	7119	12	20	8.90E-06	12	13	0.6703	12	17	0.0189	FALSE	No
Pirozzolo G	2019	NLR	Oesophageal (Surgery+/-Chemo)	OS	14	3580	10	9	0.7819	10	13	0.0146	10	13	0.0146	TRUE	None Detected
Zhang X	2018	NLR	Oesophageal SCC	OS	18	7403	10	18	< 2.2e-16	10	14	0.0408	10	14	0.0408	FALSE	No
Yang Y	2018	NLR	Oral cavity	OS	9	2373	5	8	0.0121	5	8	0.0121	5	8	0.0121	FALSE	No
Yang Y	2018	NLR	Oral cavity	DFS	10	1920	6	9	0.0128	6	9	0.0128	6	9	0.0128	FALSE	No
Wang Y	2018	NLR	Oral SCC	OS	6	1360	3	4	NA	3	5	NA	3	5	NA	FALSE	No
Wang Y	2018	NLR	Oral SCC	DFS	6	1161	4	5	NA	4	6	NA	4	6	NA	FALSE	No

Yang L	2019	NLR	Oropharynx	DFS	3	NA†	1	3	< 2.2e-16	1	3	< 2.2e-16	1	3	< 2.2e-16	FALSE	No
Zhou Q	2017	NLR	Ovarian	PFS	12	3884	10	5	0.0056	10	1	9.11E-10	10	10	NA	TRUE	Yes
Zhou Q	2017	NLR	Ovarian	OS	16	4910	12	5	0.0004	12	2	1.62E-08	12	14	0.1302	TRUE	Yes
Zhou Y	2018	NLR	Pancreatic	OS	43	NA†	36	13	5.59E-13	36	21	3.91E-06	36	37	NA	TRUE	Yes
Zhou Y	2018	NLR	Pancreatic	DFS	8	1236	5	1	0.0012	5	6	NA	5	6	NA	TRUE	Yes
Yang JJ	2015	NLR	Pancreatic	CSS	3	845	3	1	0.0370	3	2	0.5556	3	2	0.5556	TRUE	Yes
Mowbray N	2018	NLR	Pancreatic (SR)	OS	8	1519	7	6	0.6885	7	6	0.6885	7	6	0.6885	TRUE	None Detected
Zhou Y	2018	NLR	Pancreatic Neuroendocrine Tumour	OS	2	223	2	2	NA	2	2	NA	2	2	NA	NA	No
Zhou Y	2018	NLR	Pancreatic Neuroendocrine Tumour	RFS	2	223	2	2	NA	2	2	NA	2	2	NA	NA	No
Guo J	2018	NLR	Prostate	PFS	7	1088	5	7	< 2.2e-16	5	6	0.2635	5	6	0.2635	FALSE	No
Cao J	2016	NLR	Prostate	OS	16	15298	13	NA	NA	13	NA	NA	13	NA	NA	NA	NA
Guo J	2018	NLR	Prostate (Metastatic)	OS	8	2152	7	4	0.0703	7	7	NA	7	8	< 2.2e-16	TRUE	Yes
Cao J	2016	NLR	Prostate CR	PFS	6	1629	5	3	0.2188	5	4	0.6708	5	4	0.6708	TRUE	None Detected
Yin X	2016	NLR	Prostate CR	OS	9	2765	8	7	0.6939	8	7	0.6939	8	7	0.6939	TRUE	None Detected
Yin X	2016	NLR	Prostate Localised	OS	2	8765	1	1	NA	1	1	NA	1	2	NA	NA	No
Guan Y	2020	NLR	Prostate CR (Abitaterone)	OS	10	1626	7	2	0.0008644	7	8	0.4296	7	9	0.0702	TRUE	Yes
Guan Y	2020	NLR	Prostate CR (Abitaterone)	PFS	2	198	1	2	< 2.2e-16	1	2	< 2.2e-16	1	1	NA	FALSE	No
Guan Y	2020	NLR	Prostate CR (Enzalutamide)	OS	5	1518	5	4	0.5904	5	3	0.1648	5	4	0.5904	TRUE	None Detected
Guan Y	2020	NLR	Prostate CR (Enzalutamide)	PFS	2	427	2	2	NA	2	1	0.5	2	2	NA	NA	No
Cao J	2016	NLR	Prostate Localised	RFS	7	11745	3	NA	NA	3	NA	NA	3	NA	NA	NA	NA
Dong YW	2016	NLR	Rectal	OS	6	857	1	6	NA	1	6	NA	1	6	NA	FALSE	No
Dong YW	2016	NLR	Rectal	DFS	3	403	1	3	NA	1	3	NA	1	3	NA	FALSE	No
Dong YW	2016	NLR	Rectal	RFS	3	433	0	3	NA	0	3	NA	0	3	NA	FALSE	No
Na N	2016	NLR	Renal	PFS	8	1115	8	5	0.0289	8	1	5.96E-08	8	7	0.6073	TRUE	Yes
Luo Y	2015	NLR	Renal	OS	14	3373	13	11	0.3271	13	11	0.3271	13	12	0.7079	TRUE	None Detected
Luo Y	2015	NLR	Renal	RFS	3	700	2	3	NA	2	3	NA	2	3	NA	FALSE	No
Luo Y	2015	NLR	Renal	CSS	4	1728	1	4	NA	1	2	NA	1	3	NA	FALSE	No
Shen M	2014	IN	Renal	OS	2	206	2	2	NA	2	2	NA	2	2	NA	NA	No
Semeniuk-Wojtas A	2018	NLR	Renal (TKI)	OS	7	NA†	7	4	0.0226	7	6	0.6034	7	7	NA	TRUE	Yes
Semeniuk-Wojtas A	2018	NLR	Renal (TKI)	PFS	6	NA†	6	1	2.14E-05	6	1	2.14E-05	6	5	0.5981	TRUE	Yes
Boissier R	2017	NLR	Renal Advanced	OS	7	1304	7	6	0.6034	7	4	0.0226	7	6	0.6034	TRUE	None Detected

Boissier R	2017	NLR	Renal Advanced	PFS	3	306	3	3	NA	3	3	NA	3	3	NA	NA	No
Boissier R	2017	NLR	Renal Localised	OS	3	1168	2	0	2.20E-16	2	0	2.20E-16	2	2	NA	TRUE	Yes
Boissier R	2017	NLR	Renal Localised	RFS	6	1962	3	0	2.20E-16	3	0	2.20E-16	3	6	NA	TRUE	Yes
Li LQ	2020	NLR	STS	DFS	7	1239	3	5	0.1083	3	6	0.0102	3	6	0.0102	FALSE	No
Liu G	2018	NLR	STS	OS	13	2658	8	11	0.0377	8	3	0.0033	8	9	0.5551	FALSE	No
Li LQ	2020	NLR	STS (Synovial Sarcoma)	OS	3	NA[†]	3	3	NA	3	3	NA	3	3	NA	NA	No
Li LQ	2020	NLR	STS (Liposarcoma)	OS	2	NA[†]	2	2	NA	2	2	NA	2	2	NA	NA	No
Feng J	2020	NLR	Thyroid	DFS	7	6343	3	7	< 2.2e-16	3	4	0.4712	3	7	< 2.2e-16	FALSE	No
Luo Y	2015	NLR	Upper Urinary	RFS	2	802	2	1	0.5	2	2	NA	2	2	NA	TRUE	None Detected
Wei Y	2014	NLR	Urinary	OS	13	3391	10	3	6.06E-05	10	9	0.7659	10	11	NA	TRUE	Yes
Su S	2019	NLR	Urothelial	OS	7	2554	3	7	< 2.2e-16	3	3	NA	3	3	NA	FALSE	No
Su S	2019	NLR	Urothelial	DFS	2	400	1	2	< 2.2e-16	1	1	NA	1	1	NA	FALSE	No
Vartolomei M	2018	NLR	Urothelial	RFS	6	3563	4	0	2.20E-16	4	3	0.6875	4	5	NA	TRUE	Yes
Suh J	2019	NLR	Urothelial (Chemotherapy)	OS	5	1664	5	4	0.5904	5	4	0.5904	5	4	0.5904	TRUE	None Detected
Suh J	2019	NLR	Urothelial (Chemotherapy)	PFS	2	500	2	2	NA	2	1	0.5	2	1	0.5	NA	No
Suh J	2019	NLR	Urothelial (Nephroureterectomy)	OS	3	1103	2	3	< 2.2e-16	2	3	< 2.2e-16	2	3	< 2.2e-16	FALSE	No
Suh J	2019	NLR	Urothelial (Nephroureterectomy)	PFS	8	4411	5	1	0.0012	5	5	NA	5	7	0.06735	TRUE	Yes
Suh J	2019	NLR	Urothelial (Radical Cystectomy)	CSS	9	6974	8	4	0.0133	8	1	1.88E-07	8	8	NA	TRUE	Yes
Suh J	2019	NLR	Urothelial (Radical Cystectomy)	OS	10	7049	8	2	7.79E-05	8	1	3.74E-07	8	8	NA	TRUE	Yes
Suh J	2019	NLR	Urothelial (Radical Cystectomy)	PFS	5	5935	4	3	0.6544	4	1	0.0067	4	2	0.0870	TRUE	None Detected
Mori K	2020	NLR	UTUC (Nephroureterectomy)	CSS	12	6350	7	1	1.52E-05	7	10	0.0364	7	11	0.0019	TRUE	Yes
Mori K	2020	NLR	UTUC (Radical Cystectomy)	CSS	12	11158	11	5	0.0005	11	2	2.80E-08	11	7	0.0189	TRUE	Yes

IA) Not applicable, N=number of component studies with in review, (O) Observed number of significant studies, (E) Expected number of significant studies, (T) True, (F) False, (HR), Hazard ratio, (NLR) Neutrophil to lymphocyte ratio, (IN) tratumoural neutrophils, (PN) Peritumoural neutrophils, (SN) Stromal Neutrophils, (OS) Overall Survival, (DFS) Disease-Free Survival, (PFS) Progression-Free Survival, (RFS) Reoccurrence-Free Survival, (CSS) Cancer Specific Survival, 'EGFR) Vascular Endothelial Growth Factor Receptor, (PC) Palliative Chemotherapy, (SBR) Surgical Bowel Resection, (NAC) Neoadjuvant Chemotherapy, (STS) Soft Tissue Sarcoma, (UTR) Urothelial Transurethral Resection, (SR) Surgical esection, (GNT) Gastroenteropancreatic Neuroendocrine Tumors, (SCC) Squamous Cell Carcinoma, (ST) Systematic Therapy, (MT) Mixed Therapy, (TT) Targeted Therapy, (CLM) Colorectal Liver Metastasis, (NS) Non-surgical, (ICC) trahepatic Cholangiocarcinoma, (RFA) Radiofrequency Ablation, (TACE) Trans-Arterial Chemoembolization, (MPM) Malignant Pleural Mesothelioma, (NSCLC) Non-Small Cell Lung Cancer, (NHC), Neck and Head Cancer, (UTUC) Upper act Urothelial Carcinoma, (DCRT) Definitive Chemoradiotherapy, (CR) Castration Resistant, (TKI) Tyrosine Kinase Inhibitor.

ll-cancers are defined as a grouping of cancer diagnosis unrelated to site, stage or treatment unless otherwise specified.
eview reported incomplete data on sample size.

ID) None detected – binomial probability test yielded a p-value>0.10.
Io) – no excess significance, observed<expected.
Io (NS)) - No excess significance, no observed significant studies.

Appendix I: Grading of evidence (GRADE criteria)

Author	Year	Biomarker	Cancer site	meta-analysis metric	Significance			Sample Size		Largest Study Sig. at <0·05	Heterogeneity		Pred. Sig.	No Small Study Effects			Excess Sig.	10% Cred. Ceiling Sig.	Level of Evidence
					Sig at <0·05	Sig at 10^{-4}	Sig at 10^{-6}	>1000 Ind.	N>3		Q	I^2 > 50%		T / F	ARC-Thompso-n	Largest > Rando-m			
Suh J	2019	NLR	Bladder (UTR)	RFS	T	T	T	T	T	T	0.284	20%	T	T	0.180	F	No	T	Strong
Ni L	2020	NLR	Endometrial	OS	T	T	T	T	T	T	0.830	0%	T	T	0.261	F	No	T	Strong
Yang S	2019	NLR	Nasopharyngeal	OS	T	T	T	T	T	T	0.358	9%	T	T	0.171	F	No	T	Strong
Yin J	2017	NLR	Nasopharyngeal	PFS	T	T	T	T	T	T	0·452	0%	T	T	0·183	F	No	T	Strong
Vartolomei MD	2018	NLR	Non muscle Invasive Bladder	PFS	T	T	T	T	T	T	0·506	0%	T	T	0·149	F	No	T	Strong
Yin X	2016	NLR	Prostate CR	OS	T	T	T	T	T	T	0·380	7%	T	T	0·340	F	None Detected	T	Strong
Li LQ	2020	NLR	STS	DFS	T	T	T	T	T	T	0.590	0%	T	T	0.125	F	No	T	Strong
Jiang T	2018	NLR	Advanced Cancer (Anti-VEGFR)	OS	T	T	T	T	T	T	<0·001	88%	F	F	0·016	T	No	T	Highly
Jiang T	2018	NLR	Advanced Cancer (Immunotherapy)	PFS	T	T	T	T	T	T	<0·001	86%	F	F	0·002	F	None Detected	T	Highly
Mei Z	2017	NLR	Composite endpoints	PFS	T	T	T	T	T	T	<0·001	89%	F	F	0·001	T	No	T	Highly
Paramanathan A	2014	NLR	Composite endpoints	DFS	T	T	T	T	T	T	<0·001	97%	F	F	<0·001	F	Yes	T	Highly
Tang H	2017	NLR	Biliary Tract	OS	T	T	T	T	T	T	0·298	14%	T	T	0·154	F	Yes	T	Highly
Wu S	2018	NLR	Bladder	RFS	T	T	T	T	T	T	0·001	71%	F	F	0·005	F	Yes	T	Highly
Ethier JL	2017	NLR	Breast	OS	T	T	T	T	T	T	0·009	55%	T	F	0·001	F	None Detected	F	Highly
Chen J	2018	NLR	Breast (Metastasis)	DFS	T	T	T	T	F	T	0·976	0%	F	F	0·862	T	No	T	Highly
Duan J	2018	NLR	Breast (SR)	OS	T	T	T	T	T	T	0.008	57%	F	F	0.219	T	No	T	Highly
Guo W	2019	NLR	Breast (Triple Negative and Her2 Positive)	DFS	T	T	T	T	T	T	0.002	50%	T	F	0.015	F	No	T	Highly
Zheng DC	2016	NLR	Colorectal	OS	T	T	T	T	T	T	<0·001	66%	F	F	0·010	F	None Detected	T	Highly
Tang H	2016	NLR	CLM	OS	T	T	T	T	T	T	0·655	0%	T	F	0·246	T	No	T	Highly
Tang H	2016	NLR	CLM	RFS	T	T	T	T	T	T	0·102	45%	F	F	0·455	T	No	T	Highly
Tang H	2016	NLR	CLM (SR)	OS	T	T	T	T	T	T	0·731	0%	T	F	0·713	T	No	T	Highly
Sun J	2016	NLR	Gastric	OS	T	T	T	T	T	T	0·004	53%	T	T	0·105	F	None Detected	T	Highly

Szor D	2018	NLR	Gastric (SR)	OS	T	T	T	T	T	T	<0·001	85%	F	F	0·737	T	No	T	Highly
Ethier JL	2017	NLR	Gynecologic	OS	T	T	T	T	T	T	<0·001	71%	F	F	<0·001	F	None Detected	T	Highly
Tham T	2018	NLR	Head and Neck	OS	T	T	T	T	T	T	0.066	31%	T	F	< 0·001	F	No	T	Highly
Min GT	2018	NLR	Hepatocellular	OS	T	T	T	T	T	T	<0·001	81%	F	F	<0·001	F	Yes	T	Highly
Min GT	2018	NLR	Hepatocellular	DFS	T	T	T	T	T	T	<0·001	80%	F	F	<0·001	F	None Detected	T	Highly
Min GT	2018	NLR	Hepatocellular	RFS	T	T	T	T	T	T	<0·001	84%	F	F	0·001	F	None Detected	T	Highly
Liu L	2019	NLR	Hepatocellular (Sorfenib)	OS	T	T	T	T	T	T	0.001	68%	T	F	0.001	T	No	T	Highly
Wang Y	2018	NLR	Hepatocellular (SR)	RFS	T	T	T	T	T	T	0·236	22%	T	F	0·079	F	None Detected	T	Highly
Wang Y	2018	NLR	Hepatocellular (SR)	OS	T	T	T	T	T	T	0.137	31%	T	F	0.010	F	None Detected	T	Highly
Wang Y	2018	NLR	Hepatocellular (SR)	DFS	T	T	T	T	T	T	0.203	30%	T	F	0.052	F	Yes	T	Highly
Li S	2020	NLR	Hepatocellular (TACE)	OS	T	T	T	T	T	T	0.019	47%	T	F	0.031	F	Yes	T	Highly
Jiang T	2018	NLR	Immunotherapy (Advanced Cancer)	OS	T	T	T	T	T	T	<0·001	81%	T	F	0·009	T	No	T	Highly
Tan Q	2018	NLR	Immunotherapy (Immune checkpoint inhibitors)	OS	T	T	T	T	T	T	<0·001	87%	F	F	<0·001	F	No	T	Highly
Tan Q	2018	NLR	Immunotherapy (Immune checkpoint inhibitors)	PFS	T	T	T	T	T	T	<0·001	85%	F	F	<0·001	F	Yes	T	Highly
Zhao QT	2015	NLR	Lung (both)	OS	T	T	T	T	T	T	<0·001	80%	F	F	0·001	T	None Detected	T	Highly
Xie X	2019	NLR	Melanoma (Immune checkpoint inhibitors)	OS	T	T	T	T	T	T	<0·001	95%	F	F	0.050	F	None Detected	T	Highly
Li X	2018	NLR	NAC	OS	T	T	T	T	T	T	<0·001	77%	F	F	<0·001	T	No	T	Highly
Gu XB	2015	NLR	NSCLC	PFS	T	T	T	T	T	T	<0·001	82%	F	F	<0·001	F	None Detected	T	Highly
Jiang T	2019	NLR	NSCLC (PD-1 inhibitor)	OS	T	T	T	T	T	T	0.222	22%	T	F	0.006	F	Yes	T	Highly
Jiang T	2019	NLR	NSCLC (PD-1 inhibitor)	PFS	T	T	T	T	T	T	0.982	0%	T	F	0.742	T	No	T	Highly
Wang Z	2019	NLR	NSCLC (ST)	PFS	T	T	T	T	T	T	<0·001	78%	F	F	< 0·001	F	None Detected	T	Highly
Zhang X	2018	NLR	Oesophageal	OS	T	T	T	T	T	T	<0·001	74%	F	F	0.039	T	No	T	Highly
Pirozzolo G	2019	NLR	Oesophageal (DCRT)	OS	T	T	T	T	F	T	0.562	0%	F	T	0.513	F	No	T	Highly
Zhang X	2018	NLR	Oesophageal (Surgery)	OS	T	T	T	T	T	T	<0·001	76%	F	F	0.076	T	No	T	Highly

Pirozzolo G	2019	NLR	Oesophageal (Surgery+/-Chemo)	OS	T	T	T	T	T	T	<0·001	69%	F	T	0.225	F	None Detected	T	Highly
Zhang X	2018	NLR	Oesophageal SCC	OS	T	T	T	T	T	T	<0·001	68%	F	F	0.331	T	No	T	Highly
Yang Y	2018	NLR	Oral cavity	OS	T	T	T	T	T	T	0.112	38%	T	F	0.747	T	No	T	Highly
Yang Y	2018	NLR	Oral cavity	DFS	T	T	T	T	T	T	0.484	0%	T	F	0.045	T	No	T	Highly
Zhou Y	2018	NLR	Pancreatic	OS	T	T	T	T	T	T	<0·001	82%	F	F	<0·001	F	Yes	T	Highly
Mowbray N	2018	NLR	Pancreatic (SR)	OS	T	T	T	T	T	T	0·180	31%	T	F	0·054	F	None Detected	T	Highly
Guo J	2018	NLR	Prostate	PFS	T	T	T	T	T	T	0.222	27%	T	F	0.178	T	No	T	Highly
Guo J	2018	NLR	Prostate (Metastatic)	OS	T	T	T	T	T	T	0.099	40%	T	F	0.023	F	Yes	T	Highly
Guan Y	2020	NLR	Prostate CR (Enzalutamide)	OS	T	T	T	T	T	T	0.037	61%	F	T	0.110	F	None Detected	T	Highly
Luo Y	2015	NLR	Renal	OS	T	T	T	T	T	T	0·104	35%	T	F	0·017	F	None Detected	T	Highly
Semeniuk-Wojtas A	2018	NLR	Renal (TKI)	OS	T	T	T	T	T	T	0·113	42%	T	T	0·385	F	Yes	T	Highly
Boissier R	2017	NLR	Renal Advanced	OS	T	T	T	T	T	T	0·004	69%	F	F	0·009	T	None Detected	T	Highly
Liu G	2018	NLR	STS	OS	T	T	T	T	T	T	<0·001	76%	F	F	0.018	T	No	T	Highly
Suh J	2019	NLR	Urothelial (Chemotherapy)	OS	T	T	T	T	T	T	0.224	30%	T	F	0.131	T	None Detected	T	Highly
Mei Z	2017	NLR	Composite endpoints	OS	T	T	T	T	T	F	<0·001	90%	T	F	<0·001	F	Yes	T	Suggestive
Shen M	2014	IN	Composite endpoints	OS	T	T	T	T	T	F	<0·001	70%	F	F	0·033	F	Yes	T	Suggestive
Dolan R	2017	NLR	Composite endpoints Operable	OS	T	T	T	T	T	F	<0·001	98%	F	F	<0·001	F	Yes	T	Suggestive
Dolan R	2017	NLR	Composite endpoints Operable	CSS	T	T	T	T	T	F	<0·001	80%	F	F	<0·001	F	Yes	T	Suggestive
Hu G	2019	NLR	Bladder	OS	T	T	F	T	T	T	<0·001	78%	F	F	< 0·001	F	Yes	T	Suggestive
Suh J	2019	NLR	Bladder (UTR)	PFS	T	T	F	T	T	T	0.635	0%	F	T	0.717	F	No	T	Suggestive
Li X	2017	NLR	Bladder and Upper Urinary	PFS	T	T	T	T	T	F	0·018	45%	T	F	<0·001	F	Yes	T	Suggestive
Li X	2017	NLR	Bladder and Upper Urinary	CSS	T	T	T	T	T	F	<0·001	66%	F	F	0·001	F	Yes	T	Suggestive
Liu X	2017	NLR	Breast	DFS	T	T	F	T	T	T	<0·001	76%	F	F	<0·001	F	No	T	Suggestive
Duan J	2018	NLR	Breast (SR)	DFS	T	T	F	T	T	T	<0·001	85%	F	F	0.046	T	No	T	Suggestive
Guo W	2019	NLR	Breast (Triple Negative and Her2 Positive)	OS	T	T	T	T	T	F	<0·001	72%	F	F	0.005	F	None Detected	T	Suggestive
Wu J	2017	NLR	Cervical	OS	T	T	F	T	T	T	0·006	58%	F	F	0·016	F	None Detected	T	Suggestive
Wu J	2017	NLR	Cervical	PFS	T	T	F	T	T	T	<0·001	90%	F	T	0·192	F	Yes	T	Suggestive
Zhang J	2017	NLR	Colorectal	DFS	T	T	F	T	T	F	0·229	29%	F	T	0·253	F	No	T	Suggestive

Zheng DC	2016	NLR	Colorectal	PFS	T	T	T	T	T	F	0·009	54%	F	F	0·041	T	No	T	Suggestive
Li H	2019	NLR	Colorectal (SR)	OS	T	T	T	T	T	F	0.015	54%	F	F	0.034	F	No	T	Suggestive
Tang H	2016	NLR	CLM (Non-surgical)	OS	T	T	F	F	F	T	0·710	0%	F	T	NA	F	No	F	Suggestive
Ni L	2020	NLR	Endometrial	PFS	T	T	F	T	T	T	0.203	33%	F	T	0.284	F	No	T	Suggestive
Lei Y	2019	NLR	Glioma	OS	T	T	T	T	T	F	<0·001	83%	F	F	< 0·001	F	No	F	Suggestive
Tham T	2018	NLR	Head and neck	DFS	T	T	F	T	T	F	0.114	36%	F	F	0.032	F	No (NM)	T	Suggestive
Tham T	2018	NLR	Head and Neck (No Surgery)	OS	T	T	F	T	T	T	0.238	23%	F	F	0.052	T	No	T	Suggestive
Yang L	2019	NLR	Head and Neck (SCC)	CSS	T	T	F	T	T	T	0.110	47%	T	T	0.124	F	No	T	Suggestive
Cho J	2018	NLR	Head and Neck (SCC)	OS	T	T	T	T	T	F	0.005	48%	T	F	0.001	F	No	T	Suggestive
Cho J	2018	NLR	Head and Neck (SCC)	DFS	T	T	F	T	T	F	<0·001	69%	F	F	0.003	F	None Detected	T	Suggestive
Xu ZG	2018	NLR	Hepatocellular (Transplant)	OS	T	T	F	T	T	F	<0·001	86%	F	F	0·008	F	None Detected	F	Suggestive
Yang L	2019	NLR	Larynx	DFS	T	T	F	T	F	T	0.399	0%	F	T	0.415	F	No	T	Suggestive
Mascarella M	2018	NLR	Larynx	OS	T	T	F	T	T	F	0·364	8%	T	T	0·268	F	None Detected	T	Suggestive
Yu Y	2017	NLR	Lung (Surgery)	OS	T	T	F	T	T	T	<0·001	83%	F	F	0·017	T	None Detected	T	Suggestive
Zhan H	2018	NLR	Melanoma	OS	T	T	F	T	T	T	<0·001	91%	F	F	0·013	F	None Detected	T	Suggestive
Zeng Q	2018	NLR	Multiple Myeloma	OS	T	T	F	T	T	T	<0·001	81%	F	F	0.286	T	No	F	Suggestive
Su L	2017	NLR	Nasopharyngeal	CSS	T	T	F	T	F	T	0·480	0%	F	T	0·487	F	No	T	Suggestive
Vartolomei MD	2018	NLR	Non muscle Invasive Bladder	RFS	T	T	F	T	T	T	0·015	64%	F	T	0·282	F	None Detected	T	Suggestive
Lu Y	2020	NLR	NSCLC	OS	T	T	T	T	T	F	<0·001	64%	F	F	0.002	F	Yes	T	Suggestive
Wang Z	2019	NLR	NSCLC (Chemotherapy)	OS	T	T	F	T	T	T	<0·001	85%	F	F	0.049	F	No	F	Suggestive
Wang Z	2019	NLR	NSCLC (Immunotherapy)	OS	T	T	T	T	T	F	<0·001	80%	F	F	0.001	F	Yes	T	Suggestive
Wang Z	2019	NLR	NSCLC (ST)	OS	T	T	T	T	T	F	<0·001	83%	F	F	< 0·001	F	None Detected	T	Suggestive
Zhang X	2018	NLR	Oesophageal (No Surgery)	OS	T	T	F	T	T	T	0.080	56%	F	F	0.549	T	No	F	Suggestive
Wang Y	2018	NLR	Oral SCC	OS	T	T	F	T	T	T	0·496	0%	T	T	0·327	F	No	T	Suggestive
Zhou Q	2017	NLR	Ovarian	PFS	T	T	F	T	T	T	<0·001	85%	F	F	0·004	F	Yes	T	Suggestive
Zhou Q	2017	NLR	Ovarian	OS	T	T	F	T	T	F	<0·001	80%	F	F	< 0·001	F	Yes	T	Suggestive
Cao J	2016	NLR	Prostate	OS	T	T	T	T	T	F	<0·001	69%	T	F	0.002	F	NA	T	Suggestive
Cao J	2016	NLR	Prostate CR	PFS	T	T	F	T	T	T	0·090	47%	F	T	0·163	F	None Detected	T	Suggestive

Guan Y	2020	NLR	Prostate CR (Abitaterone)	OS	T	T	T	T	T	F	0.053	46%	T	F	0.069	F	Yes	T	Suggestive
Dong YW	2016	NLR	Rectal	OS	T	T	F	F	T	T	0·879	0%	T	F	0·298	T	No	T	Suggestive
Na N	2016	NLR	Renal	PFS	T	T	F	T	T	T	<0·001	88%	F	F	0·046	F	Yes	T	Suggestive
Wei Y	2014	NLR	Urinary	OS	T	T	T	T	T	F	0·005	58%	F	F	<0·001	F	Yes	T	Suggestive
Suh J	2019	NLR	Urothelial (Nephroureterectomy)	OS	T	T	F	T	F	T	0.463	0%	F	T	0.544	F	No	T	Suggestive
Suh J	2019	NLR	Urothelial (Nephroureterectomy)	PFS	T	T	F	T	T	F	0.013	61%	F	F	0.011	F	Yes	F	Suggestive
Suh J	2019	NLR	Urothelial (Radical Cystectomy)	CSS	T	T	F	T	T	T	<0·001	88%	F	F	0.001	F	Yes	T	Suggestive
Mori K	2020	NLR	UTUC (Nephroureterectomy)	CSS	T	T	T	T	T	F	0.022	51%	F	T	0.104	F	Yes	T	Suggestive
Mori K	2020	NLR	UTUC (Radical Cystectomy)	CSS	T	T	F	T	T	T	<0·001	78%	F	F	< 0·001	F	Yes	T	Suggestive
Shen M	2014	IN	Composite endpoints	CSS	T	T	T	F	F	T	0·8243	0%	F	T	NA	F	No	F	Weak
Tang H	2017	NLR	Biliary Tract	RFS	T	T	F	F	T	F	0·144	42%	F	F	0·093	F	Yes	T	Weak
Wu S	2018	NLR	Bladder (Metastasis)	OS	T	T	T	F	T	T	0·922	0%	T	F	0·976	T	No	T	Weak
Hu G	2019	NLR	Bladder (Radical Cystectomy)	OS	T	F	F	T	T	T	0.001	73%	F	F	0.003	F	Yes	T	Weak
Liu X	2017	NLR	Breast	CSS	T	F	F	T	T	F	0·004	77%	F	F	0·071	F	None Detected	F	Weak
Chen J	2018	NLR	Breast (Metastasis)	OS	T	T	T	F	F	T	0·923	0%	F	T	NA	F	No	F	Weak
Duan J	2018	NLR	Breast (SR)	RFS	T	T	F	F	F	T	0.839	0%	F	F	NA	T	No	F	Weak
Liu X	2017	NLR	Breast (Triple-negative)	OS	T	T	F	F	T	T	0·479	0%	F	F	0·262	T	No	T	Weak
Liu X	2017	NLR	Breast (Triple-negative)	DFS	T	T	F	F	T	T	0·609	0%	F	T	0·357	F	No	T	Weak
Malietzis G	2014	NLR	Colorectal (PC)	DFS	T	T	F	F	F	T	0·834	0%	F	T	0·686	F	No	F	Weak
Li H	2019	NLR	Colorectal (SR)	DFS	T	F	F	T	T	F	0.032	52%	F	F	0.027	F	No	F	Weak
Li H	2019	NLR	Colorectal (SR)	RFS	T	T	T	F	F	T	0.424	0%	F	T	0.638	F	No	T	Weak
Tang H	2016	NLR	CLM (Non-surgical)	RFS	T	F	F	F	F	F	0·232	30%	F	T	NA	F	No	F	Weak
Tang H	2016	NLR	CLM (SR)	RFS	T	T	F	F	T	T	0·063	59%	F	F	0·576	T	Yes	F F	Weak
Sun J	2016	NLR	Gastric	DFS	T	T	T	F	T	T	0·780	0%	T	F	0·887	T	No	T	Weak
Zhang X	2014	NLR	Gastric	PFS	T	F	F	F	F	T	0·275	16%	F	T	NA	F	None Detected	F	Weak
Mellor K	2018	NLR	Gastric (SR)	DFS	T	F	F	F	F	T	0·007	80%	F	F	0·197	T	No	T	Weak
Zhou Y	2018	NLR	GNT	OS	T	T	T	F	T	T	0·571	0%	T	F	0·299	T	No	T	Weak
Zhou Y	2018	NLR	GNT	RFS	T	T	T	F	F	T	0·484	0%	F	T	0·466	F	No	T	Weak

Yu Y	2018	NLR	Head and Neck	RFS	T	F	F	F	T	T	0·023	69%	F	F	0·182	T	No	F	Weak
Tham T	2018	NLR	Head and neck	PFS	T	F	F	T	T	T	0.053	54%	F	F	0.276	T	No	F	Weak
Shen M	2014	IN	Head and Neck	OS	T	F	F	F	F	F	0·391	0%	F	F	0·419	T	No	F	Weak
Yang L	2019	NLR	Head and Neck (SCC)	PFS	T	F	F	F	T	T	0.104	51%	F	F	0.177	T	No	F	Weak
Tham T	2018	NLR	Head and Neck (SR)	OS	T	F	F	F	F	T	0.087	59%	F	T	0.272	F	None Detected	F	Weak
Xiao WK	2014	NLR	Hepatocellular (MT)	OS	T	T	F	F	F	T	0·374	0%	F	T	0·394	F	None Detected	T	Weak
Xiao WK	2014	NLR	Hepatocellular (RFA)	OS	T	T	F	F	F	T	0·333	0%	F	F	NA	T	Yes	F	Weak
Xu ZG	2018	NLR	Hepatocellular (Transplant)	RFS	T	T	F	F	T	T	0·029	67%	F	T	0·262	F	No	T	Weak
Shen M	2014	IN	Hepatocellular and ICC	OS	T	T	F	F	T	T	0·071	57%	F	F	0·742	T	No	F	Weak
Yang L	2019	NLR	Hypopharynx	OS	T	T	T	F	F	T	0.622	0%	F	F	0.589	T	No	T	Weak
Xie X	2019	NLR	Ipilimumuab	OS	T	T	F	F	T	T	<0·001	96%	F	F	0.046	F	None Detected	T	Weak
Xie X	2019	NLR	Ipilimumuab	PFS	T	F	F	F	F	F	0.036	70%	F	T	0.461	F	No (NM)	F	Weak
Mu S	2018	NLR	Large B	OS	T	F	F	T	T	F	<0·001	71%	F	F	0·131	F	Yes	T	Weak
Mu S	2018	NLR	Large B	PFS	T	F	F	T	T	T	0·001	69%	F	T	T	F	No	T	Weak
Zhao QT	2015	NLR	Lung (both)	PFS	T	F	F	T	T	T	<0·001	80%	F	F	0·205	T	No	T	Weak
Ding Y	2018	NLR	Melanoma	PFS	T	T	T	F	T	F	0·558	0%	T	T	0·241	F	No	T	Weak
Xie X	2019	NLR	Melanoma (Immune checkpoint inhibitors)	PFS	T	T	T	F	T	T	0.199	35%	T	T	0.716	F	None Detected	T	Weak
Chen	2017	NLR	MPM	OS	T	F	F	T	T	F	<0·001	77%	F	T	0·719	F	Yes	F	Weak
Zeng Q	2018	NLR	Multiple Myeloma	PFS	T	F	F	T	T	T	<0·001	88%	F	F	0.039	T	No	F	Weak
Li X	2018	NLR	NAC	RFS	T	T	F	F	T	T	0·151	36%	F	F	0·104	T	No	T	Weak
Li X	2018	NLR	NAC	CSS	T	F	F	F	T	T	<0·001	81%	F	F	0·126	T	No	T	Weak
Xie X	2019	NLR	Nivolumab	OS	T	T	T	F	T	T	0.158	37%	T	T	0.345	F	No	T	Weak
Xie X	2019	NLR	Nivolumab	PFS	T	T	F	F	T	T	<0·001	87%	F	F	0.068	F	None Detected	F	Weak
Vartolomei MD	2018	NLR	Non muscle Invasive Bladder (High Risk)	RFS	T	F	F	F	T	T	0·068	58%	F	T	0·163	F	None Detected	T	Weak
Vartolomei MD	2018	NLR	Non muscle Invasive Bladder (High Risk)	PFS	T	F	F	F	F	T	0·413	0%	F	F	0·751	T	No	F	Weak
Wang Z	2019	NLR	NSCLC (Chemotherapy)	PFS	T	T	T	F	T	F	0.245	26%	F	T	0.146	F	Yes	T	Weak

Xie X	2019	NLR	NSCLC (Immune Checkpoint Inhibitors)	PFS	T	F	F	F	T	T	0.003	72%	F	F	0.018	T	None Detected	F	Weak
Cao D	2018	NLR	NSCLC (Nivoumab)	OS	T	T	F	F	T	T	<0·001	87%	F	F	0.015	T	None Detected	T	Weak
Cao D	2018	NLR	NSCLC (Nivoumab)	PFS	T	F	F	F	T	T	<0·001	86%	F	F	0.008	F	Yes	F	Weak
Wang Z	2019	NLR	NSCLC (TT)	OS	T	F	F	F	T	T	0.001	77%	F	F	0.025	F	No (NM)	F	Weak
Wang Z	2019	NLR	NSCLC (TT)	PFS	T	F	F	F	T	F	0.003	75%	F	F	0.034	F	Yes	F	Weak
Zhang X	2018	NLR	Oesophageal	CSS	T	F	F	T	T	T	0.025	59%	F	F	0.877	T	No	F	Weak
Zhang X	2018	NLR	Oesophageal	RFS	T	F	F	F	F	T	0.187	40%	F	T	0.675	F	No	F	Weak
Zhang X	2018	NLR	Oesophageal	DFS	T	F	F	T	T	F	<0·001	86%	F	F	0.049	F	No	F	Weak
Zhang X	2018	NLR	Oesophageal	PFS	T	T	F	F	T	T	0.484	0%	F	F	0.568	T	No	T	Weak
Pirozzolo G	2019	NLR	Oesophageal (Neo+Surgery)	OS	T	F	F	T	F	T	0.493	0%	F	T	0.451	F	No	T	Weak
Wang Y	2018	NLR	Oral SCC	DFS	T	F	F	T	T	T	0·629	0%	T	T	0·203	F	No	T	Weak
Yang L	2019	NLR	Oropharynx	DFS	T	F	F	NA	F	T	0.822	0%	F	F	0.670	T	No	T	Weak
Zhou Y	2018	NLR	Pancreatic	DFS	T	F	F	T	T	F	0·003	67%	F	T	0·370	F	Yes	F	Weak
Yang JJ	2015	NLR	Pancreatic	CSS	T	F	F	F	F	T	0·012	78%	F	T	0·424	F	Yes	F	Weak
Zhou Y	2018	NLR	Pancreatic Neuroendocrine Tumour	OS	T	T	F	F	F	T	0·623	0%	F	T	NA	F	No	F	Weak
Zhou Y	2018	NLR	Pancreatic Neuroendocrine Tumour	RFS	T	T	F	F	F	T	0·766	0%	F	T	NA	F	No	F	Weak
Dong YW	2016	NLR	Rectal	DFS	T	T	F	F	F	T	0·635	0%	F	T	0·538	F	No	T	Weak
Dong YW	2016	NLR	Rectal	RFS	T	F	F		F	T	0·684	0%	F	T	0·545	F	No	T	Weak
Luo Y	2015	NLR	Renal	RFS	T	T	F	F	F	T	0·364	1%	F	F	0·392	T	No	T	Weak
Shen M	2014	IN	Renal	OS	T	T	T	F	F	T	0·411	0%	F	F	NA	T	No	F	Weak
Semeniuk-Wojtas A	2018	NLR	Renal (TKI)	PFS	T	F	F	T	T	T	<0·001	90%	F	T	0·120	F	Yes	F	Weak
Boissier R	2017	NLR	Renal Advanced	PFS	T	T	T	F	F	T	0·779	0%	F	F	0·981	T	No	T	Weak
Boissier R	2017	NLR	Renal Localised	RFS	T	F	F	T	T	F	<0·001	82%	F	F	0·007	F	Yes	F	Weak
Li LQ	2020	NLR	STS (Synovial Sarcoma)	OS	T	T	T	NA	F	T	0.510	0%	F	F	0.464	T	No	T	Weak
Li LQ	2020	NLR	STS (Liposarcoma)	OS	T	T	F	NA	F	T	0.952	0%	F	T	NA	F	No	F	Weak
Luo Y	2015	NLR	Upper Urinary	RFS	T	F	F	F	F	T	0·287	12%	F	T	NA	F	None Detected	F	Weak
Su S	2019	NLR	Urothelial	OS	T	F	F	T	T	F	0.042	54%	F	F	0.844	T	No	T	Weak
Vartolomei M	2018	NLR	Urothelial	RFS	T	F	F	T	T	F	0·015	65%	F	F	0·021	F	Yes	F	Weak

Suh J	2019	NLR	Urothelial (Chemotherapy)	PFS	T	F	F	F	F	T	0.097	64%	F	F	NA	T	No	F	Weak
Suh J	2019	NLR	Urothelial (Radical Cystectomy)	OS	T	F	F	T	T	T	<0·001	84%	F	F	< 0·001	F	Yes	F	Weak
Suh J	2019	NLR	Urothelial (Radical Cystectomy)	PFS	T	F	F	T	T	T	<0·001	82%	F	F	0.027	T	None Detected	F	Weak
Cao J	2016	NLR	Prostate Localised	RFS	T	F	F	T	T	F	<0·001	90%	F	T	0.351	F	NA	F	Weak
Shen M	2014	PN	Composite endpoints	OS	F	F	F	F	F	F	0·005	87%	F	T	NA	F	Yes	F	Uncertain
Shen M	2014	SN	Composite endpoints	OS	F	F	F	F	F	F	0·129	57%	F	T	NA	F	No (NM)	F	Uncertain
Hu G	2019	NLR	Bladder (Radical Cystectomy and NAC)	OS	F	F	F	F	T	F	0.296	19%	F	T	0.475	F	No (NM)	F	Uncertain
Chen J	2018	NLR	Breast (No Metastasis)	OS	F	F	F	F	F	F	<0·001	92%	F	T	NA	F	No	F	Uncertain
Chen J	2018	NLR	Breast (No Metastasis)	DFS	F	F	F	F	F	T	0·439	0%	F	T	NA	F	No	F	Uncertain
Shen M	2014	IN	Gastric	OS	F	F	F	F	F	T	0·011	84%	F	F	NA	T	No	F	Uncertain
Luo XF	2018	NLR	Gastrointestinal Stromal	OS	F	F	F	F	F	T	0·014	83%	F	T	NA	F	Yes	F	Uncertain
Xiao WK	2014	NLR	Hepatocellular (RFA)	DFS	F	F	F	T	F	F	0·333	0%	F	T	NA	T	No	F	Uncertain
Shen M	2014	IN	NSCLC	OS	F	F	F	F	F	F	0·960	0%	F	F	NA	T	No	F	Uncertain
Yin X	2016	NLR	Prostate Localised	OS	F	F	F	T	F	T	0·039	76%	F	T	NA	F	No	F	Uncertain
Guan Y	2020	NLR	Prostate CR (Abitaterone)	PFS	F	F	F	F	F	T	0.060	72%	F	F	NA	T	No	F	Uncertain
Guan Y	2020	NLR	Prostate CR (Enzalutamide)	PFS	F	F	F	F	F	T	0.002	90%	F	F	NA	T	No	F	Uncertain
Luo Y	2015	NLR	Renal	CSS	F	F	F	T	T	T	<0·001	86%	F	F	0·534	T	No	F	Uncertain
Boissier R	2017	NLR	Renal Localised	OS	F	F	F	T	F	T	0·020	74%	F	T	0·219	F	Yes	F	Uncertain
Feng J	2020	NLR	Thyroid	DFS	F	F	F	T	T	T	0.007	66%	F	F	0.207	T	No	F	Uncertain
Su S	2019	NLR	Urothelial	DFS	F	F	F	F	F	T	0.119	59%	F	F	NA	T	No	F	Uncertain

A) Not applicable, (T) True, (F) False, (HR), Hazard ratio, (NLR) Neutrophil to lymphocyte ratio, (IN) Intratumoural neutrophils, (PN) Peritumoural neutrophils, (SN) Stromal Neutrophils, (OS) Overall Survival, (DFS) Disease-Free Survival, FS) Progression-Free Survival, (RFS) Reoccurrence-Free Survival, (CSS) Cancer Specific Survival, (VEGFR) Vascular Endothelial Growth Factor Receptor, (PC) Palliative Chemotherapy, (SBR) Surgical Bowel Resection, (NAC) Neoadjuvant hemotherapy, (STS) Soft Tissue Sarcoma, (UTR) Urothelial Transurethral Resection, (SR) Surgical Resection, (GNT) Gastroenteropancreatic Neuroendocrine Tumors, (SCC) Squamous Cell Carcinoma, (ST) Systematic Therapy, (MT) Mixed herapy, (TT) Targeted Therapy, (CLM) Colorectal Liver Metastasis, (NS) Non-surgical, (ICC) Intrahepatic Cholangiocarcinoma, (RFA) Radiofrequency Ablation, (TACE) Trans-Arterial Chemoembolization, (MPM) Malignant Pleural esothelioma, (NSCLC) Non-Small Cell Lung Cancer, (NHC), Neck and Head Cancer, (UTUC) Upper Tract Urothelial Carcinoma, (DCRT) Definitive Chemoradiotherapy, (CR) Castration Resistant, (TKI) Tyrosine Kinase Inhibitor.

ll-cancers are defined as a grouping of cancer diagnosis unrelated to site, stage or treatment unless otherwise specified.
eview reported incomplete data on sample size.

D) None detected – binomial probability test yielded a p-value>0·10.
o) – no excess significance, observed<expected.
o (NS)) - No excess significance, no observed significant studies.

Meets criteria	Doesn’t meet criteria

Appendix J: Effect size upgrading of GRADE

Author	Year	Biomarker	Cancer site	meta-analysis metric	Significance			Sample Size		HR	Lower CI	Upper CI	Level of Evidence	Up-GRADE
					Sig at <0.05	Sig at 10^{-4}	Sig at 10^{-6}	>1000 Ind.	>3 Studies					
Jiang T	2018	NLR	Advanced Cancer (Anti-VEGFR)	OS	T	T	T	T	T	2.02	1.61	2.53	Highly	Strong
Paramanathan A	2014	NLR	Composite endpoints	DFS	T	T	T	T	T	2.11	1.71	2.6	Highly	Strong
Suh J	2019	NLR	Bladder (UTR)	RFS	T	T	T	T	T	2.22	1.81	2.74	Strong	Strong
Ethier JL	2017	NLR	Breast	OS	T	T	T	T	T	2.54	1.96	3.30	Highly	Strong
Duan J	2018	NLR	Breast (SR)	OS	T	T	T	T	T	2.47	1.71	3.56	Highly	Strong
Tang H	2016	NLR	CLM	OS	T	T	T	T	T	2.17	1.83	2.57	Highly	Strong
Tang H	2016	NLR	CLM (SR)	OS	T	T	T	T	T	2.08	1.73	2.49	Highly	Strong
Ni L	2020	NLR	Endometrial	OS	T	T	T	T	T	2.22	1.76	2.79	Strong	Strong
Szor D	2018	NLR	Gastric (SR)	OS	T	T	T	T	T	3.13	1.99	4.92	Highly	Strong
Tan Q	2018	NLR	Immunotherapy (Immune checkpoint inhibitors)	OS	T	T	T	T	T	2.21	1.70	2.88	Highly	Strong
Xie X	2019	NLR	Melanoma (Immune checkpoint inhibitors)	OS	T	T	T	T	T	2.49	1.72	3.61	Highly	Strong
Yang S	2019	NLR	Nasopharyngeal	OS	T	T	T	T	T	1.48	1.29	1.69	Strong	Strong
Yin J	2017	NLR	Nasopharyngeal	PFS	T	T	T	T	T	1.50	1.30	1.73	Strong	Strong
Vartolomei MD	2018	NLR	Non muscle Invasive Bladder	PFS	T	T	T	T	T	2.26	1.59	3.22	Strong	Strong
Jiang T	2019	NLR	NSCLC (PD-1 inhibitor)	OS	T	T	T	T	T	2.59	2.1	3.2	Highly	Strong
Yin X	2016	NLR	Prostate CR	OS	T	T	T	T	T	1.56	1.42	1.72	Strong	Strong
Semeniuk-Wojtas A	2018	NLR	Renal (TKI)	OS	T	T	T	T	T	2.14	1.66	2.76	Highly	Strong
Li LQ	2020	NLR	STS	DFS	T	T	T	T	T	1.72	1.43	2.08	Strong	Strong
Jiang T	2018	NLR	Advanced Cancer (Immunotherapy)	PFS	T	T	T	T	T	1.78	1.47	2.15	Highly	Highly
Mei Z	2017	NLR	Composite endpoints	PFS	T	T	T	T	T	1.63	1.43	1.86	Highly	Highly
Tang H	2017	NLR	Biliary Tract	OS	T	T	T	T	T	1.52	1.36	1.70	Highly	Highly
Wu S	2018	NLR	Bladder	RFS	T	T	T	T	T	1.52	1.29	1.79	Highly	Highly
Chen J	2018	NLR	Breast (Metastasis)	DFS	T	T	T	T	F	2.15	1.53	3.01	Highly	Highly
Guo W	2019	NLR	Breast (Triple Negative and Her2 Positive)	DFS	T	T	T	T	T	1.59	1.41	1.8	Highly	Highly

Zheng DC	2016	NLR	Colorectal	OS	T	T	T	T	T	1.92	1.58	2.32	Highly	Highly
Tang H	2016	NLR	CLM	RFS	T	T	T	T	T	1.97	1.48	2.62	Highly	Highly
Sun J	2016	NLR	Gastric	OS	T	T	T	T	T	1.98	1.75	2.23	Highly	Highly
Ethier JL	2017	NLR	Gynecologic	OS	T	T	T	T	T	1.72	1.46	2.03	Highly	Highly
Tham T	2018	NLR	Head and Neck	OS	T	T	T	T	T	1.72	1.51	1.96	Highly	Highly
Min GT	2018	NLR	Hepatocellular	OS	T	T	T	T	T	1.64	1.41	1.90	Highly	Highly
Min GT	2018	NLR	Hepatocellular	DFS	T	T	T	T	T	1.82	1.40	2.37	Highly	Highly
Min GT	2018	NLR	Hepatocellular	RFS	T	T	T	T	T	1.85	1.48	2.32	Highly	Highly
Liu L	2019	NLR	Hepatocellular (Sorfenib)	OS	T	T	T	T	T	1.73	1.45	2.07	Highly	Highly
Wang Y	2018	NLR	Hepatocellular (SR)	RFS	T	T	T	T	T	1.71	1.45	2.03	Highly	Highly
Wang Y	2018	NLR	Hepatocellular (SR)	OS	T	T	T	T	T	1.6	1.39	1.84	Highly	Highly
Wang Y	2018	NLR	Hepatocellular (SR)	DFS	T	T	T	T	T	1.5	1.35	1.67	Highly	Highly
Li S	2020	NLR	Hepatocellular (TACE)	OS	T	T	T	T	T	1.85	1.64	2.08	Highly	Highly
Jiang T	2018	NLR	Immunotherapy (Advanced Cancer)	OS	T	T	T	T	T	1.95	1.67	2.29	Highly	Highly
Tan Q	2018	NLR	Immunotherapy (Immune checkpoint inhibitors)	PFS	T	T	T	T	T	1.75	1.39	2.21	Highly	Highly
Zhao QT	2015	NLR	Lung (both)	OS	T	T	T	T	T	1.55	1.33	1.80	Highly	Highly
Li X	2018	NLR	NAC	OS	T	T	T	T	T	1.66	1.36	2.04	Highly	Highly
Gu XB	2015	NLR	NSCLC	PFS	T	T	T	T	T	1.59	1.29	1.95	Highly	Highly
Jiang T	2019	NLR	NSCLC (PD-1 inhibitor)	PFS	T	T	T	T	T	1.63	1.42	1.87	Highly	Highly
Wang Z	2019	NLR	NSCLC (ST)	PFS	T	T	T	T	T	1.5	1.29	1.74	Highly	Highly
Zhang X	2018	NLR	Oesophageal	OS	T	T	T	T	T	1.5	1.32	1.71	Highly	Highly
Pirozzolo G	2019	NLR	Oesophageal (DCRT)	OS	T	T	T	T	F	1.74	1.55	1.95	Highly	Highly
Zhang X	2018	NLR	Oesophageal (Surgery)	OS	T	T	T	T	T	1.48	1.27	1.73	Highly	Highly
Pirozzolo G	2019	NLR	Oesophageal (Surgery+/-Chemo)	OS	T	T	T	T	T	1.77	1.43	2.2	Highly	Highly
Zhang X	2018	NLR	Oesophageal SCC	OS	T	T	T	T	T	1.39	1.22	1.58	Highly	Highly
Yang Y	2018	NLR	Oral cavity	OS	T	T	T	T	T	1.71	1.42	2.06	Highly	Highly
Yang Y	2018	NLR	Oral cavity	DFS	T	T	T	T	T	1.72	1.44	2.07	Highly	Highly
Zhou Y	2018	NLR	Pancreatic	OS	T	T	T	T	T	1.81	1.59	2.05	Highly	Highly
Mowbray N	2018	NLR	Pancreatic (SR)	OS	T	T	T	T	T	1.72	1.48	2.00	Highly	Highly

Guo J	2018	NLR	Prostate	PFS	T	T	T	T	T	1.97	1.49	2.62	Highly	Highly
Guo J	2018	NLR	Prostate (Metastatic)	OS	T	T	T	T	T	1.67	1.38	2.02	Highly	Highly
Guan Y	2020	NLR	Prostate CR (Enzalutamide)	OS	T	T	T	T	T	1.48	1.26	1.76	Highly	Highly
Luo Y	2015	NLR	Renal	OS	T	T	T	T	T	1.92	1.63	2.27	Highly	Highly
Boissier R	2017	NLR	Renal Advanced	OS	T	T	T	T	T	1.91	1.44	2.53	Highly	Highly
Liu G	2018	NLR	STS	OS	T	T	T	T	T	1.56	1.3	1.88	Highly	Highly
Suh J	2019	NLR	Urothelial (Chemotherapy)	OS	T	T	T	T	T	1.45	1.27	1.65	Highly	Highly

(NA) Not applicable, (T) True, (F) False, (HR), Hazard ratio, (NLR) Neutrophil to lymphocyte ratio, (IN) Intratumoural neutrophils, (PN) Peritumoural neutrophils, (SN) Stromal Neutrophils, (OS) Overall Survival, (DFS) Disease-Free Survival, (PFS) Progression-Free Survival, (RFS) Reoccurrence-Free Survival, (CSS) Cancer Specific Survival, (VEGFR) Vascular Endothelial Growth Factor Receptor, (PC) Palliative Chemotherapy, (SBR) Surgical Bowel Resection, (NAC) Neoadjuvant Chemotherapy, (STS) Soft Tissue Sarcoma, (UTR) Urothelial Transurethral Resection, (SR) Surgical Resection, (GNT) Gastroenteropancreatic Neuroendocrine Tumors, (SCC) Squamous Cell Carcinoma, (ST) Systematic Therapy, (MT) Mixed Therapy, (TT) Targeted Therapy, (CLM) Colorectal Liver Metastasis, (NS) Non-surgical, (ICC) Intrahepatic Cholangiocarcinoma, (RFA) Radiofrequency Ablation, (TACE) Trans-Arterial Chemoembolization, (MPM) Malignant Pleural Mesothelioma, (NSCLC) Non-Small Cell Lung Cancer, (NHC), Neck and Head Cancer, (UTUC) Upper Tract Urothelial Carcinoma, (DCRT) Definitive Chemoradiotherapy, (CR) Castration Resistant, (TKI) Tyrosine Kinase Inhibitor.

*All-cancers are defined as a grouping of cancer diagnosis unrelated to site, stage or treatment unless otherwise specified.
†review reported incomplete data on sample size.

Meets criteria	Doesn’t meet criteria

Appendix K: Sensitivity analysis of GRADE

Author	Year	Biomarker	Cancer site	meta-analysis metric	Significance			Sample Size		Largest Study Sig. at <0.05	Pred. Sig.	No Small Study Effects			Excess Sig.	10% Cred. Ceiling Sig.	Level of Evidence
					Sig at <0.05	Sig at 10^{-4}	Sig at 10^{-6}	>1000 Ind.	N>3			T/F	ARC-Thompson	Largest > Random			
Suh J	2019	NLR	Bladder (UTR)	RFS	T	T	T	T	T	T	T	T	0.180	F	No	T	Strong
Ni L	2020	NLR	Endometrial	OS	T	T	T	T	T	T	T	T	0.261	F	No	T	Strong
Sun J	2016	NLR	Gastric	OS	T	T	T	T	T	T	T	T	0.105	F	None Detected	T	Strong
Yang S	2019	NLR	Nasopharyngeal	OS	T	T	T	T	T	T	T	T	0.171	F	No	T	Strong
Yin J	2017	NLR	Nasopharyngeal	PFS	T	T	T	T	T	T	T	T	0.183	F	No	T	Strong
Vartolomei MD	2018	NLR	Non muscle Invasive Bladder	PFS	T	T	T	T	T	T	T	T	0.149	F	No	T	Strong
Yin X	2016	NLR	Prostate CR	OS	T	T	T	T	T	T	T	T	0.340	F	None Detected	T	Strong
Li LQ	2020	NLR	STS	DFS	T	T	T	T	T	T	T	T	0.125	F	No	T	Strong
Jiang T	2018	NLR	Advanced Cancer (Anti-VEGFR)	OS	T	T	T	T	T	T	F	F	0.016	T	No	T	Highly
Jiang T	2018	NLR	Advanced Cancer (Immunotherapy)	PFS	T	T	T	T	T	T	F	F	0.002	F	None Detected	T	Highly
Mei Z	2017	NLR	Composite endpoints	PFS	T	T	T	T	T	T	F	F	0.001	T	No	T	Highly
Paramanathan A	2014	NLR	Composite endpoints	DFS	T	T	T	T	T	T	F	F	<0.001	F	Yes	T	Highly
Tang H	2017	NLR	Biliary Tract	OS	T	T	T	T	T	T	T	T	0.154	F	Yes	T	Highly
Wu S	2018	NLR	Bladder	RFS	T	T	T	T	T	T	F	F	0.005	F	Yes	T	Highly
Ethier JL	2017	NLR	Breast	OS	T	T	T	T	T	T	T	F	0.001	F	None Detected	T	Highly
Chen J	2018	NLR	Breast (Metastasis)	DFS	T	T	T	T	F	T	F	F	0.862	T	No	T	Highly
Duan J	2018	NLR	Breast (SR)	OS	T	T	T	T	T	T	F	F	0.219	T	No	T	Highly
Guo W	2019	NLR	Breast (Triple Negative and Her2 Positive)	DFS	T	T	T	T	T	T	T	F	0.015	F	No	T	Highly
Zheng DC	2016	NLR	Colorectal	OS	T	T	T	T	T	T	F	F	0.010	F	None Detected	T	Highly
Tang H	2016	NLR	CLM	OS	T	T	T	T	T	T	T	F	0.246	T	No	T	Highly
Tang H	2016	NLR	CLM	RFS	T	T	T	T	T	T	F	F	0.455	T	No	T	Highly
Tang H	2016	NLR	CLM (SR)	OS	T	T	T	T	T	T	T	F	0.713	T	No	T	Highly
Szor D	2018	NLR	Gastric (SR)	OS	T	T	T	T	T	T	F	F	0.737	T	No	T	Highly
Ethier JL	2017	NLR	Gynecologic	OS	T	T	T	T	T	T	F	F	<0.001	F	None Detected	T	Highly
Tham T	2018	NLR	Head and Neck	OS	T	T	T	T	T	T	T	F	< 0.001	F	No	T	Highly
Min GT	2018	NLR	Hepatocellular	OS	T	T	T	T	T	T	F	F	<0.001	F	Yes	T	Highly

Min GT	2018	NLR	Hepatocellular	DFS	T	T	T	T	T	T	F	F	<0.001	F	None Detected	T	Highly
Min GT	2018	NLR	Hepatocellular	RFS	T	T	T	T	T	T	F	F	0.001	F	None Detected	T	Highly
Liu L	2019	NLR	Hepatocellular (Sorfenib)	OS	T	T	T	T	T	T	T	F	0.001	T	No	T	Highly
Wang Y	2018	NLR	Hepatocellular (SR)	RFS	T	T	T	T	T	T	T	F	0.079	F	None Detected	T	Highly
Wang Y	2018	NLR	Hepatocellular (SR)	OS	T	T	T	T	T	T	T	F	0.010	F	None Detected	T	Highly
Wang Y	2018	NLR	Hepatocellular (SR)	DFS	T	T	T	T	T	T	T	F	0.052	F	Yes	T	Highly
Li S	2020	NLR	Hepatocellular (TACE)	OS	T	T	T	T	T	T	T	F	0.031	F	Yes	T	Highly
Jiang T	2018	NLR	Immunotherapy (Advanced Cancer)	OS	T	T	T	T	T	T	T	F	0.009	T	No	T	Highly
Tan Q	2018	NLR	Immunotherapy (Immune checkpoint inhibitors)	OS	T	T	T	T	T	T	F	F	<0.001	F	No	T	Highly
Tan Q	2018	NLR	Immunotherapy (Immune checkpoint inhibitors)	PFS	T	T	T	T	T	T	F	F	<0.001	F	Yes	T	Highly
Zhao QT	2015	NLR	Lung (both)	OS	T	T	T	T	T	T	F	F	0.001	T	None Detected	T	Highly
Xie X	2019	NLR	Melanoma (Immune checkpoint inhibitors)	OS	T	T	T	T	T	T	F	F	0.050	F	None Detected	T	Highly
Li X	2018	NLR	NAC	OS	T	T	T	T	T	T	F	F	<0.001	T	No	T	Highly
Gu XB	2015	NLR	NSCLC	PFS	T	T	T	T	T	T	F	F	<0.001	F	None Detected	T	Highly
Jiang T	2019	NLR	NSCLC (PD-1 inhibitor)	OS	T	T	T	T	T	T	T	F	0.006	F	Yes	T	Highly
Jiang T	2019	NLR	NSCLC (PD-1 inhibitor)	PFS	T	T	T	T	T	T	T	F	0.742	T	No	T	Highly
Wang Z	2019	NLR	NSCLC (ST)	PFS	T	T	T	T	T	T	F	F	< 0.001	F	None Detected	T	Highly
Zhang X	2018	NLR	Oesophageal	OS	T	T	T	T	T	T	F	F	0.039	T	No	T	Highly
Pirozzolo G	2019	NLR	Oesophageal (DCRT)	OS	T	T	T	T	F	T	F	T	0.513	F	No	T	Highly
Zhang X	2018	NLR	Oesophageal (Surgery)	OS	T	T	T	T	T	T	F	F	0.076	T	No	T	Highly
Pirozzolo G	2019	NLR	Oesophageal (Surgery+/-Chemo)	OS	T	T	T	T	T	T	F	T	0.225	F	None Detected	T	Highly
Zhang X	2018	NLR	Oesophageal SCC	OS	T	T	T	T	T	T	F	F	0.331	T	No	T	Highly
Yang Y	2018	NLR	Oral cavity	OS	T	T	T	T	T	T	T	F	0.747	T	No	T	Highly
Yang Y	2018	NLR	Oral cavity	DFS	T	T	T	T	T	T	T	F	0.045	T	No	T	Highly
Zhou Y	2018	NLR	Pancreatic	OS	T	T	T	T	T	T	F	F	<0.001	F	Yes	T	Highly
Mowbray N	2018	NLR	Pancreatic (SR)	OS	T	T	T	T	T	T	T	F	0.054	F	None Detected	T	Highly
Guo J	2018	NLR	Prostate	PFS	T	T	T	T	T	T	T	F	0.178	T	No	T	Highly
Guo J	2018	NLR	Prostate (Metastatic)	OS	T	T	T	T	T	T	T	F	0.023	F	Yes	T	Highly
Guan Y	2020	NLR	Prostate CR (Enzalutamide)	OS	T	T	T	T	T	T	F	T	0.110	F	None Detected	T	Highly

Luo Y	2015	NLR	Renal	OS	T	T	T	T	T	T	T	F	0.017	F	None Detected	T	Highly
Semeniuk-Wojtas A	2018	NLR	Renal (TKI)	OS	T	T	T	T	T	T	T	T	0.385	F	Yes	T	Highly
Boissier R	2017	NLR	Renal Advanced	OS	T	T	T	T	T	T	F	F	0.009	T	None Detected	T	Highly
Liu G	2018	NLR	STS	OS	T	T	T	T	T	T	F	F	0.018	T	No	T	Highly
Suh J	2019	NLR	Urothelial (Chemotherapy)	OS	T	T	T	T	T	T	T	F	0.131	T	None Detected	T	Highly

A) Not applicable, (T) True, (F) False, (HR) Hazard ratio, (NLR) Neutrophil to lymphocyte ratio, (IN) Intratumoural neutrophils, (PN) Peritumoural neutrophils, (SN) Stromal Neutrophils, (OS) Overall Survival, (DFS) Disease-Free Survival, FS) Progression-Free Survival, (RFS) Reoccurrence-Free Survival, (CSS) Cancer Specific Survival, (VEGFR) Vascular Endothelial Growth Factor Receptor, (PC) Palliative Chemotherapy, (SBR) Surgical Bowel Resection, (NAC) Neoadjuvant hemotherapy, (STS) Soft Tissue Sarcoma, (UTR) Urothelial Transurethral Resection, (SR) Surgical Resection, (GNT) Gastroenteropancreatic Neuroendocrine Tumors, (SCC) Squamous Cell Carcinoma, (ST) Systematic Therapy, (MT) Mixed herapy, (TT) Targeted Therapy, (CLM) Colorectal Liver Metastasis, (NS) Non-surgical, (ICC) Intrahepatic Cholangiocarcinoma, (RFA) Radiofrequency Ablation, (TACE) Trans-Arterial Chemoembolization, (MPM) Malignant Pleural esothelioma, (NSCLC) Non-Small Cell Lung Cancer, (NHC), Neck and Head Cancer, (UTUC) Upper Tract Urothelial Carcinoma, (DCRT) Definitive Chemoradiotherapy, (CR) Castration Resistant, (TKI) Tyrosine Kinase Inhibitor.

All-cancers are defined as a grouping of cancer diagnosis unrelated to site, stage or treatment unless otherwise specified.

review reported incomplete data on sample size.

D) None detected – binomial probability test yielded a p-value>0.10.

o) – no excess significance, observed<expected.

o (NS)) - No excess significance, no observed significant studies.

Meets criteria	Doesn't meet criteria

Appendix L: Ranking based in precision versus effect size

Appendix 12 Figure 1 – Inverse Standard Error versus log(Hazard Ratio)

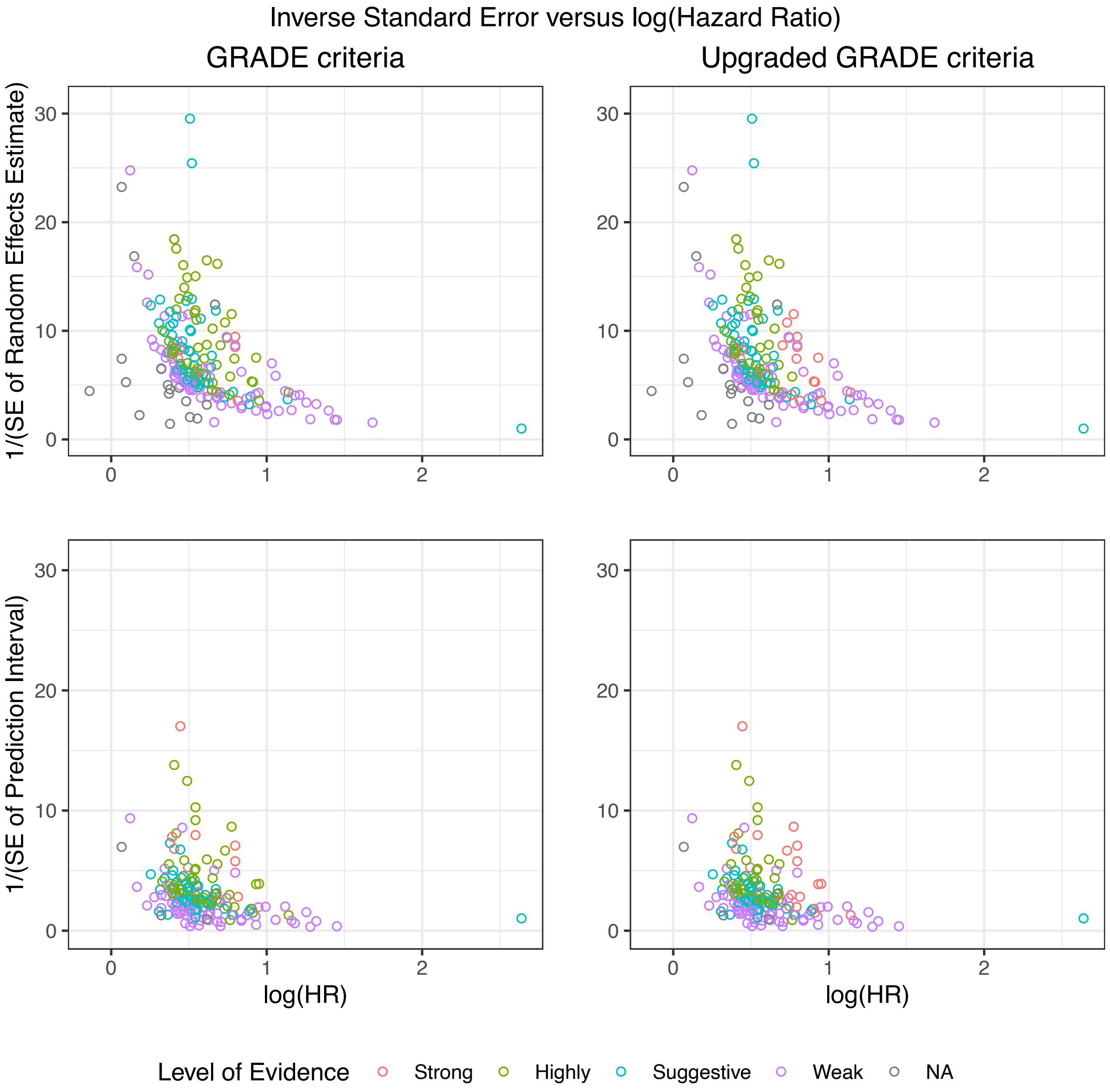

Appendix 12 Table 1 – Ranking of evidence comparison

Study	Association	GRADE	Upgrade	Funnel Rank (sePI)	Funnel Rank (seCI)
Dolan R	NLR-Composite endpoints Operable (OS)	Suggestive	Suggestive	50	1
Mei Z	NLR-Composite endpoints (OS)	Suggestive	Suggestive	40	2
Hu G	NLR-Bladder (Radical Cystectomy) (OS)	Weak	Weak	5	3
Hu G	NLR-Bladder (Radical Cystectomy and NAC) (OS)	Uncertain	Uncertain	14	4
Wang Y	NLR-Hepatocellular (SR) (DFS)	Highly	Highly	2	5
Dolan R	NLR-Composite endpoints Operable (CSS)	Suggestive	Suggestive	52	6
Tang H	NLR-Biliary Tract (OS)	Highly	Highly	9	7
Shen M	IN-NSCLC (OS)	Uncertain	Uncertain	180	8
Shuangshuang L	NLR-Hepatocellular (TACE) (OS)	Highly	Highly	18	9
Sun J	NLR-Gastric (OS)	Highly	Highly	22	10
Guo W	NLR-Breast (Triple Negative and Her2 Positive) (DFS)	Highly	Highly	32	11
Suh J	NLR-Urothelial (Radical Cystectomy) (PFS)	Weak	Weak	49	12
Xiao WK	NLR-Hepatocellular (RFA) (OS)	Weak	Weak	182	13
Tham T	NLR-Head and Neck (OS)	Highly	Highly	25	14
Mei Z	NLR-Composite endpoints (PFS)	Highly	Highly	54	15
Wang Y	NLR-Hepatocellular (SR) (OS)	Highly	Highly	19	16
Min GT	NLR-Hepatocellular (OS)	Highly	Highly	114	17
Zhao QT	NLR-Lung (both) (OS)	Highly	Highly	64	18
Li X	NLR-Bladder and Upper Urinary (PFS)	Suggestive	Suggestive	35	19
Hu G	NLR-Bladder (OS)	Suggestive	Suggestive	59	20
Li X	NLR-Bladder and Upper Urinary (CSS)	Suggestive	Suggestive	42	21
Pirozzolo G	NLR-Oesophageal (Neo+Surgery) (OS)	Weak	Weak	113	22
Jiang T	NLR-Advanced Cancer (Immunotherapy) (PFS)	Highly	Highly	69	23
Jiang T	NLR-Immunotherapy (Advanced Cancer) (OS)			70	24
Mori K	NLR-UTUC (Radical Cystectomy) (CSS)	Suggestive	Suggestive	30	25
Wu S	NLR-Bladder (RFS)	Highly	Highly	41	26
Ethier JL	NLR-Gynecologic (OS)	Highly	Highly	75	27
Cho J	NLR-Head and Neck (SCC) (OS)	Suggestive	Suggestive	56	28
Yang L	NLR-Head and Neck (SCC) (CSS)	Suggestive	Suggestive	12	29
Wang Y	NLR-Hepatocellular (SR) (RFS)	Highly	Highly	26	30
Tang H	NLR-CLM (OS)	Highly	Strong	7	31
Wu S	NLR-Bladder (Metastasis) (OS)	Weak	Weak	23	32
Tang H	NLR-Biliary Tract (RFS)	Weak	Weak	24	33

Sun J	NLR-Gastric (DFS)	Weak	Weak	8	34
Lei Y	NLR-Glioma (OS)	Suggestive	Suggestive	68	35
Guo W	NLR-Breast (Triple Negative and Her2 Positive) (OS)	Suggestive	Suggestive	90	36
Liu L	NLR-Hepatocellular (Sorfenib) (OS)	Highly	Highly	47	37
Tang H	NLR-CLM (SR) (OS)	Highly	Strong	17	38
Yang L	NLR-Larynx (DFS)	Suggestive	Suggestive	136	39
Yu Y	NLR-Lung (Surgery) (OS)	Suggestive	Suggestive	55	40
Wu J	NLR-Cervical (OS)	Suggestive	Suggestive	57	41
Zheng DC	NLR-Colorectal (OS)	Highly	Highly	80	42
Shen M	IN-Composite endpoints (OS)	Suggestive	Suggestive	83	43
Zhang X	NLR-Oesophageal SCC (OS)	Highly	Highly	39	44
Li H	NLR-Colorectal (SR) (OS)	Suggestive	Suggestive	61	45
Lu Y	NLR-NSCLC (OS)	Suggestive	Suggestive	36	46
Tham T	NLR-Head and Neck (No Surgery) (OS)	Suggestive	Suggestive	31	47
Suh J	NLR-Bladder (UTR) (RFS)	Strong	Strong	20	48
Xie X	NLR-Melanoma (Immune checkpoint inhibitors) (PFS)	Weak	Weak	84	49
Paramanathan A	NLR-Composite endpoints (DFS)	Highly	Strong	116	50
Suh J	NLR-Urothelial (Chemotherapy) (PFS)	Weak	Weak	183	51
Suh J	NLR-Urothelial (Chemotherapy) (OS)	Highly	Highly	21	52
Duan J	NLR-Breast (SR) (DFS)	Suggestive	Suggestive	87	53
Malietzis G	NLR-Colorectal (PC) (DFS)	Weak	Weak	143	54
Cao J	NLR-Prostate (OS)	Suggestive	Suggestive	28	55
Min GT	NLR-Hepatocellular (RFS)	Highly	Highly	103	56
Ding Y	NLR-Melanoma (PFS)	Weak	Weak	29	57
Jiang T	NLR-Advanced Cancer (Anti-VEGFR) (OS)	Highly	Strong	102	58
Su S	NLR-Urothelial (OS)	Weak	Weak	79	59
Yang S	NLR-Nasopharyngeal (OS)	Strong	Strong	11	60
Mascarella M	NLR-Larynx (OS)	Suggestive	Suggestive	33	61
Ni L	NLR-Endometrial (OS)	Strong	Strong	13	62
Tan Q	NLR-Immunotherapy (Immune checkpoint inhibitors) (PFS)	Highly	Highly	88	63
Zhang X	NLR-Oesophageal (OS)	Highly	Highly	58	64
Zhan H	NLR-Melanoma (OS)	Suggestive	Suggestive	97	65
Xie X	NLR-NSCLC (Immune Checkpoint Inhibitors) (PFS)	Weak	Weak	73	66
Yin X	NLR-Prostate CR (OS)	Strong	Strong	1	67
Liu X	NLR-Breast (DFS)	Suggestive	Suggestive	77	68

Zhang X	NLR-Oesophageal (Surgery) (OS)	Highly	Highly	67	69
Yin J	NLR-Nasopharyngeal (PFS)	Strong	Strong	15	70
Tham T	NLR-Head and neck (DFS)	Suggestive	Suggestive	62	71
Su L	NLR-Nasopharyngeal (CSS)	Suggestive	Suggestive	146	72
Wang Z	NLR-NSCLC (ST) (PFS)	Highly	Highly	46	73
Zhang X	NLR-Oesophageal (PFS)	Weak	Weak	45	74
Tham T	NLR-Head and neck (PFS)	Weak	Weak	81	75
Guan Y	NLR-Prostate CR (Enzalutamide) (OS)	Highly	Highly	53	76
Zheng DC	NLR-Colorectal (PFS)	Suggestive	Suggestive	98	77
Wu J	NLR-Cervical (PFS)	Suggestive	Suggestive	109	78
Zhang X	NLR-Oesophageal (CSS)	Weak	Weak	76	79
Ethier JL	NLR-Breast (OS)	Highly	Strong	44	80
Min GT	NLR-Hepatocellular (DFS)	Highly	Highly	122	81
Chen	NLR-MPM (OS)	Weak	Weak	106	82
Tan Q	NLR-Immunotherapy (Immune checkpoint inhibitors) (OS)	Highly	Strong	121	83
Li H	NLR-Colorectal (SR) (DFS)	Weak	Weak	85	84
Xiao WK	NLR-Hepatocellular (RFA) (DFS)	Uncertain	Uncertain	178	85
Jiang T	NLR-NSCLC (PD-1 inhibitor) (PFS)	Highly	Highly	3	86
Chen J	NLR-Breast (Metastasis) (OS)	Weak	Weak	198	87
Xie X	NLR-Nivolumab (PFS)	Weak	Weak	74	88
Liu G	NLR-STS (OS)	Highly	Highly	60	89
Tang H	NLR-CLM (RFS)	Highly	Highly	93	90
Zeng Q	NLR-Multiple Myeloma (OS)	Suggestive	Suggestive	101	91
Wang Y	NLR-Oral SCC (OS)	Suggestive	Suggestive	16	92
Cho J	NLR-Head and Neck (SCC) (DFS)	Suggestive	Suggestive	111	93
Xiao WK	NLR-Hepatocellular (MT) (OS)	Weak	Weak	161	94
Luo Y	NLR-Renal (CSS)	Uncertain	Uncertain	151	95
Gu XB	NLR-NSCLC (PFS)	Highly	Highly	65	96
Pirozzolo G	NLR-Oesophageal (DCRT) (OS)	Highly	Highly	89	97
Shen M	IN-Hepatocellular and ICC (OS)	Weak	Weak	132	98
Zhao QT	NLR-Lung (both) (PFS)	Weak	Weak	124	99
Suh J	NLR-Urothelial (Radical Cystectomy) (CSS)	Suggestive	Suggestive	104	100
Suh J	NLR-Urothelial (Radical Cystectomy) (OS)	Weak	Weak	112	101
Zhou Q	NLR-Ovarian (PFS)	Suggestive	Suggestive	95	102
Cao D	NLR-NSCLC (Nivoumab) (PFS)	Weak	Weak	119	103

Mowbray N	NLR-Pancreatic (SR) (OS)	Highly	Highly	4	104
Li H	NLR-Colorectal (SR) (RFS)	Weak	Weak	158	105
Mori K	NLR-UTUC (Nephroureterectomy) (CSS)	Suggestive	Suggestive	51	106
Guo J	NLR-Prostate (Metastatic) (OS)	Highly	Highly	38	107
Yang L	NLR-Head and Neck (SCC) (PFS)	Weak	Weak	135	108
Zhang X	NLR-Gastric (PFS)	Weak	Weak	190	109
Zhang J	NLR-Colorectal (DFS)	Suggestive	Suggestive	107	110
Li X	NLR-NAC (OS)	Highly	Highly	86	111
Zhou Y	NLR-Pancreatic (OS)	Highly	Highly	82	112
Yang Y	NLR-Oral cavity (OS)	Highly	Highly	37	113
Yang Y	NLR-Oral cavity (DFS)	Highly	Highly	6	114
Luo Y	NLR-Upper Urinary (RFS)	Weak	Weak	188	115
Li LQ	NLR-STS (DFS)	Strong	Strong	10	116
Yang L	NLR-Hypopharynx (OS)	Weak	Weak	159	117
Ni L	NLR-Endometrial (PFS)	Suggestive	Suggestive	100	118
Zhang X	NLR-Oesophageal (DFS)	Weak	Weak	117	119
Chen J	NLR-Breast (Metastasis) (DFS)	Highly	Highly	160	120
Zhou Q	NLR-Ovarian (OS)	Suggestive	Suggestive	110	121
Tang H	NLR-CLM (SR) (RFS)	Weak	Weak	140	122
Wang Z	NLR-NSCLC (TT) (PFS)	Weak	Weak	130	123
Guan Y	NLR-Prostate CR (Abitaterone) (OS)	Suggestive	Suggestive	48	124
Suh J	NLR-Urothelial (Nephroureterectomy) (PFS)	Suggestive	Suggestive	99	125
Wang Z	NLR-NSCLC (Chemotherapy) (PFS)	Weak	Weak	63	126
Pirozzolo G	NLR-Oesophageal (Surgery+/-Chemo) (OS)	Highly	Highly	91	127
Vartolomei M	NLR-Urothelial (RFS)	Weak	Weak	115	128
Duan J	NLR-Breast (SR) (OS)	Highly	Strong	126	129
Cao D	NLR-NSCLC (Nivoumab) (OS)	Weak	Weak	94	130
Xie X	NLR-Melanoma (Immune checkpoint inhibitors) (OS)	Highly	Strong	139	131
Zhang X	NLR-Oesophageal (RFS)	Weak	Weak	169	132
Suh J	NLR-Urothelial (Nephroureterectomy) (OS)	Suggestive	Suggestive	154	133
Shen M	SN-Composite endpoints (OS)	Uncertain	Uncertain	179	134
Cao J	NLR-Prostate CR (PFS)	Suggestive	Suggestive	96	135
Wei Y	NLR-Urinary (OS)	Suggestive	Suggestive	72	136
Mu S	NLR-Large B (PFS)	Weak	Weak	134	137
Luo Y	NLR-Renal (OS)	Highly	Highly	34	138

Wang Z	NLR-NSCLC (ST) (OS)	Suggestive	Suggestive	108	139
Yang L	NLR-Oropharynx (DFS)	Weak	Weak	156	140
Zhang X	NLR-Oesophageal (No Surgery) (OS)	Suggestive	Suggestive	131	141
Vartolomei MD	NLR-Non muscle Invasive Bladder (RFS)	Suggestive	Suggestive	194	142
Su S	NLR-Urothelial (DFS)	Uncertain	Uncertain	184	143
Yu Y	NLR-Head and Neck (RFS)	Weak	Weak	152	144
Cao J	NLR-Prostate Localised (RFS)	Weak	Weak	142	145
Zhou Y	NLR-Pancreatic (DFS)	Weak	Weak	129	146
Wang Z	NLR-NSCLC (Chemotherapy) (OS)	Suggestive	Suggestive	128	147
Tham T	NLR-Head and Neck (SR) (OS)	Weak	Weak	172	148
Guan Y	NLR-Prostate CR (Enzalutamide) (PFS)	Uncertain	Uncertain	189	149
Boissier R	NLR-Renal Localised (RFS)	Weak	Weak	137	150
Boissier R	NLR-Renal Localised (OS)	Uncertain	Uncertain	187	151
Yang JJ	NLR-Pancreatic (CSS)	Weak	Weak	173	152
Boissier R	NLR-Renal Advanced (OS)	Highly	Highly	105	153
Shen M	IN-Head and Neck (OS)	Weak	Weak	168	154
Mu S	NLR-Large B (OS)	Weak	Weak	144	155
Chen J	NLR-Breast (No Metastasis) (DFS)	Uncertain	Uncertain	177	156
Zhou Y	NLR-GNT (OS)	Weak	Weak	118	157
Guo J	NLR-Prostate (PFS)	Highly	Highly	66	158
Xu ZG	NLR-Hepatocellular (Transplant) (OS)	Suggestive	Suggestive	147	159
Xie X	NLR-Ipilimumuab (PFS)	Weak	Weak	174	160
Szor D	NLR-Gastric (SR) (OS)	Highly	Strong	149	161
Liu X	NLR-Breast (Triple-negative) (OS)	Weak	Weak	123	162
Yin X	NLR-Prostate Localised (OS)	Uncertain	Uncertain	185	163
Xie X	NLR-Ipilimumuab (OS)	Weak	Weak	153	164
Li X	NLR-NAC (RFS)	Weak	Weak	92	165
Semeniuk-Wojtas A	NLR-Renal (TKI) (OS)	Highly	Strong	71	166
Shen M	IN-Composite endpoints (CSS)	Weak	Weak	201	167
Zhou Y	NLR-GNT (RFS)	Weak	Weak	170	168
Suh J	NLR-Bladder (UTR) (PFS)	Suggestive	Suggestive	127	169
Luo Y	NLR-Renal (RFS)	Weak	Weak	166	170
Wang Z	NLR-NSCLC (TT) (OS)	Weak	Weak	145	171
Na N	NLR-Renal (PFS)	Suggestive	Suggestive	125	172
Zeng Q	NLR-Multiple Myeloma (PFS)	Weak	Weak	162	173

Semeniuk-Wojtas A	NLR-Renal (TKI) (PFS)	Weak	Weak	141	174
Li LQ	NLR-STS (Synovial Sarcoma) (OS)	Weak	Weak	150	175
Tang H	NLR-CLM (Non-surgical) (OS)	Suggestive	Suggestive	200	176
Jiang T	NLR-NSCLC (PD-1 inhibitor) (OS)	Highly	Strong	43	177
Vartolomei MD	NLR-Non muscle Invasive Bladder (PFS)	Strong	Strong	78	178
Guan Y	NLR-Prostate CR (Abitaterone) (PFS)	Uncertain	Uncertain	191	179
Liu X	NLR-Breast (Triple-negative) (DFS)	Weak	Weak	138	180
Li X	NLR-NAC (CSS)	Weak	Weak	148	181
Xu ZG	NLR-Hepatocellular (Transplant) (RFS)	Weak	Weak	165	182
Wang Z	NLR-NSCLC (Immunotherapy) (OS)	Suggestive	Suggestive	133	183
Feng J	NLR-Thyroid (DFS)	Uncertain	Uncertain	157	184
Liu X	NLR-Breast (CSS)	Weak	Weak	167	185
Tang H	NLR-CLM (Non-surgical) (RFS)	Weak	Weak	195	186
Shen M	IN-Renal (OS)	Weak	Weak	196	187
Xie X	NLR-Nivolumab (OS)	Weak	Weak	120	188
Vartolomei MD	NLR-Non muscle Invasive Bladder (High Risk) (RFS)	Weak	Weak	164	189
Boissier R	NLR-Renal Advanced (PFS)	Weak	Weak	163	190
Duan J	NLR-Breast (SR) (RFS)	Weak	Weak	202	191
Vartolomei MD	NLR-Non muscle Invasive Bladder (High Risk) (PFS)	Weak	Weak	171	192
Li LQ	NLR-STS (Liposarcoma) (OS)	Weak	Weak	199	193
Mellor K	NLR-Gastric (SR) (DFS)	Weak	Weak	197	194
Shen M	IN-Gastric (OS)	Uncertain	Uncertain	181	195
Shen M	PN-Composite endpoints (OS)	Uncertain	Uncertain	192	196
Luo XF	NLR-Gastrointestinal Stromal (OS)	Uncertain	Uncertain	193	197
Dong YW	NLR-Rectal (RFS)	Weak	Weak	176	198
Zhou Y	NLR-Pancreatic Neuroendocrine Tumour (OS)	Weak	Weak	203	199
Dong YW	NLR-Rectal (DFS)	Weak	Weak	175	200
Wang Y	NLR-Oral SCC (DFS)	Weak	Weak	27	201
Zhou Y	NLR-Pancreatic Neuroendocrine Tumour (RFS)	Weak	Weak	204	202
Chen J	NLR-Breast (No Metastasis) (OS)	Uncertain	Uncertain	186	203
Dong YW	NLR-Rectal (OS)	Suggestive	Suggestive	155	204

Appendix M: AMSTAR 2 quality rating of meta-analysis with highly suggestive or strong evidence

Author	Year	Indicators	Cancer sites	meta-analysis metrics	Level of Evidence	AMSTAR 2 Quality Rating
Ni L	2020	NLR	Endometrial carcinoma	OS	Strong	High
Suh J	2019	NLR	Urothelial transurethral resection of bladder tumor	RFS	Strong	High
			Urothelial Carcinoma with Chemo	OS	Highly	
Vartolomei MD	2018	NLR	Non muscle Invasive Bladder	PFS	Strong	Moderate
Yin J	2017	NLR	Nasopharyngeal	PFS	Strong	Moderate
Yin X	2016	NLR	Prostate CR	OS	Strong	Low
Li LQ	2020	NLR	Soft tissue sarcoma	DFS	Strong	Critically Low
Yang S	2019	NLR	Nasopharyngeal	OS	Strong	Critically Low
Duan J	2018	NLR	Breast Surgical Resection	OS	Strong (upgraded)	High
Jiang T	2018	NLR	Advanced Cancer with Anti-VEGFR	OS	Strong (upgraded)	Moderate
Tang H	2016	NLR	CLM	OS	Strong (upgraded)	Moderate
			CLM SR	OS	Strong (upgraded)	
Szor D	2018	NLR	CLM SR	OS	Strong (upgraded)	Moderate
Ethier JL	2017	NLR	Breast	OS	Strong (upgraded)	Moderate
Tan Q	2018	NLR	Immune checkpoint inhibitors	OS	Strong (upgraded)	Low
Paramanathan A	2014	NLR	Composite endpoints*	DFS	Strong (upgraded)	Low
Semeniuk-Wojtas A	2018	NLR	Renal (TKI)	OS	Strong (upgraded)	Critically Low
Xie X	2019	NLR	Melanoma with Immune checkpoint inhibitors	OS	Strong (upgraded)	Critically Low
Jiang T	2019	NLR	NSCLC treated with PD-1 inhibitor	OS	Strong (upgraded)	High
				PFS	Highly	
Zhou Y	2018	NLR	Pancreatic	OS	Highly	High
Liu L	2019	NLR	Hepatocellular carcinoma treated with Sorfenib	OS	Highly	High
Zhang X	2018	NLR	Oesophageal	OS	Highly	High
			Oesophageal Surgery	OS	Highly	
			Oesophageal Squamous cell carcinoma	OS	Highly	

Wang Y	2018	NLR	Hepatocellular Resection	OS	Highly	High
				DFS	Highly	
Mei Z	2017	NLR	Composite endpoints*	PFS	Highly	Moderate
Jiang T	2018	NLR	Advanced Cancer with immunotherapy	OS	Highly	Moderate
				PFS	Highly	
Zheng DC	2016	NLR	Colorectal	OS	Highly	Moderate
Mowbray N	2018	NLR	Pancreatic SR	OS	Highly	Moderate
Wang Y	2018	NLR	Hepatocellular SR	RFS	Highly	Moderate
Tang H	2017	NLR	Biliary Tract	OS	Highly	Moderate
Luo Y	2015	NLR	Renal	OS	Highly	Moderate
Wu S	2018	NLR	Bladder	RFS	Highly	Moderate
Min GT	2018	NLR	Hepatocellular	OS	Highly	Moderate
				DFS	Highly	
				RFS	Highly	
Gu XB	2015	NLR	NSCLC	PFS	Highly	Moderate
Tang H	2016	NLR	CLM	RFS	Highly	Moderate
Guo W	2019	NLR	Breast (triple negative and Her2 positive)	DFS	Highly	Moderate
Pirozzolo G	2019	NLR	Oesophageal DCRT	OS	Highly	Moderate
			Oesophageal Surgery+/-chemo	OS	Highly	
Tham T	2018	NLR	Head and Neck	OS	Highly	Moderate
Wang Z	2019	NLR	NSCLC with systematic therapy	PFS	Highly	Low
Tan Q	2018	NLR	Immune checkpoint inhibitors	PFS	Highly	Low
Li X	2018	NLR	Neoadjuvant Chemotherapy	OS	Highly	Low
Sun J	2016	NLR	Gastric	OS	Highly	Low
Liu X	2017	NLR	Breast	DFS	Highly	Low
Zhao QT	2015	NLR	Lung (both)	OS	Highly	Low
Guo J	2018	NLR	Prostate	PFS	Highly	Low
			Prostate metastatic	OS	Highly	

Guan Y	2020	NLR	Prostate (castration resistant) with enzalutamide	OS	Highly	Low
Boissier R	2017	NLR	Renal Advanced	OS	Highly	Critically Low
Ethier JL	2017	NLR	Gynaecologic	OS	Highly	Critically Low
Liu G	2018	NLR	Soft Tissue Sarcoma	OS	Highly	Critically Low
Li S	2020	NLR	Hepatocellular (TACE)	OS	Highly	Critically Low
Yang Y	2018	NLR	Oral cavity	OS	Highly	Critically Low
				DFS	Highly	

N=number of component studies with in review, (NA) Not Applicable, (HR), Hazard ratio, (NLR) Neutrophil to Lymphocyte Ratio, (IN) Intratumoural Neutrophils, (PN) Peritumoural Neutrophils, (SN) Stromal Neutrophils, (OS) Overall Survival, (DFS) Disease-Free Survival, (PFS) Progression-Free Survival, (RFS) Reoccurrence-Free Survival, (CSS) Cancer Specific Survival, (VEGFR) Vascular Endothelial Growth Factor Receptor, (PC) Palliative Chemotherapy, (SBR) Surgical Bowel Resection, (SR) Surgical Resection, (UUB) Upper Urinary and Bladder, (CLM) Colorectal Liver Metastasis, (NS) Non-surgical, (HCC) Hepatocellular Carcinoma, (ICC) Intrahepatic Cholangiocarcinoma, (RFA) Radiofrequency Ablation, (TACE) Trans-Arterial Chemoembolization, (MPM) Malignant Pleural Mesothelioma, (NSCLC) Non-Small Cell Lung Cancer, (NHC), Neck and Head Cancer, (CR) Castration Resistant, (TKI) Tyrosine Kinase Inhibitor. *Composite endpoints are defined as a grouping of cancer diagnosis unrelated to site, stage or treatment unless otherwise specified. †review reported incomplete data on sample size.

Appendix N: Assessment of quality of component studies

Appendix N, Table 1 – Quality Assessment Tools used by Reviews

Review		Tools and Guidance Used by Review	
Author	Year	Quality Assessment Tool Used for Component Studies	Systematic Review Guidance Used
Shen M	2014	None	PRISMA
Mei Z	2017	QUIPS	PRISMA
Dolan R	2017	None	PRISMA
Paramanathan	2014	QUIPS	Not stated
Jiang T	2018	QUIPS	PRISMA
Tan Q	2018	NOS	PRISMA
Sacdalan DB	2018	Cochrane	Not stated
Li X	2018	NOS	PRISMA
Sun J	2016	NOS	PRISMA
Szor DJ	2018	NOS	PRISMA
Luo ZF	2018	NOS	Not stated
Zhang X	2014	NOS	PRISMA
Mellor KL	2018	NOS	PRISMA
Huang Y	2017	NOS	Not stated
Zheng DC	2016	NOS	MOOSE
Malietzis G	2014	SIGN	PRISMA
Zhang J	2017	NOS	Not stated
Dong Y	2016	NOS	Not stated
Tang H	2016	NOS	MOOSE
Zhou Y	2018	NOS	PRISMA
Mowbray N	2018	QUIPS	PRISMA
Yang J	2015	NOS	Not stated
Ethier J	2017	None	PRISMA
Wu J	2017	NOS	PRISMA
Zhou Q	2017	NOS	PRISMA
Ethier J	2017	QUIPS	PRISMA
Chen J	2018	None	PRISMA
Liu X	2017	None	PRISMA
Min GT	2018	NOS	PRISMA
Xu ZG	2018	NOS	PRISMA
Xiao WK	2014	NOS	Not stated
Xu W	2018	NOS	PRISMA
Sun XD	2016	NOS	Not stated
Wang Y	2018	NOS	PRISMA
Tang H	2017	NOS	MOOSE
Mu S	2018	NOS	REMARK
Zhang J	2017	None	PRISMA
Zhou Y	2018	NOS	Not stated
Gu XB	2015	NOS	Not stated
Zhoa QT	2015	NOS	Not stated
Yu Y	2018	NOS	PRISMA
Yu Y	2017	None	Not stated
Mascarella M	2018	None	PRISMA
Takenaka Y	2017	NOS	PRISMA
Yin J	2017	NOS	PRISMA
Su L	2017	NOS	PRISMA
Wang Y	2018	NOS	PRISMA
Chen N	2017	NOS	Not stated
Zhan H	2018	NOS	Not stated

Ding Y	2018	NOS	PRISMA
Luo Y	2015	NOS	MOOSE
Boissier R	2017	None	PRISMA
Semeniuk-Wojtas A	2018	GRADE	Not stated
Na N	2016	NOS	Not stated
Lucca I	2015	NOS	PRISMA
Vartolomei M	2018	NOS	PRISMA
Wei Y	2014	MOOSE	PRISMA
Li X	2017	NOS	MOOSE
Wu S	2018	NOS	Not stated
Cao J	2016	None	PRISMA
Yin X	2016	MOOSE	PRISMA
Vartolomei M	2018	NOS	PRISMA
Zhou Q	2017	NOS	PRISMA
Zhang X	2018	NOS	PRISMA
Zeng Q	2018	NOS	PRISMA
Yang Y	2018	NOS	PRISMA
Yang S	2019	NOS	PRISMA
Yang L	2019	NOS	Not stated
Xie X	2019	Not stated	Not stated
Wang Z	2019	NOS	Not stated
Wang Y	2018	NOS	PRISMA
Tham T	2018	QUIPS	PRISMA
Suh J	2019	NOS	PRISMA
Su S	2019	NOS	PRISMA & AMSTAR
Pirozzolo G	2019	NOS	PRISMA
Ni L	2020	NOS	PRISMA
Mori K	2020	NOS	PRISMA
Mori K	2020	NOS	PRISMA
Lu Y	2020	NOS	PRISMA
Liu L	2019	NOS	PRISMA
Liu G	2018	NOS	Not stated
Li S	2020	NOS	PRISMA
Li LQ	2020	NOS	Not stated
Li H	2019	NOS	PRISMA
Lei Y	2019	NOS	PRISMA
Jiang T	2019	NOS	PRISMA
Hu G	2019	NOS	Not stated
Guo W	2019	QUIPS	Not stated
Guo J	2018	NOS	PRISMA & AMSTAR
Guan Y	2020	NOS	PRISMA
Feng J	2020	NOS	PRISMA
Duan J	2018	SIGN	PRISMA
Cho J	2018	NOS	PRISMA
Cao D	2018	NOS	Not stated

(QUIPS) Quality in Prognostic Studies, (NOS) Newcastle-Ottawa Scale, (SIGN) Scottish International Guideline Network, (MOOSE) Meta-analyses Of Observational Studies in Epidemiology, (PRISMA) Preferred Reporting Items for Systematic Reviews and Meta-analyses, (REMARK) Reporting Recommendations for Tumor Marker Prognostic Studies.

Appendix N, Table 2 – QUIPS Risk of Bias Assessment Instrument for Prognostic Factor Studies (Reviews with Strong Evidence)

Review					Component Study		Risk Across QUIPS Domains					
Author	Year	Biomarker	Cancer Site	Meta-analysis Metric	Author	Year	Study Participation	Study Attrition	Prognostic Factor Measurement	Outcome Measurement	Study Confounding	Statistical Analysis and Reporting
Vartolomei MD	2018	NLR	Non-muscle Invasive Bladder	PFS	Mano*	2015	Low	Moderate	Low	Low	Low	Low
					Favilla*	2016	Low	Low	Low	Low	Low	Low
					Mbeutcha	2016	Low	Low	Low	Low	Low	Low
					Ogihara*	2016	Low	Moderate	Low	Low	Low	Low
					Mao	2017	Low	Moderate	Low	Low	Low	Low
					Ramos	2017	Moderate	Moderate	Low	Moderate	Low	Low
Yin J	2017	NLR	Nasopharyngeal	PFS	Sun*	2016	Low	Moderate	Low	Low	Low	Low
					Chen*	2014	Low	Moderate	Low	Low	Low	Low
					Jin	2015	Low	Low	Moderate	Low	Low	Low
					He*	2012	Low	Moderate	Moderate	Low	Low	Low
					An*	2011	Low	High	Low	Low	Low	Low
					Chang*	2013	Moderate	Moderate	Low	Low	Low	Low
Yin X	2016	NLR	Prostate Castration Resistant	OS	Yao	2015	Low	Moderate	Low	Low	Low	Low
					Nakagami	2015	High	High	Low	Moderate	Moderate	Moderate
					MacLanchlan	2015	Low	Moderate	Low	Low	Low	Moderate
					Lorente	2015	High	Moderate	Low	Low	Moderate	Moderate
					Templeton*	2014	Low	Low	Low	Low	Low	Low
					Templeton*	2014	Low	Low	Low	Low	Low	Low
					Sonpavde	2014	Low	Moderate	Low	Low	Low	Low
					Nuhn	2014	Low	Low	Low	Low	Low	Low
					Linton	2013	Low	Low	Low	Low	Low	Low
Suh J	2019	NLR	Bladder (UTR)	RFS	Ozyalvacli	2015	Low	High	Low	Moderate	High	Moderate
					Mano*	2015	Low	Moderate	Low	Low	Low	Low
					Favilla*	2016	Low	Low	Low	Low	Low	Low
					Ogihara*	2016	Low	Moderate	Low	Low	Low	Low
					Camtosun	2017	Moderate	High	Low	Low	Low	Low
Ni L	2020	NLR	Endometrial	OS	Aoyama	2019	Low	Moderate	Low	Low	Low	Low
					Comert	2018	Low	Low	Low	Low	Moderate	Low
					Cummings	2015	Low	Moderate	Low	Low	Low	Low
					Ding	2017	Low	Low	Low	Low	Moderate	Low
					Eo	2016	Low	Low	Low	Low	Low	Low

					Haruma	2015	Low	Moderate	Low	Low	Low	Low
					Li	2015	Low	Moderate	Low	Low	Low	Low
					Matsuo	2015	Low	Low	Low	Moderate	Low	Low
					Takahashi	2015	Low	Low	Moderate	Low	Low	Low
Yang S	2019	NLR	Nasopharyngeal	OS	Jin	2015	Low	Low	Low	Low	Moderate	Low
					An*	2011	Low	High	Low	Low	Low	Low
					Li	2017	Low	Low	Low	Low	Moderate	Low
					Sun*	2016	Low	Moderate	Low	Low	Low	Low
					Chua	2016	Moderate	Low	Low	Low	Low	Low
					He*	2012	Low	Moderate	Moderate	Low	Low	Low
					Li	2016	Moderate	Moderate	Low	Low	Low	Low
					Chang*	2013	Moderate	Moderate	Low	Low	Low	Low
					Chen*	2014	Low	Moderate	Low	Low	Low	Low
					Lu	2017	Moderate	Low	Low	Low	Low	Low
Li LQ	2020	NLR	STS	DFS	Chan	2018	Low	Low	Low	Low	Low	Low
					Chen	2019	Low	Low	Moderate	Low	Low	Low
					Cheng	2019	Low	Low	Low	Low	Low	Low
					Idowu	2012	High	Low	Low	Moderate	Low	Low
					Jiang	2015	Moderate	Low	Low	Low	Low	Low
					Szkandera*	2014	Low	Moderate	Low	Low	Low	Low
					Szkandera*	2014	Low	Moderate	Low	Low	Low	Low

Modified from: Hayden JA, Côté P, Bombardier C. Evaluation of the Quality of Prognosis Studies in Systematic Reviews. Annals of Internal Medicine. 2006;144:427-437, with the assistance of the QUIPS-LBP Working Group.

Ratings:
High bias: The relationship between the PF and outcome is very likely to be different for participants and eligible nonparticipants
Moderate bias: The relationship between the PF and outcome may be different for participants and eligible nonparticipants
Low bias: The relationship between the PF and outcome is unlikely to be different for participants and eligible nonparticipants

Appendix O: Sensitivity analyses of overall survival in rectal cancer

Appendix O Figure 1 - NLR: Overall survival in rectal cancer

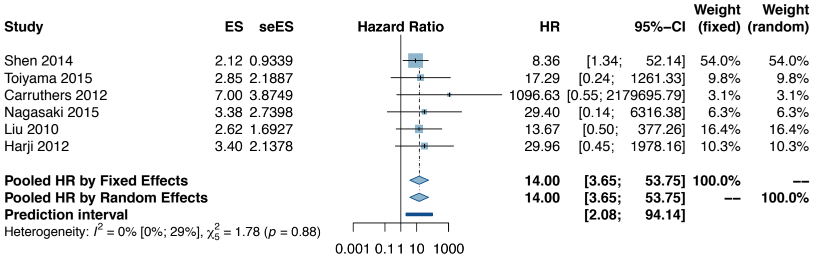

Association between elevated NLR and OS in rectal cancer,
as reported by Dong et al. 2016. Weightings and summary effect estimates are pictured for both fixed and random effects. (ES) Effect Size, (seES) Standard error of Effect Size, (HR) Hazard ratio.

Appendix O Figure 2 - NLR: Overall survival in rectal cancer with Carruthers et al. 2012 removed

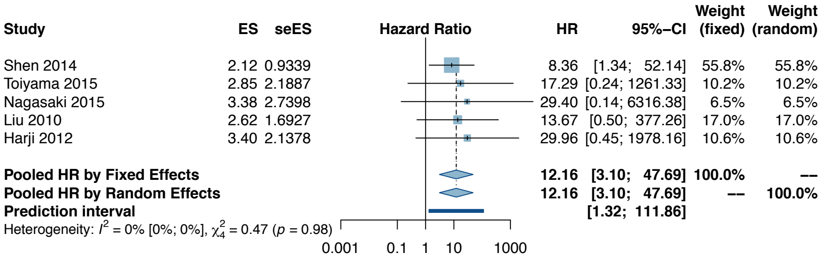

Association between elevated NLR and OS in rectal cancer,
with point estimate from Carruthers et al. 2012 removed. Weightings and summary effect estimates are pictured for both fixed and random effects. (ES) Effect Size, (seES) Standard error of Effect Size, (HR) Hazard ratio.

The association between elevated NLR and OS in rectal cancer (Dong et al 2016) represented an outlier among all included meta-analyses, with an HR of 14·00 [95% CI 3·65-53·75] through the random effects model. Within this meta-analysis, the point estimate by Carruthers et al. 2012 reported an HR of 1096·63 [95% CI 0·55-2179695·79]. This point estimate was removed for sensitivity analysis and resulted in a new HR of 12·16 [95% CI 3·10-47·69] through the random effects model. This 13% difference in HR suggests that the effect size estimate reported by Dong et al. 2014 is not overly sensitive to the largest reported effect size of its included studies.
