# Supplementary material for: Neutrophil to lymphocyte ratio and cancer prognosis: an umbrella review of systematic reviews and meta-analyses of observational studies
Source: BMC Med. 2020 Nov 20;18:360. doi: 10.1186/s12916-020-01817-1 (PMC7678319; doi:10.1186/s12916-020-01817-1)
Supplement: Supplementary file 2 — Additional file 2. Supplementary Figs. 1–205. [file 12916_2020_1817_MOESM2_ESM.pdf]

Supplementary Figures 1 - 205

Supplementary Figure 1 - Grading criteria for evidence

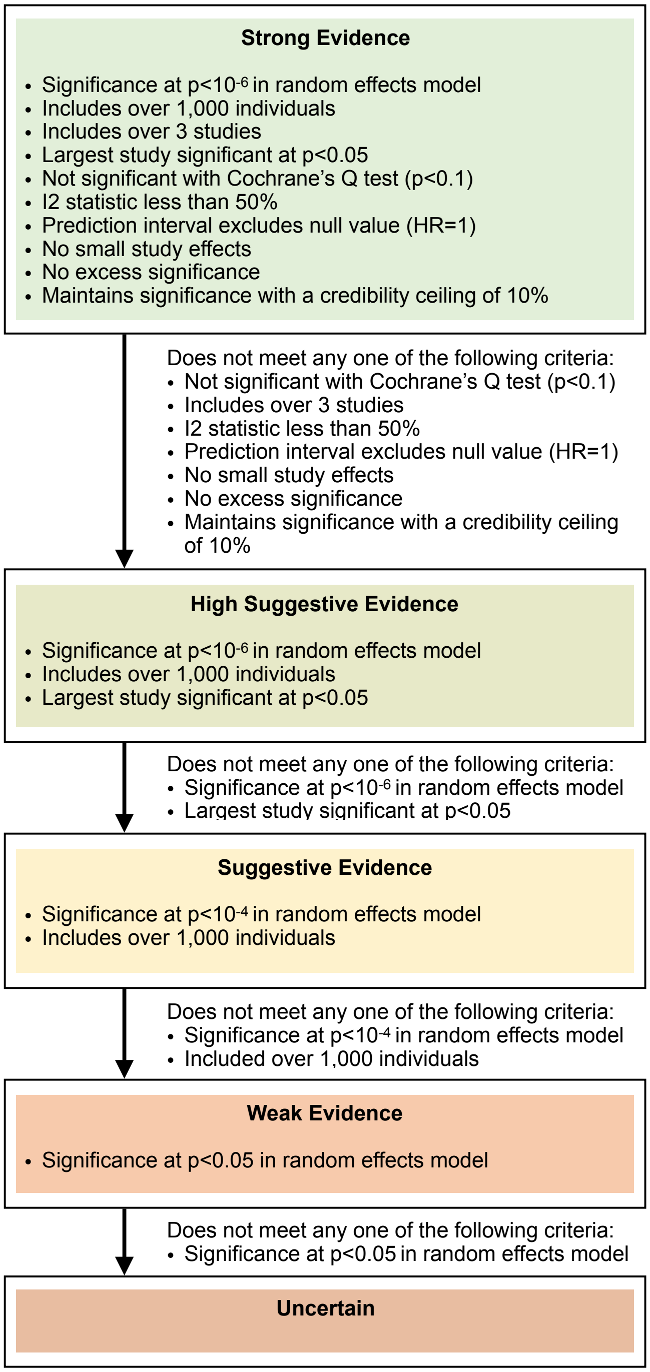

Supplementary Figure 1. Grading criteria for evidence. Associations were categorised according to pre-defined Grading of Recommendations, Assessment, Development and Evaluations (GRADE) based on previously published umbrella review studies (Kyrgiou et al., 2017; Theodoratou et al., 2014; Tsilidis et al., 2015). We additionally required studies to have 3 or more component studies due to the prevalence of associations with very small sample sizes and a new category "not significant" for those associations which were not backed by a significant effect estimate and could not be classified as "weak".

Supplementary Figure 2 - NLR: OS in Composite endpoints

Study	ES	seES	HR	95%–CI	Weight (fixed)	Weight (random)
Aiao 2014	0.64	0.1240	1.89	[1.48; 2.41]	0.4%	2.0%
Alagappan 2016	0.41	0.1779	1.51	[1.07; 2.14]	0.2%	1.7%
An 2010	1.50	0.6048	4.49	[1.37; 14.69]	0.0%	0.3%
Berardi 2016	0.55	0.1662	1.74	[1.26; 2.41]	0.2%	1.7%
Cedres 2012	0.00	0.0101	1.00	[0.98; 1.02]	66.2%	2.6%
Cetin 2013	0.88	0.3048	2.41	[1.33; 4.38]	0.1%	1.0%
Chen 2012	1.28	0.4689	3.59	[1.43; 9.00]	0.0%	0.5%
Cho 2014	0.45	0.1261	1.57	[1.23; 2.01]	0.4%	2.0%
Choi 2016	1.08	0.1498	2.93	[2.18; 3.93]	0.3%	1.9%
Chrom 2016	0.77	0.1909	2.16	[1.49; 3.14]	0.2%	1.6%
Chua 2011	0.53	0.1865	1.70	[1.18; 2.45]	0.2%	1.6%
de Fonseca 2014	0.69	0.2269	2.00	[1.28; 3.12]	0.1%	1.4%
Del Prete 2015	0.53	0.2303	1.70	[1.08; 2.67]	0.1%	1.3%
Fox 2013	0.35	0.1322	1.42	[1.10; 1.84]	0.4%	2.0%
Fu 2016	0.27	0.1360	1.31	[1.00; 1.71]	0.4%	2.0%
Ghanim 2015	1.10	0.6802	3.00	[0.79; 11.38]	0.0%	0.3%
Giakoustidis 2015	0.75	0.2991	2.12	[1.18; 3.81]	0.1%	1.0%
Giordano 2015	1.22	0.2868	3.38	[1.93; 5.93]	0.1%	1.1%
Grenader 2015	0.48	0.1075	1.62	[1.31; 2.00]	0.6%	2.2%
Grenader 2016	0.51	0.0738	1.67	[1.45; 1.93]	1.2%	2.4%
Halazun 2008	0.82	0.1617	2.28	[1.66; 3.13]	0.3%	1.8%
He 2013	0.39	0.1795	1.47	[1.03; 2.09]	0.2%	1.7%
Huang 2011	1.20	0.4465	3.33	[1.39; 7.99]	0.0%	0.6%
Jafri 2013	0.56	0.1675	1.75	[1.26; 2.43]	0.2%	1.7%
Jeong 2012	0.50	0.2398	1.65	[1.03; 2.64]	0.1%	1.3%
Jin 2013	0.51	0.1367	1.66	[1.27; 2.17]	0.4%	2.0%
Kaneko 2012	1.48	0.4517	4.39	[1.81; 10.64]	0.0%	0.6%
Keizman (1) 2014	1.08	0.2783	2.95	[1.71; 5.09]	0.1%	1.1%
Keizman (2) 2014	0.81	0.3387	2.25	[1.16; 4.37]	0.1%	0.9%
Kim 2011	0.48	0.1788	1.62	[1.14; 2.30]	0.2%	1.7%
Kobayashi 2013	0.96	0.4828	2.62	[1.02; 6.75]	0.0%	0.5%
Kou 2016	0.93	0.1905	2.54	[1.75; 3.69]	0.2%	1.6%
Lee (1) 2012	0.48	0.2048	1.62	[1.08; 2.42]	0.2%	1.5%
Lee (2) 2012	0.05	0.0237	1.05	[1.00; 1.10]	12.0%	2.6%
Lee 2013	0.81	0.1422	2.24	[1.70; 2.96]	0.3%	1.9%
Linton 2013	0.17	0.2026	1.19	[0.80; 1.77]	0.2%	1.5%
Luo 2014	0.35	0.1066	1.42	[1.15; 1.75]	0.6%	2.2%
Malik 2007	0.53	0.1608	1.70	[1.24; 2.33]	0.3%	1.8%
Mangla 2014	0.17	0.0494	1.18	[1.07; 1.30]	2.8%	2.5%
McNamara 2014	0.23	0.1090	1.26	[1.02; 1.56]	0.6%	2.1%
Mohri 2014	0.83	0.2384	2.30	[1.44; 3.67]	0.1%	1.3%
Nagasaki 2015	1.34	0.4851	3.81	[1.47; 9.86]	0.0%	0.5%
Neal 2009	0.85	0.2739	2.35	[1.37; 4.02]	0.1%	1.1%
Nuhn 2013	0.63	0.2105	1.88	[1.24; 2.84]	0.2%	1.5%
Yoshio 2014	1.23	0.3923	3.43	[1.59; 7.40]	0.0%	0.7%
Peng 2014	0.65	0.2104	1.92	[1.27; 2.90]	0.2%	1.5%
Piciucchi 2014	0.13	0.0551	1.14	[1.02; 1.27]	2.2%	2.5%
Pinato 2014	0.69	0.3724	2.00	[0.96; 4.15]	0.0%	0.7%
Rossi 2015	0.55	0.1401	1.74	[1.32; 2.29]	0.3%	1.9%
Song 2015	0.87	0.2004	2.39	[1.61; 3.54]	0.2%	1.5%
Sonpavde 2014	0.44	0.0847	1.55	[1.31; 1.83]	0.9%	2.3%
Taguchi 2015	0.40	0.1942	1.49	[1.02; 2.18]	0.2%	1.6%
Tan 2015	0.12	0.3376	1.13	[0.58; 2.19]	0.1%	0.9%
Tang 2016	0.69	0.3010	1.99	[1.10; 3.59]	0.1%	1.0%
Templeton 2014	0.41	0.1907	1.50	[1.03; 2.18]	0.2%	1.6%
Templeton 2016	0.52	0.0730	1.69	[1.46; 1.95]	1.3%	2.4%
Tohme 2015	0.66	0.2401	1.93	[1.21; 3.09]	0.1%	1.3%
Turner 2015	−0.22	0.1896	0.80	[0.55; 1.16]	0.2%	1.6%
Van Soest 2015	0.25	0.0770	1.29	[1.11; 1.50]	1.1%	2.4%
Vivaldi 2016	0.89	0.2864	2.43	[1.39; 4.26]	0.1%	1.1%
Wang 2011	0.30	0.1206	1.35	[1.07; 1.71]	0.5%	2.1%
Wu 2015	0.58	0.2201	1.78	[1.16; 2.74]	0.1%	1.4%
Yamanaka 2007	0.42	0.0719	1.52	[1.32; 1.75]	1.3%	2.4%
Zeng 2016	0.41	0.1920	1.51	[1.04; 2.20]	0.2%	1.6%
Zhang 2016	0.33	0.1568	1.39	[1.02; 1.89]	0.3%	1.8%
**Pooled HR by Fixed Effects**			**1.10**	**[1.08; 1.12]**	**100.0%**	**--**
**Pooled HR by Random Effects**			**1.68**	**[1.56; 1.82]**	**--**	**100.0%**
**Prediction interval**				**[1.04; 2.72]**		

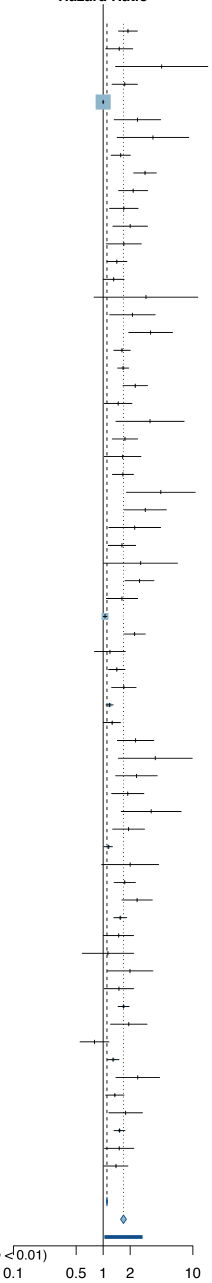

Heterogeneity: $I^2 = 90\%$ [88%; 92%], $\chi^2_{64} = 627.98$ ($p < 0.01$)

Tau²= 0.236

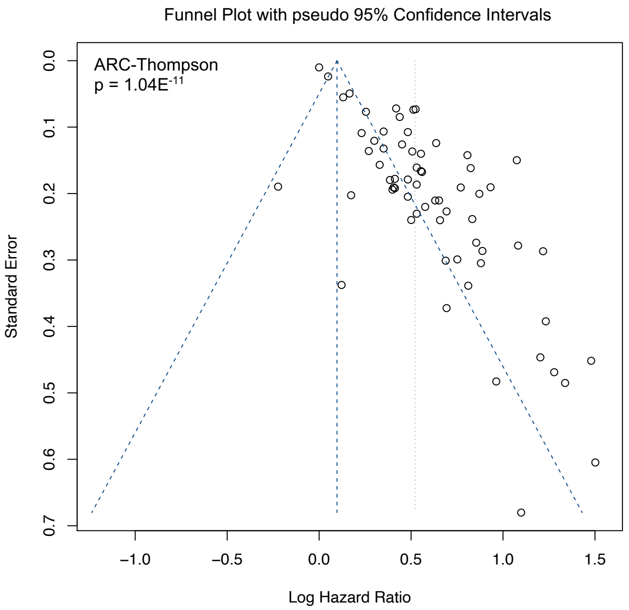

All funnel plots within Appendix 5 display the standard error of the individual studies on the Y-axis labelled "Standard Error" and the log(HR) from the random effects estimate on the X-axis labelled "Log Hazard Ratio". The pseudo 95% CI are centred around the fixed effects summary effect estimate.

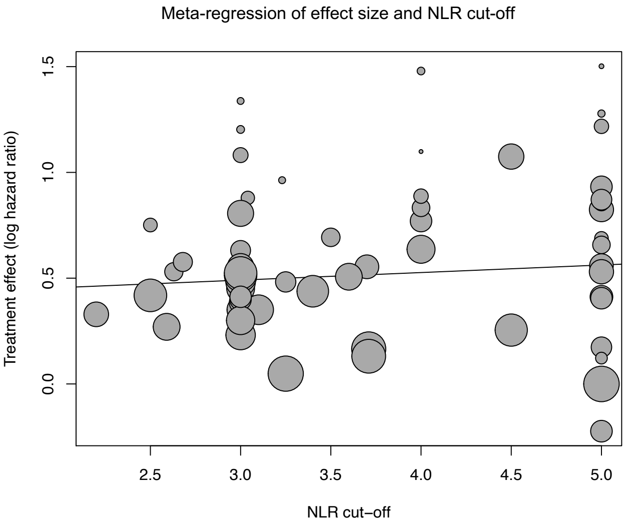

All forest plots display both the weightings and summary effect size estimates for both fixed and random effects models. (ES) Effect Size, (seES) Standard error of Effect Size.

Supplementary Figure 3 - NLR: DFSin Composite endpoints (solid tumours)

Study	TE	seTE	HR	95%–CI	Weight (fixed)	Weight (random)
**Adjustment = Multivariate**						
Caruthers 2012	1.41	0.1993	4.10	[2.77; 6.06]	0.1%	4.9%
Chiang (1) 2012	0.27	0.0077	1.31	[1.29; 1.33]	50.2%	5.9%
Chiang (2) 2012	0.18	0.0084	1.20	[1.18; 1.22]	42.2%	5.9%
Ding 2010	1.59	0.2795	4.88	[2.82; 8.44]	0.0%	4.2%
Gomez 2008	0.95	0.1722	2.59	[1.85; 3.63]	0.1%	5.1%
Guo 2011	1.30	0.1330	3.66	[2.82; 4.75]	0.2%	5.4%
Halazun 2007	1.51	0.0945	4.52	[3.76; 5.44]	0.3%	5.6%
Halazun 2009	3.00	1.1347	19.99	[2.16; 184.77]	0.0%	0.8%
Hung 2011	0.22	0.0357	1.24	[1.16; 1.33]	2.3%	5.8%
Jung 2011	0.50	0.0472	1.65	[1.50; 1.81]	1.3%	5.8%
Mallapa 2012	0.59	0.0734	1.81	[1.57; 2.09]	0.6%	5.7%
Ohno 2010	0.77	0.1195	2.16	[1.71; 2.73]	0.2%	5.5%
Ohno 2012	0.13	0.1121	1.14	[0.92; 1.42]	0.2%	5.5%
Perez 2012	1.19	0.1661	3.30	[2.38; 4.57]	0.1%	5.1%
Pichler 2013	0.33	0.2525	1.39	[0.85; 2.28]	0.0%	4.4%
Sharaiha 2011	0.82	0.0535	2.26	[2.03; 2.51]	1.0%	5.8%
Szkandera 2013	0.68	0.3217	1.98	[1.05; 3.72]	0.0%	3.8%
Wang 2011	0.57	0.1253	1.76	[1.38; 2.25]	0.2%	5.4%
**Pooled HR by Fixed Effects**			**1.29**	**[1.27; 1.30]**	**99.2%**	**--**
**Pooled HR by Random Effects**			**2.16**	**[1.71; 2.72]**	**--**	**90.4%**
Heterogeneity: $I^2 = 97\%$ [96%; 98%], $\chi^2_{17} = 612.55$ ($p < 0.01$)						
**Adjustment = Univariate**						
Leitch 2007	0.40	0.3108	1.49	[0.81; 2.74]	0.0%	3.9%
Neal 2008	0.67	0.0639	1.95	[1.72; 2.21]	0.7%	5.7%
**Pooled HR by Fixed Effects**			**1.93**	**[1.71; 2.18]**	**0.8%**	**--**
**Pooled HR by Random Effects**			**1.93**	**[1.71; 2.18]**	**--**	**9.6%**
Heterogeneity: $I^2 = 0\%$, $\chi^2_1 = 0.72$ ($p = 0.40$)						
**Pooled HR by Fixed Effects**			**1.29**	**[1.28; 1.31]**	**100.0%**	**--**
**Pooled HR by Random Effects**			**2.11**	**[1.71; 2.60]**	**--**	**100.0%**
**Prediction interval**				**[0.81; 5.47]**		
Heterogeneity: $I^2 = 97\%$ [96%; 98%], $\chi^2_{19} = 654.72$ ($p < 0.01$)						
Tau²= 0.441						

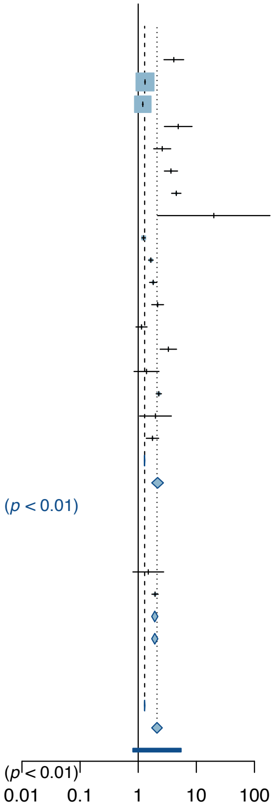

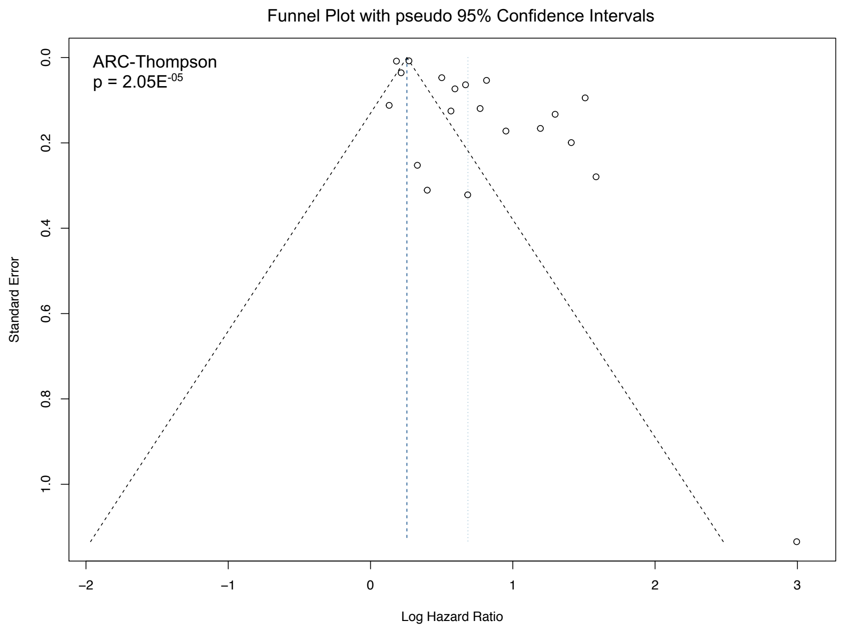

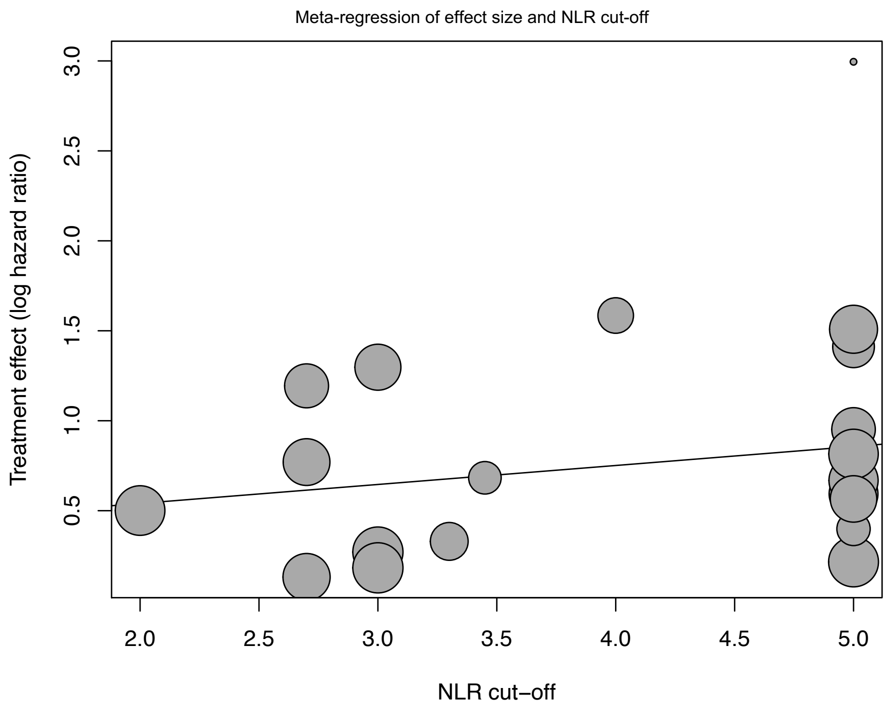

Supplementary Figure 4 - NLR: PFS in Composite endpoints

Study	ES	seES	HR	95%–CI	Weight (fixed)	Weight (random)
Berardi	0.31	0.1344	1.36	[1.04; 1.77]	1.7%	5.4%
Botta	0.69	0.3039	1.99	[1.10; 3.61]	0.3%	2.8%
Botta	0.51	0.2618	1.67	[1.00; 2.79]	0.4%	3.3%
Cetin	0.10	0.0355	1.11	[1.04; 1.19]	23.8%	6.7%
Cho	0.39	0.1248	1.48	[1.16; 1.89]	1.9%	5.5%
Chua	0.47	0.1762	1.60	[1.13; 2.26]	1.0%	4.6%
Giordano	1.17	0.3217	3.21	[1.71; 6.03]	0.3%	2.6%
Grenader	0.34	0.1080	1.40	[1.13; 1.73]	2.6%	5.8%
Grenader	0.55	0.0424	1.73	[1.59; 1.88]	16.7%	6.7%
Halazun	1.51	0.3076	4.52	[2.47; 8.26]	0.3%	2.8%
He	0.41	0.1634	1.51	[1.10; 2.08]	1.1%	4.8%
Huang	0.97	0.3708	2.63	[1.27; 5.44]	0.2%	2.2%
Jafri	0.54	0.1636	1.72	[1.25; 2.37]	1.1%	4.8%
Kaneko	0.18	0.4726	1.20	[0.48; 3.03]	0.1%	1.5%
Keizman	1.04	0.2664	2.83	[1.68; 4.77]	0.4%	3.3%
Keizman	1.26	0.3215	3.52	[1.87; 6.61]	0.3%	2.6%
Lee	0.02	0.0292	1.02	[0.96; 1.08]	35.3%	6.8%
Pinato	0.59	0.3536	1.80	[0.90; 3.60]	0.2%	2.3%
Rossi	0.41	0.1331	1.51	[1.16; 1.96]	1.7%	5.4%
Templeton	0.26	0.0627	1.30	[1.15; 1.47]	7.6%	6.5%
Wu	0.43	0.2135	1.54	[1.01; 2.34]	0.7%	4.0%
Zeng	0.58	0.1982	1.79	[1.21; 2.64]	0.8%	4.3%
Zhang	0.43	0.1435	1.54	[1.16; 2.04]	1.5%	5.2%
**Pooled HR by Fixed Effects**			**1.25**	**[1.21; 1.30]**	**100.0%**	**--**
**Pooled HR by Random Effects**			**1.63**	**[1.43; 1.86]**	**--**	**100.0%**
**Prediction interval**				**[0.94; 2.81]**		

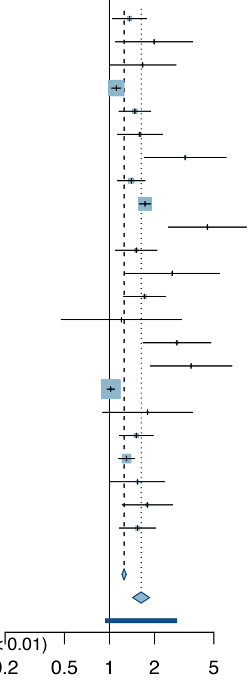

Heterogeneity: I^2 = 89% [84%; 92%], χ^2_{22} = 192.17 (p < 0.01)

Tau²= 0.254

Funnel Plot with pseudo 95% Confidence Intervals

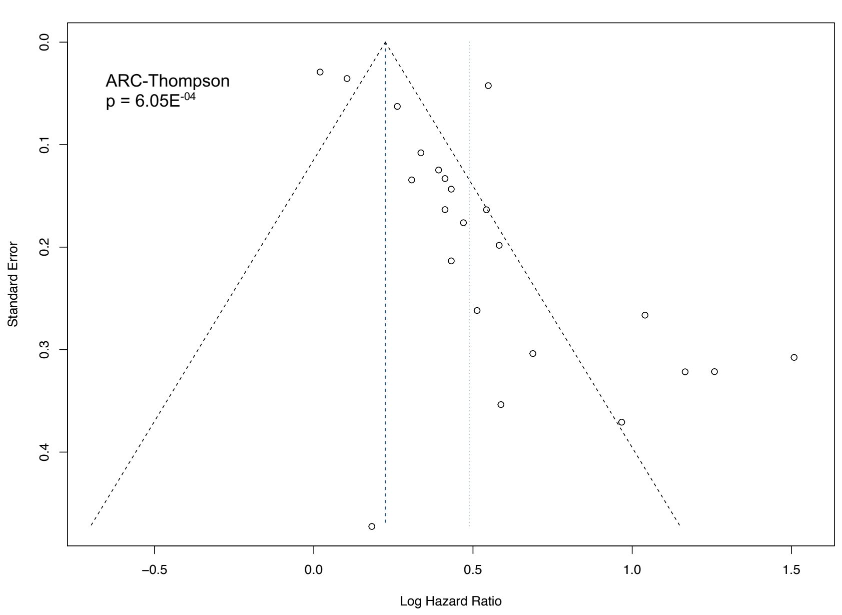

Meta-regression of effect size and NLR cut-off

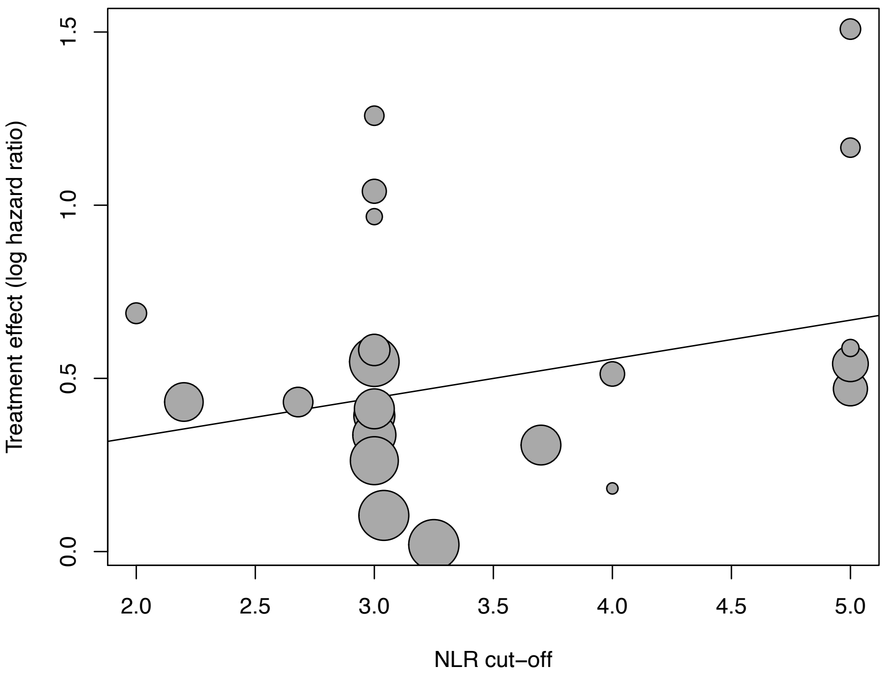

Supplementary Figure 5 - IN: CSS in Composite endpoints

Study	ES	seES	HR	95%-CI	Weight (fixed)	Weight (random)
Jensen 2009	1.25	0.3079	3.50	[1.91; 6.40]	62.6%	62.6%
Jenson 2011	1.14	0.3981	3.13	[1.43; 6.83]	37.4%	37.4%
**Pooled HR by Fixed Effects**			**3.36**	**[2.08; 5.41]**	**100.0%**	**--**
**Pooled HR by Random Effects**			**3.36**	**[2.08; 5.41]**	**--**	**100.0%**

Heterogeneity: $I^2 = 0\%$, $\chi^2_1 = 0.05$ ($p = 0.82$)

Tau²= 0

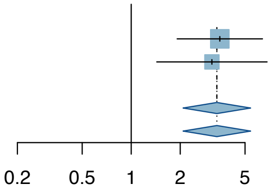

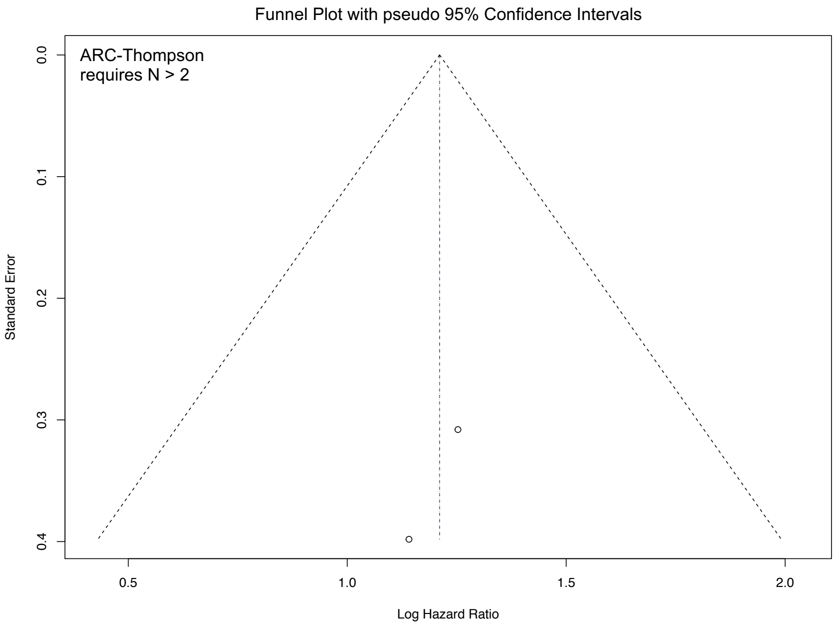

Supplementary Figure 6 – IN: OS in Composite endpoints

Study	ES	seES	HR	95%-CI	Weight (fixed)	Weight (random)
Caruso 2002	−0.24	0.1871	0.79	[0.55; 1.14]	6.8%	7.3%
Donskov 2006	0.83	0.2694	2.30	[1.36; 3.90]	3.3%	5.8%
Jensen 2009	1.13	0.2439	3.10	[1.92; 5.00]	4.0%	6.2%
Trellakis 2010	0.96	0.4848	2.61	[1.01; 6.75]	1.0%	3.0%
Kuang 2010	0.17	0.1997	1.19	[0.80; 1.76]	5.9%	7.1%
Li 2011	0.95	0.2376	2.58	[1.62; 4.11]	4.2%	6.3%
Gu 2012	0.57	0.2088	1.76	[1.17; 2.65]	5.4%	6.9%
Ilie 2012	0.15	0.0886	1.16	[0.98; 1.38]	30.1%	9.2%
Jensen 2012	0.88	0.3186	2.41	[1.29; 4.50]	2.3%	4.9%
Rao 2012	0.71	0.2769	2.04	[1.19; 3.51]	3.1%	5.6%
Zhao 2012	0.66	0.3011	1.94	[1.08; 3.50]	2.6%	5.2%
Zhou 2012	0.69	0.1625	2.00	[1.45; 2.75]	9.0%	7.8%
Carus 2013	0.16	0.1468	1.17	[0.88; 1.56]	11.0%	8.1%
Dumitru (1) 2013	0.22	0.3262	1.24	[0.65; 2.35]	2.2%	4.8%
Dumitru (2) 2013	0.68	0.3812	1.98	[0.94; 4.18]	1.6%	4.1%
Ino 2013	0.40	0.1775	1.49	[1.05; 2.11]	7.5%	7.5%
**Pooled HR by Fixed Effects**			**1.46**	**[1.32; 1.60]**	**100.0%**	**--**
**Pooled HR by Random Effects**			**1.66**	**[1.37; 2.02]**	**--**	**100.0%**
**Prediction interval**				**[0.81; 3.40]**		

Heterogeneity: $I^2 = 70\%$ [51%; 82%], $\chi^2_{15} = 50.67$ ($p < 0.01$)

Tau²= 0.318

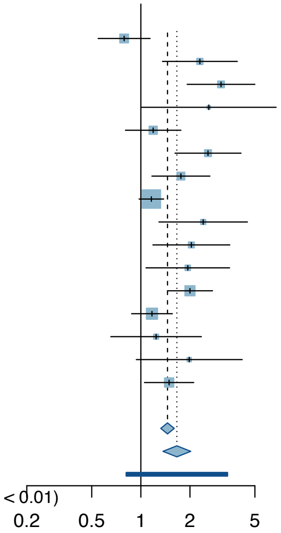

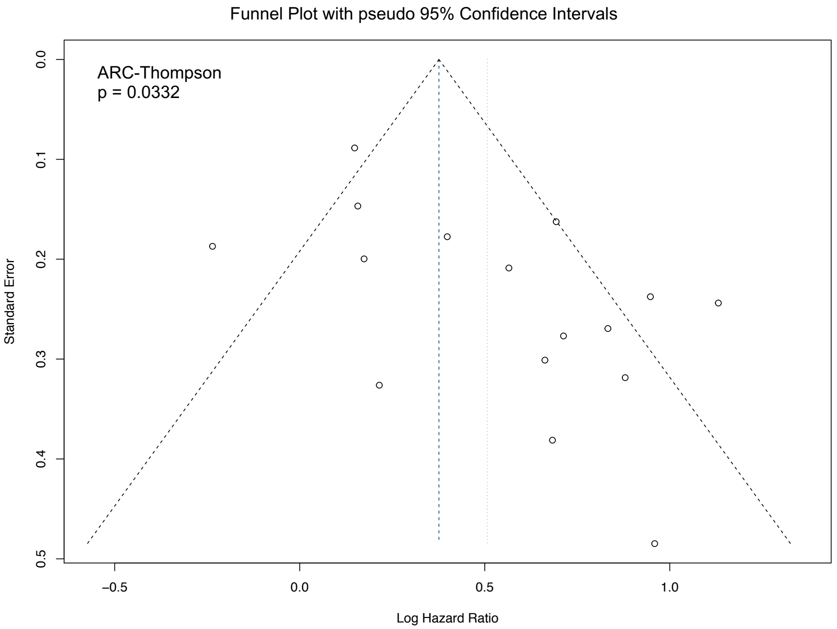

Supplementary Figure 7 – PN: OS in Composite endpoints

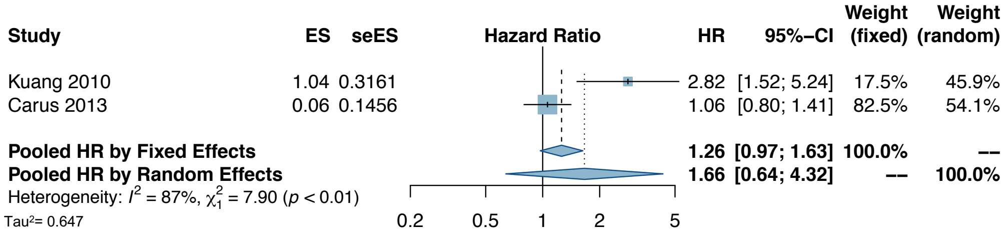

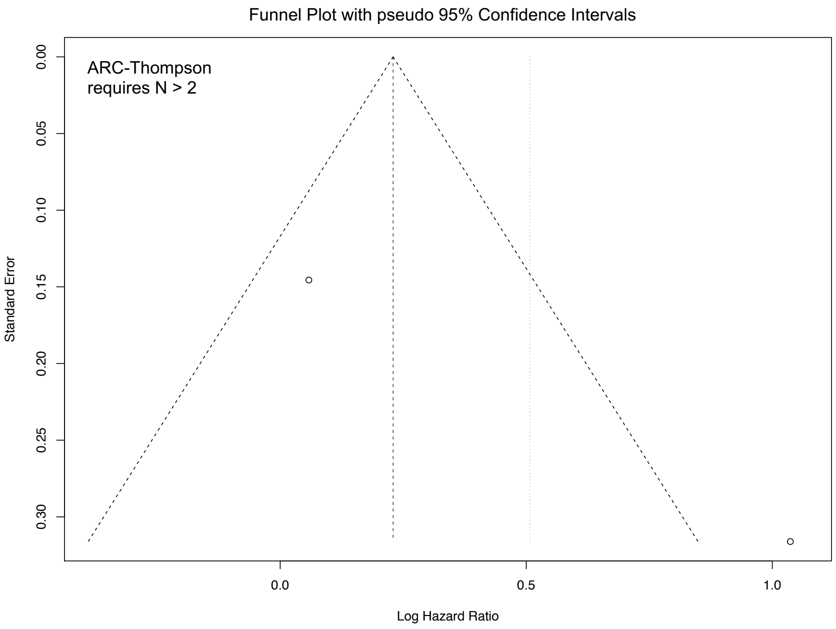

Supplementary Figure 8 – SN: OS in Composite endpoints

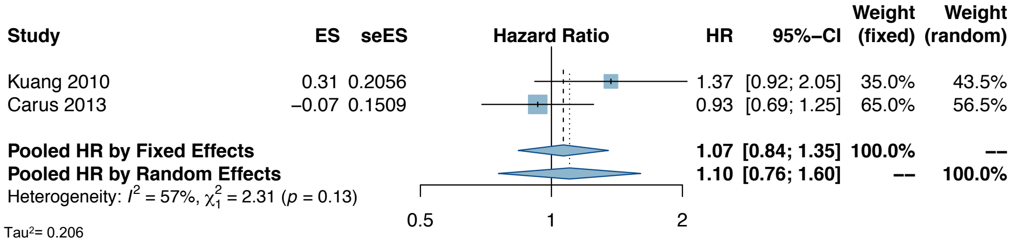

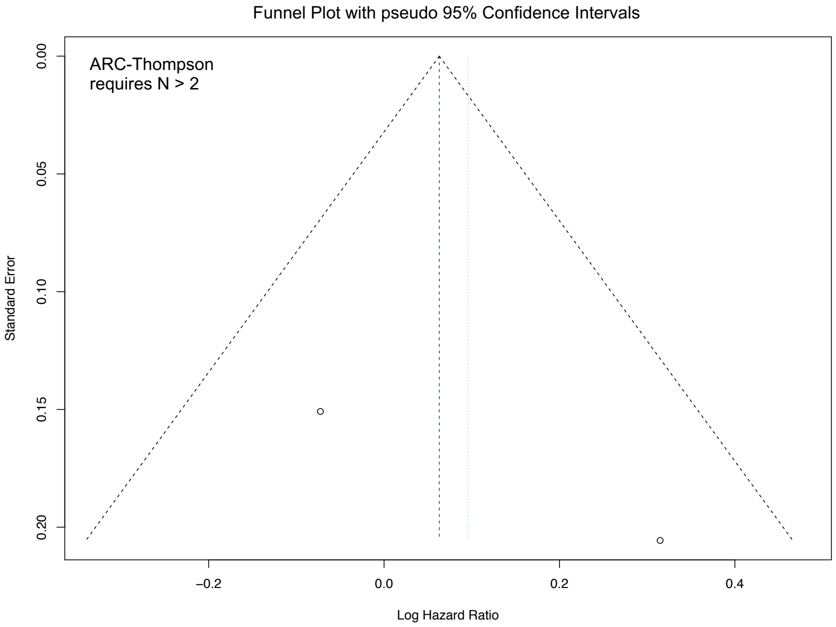

Supplementary Figure 9 - NLR: OS in all operable cancers

Study	TE	seTE	Hazard Ratio	HR	95%–CI	Weight (fixed)	Weight (random)
**Adjustment = Multivariate**							
Halazun 2008	0.82	0.1617		2.28	[1.66; 3.13]	0.1%	1.1%
Halazun 2009	1.81	0.5015		6.10	[2.28; 16.30]	0.0%	0.3%
Cho 2009	2.13	1.0422		8.42	[1.09; 64.93]	0.0%	0.1%
Sarraf 2009	0.10	0.0315		1.10	[1.03; 1.17]	1.8%	1.5%
Kishi 2009	0.69	0.3407		2.00	[1.03; 3.90]	0.0%	0.6%
Shimada 2010	0.61	0.2050		1.84	[1.23; 2.75]	0.0%	0.9%
Mohri 2010	1.02	0.2273		2.78	[1.78; 4.34]	0.0%	0.9%
Bhatti 2010	0.19	0.0923		1.21	[1.01; 1.45]	0.2%	1.3%
Liu 2010	0.96	0.4187		2.61	[1.15; 5.93]	0.0%	0.4%
Kao 2011	0.58	0.2752		1.79	[1.04; 3.07]	0.0%	0.7%
Sharaiha 2011	0.84	0.2112		2.32	[1.53; 3.51]	0.0%	0.9%
Jung 2011	0.38	0.1781		1.46	[1.03; 2.07]	0.1%	1.0%
Miyata 2011	0.26	0.2730		1.30	[0.76; 2.22]	0.0%	0.7%
Wang 2011	0.97	0.3198		2.65	[1.42; 4.96]	0.0%	0.6%
Hung 2011	0.25	0.1317		1.29	[1.00; 1.67]	0.1%	1.2%
Asher 2011	−0.14	0.2571		0.87	[0.53; 1.44]	0.0%	0.8%
Carruthers 2012	1.95	0.5100		7.00	[2.58; 19.02]	0.0%	0.3%
Kwon 2012	0.42	0.4635		1.52	[0.61; 3.77]	0.0%	0.4%
Wang 2012	0.63	0.3711		1.87	[0.90; 3.87]	0.0%	0.5%
Idowu 2012	1.63	0.7225		5.12	[1.24; 21.10]	0.0%	0.2%
Toiyama 2013	−0.02	0.4939		0.98	[0.37; 2.58]	0.0%	0.3%
Szkandera 2013	0.59	0.2790		1.80	[1.04; 3.11]	0.0%	0.7%
Bambury 2013	0.59	0.2612		1.81	[1.08; 3.02]	0.0%	0.8%
Mano 2013	1.32	0.0148		3.74	[3.63; 3.85]	8.0%	1.5%
Jankova 2013	0.06	0.0281		1.06	[1.00; 1.12]	2.2%	1.5%
Pichler 2013	0.46	0.1884		1.59	[1.10; 2.30]	0.0%	1.0%
Forget (c) 2013	0.51	0.2637		1.67	[1.00; 2.80]	0.0%	0.8%
Absenger 2013	0.52	0.2496		1.68	[1.03; 2.74]	0.0%	0.8%
Fu 2013	0.36	0.1634		1.43	[1.04; 1.97]	0.1%	1.1%
Feng 2013	0.27	0.1389		1.31	[1.00; 1.72]	0.1%	1.2%
Wang 2013	4.93	1.5390		138.52	[6.78; 2828.01]	0.0%	0.0%
Son 2013	0.61	0.6961		1.84	[0.47; 7.20]	0.0%	0.2%
Liao 2013	0.00	0.4655		1.00	[0.40; 2.49]	0.0%	0.4%
Yuan 2014	0.94	0.1645		2.55	[1.85; 3.52]	0.1%	1.1%
Koh 2014	3.21	1.0670		24.88	[3.07; 201.39]	0.0%	0.1%
Ying 2014	1.00	0.2294		2.73	[1.74; 4.28]	0.0%	0.9%
Hermanns 2014	0.51	0.1829		1.67	[1.17; 2.39]	0.1%	1.0%
East 2014	0.36	0.1530		1.43	[1.06; 1.93]	0.1%	1.1%
Viers 2014	0.02	0.0050		1.02	[1.01; 1.03]	70.3%	1.5%
Jiang 2014	0.47	0.2153		1.60	[1.05; 2.44]	0.0%	0.9%
Feng 2014	0.36	0.1088		1.43	[1.16; 1.77]	0.1%	1.3%
Ozdemir 2014	1.20	0.3348		3.31	[1.72; 6.38]	0.0%	0.6%
Szkandera 2014	1.04	0.3713		2.84	[1.37; 5.88]	0.0%	0.5%
Dalpiaz 2014	0.91	0.3262		2.48	[1.31; 4.70]	0.0%	0.6%
Pinato 2014	1.34	0.4382		3.80	[1.61; 8.97]	0.0%	0.4%
McNamara 2014	0.14	0.1457		1.15	[0.86; 1.53]	0.1%	1.1%
Viers 2014	0.03	0.0146		1.03	[1.00; 1.06]	8.1%	1.5%
Neofytou 2014	0.42	0.3435		1.52	[0.78; 2.98]	0.0%	0.6%
Zhang 2014	0.73	0.2308		2.08	[1.32; 3.27]	0.0%	0.9%
Aurello 2014	0.41	0.3973		1.51	[0.69; 3.29]	0.0%	0.5%
Shen 2014	0.75	0.3175		2.12	[1.14; 3.95]	0.0%	0.6%
Dalpiaz (a) 2014	0.19	0.0521		1.21	[1.09; 1.34]	0.6%	1.4%
Cummings 2015	0.60	0.1859		1.82	[1.26; 2.62]	0.1%	1.0%
Tu 2015	0.78	0.2994		2.18	[1.21; 3.92]	0.0%	0.7%
Han 2015	0.05	0.0681		1.05	[0.92; 1.20]	0.4%	1.4%
Shimizu 2015	0.47	0.2277		1.60	[1.02; 2.50]	0.0%	0.9%
Hsu 2015	0.45	0.1336		1.57	[1.21; 2.04]	0.1%	1.2%
Que 2015	0.06	0.3632		1.06	[0.52; 2.16]	0.0%	0.5%
Han 2015	0.10	0.2161		1.10	[0.72; 1.68]	0.0%	0.9%
Pine 2015	0.60	0.1681		1.82	[1.31; 2.53]	0.1%	1.1%
Zhang 2015	0.48	0.1126		1.62	[1.30; 2.02]	0.1%	1.3%
Chen 2015	0.47	0.1555		1.60	[1.18; 2.17]	0.1%	1.1%
Graziosi 2015	0.53	0.2618		1.70	[1.02; 2.84]	0.0%	0.8%
Deng 2015	0.12	0.2570		1.13	[0.68; 1.87]	0.0%	0.8%
Kim 2015	0.34	0.1504		1.40	[1.04; 1.88]	0.1%	1.1%
Ben 2015	0.41	0.1408		1.51	[1.15; 1.99]	0.1%	1.2%
Takahashi 2015	0.76	0.2525		2.14	[1.30; 3.51]	0.0%	0.8%
Neal 2015	0.57	0.1554		1.77	[1.31; 2.40]	0.1%	1.1%
Li 2015	1.06	0.1907		2.89	[1.99; 4.20]	0.0%	1.0%
Li 2015	0.83	0.6218		2.30	[0.68; 7.78]	0.0%	0.2%
Szkandera 2015	0.47	0.2069		1.60	[1.07; 2.40]	0.0%	0.9%
Chen 2015	0.34	0.1691		1.40	[1.01; 1.95]	0.1%	1.1%
Choi 2015	0.52	0.1415		1.69	[1.28; 2.23]	0.1%	1.2%
Yu 2015	0.47	0.1578		1.60	[1.17; 2.18]	0.1%	1.1%
Liao 2015	1.10	0.3131		3.01	[1.63; 5.56]	0.0%	0.6%
Shibutani 2015	1.89	0.9999		6.62	[0.93; 46.99]	0.0%	0.1%
Song 2015	0.86	0.2917		2.36	[1.33; 4.18]	0.0%	0.7%
Okamura 2015	0.88	0.2610		2.41	[1.44; 4.02]	0.0%	0.8%
Sun 2015	0.06	0.1196		1.06	[0.84; 1.34]	0.1%	1.2%
Choi 2015	0.65	0.2131		1.91	[1.26; 2.90]	0.0%	0.9%
Wen 2015	0.51	0.2139		1.67	[1.10; 2.54]	0.0%	0.9%
Wang 2015	1.59	0.5079		4.90	[1.81; 13.26]	0.0%	0.3%
Qu 2015	0.32	0.1241		1.38	[1.08; 1.76]	0.1%	1.2%
Spolverato 2015	0.66	0.3225		1.94	[1.03; 3.65]	0.0%	0.6%
Kosumi 2016	0.61	0.2339		1.84	[1.16; 2.91]	0.0%	0.8%
Mohri 2016	0.74	0.3248		2.09	[1.11; 3.95]	0.0%	0.6%
Wang 2016	1.21	0.4512		3.37	[1.39; 8.16]	0.0%	0.4%
Li 2016	0.21	0.1013		1.23	[1.01; 1.50]	0.2%	1.3%
Toyokawa 2016	0.17	0.3295		1.19	[0.62; 2.27]	0.0%	0.6%
Fu 2016	0.27	0.1360		1.31	[1.00; 1.71]	0.1%	1.2%
Morizawa 2016	1.03	0.3445		2.80	[1.43; 5.50]	0.0%	0.6%
Ishizuka 2016	0.59	0.1983		1.81	[1.23; 2.67]	0.0%	1.0%
Ha 2016	0.25	0.3078		1.28	[0.70; 2.34]	0.0%	0.6%
Kawahara 2016	1.53	0.7037		4.62	[1.16; 18.35]	0.0%	0.2%
Kang 2016	0.12	0.0391		1.13	[1.05; 1.22]	1.1%	1.5%
Lu 2016	0.26	0.0930		1.30	[1.08; 1.56]	0.2%	1.3%
Takahashi 2016	0.60	0.2429		1.82	[1.13; 2.93]	0.0%	0.8%
Turner 2016	0.56	0.3566		1.75	[0.87; 3.52]	0.0%	0.5%
Cheng 2016	0.48	0.3037		1.61	[0.89; 2.92]	0.0%	0.7%
Bhindi 2016	0.44	0.1517		1.56	[1.16; 2.10]	0.1%	1.1%
**Pooled HR by Fixed Effects**				**1.16**	**[1.15; 1.17]**	**96.5%**	**---**
**Pooled HR by Random Effects**				**1.67**	**[1.55; 1.79]**	**---**	**82.8%**
Heterogeneity: $I^2 = 99\%$ [99%; 99%], $\chi^2_{99} = 7422.78$ ($p = 0$)							
**Adjustment = Univariate**							
Smith 2009	0.05	0.0284		1.05	[0.99; 1.11]	2.2%	1.5%
Jagdev 2010	0.74	0.1594		2.10	[1.54; 2.87]	0.1%	1.1%
Neal 2011	0.92	0.2403		2.51	[1.57; 4.02]	0.0%	0.8%
Forget (b) 2013	0.42	0.1793		1.52	[1.07; 2.16]	0.1%	1.0%
Forget (a) 2013	1.39	0.6500		4.00	[1.12; 14.30]	0.0%	0.2%
Linton 2014	−0.15	0.3853		0.86	[0.40; 1.83]	0.0%	0.5%
Wu 2014	0.57	0.2358		1.77	[1.11; 2.81]	0.0%	0.8%
Chan 2016	0.60	0.0893		1.83	[1.54; 2.18]	0.2%	1.3%
Chen 2015	0.86	0.3910		2.37	[1.10; 5.10]	0.0%	0.5%
Zhang 2015	0.43	0.0538		1.53	[1.38; 1.70]	0.6%	1.4%
Kawashima 2015	0.43	0.2168		1.53	[1.00; 2.34]	0.0%	0.9%
Xu 2015	0.41	0.2247		1.50	[0.97; 2.33]	0.0%	0.9%
Kadota 2015	0.60	0.1878		1.82	[1.26; 2.63]	0.0%	1.0%
Shirai 2015	−0.02	0.3351		0.98	[0.51; 1.89]	0.0%	0.6%
Takahashi 2015	0.90	0.2745		2.47	[1.44; 4.23]	0.0%	0.7%
Ishizuka 2014	0.69	0.1724		1.99	[1.42; 2.79]	0.1%	1.0%
Chan 2015	0.46	0.3373		1.59	[0.82; 3.08]	0.0%	0.6%
Hirahara 2015	0.15	0.3172		1.16	[0.62; 2.16]	0.0%	0.6%
Zhang 2015	0.77	0.1754		2.17	[1.54; 3.06]	0.1%	1.0%
Christina 2016	0.15	0.2930		1.16	[0.65; 2.06]	0.0%	0.7%
**Pooled HR by Fixed Effects**				**1.26**	**[1.20; 1.31]**	**3.5%**	**---**
**Pooled HR by Random Effects**				**1.64**	**[1.43; 1.87]**	**---**	**17.2%**
Heterogeneity: $I^2 = 85\%$ [79%; 90%], $\chi^2_{19} = 129.34$ ($p < 0.01$)							
**Pooled HR by Fixed Effects**				**1.17**	**[1.16; 1.18]**	**100.0%**	**---**
**Pooled HR by Random Effects**				**1.66**	**[1.55; 1.77]**	**---**	**100.0%**
**Prediction interval**					**[0.97; 2.84]**		
Heterogeneity: $I^2 = 98\%$ [98%; 99%], $\chi^2_{119} = 7563.80$ ($p = 0$)			0.001 0.1 1 10 1000				

$Tau^2 = 0.269$

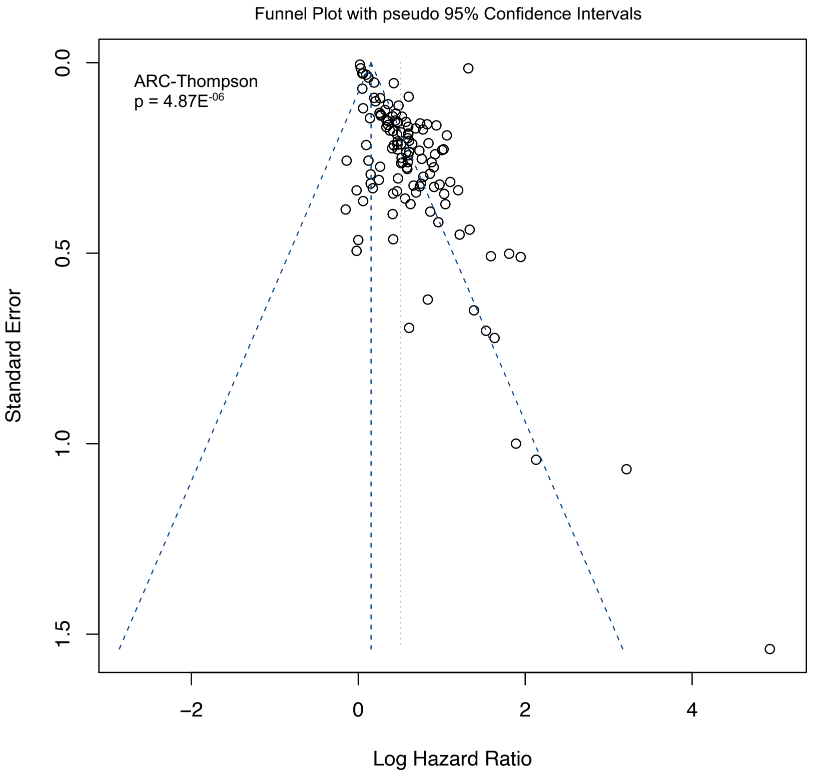

Supplementary Figure 10 - NLR: CSS in all operable cancers

Study	TE	seTE	HR	95%–CI	Weight (fixed)	Weight (random)
**Adjustment = Multivariate**						
Jagdev 2010	1.44	0.4922	4.20	[1.60; 11.02]	0.0%	1.0%
Gondo 2012	0.67	0.3212	1.95	[1.04; 3.66]	0.1%	1.7%
Stotz 2013	0.48	0.2306	1.61	[1.02; 2.53]	0.1%	2.4%
Azuma 2013	1.12	0.3968	3.06	[1.41; 6.66]	0.0%	1.3%
Pichler 2013	0.46	0.3239	1.59	[0.84; 3.00]	0.1%	1.7%
Noh 013	1.41	0.4710	4.08	[1.62; 10.27]	0.0%	1.0%
Shibutani 2013	0.48	0.1864	1.61	[1.12; 2.32]	0.2%	2.9%
Guthrie 2013	1.12	0.4658	3.07	[1.23; 7.65]	0.0%	1.0%
Perisanidis 2013	2.34	1.0680	10.37	[1.28; 84.12]	0.0%	0.2%
Jankova 2013	0.01	0.0482	1.01	[0.92; 1.11]	2.4%	4.2%
Luo 2014	1.85	0.6609	6.38	[1.75; 23.30]	0.0%	0.6%
Viers 2014	0.04	0.0193	1.04	[1.00; 1.08]	14.9%	4.3%
Kubo 2014	0.54	0.2624	1.71	[1.02; 2.86]	0.1%	2.2%
Dalpiaz 2014	1.00	0.3976	2.72	[1.25; 5.93]	0.0%	1.3%
Tanaka 2014	0.39	0.1820	1.47	[1.03; 2.10]	0.2%	2.9%
Ying 2014	1.02	0.2430	2.77	[1.72; 4.46]	0.1%	2.3%
Hermanns 2014	0.63	0.1535	1.88	[1.39; 2.54]	0.2%	3.2%
Dalpiaz (a) 2014	0.15	0.0736	1.16	[1.00; 1.34]	1.0%	4.0%
Viers 2014	0.02	0.0099	1.02	[1.00; 1.04]	56.5%	4.3%
Duan 2015	0.46	0.1749	1.59	[1.13; 2.24]	0.2%	3.0%
Bagante 2015	0.79	0.3312	2.21	[1.15; 4.23]	0.1%	1.7%
Deng 2015	0.43	0.1640	1.53	[1.11; 2.11]	0.2%	3.1%
Shin 2015	1.82	0.9131	6.19	[1.03; 37.06]	0.0%	0.3%
Cummings 2015	0.52	0.2514	1.68	[1.03; 2.75]	0.1%	2.2%
Neal 2015	0.66	0.1637	1.93	[1.40; 2.66]	0.2%	3.1%
Fu 2016	0.35	0.1512	1.42	[1.06; 1.91]	0.2%	3.2%
Morizawa 2016	0.96	0.2567	2.60	[1.57; 4.30]	0.1%	2.2%
Kosumi 2016	0.61	0.2807	1.84	[1.06; 3.19]	0.1%	2.0%
Wang 2016	0.10	0.0182	1.10	[1.06; 1.14]	16.7%	4.3%
Chen 2016	0.05	0.1786	1.05	[0.74; 1.49]	0.2%	2.9%
Mohri 2016	0.68	0.3062	1.97	[1.08; 3.59]	0.1%	1.8%
Bhindi 2016	0.39	0.1033	1.47	[1.20; 1.80]	0.5%	3.7%
Xie 2016	0.18	0.1837	1.20	[0.84; 1.72]	0.2%	2.9%
Kang 2016	0.15	0.0462	1.16	[1.06; 1.27]	2.6%	4.2%
**Pooled HR by Fixed Effects**			**1.06**	**[1.04; 1.07]**	**97.3%**	**--**
**Pooled HR by Random Effects**			**1.58**	**[1.40; 1.79]**	**--**	**83.3%**
Heterogeneity: $I^2 = 83\%$ [77%; 87%], $\chi^2_{33} = 192.13$ ($p < 0.01$)						
**Adjustment = Univariate**						
Dutta 2011	0.08	0.1876	1.08	[0.75; 1.56]	0.2%	2.9%
Dutta 2012	0.17	0.2306	1.19	[0.76; 1.87]	0.1%	2.4%
Szkandera 2014	0.68	0.4807	1.98	[0.77; 5.08]	0.0%	1.0%
Forrest 2014	0.82	0.4229	2.27	[0.99; 5.20]	0.0%	1.2%
Neofytou 2015	0.18	0.0639	1.20	[1.06; 1.36]	1.4%	4.1%
Lee 2015	0.09	0.0739	1.09	[0.94; 1.26]	1.0%	4.0%
Kim 2015	0.17	0.4261	1.18	[0.51; 2.72]	0.0%	1.2%
**Pooled HR by Fixed Effects**			**1.16**	**[1.07; 1.27]**	**2.7%**	**--**
**Pooled HR by Random Effects**			**1.16**	**[1.07; 1.27]**	**--**	**16.7%**
Heterogeneity: $I^2 = 0\%$ [0%; 64%], $\chi^2_6 = 4.9$ ($p = 0.56$)						
**Pooled HR by Fixed Effects**			**1.06**	**[1.05; 1.08]**	**100.0%**	**--**
**Pooled HR by Random Effects**			**1.50**	**[1.35; 1.67]**	**--**	**100.0%**
**Prediction interval**				**[0.87; 2.58]**		
Heterogeneity: $I^2 = 80\%$ [74%; 85%], $\chi^2_{40} = 201.35$ ($p < 0.01$)						

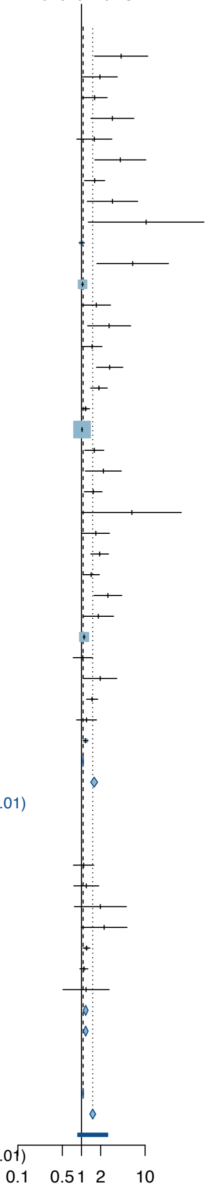

Tau²= 0.262

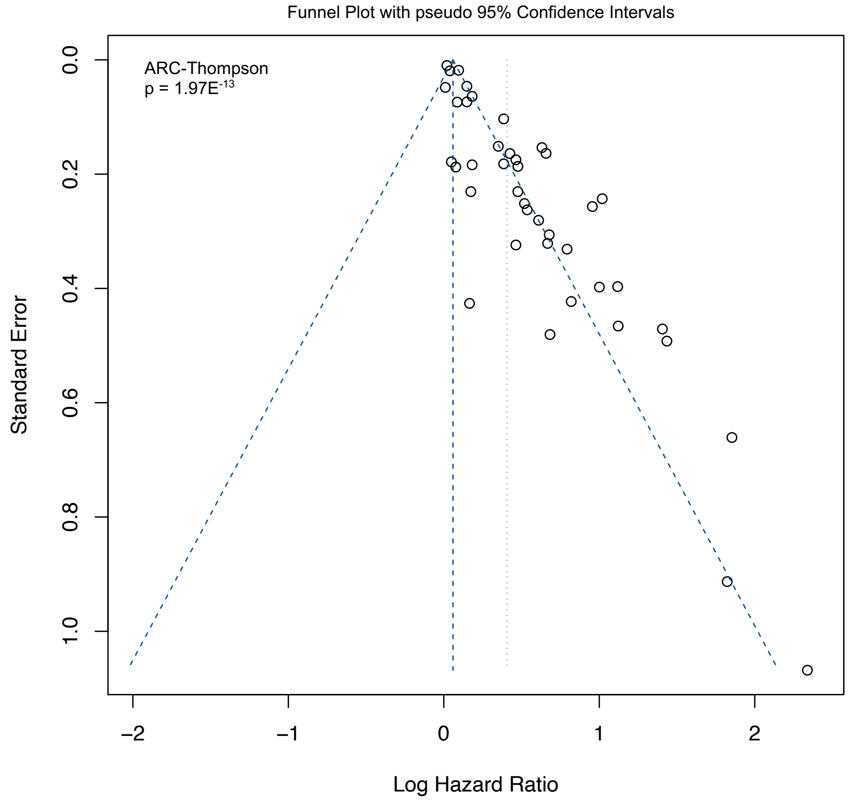

Supplementary *Figure 11* - NLR: OS in advanced cancer with immunotherapy

Study	ES	seES	HR	95%–CI	Weight (fixed)	Weight (random)
Zheng 2013	1.04	0.3791	2.84	[1.35; 5.97]	0.8%	2.8%
Cetin 2013	0.88	0.3025	2.41	[1.33; 4.36]	1.2%	3.6%
Dirican 2013	0.84	0.3878	2.31	[1.08; 4.94]	0.7%	2.7%
Kobayashi (a) 2013	0.96	0.4828	2.62	[1.02; 6.75]	0.5%	2.0%
Wang 2014	0.88	0.4895	2.41	[0.92; 6.29]	0.5%	2.0%
Dana 2014	0.81	0.2435	2.24	[1.39; 3.61]	1.8%	4.4%
Fonseca 2014	0.69	0.2269	2.00	[1.28; 3.12]	2.1%	4.6%
Keizman 2014	1.08	0.1981	2.95	[2.00; 4.35]	2.8%	5.1%
Park 2014	0.02	0.0872	1.02	[0.86; 1.21]	14.2%	6.7%
Ferrucci (a) 2015	0.67	0.2673	1.96	[1.16; 3.31]	1.5%	4.0%
Ferrucci (b) 2015	0.71	0.3586	2.04	[1.01; 4.12]	0.8%	3.0%
Namikawa 2015	0.25	0.2901	1.28	[0.72; 2.26]	1.3%	3.7%
Santoni 2015	0.79	0.3078	2.21	[1.21; 4.04]	1.1%	3.5%
Chrom 2016	0.75	0.2157	2.11	[1.38; 3.22]	2.3%	4.8%
Gardini 2016	0.14	0.0546	1.15	[1.03; 1.28]	36.2%	7.0%
Zhang 2016	0.33	0.1568	1.39	[1.02; 1.89]	4.4%	5.7%
Passardi 2016	0.41	0.1252	1.51	[1.18; 1.93]	6.9%	6.2%
Ferrucci 2016	0.83	0.1062	2.29	[1.86; 2.82]	9.6%	6.5%
Bagley 2017	0.73	0.2379	2.07	[1.30; 3.30]	1.9%	4.5%
Kuzman 2017	0.89	0.4338	2.44	[1.04; 5.71]	0.6%	2.3%
Tanaka (b) 2017	0.86	0.1841	2.37	[1.65; 3.40]	3.2%	5.3%
Cassidy 2017	0.71	0.1586	2.03	[1.49; 2.77]	4.3%	5.7%
Tanaka (a) 2017	1.47	0.2736	4.34	[2.54; 7.42]	1.4%	4.0%
**Pooled HR by Fixed Effects**			**1.51**	**[1.41; 1.61]**	**100.0%**	**--**
**Pooled HR by Random Effects**			**1.95**	**[1.67; 2.29]**	**--**	**100.0%**
**Prediction interval**				**[1.03; 3.71]**		

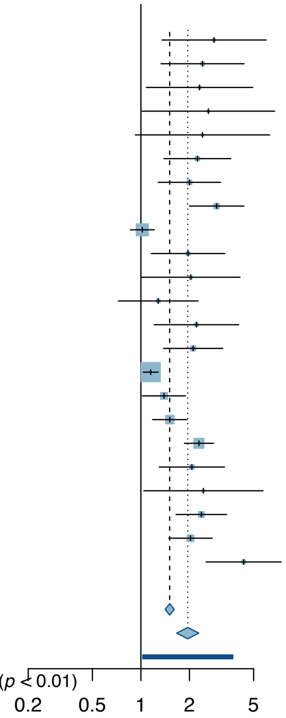

Heterogeneity: $I^2 = 81\%$ [73%; 87%], $\chi^2_{22} = 118.18$ ($p < 0.01$)

Tau²= 0.298

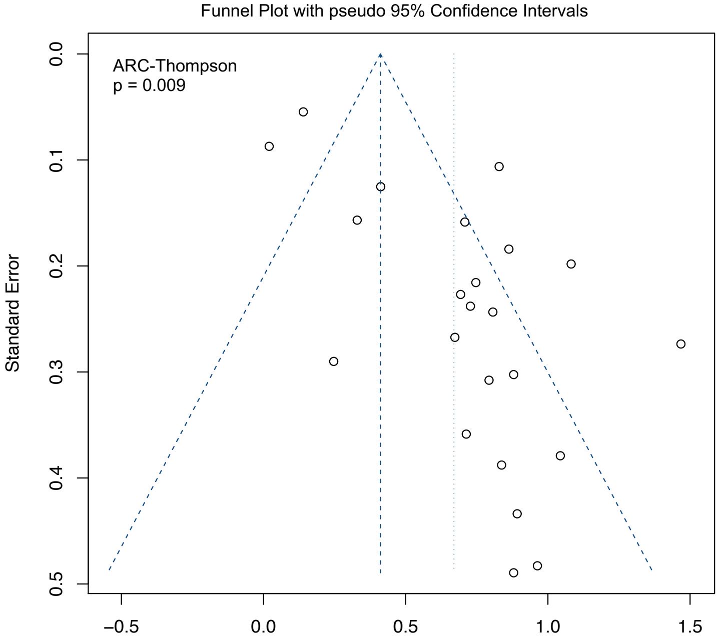

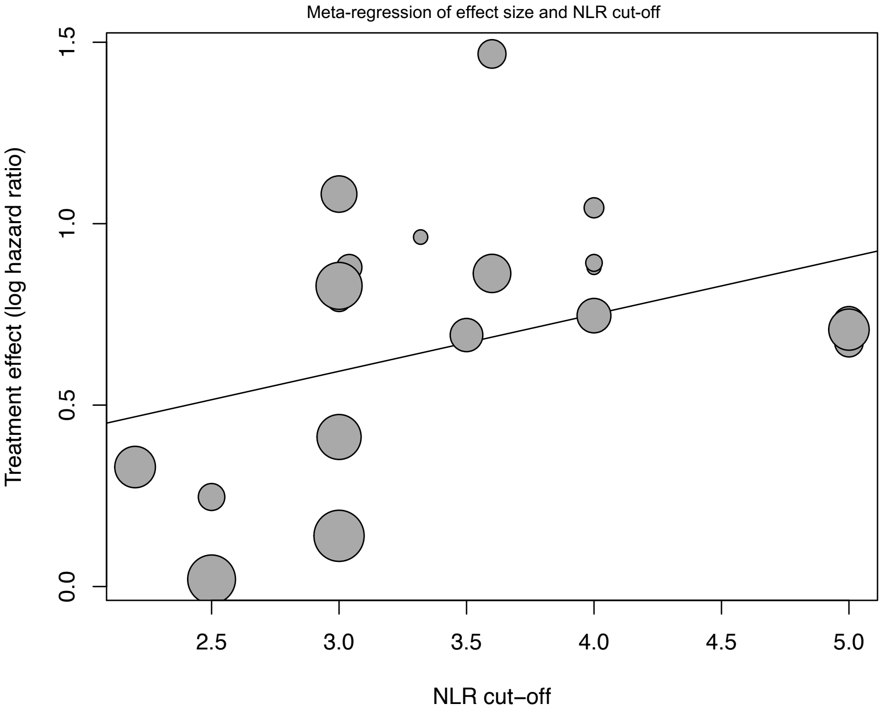

Supplementary *Figure 12* - NLR: OS in advanced cancer with anti-VEGFR

Study	ES	esES	HR	95%-CI	Weight (fixed)	Weight (random)
Zheng 2013	1.04	0.3791	2.84	[1.35; 5.97]	0.9%	4.8%
Dirican 2013	0.84	0.3878	2.31	[1.08; 4.94]	0.9%	4.7%
Kobayashi (a) 2013	0.96	0.4828	2.62	[1.02; 6.75]	0.6%	3.7%
Dana 2014	0.81	0.2435	2.24	[1.39; 3.61]	2.2%	7.0%
Fonseca 2014	0.69	0.2269	2.00	[1.28; 3.12]	2.6%	7.2%
Keizman 2014	1.08	0.1981	2.95	[2.00; 4.35]	3.4%	7.8%
Park 2014	0.02	0.0872	1.02	[0.86; 1.21]	17.5%	9.5%
Santoni 2015	0.79	0.3078	2.21	[1.21; 4.04]	1.4%	5.9%
Chrom 2016	0.75	0.2157	2.11	[1.38; 3.22]	2.9%	7.4%
Gardini 2016	0.14	0.0546	1.15	[1.03; 1.28]	44.6%	9.8%
Zhang 2016	0.33	0.1568	1.39	[1.02; 1.89]	5.4%	8.5%
Ferrucci 2016	0.83	0.1062	2.29	[1.86; 2.82]	11.8%	9.3%
Tanaka (b) 2017	0.86	0.1841	2.37	[1.65; 3.40]	3.9%	8.0%
Tanaka (a) 2017	1.47	0.2736	4.34	[2.54; 7.42]	1.8%	6.4%
**Pooled HR by Fixed Effects**			**1.45**	**[1.35; 1.55]**	**100.0%**	**--**
**Pooled HR by Random Effects**			**2.02**	**[1.61; 2.53]**	**--**	**100.0%**
**Prediction interval**				**[0.88; 4.64]**		

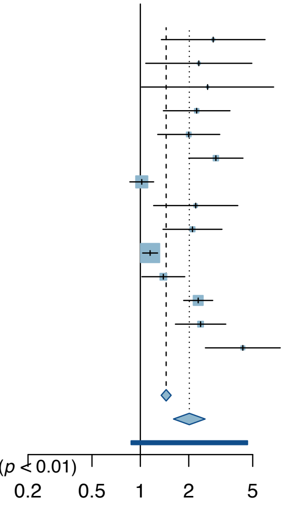

Heterogeneity: I^2 = 88% [81%; 92%], χ^2_{13} = 105.08 (p < 0.01)

Tau²= 0.365

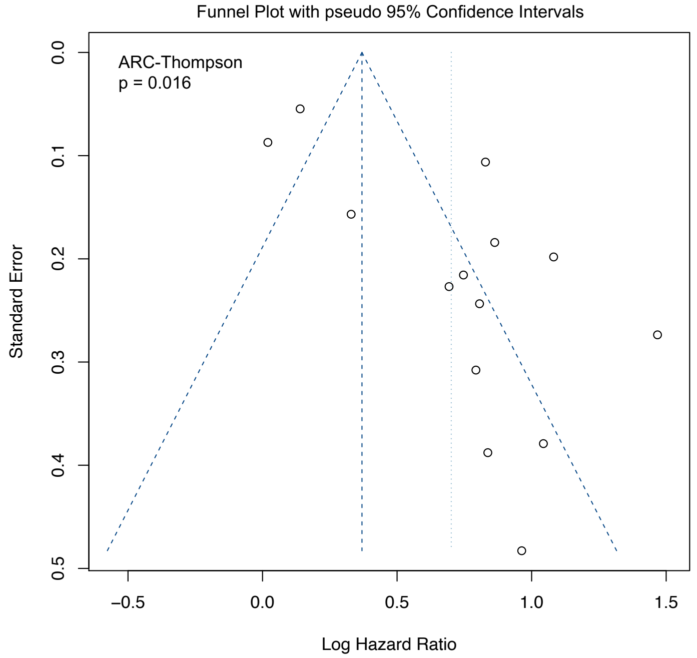

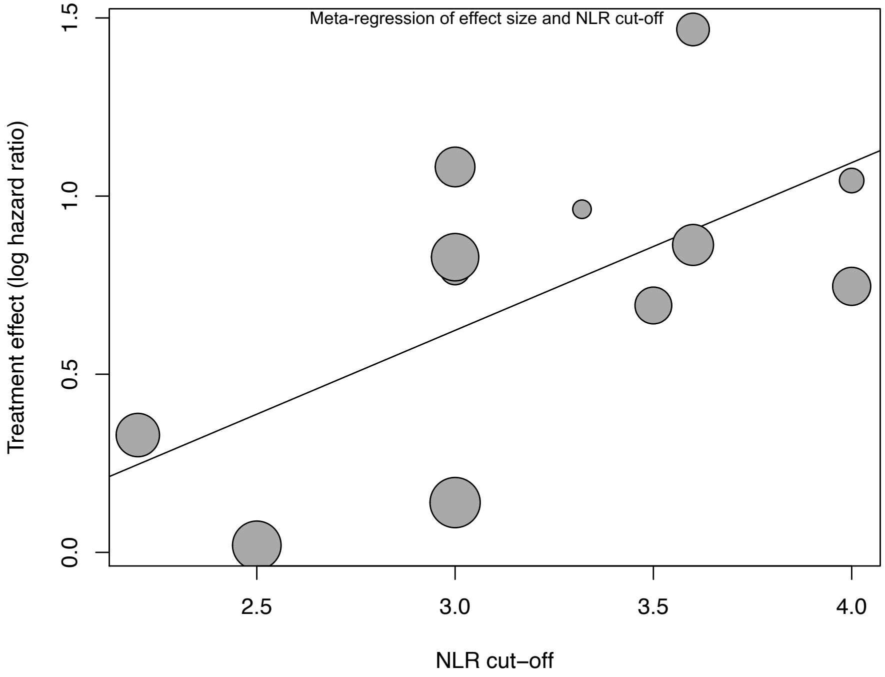

Supplementary *Figure 13* - NLR: OS with immune checkpoint inhibitors

Study	TE	seTE	HR	95%–CI	Weight (fixed)	Weight (random)
**Adjustment = Mutlivariate**						
Bagley 2017	0.73	0.2379	2.07	[1.30; 3.30]	1.0%	7.0%
Diem 2017	1.61	0.4607	5.01	[2.03; 12.36]	0.3%	4.4%
Cassidy 2017	0.71	0.1586	2.03	[1.49; 2.77]	2.2%	8.0%
Ferrucci 2015	1.43	0.3122	4.17	[2.26; 7.69]	0.6%	6.0%
Ferrucci 2015	0.67	0.2704	1.96	[1.15; 3.33]	0.8%	6.6%
Ferrucci 2015	0.71	0.3435	2.04	[1.04; 4.00]	0.5%	5.7%
Ferrucci 2015	1.35	0.6008	3.85	[1.19; 12.50]	0.2%	3.2%
Jung 2017	−0.01	0.1429	0.99	[0.75; 1.31]	2.7%	8.1%
Khoja 2016	−0.01	0.0251	0.99	[0.94; 1.04]	87.9%	8.9%
Zaragoza 2016	0.79	0.3959	2.20	[1.01; 4.78]	0.4%	5.0%
Fukui 2018	1.43	0.5770	4.17	[1.35; 12.92]	0.2%	3.4%
Rosner 2018	0.67	0.2881	1.95	[1.11; 3.43]	0.7%	6.4%
Fujisawa 2018	1.43	0.4733	4.16	[1.65; 10.52]	0.2%	4.2%
**Pooled HR by Fixed Effects**			**1.05**	**[1.00; 1.10]**	**97.5%**	**--**
**Pooled HR by Random Effects**			**2.14**	**[1.57; 2.91]**	**--**	**76.9%**
Heterogeneity: $I^2 = 88\%$ [81%; 92%], $\chi^2_{12} = 98.09$ ($p < 0.01$)						
**Adjustment = Univariate**						
Suh 2017	0.51	0.4487	1.66	[0.69; 4.00]	0.3%	4.5%
Sun 2017	0.41	0.1954	1.50	[1.02; 2.20]	1.5%	7.5%
Jeyakumar 017	1.38	0.5996	3.98	[1.23; 12.89]	0.2%	3.2%
Bilen 2018	2.81	0.9141	16.67	[2.78; 100.00]	0.1%	1.7%
Park 2018	1.11	0.3097	3.03	[1.65; 5.56]	0.6%	6.1%
**Pooled HR by Fixed Effects**			**2.01**	**[1.51; 2.69]**	**2.5%**	**--**
**Pooled HR by Random Effects**			**2.56**	**[1.45; 4.52]**	**--**	**23.1%**
Heterogeneity: $I^2 = 63\%$ [2%; 86%], $\chi^2_4 = 10.84$ ($p = 0.03$)						
**Pooled HR by Fixed Effects**			**1.07**	**[1.02; 1.12]**	**100.0%**	**--**
**Pooled HR by Random Effects**			**2.21**	**[1.70; 2.88]**	**--**	**100.0%**
**Prediction interval**				**[0.82; 5.97]**		
Heterogeneity: $I^2 = 87\%$ [80%; 91%], $\chi^2_{17} = 127.64$ ($p < 0.01$)						

Hazard Ratio axis: 0.1 0.51 2 10

Tau²= 0.448

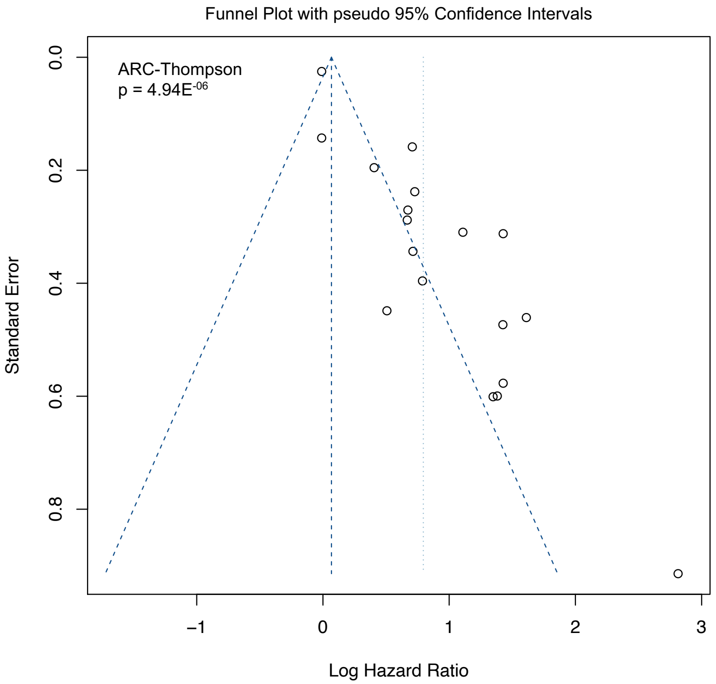

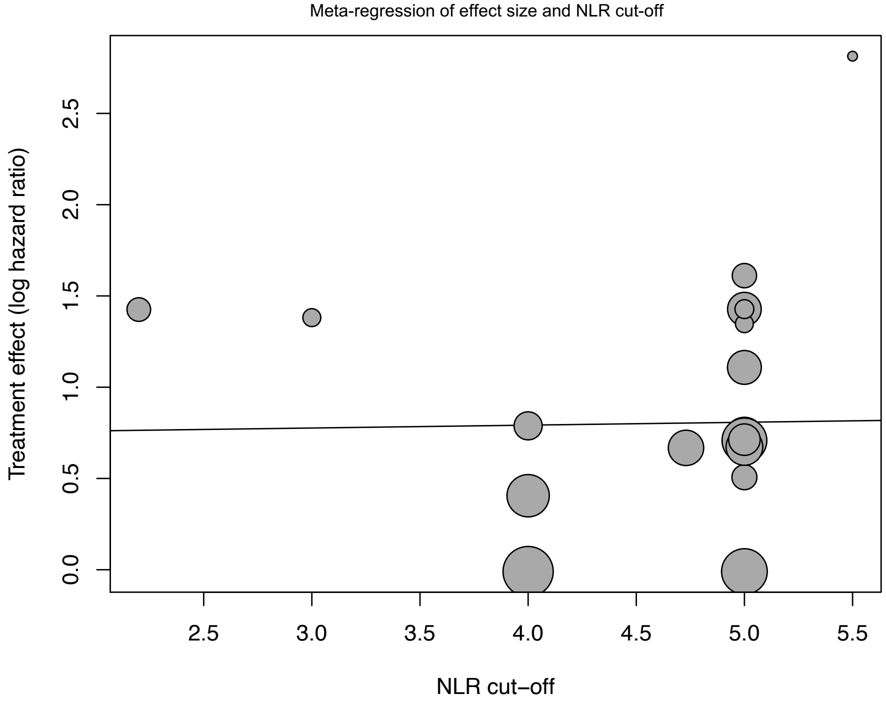

Supplementary *Figure 14* - NLR: PFS in advanced cancer with immunotherapy

Study	ES	esES	HR	95%–CI	Weight (fixed)	Weight (random)
Kobayashi (a) 2013	1.21	0.5135	3.37	[1.23; 9.22]	0.2%	2.6%
Kobayashi (b) 2013	1.17	0.5110	3.21	[1.18; 8.74]	0.2%	2.6%
Botta 2013	0.51	0.2618	1.67	[1.00; 2.79]	0.9%	5.8%
Cetin 2013	0.10	0.0355	1.11	[1.04; 1.19]	50.8%	9.9%
Dirican 2013	0.72	0.3586	2.06	[1.02; 4.16]	0.5%	4.2%
Dana 2014	0.78	0.2550	2.19	[1.33; 3.61]	1.0%	5.9%
Gunduz 214	1.52	0.7028	4.56	[1.15; 18.08]	0.1%	1.6%
Keizman 2016	1.26	0.1936	3.51	[2.40; 5.13]	1.7%	7.2%
Santoni 2015	0.79	0.3078	2.21	[1.21; 4.04]	0.7%	5.0%
Ferrucci 2016	0.71	0.1022	2.03	[1.66; 2.48]	6.1%	9.0%
Gardini 2016	0.08	0.0495	1.08	[0.98; 1.19]	26.1%	9.8%
Passardi 2016	0.57	0.1431	1.76	[1.33; 2.33]	3.1%	8.2%
Zhang 2016	0.43	0.1435	1.54	[1.16; 2.04]	3.1%	8.2%
Bagley 2017	0.36	0.1712	1.43	[1.02; 2.00]	2.2%	7.7%
Cassidy 2017	0.59	0.1565	1.81	[1.33; 2.46]	2.6%	8.0%
Kuzman 2017	0.39	0.3657	1.47	[0.72; 3.01]	0.5%	4.1%
**Pooled HR by Fixed Effects**			**1.25**	**[1.19; 1.31]**	**100.0%**	**--**
**Pooled HR by Random Effects**			**1.78**	**[1.47; 2.15]**	**--**	**100.0%**
**Prediction interval**				**[0.90; 3.54]**		

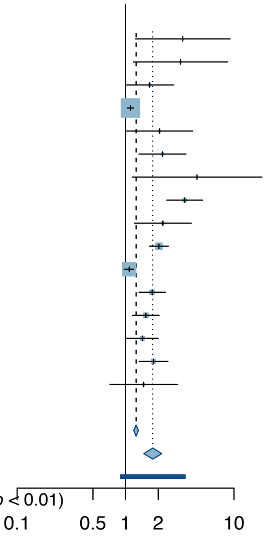

Heterogeneity: I^2 = 86% [79%; 91%], χ^2_{15} = 107.08 ($p < 0.01$)

Tau^2= 0.305

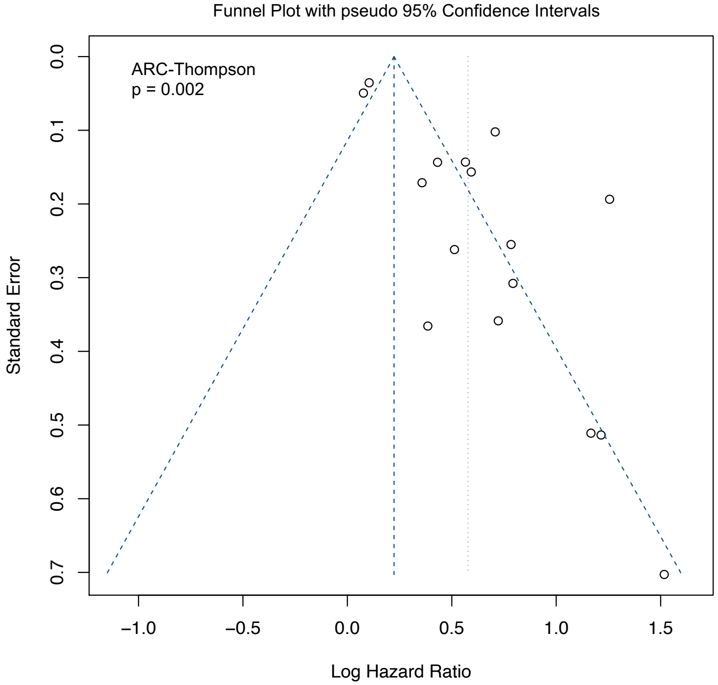

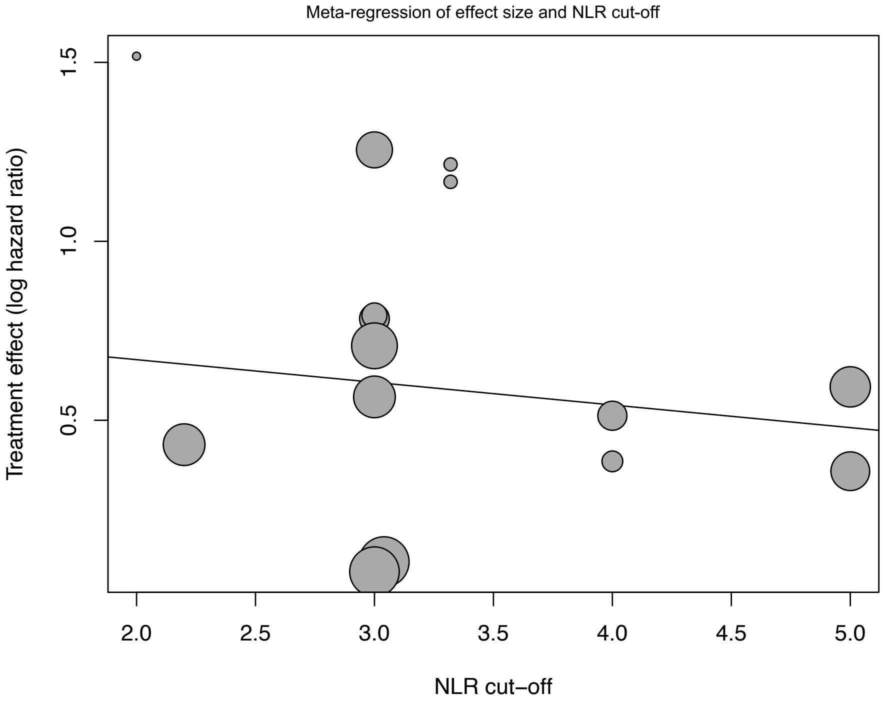

Supplementary *Figure 15* - NLR: PFS with immune checkpoint inhibitors

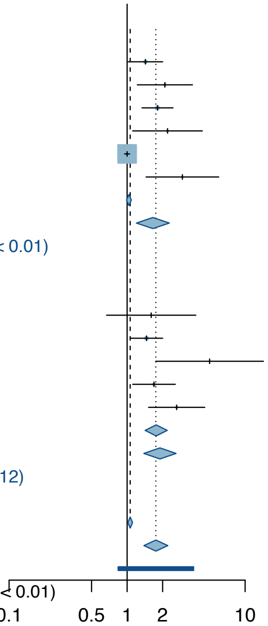

Study	TE	seTE	HR	95%–CI	Weight (fixed)	Weight (random)
**Adjustment = Mutlivariate**						
Bagley 2017	0.36	0.1712	1.43	[1.02; 2.00]	1.9%	11.7%
Diem 2017	0.74	0.2746	2.09	[1.22; 3.58]	0.7%	8.5%
Cassidy 2017	0.59	0.1545	1.81	[1.34; 2.45]	2.3%	12.2%
Jung 2017	0.79	0.3466	2.20	[1.12; 4.34]	0.5%	6.7%
Khoja 2016	0.00	0.0249	1.00	[0.95; 1.05]	89.6%	15.3%
Jeyakumar 2017	1.08	0.3614	2.94	[1.45; 5.97]	0.4%	6.4%
**Pooled HR by Fixed Effects**			**1.04**	**[0.99; 1.09]**	**95.4%**	**--**
**Pooled HR by Random Effects**			**1.65**	**[1.20; 2.28]**	**--**	**60.7%**
Heterogeneity: I^2 = 87% [74%; 93%], χ^2_5 = 38.19 (p < 0.01)						
**Adjustment = Univariate**						
Suh 2017	0.47	0.4440	1.60	[0.67; 3.82]	0.3%	4.9%
Shiroyama 2017	0.38	0.1606	1.46	[1.07; 2.00]	2.2%	12.0%
Bilen 2018	1.61	0.5358	5.00	[1.75; 14.29]	0.2%	3.7%
Park 2018	0.52	0.2119	1.69	[1.12; 2.56]	1.2%	10.3%
Ferrucci 2015	0.97	0.2797	2.63	[1.52; 4.55]	0.7%	8.3%
**Pooled HR by Fixed Effects**			**1.76**	**[1.42; 2.19]**	**4.6%**	**--**
**Pooled HR by Random Effects**			**1.90**	**[1.39; 2.60]**	**--**	**39.3%**
Heterogeneity: I^2 = 45% [0%; 80%], χ^2_4 = 7.3 (p = 0.12)						
**Pooled HR by Fixed Effects**			**1.06**	**[1.01; 1.11]**	**100.0%**	**--**
**Pooled HR by Random Effects**			**1.75**	**[1.39; 2.21]**	**--**	**100.0%**
**Prediction interval**				**[0.84; 3.66]**		
Heterogeneity: I^2 = 85% [75%; 91%], χ^2_{10} = 67.67 (p < 0.01)						

Tau²= 0.303

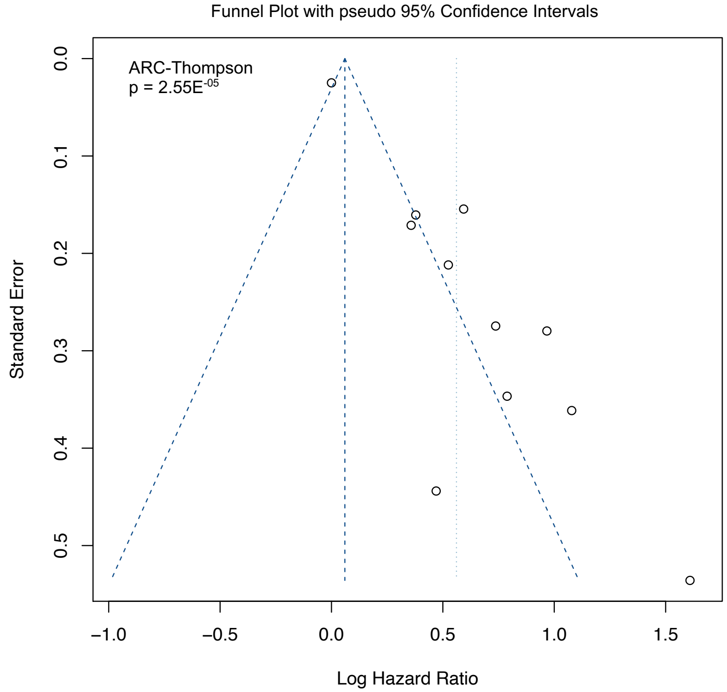

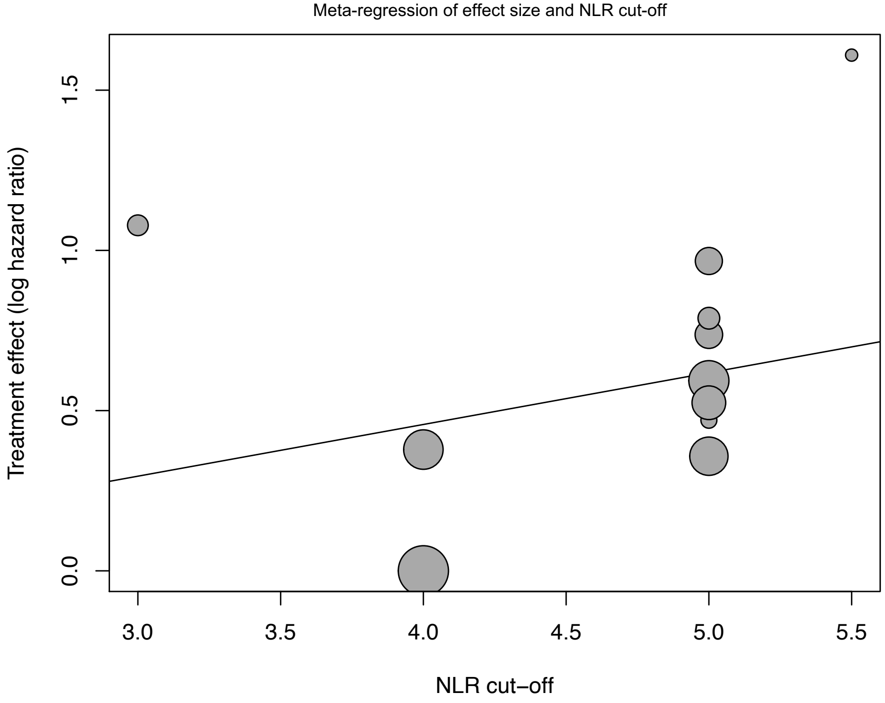

Supplementary *Figure 16* - NLR: OS with neoadjuvant chemotherapy

Study	ES	seES	HR	95%−CI	Weight (fixed)	Weight (random)
Asano 2015	0.16	0.4770	1.17	[0.46; 2.98]	0.3%	3.2%
Aziz 2014	1.18	0.5337	3.26	[1.15; 9.28]	0.2%	2.8%
Buisan 2017	0.11	0.0560	1.12	[1.00; 1.25]	19.4%	9.7%
Carruthers 2012	1.95	0.5148	7.00	[2.55; 19.20]	0.2%	2.9%
Chen 2017	0.21	0.4197	1.23	[0.54; 2.80]	0.3%	3.8%
Enriquez 2017	0.49	0.1748	1.64	[1.16; 2.31]	2.0%	7.8%
Hodek 2016	0.19	0.0817	1.21	[1.03; 1.42]	9.2%	9.4%
Jin 2017	0.57	0.2583	1.76	[1.06; 2.92]	0.9%	6.2%
Koh 2014	3.21	1.0665	24.87	[3.08; 201.13]	0.1%	0.9%
Miyata 2011	0.26	0.2730	1.30	[0.76; 2.22]	0.8%	5.9%
Mmeje 2016	0.74	0.1394	2.10	[1.60; 2.76]	3.1%	8.4%
Nagasaki 2015	1.22	0.4853	3.38	[1.31; 8.75]	0.3%	3.2%
Omichi 2017	1.12	0.4215	3.06	[1.34; 6.99]	0.3%	3.8%
Rosculet 2017	1.10	0.4779	3.01	[1.18; 7.68]	0.3%	3.2%
Salih 2016	0.08	0.0320	1.08	[1.01; 1.15]	59.4%	9.8%
Shen 2017	0.07	0.2271	1.07	[0.69; 1.67]	1.2%	6.8%
Shen 2014	0.75	0.3175	2.12	[1.14; 3.95]	0.6%	5.2%
Siano 2016	0.43	0.2135	1.54	[1.01; 2.34]	1.3%	7.0%
**Pooled HR by Fixed Effects**			**1.17**	**[1.11; 1.23]**	**100.0%**	**--**
**Pooled HR by Random Effects**			**1.66**	**[1.36; 2.04]**	**--**	**100.0%**
**Prediction interval**				**[0.80; 3.48]**		

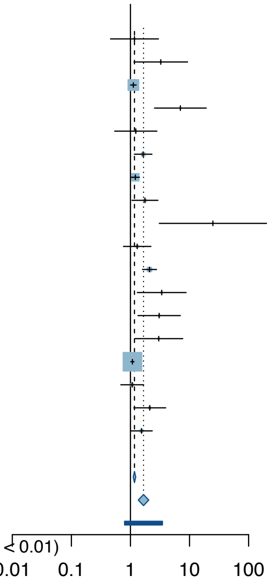

Heterogeneity: $I^2 = 77\%$ [64%; 85%], $\chi^2_{17} = 74.20$ ($p < 0.01$)

Tau²= 0.332

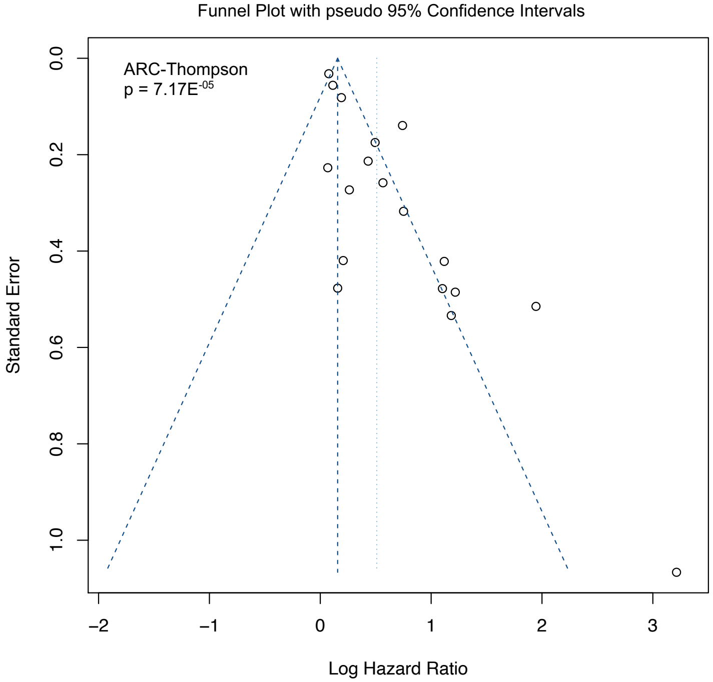

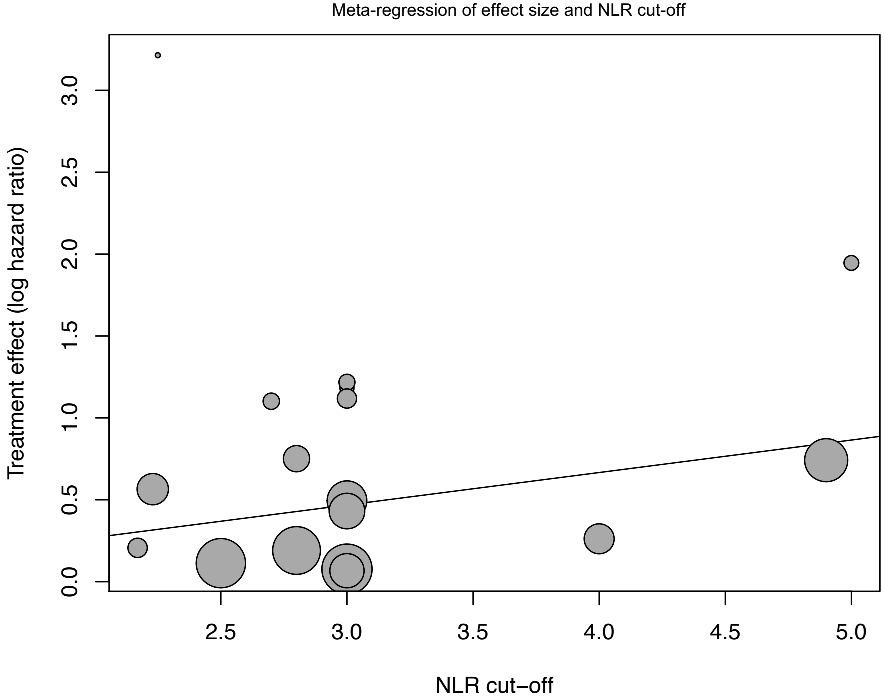

Supplementary *Figure 17* - NLR: RFS with neoadjuvant chemotherapy

Study	ES	seES	HR	95%-CI	Weight (fixed)	Weight (random)
Chen 2017	0.45	0.2058	1.57	[1.05; 2.35]	34.6%	24.6%
Jin 2017	0.52	0.2551	1.68	[1.02; 2.77]	22.5%	20.2%
Kim 2014	1.03	0.4527	2.80	[1.15; 6.80]	7.1%	9.7%
Koh 2014	1.35	0.4390	3.87	[1.64; 9.15]	7.6%	10.1%
Nagasaki 2015	0.07	0.3607	1.07	[0.53; 2.17]	11.3%	13.4%
Omichi 2017	1.20	0.4070	3.31	[1.49; 7.35]	8.8%	11.3%
Rosculet 2017	0.97	0.4267	2.63	[1.14; 6.07]	8.0%	10.6%
**Pooled HR by Fixed Effects**			**1.90**	**[1.50; 2.41]**	**100.0%**	**--**
**Pooled HR by Random Effects**			**2.01**	**[1.47; 2.76]**	**--**	**100.0%**
**Prediction interval**				**[0.94; 4.33]**		

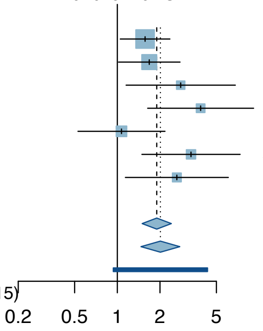

Heterogeneity: $I^2 = 36\%$ [0%; 73%], $\chi^2_6 = 9.43$ ($p = 0.15$)

Tau²= 0.251

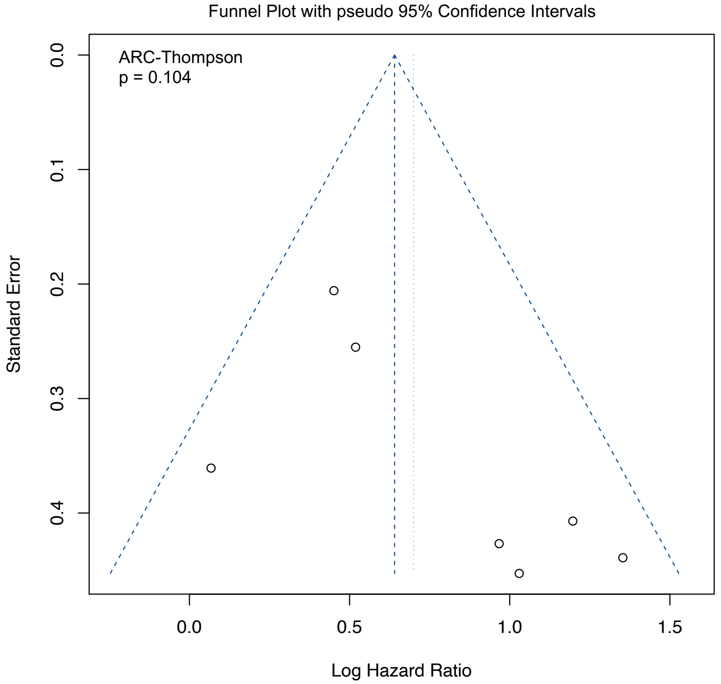

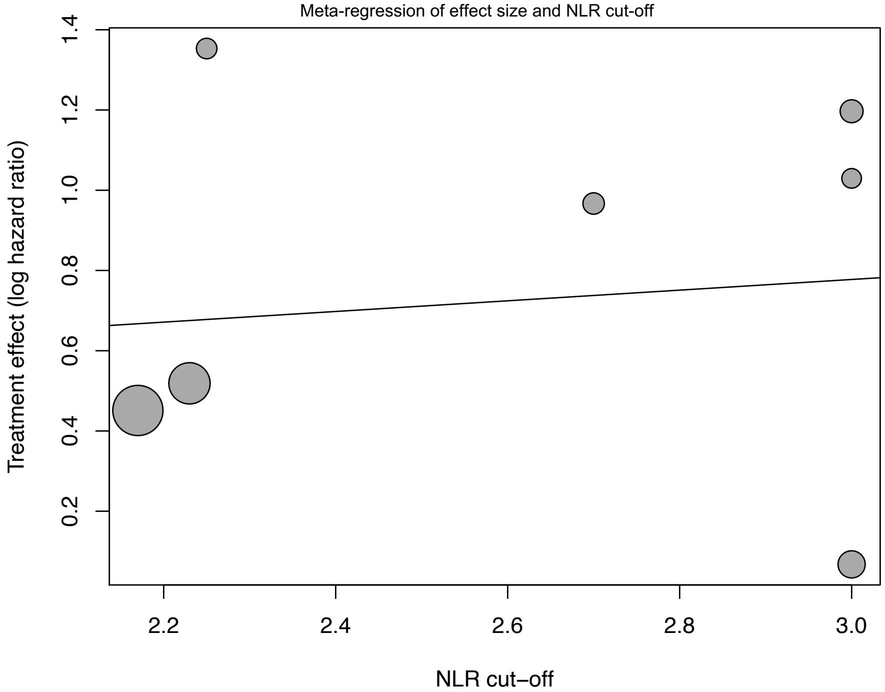

Supplementary *Figure 18* - NLR: CSS with neoadjuvant chemotherapy

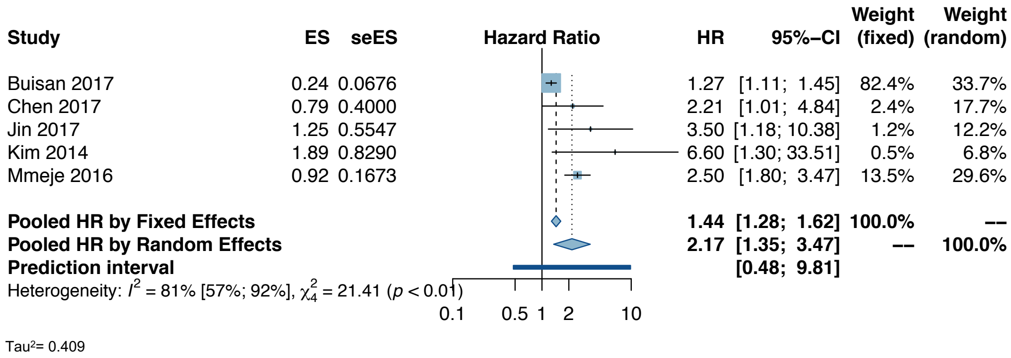

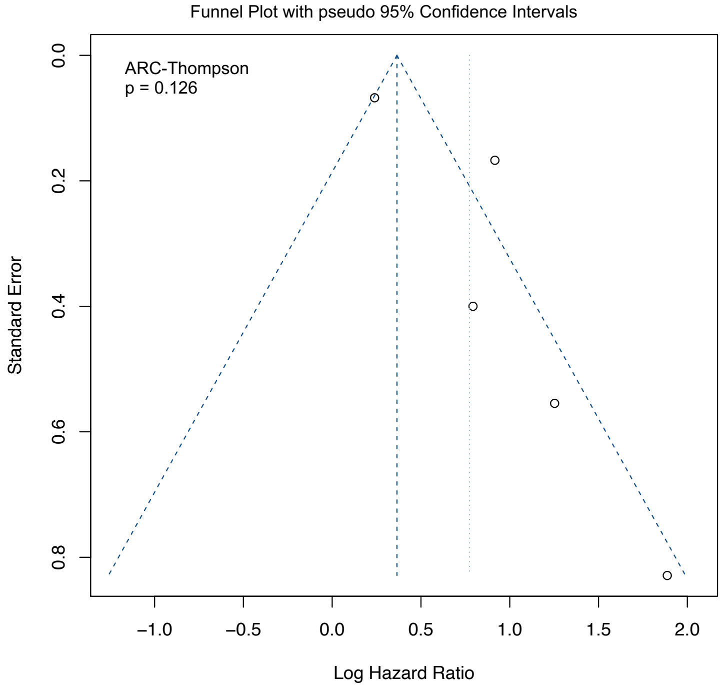

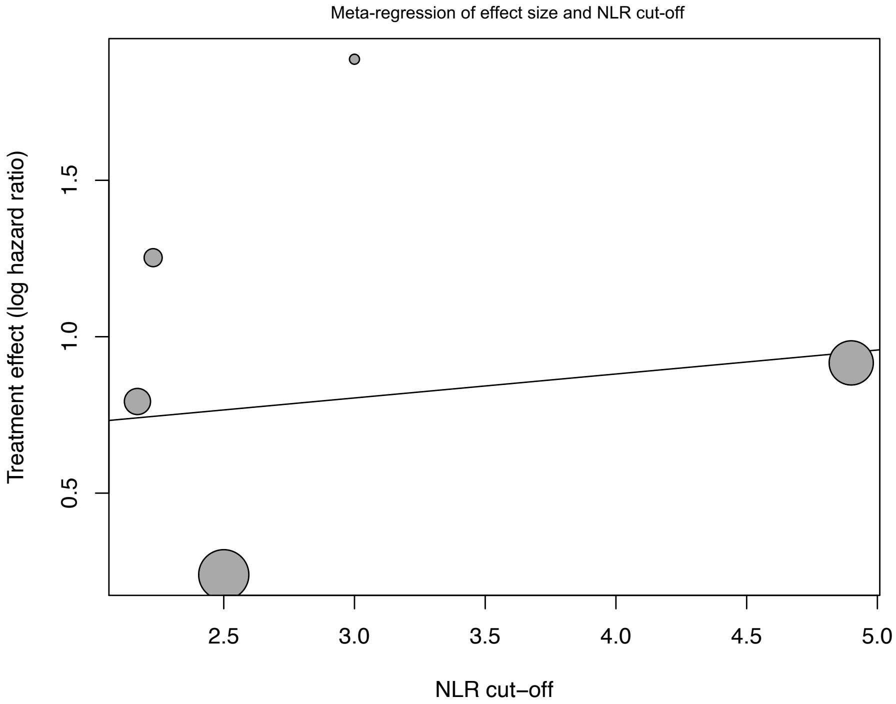

Supplementary *Figure 19* – IN: OS in gastric cancers

Study	ES	seES	Hazard Ratio	HR	95%–CI	Weight (fixed)	Weight (random)
Zhao 2012	0.66	0.3011		1.94	[1.08; 3.50]	27.9%	46.6%
Caruso 2002	–0.24	0.1871		0.79	[0.55; 1.14]	72.1%	53.4%
**Pooled HR by Fixed Effects**				**1.01**	**[0.74; 1.39]**	**100.0%**	**--**
**Pooled HR by Random Effects**				**1.20**	**[0.50; 2.89]**	**--**	**100.0%**

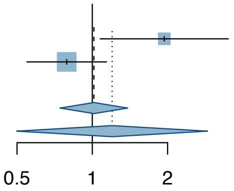

Heterogeneity: $I^2 = 84\%$, $\chi^2_1 = 6.42$ ($p = 0.01$)

Tau^2= 0.584

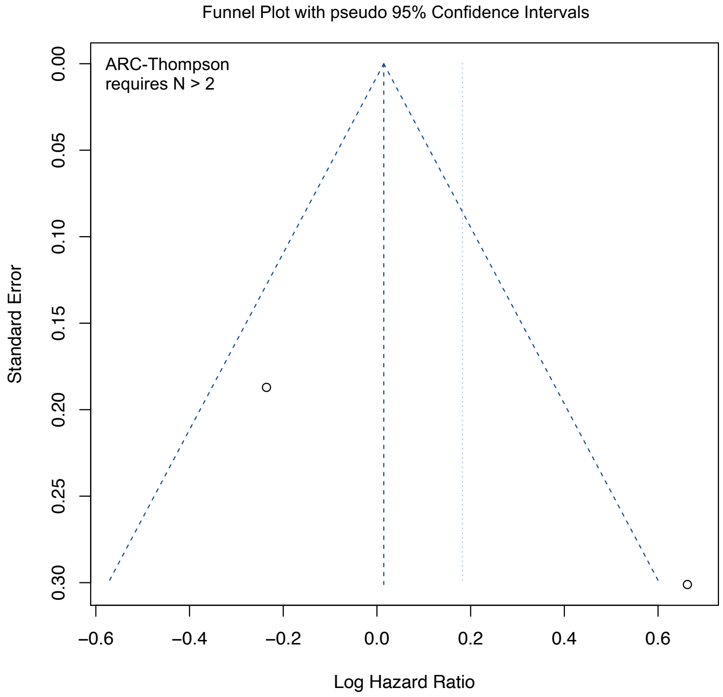

Supplementary Figure 20 - NLR: OS in gastric cancers

Study	ES	seES	HR	95%–CI	Weight (fixed)	Weight (random)
Aurello 2014	0.41	0.3989	1.51	[0.69; 3.30]	0.5%	2.1%
Cho 2014	0.45	0.1286	1.57	[1.22; 2.02]	5.2%	9.4%
Dirican 2013	1.01	0.1690	2.75	[1.97; 3.83]	3.0%	7.2%
El Aziz 2014	1.18	0.5309	3.25	[1.15; 9.20]	0.3%	1.2%
Graziosi 2015	0.53	0.2600	1.70	[1.02; 2.83]	1.3%	4.2%
Jeong 2012	0.76	0.0390	2.14	[1.98; 2.31]	56.2%	14.9%
Jiang 2014	0.47	0.2195	1.60	[1.04; 2.46]	1.8%	5.3%
Jin 2013	0.85	0.3697	2.34	[1.13; 4.83]	0.6%	2.4%
Jung 2011	0.48	0.1699	1.62	[1.16; 2.26]	3.0%	7.2%
Kim and Choi 2012	1.32	0.7604	3.74	[0.84; 16.60]	0.1%	0.6%
Kunisaki 2012	0.53	0.3098	1.70	[0.93; 3.12]	0.9%	3.2%
Lee D 2013	1.01	0.3790	2.75	[1.31; 5.78]	0.6%	2.3%
Lee S 2013	0.81	0.1399	2.25	[1.71; 2.96]	4.4%	8.7%
Mohri 2010	0.76	0.2996	2.14	[1.19; 3.85]	1.0%	3.4%
Mohri 2014	0.83	0.2406	2.29	[1.43; 3.67]	1.5%	4.7%
Shimada 2010	0.61	0.1994	1.84	[1.24; 2.72]	2.1%	6.0%
Ubukata 2010	1.73	0.4300	5.64	[2.43; 13.10]	0.5%	1.8%
Wang 2012	0.91	0.3587	2.49	[1.23; 5.03]	0.7%	2.5%
Yamanaka 2007	0.42	0.0719	1.52	[1.32; 1.75]	16.5%	13.0%
**Pooled HR by Fixed Effects**			**1.98**	**[1.87; 2.10]**	**100.0%**	**--**
**Pooled HR by Random Effects**			**1.98**	**[1.75; 2.23]**	**--**	**100.0%**
**Prediction interval**				**[1.39; 2.82]**		

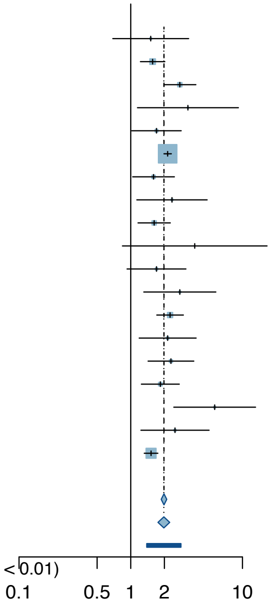

Heterogeneity: $I^2 = 53\%$ [21%; 72%], $\chi^2_{18} = 38.17$ ($p < 0.01$)

Tau²= 0.155

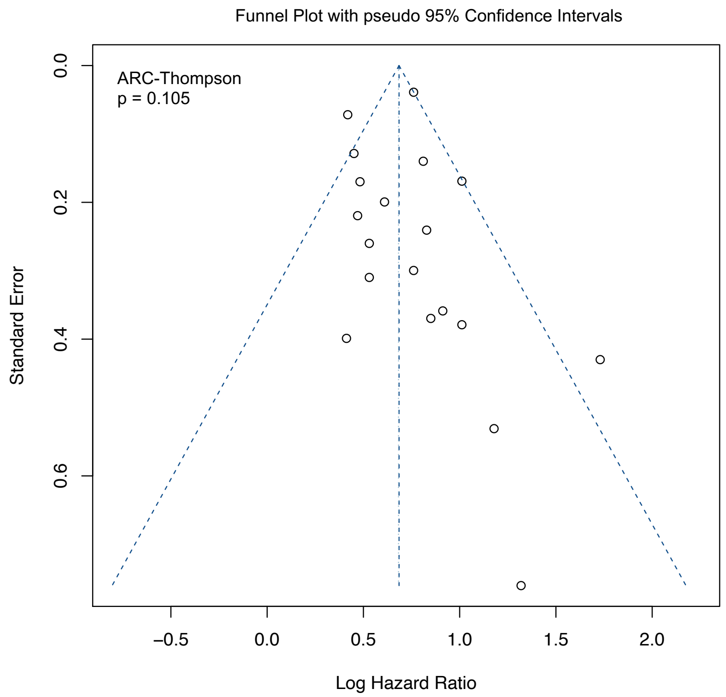

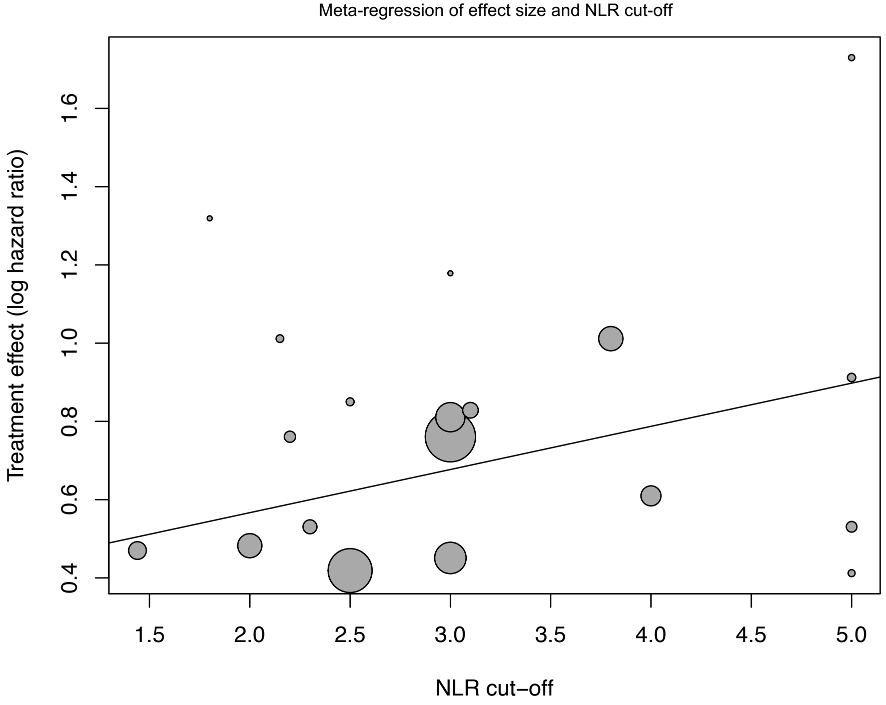

Supplementary Figure 21 - NLR: OS in gastric cancer with resection

Study	ES	seES	HR	95%–CI	Weight (fixed)	Weight (random)
Graziosi 2014	1.05	0.3317	2.85	[1.49; 5.46]	7.9%	13.2%
Hsu 2015	0.70	0.1525	2.01	[1.49; 2.71]	37.3%	16.8%
Jiang 2014	0.94	0.2766	2.57	[1.49; 4.42]	11.3%	14.4%
Lee 2013	0.59	0.3290	1.80	[0.94; 3.43]	8.0%	13.2%
Lian 2015	0.97	0.3478	2.65	[1.34; 5.24]	7.2%	12.8%
Shimada 2010	2.30	0.2224	10.01	[6.47; 15.48]	17.5%	15.5%
Yu 2015	1.35	0.2834	3.85	[2.21; 6.71]	10.8%	14.2%
**Pooled HR by Fixed Effects**			**3.05**	**[2.54; 3.66]**	**100.0%**	**--**
**Pooled HR by Random Effects**			**3.13**	**[1.99; 4.92]**	**--**	**100.0%**
**Prediction interval**				**[0.69; 14.24]**		

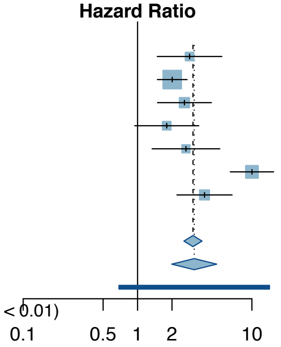

Heterogeneity: $I^2 = 85\%$ [71%; 92%], $\chi^2_6 = 39.86$ ($p < 0.01$)

Tau²= 0.542

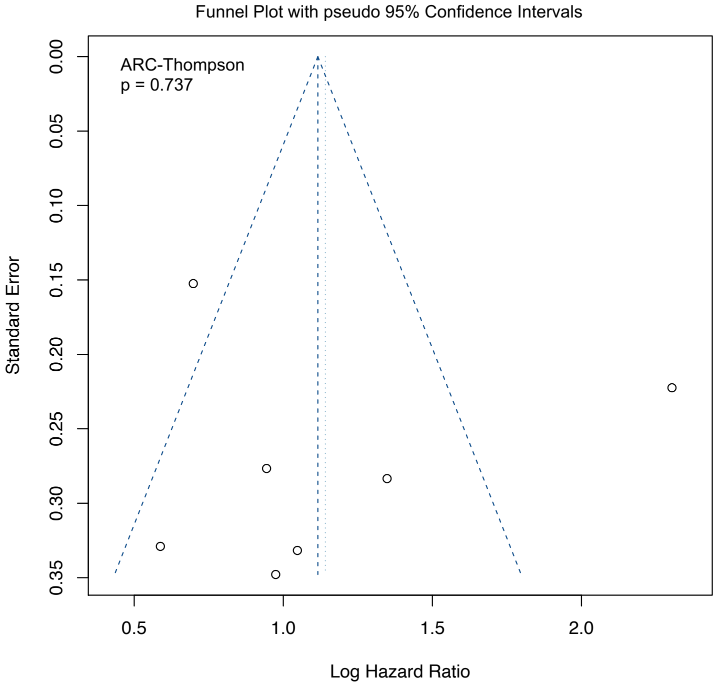

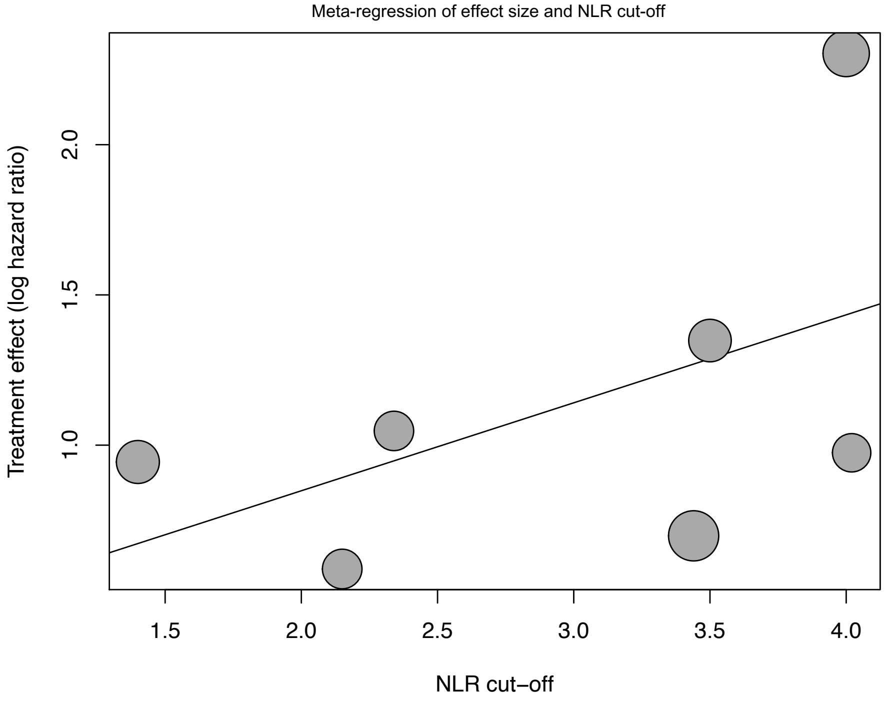

Supplementary Figure 22 - NLR: OS in gastrointestinal stromal cancer

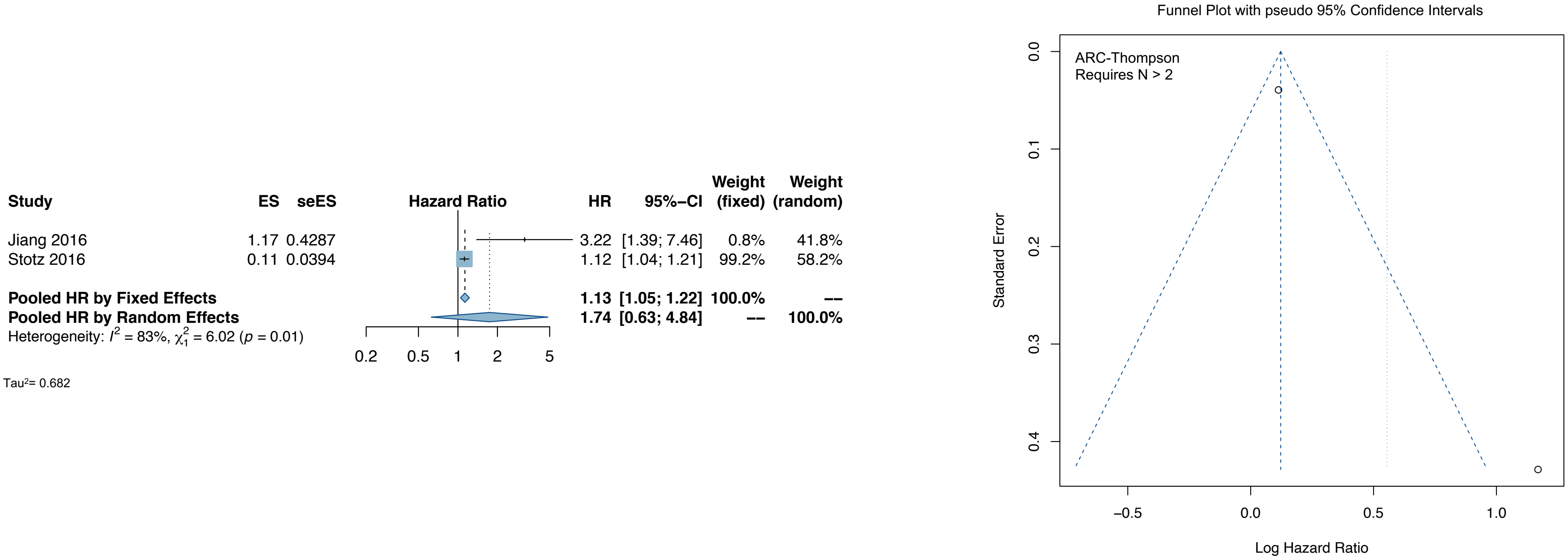

Supplementary Figure 23 - NLR: PFS in gastric cancers

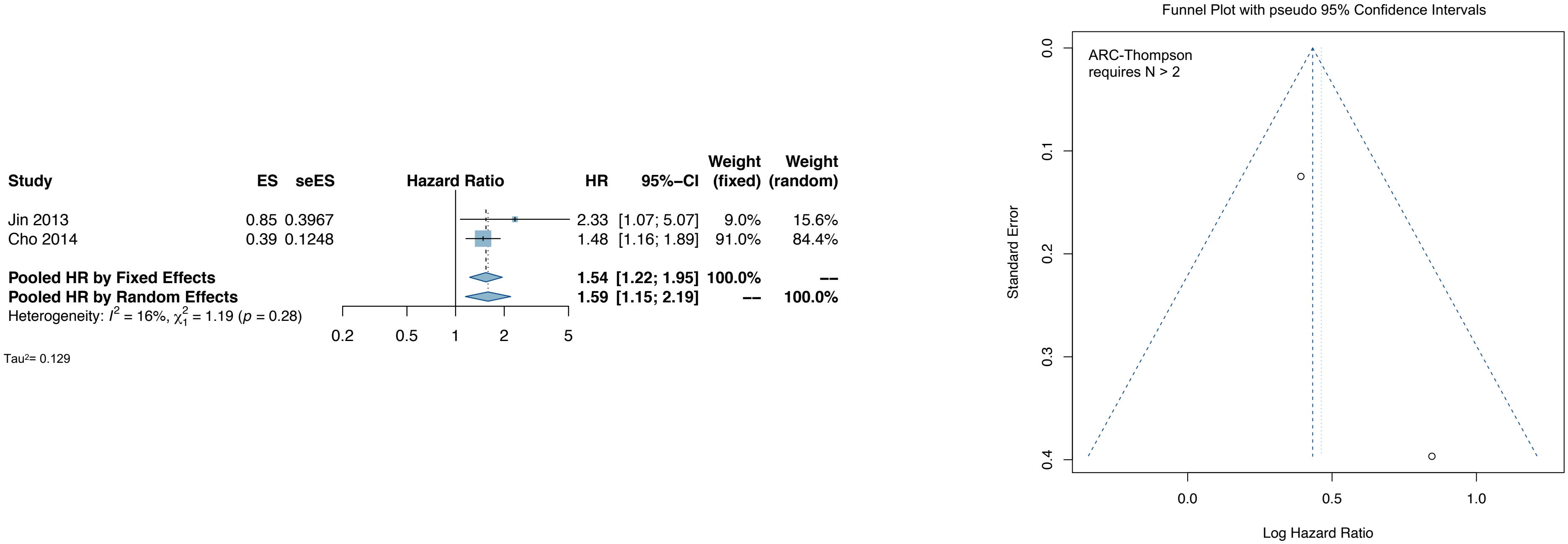

Supplementary Figure 24 - NLR: DFSin gastric cancers

Study	ES	seES	HR	95%−CI	Weight (fixed)	Weight (random)
Aurello 2014	−0.05	0.5097	0.95	[0.35; 2.58]	3.1%	3.1%
Cho 2014	0.39	0.1301	1.48	[1.15; 1.91]	47.0%	47.0%
El Aziz 2014	0.33	0.4697	1.39	[0.55; 3.49]	3.6%	3.6%
Jeong 2012	0.63	0.2087	1.88	[1.25; 2.83]	18.3%	18.3%
Jin 2013	0.85	0.3995	2.34	[1.07; 5.12]	5.0%	5.0%
Jung 2011	0.50	0.2100	1.65	[1.09; 2.49]	18.1%	18.1%
Kim and Choi 2012	0.28	0.4016	1.32	[0.60; 2.90]	4.9%	4.9%
**Pooled HR by Fixed Effects**			**1.58**	**[1.33; 1.88]**	**100.0%**	**--**
**Pooled HR by Random Effects**			**1.58**	**[1.33; 1.88]**	**--**	**100.0%**
**Prediction interval**				**[1.26; 1.99]**		

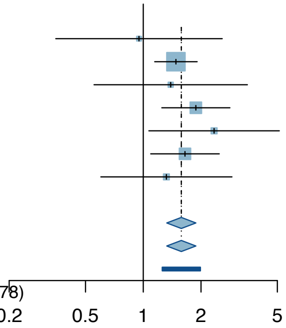

Heterogeneity: I^2 = 0% [0%; 46%], χ^2_6 = 3.23 (p = 0.78)

Tau^2= 0

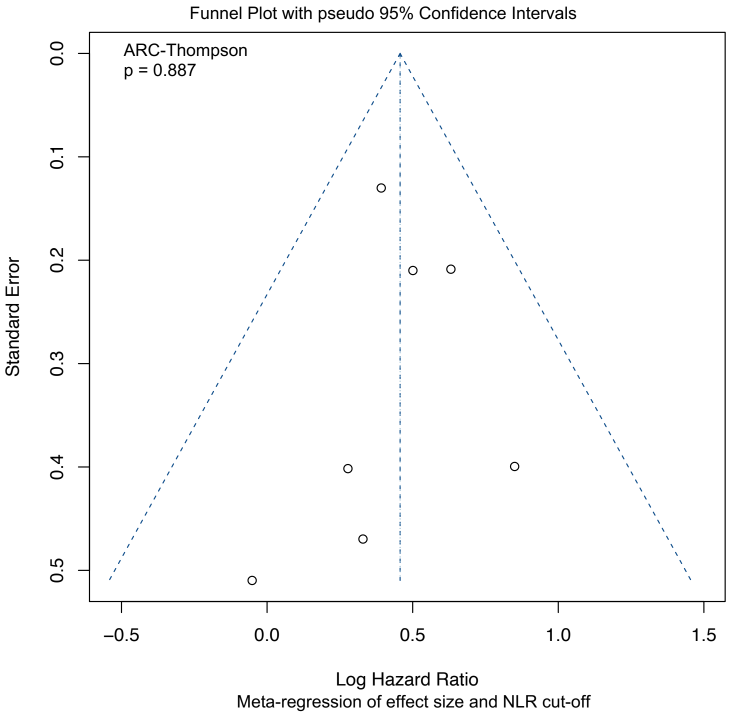

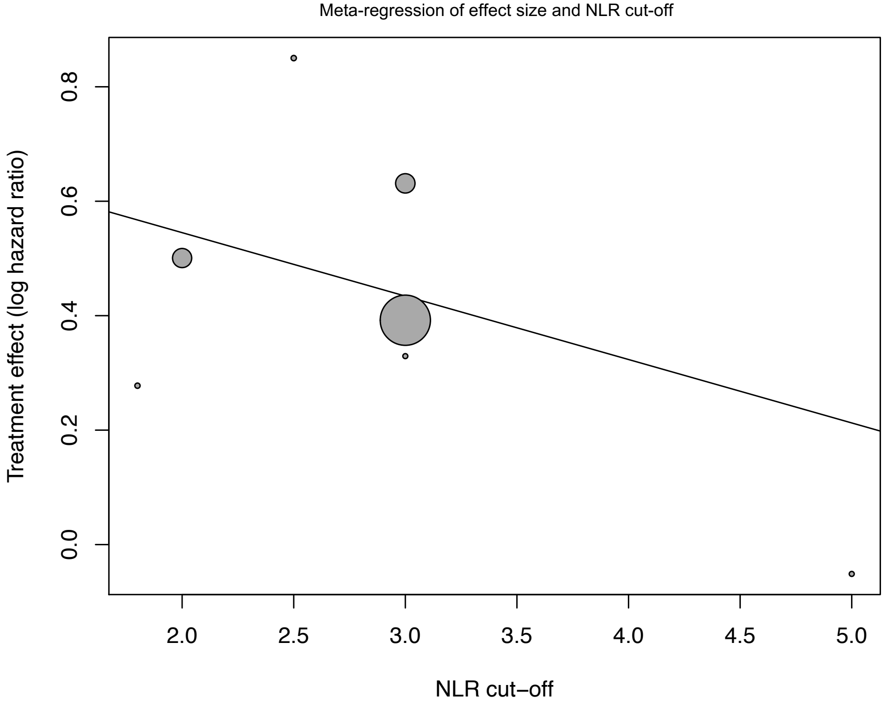

Supplementary Figure 25 - NLR: DFS in gastric cancers with resection

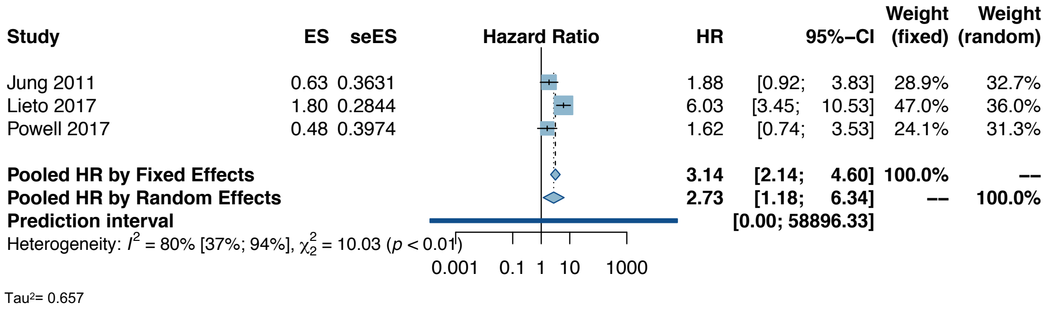

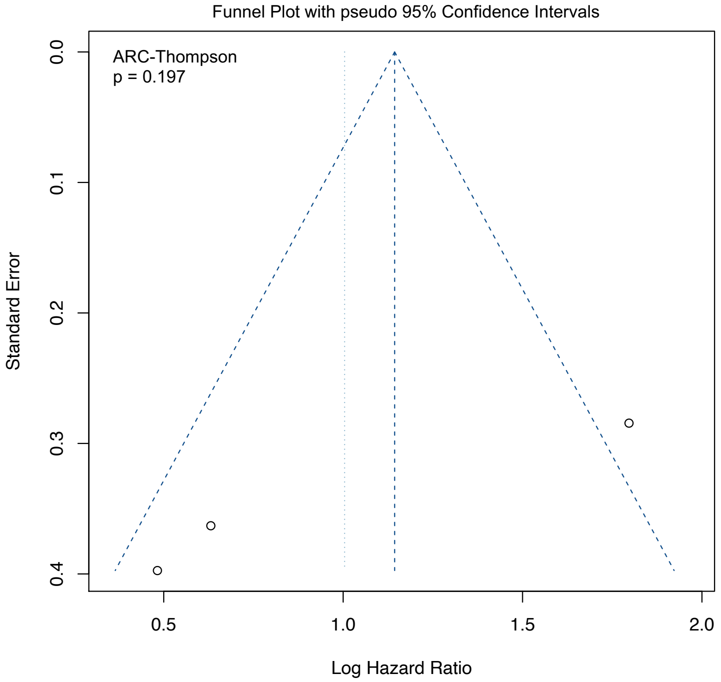

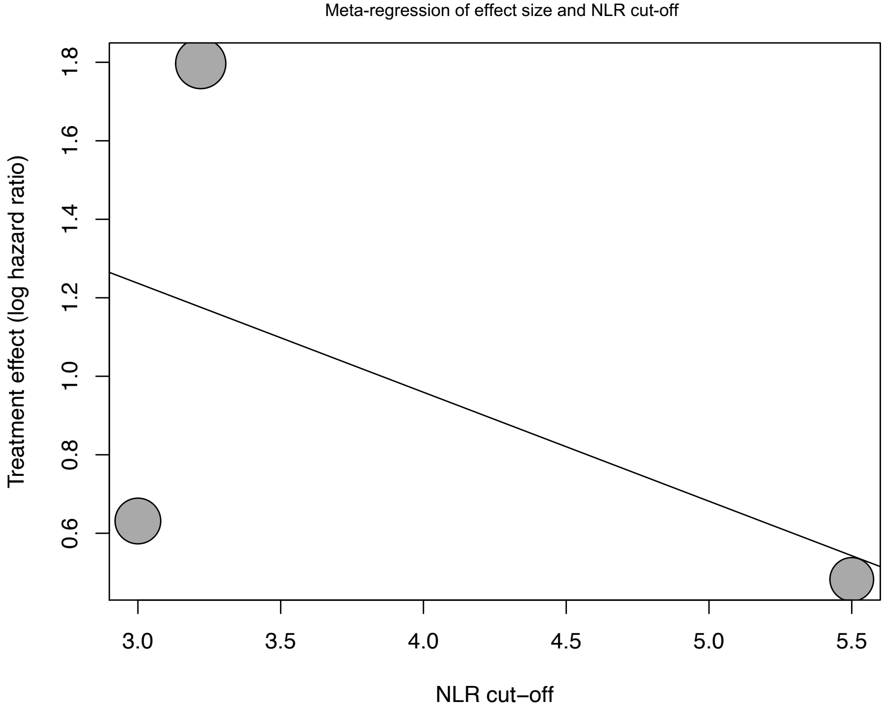

Supplementary Figure 26 - NLR: OS in colorectal cancer

Study	TE	seTE	HR	95%–CI	Weight (fixed)	Weight (random)
**Adjustment = Multivariate**						
Leitch 2007	0.52	0.3582	1.68	[0.83; 3.39]	1.7%	4.1%
Leitch 2007	0.30	0.2398	1.35	[0.84; 2.16]	3.8%	5.9%
Halazun 2007	0.82	0.1639	2.27	[1.65; 3.13]	8.1%	7.2%
Kishi 2009	0.79	0.3419	2.20	[1.13; 4.30]	1.9%	4.3%
Kishi 2009	1.06	0.3265	2.90	[1.53; 5.50]	2.0%	4.5%
Liu 2010	0.96	0.4187	2.61	[1.15; 5.93]	1.2%	3.4%
Chua 2011	0.53	0.1968	1.70	[1.16; 2.50]	5.6%	6.6%
Hung 2011	0.25	0.1700	1.29	[0.92; 1.80]	7.5%	7.1%
Zhang 2011	1.28	0.5055	3.59	[1.33; 9.67]	0.8%	2.6%
Carruthers 2012	1.95	0.5148	7.00	[2.55; 19.20]	0.8%	2.6%
Chiang 2012	0.27	0.0924	1.31	[1.09; 1.57]	25.4%	8.4%
Chiang 2012	0.18	0.1001	1.20	[0.99; 1.46]	21.6%	8.3%
Kaneko 2012	1.48	0.4236	4.39	[1.91; 10.07]	1.2%	3.3%
Kwon 2012	0.42	0.4635	1.52	[0.61; 3.77]	1.0%	3.0%
He 2013	0.39	0.1761	1.48	[1.05; 2.09]	7.0%	7.0%
Son 2013	0.61	0.6961	1.84	[0.47; 7.20]	0.4%	1.6%
East 2013	1.22	0.2099	3.40	[2.25; 5.13]	4.9%	6.4%
Absenger 2013	0.59	0.3480	1.81	[0.92; 3.58]	1.8%	4.2%
Toiyama 2013	−0.02	0.4899	0.98	[0.38; 2.56]	0.9%	2.8%
**Pooled HR by Fixed Effects**			**1.57**	**[1.43; 1.72]**	**97.6%**	**---**
**Pooled HR by Random Effects**			**1.88**	**[1.54; 2.30]**	**---**	**93.4%**
Heterogeneity: $I^2 = 68\%$ [48%; 80%], $\chi^2_{18} = 55.85$ ($p < 0.01$)						
**Adjustment = Univariate**						
Guthrie 2013	1.12	0.4645	3.07	[1.24; 7.63]	1.0%	3.0%
Paik 2014	0.79	0.3959	2.20	[1.01; 4.78]	1.4%	3.6%
**Pooled HR by Fixed Effects**			**2.53**	**[1.40; 4.57]**	**2.4%**	**---**
**Pooled HR by Random Effects**			**2.53**	**[1.40; 4.57]**	**---**	**6.6%**
Heterogeneity: $I^2 = 0\%$, $\chi^2_1 = 0.3$ ($p = 0.59$)						
**Pooled HR by Fixed Effects**			**1.59**	**[1.45; 1.74]**	**100.0%**	**---**
**Pooled HR by Random Effects**			**1.92**	**[1.58; 2.32]**	**---**	**100.0%**
**Prediction interval**				**[0.95; 3.88]**		
Heterogeneity: $I^2 = 66\%$ [46%; 78%], $\chi^2_{20} = 58.60$ ($p < 0.01$)						

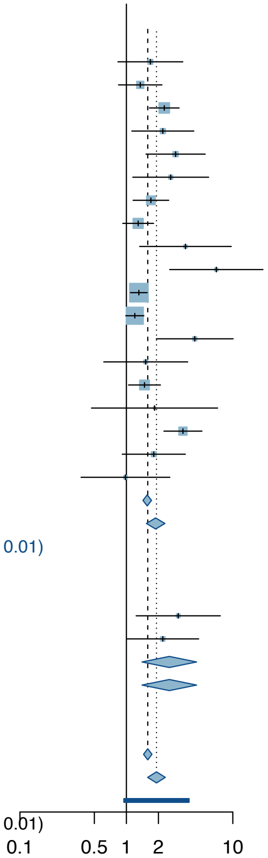

Tau²= 0.322

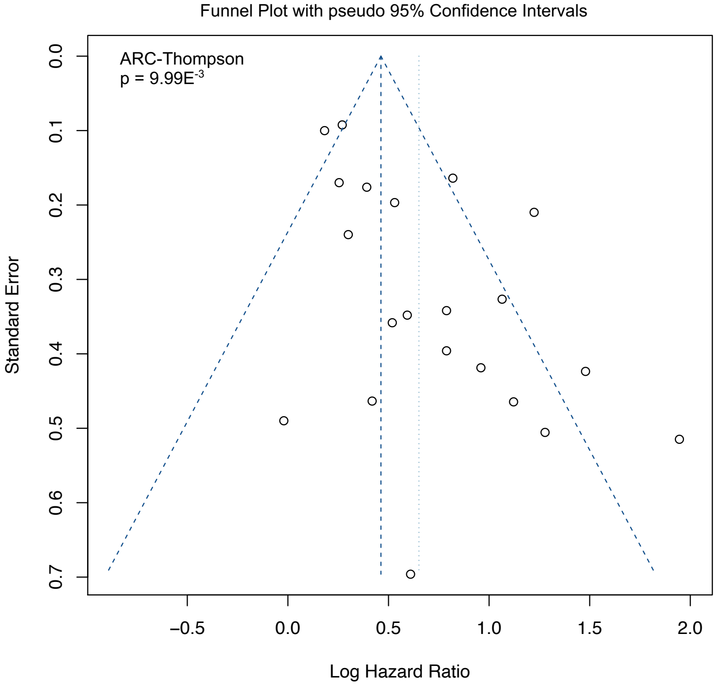

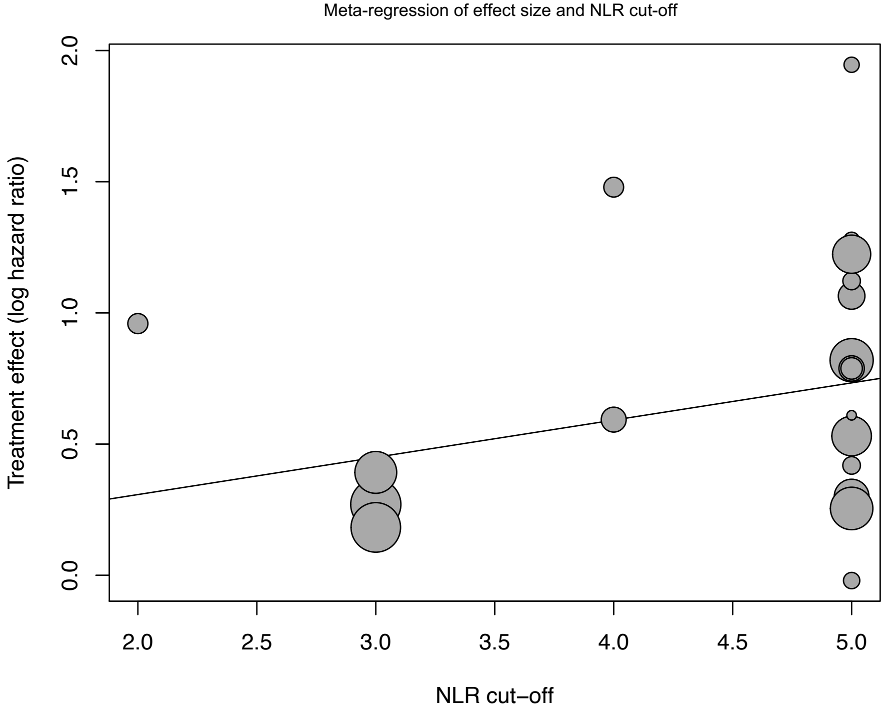

Supplementary Figure 27 - NLR: DFS in colorectal cancer with palliative chemotherapy

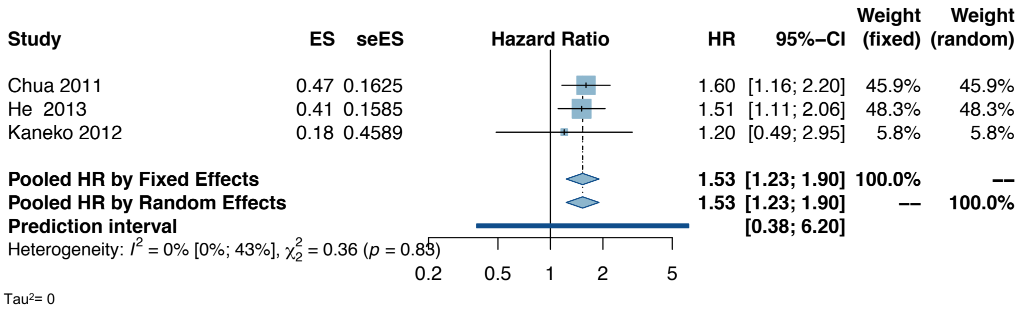

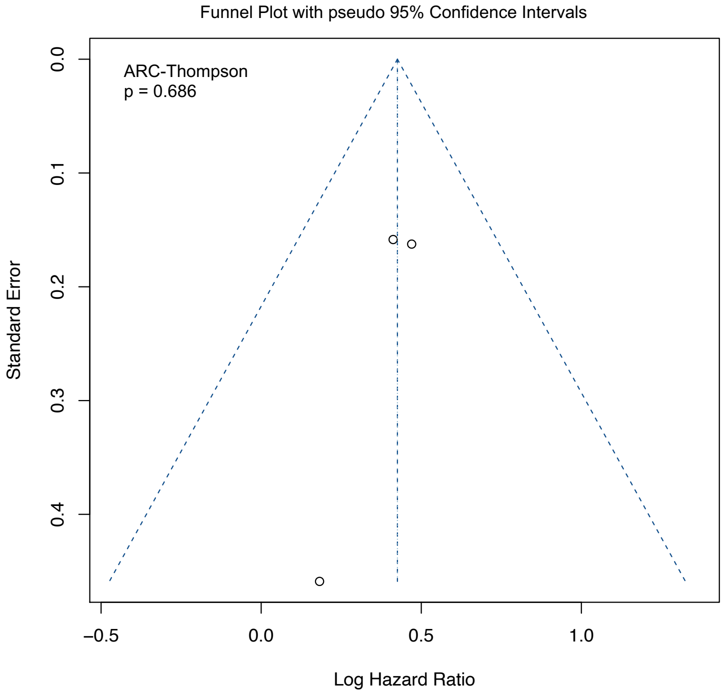

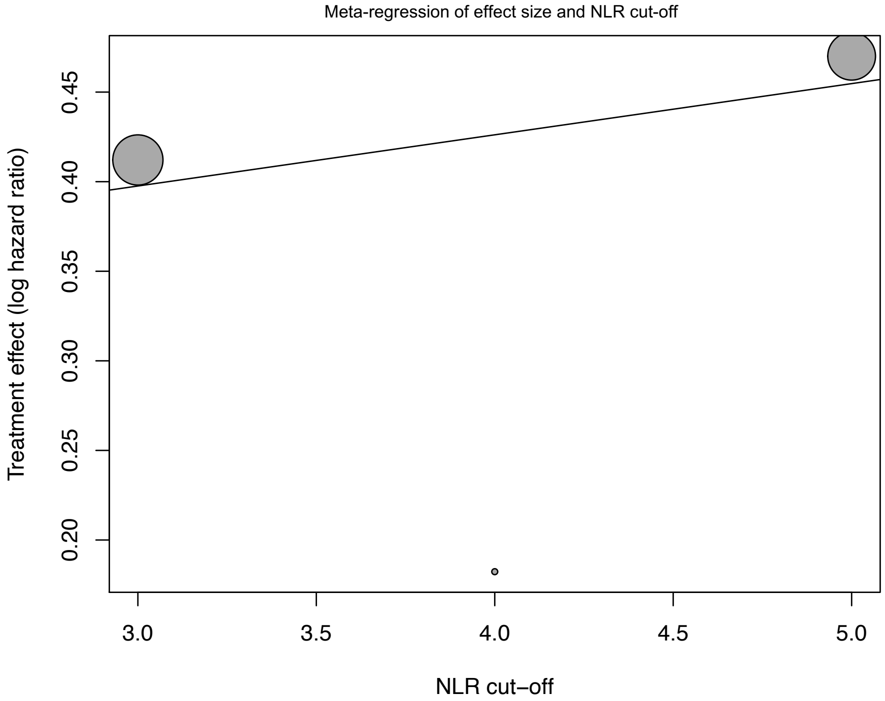

Supplementary *Figure 28* - NLR: PFS in colorectal cancer

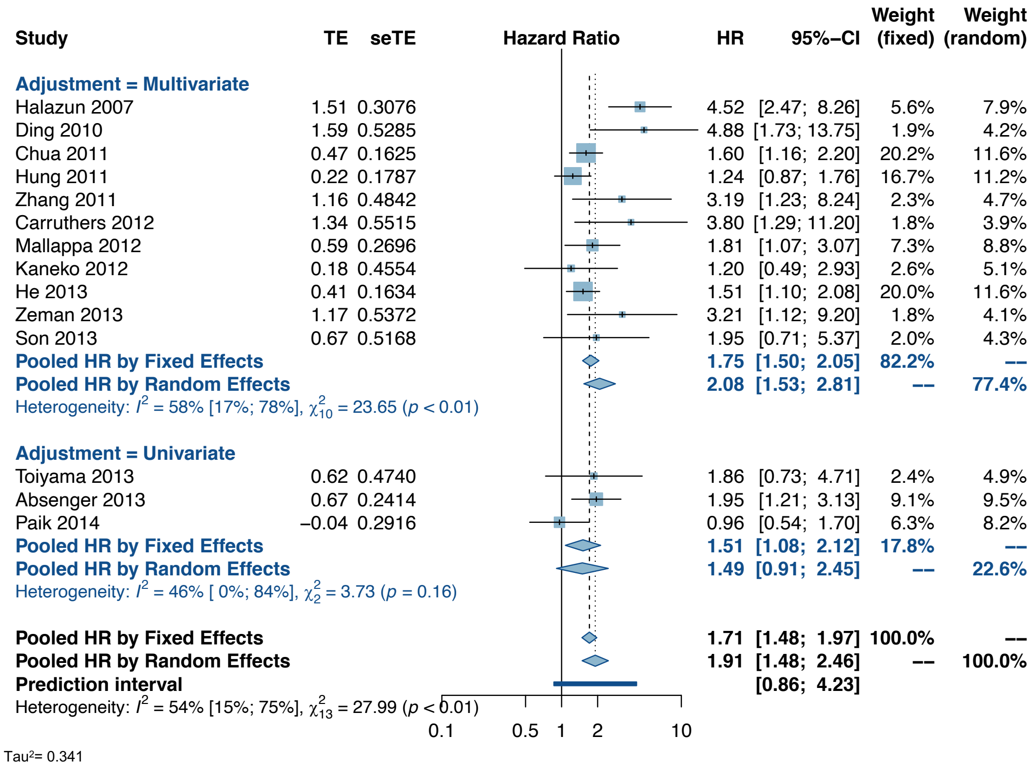

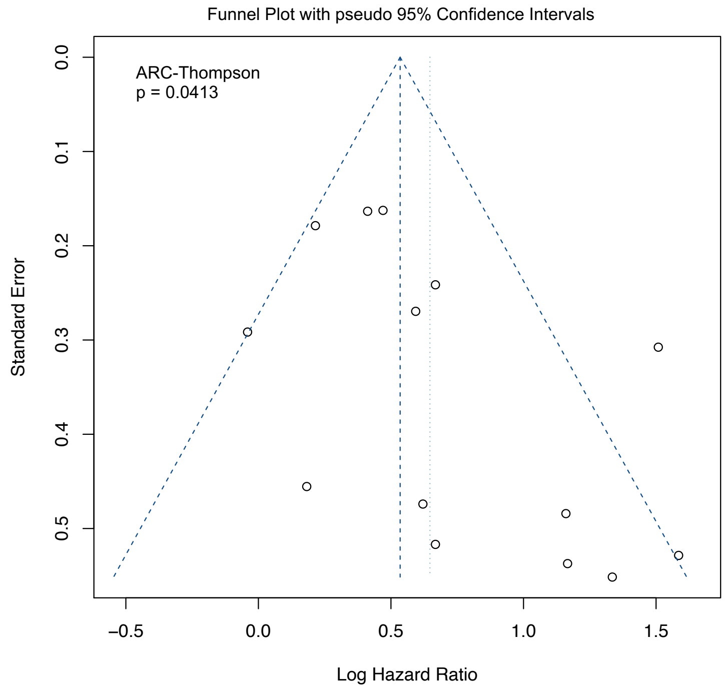

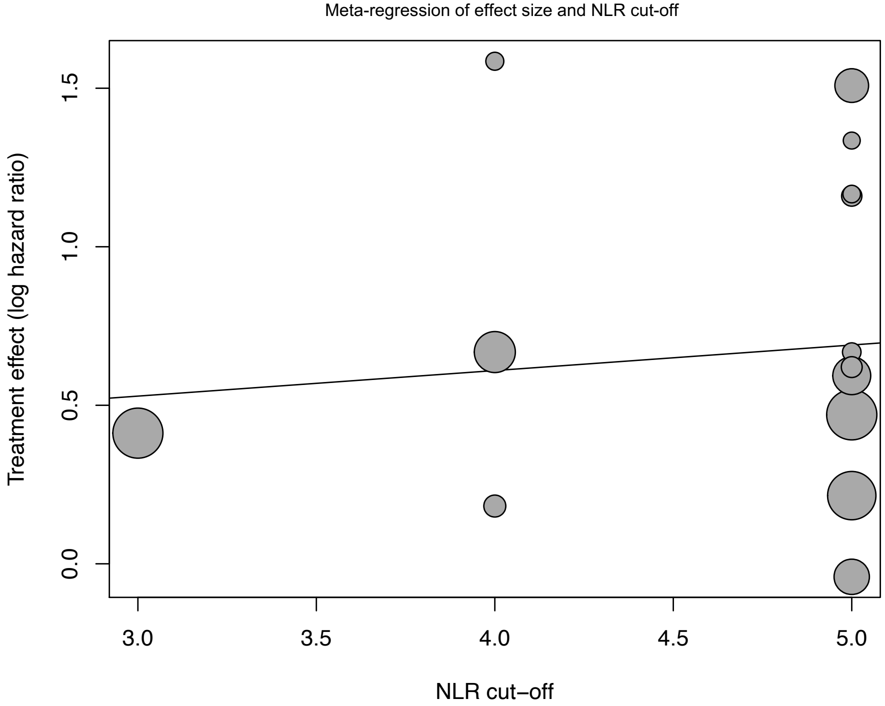

Supplementary *Figure 29*- NLR: DFS in colorectal cancer

Study	ES	seES	HR	95%–CI	Weight (fixed)	Weight (random)
Carruthers 2012	1.03	0.3188	2.80	[1.50; 5.23]	14.7%	17.9%
Hung 2011	0.22	0.1873	1.24	[0.86; 1.79]	42.6%	32.4%
Neal 2009	0.67	0.2527	1.95	[1.19; 3.20]	23.4%	24.0%
Son 2013	0.67	0.5152	1.96	[0.71; 5.38]	5.6%	8.5%
Sun 2014	0.53	0.3306	1.70	[0.89; 3.25]	13.7%	17.1%
**Pooled HR by Fixed Effects**			**1.66**	**[1.31; 2.12]**	**100.0%**	**--**
**Pooled HR by Random Effects**			**1.76**	**[1.27; 2.42]**	**--**	**100.0%**
**Prediction interval**				**[0.74; 4.17]**		

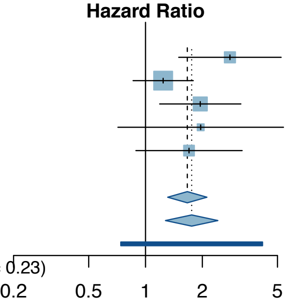

Heterogeneity: $I^2 = 29\%$ [0%; 72%], $\chi^2_4 = 5.63$ ($p = 0.23$)

Tau²= 0.217

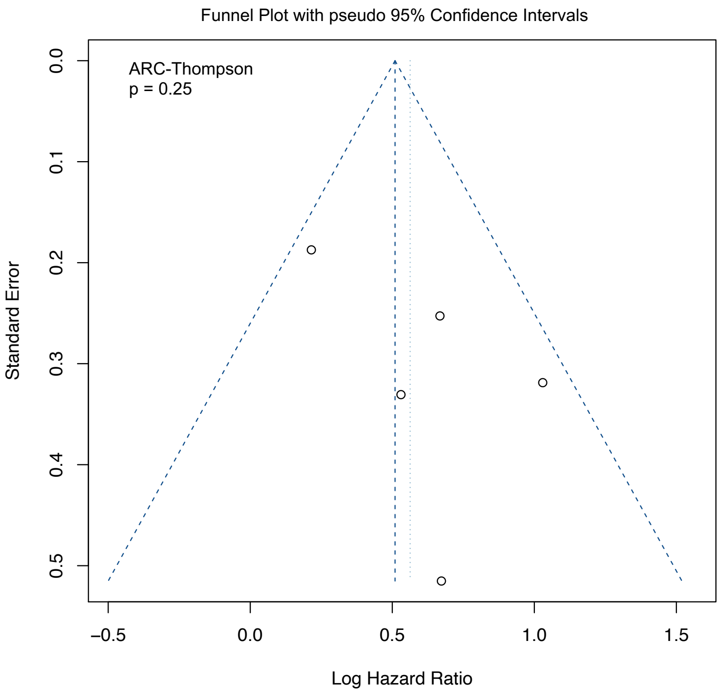

Supplementary *Figure 30* - NLR: OS in rectal cancer

Study	ES	seES	HR	95%-CI	Weight (fixed)	Weight (random)
Shen 2014	2.12	0.9339	8.36	[1.34; 52.14]	54.0%	54.0%
Toiyama 2015	2.85	2.1887	17.29	[0.24; 1261.33]	9.8%	9.8%
Carruthers 2012	7.00	3.8749	1096.63	[0.55; 2179695.79]	3.1%	3.1%
Nagasaki 2015	3.38	2.7398	29.40	[0.14; 6316.38]	6.3%	6.3%
Liu 2010	2.62	1.6927	13.67	[0.50; 377.26]	16.4%	16.4%
Harji 2012	3.40	2.1378	29.96	[0.45; 1978.16]	10.3%	10.3%
**Pooled HR by Fixed Effects**			**14.00**	**[3.65; 53.75]**	**100.0%**	**--**
**Pooled HR by Random Effects**			**14.00**	**[3.65; 53.75]**	**--**	**100.0%**
**Prediction interval**				**[2.08; 94.14]**		

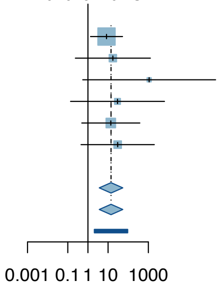

Heterogeneity: $I^2 = 0\%$ [0%; 29%], $\chi^2_5 = 1.78$ ($p = 0.88$)

Tau²= 0

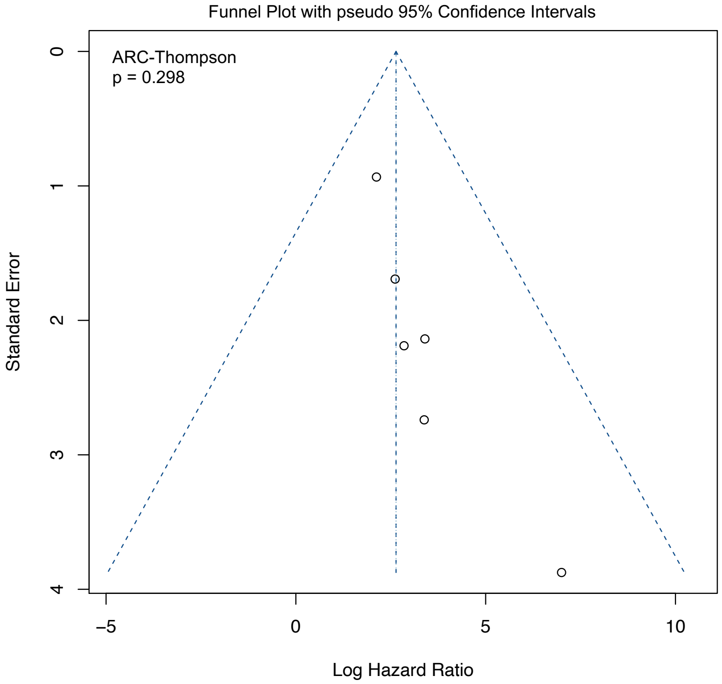

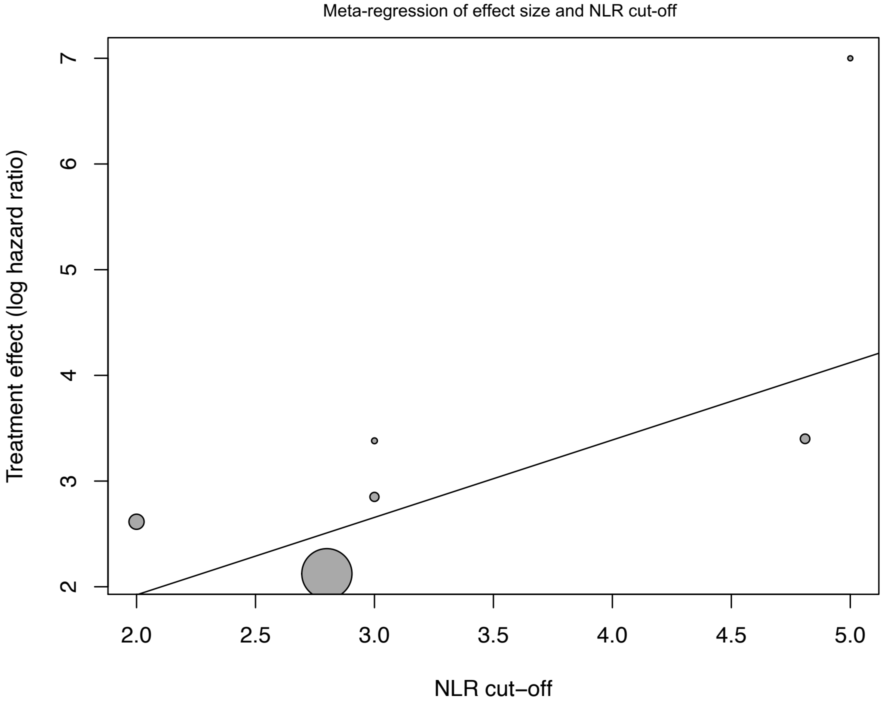

Supplementary *Figure 31* - NLR: DFS in rectal cancer

Study	ES	seES	HR	95%-CI	Weight (fixed)	Weight (random)
Shen 2014	1.36	0.4338	3.91	[1.67; 9.15]	87.0%	87.0%
Toiyama 2015	1.69	1.2194	5.42	[0.50; 59.15]	11.0%	11.0%
Carruthers 2012	4.10	2.9082	60.34	[0.20; 18031.85]	1.9%	1.9%
**Pooled HR by Fixed Effects**			**4.27**	**[1.93; 9.45]**	**100.0%**	**--**
**Pooled HR by Random Effects**			**4.27**	**[1.93; 9.45]**	**--**	**100.0%**
**Prediction interval**				**[0.02; 731.37]**		

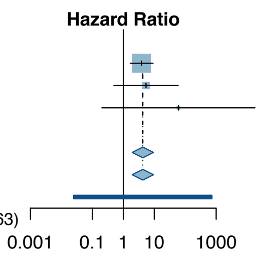

Heterogeneity: $I^2 = 0\%$ [0%; 77%], $\chi^2_2 = 0.91$ ($p = 0.63$)

Tau²= 0.0

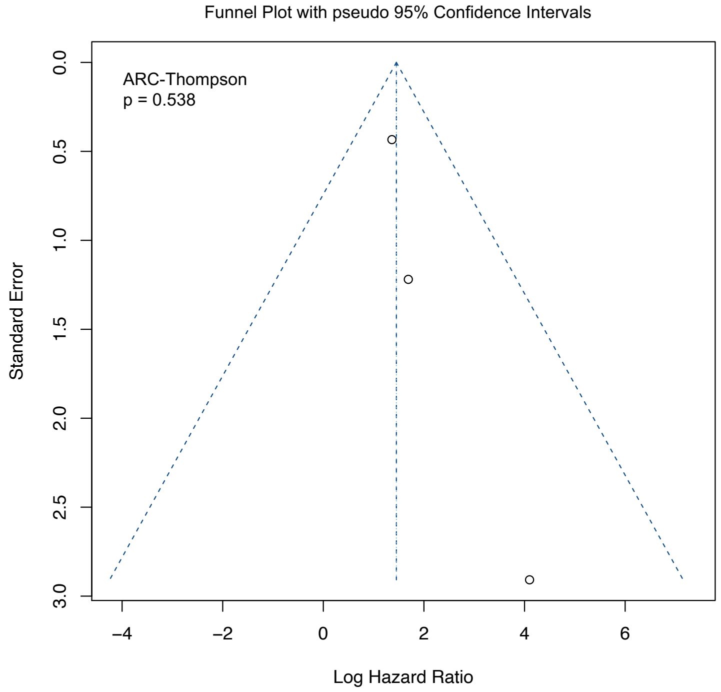

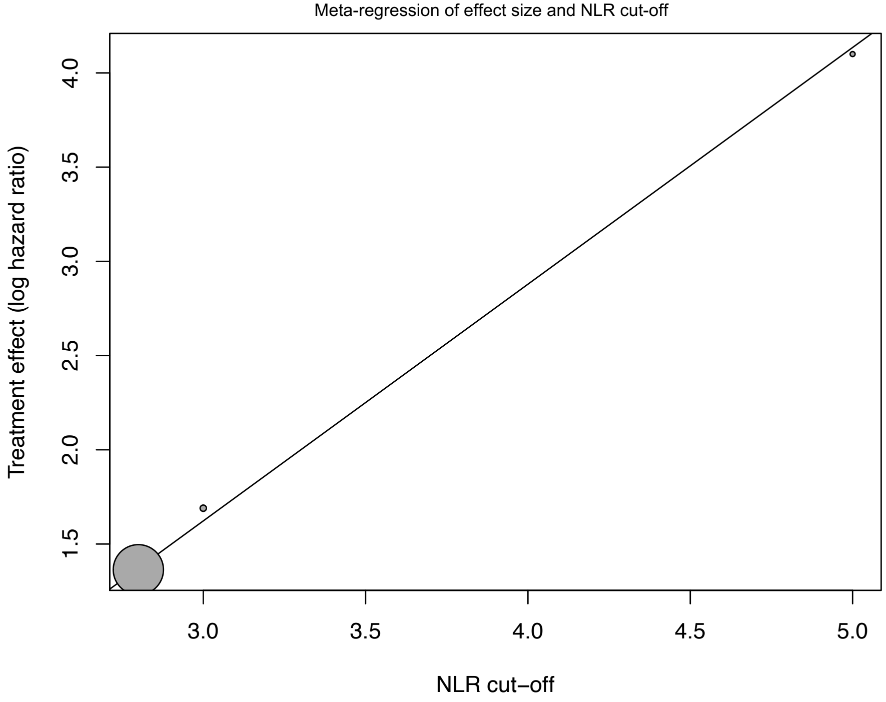

Supplementary Figure 32 - NLR: RFS in rectal cancer

Study	ES	seES	HR	95%–CI	Weight (fixed)	Weight (random)
Nagasaki 2015	1.07	0.5594	2.92	[0.98; 8.74]	66.2%	66.2%
Kim 2014	2.80	2.0410	16.44	[0.30; 897.78]	5.0%	5.0%
Harji 2012	1.50	0.8471	4.48	[0.85; 23.57]	28.9%	28.9%
**Pooled HR by Fixed Effects**			**3.60**	**[1.48; 8.78]**	**100.0%**	**--**
**Pooled HR by Random Effects**			**3.60**	**[1.48; 8.78]**	**--**	**100.0%**
**Prediction interval**				**[0.01; 1167.53]**		

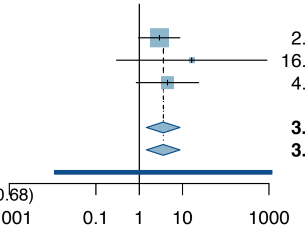

Heterogeneity: $I^2 = 0\%$ [0%; 73%], $\chi^2_2 = 0.76$ ($p = 0.68$)

Tau²= 0

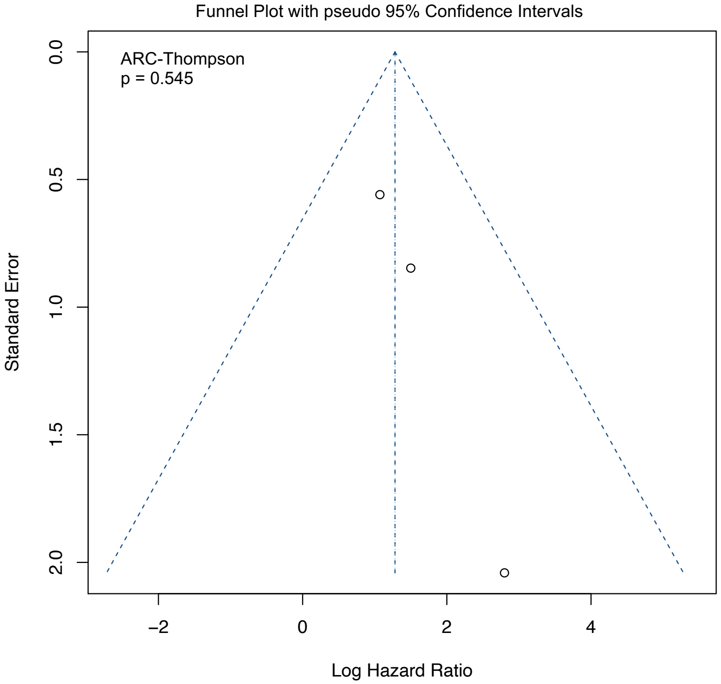

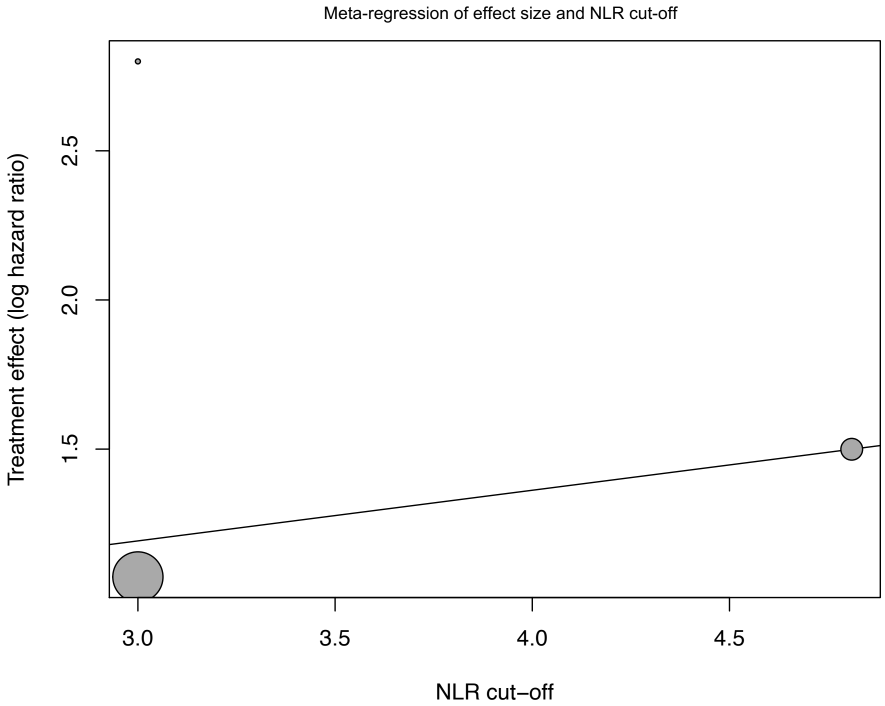

Supplementary *Figure 33* - NLR: OS in colorectal liver metastasis

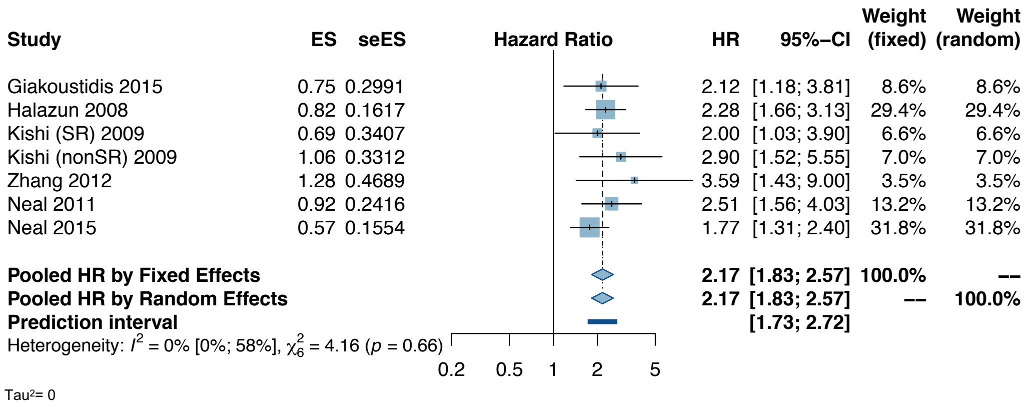

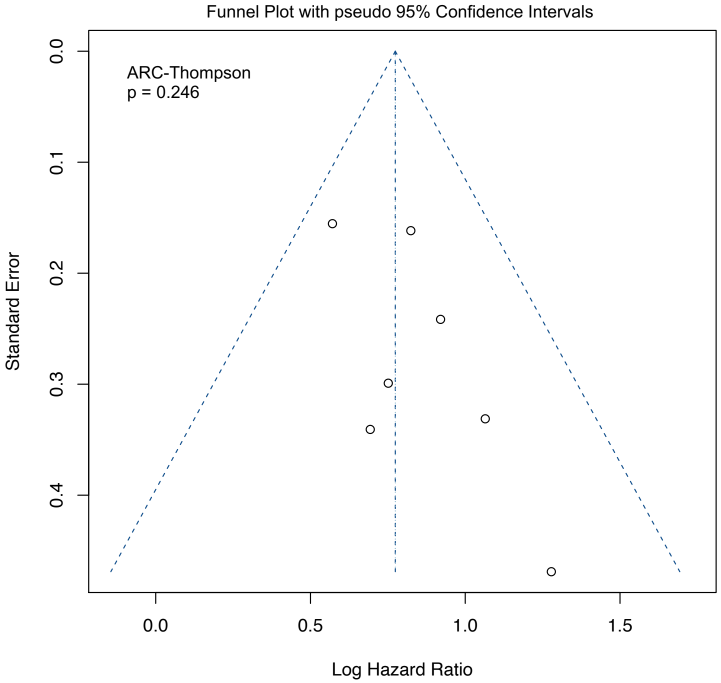

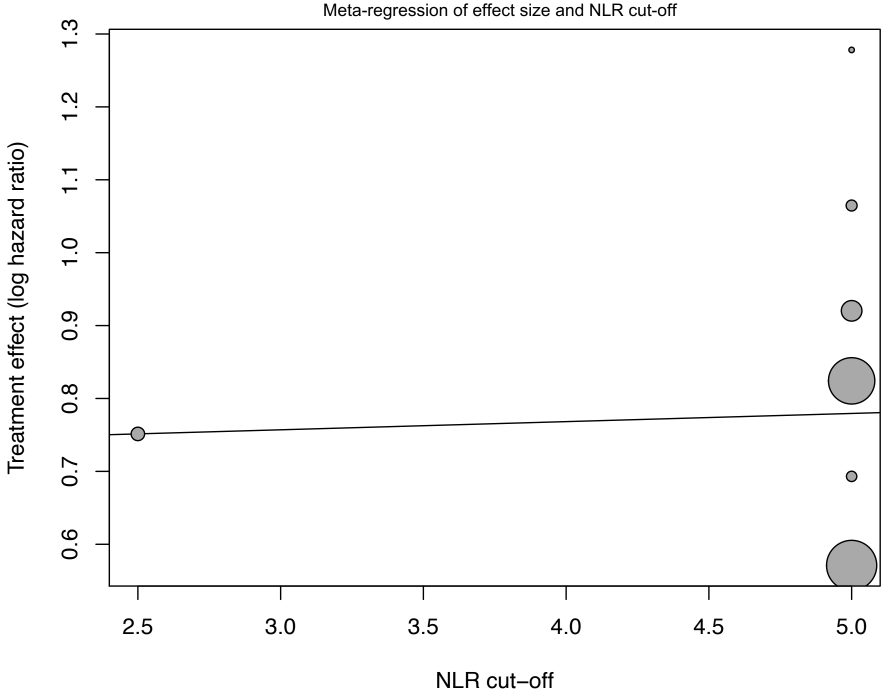

Supplementary *Figure 34* - NLR: OS in colorectal liver metastasis with surgical resection

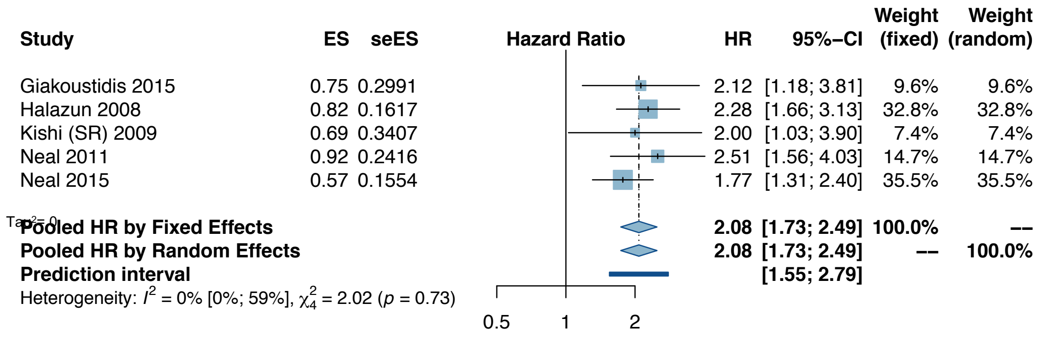

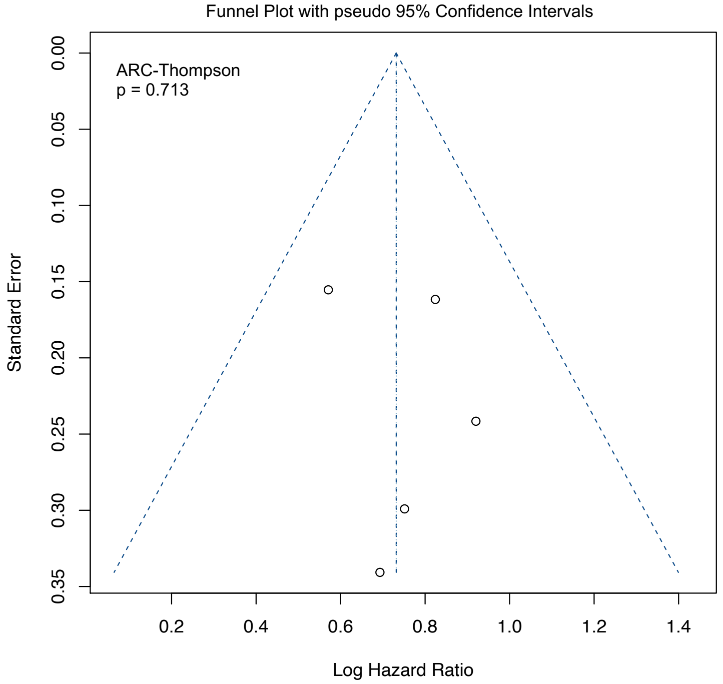

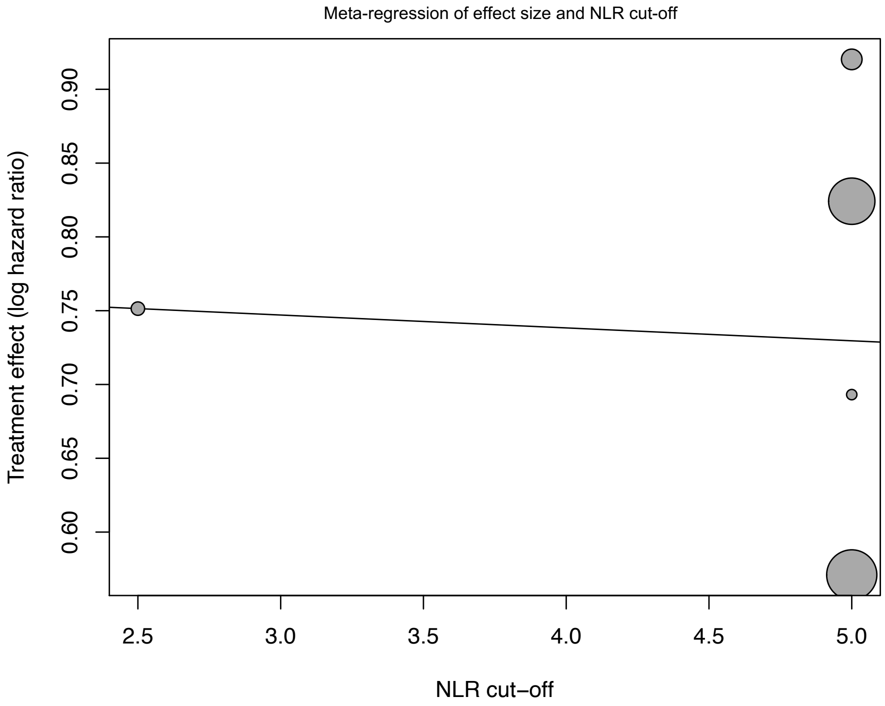

Supplementary *Figure 35* - NLR: OS in colorectal liver metastasis with non-surgical treatment

Study	ES	seES	Hazard Ratio	HR	95%−CI	Weight (fixed)	Weight (random)
Kishi (nonSR) 2009	1.06	0.3312		2.90	[1.52; 5.55]	66.7%	66.7%
Zhang 2012	1.28	0.4689		3.59	[1.43; 9.00]	33.3%	33.3%
**Pooled HR by Fixed Effects**				**3.11**	**[1.83; 5.29]**	**100.0%**	**--**
**Pooled HR by Random Effects**				**3.11**	**[1.83; 5.29]**	**--**	**100.0%**

Heterogeneity: $I^2 = 0\%$, $\chi^2_1 = 0.14$ ($p = 0.71$)

Tau²= 0

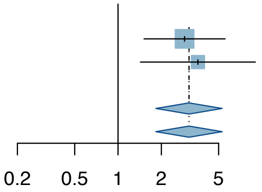

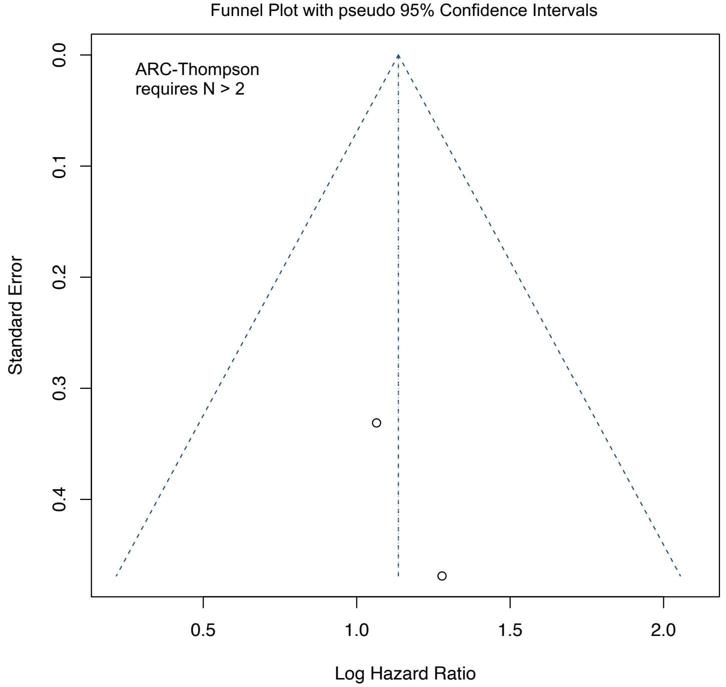

Supplementary *Figure 36* - NLR: RFS in colorectal liver metastasis

Study	ES	seES	HR	95%-CI	Weight (fixed)	Weight (random)
Chang 2014	0.51	0.3876	1.67	[0.78; 3.57]	5.7%	10.4%
Giakoustidis 2015	0.19	0.2026	1.21	[0.81; 1.80]	20.8%	22.3%
Neal 2011	0.72	0.2192	2.05	[1.33; 3.15]	17.8%	20.8%
Halazun 2008	0.79	0.1351	2.21	[1.70; 2.88]	46.8%	29.3%
Zeman 2013	1.17	0.5350	3.21	[1.12; 9.16]	3.0%	6.3%
Zhang 2012	1.16	0.3786	3.19	[1.52; 6.70]	6.0%	10.8%
**Pooled HR by Fixed Effects**			**1.96**	**[1.63; 2.35]**	**100.0%**	**--**
**Pooled HR by Random Effects**			**1.97**	**[1.48; 2.62]**	**--**	**100.0%**
**Prediction interval**				**[0.91; 4.23]**		

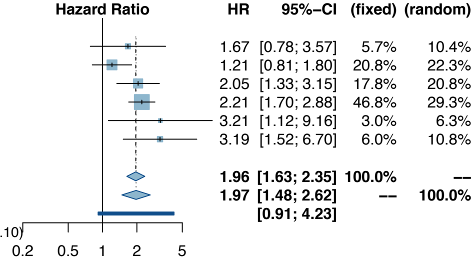

Heterogeneity: I^2 = 45% [0%; 78%], χ^2_5 = 9.17 (p = 0.10)

Tau²= 0.234

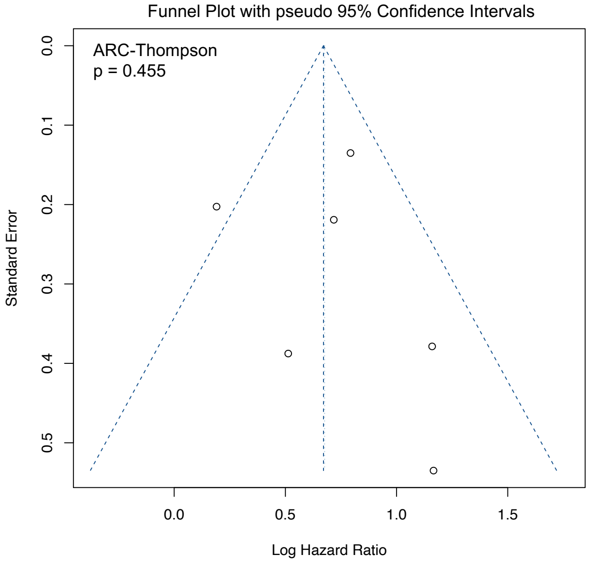

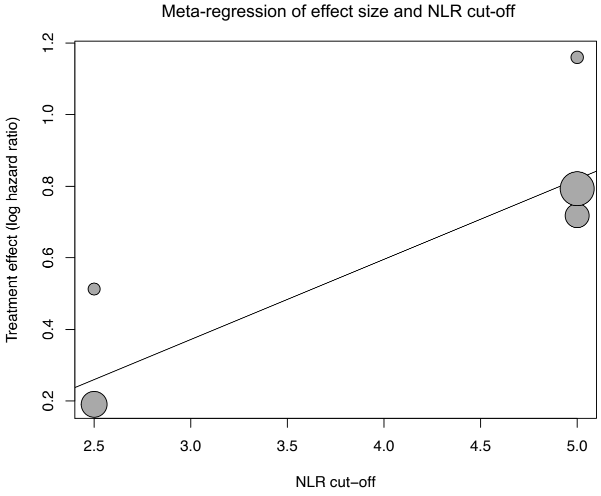

Supplementary *Figure 37* - NLR: RFS in colorectal liver metastasis with surgical resection

Study	ES	seES	Hazard Ratio	HR	95%–CI	Weight (fixed)	Weight (random)
Giakoustidis 2015	0.19	0.2026		1.21	[0.81; 1.80]	23.5%	28.5%
Neal 2011	0.72	0.2192		2.05	[1.33; 3.15]	20.1%	26.7%
Halazun 2008	0.79	0.1351		2.21	[1.70; 2.88]	53.0%	36.1%
Zeman 2013	1.17	0.5350		3.21	[1.12; 9.16]	3.4%	8.7%
**Pooled HR by Fixed Effects**				**1.91**	**[1.58; 2.32]**	**100.0%**	**--**
**Pooled HR by Random Effects**				**1.89**	**[1.34; 2.66]**	**--**	**100.0%**
**Prediction interval**					**[0.49; 7.25]**		

Heterogeneity: $I^2 = 59\%$ [0%; 86%], $\chi^2_3 = 7.29$ ($p = 0.06$)

Tau²= 0.259

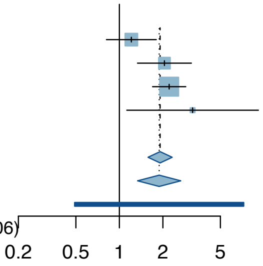

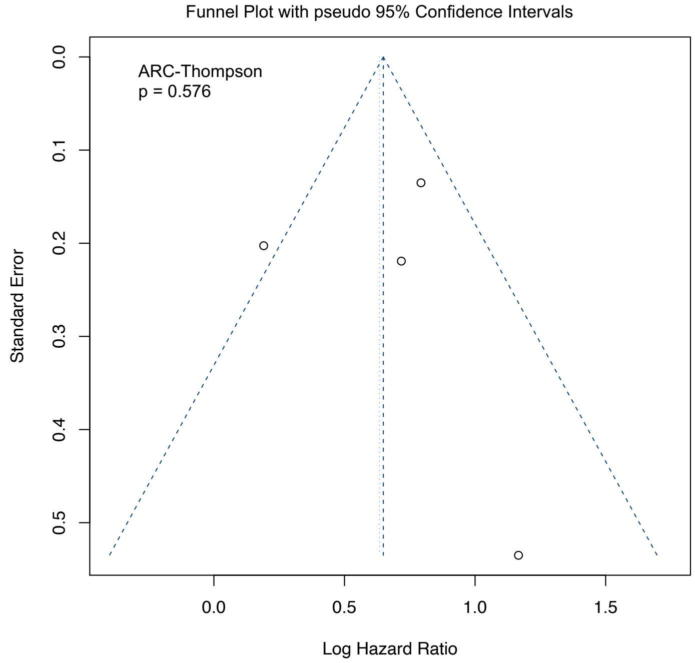

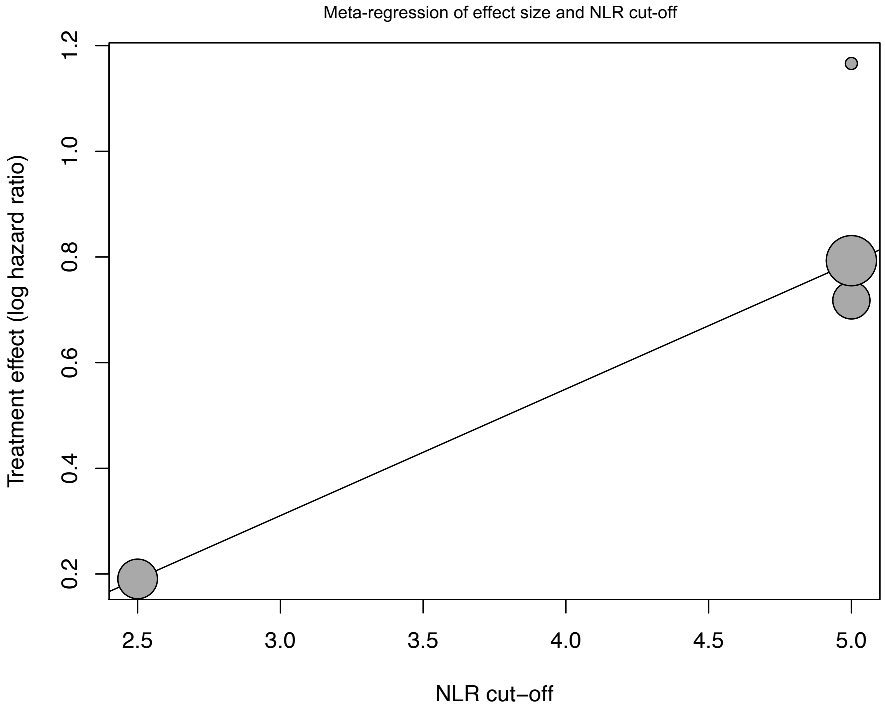

Supplementary *Figure 38* - NLR: RFS in colorectal liver metastasis with non-surgical treatment

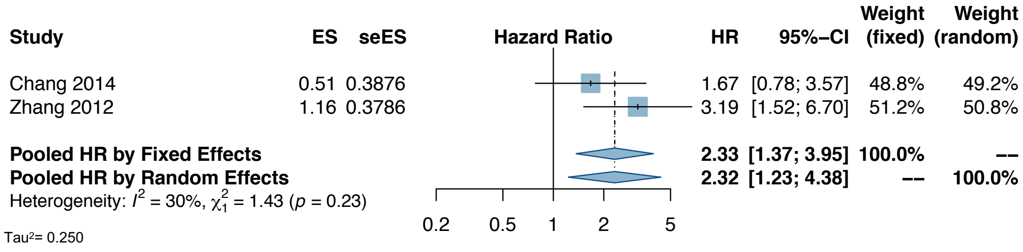

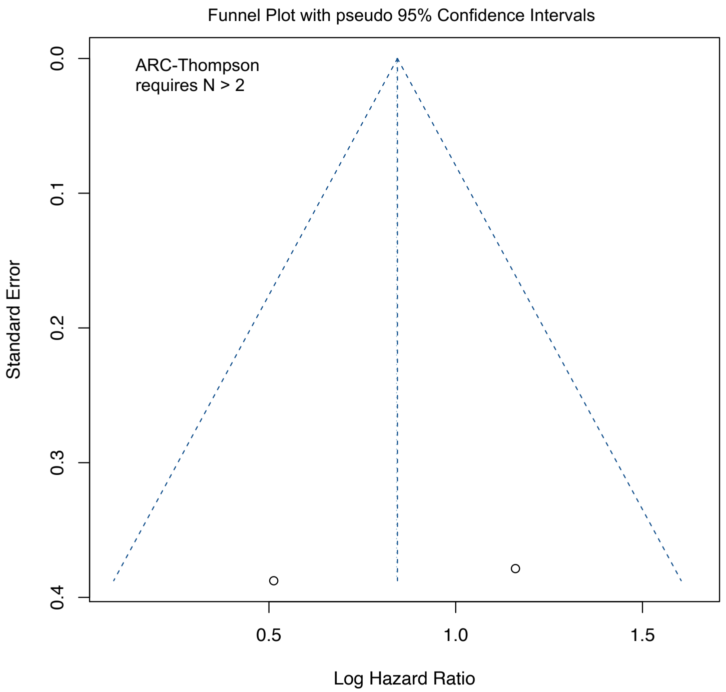

Supplementary *Figure 39* - NLR: OS in pancreatic cancer

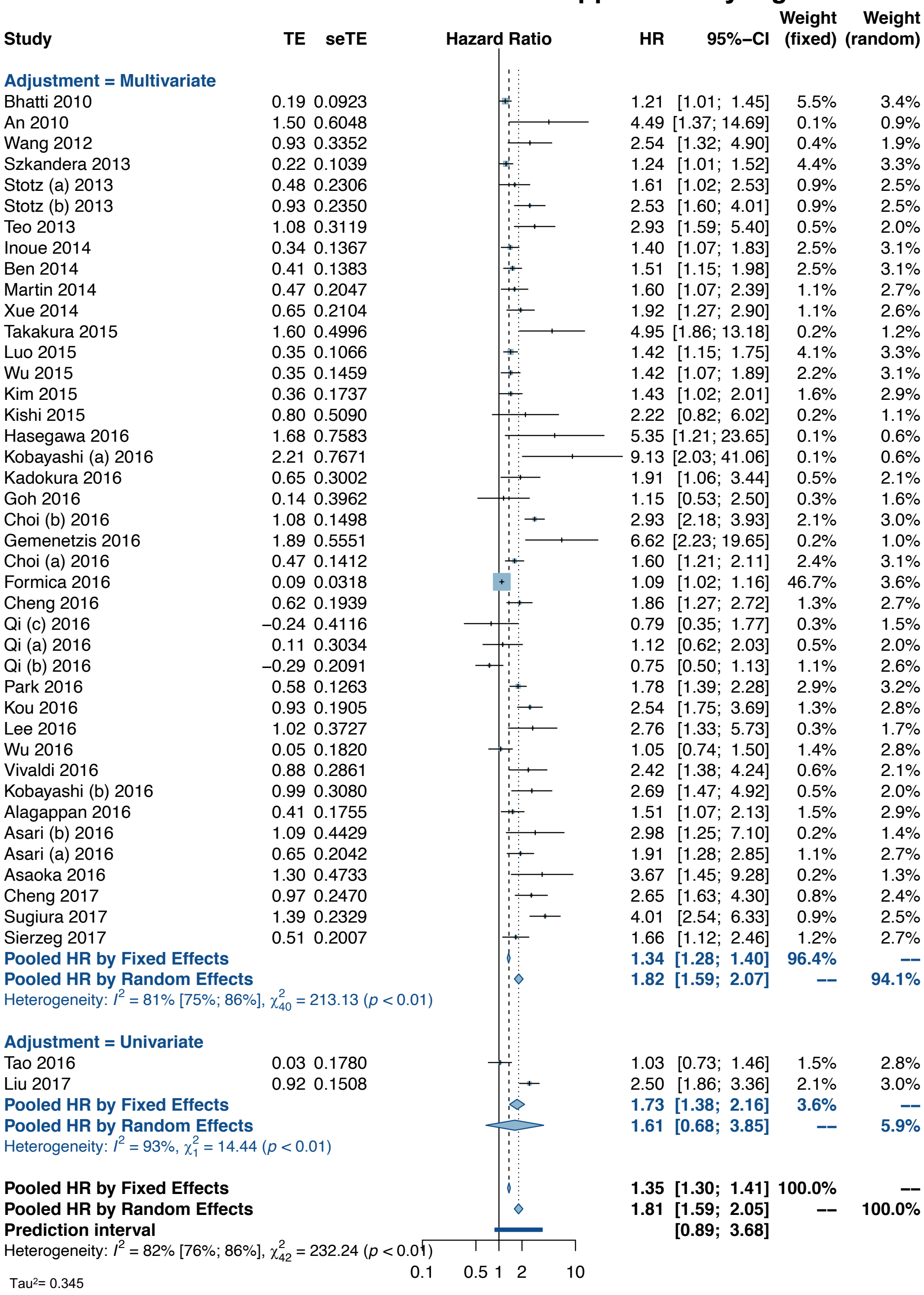

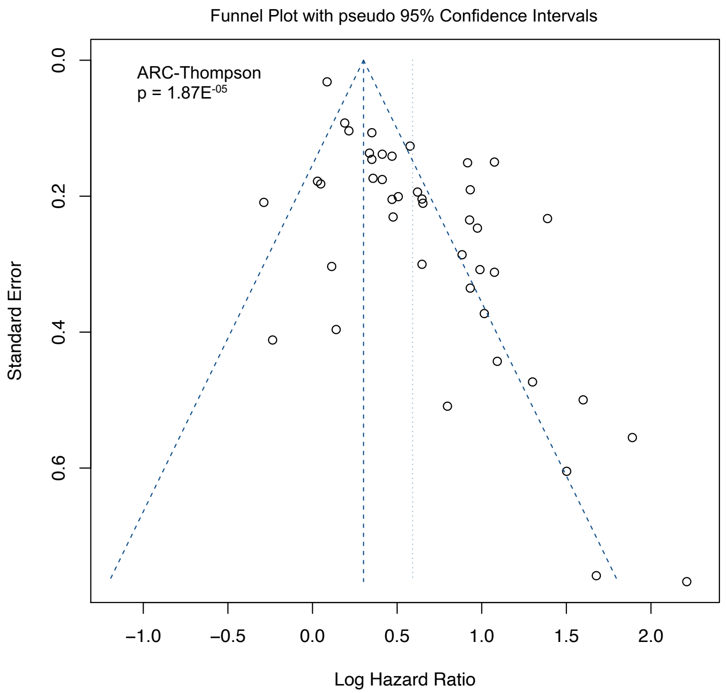

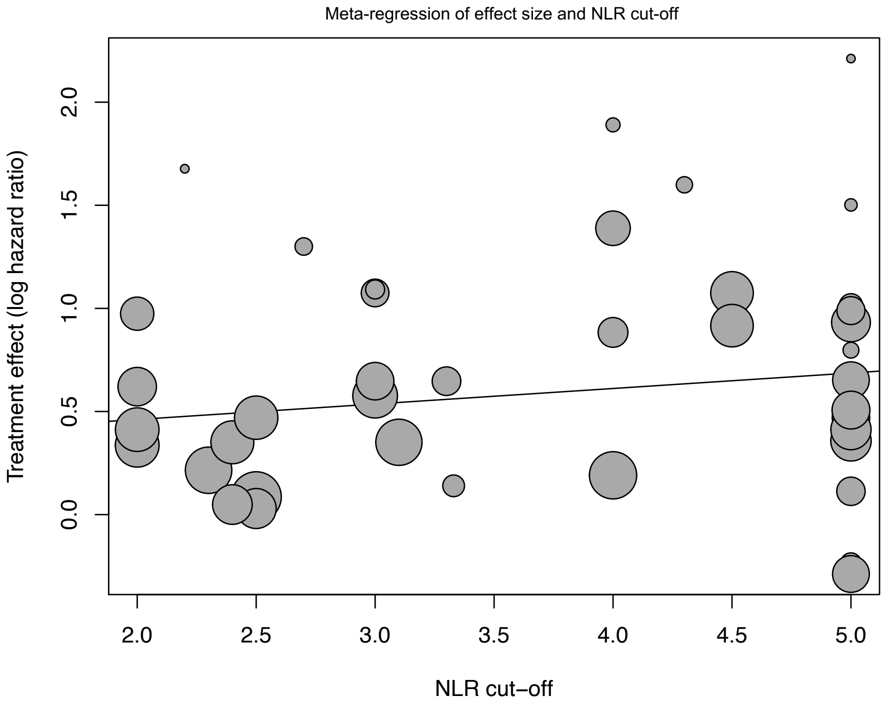

Supplementary *Figure 40* - NLR: OS in pancreatic cancer with resection

Study	TE	seTE	HR	95%–CI	Weight (fixed)	Weight (random)
**Adjustment = Multivariate**						
Asaoka 2016	1.30	0.4733	3.67	[1.45; 9.28]	2.7%	2.7%
Asari 2016	0.65	0.2042	1.91	[1.28; 2.85]	14.4%	14.4%
Ben 2015	0.41	0.1383	1.51	[1.15; 1.98]	31.4%	31.4%
Cheng 2016	0.62	0.1958	1.86	[1.27; 2.73]	15.6%	15.6%
Sierzega 2017	0.51	0.2007	1.66	[1.12; 2.46]	14.9%	14.9%
Takakura 2015	1.60	0.4996	4.95	[1.86; 13.18]	2.4%	2.4%
**Pooled HR by Fixed Effects**			**1.78**	**[1.50; 2.10]**	**81.4%**	**--**
**Pooled HR by Random Effects**			**1.79**	**[1.50; 2.13]**	**--**	**81.4%**
Heterogeneity: $I^2 = 39\%$ [0%; 76%], $\chi^2_5 = 8.23$ ($p = 0.14$)						
**Adjustment = Univariate**						
Bhatti 2010	0.58	0.2543	1.78	[1.08; 2.93]	9.3%	9.3%
Tao 2016	0.21	0.2531	1.23	[0.75; 2.02]	9.4%	9.4%
**Pooled HR by Fixed Effects**			**1.48**	**[1.04; 2.10]**	**18.6%**	**--**
**Pooled HR by Random Effects**			**1.48**	**[1.03; 2.12]**	**--**	**18.6%**
Heterogeneity: $I^2 = 6\%$, $\chi^2_1 = 1.06$ ($p = 0.30$)						
**Pooled HR by Fixed Effects**			**1.72**	**[1.48; 2.00]**	**100.0%**	**--**
**Pooled HR by Random Effects**			**1.72**	**[1.48; 2.00]**	**--**	**100.0%**
**Prediction interval**				**[1.42; 2.08]**		
Heterogeneity: $I^2 = 31\%$ [0%; 69%], $\chi^2_7 = 10.15$ ($p = 0.18$)						

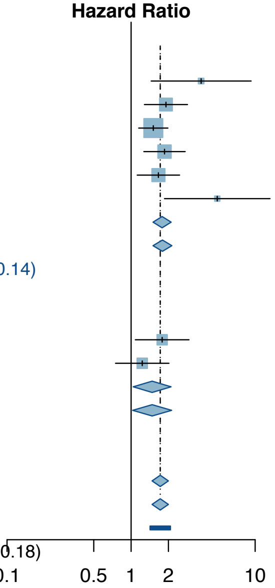

Tau²= 0.0012

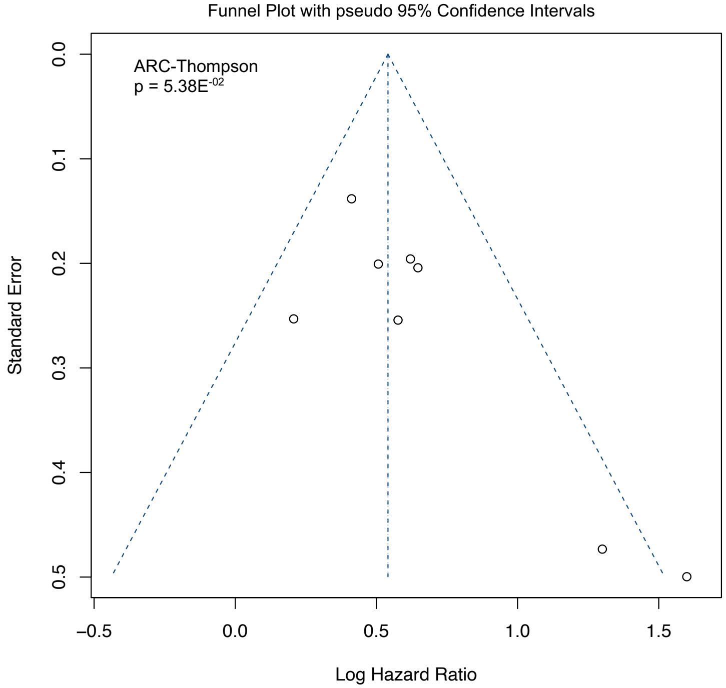

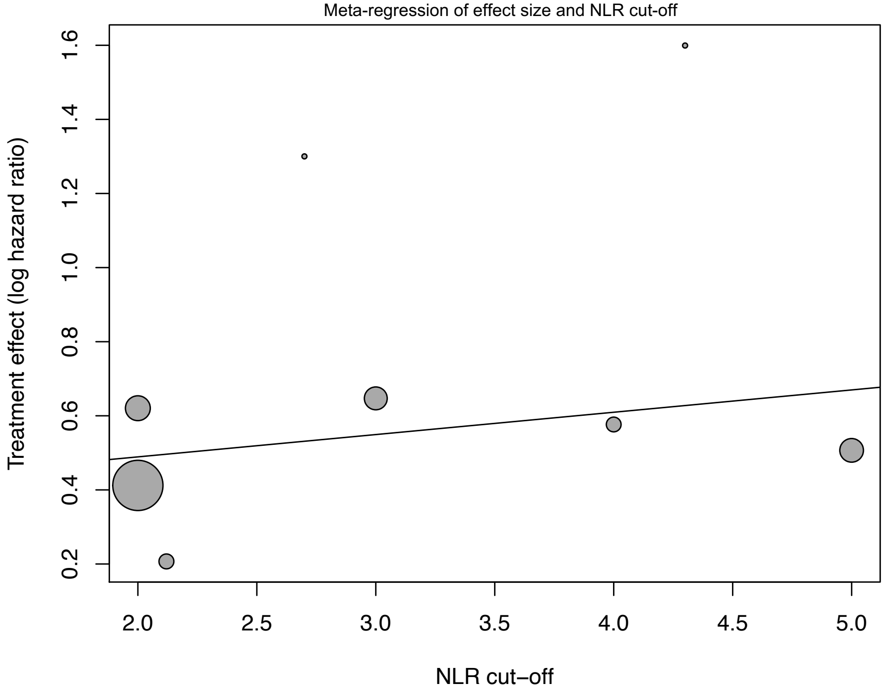

Supplementary *Figure 41* - NLR: DFS in pancreatic cancer

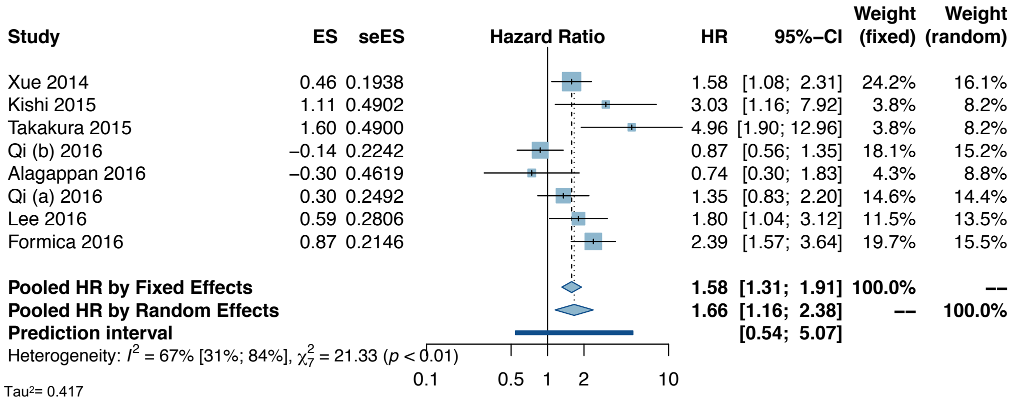

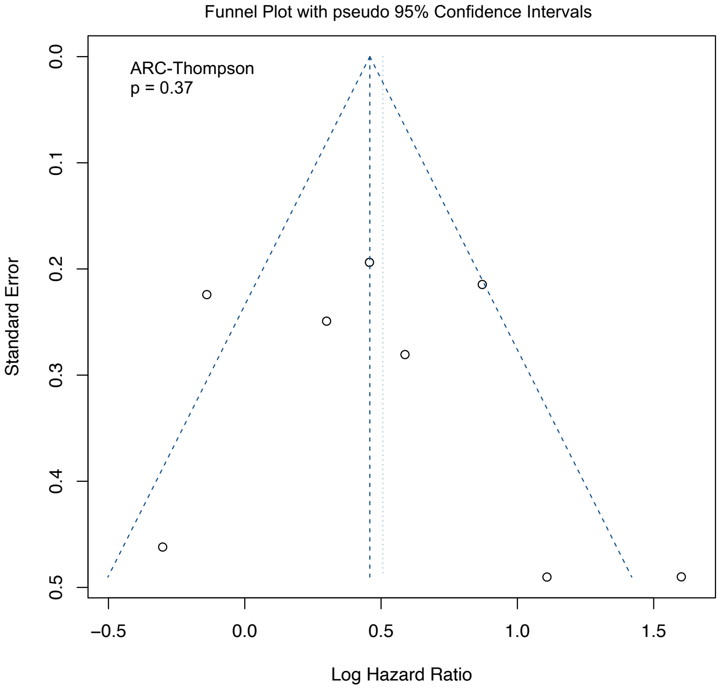

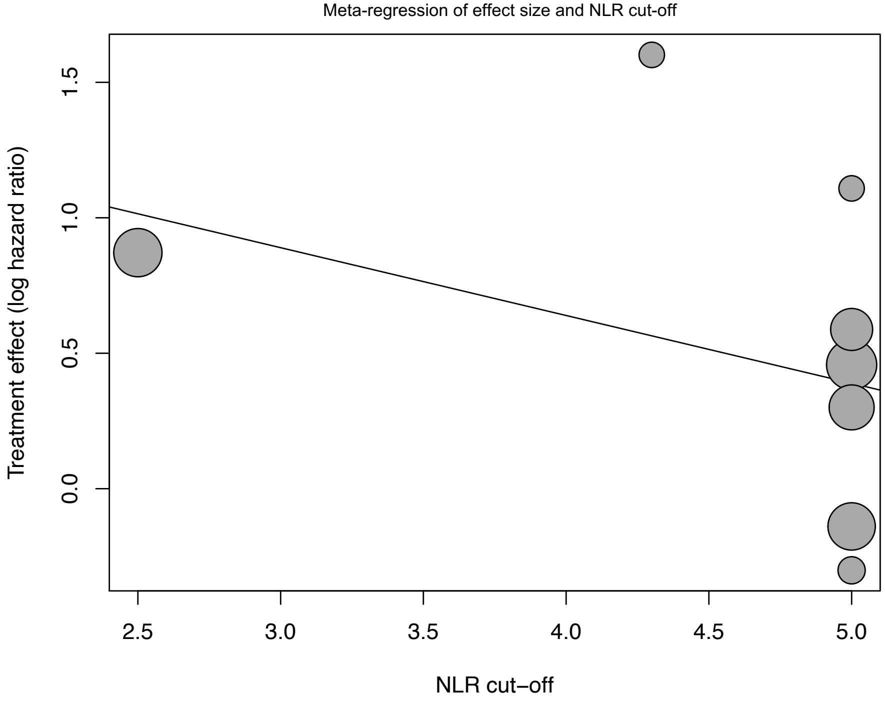

Supplementary *Figure 42* - NLR: CSS in pancreatic cancer

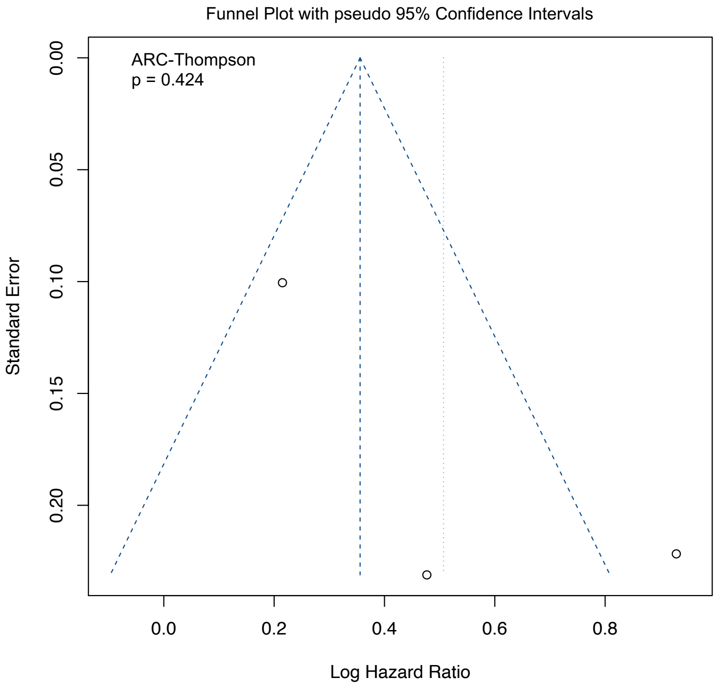

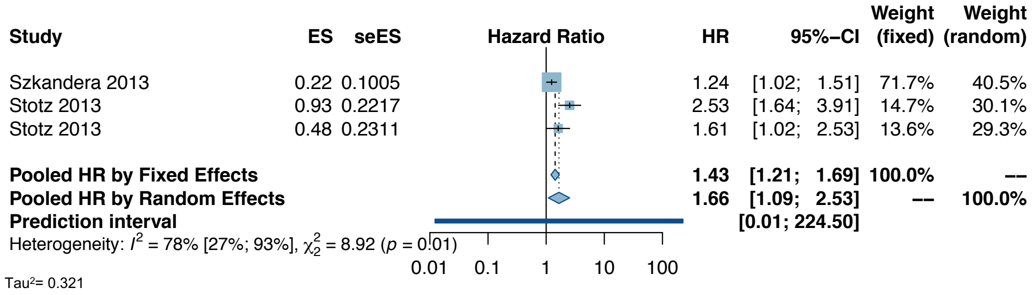

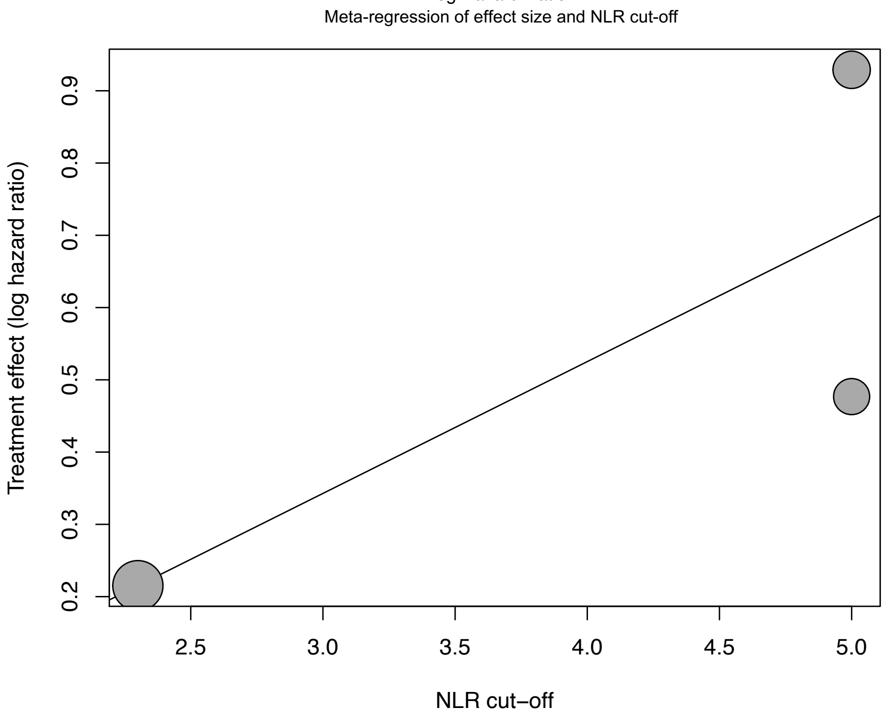

Supplementary *Figure 43* - NLR: OS in gynaecologic cancer

Study	ES	seES	HR	95%−CI	Weight (fixed)	Weight (random)
Asher 2011	−0.14	0.2571	0.87	[0.53; 1.44]	0.6%	4.3%
Badore−Rybicka 2016	0.09	0.0533	1.09	[0.98; 1.21]	14.8%	7.1%
Chen 2016	0.78	0.2968	2.18	[1.22; 3.90]	0.5%	3.8%
Cho 2009	2.13	1.0431	8.42	[1.09; 65.04]	0.0%	0.6%
Cho 2016	0.26	0.1123	1.30	[1.04; 1.62]	3.3%	6.4%
Cummings 2015	0.60	0.1839	1.82	[1.27; 2.61]	1.2%	5.4%
Eo 2016	0.70	0.2712	2.01	[1.18; 3.42]	0.6%	4.1%
Feng 2016	0.17	0.1181	1.19	[0.94; 1.50]	3.0%	6.4%
Gungorduk 2015	1.16	0.5449	3.20	[1.10; 9.31]	0.1%	1.8%
Haraga 2016(1)	0.43	0.3873	1.54	[0.72; 3.29]	0.3%	2.9%
Haraga 2016 (2)	0.52	0.8132	1.68	[0.34; 8.27]	0.1%	0.9%
Haruma 2015	1.20	0.5385	3.32	[1.16; 9.54]	0.1%	1.8%
Kim 2016	1.24	0.4348	3.45	[1.47; 8.09]	0.2%	2.5%
Lee 2012	0.17	0.0251	1.19	[1.13; 1.25]	66.7%	7.2%
Li 2015	0.83	0.6218	2.30	[0.68; 7.78]	0.1%	1.5%
Matuso 2015	0.78	0.3007	2.18	[1.21; 3.93]	0.5%	3.8%
Miao 2016	0.48	0.1766	1.62	[1.15; 2.29]	1.3%	5.5%
Mizunuma 2015	1.03	0.5253	2.80	[1.00; 7.84]	0.2%	1.9%
Nakamura 2015	0.94	0.5788	2.56	[0.82; 7.96]	0.1%	1.6%
Onal 2016	1.20	0.2838	3.32	[1.90; 5.79]	0.5%	4.0%
Takahashi 2015	0.90	0.2721	2.47	[1.45; 4.21]	0.6%	4.1%
Thavaramara 2011	−0.36	0.4218	0.70	[0.31; 1.60]	0.2%	2.6%
Wang 2016	1.21	0.4518	3.37	[1.39; 8.17]	0.2%	2.3%
Wang 2016	1.32	0.6319	3.73	[1.08; 12.87]	0.1%	1.4%
Williams 2014	0.31	0.1307	1.37	[1.06; 1.77]	2.5%	6.2%
Zhang 2015	0.77	0.1737	2.17	[1.54; 3.05]	1.4%	5.6%
Zhang 2014	0.49	0.2668	1.63	[0.97; 2.75]	0.6%	4.2%
**Pooled HR by Fixed Effects**			**1.24**	**[1.19; 1.29]**	**100.0%**	**--**
**Pooled HR by Random Effects**			**1.72**	**[1.46; 2.03]**	**--**	**100.0%**
**Prediction interval**				**[0.89; 3.35]**		

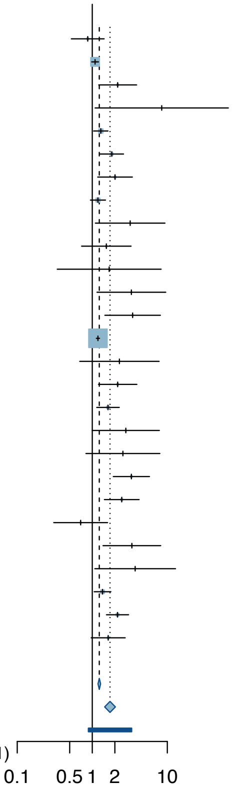

Heterogeneity: I^2 = 71% [57%; 80%], χ^2_{26} = 88.64 (p < 0.01)

Tau² = 0.312

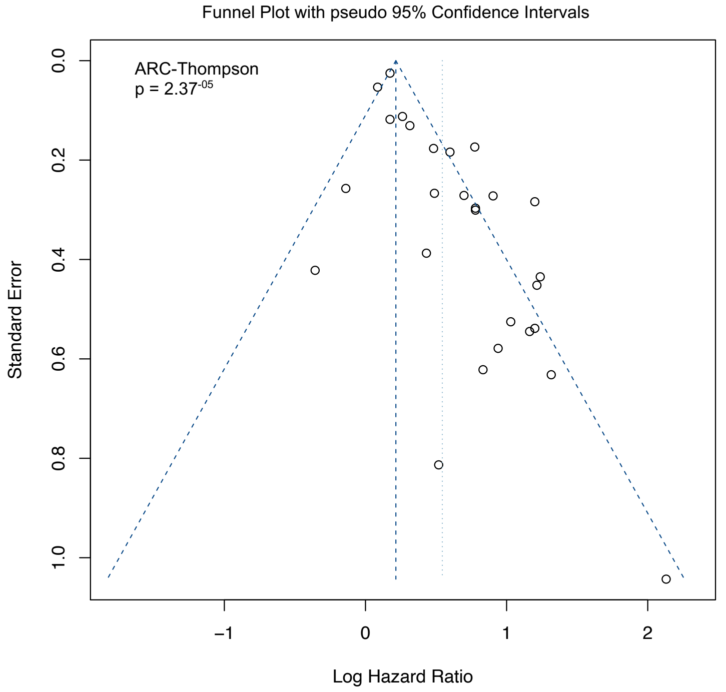

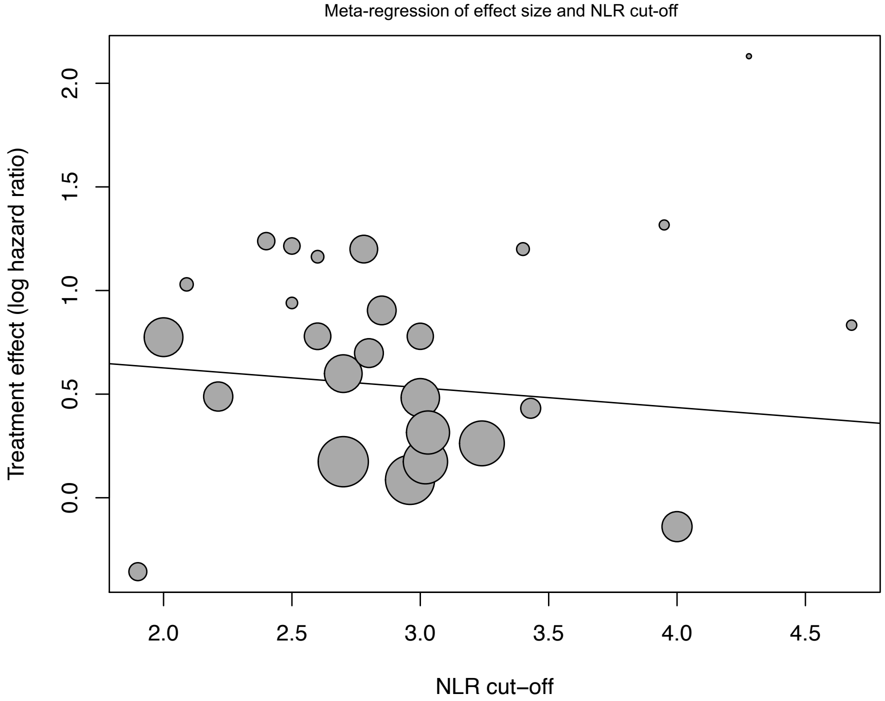

Supplementary *Figure 44* - NLR: OS in cervical cancer

Study	TE	seTE	HR	95%-CI	Weight (fixed)	Weight (random)
**Adjustment = Multivariate**						
Onal Chem 2016	1.20	0.2838	3.32	[1.90; 5.79]	0.6%	6.7%
Chen L 2016	0.78	0.2968	2.18	[1.22; 3.90]	0.5%	6.4%
Zhou WY 2016	−0.17	0.2606	0.84	[0.50; 1.40]	0.7%	7.4%
Mizunumq M 2015	1.03	0.6146	2.80	[0.84; 9.34]	0.1%	2.1%
Yang JW 2015	0.32	0.3113	1.38	[0.75; 2.54]	0.5%	6.0%
Li WT 2015	0.17	0.0494	1.18	[1.07; 1.30]	19.2%	15.8%
Zhang Y 2014	0.49	0.2668	1.63	[0.97; 2.75]	0.7%	7.2%
Lee YY 2012	0.17	0.0251	1.19	[1.13; 1.25]	74.3%	16.3%
**Pooled HR by Fixed Effects**			**1.20**	**[1.15; 1.25]**	**96.5%**	**--**
**Pooled HR by Random Effects**			**1.51**	**[1.11; 2.03]**	**--**	**67.9%**
Heterogeneity: $I^2 = 69\%$ [35%; 85%], $\chi^2_7 = 22.43$ ($p < 0.01$)						
**Adjustment = Univariate**						
Zheng RR 2016	0.39	0.2023	1.48	[1.00; 2.20]	1.1%	9.5%
Haraga J 2016	0.35	0.3428	1.42	[0.73; 2.78]	0.4%	5.3%
Wang YY 2016	0.60	0.2640	1.83	[1.09; 3.07]	0.7%	7.3%
Wang D 2013	0.28	0.1885	1.32	[0.91; 1.91]	1.3%	10.0%
**Pooled HR by Fixed Effects**			**1.47**	**[1.17; 1.84]**	**3.5%**	**--**
**Pooled HR by Random Effects**			**1.47**	**[1.17; 1.84]**	**--**	**32.1%**
Heterogeneity: $I^2 = 0\%$ [0%; 55%], $\chi^2_3 = 1.03$ ($p = 0.79$)						
**Pooled HR by Fixed Effects**			**1.21**	**[1.16; 1.26]**	**100.0%**	**--**
**Pooled HR by Random Effects**			**1.46**	**[1.21; 1.76]**	**--**	**100.0%**
**Prediction interval**				**[0.83; 2.57]**		
Heterogeneity: $I^2 = 58\%$ [21%; 78%], $\chi^2_{11} = 26.42$ ($p < 0.01$)						

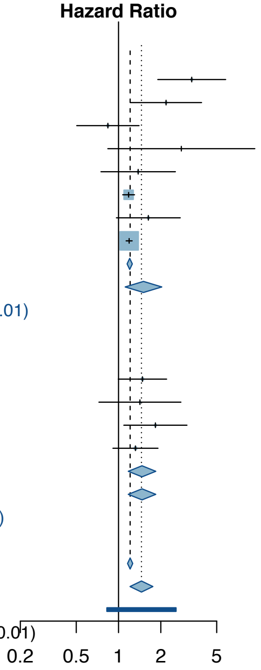

Tau²= 0.236

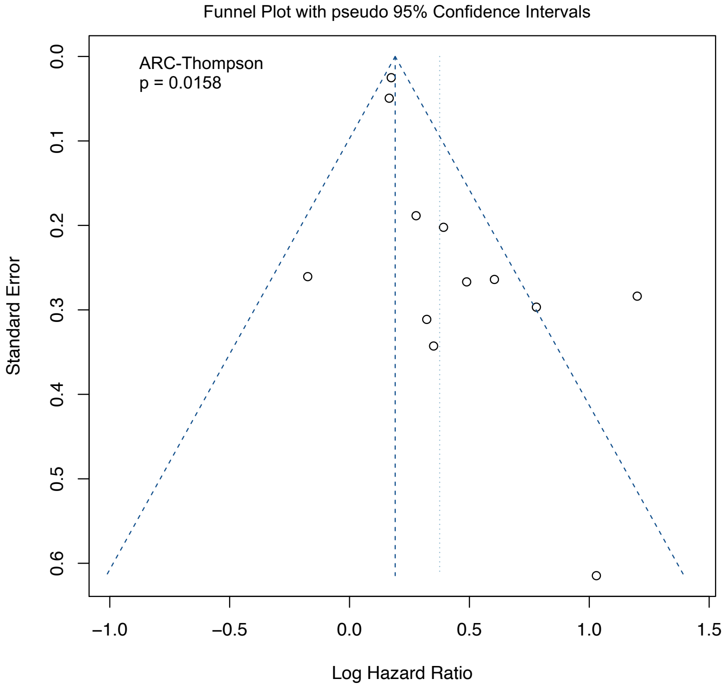

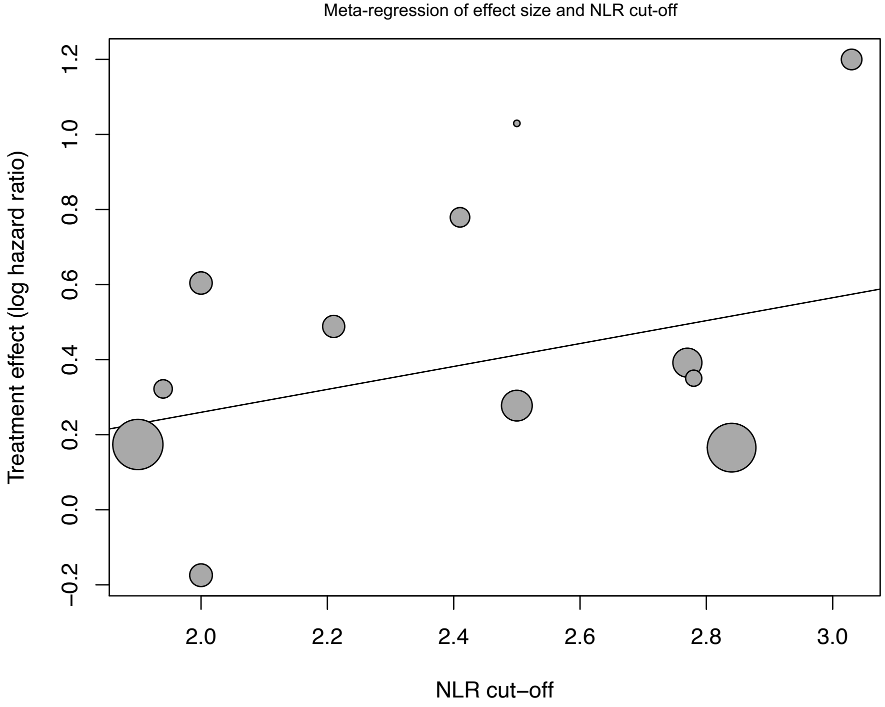

Supplementary *Figure 45* - NLR: PFS in cervical cancer

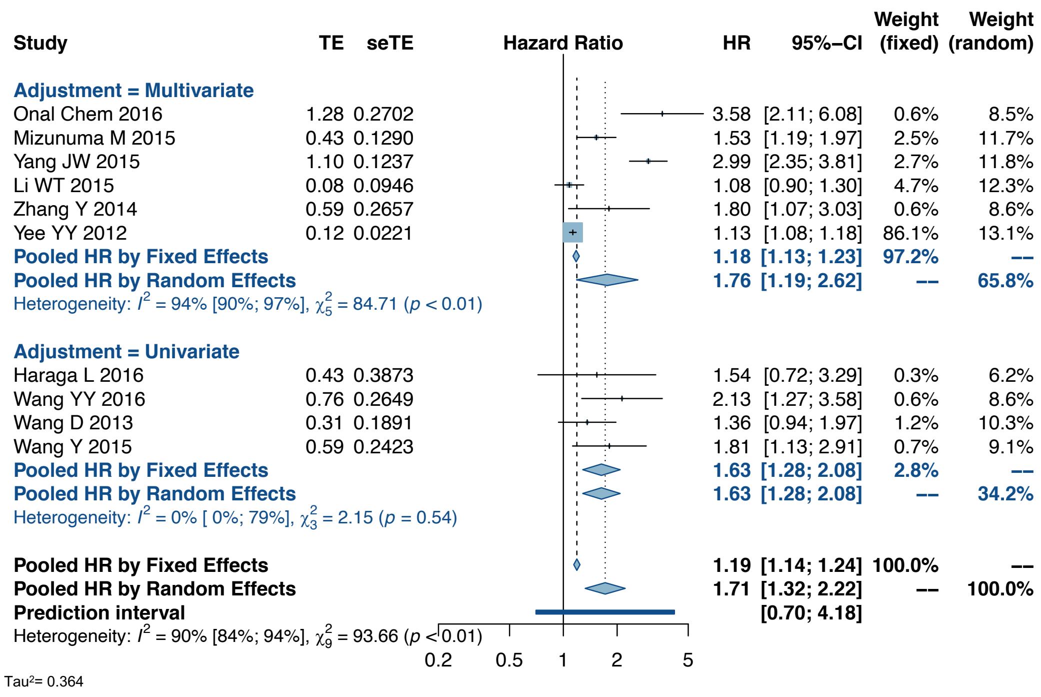

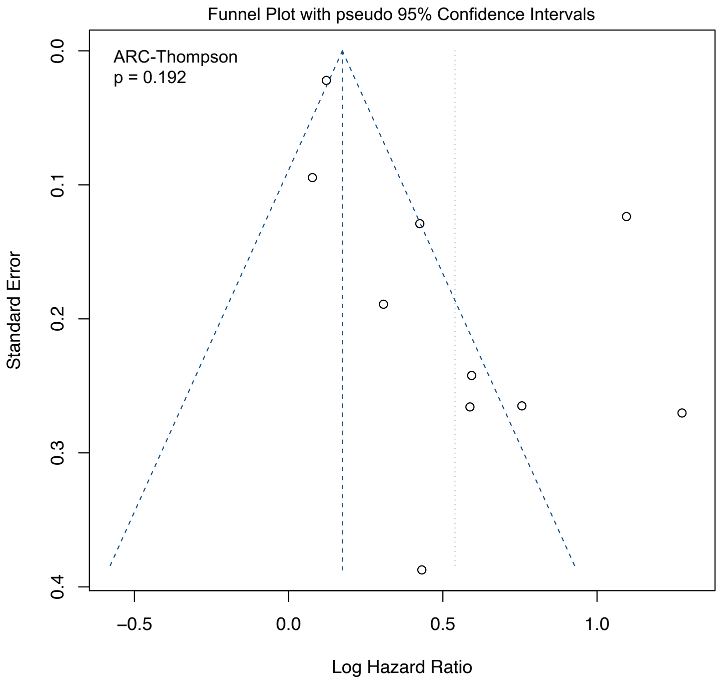

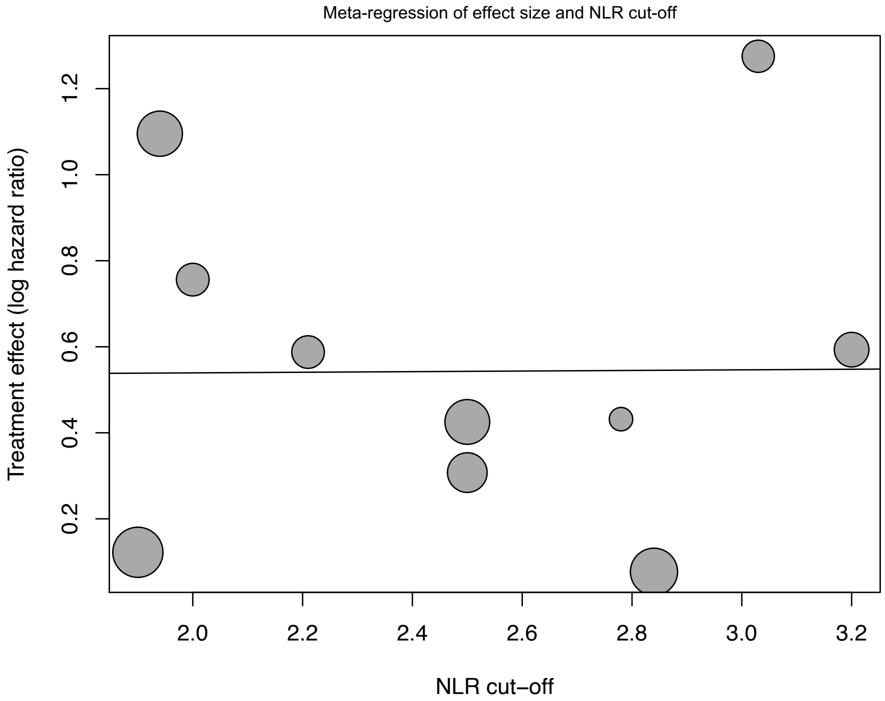

Supplementary *Figure 46* - NLR: PFS in ovarian cancer

Study	TE	seTE	HR	95%-CI	Weight (fixed)	Weight (random)
**Adjustment = Multivariate**						
Badora-Rybicka 2016	0.20	0.0666	1.22	[1.07; 1.39]	4.2%	11.8%
Feng 2016	0.22	0.0862	1.25	[1.06; 1.48]	2.5%	11.5%
Gungorduk 2015	0.74	0.6215	2.10	[0.62; 7.10]	0.0%	2.7%
Kim 2015	0.33	0.1057	1.39	[1.13; 1.71]	1.7%	11.1%
Li 2017	0.71	0.3435	2.04	[1.04; 4.00]	0.2%	5.9%
Miao 2016	0.55	0.1775	1.73	[1.22; 2.45]	0.6%	9.5%
Thavaramara 2011	−0.36	0.3536	0.70	[0.35; 1.40]	0.1%	5.7%
Wang 2015	1.93	0.4889	6.87	[2.64; 17.91]	0.1%	3.8%
Wang 2016	0.75	0.2523	2.11	[1.29; 3.46]	0.3%	7.7%
Zhang 2015	0.70	0.1581	2.01	[1.47; 2.74]	0.7%	10.0%
**Pooled HR by Fixed Effects**			**1.37**	**[1.26; 1.48]**	**10.3%**	**--**
**Pooled HR by Random Effects**			**1.60**	**[1.26; 2.02]**	**--**	**79.8%**
Heterogeneity: $I^2 = 71\%$ [45%; 85%], $\chi^2_9 = 31.01$ ($p < 0.01$)						
**Adjustment = Univariate**						
Eo 2015	0.85	0.2433	2.34	[1.45; 3.77]	0.3%	7.9%
Paik 2016	0.05	0.0144	1.05	[1.02; 1.08]	89.3%	12.3%
**Pooled HR by Fixed Effects**			**1.05**	**[1.02; 1.08]**	**89.7%**	**--**
**Pooled HR by Random Effects**			**1.51**	**[0.69; 3.30]**	**--**	**20.2%**
Heterogeneity: $I^2 = 91\%$, $\chi^2_1 = 10.81$ ($p < 0.01$)						
**Pooled HR by Fixed Effects**			**1.08**	**[1.05; 1.11]**	**100.0%**	**--**
**Pooled HR by Random Effects**			**1.58**	**[1.26; 1.98]**	**--**	**100.0%**
**Prediction interval**				**[0.72; 3.43]**		
Heterogeneity: $I^2 = 85\%$ [76%; 91%], $\chi^2_{11} = 75.78$ ($p < 0.01$)						

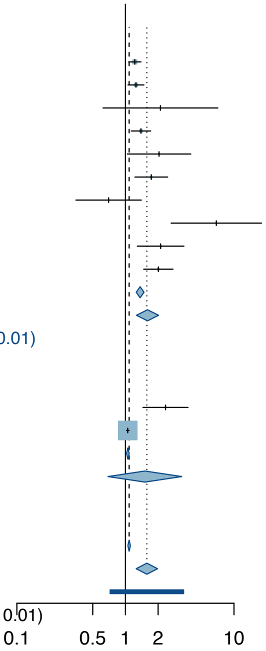

Tau²= 0.330

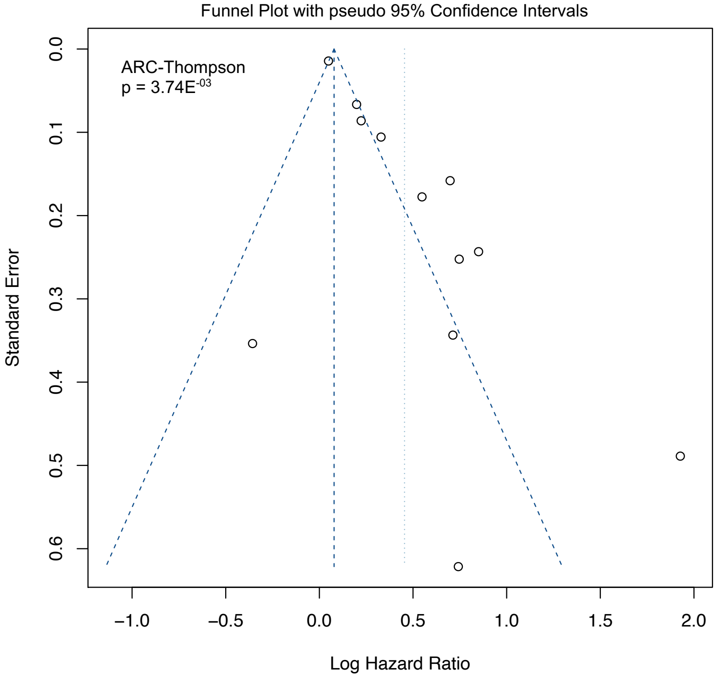

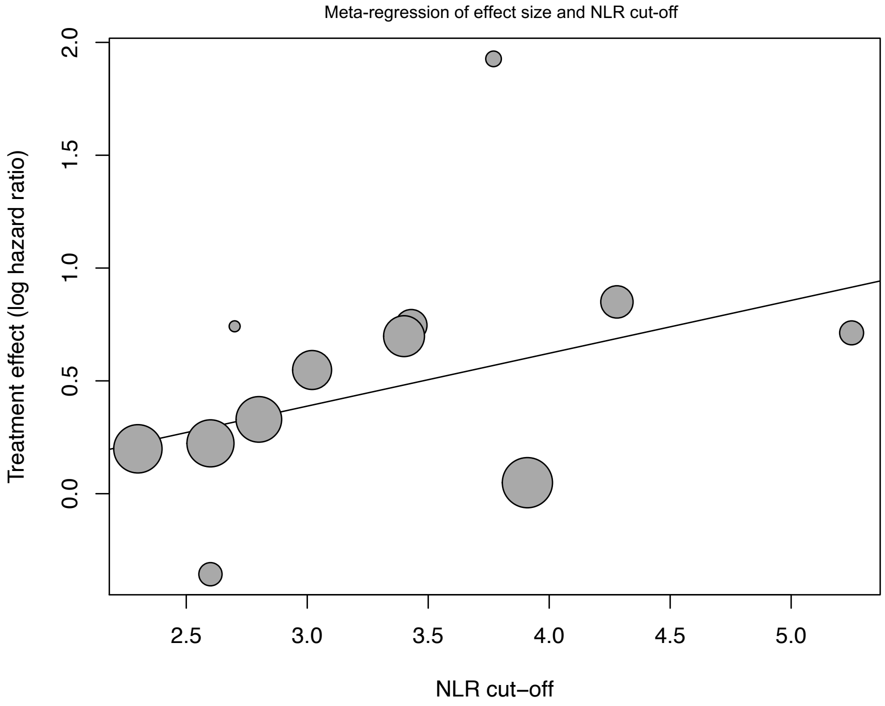

Supplementary *Figure 47* - NLR: OS in breast cancer

Study	ES	seES	HR	95%–CI	Weight (fixed)	Weight (random)
Azab 2012	1.41	0.4510	4.09	[1.69; 9.90]	3.1%	5.8%
Azab 2013	1.28	0.2674	3.60	[2.13; 6.08]	8.9%	10.4%
Bozkurt 2015	1.05	0.5158	2.86	[1.04; 7.86]	2.4%	4.8%
Dirican 2015	0.65	0.1915	1.91	[1.31; 2.78]	17.4%	13.0%
Forget 2014	0.85	0.4282	2.35	[1.02; 5.44]	3.5%	6.3%
Jia 2015	0.49	0.2141	1.63	[1.07; 2.48]	13.9%	12.2%
Koh 2014	3.21	1.0665	24.87	[3.08; 201.13]	0.6%	1.4%
Koh 2015	0.37	0.1512	1.45	[1.08; 1.95]	27.9%	14.5%
Nakano 2014	0.99	0.4954	2.70	[1.02; 7.13]	2.6%	5.1%
Noh 2013	1.41	0.4715	4.08	[1.62; 10.28]	2.9%	5.5%
Pistelli 2015	1.82	0.7073	6.16	[1.54; 24.64]	1.3%	3.0%
Rimando 2016	0.91	0.2310	2.48	[1.58; 3.90]	12.0%	11.6%
Yao 2014	1.29	0.4195	3.63	[1.60; 8.26]	3.6%	6.4%
**Pooled HR by Fixed Effects**			**2.14**	**[1.83; 2.50]**	**100.0%**	**--**
**Pooled HR by Random Effects**			**2.54**	**[1.96; 3.30]**	**--**	**100.0%**
**Prediction interval**				**[1.20; 5.41]**		

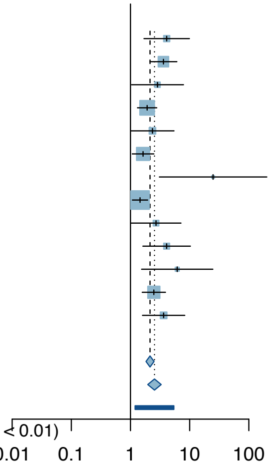

Heterogeneity: $I^2 = 55\%$ [15%; 76%], $\chi^2_{12} = 26.42$ ($p < 0.01$)

Tau²= 0.316

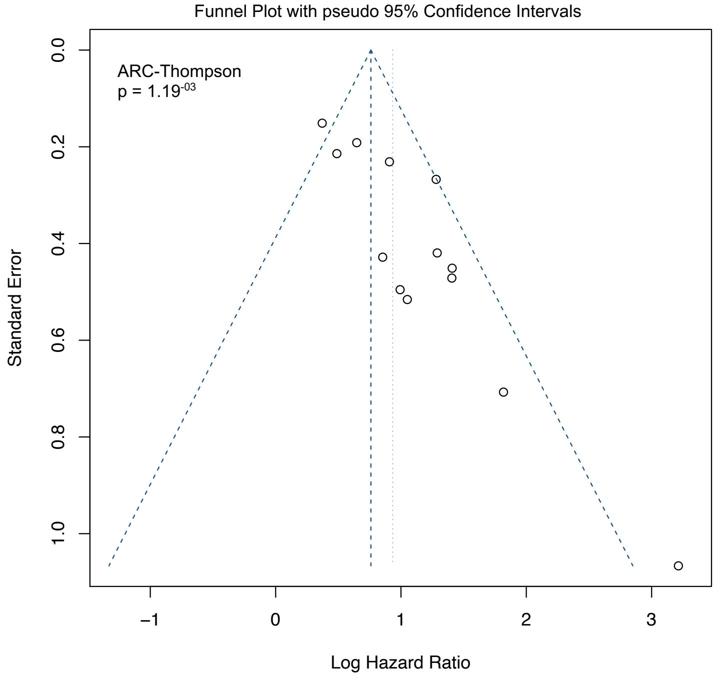

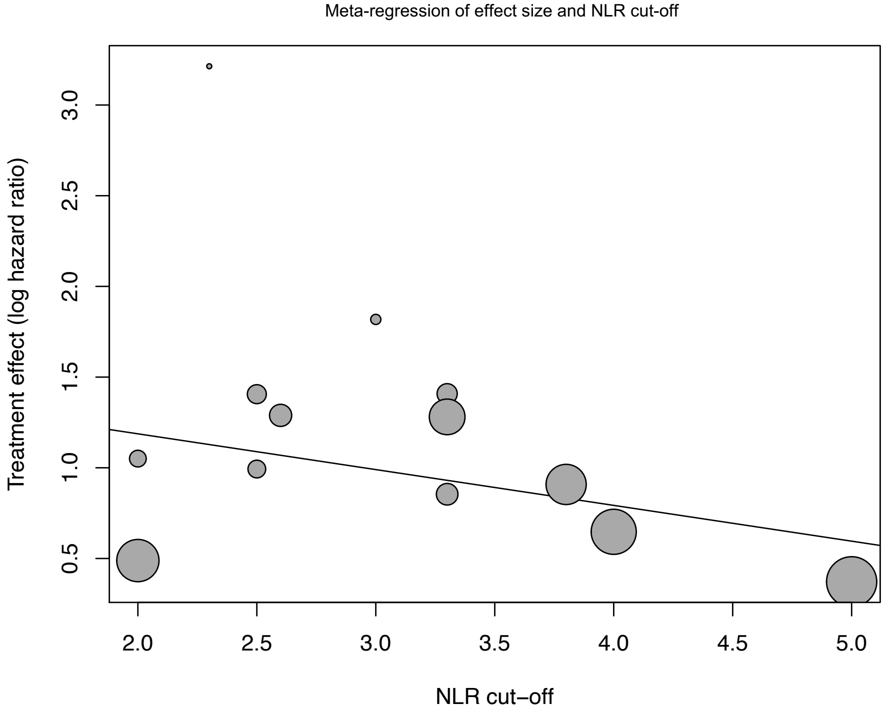

Supplementary *Figure 48* - NLR: OS in breast cancer with metastasis

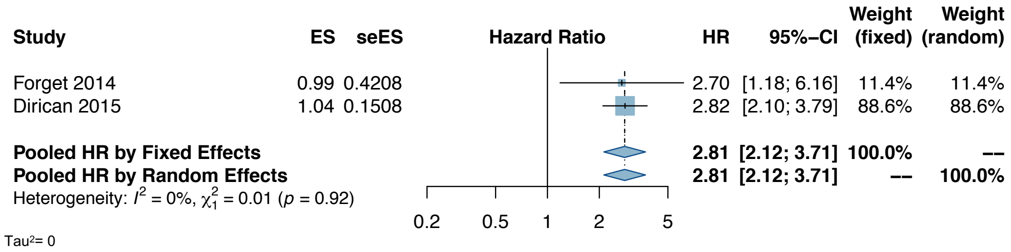

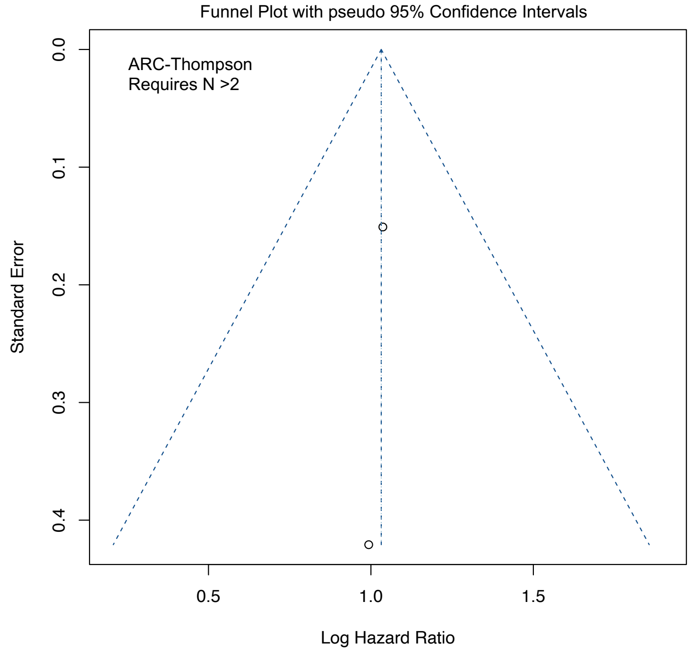

Supplementary *Figure 49* - NLR: OS in breast cancer without metastasis

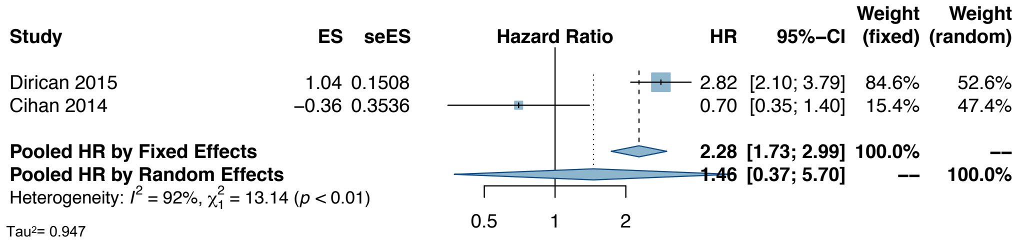

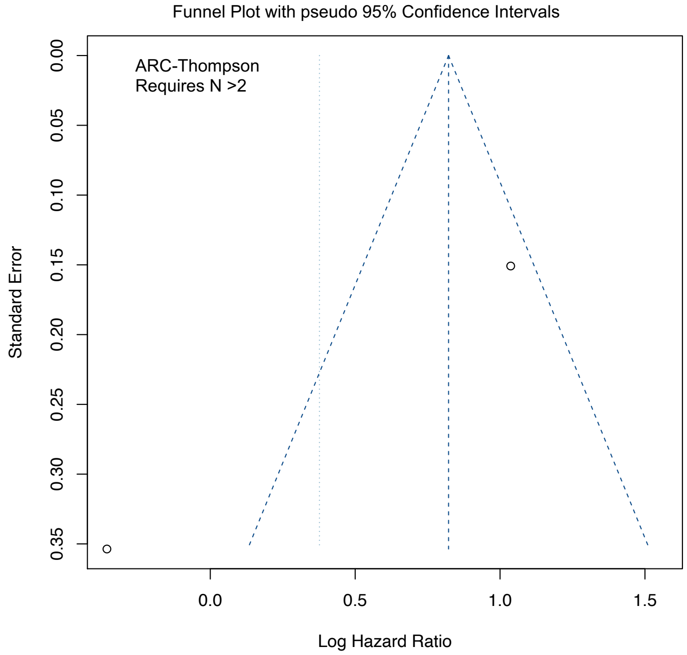

Supplementary *Figure 50* - NLR: OS in triple-negative breast cancer

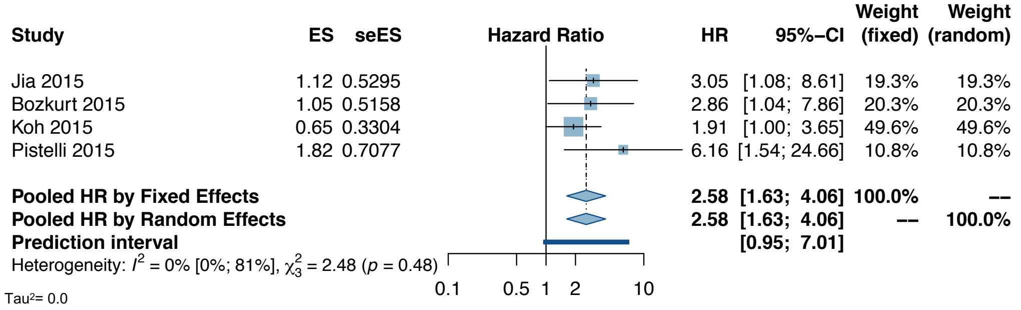

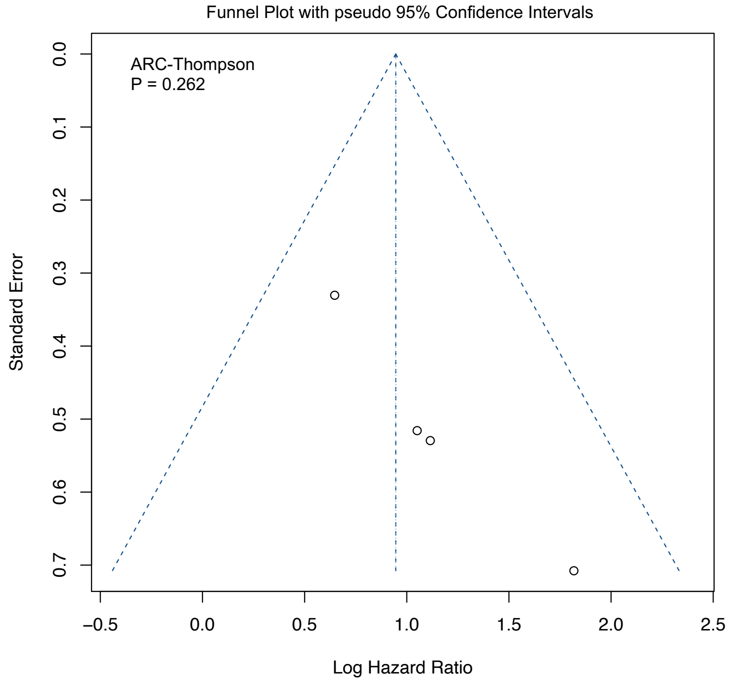

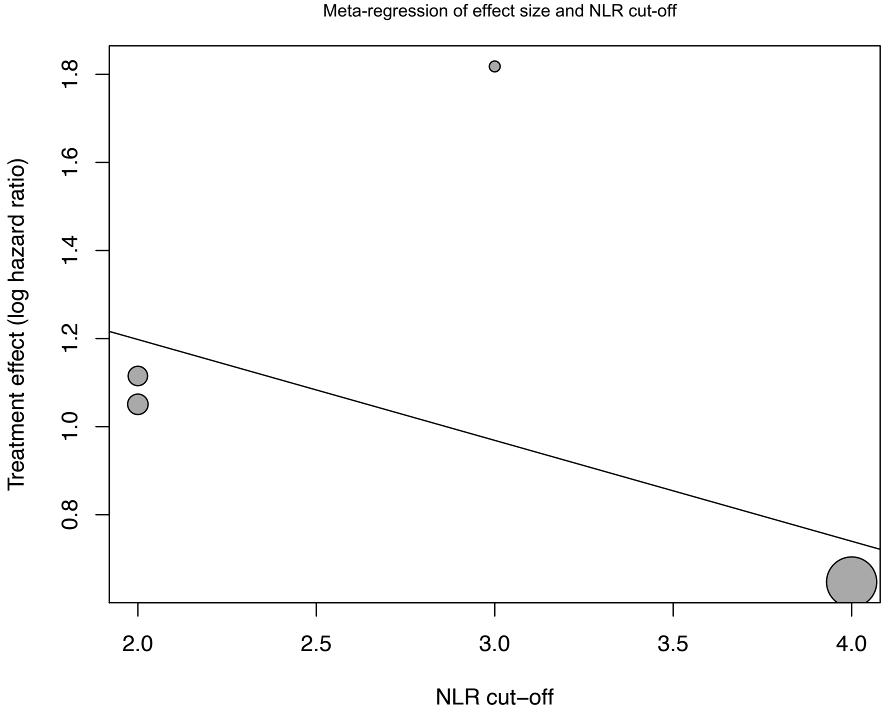

Supplementary Figure 51 - NLR: DFS in breast cancer

Study	TE	seTE	HR	95%–CI	Weight (fixed)	Weight (random)
**Adjustment = Multivariate**						
Kim 2016	1.37	0.5742	3.93	[1.28; 12.11]	0.2%	3.7%
Jia 2015	0.41	0.1391	1.50	[1.14; 1.97]	2.9%	15.2%
Bozkurt 2015	1.70	0.6226	5.46	[1.61; 18.50]	0.1%	3.2%
Suppan 2015	0.01	0.0247	1.01	[0.96; 1.06]	91.7%	18.8%
Forget 2014	0.69	0.2748	1.99	[1.16; 3.41]	0.7%	9.7%
Nakano 2014	0.69	0.3662	2.00	[0.98; 4.10]	0.4%	7.0%
Dirican 2015	0.38	0.1707	1.46	[1.04; 2.04]	1.9%	13.8%
Pistelli 2015	1.64	0.7827	5.15	[1.11; 23.88]	0.1%	2.2%
Asano 2016	2.41	1.7670	11.11	[0.35; 354.67]	0.0%	0.5%
Hong 2016	0.63	0.2446	1.87	[1.16; 3.02]	0.9%	10.8%
**Pooled HR by Fixed Effects**			**1.05**	**[1.00; 1.10]**	**99.0%**	**--**
**Pooled HR by Random Effects**			**1.78**	**[1.32; 2.39]**	**--**	**84.9%**
Heterogeneity: $I^2 = 80\%$ [64%; 89%], $\chi^2_9 = 45.08$ ($p < 0.01$)						
**Adjustment = Univariate**						
Zhang 2016	0.36	0.2971	1.43	[0.80; 2.56]	0.6%	9.0%
Ulas 2015	0.30	0.4057	1.35	[0.61; 2.99]	0.3%	6.1%
**Pooled HR by Fixed Effects**			**1.40**	**[0.88; 2.24]**	**1.0%**	**--**
**Pooled HR by Random Effects**			**1.40**	**[0.88; 2.24]**	**--**	**15.1%**
Heterogeneity: $I^2 = 0\%$, $\chi^2_1 = 0.01$ ($p = 0.91$)						
**Pooled HR by Fixed Effects**			**1.05**	**[1.01; 1.10]**	**100.0%**	**--**
**Pooled HR by Random Effects**			**1.65**	**[1.30; 2.10]**	**--**	**100.0%**
**Prediction interval**				**[0.83; 3.27]**		
Heterogeneity: $I^2 = 76\%$ [59%; 86%], $\chi^2_{11} = 46.52$ ($p < 0.01$)						

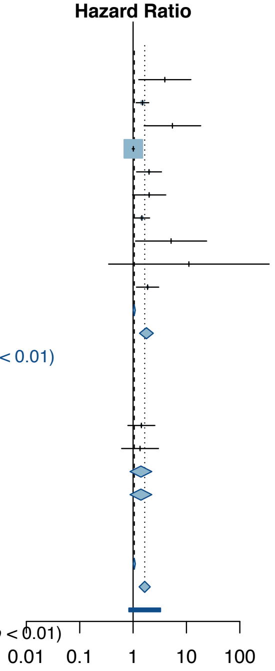

Tau²= 0.281

Funnel Plot with pseudo 95% Confidence Intervals

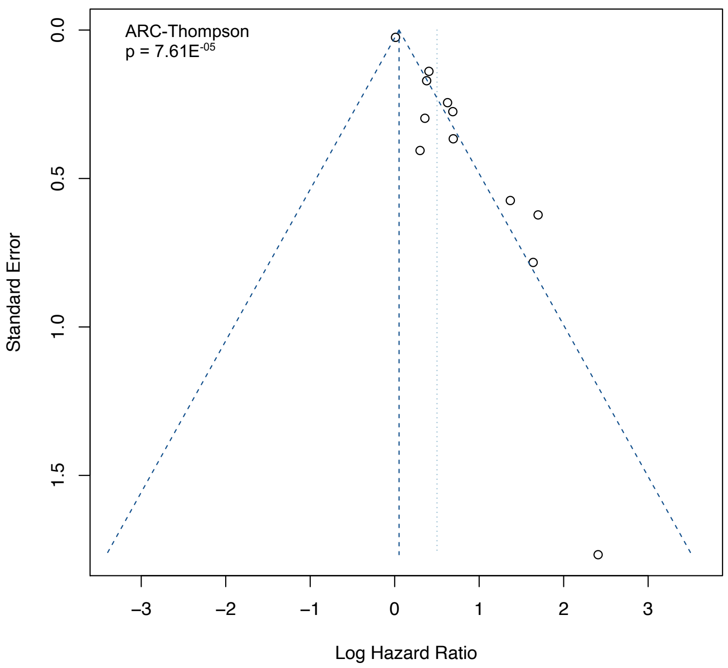

Meta-regression of effect size and NLR cut-off

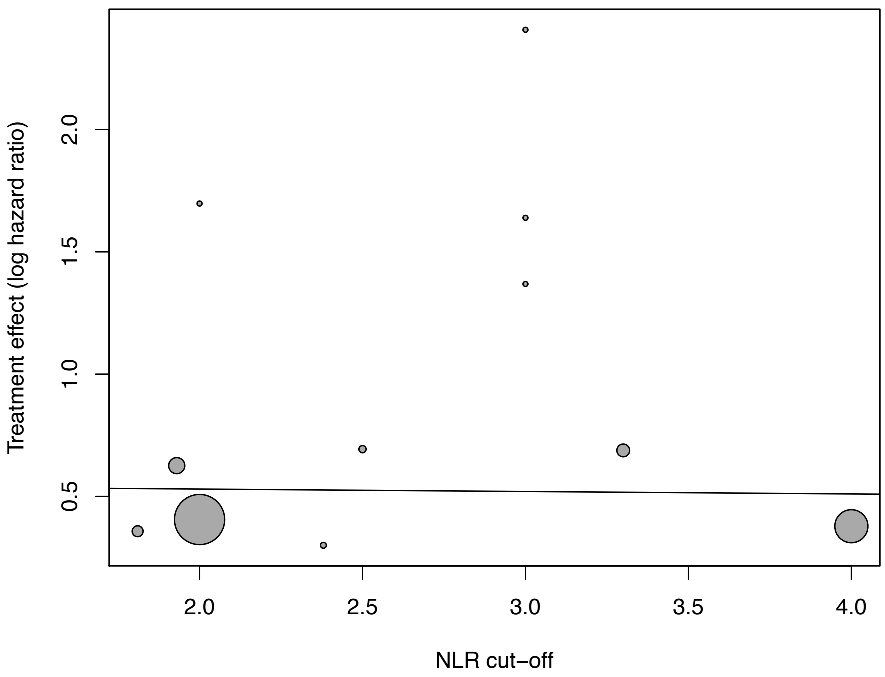

Supplementary Figure 52 - NLR: DFS in breast cancer with metastasis

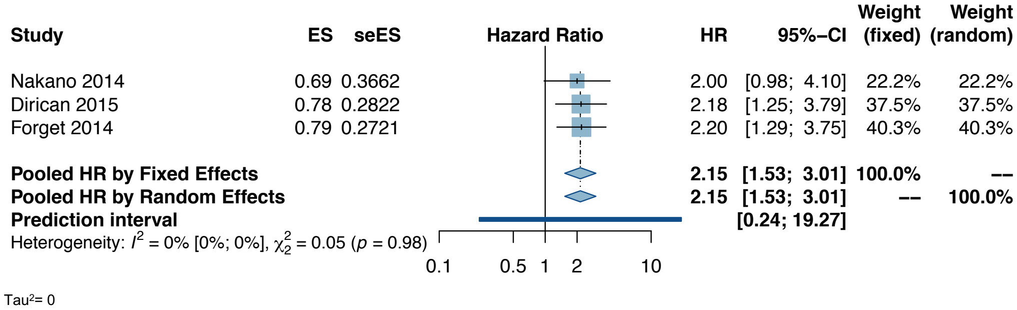

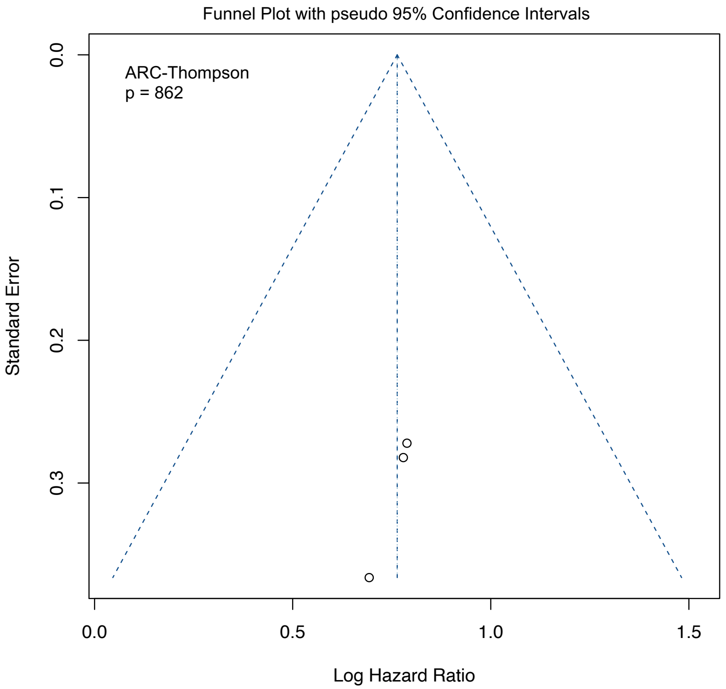

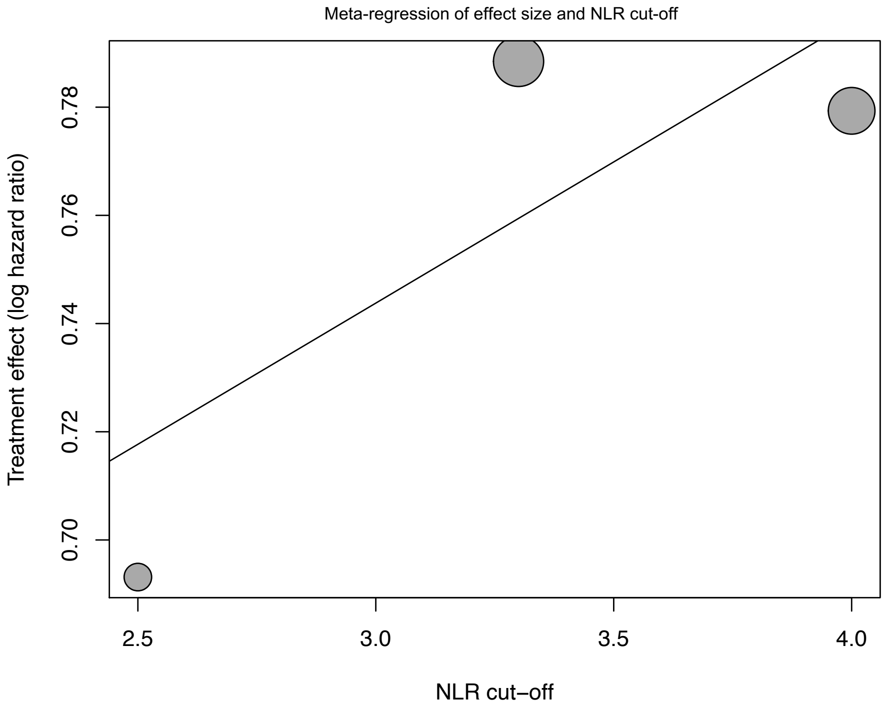

Supplementary Figure 53 - NLR: DFS in breast cancer without metastasis

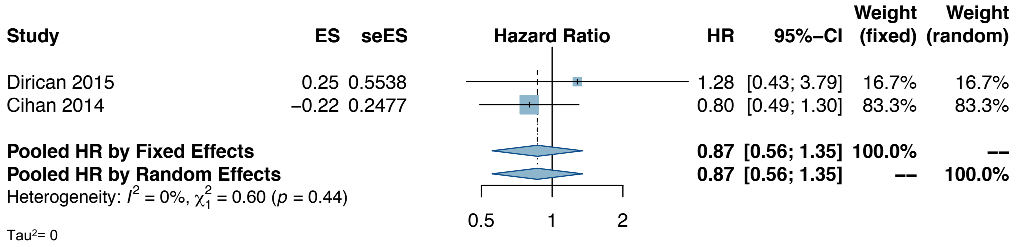

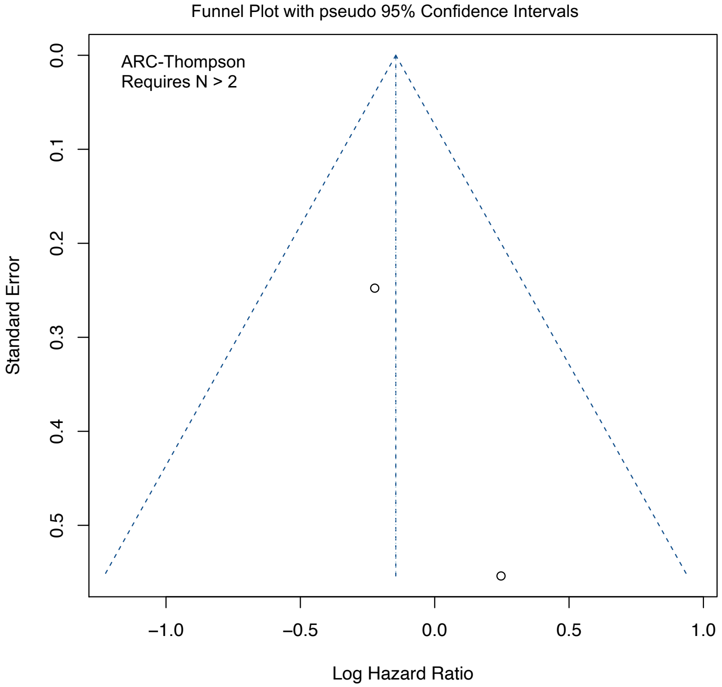

Supplementary Figure 54 - NLR: DFS in triple-negative breast cancer

Study	ES	seES	HR	95%−CI	Weight (fixed)	Weight (random)
Jia 2015	0.95	0.3787	2.58	[1.23; 5.42]	60.6%	60.6%
Bozkurt 2015	1.70	0.6226	5.46	[1.61; 18.50]	22.4%	22.4%
Pistelli 2015	1.64	0.7827	5.15	[1.11; 23.88]	14.2%	14.2%
Asano 2016	2.41	1.7670	11.11	[0.35; 354.67]	2.8%	2.8%
**Pooled HR by Fixed Effects**			**3.51**	**[1.97; 6.25]**	**100.0%**	**--**
**Pooled HR by Random Effects**			**3.51**	**[1.97; 6.25]**	**--**	**100.0%**
**Prediction interval**				**[0.99; 12.47]**		

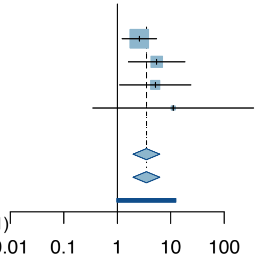

Heterogeneity: $I^2 = 0\%$ [0%; 75%], $\chi^2_3 = 1.83$ ($p = 0.61$)

Tau²= 0

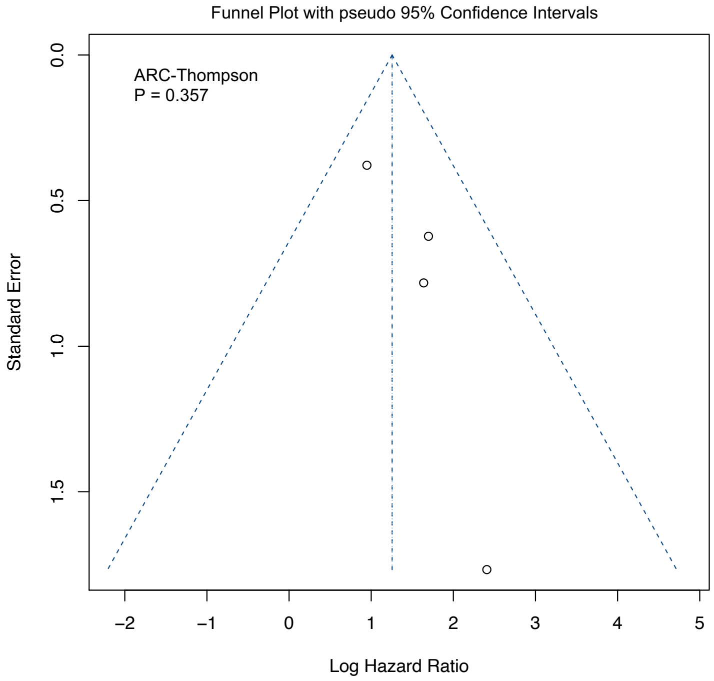

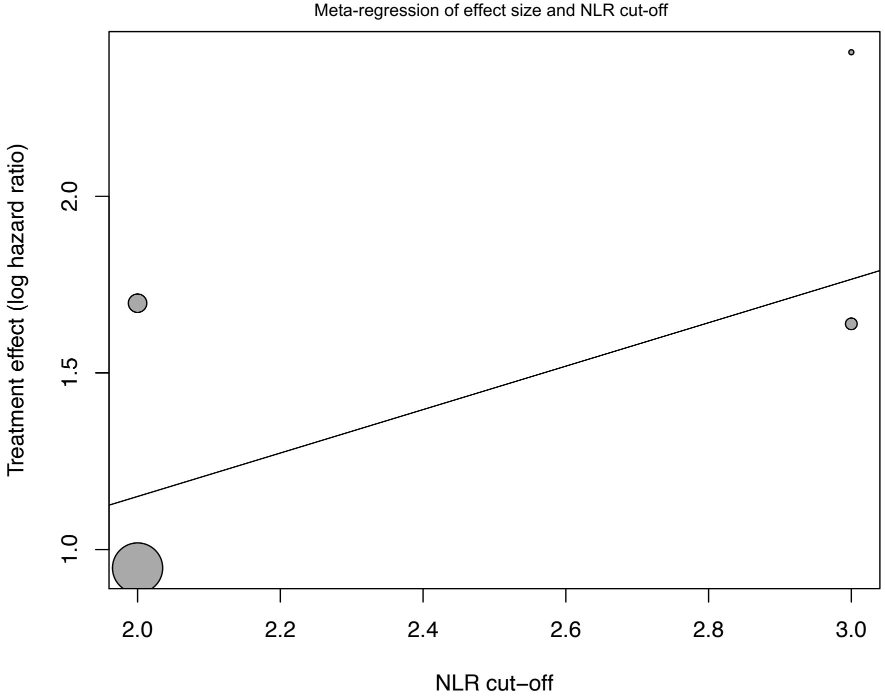

Supplementary Figure 55 - NLR: CSS in breast cancer

Study	ES	seES	HR	95%–CI	Weight (fixed)	Weight (random)
Lee 2015	0.09	0.0739	1.09	[0.94; 1.26]	92.7%	36.6%
Nakano 2014	0.99	0.5075	2.70	[1.00; 7.30]	2.0%	19.4%
Noh 2013	1.41	0.4715	4.08	[1.62; 10.28]	2.3%	20.8%
Rimando 2016	0.82	0.4110	2.27	[1.01; 5.08]	3.0%	23.3%
**Pooled HR by Fixed Effects**			**1.17**	**[1.02; 1.34]**	**100.0%**	**--**
**Pooled HR by Random Effects**			**2.03**	**[1.08; 3.82]**	**--**	**100.0%**
**Prediction interval**				**[0.14; 29.25]**		

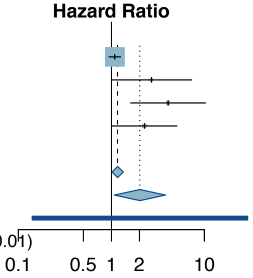

Heterogeneity: $I^2 = 77\%$ [38%; 92%], $\chi^2_3 = 13.25$ ($p < 0.01$)

Tau²= 0.529

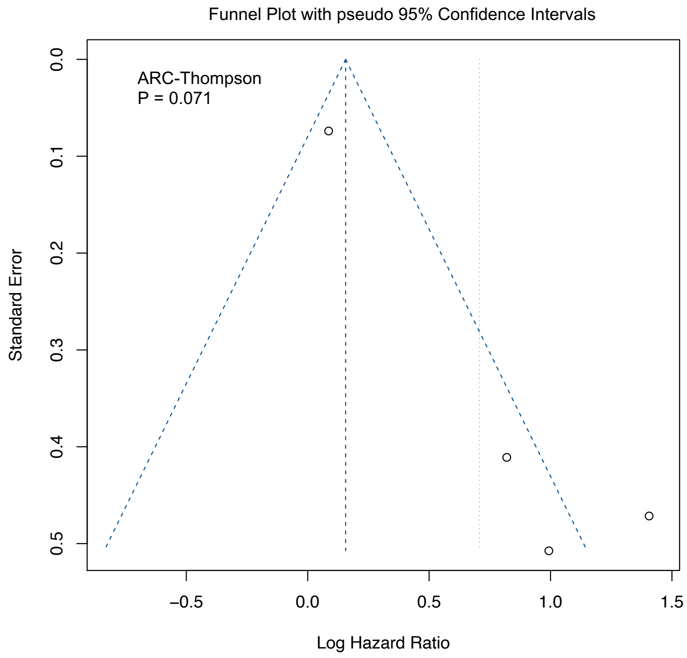

Supplementary Figure 56 - IN: OS in hepatocellular carcinoma and cholangiocarcinoma

Study	ES	seES	HR	95%–CI	Weight (fixed)	Weight (random)
Zhou 2012	0.69	0.1625	2.00	[1.45; 2.75]	36.6%	29.3%
Gu 2012	0.57	0.2088	1.76	[1.17; 2.65]	22.1%	24.2%
Li 2011	0.95	0.2376	2.58	[1.62; 4.11]	17.1%	21.4%
Kuang 2010	0.17	0.1997	1.19	[0.80; 1.76]	24.2%	25.1%
**Pooled HR by Fixed Effects**			**1.79**	**[1.48; 2.17]**	**100.0%**	**--**
**Pooled HR by Random Effects**			**1.80**	**[1.33; 2.43]**	**--**	**100.0%**
**Prediction interval**				**[0.54; 5.99]**		

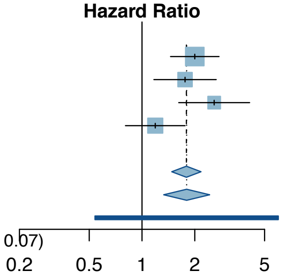

Heterogeneity: $I^2 = 57\%$ [0%; 86%], $\chi^2_3 = 7.02$ ($p = 0.07$)

Tau²= 0.234

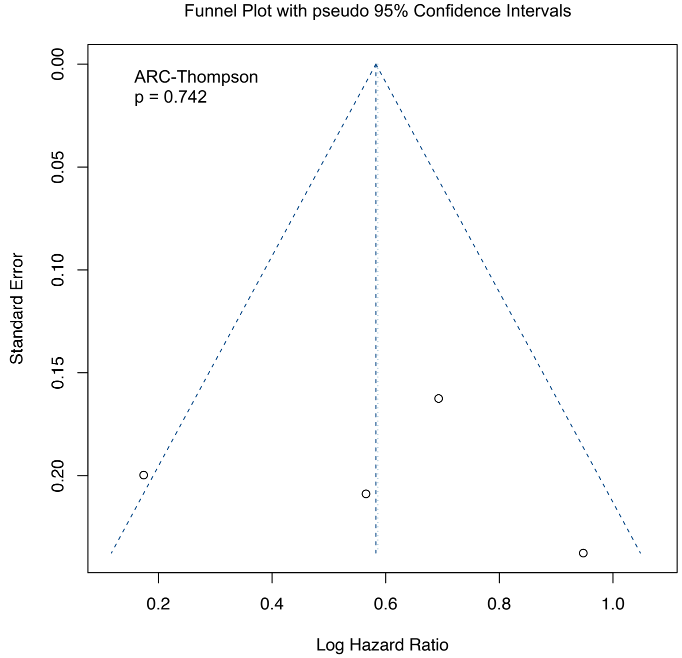

Supplementary Figure 57 - NLR: OS in hepatocellular carcinoma

Study	ES	seES	HR	95%–CI	Weight (fixed)	Weight (random)
Aino 2014	0.64	0.1240	1.89	[1.48; 2.41]	1.3%	2.9%
Arai 2017	0.85	0.4027	2.33	[1.06; 5.13]	0.1%	1.7%
Chen 2012	0.24	0.0711	1.27	[1.10; 1.46]	4.1%	3.0%
da Fonsecaet 204	−0.69	0.2269	0.50	[0.32; 0.78]	0.4%	2.4%
Dan 2013	0.46	0.2164	1.59	[1.04; 2.43]	0.4%	2.5%
Dumitrascu 2013	−0.27	0.1451	0.76	[0.57; 1.01]	1.0%	2.8%
Fan 2015	−0.82	0.2441	0.44	[0.27; 0.71]	0.3%	2.3%
Fu 2013	0.36	0.1634	1.43	[1.04; 1.97]	0.8%	2.7%
Gao 2015	0.16	0.0214	1.17	[1.12; 1.22]	45.3%	3.1%
Goh 2016	0.40	0.2661	1.49	[0.88; 2.51]	0.3%	2.2%
Gomez (ICC) 2008	0.58	0.5902	1.78	[0.56; 5.66]	0.1%	1.1%
Guo 2009	0.27	0.4652	1.31	[0.53; 3.26]	0.1%	1.4%
Halazun 2009	1.81	0.5012	6.10	[2.28; 16.29]	0.1%	1.3%
He 2017	0.97	0.3020	2.65	[1.47; 4.79]	0.2%	2.1%
Howell 2017	0.22	0.0505	1.25	[1.13; 1.38]	8.1%	3.1%
Hu 2016	0.52	0.3319	1.68	[0.88; 3.22]	0.2%	1.9%
Huang 2011	−0.39	0.1865	0.68	[0.47; 0.98]	0.6%	2.6%
Ji 2016	0.39	0.1571	1.47	[1.08; 2.00]	0.8%	2.7%
Kinoshita 2012	1.43	0.3201	4.17	[2.23; 7.81]	0.2%	2.0%
Li 2014	0.10	0.0315	1.10	[1.03; 1.17]	20.8%	3.1%
Liao 2014	0.49	0.1545	1.64	[1.21; 2.22]	0.9%	2.7%
Limaye 2013	0.75	0.2085	2.12	[1.41; 3.19]	0.5%	2.5%
Lu 2016	0.26	0.0930	1.30	[1.08; 1.56]	2.4%	3.0%
Mano 2013	1.32	0.6609	3.74	[1.02; 13.66]	0.0%	0.9%
McNally 2013	0.94	0.2602	2.57	[1.54; 4.28]	0.3%	2.3%
Motomura 2013	1.35	0.3266	3.87	[2.04; 7.34]	0.2%	2.0%
Na 2014	1.06	0.3701	2.90	[1.40; 5.99]	0.2%	1.8%
Oh 2013	0.47	0.1807	1.60	[1.12; 2.28]	0.6%	2.6%
Okamura 2015	0.88	0.2623	2.41	[1.44; 4.03]	0.3%	2.3%
Okamura 2016	0.99	0.2748	2.69	[1.57; 4.61]	0.3%	2.2%
Peng 2014	0.97	0.3389	2.64	[1.36; 5.13]	0.2%	1.9%
Pinato (M) 2012	0.72	0.2932	2.06	[1.16; 3.66]	0.2%	2.1%
Pinato (T) 2012	1.34	0.6318	3.80	[1.10; 13.11]	0.1%	1.0%
Sukato (a) 2015	0.37	0.1986	1.45	[0.98; 2.14]	0.5%	2.6%
Sukato (b) 2015	0.57	0.2709	1.77	[1.04; 3.01]	0.3%	2.2%
Sullivan 2014	0.09	0.0699	1.09	[0.95; 1.25]	4.2%	3.0%
Tajiri 2015	1.18	0.5286	3.24	[1.15; 9.13]	0.1%	1.2%
Terashima 2014	0.40	0.1527	1.49	[1.10; 2.01]	0.9%	2.8%
Wang 2011	0.97	0.3198	2.65	[1.42; 4.96]	0.2%	2.0%
Wei 2014	1.12	0.3139	3.07	[1.66; 5.68]	0.2%	2.0%
Xiao 2015	0.35	0.2460	1.42	[0.88; 2.30]	0.3%	2.3%
Xu 2014	0.52	0.2576	1.69	[1.02; 2.80]	0.3%	2.3%
Yang 2016	0.29	0.1429	1.33	[1.01; 1.76]	1.0%	2.8%
Zhang 2014	0.76	0.2013	2.13	[1.44; 3.16]	0.5%	2.5%
**Pooled HR by Fixed Effects**			**1.22**	**[1.18; 1.25]**	**100.0%**	**--**
**Pooled HR by Random Effects**			**1.64**	**[1.41; 1.90]**	**--**	**100.0%**
**Prediction interval**				**[0.68; 3.95]**		

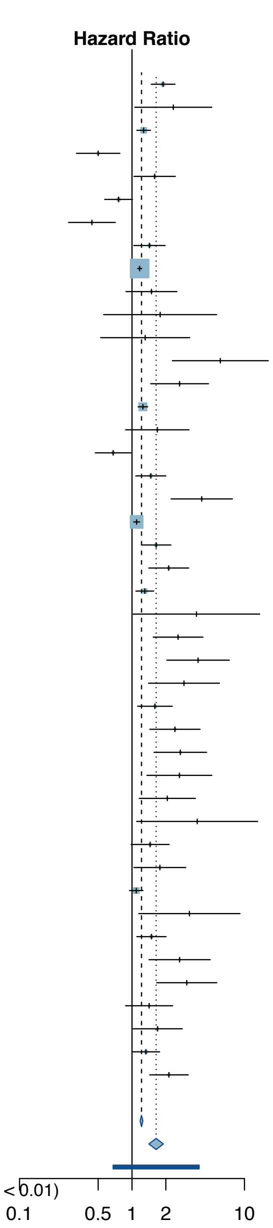

Heterogeneity: $I^2 = 81\%$ [75%; 85%], $\chi^2_{43} = 224.97$ ($p < 0.01$)

Tau²= 0.429

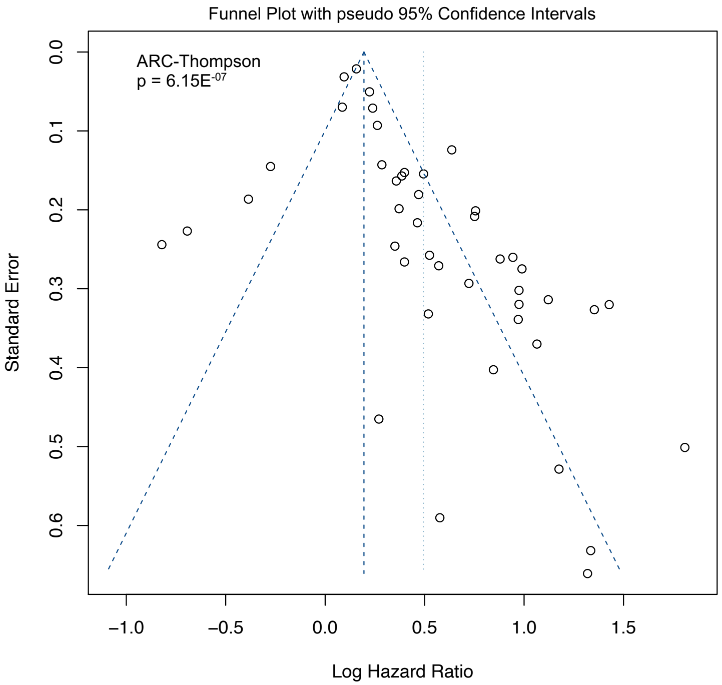

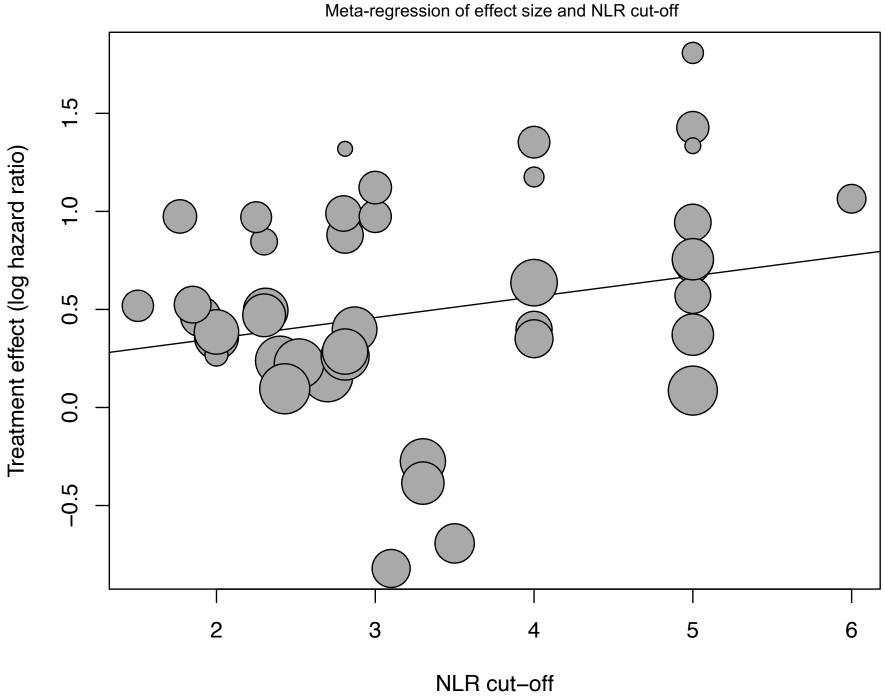

Supplementary Figure 58 - NLR: OS in hepatocellular carcinoma with transplant

Study	ES	seES	HR	95%–CI	Weight (fixed)	Weight (random)
Arai 2017	0.85	0.4027	2.33	[1.06; 5.13]	5.0%	10.9%
Dumitrascu 2013	−0.27	0.1451	0.76	[0.57; 1.01]	38.4%	15.4%
Halazun 2009	1.81	0.5012	6.10	[2.28; 16.29]	3.2%	9.2%
Limaye 2013	0.75	0.2085	2.12	[1.41; 3.19]	18.6%	14.5%
Motomura 2013	1.35	0.3266	3.87	[2.04; 7.34]	7.6%	12.3%
Na 2014	1.06	0.3701	2.90	[1.40; 5.99]	5.9%	11.5%
Wang 2011	0.97	0.3198	2.65	[1.42; 4.96]	7.9%	12.4%
Xiao 2015	0.35	0.2460	1.42	[0.88; 2.30]	13.4%	13.8%
**Pooled HR by Fixed Effects**			**1.53**	**[1.28; 1.82]**	**100.0%**	**--**
**Pooled HR by Random Effects**			**2.19**	**[1.40; 3.43]**	**--**	**100.0%**
**Prediction interval**				**[0.49; 9.71]**		

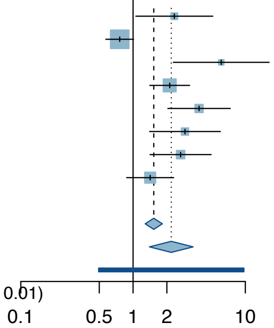

Heterogeneity: $I^2 = 86\%$ [74%; 92%], $\chi^2_7 = 48.51$ ($p < 0.01$)

Tau²= 0.564

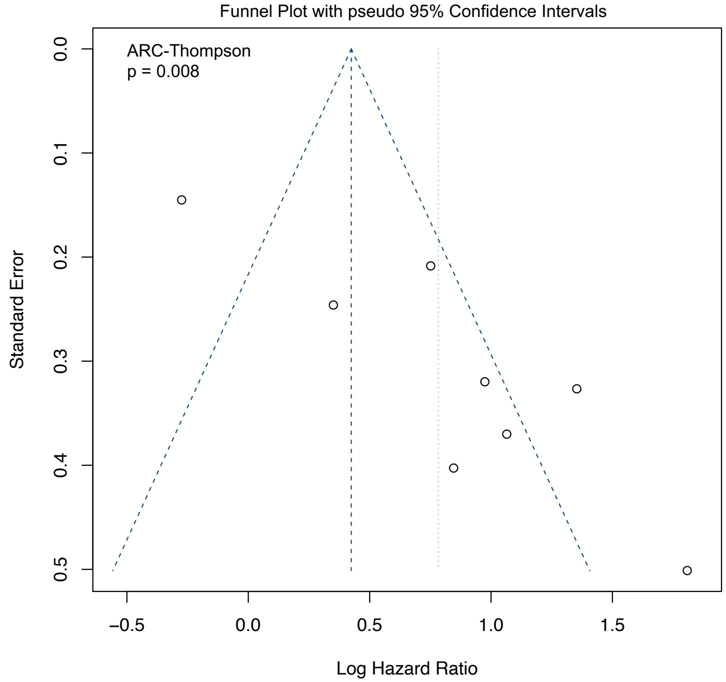

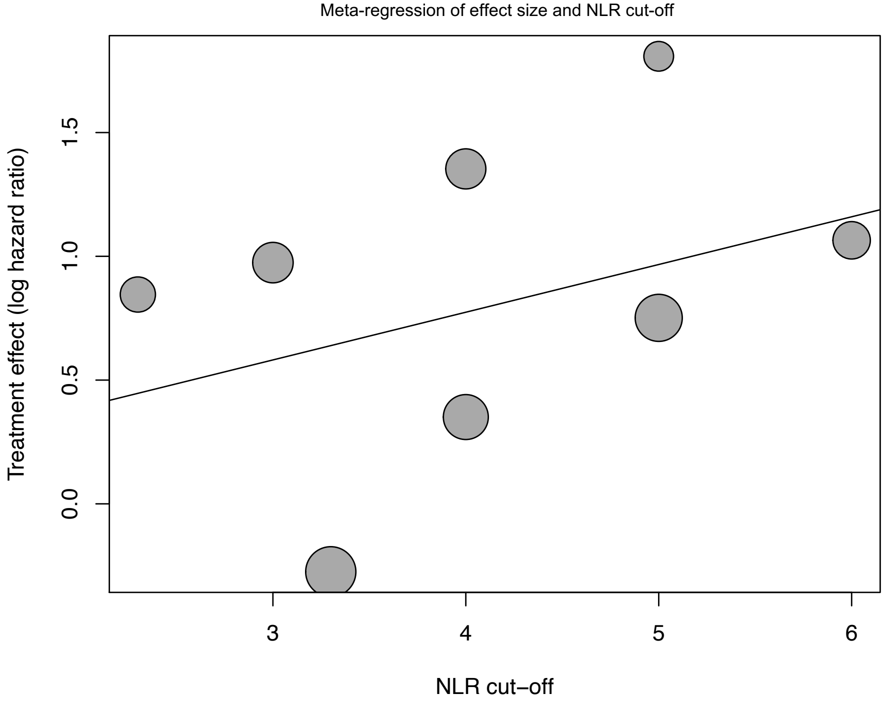

Supplementary Figure 59 - NLR: OS in hepatocellular carcinoma with radiofrequency ablation

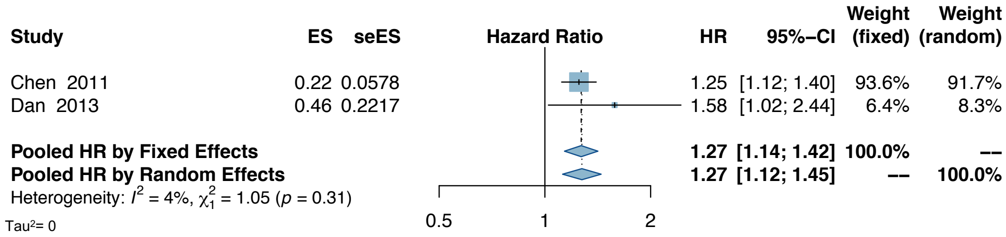

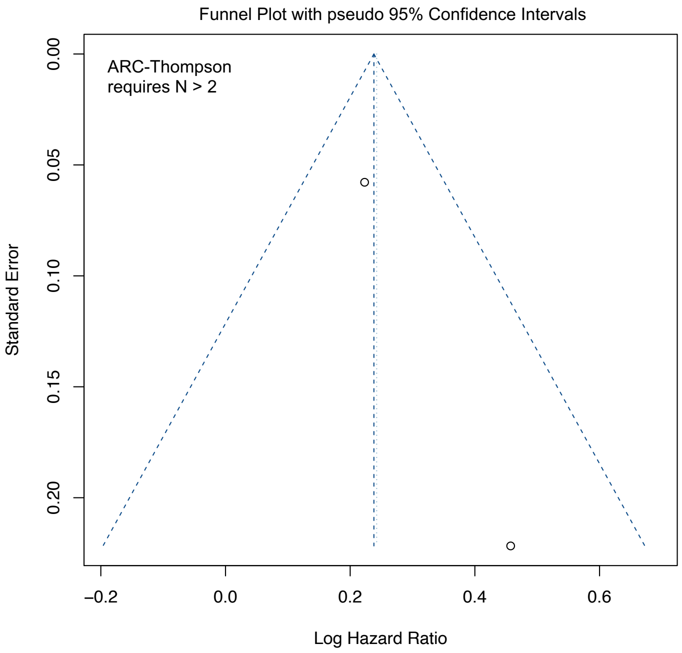

Supplementary Figure 60 - NLR: OS in hepatocellular carcinoma with mixed treatment

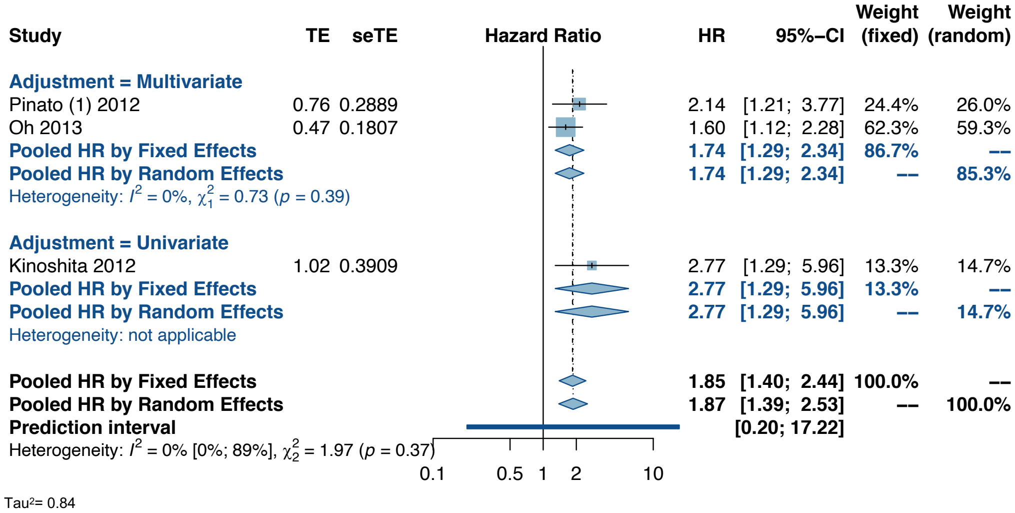

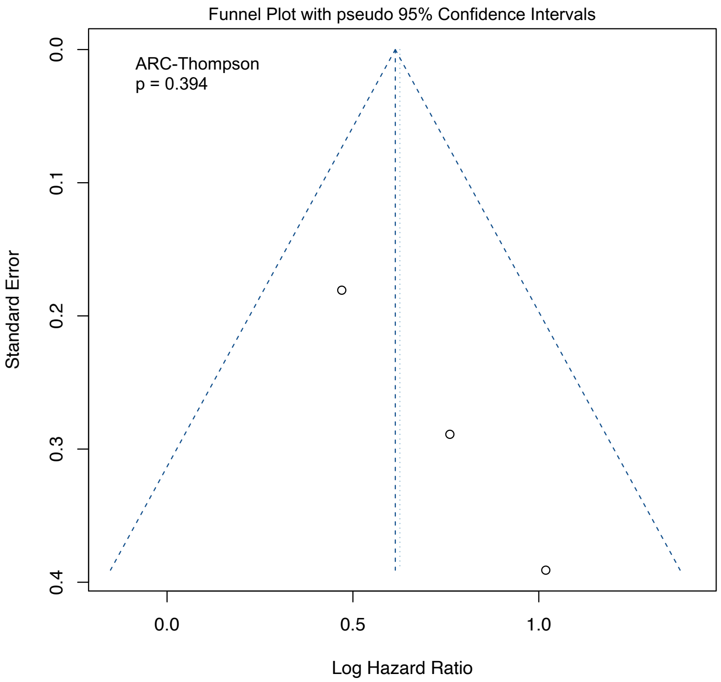

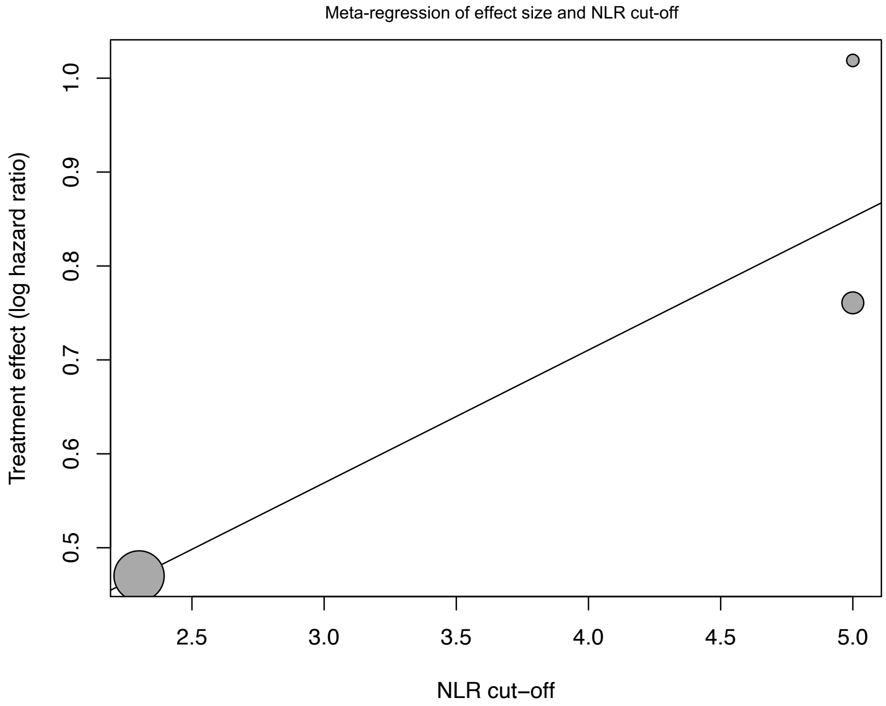

Supplementary Figure 61 - NLR: DFS in hepatocellular carcinoma

Study	ES	seES	HR	95%–CI	Weight (fixed)	Weight (random)
Chen 2012	0.05	0.0508	1.05	[0.95; 1.16]	47.1%	8.9%
Dumitrascu 2013	–0.25	0.1165	0.78	[0.62; 0.98]	9.0%	8.4%
Fu 2013	0.31	0.1458	1.36	[1.02; 1.81]	5.7%	8.1%
Gomez (HCC) 2008	0.95	0.4148	2.59	[1.15; 5.84]	0.7%	4.8%
Gomez (ICC) 2008	0.66	0.4887	1.93	[0.74; 5.03]	0.5%	4.1%
Guo 2009	1.25	0.4043	3.50	[1.58; 7.73]	0.7%	4.9%
Halazun 2009	2.13	0.5528	8.42	[2.85; 24.88]	0.4%	3.5%
Harimoto 2016	1.02	0.4766	2.77	[1.09; 7.05]	0.5%	4.2%
Ji 2016	0.34	0.1367	1.40	[1.07; 1.83]	6.5%	8.2%
Lai 2014	0.02	0.6803	1.02	[0.27; 3.87]	0.3%	2.7%
Laio 2014	0.52	0.1550	1.69	[1.25; 2.29]	5.1%	8.0%
Na 2014	0.92	0.4768	2.51	[0.99; 6.39]	0.5%	4.2%
Wang (A) 2011	1.30	0.3640	3.66	[1.79; 7.47]	0.9%	5.4%
Wang (P) 2011	1.02	0.4933	2.78	[1.06; 7.31]	0.5%	4.0%
Yang 2016	0.48	0.1282	1.61	[1.25; 2.07]	7.4%	8.3%
Yang 2017	0.32	0.0943	1.38	[1.15; 1.66]	13.7%	8.6%
Yoshizumi 2013	1.39	0.5429	4.02	[1.39; 11.65]	0.4%	3.6%
**Pooled HR by Fixed Effects**			**1.23**	**[1.15; 1.32]**	**100.0%**	**--**
**Pooled HR by Random Effects**			**1.82**	**[1.40; 2.37]**	**--**	**100.0%**
**Prediction interval**				**[0.67; 4.92]**		

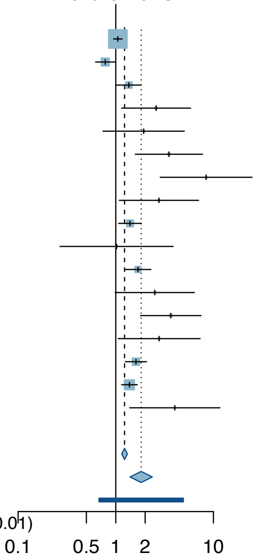

Heterogeneity: $I^2 = 80\%$ [69%; 87%], $\chi^2_{16} = 81.01$ ($p < 0.01$)

Tau^2= 0.447

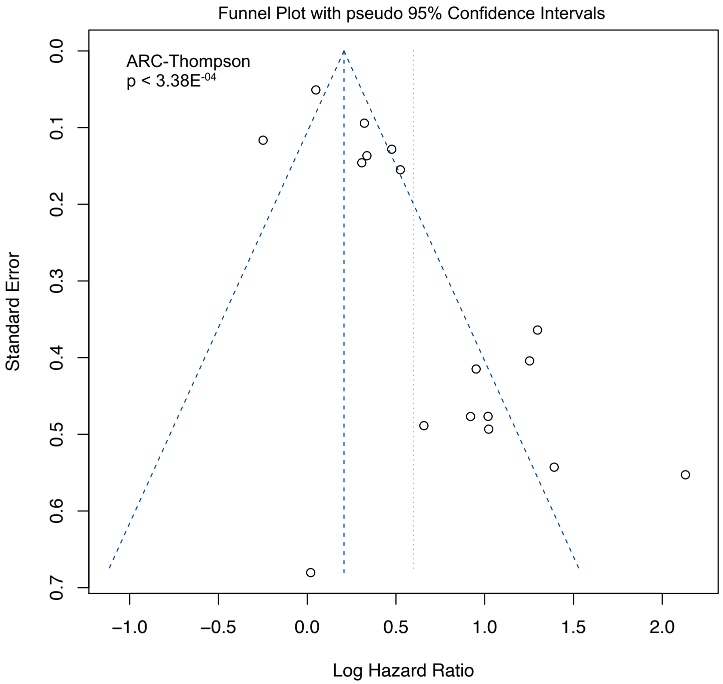

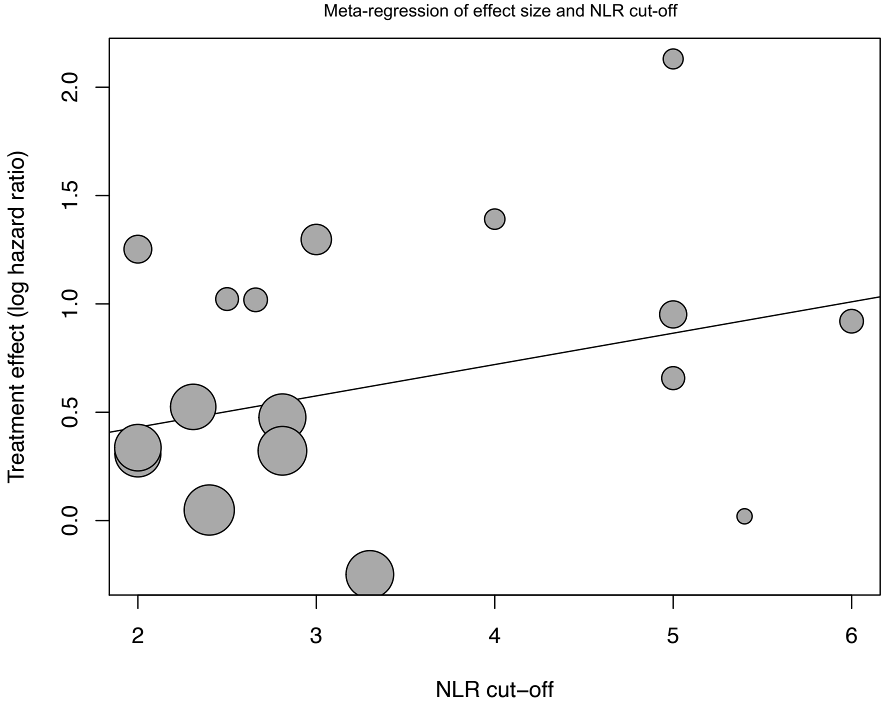

Supplementary Figure 62 - NLR: DFS in hepatocellular carcinoma with radiofrequency ablation

Study	ES	seES	HR	95%−CI	Weight (fixed)	Weight (random)
Chen 2011	0.24	0.2214	1.27	[0.82; 1.96]	36.7%	36.7%
Dan 2013	−0.03	0.1687	0.97	[0.70; 1.35]	63.3%	63.3%
**Pooled HR by Fixed Effects**			**1.07**	**[0.82; 1.39]**	**100.0%**	**--**
**Pooled HR by Random Effects**			**1.07**	**[0.82; 1.39]**	**--**	**100.0%**

Heterogeneity: $I^2 = 0\%$, $\chi^2_1 = 0.94$ ($p = 0.33$)

Tau²= 0

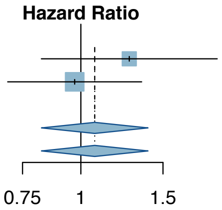

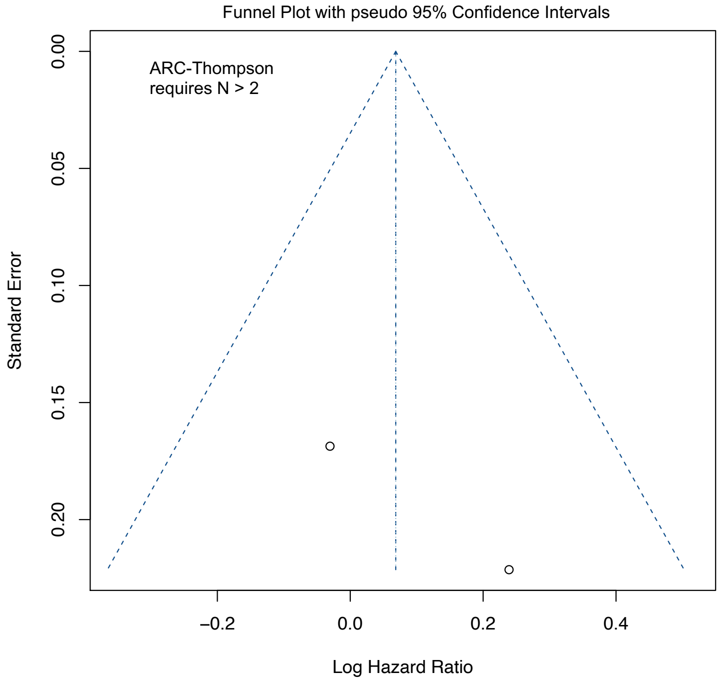

Supplementary Figure 63 - NLR: RFS in hepatocellular carcinoma

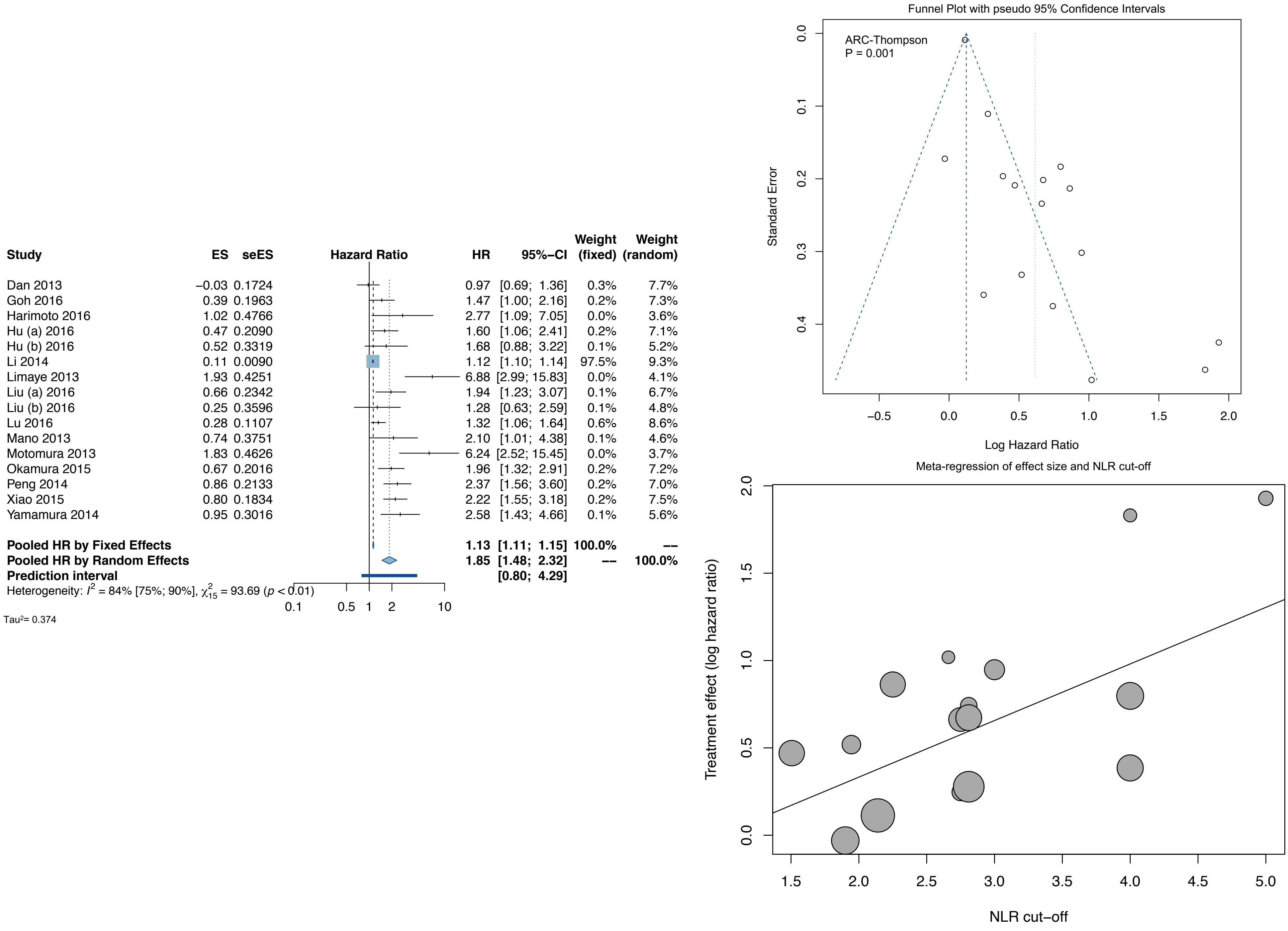

Supplementary Figure 64 - NLR: RFS in hepatocellular carcinoma with transplant

Study	ES	seES	HR	95%−CI	Weight (fixed)	Weight (random)
Harimoto 2016	1.02	0.4766	2.77	[1.09; 7.05]	9.9%	20.5%
Limaye 2013	1.93	0.4251	6.88	[2.99; 15.83]	12.5%	22.8%
Motomura 2013	1.83	0.4626	6.24	[2.52; 15.45]	10.5%	21.1%
Xiao 2015	0.80	0.1834	2.22	[1.55; 3.18]	67.1%	35.5%
**Pooled HR by Fixed Effects**			**2.91**	**[2.17; 3.91]**	**100.0%**	**--**
**Pooled HR by Random Effects**			**3.74**	**[2.05; 6.83]**	**--**	**100.0%**
**Prediction interval**				**[0.32; 43.60]**		

Heterogeneity: $I^2 = 67\%$ [3%; 89%], $\chi^2_3 = 9.01$ ($p = 0.03$)

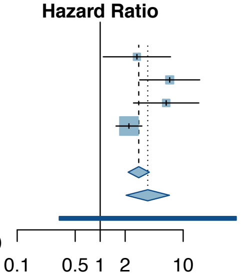

Tau²= 0.481

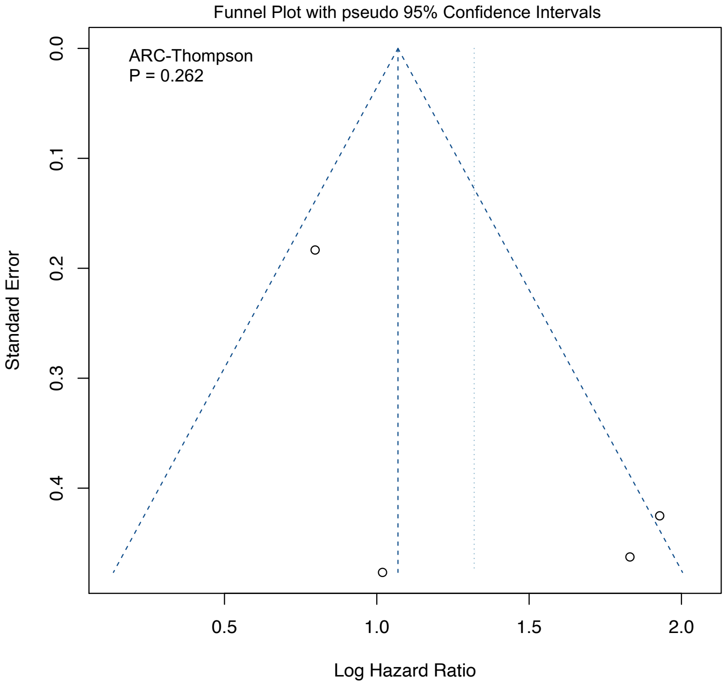

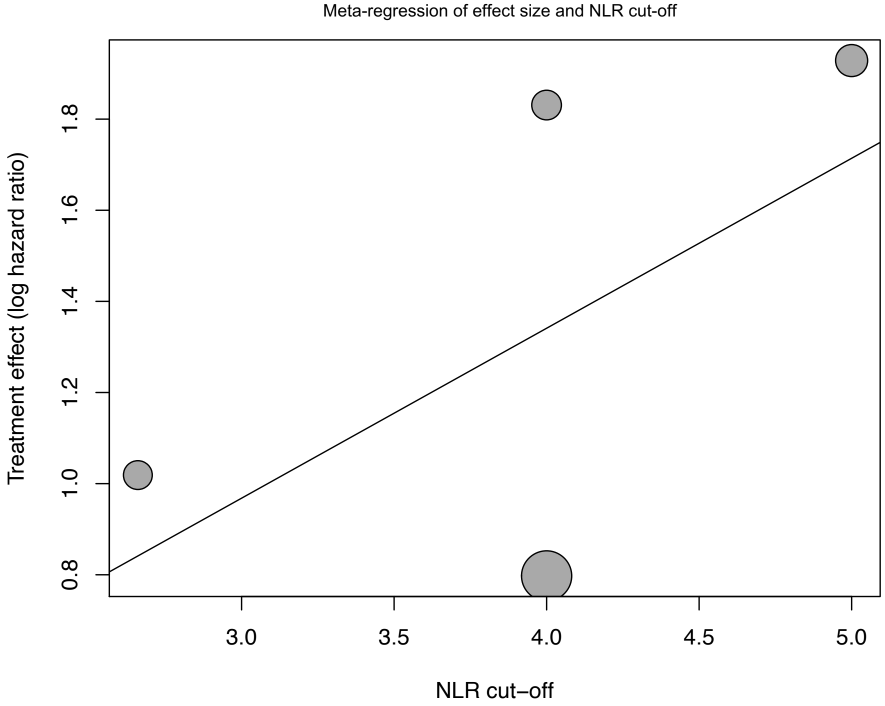

Supplementary Figure 65 - NLR: RFS in hepatocellular carcinoma with resection

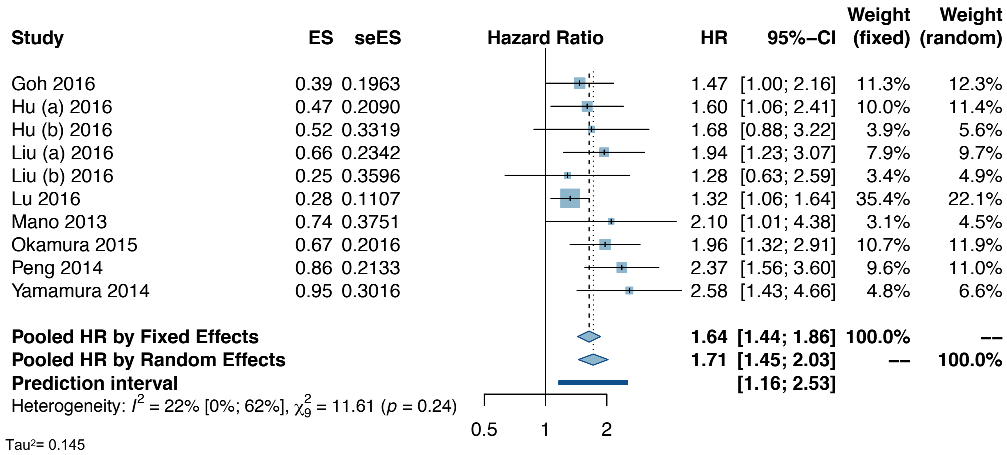

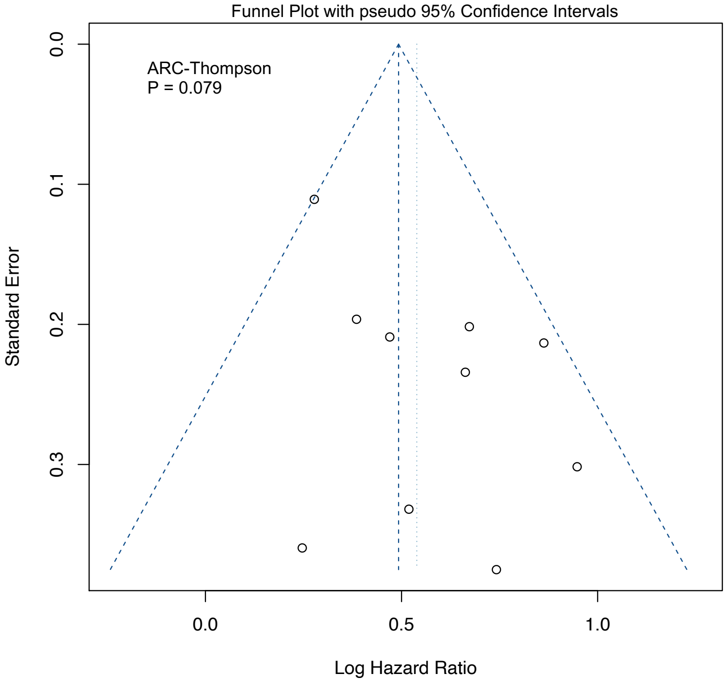

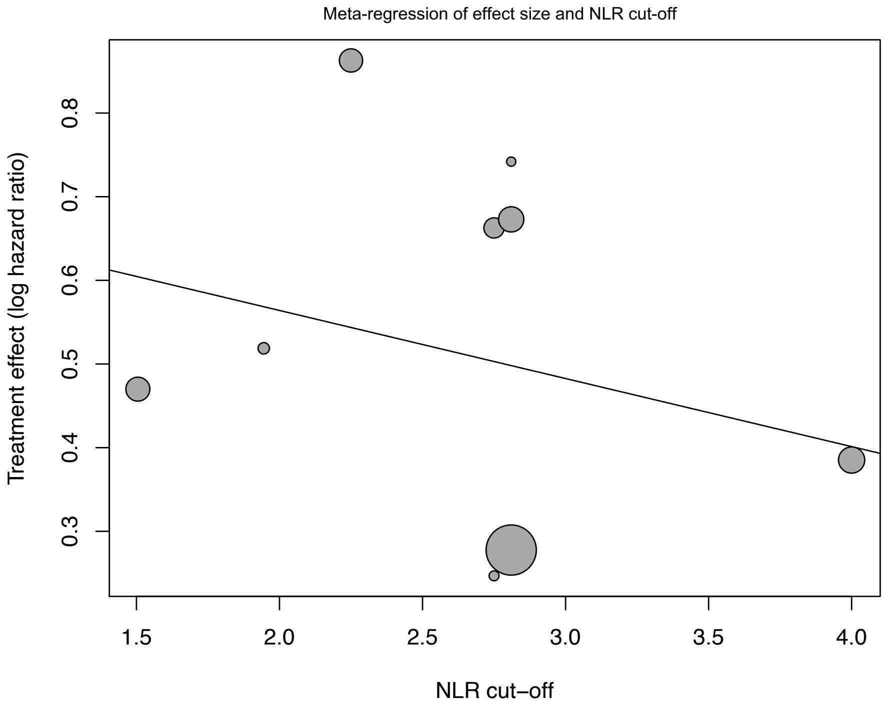

Supplementary Figure 66 - NLR: OS in biliary tract cancer

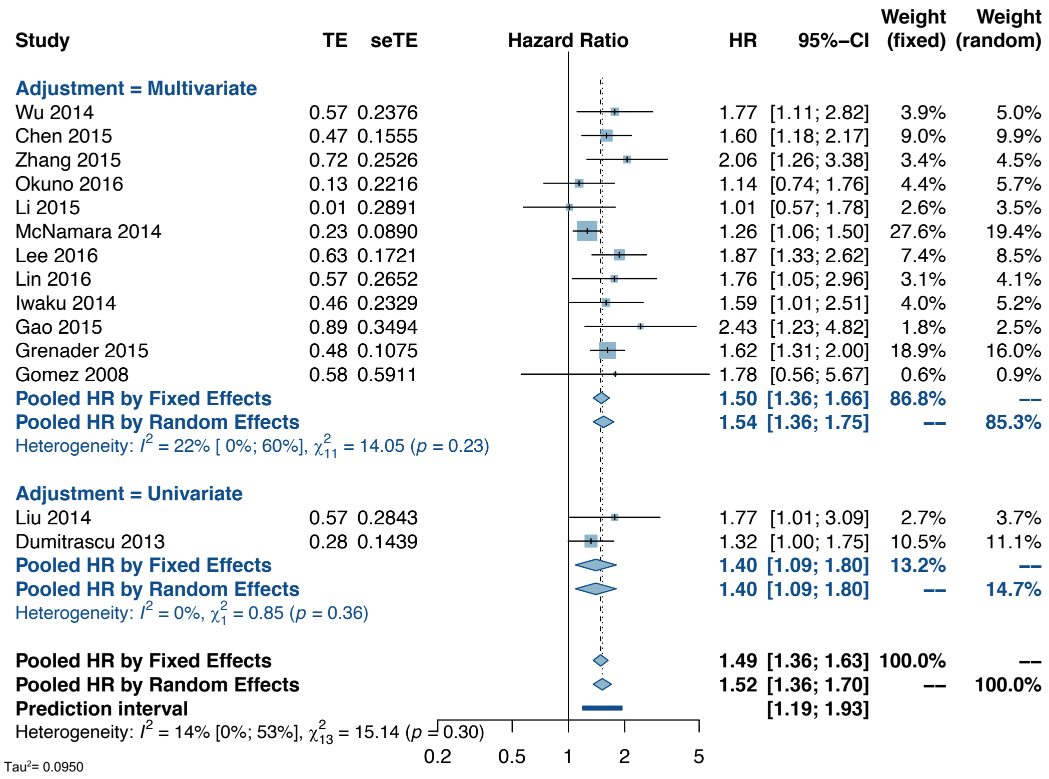

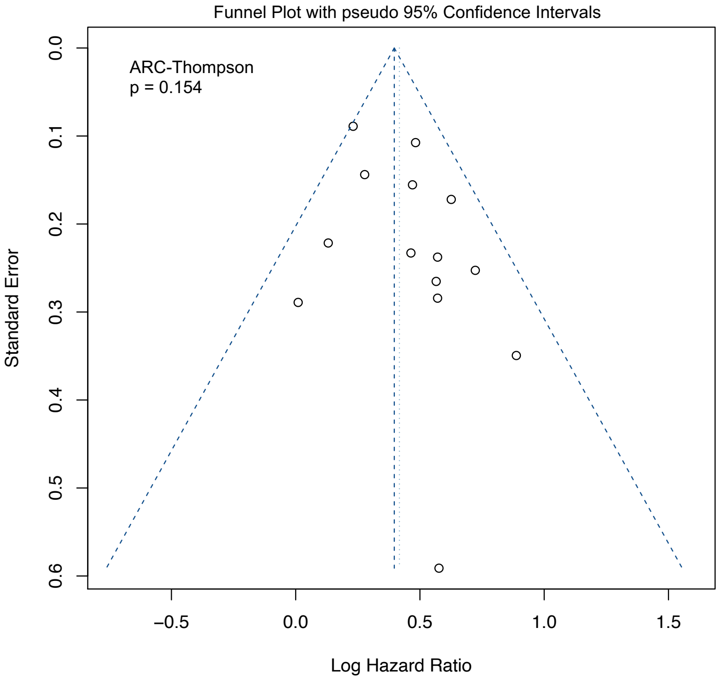

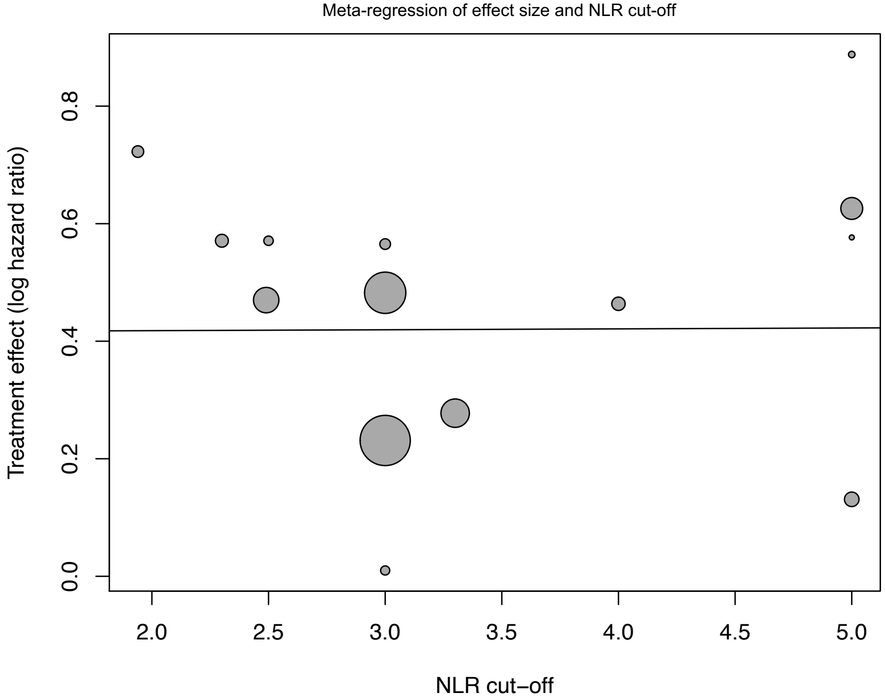

Supplementary Figure 67 - NLR: RFS in biliary tract cancer

Study	TE	seTE	HR	95%-CI	Weight (fixed)	Weight (random)
**Adjustment = Multivariate**						
Lin 2016	0.82	0.3051	2.26	[1.24; 4.11]	6.5%	7.9%
Gomez 2008	0.88	0.3039	2.42	[1.33; 4.39]	6.5%	7.9%
Chen 2015	0.22	0.1569	1.25	[0.92; 1.70]	24.4%	25.2%
**Pooled HR by Fixed Effects**			**1.55**	**[1.21; 1.99]**	**37.4%**	**---**
**Pooled HR by Random Effects**			**1.78**	**[1.13; 2.82]**	**---**	**41.0%**
Heterogeneity: $I^2 = 64\%$ [0%; 90%], $\chi^2_2 = 5.56$ ($p = 0.06$)						
**Adjustment = Univariate**						
Dumitrascu 2013	0.25	0.1170	1.28	[1.02; 1.61]	43.9%	38.8%
Liu 2014	0.30	0.1797	1.35	[0.95; 1.92]	18.6%	20.2%
**Pooled HR by Fixed Effects**			**1.30**	**[1.07; 1.58]**	**62.6%**	**---**
**Pooled HR by Random Effects**			**1.30**	**[1.07; 1.58]**	**---**	**59.0%**
Heterogeneity: $I^2 = 0\%$, $\chi^2_1 = 0.06$ ($p = 0.80$)						
**Pooled HR by Fixed Effects**			**1.39**	**[1.19; 1.62]**	**100.0%**	**---**
**Pooled HR by Random Effects**			**1.41**	**[1.19; 1.68]**	**---**	**100.0%**
**Prediction interval**				**[0.97; 2.07]**		
Heterogeneity: $I^2 = 42\%$ [0%; 78%], $\chi^2_4 = 6.85$ ($p = 0.14$)						

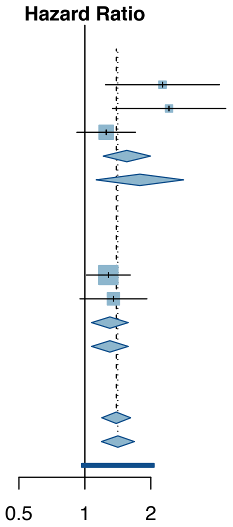

Tau²= 0.080

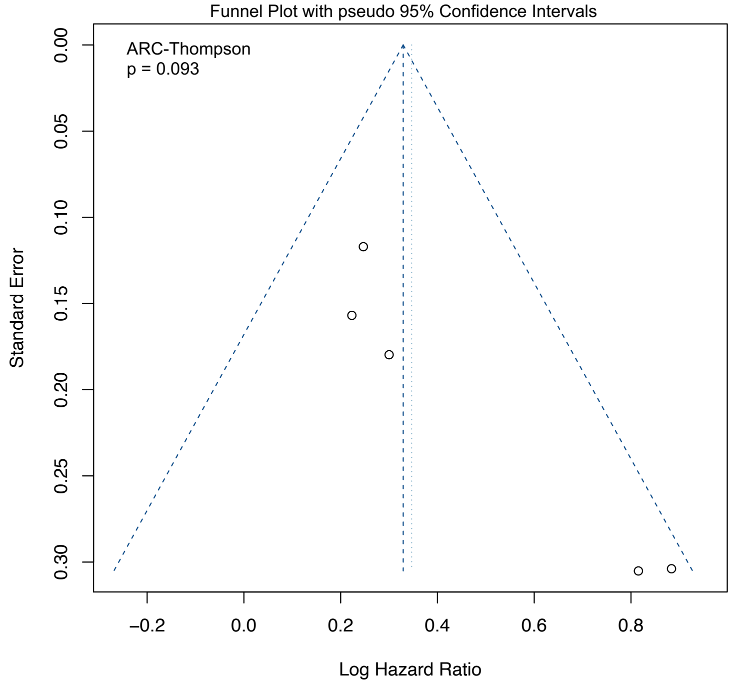

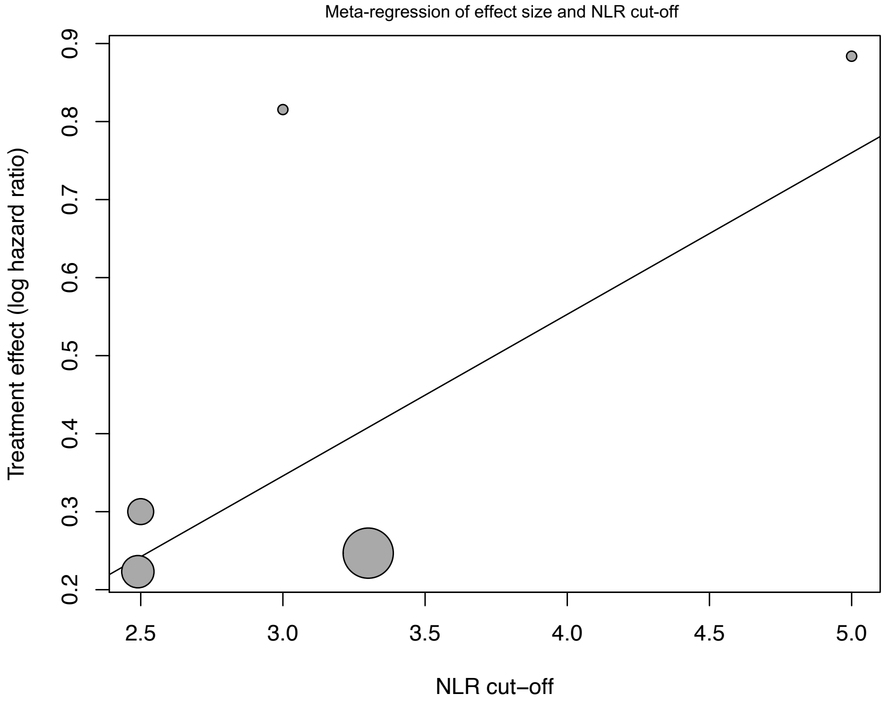

Supplementary Figure 68 - NLR: OS in large-B cell cancer

Study	ES	seES	HR	95%–CI	Weight (fixed)	Weight (random)
Porrata 2010	1.41	0.3623	4.10	[2.02; 8.34]	6.9%	10.6%
Melchardt 2015	0.13	0.1824	1.14	[0.80; 1.63]	27.2%	13.4%
Zhu 2015	1.57	0.6562	4.83	[1.33; 17.48]	2.1%	6.5%
Ho 2015	0.48	0.3457	1.62	[0.82; 3.19]	7.6%	10.9%
Lee 2015	0.77	0.3688	2.15	[1.04; 4.43]	6.6%	10.5%
Ni 2016	1.71	0.8417	5.55	[1.07; 28.89]	1.3%	4.8%
Wang 2016	−0.92	0.3785	0.40	[0.19; 0.84]	6.3%	10.3%
Perisa 2016	1.05	0.5676	2.86	[0.94; 8.70]	2.8%	7.5%
Keam 2016	0.65	0.1925	1.92	[1.32; 2.80]	24.4%	13.2%
Gao 2017	0.52	0.2465	1.69	[1.04; 2.74]	14.9%	12.4%
**Pooled HR by Fixed Effects**			**1.62**	**[1.35; 1.95]**	**100.0%**	**--**
**Pooled HR by Random Effects**			**1.86**	**[1.20; 2.88]**	**--**	**100.0%**
**Prediction interval**				**[0.44; 7.85]**		

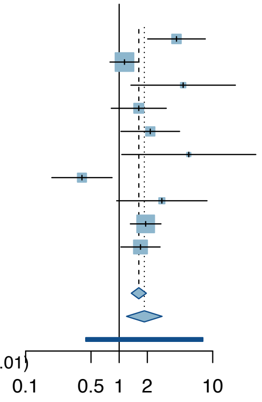

Heterogeneity: $I^2 = 71\%$ [45%; 85%], $\chi^2_9 = 31.24$ ($p < 0.01$)

Tau²= 0.584

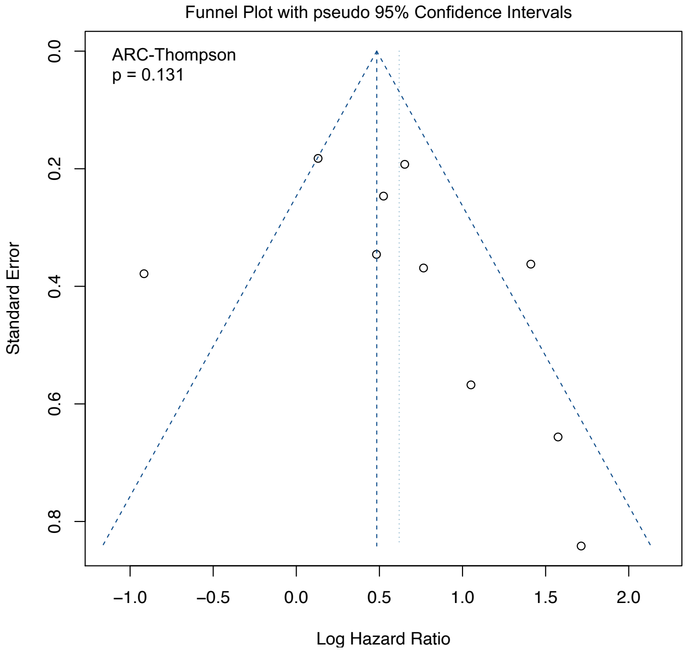

Supplementary Figure 69 - NLR: PFS in large-B cell cancer

Study	ES	seES	HR	95%–CI	Weight (fixed)	Weight (random)
Lee 2015	0.36	0.3319	1.44	[0.75; 2.76]	7.9%	10.8%
Ni 2016	0.94	0.4599	2.57	[1.04; 6.33]	4.1%	8.3%
Hong 2016	0.30	0.2561	1.35	[0.82; 2.23]	13.3%	12.5%
Keam 2016	0.43	0.1912	1.54	[1.06; 2.24]	23.8%	13.9%
Perisa 2016	1.40	0.5593	4.06	[1.36; 12.15]	2.8%	6.7%
Ho 2015	0.58	0.2980	1.79	[1.00; 3.21]	9.8%	11.6%
Gao 2016	0.16	0.2112	1.17	[0.77; 1.77]	19.5%	13.5%
Porrata 2010	1.34	0.3442	3.82	[1.95; 7.50]	7.4%	10.6%
Wang 2016	−0.56	0.2765	0.57	[0.33; 0.98]	11.4%	12.1%
**Pooled HR by Fixed Effects**			**1.45**	**[1.21; 1.74]**	**100.0%**	**--**
**Pooled HR by Random Effects**			**1.60**	**[1.10; 2.33]**	**--**	**100.0%**
**Prediction interval**				**[0.48; 5.36]**		

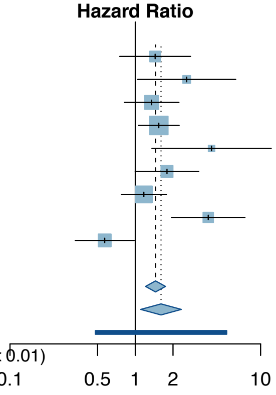

Heterogeneity: $I^2 = 69\%$ [38%; 85%], $\chi^2_8 = 25.97$ ($p < 0.01$)

Tau²= 0.473

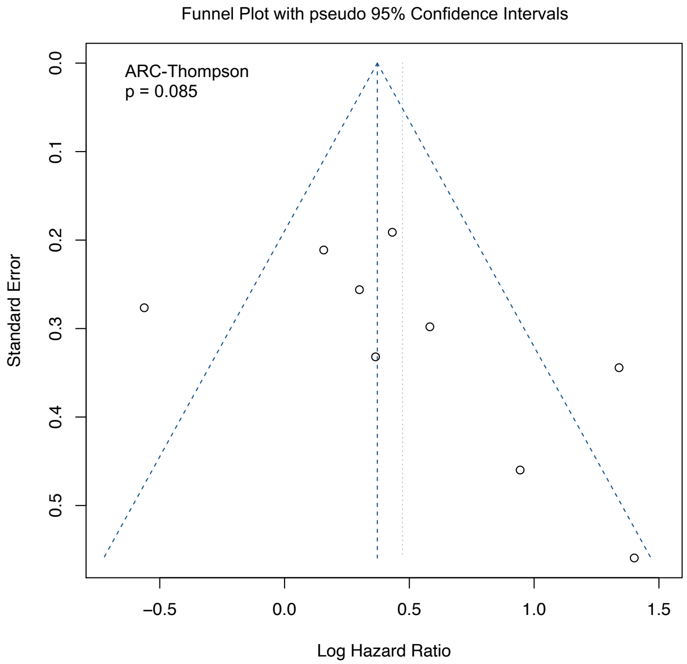

Supplementary *Figure 70* - NLR: OS in gastroenteropancreatic neuroendocrine tumour

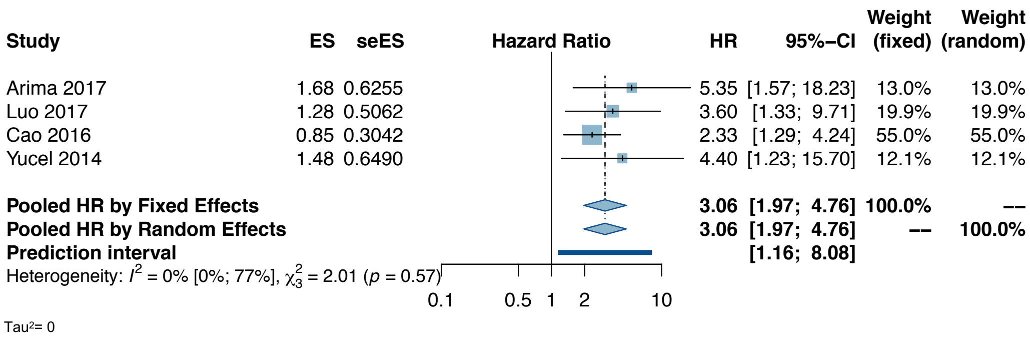

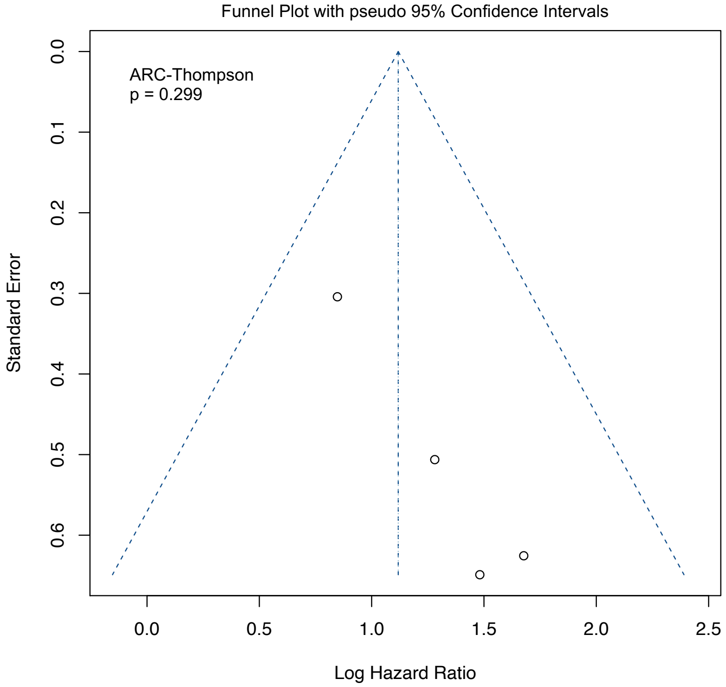

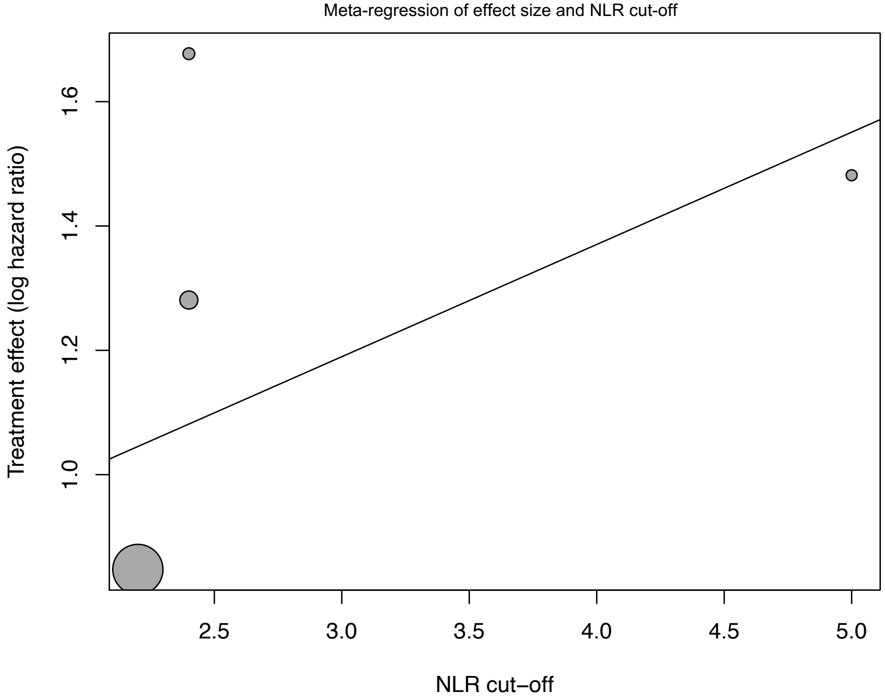

Supplementary *Figure 71* - NLR: RFS in gastroenteropancreatic neuroendocrine tumour

Study	ES	seES	HR	95%–CI	Weight (fixed)	Weight (random)
Arima 2017	1.79	0.6432	6.01	[1.70; 21.20]	14.7%	14.7%
Tong 2017	1.51	0.7455	4.52	[1.05; 19.47]	10.9%	11.0%
Cao 2016	1.01	0.2854	2.75	[1.57; 4.81]	74.4%	74.3%
**Pooled HR by Fixed Effects**			**3.26**	**[2.01; 5.28]**	**100.0%**	**--**
**Pooled HR by Random Effects**			**3.26**	**[2.01; 5.29]**	**--**	**100.0%**
**Prediction interval**				**[0.14; 76.19]**		

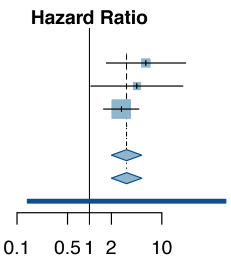

Heterogeneity: $I^2 = 0\%$ [0%; 86%], $\chi^2_2 = 1.45$ ($p = 0.48$)

Tau²= 0.024

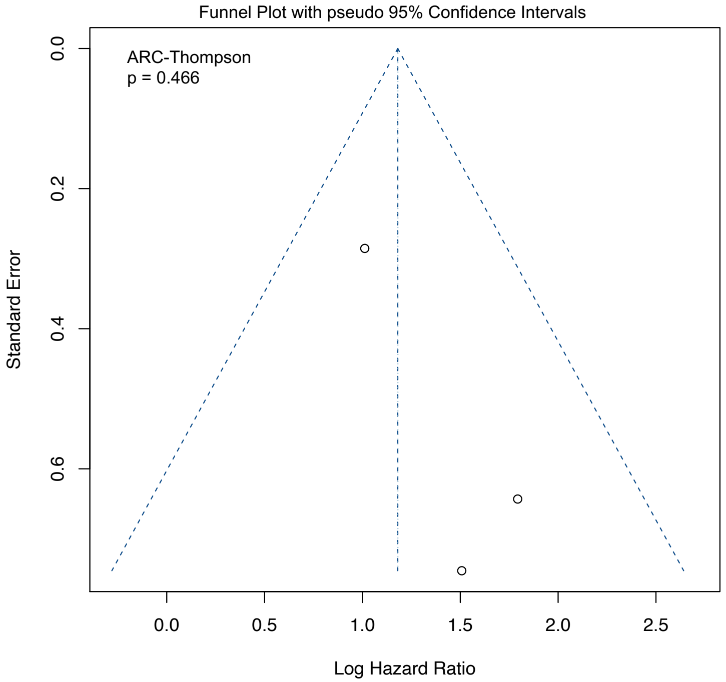

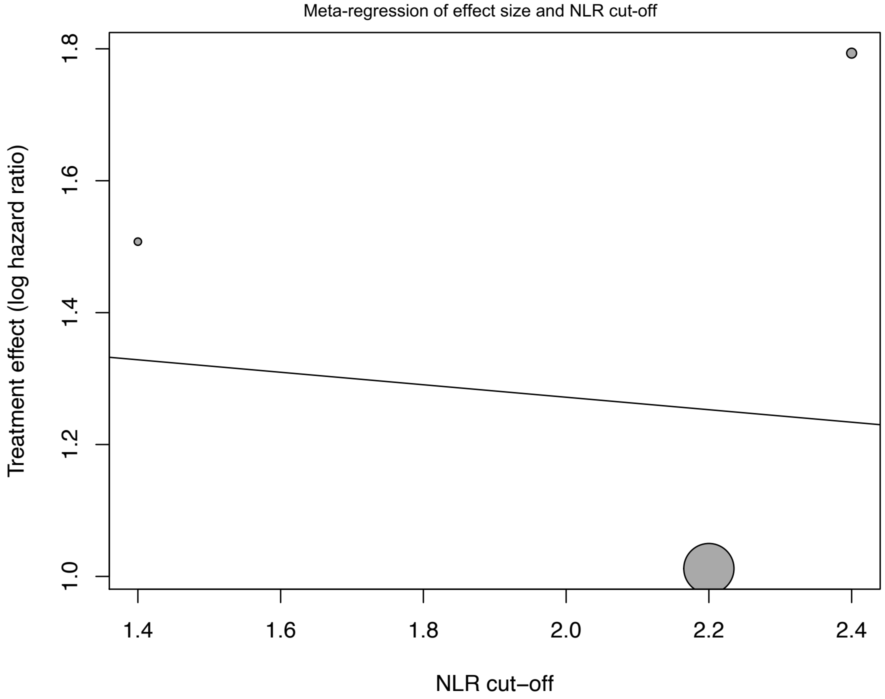

Supplementary *Figure 72* - NLR: OS in pancreatic neuroendocrine tumour

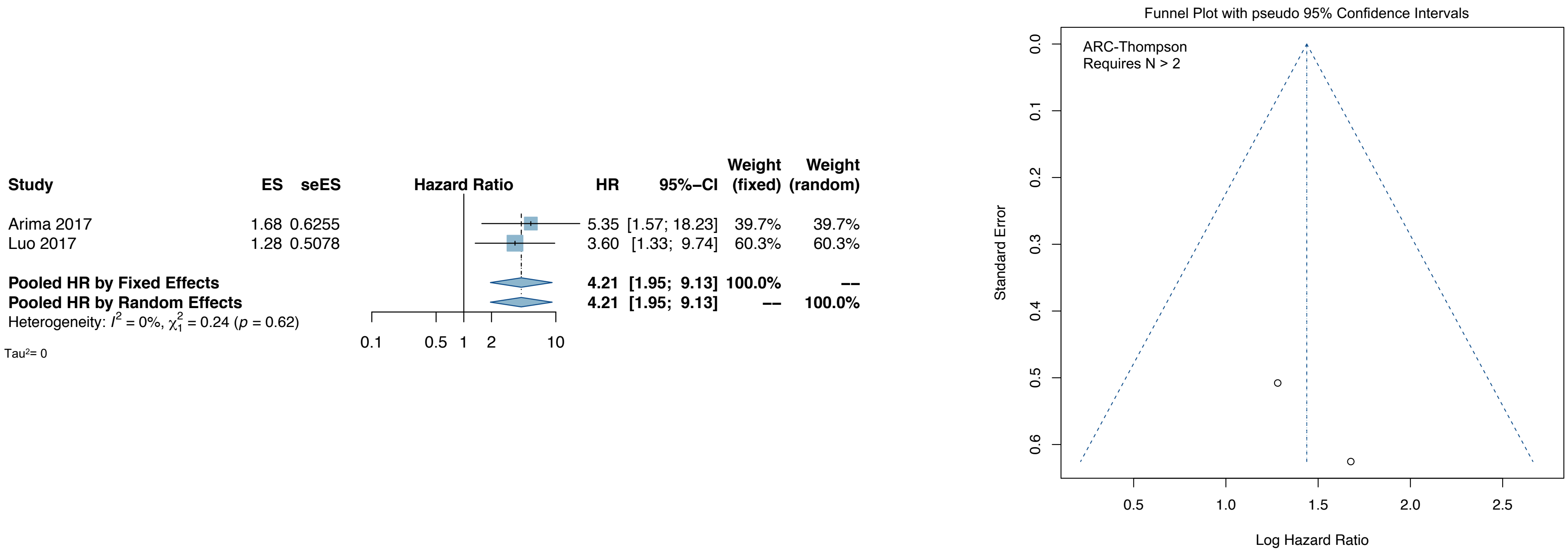

Supplementary *Figure 73* - NLR: RFS in pancreatic neuroendocrine tumour

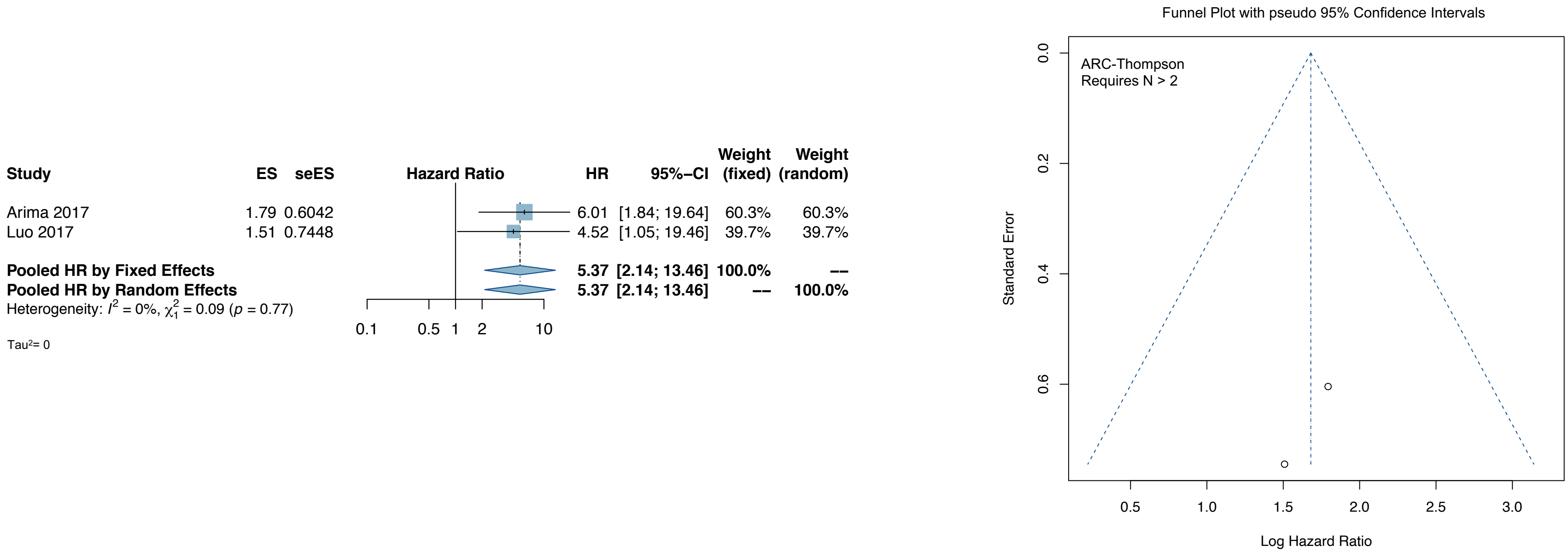

Supplementary *Figure 74* – IN: OS in non-small cell lung cancer

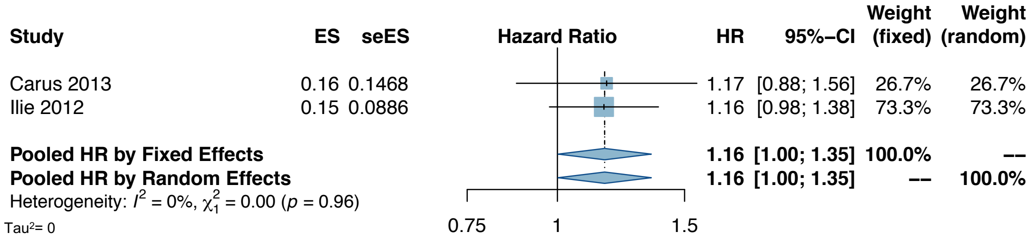

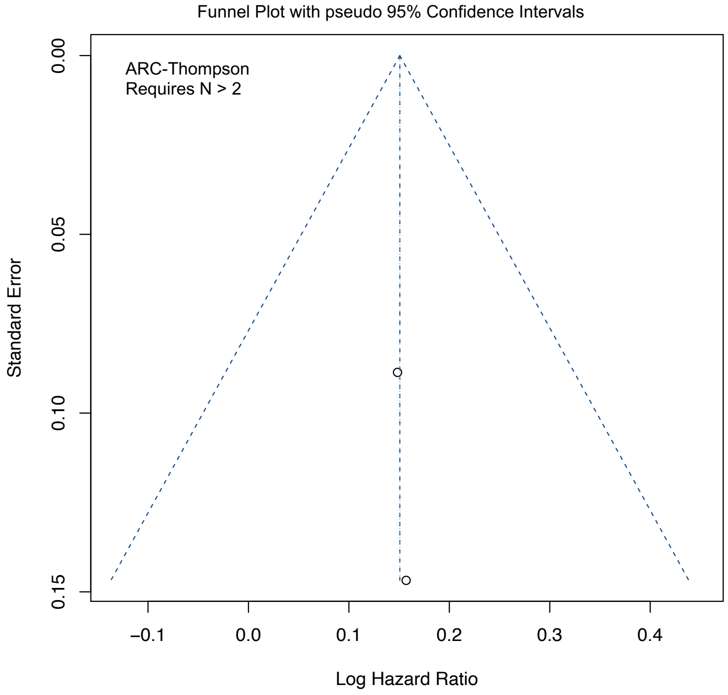

Supplementary *Figure 75* - NLR: PFS in non-small cell lung cancer

Study	ES	seES	HR	95%–CI	Weight (fixed)	Weight (random)
Teramukai 2009	0.41	0.1619	1.50	[1.09; 2.06]	2.8%	12.4%
Lee 2012	0.02	0.0292	1.02	[0.96; 1.08]	85.1%	17.3%
Botta (1) 2013	0.51	0.2637	1.67	[1.00; 2.80]	1.0%	8.3%
Botta (2) 2013	0.52	0.4515	1.68	[0.69; 4.07]	0.4%	4.1%
Forget 2013	0.61	0.2902	1.84	[1.04; 3.25]	0.9%	7.5%
Jafri 2013	0.54	0.1657	1.72	[1.24; 2.38]	2.6%	12.2%
Unal 2013	0.59	0.2254	1.80	[1.16; 2.80]	1.4%	9.7%
Yao 2013	0.59	0.2492	1.81	[1.11; 2.95]	1.2%	8.8%
Lin 2014	1.36	0.3470	3.89	[1.97; 7.68]	0.6%	6.0%
Choi 2015	0.31	0.1336	1.37	[1.05; 1.78]	4.1%	13.7%
**Pooled HR by Fixed Effects**			**1.10**	**[1.04; 1.15]**	**100.0%**	**--**
**Pooled HR by Random Effects**			**1.59**	**[1.29; 1.95]**	**--**	**100.0%**
**Prediction interval**				**[0.85; 2.97]**		

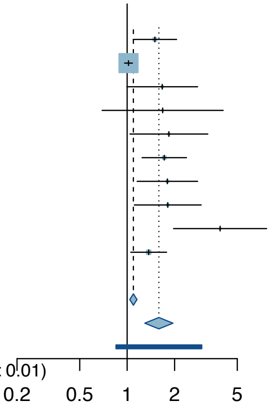

Heterogeneity: $I^2 = 82\%$ [67%; 90%], $\chi^2_9 = 48.87$ ($p < 0.01$)

Tau²= 0.251

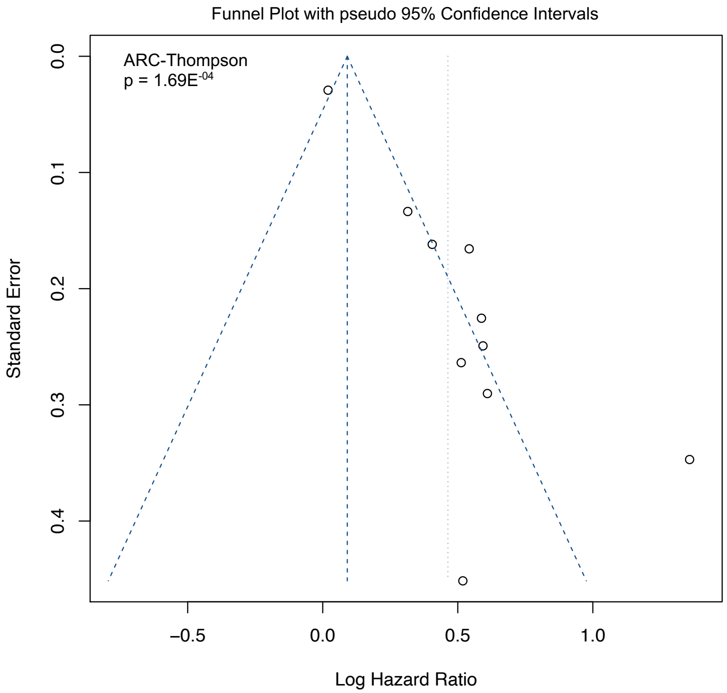

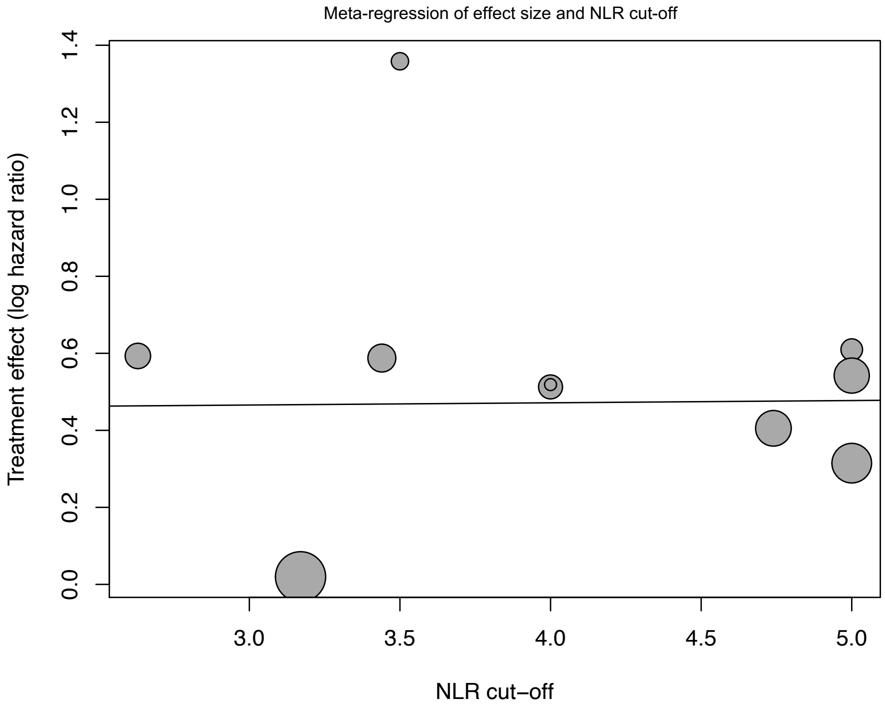

Supplementary *Figure 76* – NLR: OS in lung cancer

Study	TE	seTE	HR	95%-CI	Weight (fixed)	Weight (random)
**Adjustment = Multivariate**						
Choi 2015	0.52	0.1415	1.69	[1.28; 2.23]	2.1%	5.8%
Kos 2015	0.44	0.2177	1.56	[1.02; 2.39]	0.9%	4.6%
Go 2014	0.28	0.3556	1.32	[0.66; 2.65]	0.3%	2.8%
Kang 2014	0.39	0.1868	1.47	[1.02; 2.12]	1.2%	5.0%
Kacan 2014	0.53	0.2360	1.70	[1.07; 2.70]	0.7%	4.3%
Lin 2014	1.19	0.3636	3.29	[1.61; 6.71]	0.3%	2.8%
Pinato 2014	1.34	0.4342	3.80	[1.62; 8.90]	0.2%	2.2%
Wang 2014	0.53	0.2454	1.70	[1.05; 2.75]	0.7%	4.2%
Zhang 2014	0.73	0.2308	2.08	[1.32; 3.27]	0.8%	4.4%
Forget 2013	0.58	0.2977	1.78	[0.99; 3.19]	0.5%	3.5%
Yao 2013	0.57	0.2423	1.76	[1.09; 2.83]	0.7%	4.2%
Yildirim 2013	0.85	0.2148	2.33	[1.53; 3.55]	0.9%	4.6%
Kaya 2013	0.65	0.1897	1.91	[1.32; 2.77]	1.1%	5.0%
Cedr.s 2012	0.41	0.1717	1.50	[1.07; 2.10]	1.4%	5.3%
Lee 2012	0.12	0.0349	1.13	[1.06; 1.21]	33.8%	7.0%
Sarraf 2009	0.10	0.0315	1.10	[1.03; 1.17]	41.5%	7.1%
Teramukai 2009	0.44	0.1846	1.56	[1.09; 2.24]	1.2%	5.1%
**Pooled HR by Fixed Effects**			**1.19**	**[1.14; 1.24]**	**88.3%**	**--**
**Pooled HR by Random Effects**			**1.61**	**[1.40; 1.86]**	**--**	**77.7%**
Heterogeneity: $I^2 = 76\%$ [62%; 85%], $\chi^2_{16} = 67.13$ ($p < 0.01$)						
**Adjustment = Univariate**						
Cannon 2015	0.65	0.2411	1.92	[1.20; 3.08]	0.7%	4.2%
Mitchell 2015	0.31	0.1249	1.37	[1.07; 1.75]	2.6%	6.0%
Zhang 2015	0.43	0.0772	1.53	[1.32; 1.78]	6.9%	6.7%
Jafri 2013	−0.56	0.1665	0.57	[0.41; 0.79]	1.5%	5.4%
**Pooled HR by Fixed Effects**			**1.34**	**[1.19; 1.50]**	**11.7%**	**--**
**Pooled HR by Random Effects**			**1.22**	**[0.74; 2.04]**	**--**	**22.3%**
Heterogeneity: $I^2 = 90\%$ [79%; 96%], $\chi^2_{3} = 31.55$ ($p < 0.01$)						
**Pooled HR by Fixed Effects**			**1.21**	**[1.16; 1.25]**	**100.0%**	**--**
**Pooled HR by Random Effects**			**1.55**	**[1.33; 1.80]**	**--**	**100.0%**
**Prediction interval**				**[0.83; 2.89]**		
Heterogeneity: $I^2 = 80\%$ [71%; 87%], $\chi^2_{20} = 102.04$ ($p < 0.01$)						

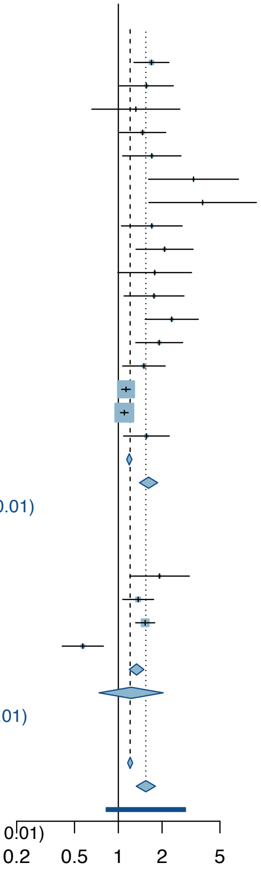

Tau²= 0.289

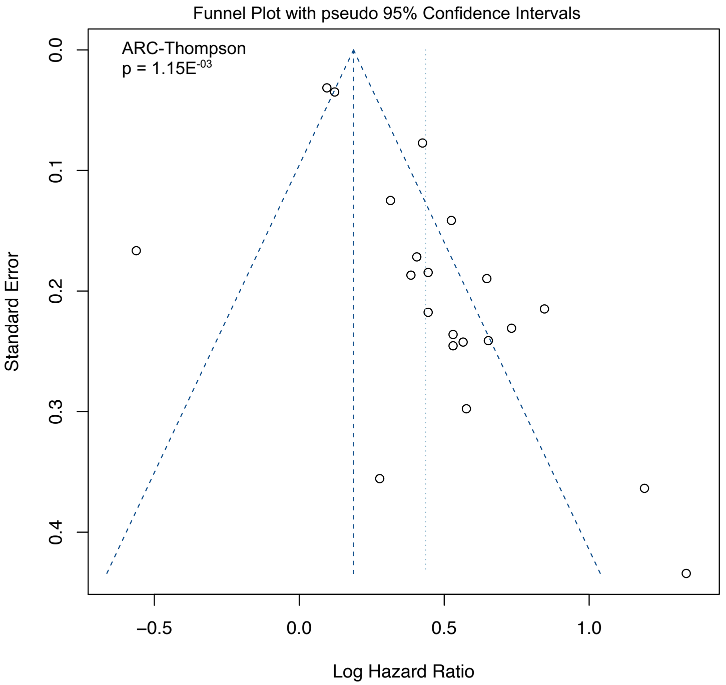

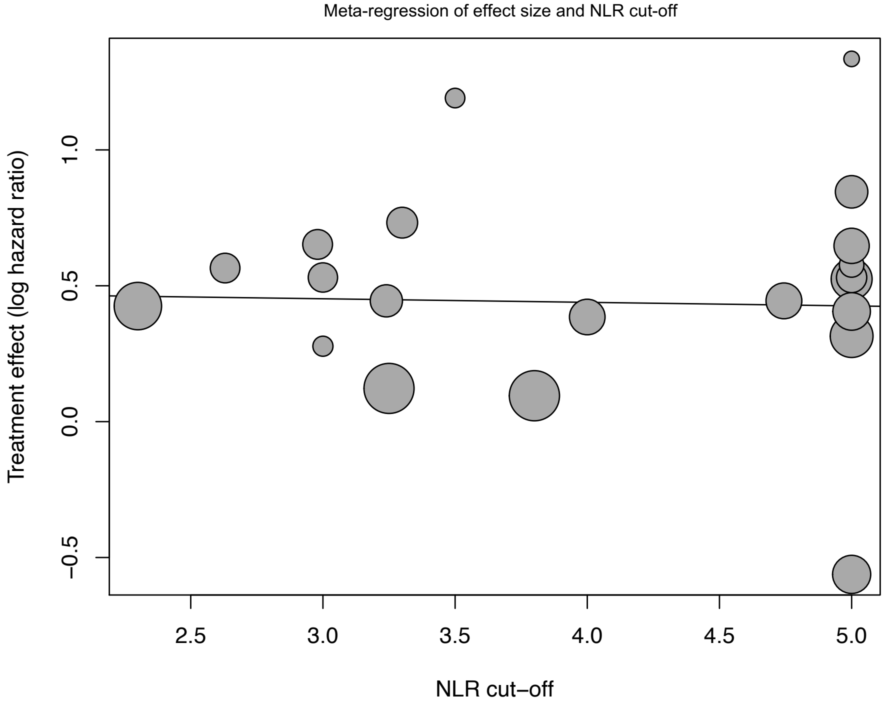

Supplementary *Figure 77* – NLR: PFS in lung cancer

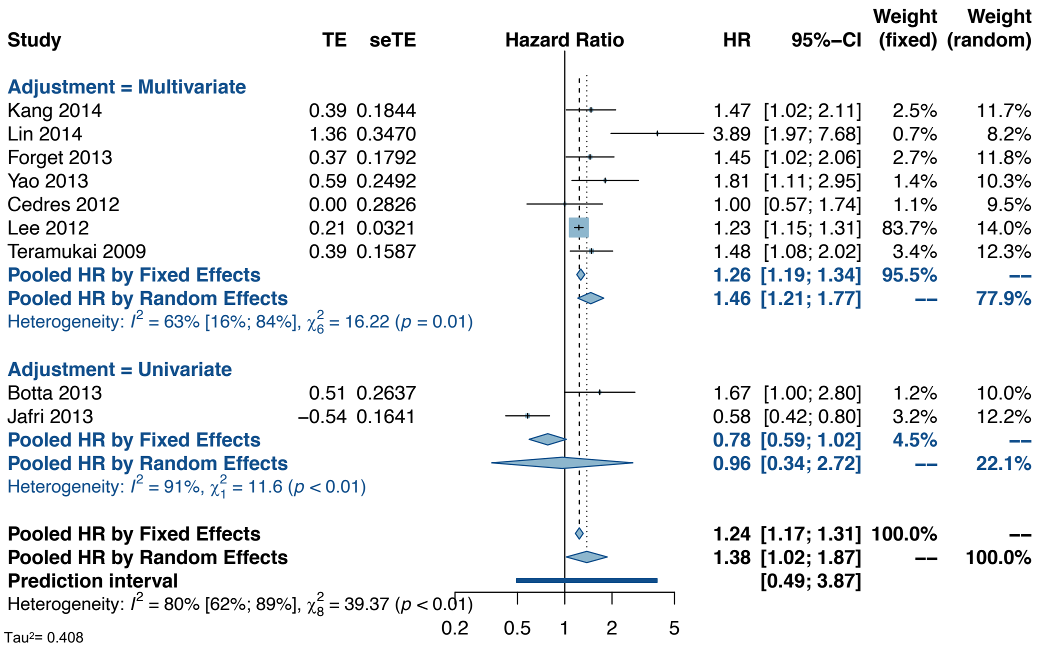

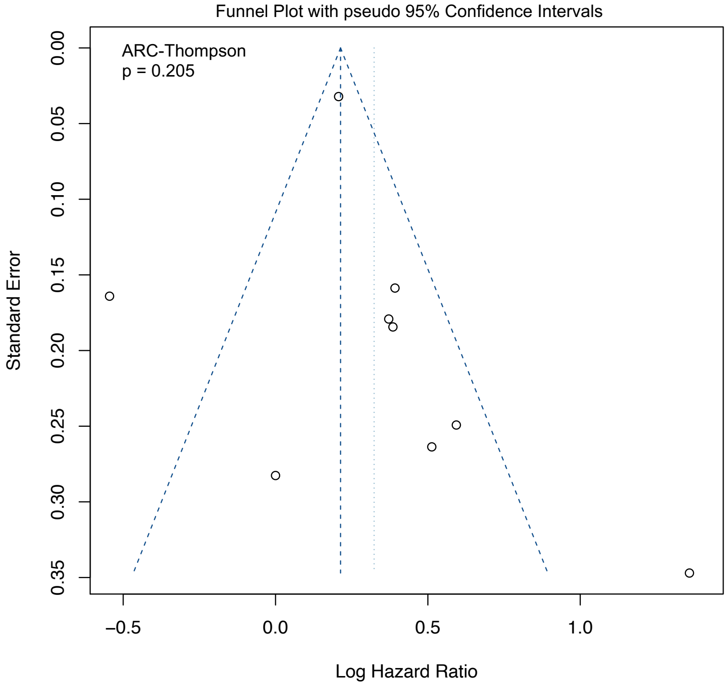

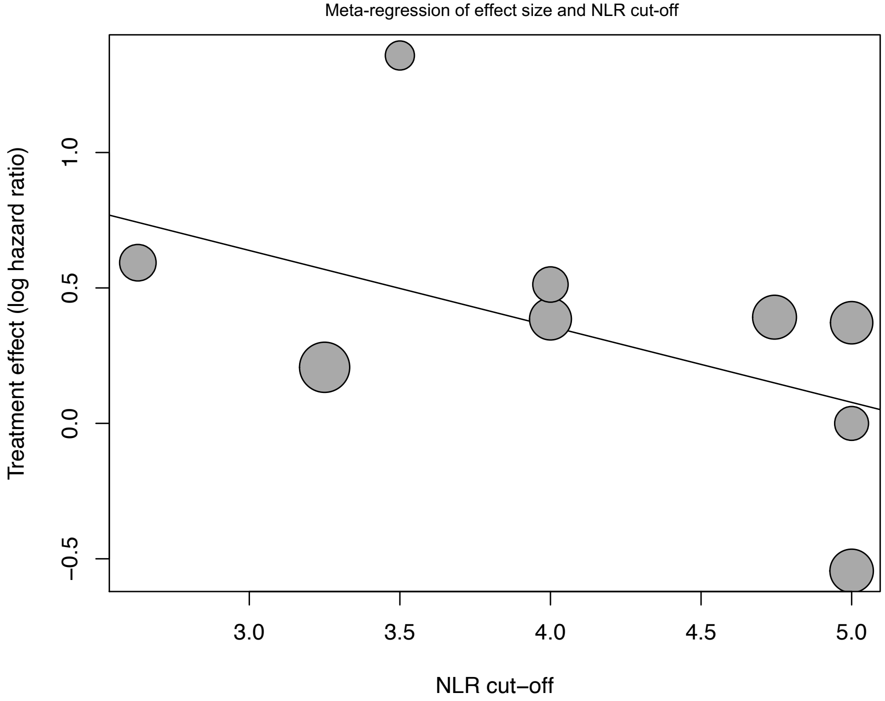

Supplementary *Figure 78* – NLR: OS in lung cancer with surgery

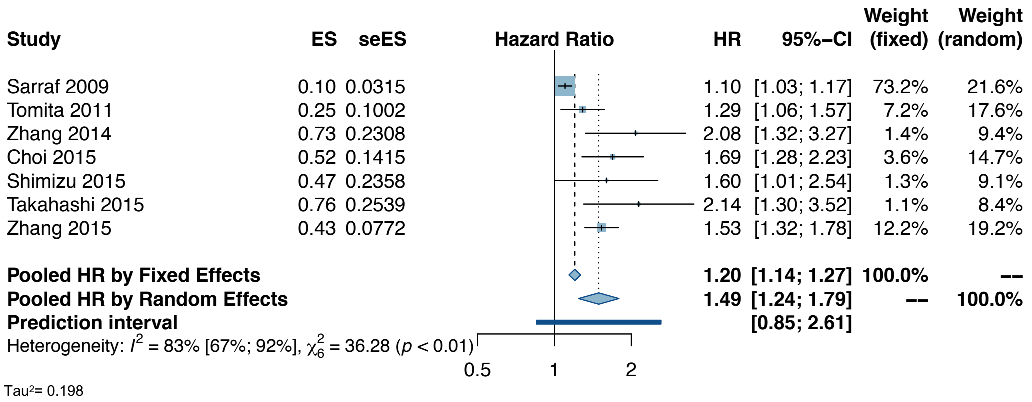

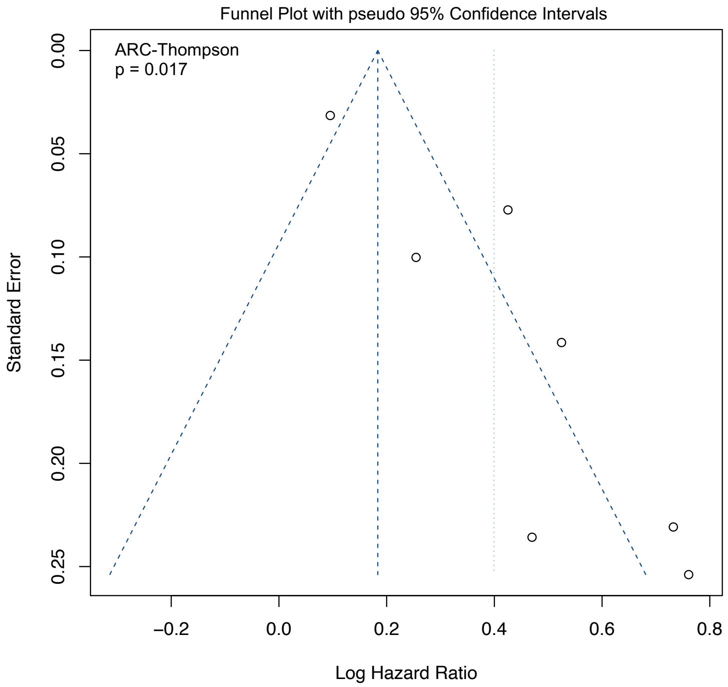

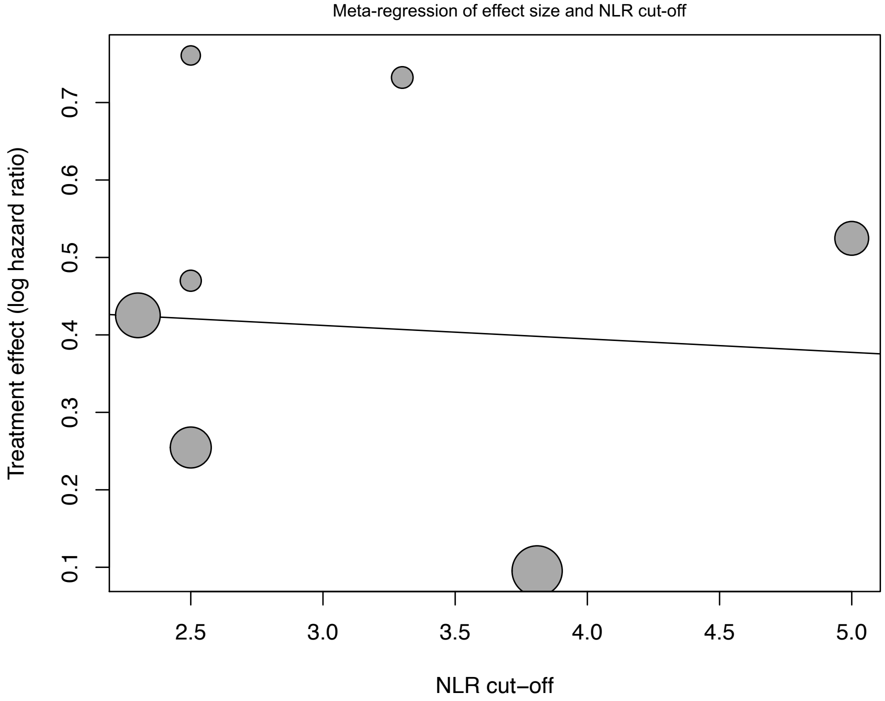

Supplementary Figure 79 - IN: OS in neck and head cancers

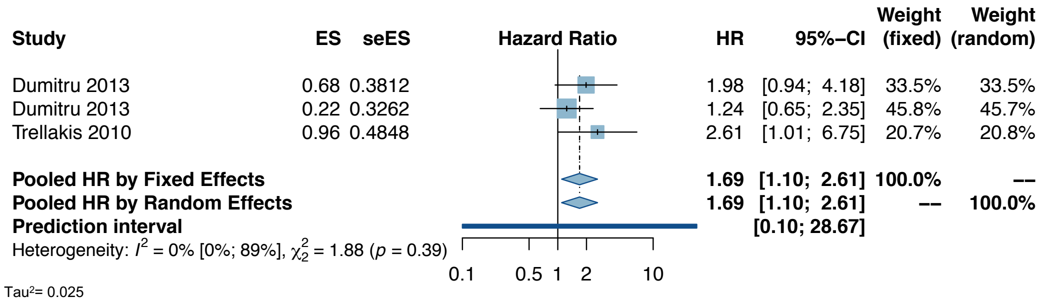

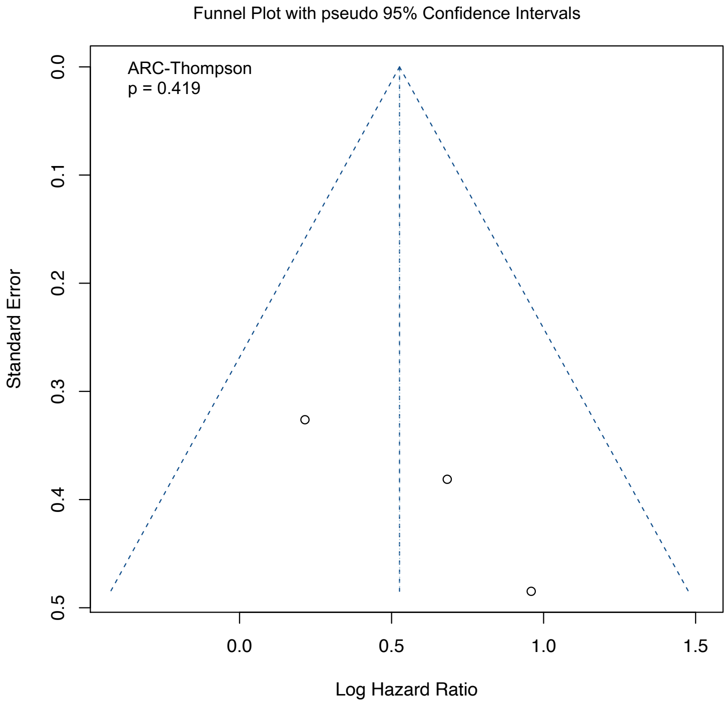

Supplementary Figure 80 - NLR: RFS in neck and head cancers

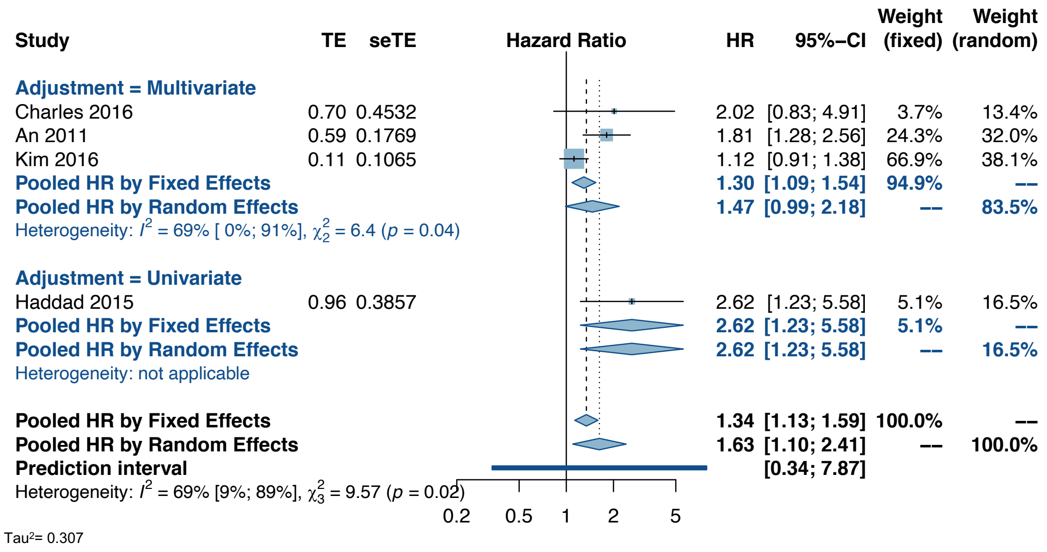

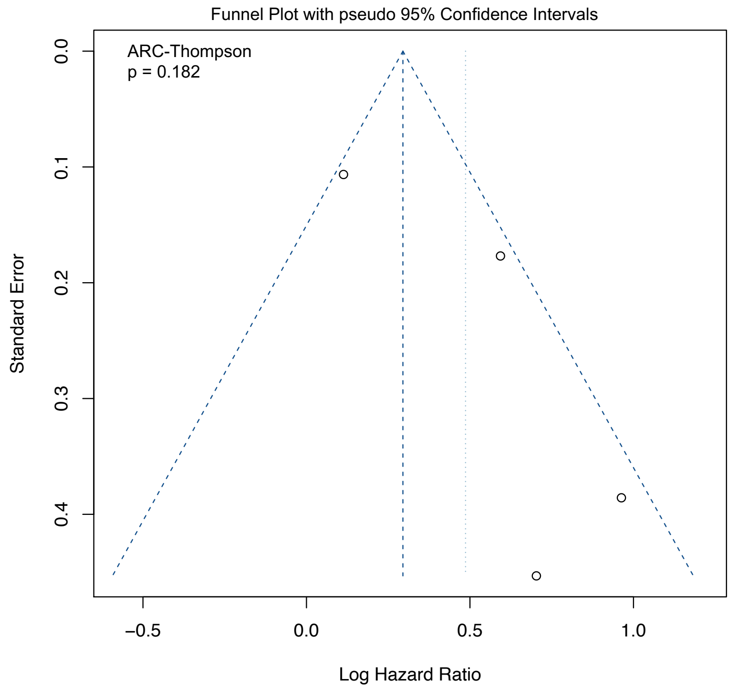

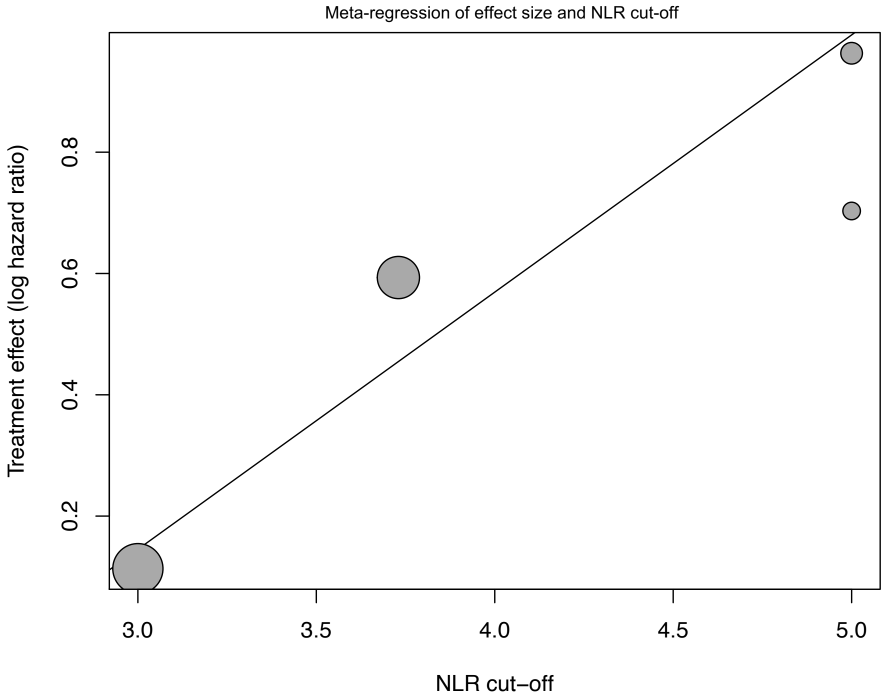

Supplementary Figure 81 - NLR: OS in larynx cancer

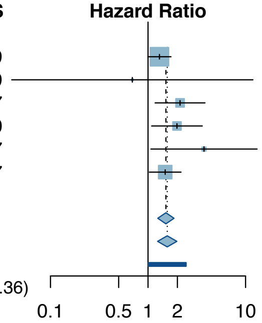

Study	ES	seES	HR	95%−CI	Weight (fixed)	Weight (random)
Fu 2016	0.27	0.1389	1.31	[1.00; 1.72]	49.8%	42.6%
Kara 2017	−0.37	1.4529	0.69	[0.04; 11.90]	0.5%	0.6%
Tu 2015	0.76	0.3007	2.13	[1.18; 3.84]	10.6%	13.1%
Wang 2016	0.68	0.3050	1.98	[1.09; 3.60]	10.3%	12.8%
Wong 2015	1.32	0.6397	3.75	[1.07; 13.14]	2.3%	3.2%
Zeng 2016	0.41	0.1907	1.50	[1.03; 2.18]	26.4%	27.7%
**Pooled HR by Fixed Effects**			**1.52**	**[1.26; 1.85]**	**100.0%**	**--**
**Pooled HR by Random Effects**			**1.57**	**[1.25; 1.98]**	**--**	**100.0%**
**Prediction interval**				**[1.01; 2.46]**		

Heterogeneity: $I^2 = 8\%$ [0%; 77%], $\chi^2_5 = 5.45$ ($p = 0.36$)

Tau²= 0.111

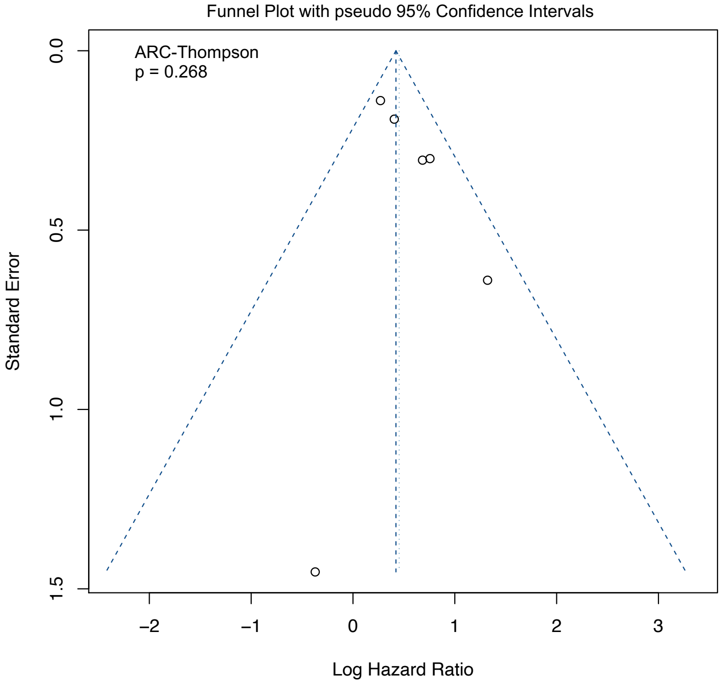

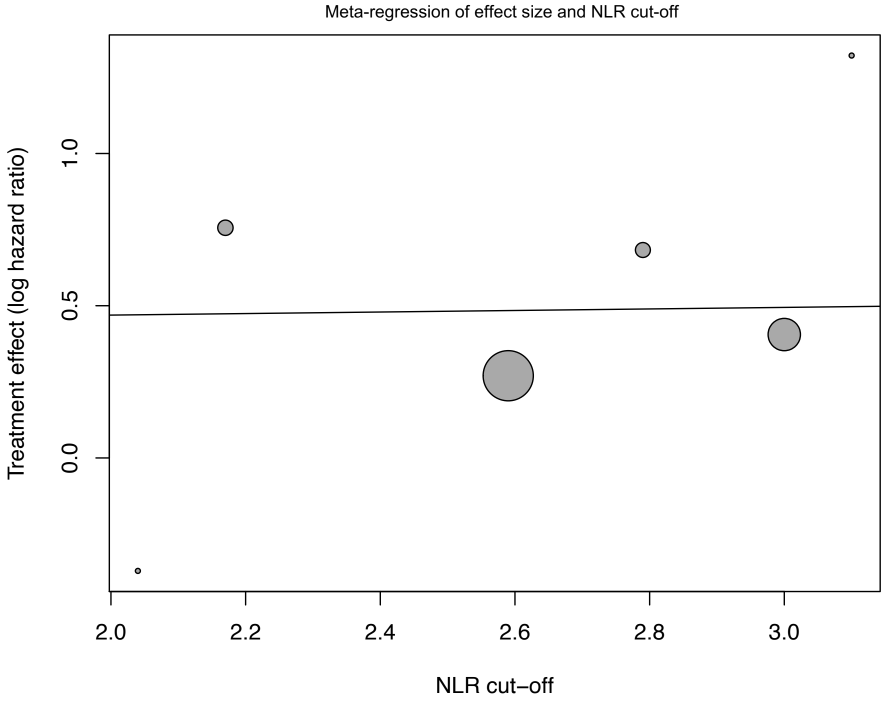

Supplementary *Figure 82* - NLR: PFS in nasopharyngeal cancer

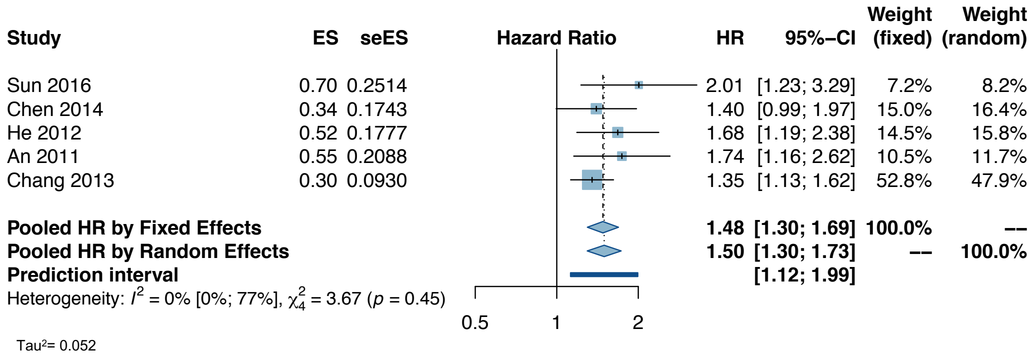

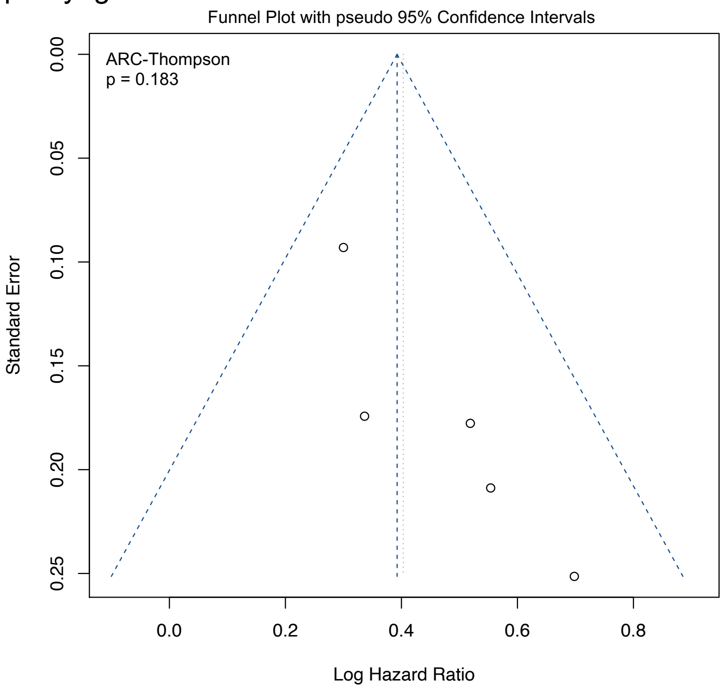

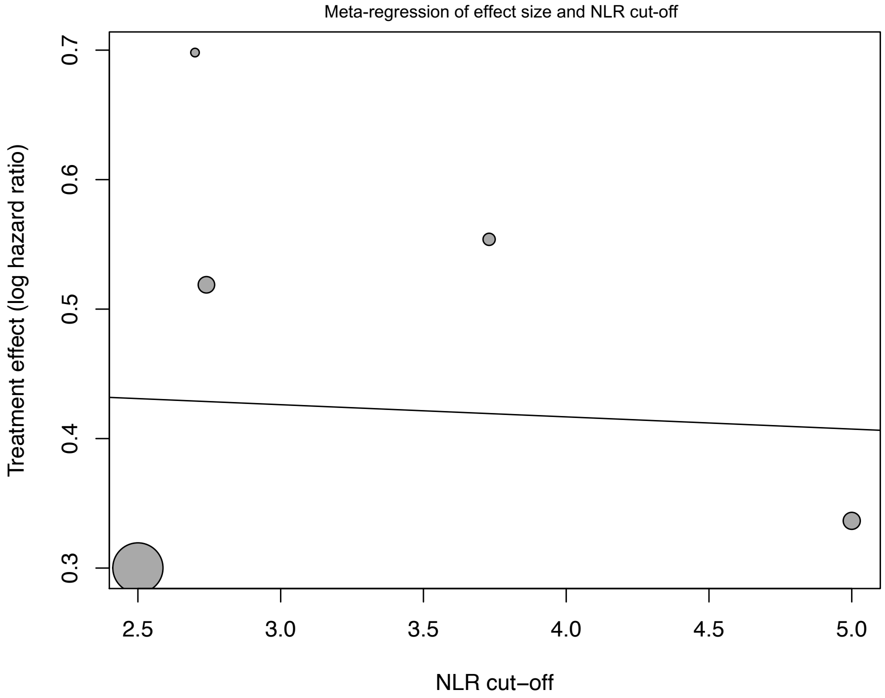

Supplementary *Figure 83* - NLR: CSS in nasopharyngeal cancer

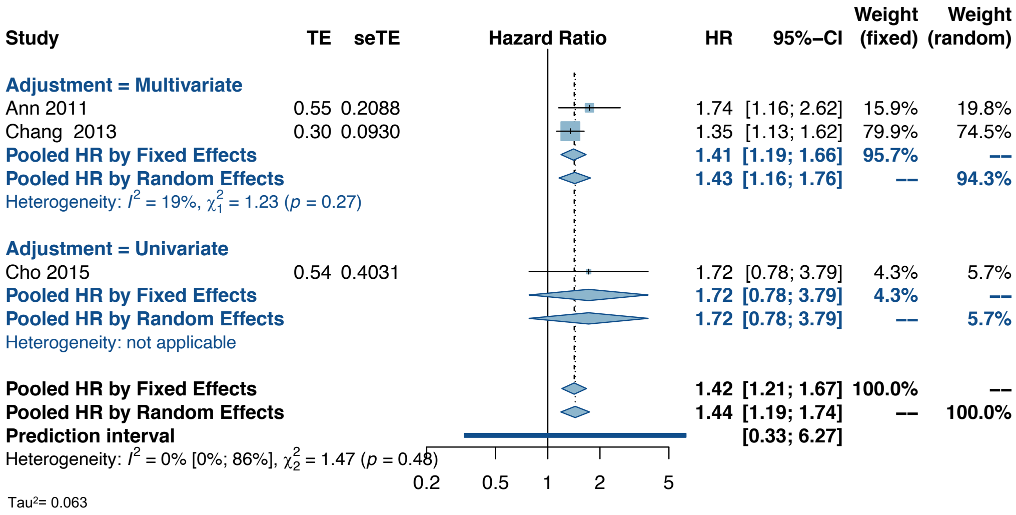

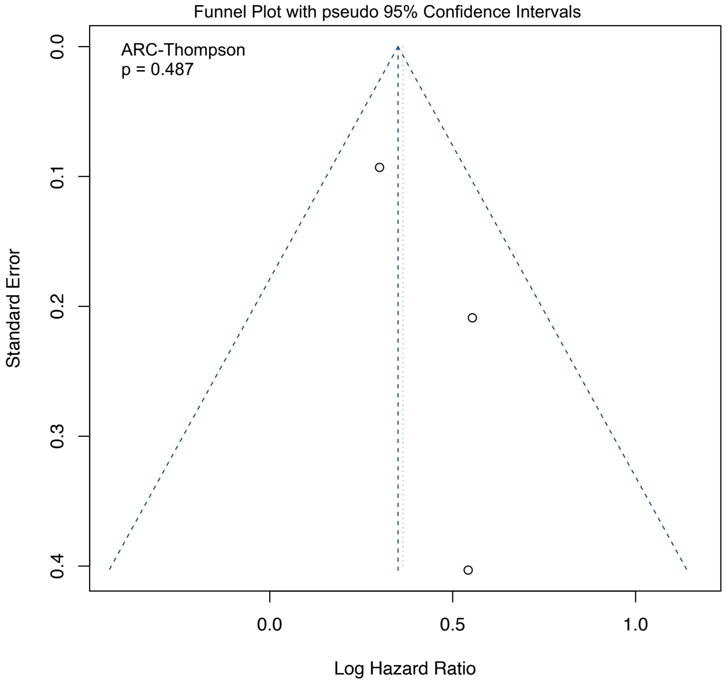

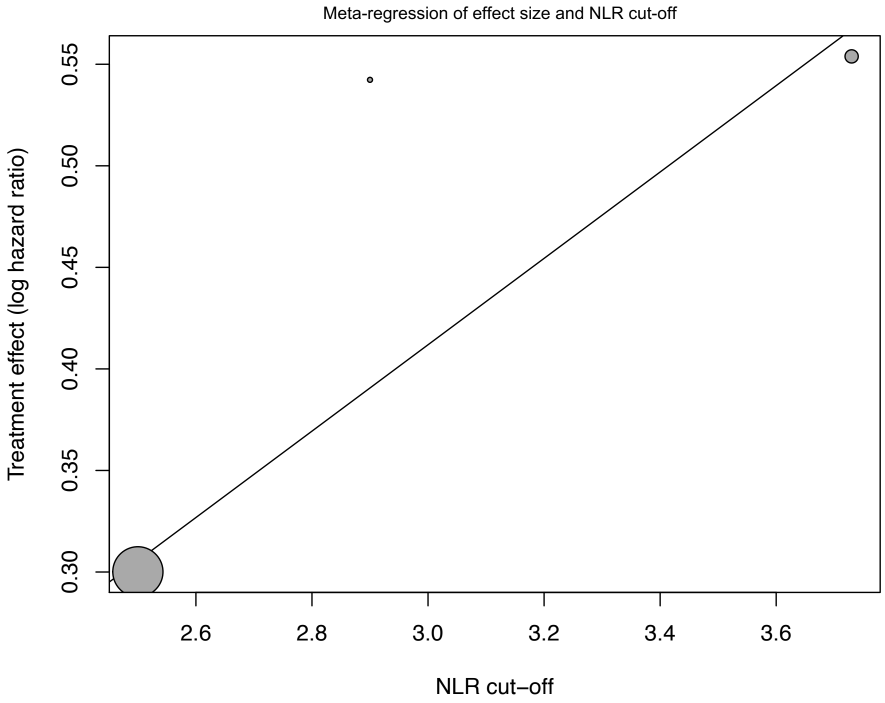

Supplementary *Figure 84* - NLR: OS in oral squamous cell carcinoma

Study	TE	seTE	HR	95%–CI	Weight (fixed)	Weight (random)
**Adjustment = Multivariate**						
Nakashima 2016	0.67	0.4495	1.96	[0.81; 4.73]	5.2%	5.2%
Ong 2016	0.46	0.2795	1.59	[0.92; 2.75]	13.3%	13.4%
Bobdey 2017	0.33	0.1486	1.39	[1.04; 1.86]	47.2%	46.9%
Wu 2017	0.83	0.2794	2.29	[1.32; 3.96]	13.4%	13.4%
**Pooled HR by Fixed Effects**			**1.58**	**[1.26; 1.98]**	**79.1%**	**--**
**Pooled HR by Random Effects**			**1.62**	**[1.25; 2.10]**	**--**	**78.9%**
Heterogeneity: $I^2 = 0\%$ [0%; 83%], $\chi^2_3 = 2.74$ (p = 0.43)						
**Adjustment = Univariate**						
Fang 2013	0.71	0.3448	2.04	[1.04; 4.01]	8.8%	8.8%
Christina 2016	0.15	0.2930	1.16	[0.65; 2.06]	12.1%	12.2%
**Pooled HR by Fixed Effects**			**1.47**	**[0.95; 2.28]**	**20.9%**	**--**
**Pooled HR by Random Effects**			**1.49**	**[0.86; 2.59]**	**--**	**21.1%**
Heterogeneity: $I^2 = 36\%$, $\chi^2_1 = 1.56$ (p = 0.21)						
**Pooled HR by Fixed Effects**			**1.56**	**[1.28; 1.90]**	**100.0%**	**--**
**Pooled HR by Random Effects**			**1.56**	**[1.27; 1.91]**	**--**	**100.0%**
**Prediction interval**				**[1.17; 2.09]**		
Heterogeneity: $I^2 = 0\%$ [0%; 71%], $\chi^2_5 = 4.38$ (p = 0.50)						

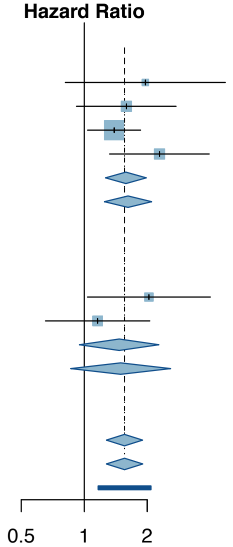

Tau²= 0.021

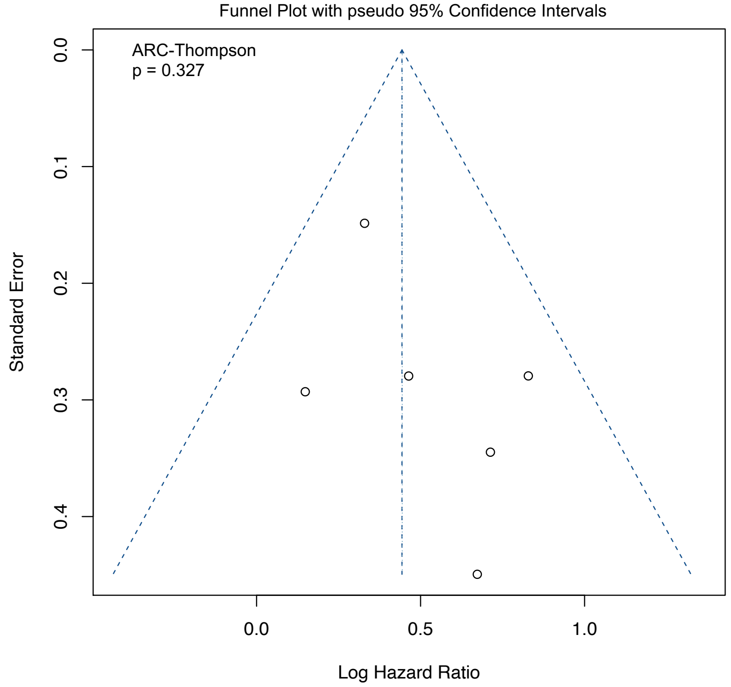

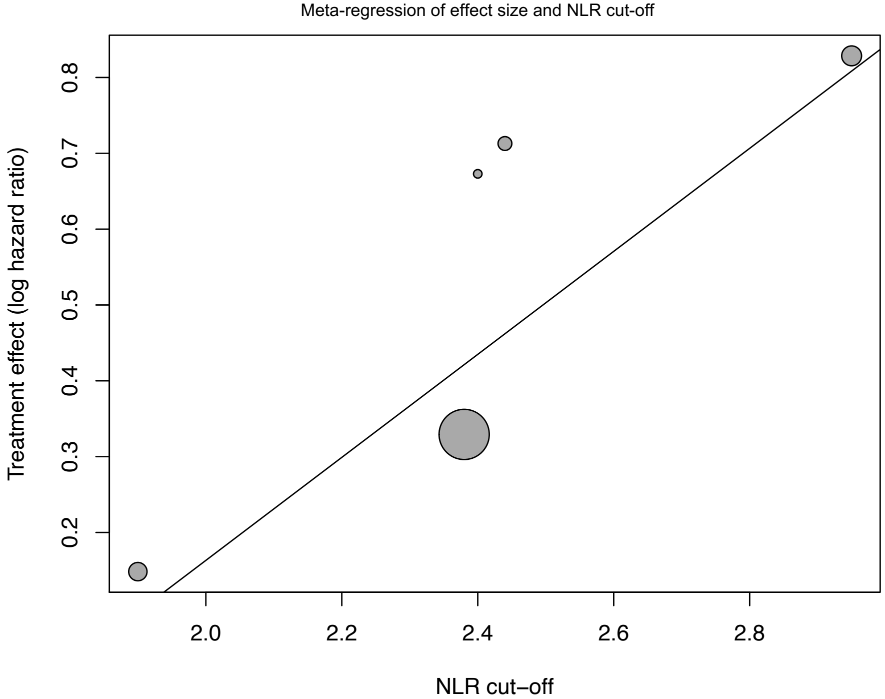

Supplementary *Figure 85* - NLR: DFS in oral squamous cell carcinoma

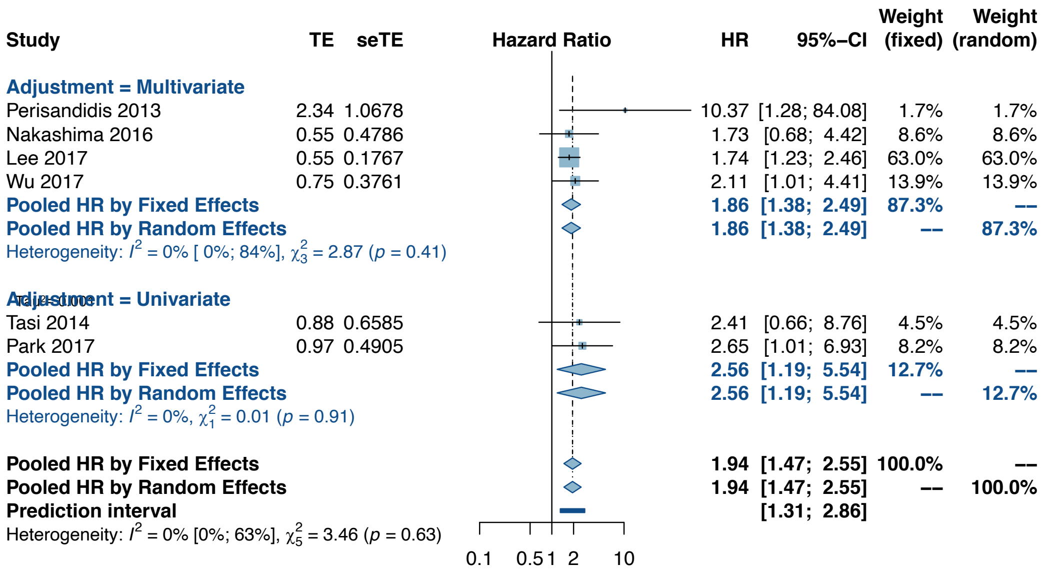

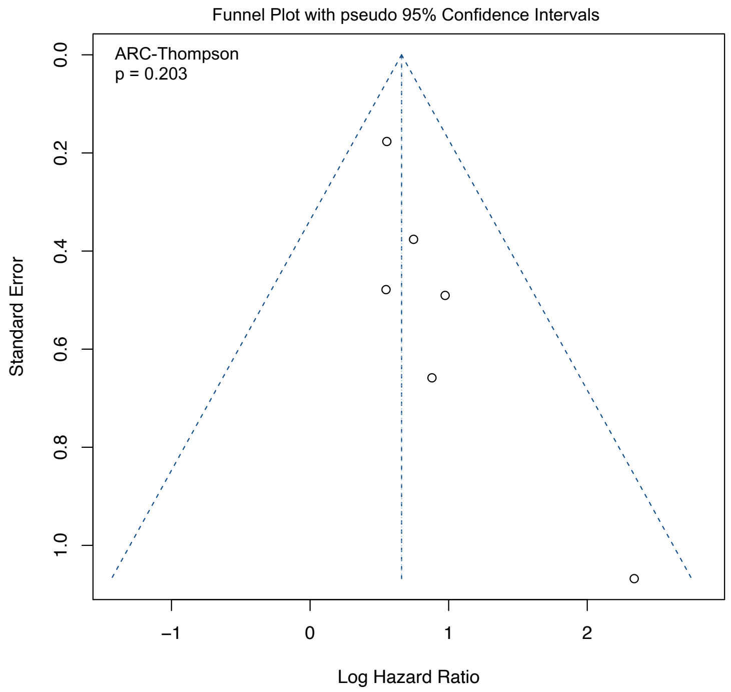

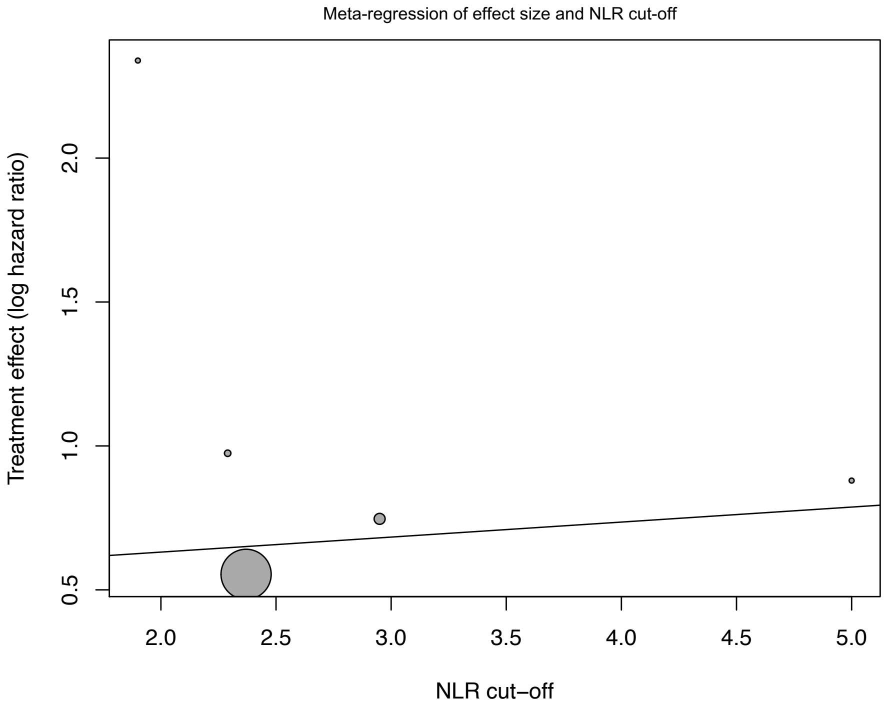

Supplementary *Figure 86* - NLR: OS in malignant pleural mesothelioma

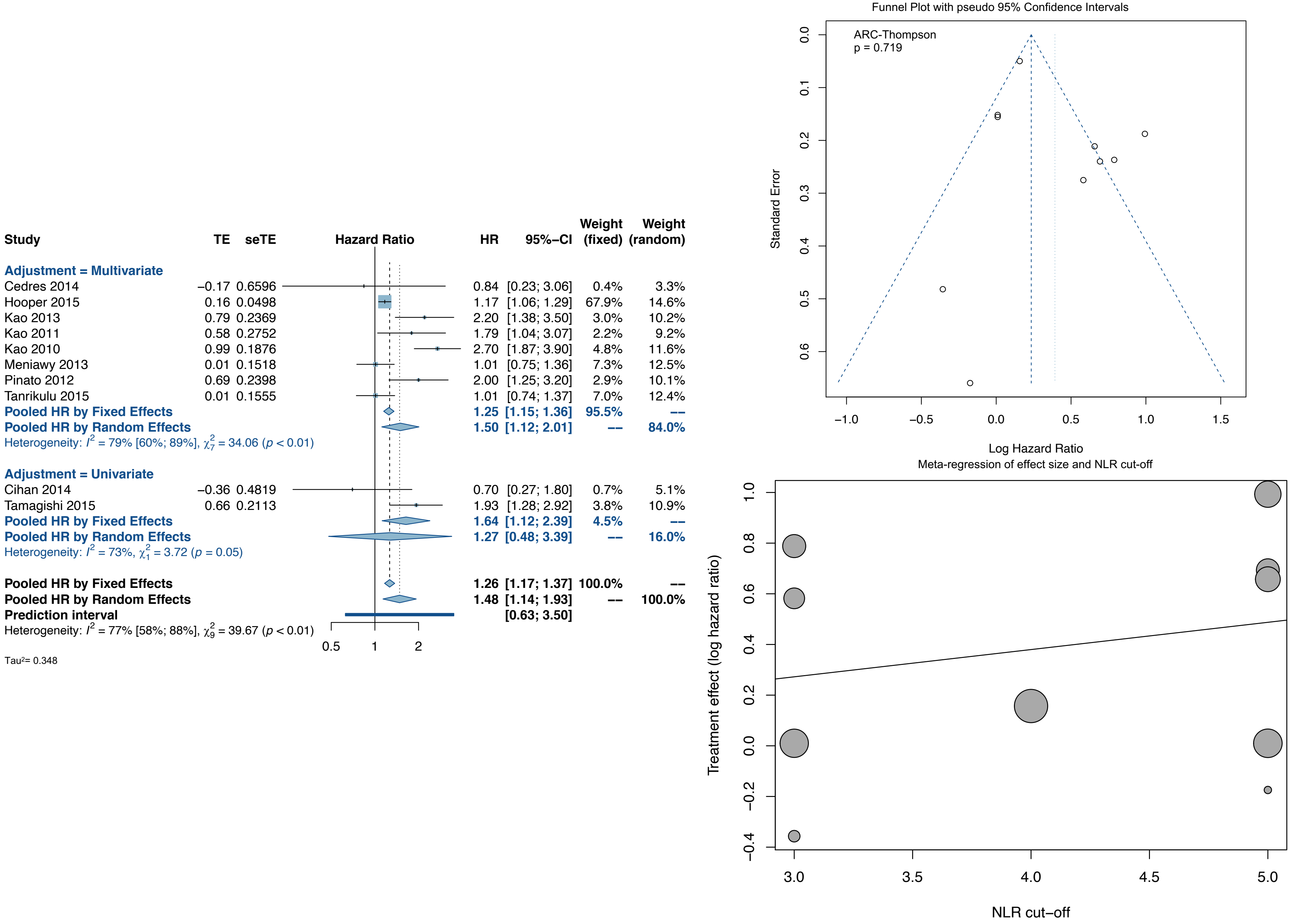

Supplementary *Figure 87* - NLR: OS in melanoma

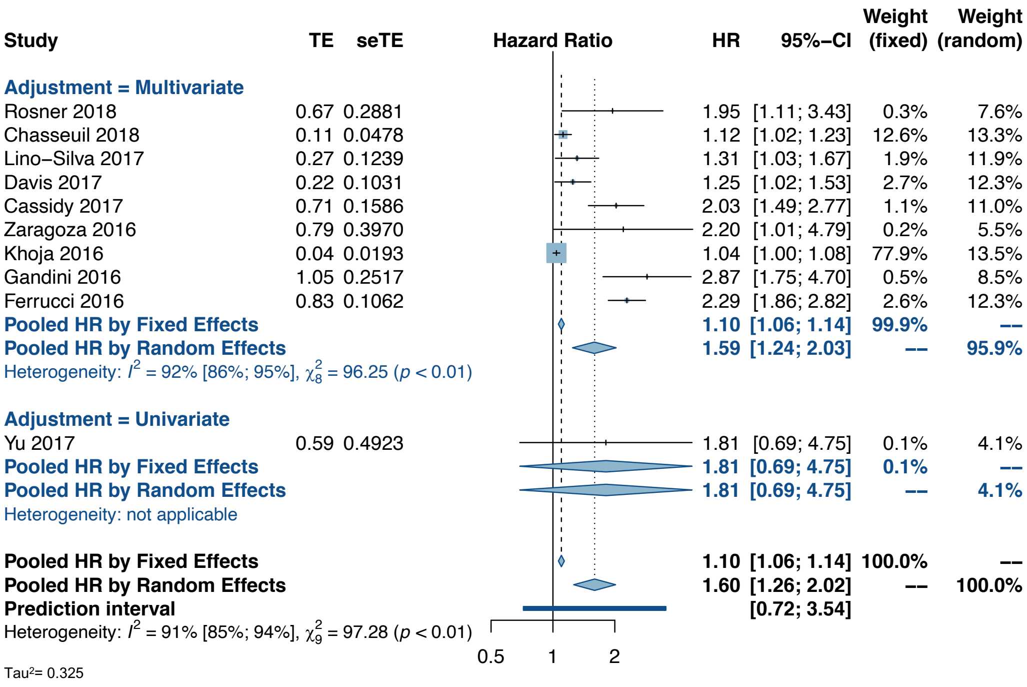

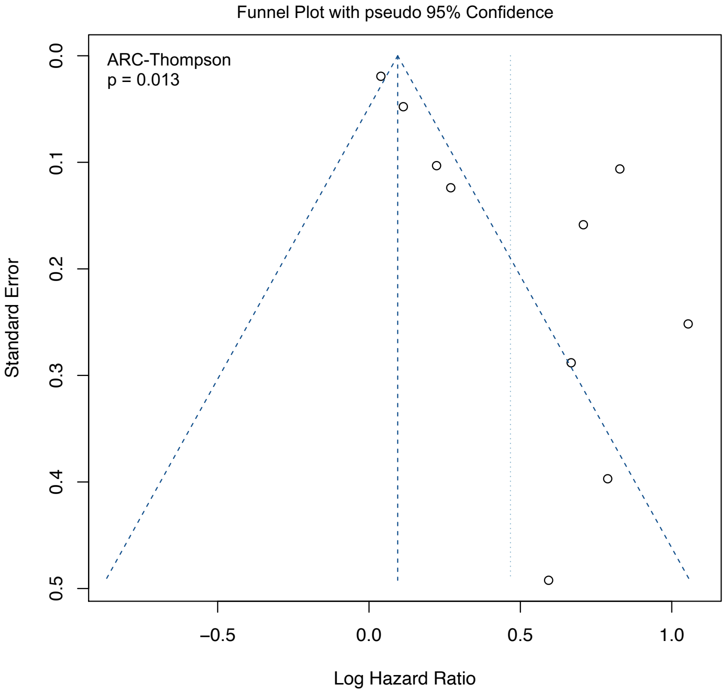

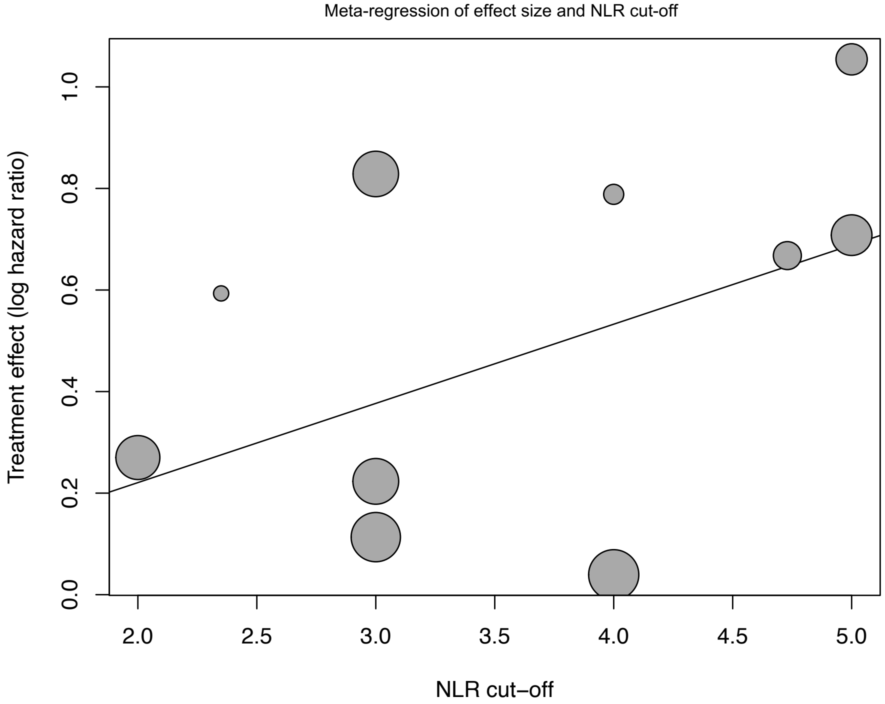

Supplementary *Figure 88* - NLR: PFS in melanoma

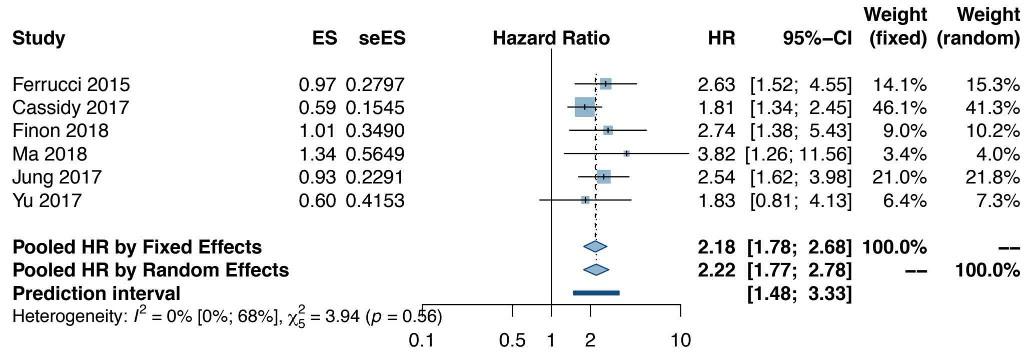

Study	ES	seES	HR	95%–CI	Weight (fixed)	Weight (random)
Ferrucci 2015	0.97	0.2797	2.63	[1.52; 4.55]	14.1%	15.3%
Cassidy 2017	0.59	0.1545	1.81	[1.34; 2.45]	46.1%	41.3%
Finon 2018	1.01	0.3490	2.74	[1.38; 5.43]	9.0%	10.2%
Ma 2018	1.34	0.5649	3.82	[1.26; 11.56]	3.4%	4.0%
Jung 2017	0.93	0.2291	2.54	[1.62; 3.98]	21.0%	21.8%
Yu 2017	0.60	0.4153	1.83	[0.81; 4.13]	6.4%	7.3%
**Pooled HR by Fixed Effects**			**2.18**	**[1.78; 2.68]**	**100.0%**	**--**
**Pooled HR by Random Effects**			**2.22**	**[1.77; 2.78]**	**--**	**100.0%**
**Prediction interval**				**[1.48; 3.33]**		

Heterogeneity: $I^2 = 0\%$ [0%; 68%], $\chi^2_5 = 3.94$ ($p = 0.56$)

Tau²= 0.090

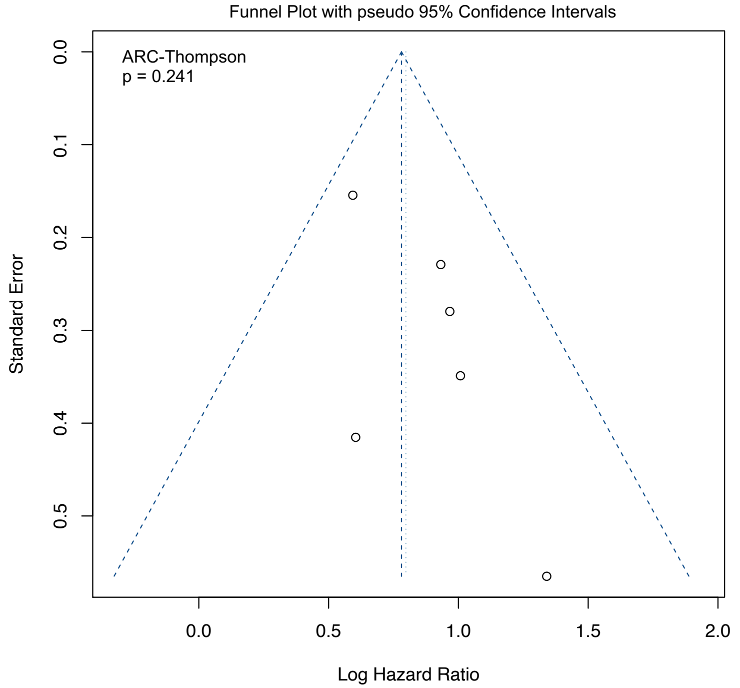

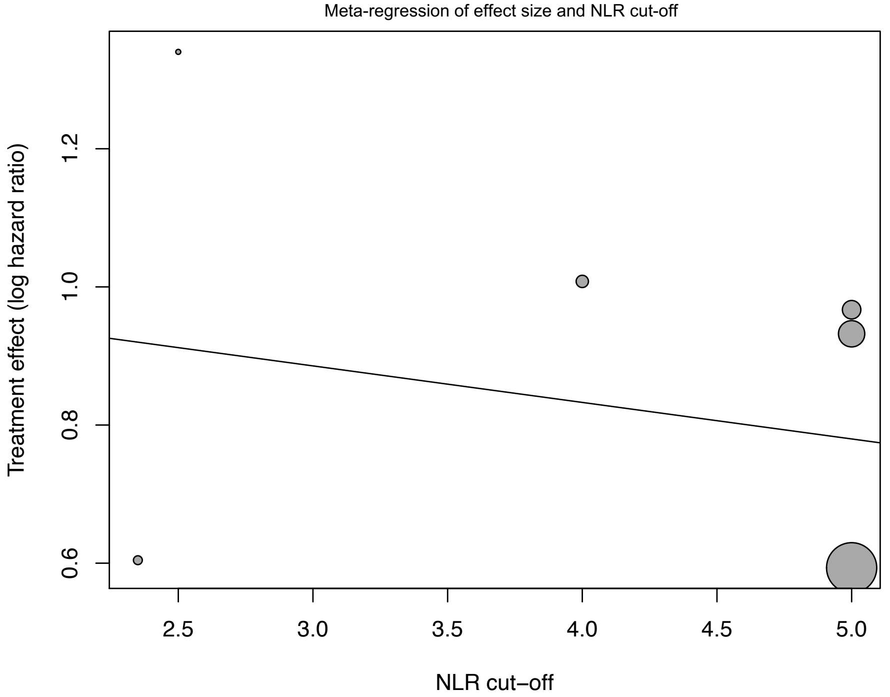

Supplementary Figure 89 – IN: OS in renal cancer

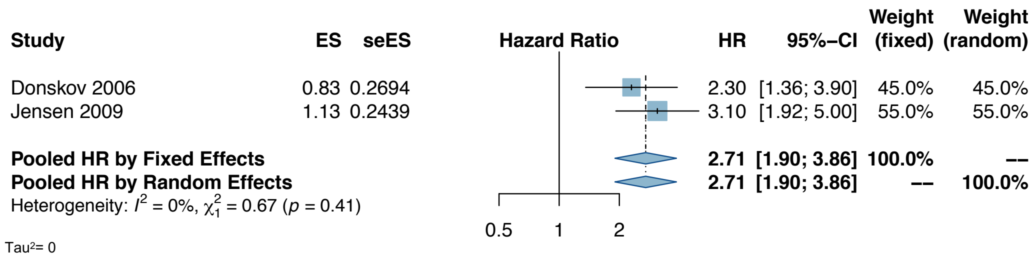

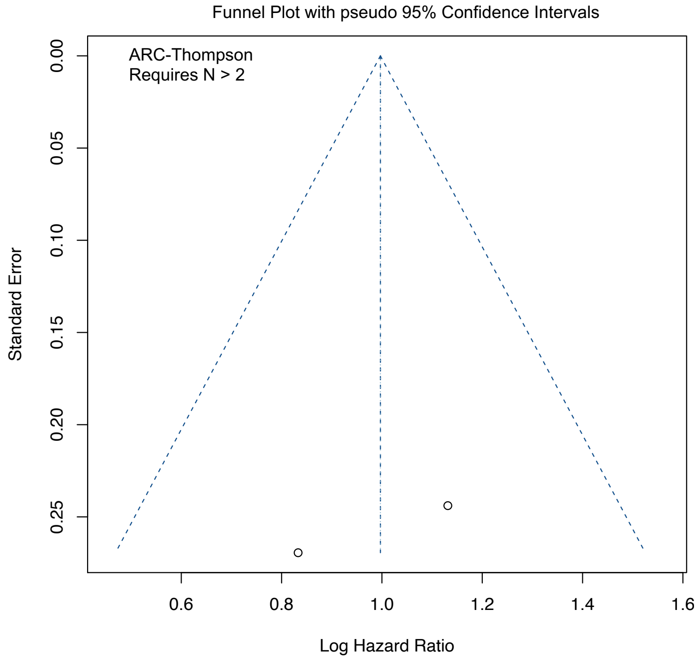

Supplementary Figure 90 - NLR: OS in renal cancer

Study	TE	seTE	HR	95%–CI	Weight (fixed)	Weight (random)
**Adjustment = Multivariate**						
Fox 2013	0.35	0.1322	1.42	[1.10; 1.84]	17.5%	14.0%
Hong 2013	0.93	0.3868	2.53	[1.19; 5.40]	2.0%	3.9%
Cetin 2013	0.88	0.3025	2.41	[1.33; 4.36]	3.3%	5.8%
Konayashi 2013	0.96	0.4828	2.62	[1.02; 6.75]	1.3%	2.7%
Forget 2013	0.52	0.2624	1.68	[1.00; 2.81]	4.4%	7.0%
Pichler 2013	0.46	0.1906	1.59	[1.09; 2.31]	8.4%	10.3%
Dirican 2013	0.79	0.3957	2.21	[1.02; 4.80]	1.9%	3.8%
Grivas 2014	1.05	0.4962	2.87	[1.09; 7.59]	1.2%	2.6%
Keizman 2014	1.08	0.1970	2.95	[2.01; 4.34]	7.9%	9.9%
Templeton 2014	0.41	0.1252	1.51	[1.18; 1.93]	19.5%	14.5%
Ohno 2014	1.21	0.3925	3.35	[1.55; 7.23]	2.0%	3.8%
Santoni 2015	0.79	0.3078	2.21	[1.21; 4.04]	3.2%	5.6%
**Pooled HR by Fixed Effects**			**1.80**	**[1.59; 2.04]**	**72.7%**	**--**
**Pooled HR by Random Effects**			**1.98**	**[1.63; 2.41]**	**--**	**84.0%**
Heterogeneity: I^2 = 40% [0%; 70%], χ^2_{11} = 18.38 (p = 0.07)						
**Adjustment = Univariate**						
Viers 2014	0.58	0.1058	1.78	[1.45; 2.19]	27.3%	16.0%
**Pooled HR by Fixed Effects**			**1.78**	**[1.45; 2.19]**	**27.3%**	**--**
**Pooled HR by Random Effects**			**1.78**	**[1.45; 2.19]**	**--**	**16.0%**
Heterogeneity: not applicable						
**Pooled HR by Fixed Effects**			**1.79**	**[1.61; 2.00]**	**100.0%**	**--**
**Pooled HR by Random Effects**			**1.92**	**[1.63; 2.27]**	**--**	**100.0%**
**Prediction interval**				**[1.23; 3.01]**		
Heterogeneity: I^2 = 35% [0%; 66%], χ^2_{12} = 18.39 (p = 0.10)						

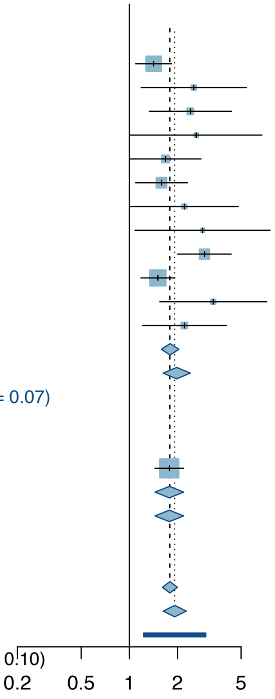

Tau²= 0.185

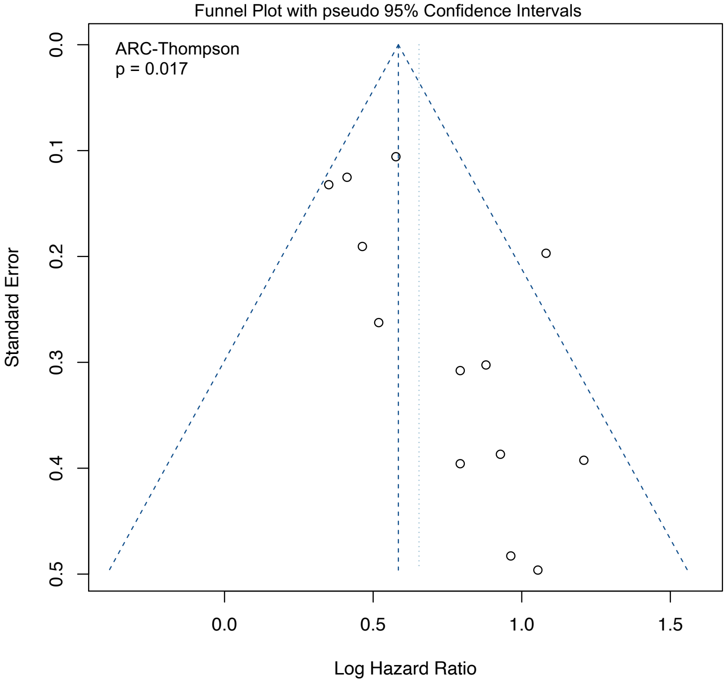

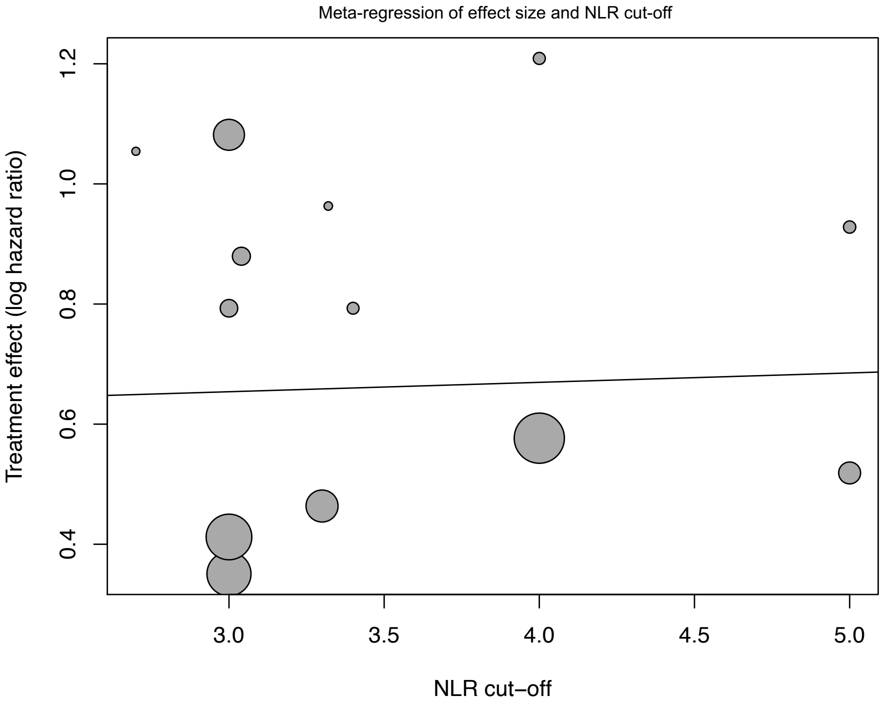

Supplementary Figure 91 - NLR: OS in advanced renal cancer

Study	ES	seES	HR	95%−CI	Weight (fixed)	Weight (random)
Cetin 2013	0.88	0.3025	2.41	[1.33; 4.36]	4.6%	11.5%
Fox 2013	0.35	0.1294	1.42	[1.10; 1.83]	25.0%	19.5%
Hatakeyama 2013	0.22	0.0964	1.25	[1.03; 1.51]	45.1%	21.0%
Keizman 2014	1.08	0.2783	2.95	[1.71; 5.09]	5.4%	12.4%
Koo 2016	0.65	0.1747	1.91	[1.36; 2.69]	13.7%	17.3%
Ohno 2014	1.23	0.4054	3.42	[1.55; 7.57]	2.6%	8.2%
Santoni 2013	0.82	0.3423	2.27	[1.16; 4.44]	3.6%	10.0%
**Pooled HR by Fixed Effects**			**1.55**	**[1.36; 1.76]**	**100.0%**	**--**
**Pooled HR by Random Effects**			**1.91**	**[1.44; 2.53]**	**--**	**100.0%**
**Prediction interval**				**[0.81; 4.47]**		

Hazard Ratio axis: 0.2, 0.5, 1, 2, 5

Heterogeneity: $I^2 = 69\%$ [32%; 86%], $\chi^2_6 = 19.39$ ($p < 0.01$)

Tau²= 0.299

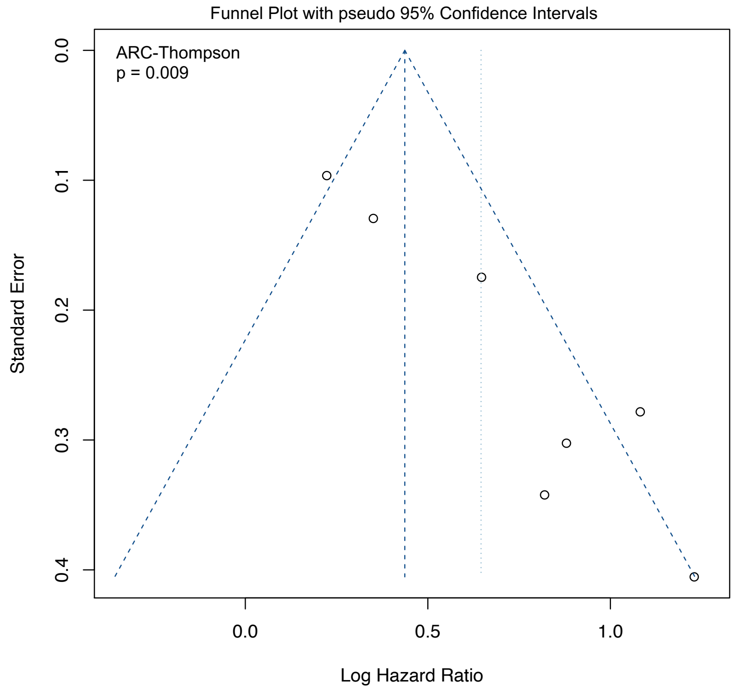

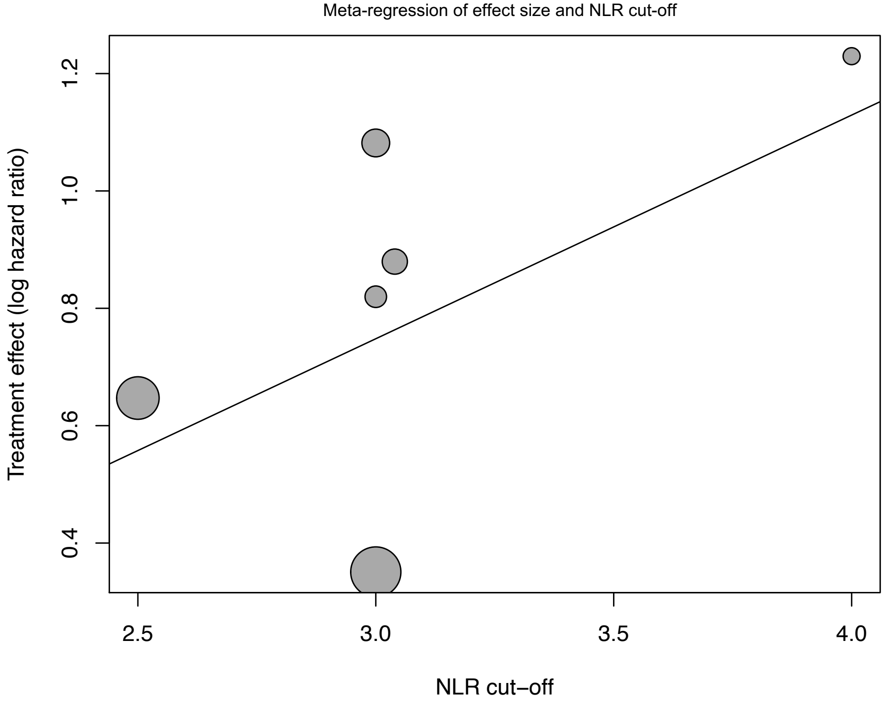

Supplementary Figure 92 - NLR: OS in localised renal cancer

Study	ES	seES	HR	95%-CI	Weight (fixed)	Weight (random)
Forget 2013	0.51	0.2618	1.67	[1.00; 2.79]	0.0%	33.4%
Grivas 2014	1.05	0.4989	2.87	[1.08; 7.63]	0.0%	18.6%
Viers 2014	0.02	0.0050	1.02	[1.01; 1.03]	100.0%	47.9%
**Pooled HR by Fixed Effects**			**1.02**	**[1.01; 1.03]**	**100.0%**	**--**
**Pooled HR by Random Effects**			**1.46**	**[0.85; 2.50]**	**--**	**100.0%**
**Prediction interval**				**[0.00; 683.18]**		

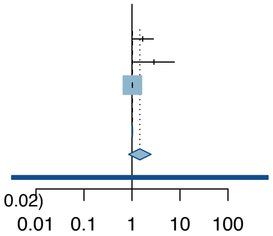

Heterogeneity: $I^2 = 74\%$ [15%; 92%], $\chi^2_2 = 7.84$ ($p = 0.02$)

Tau²= 0.398

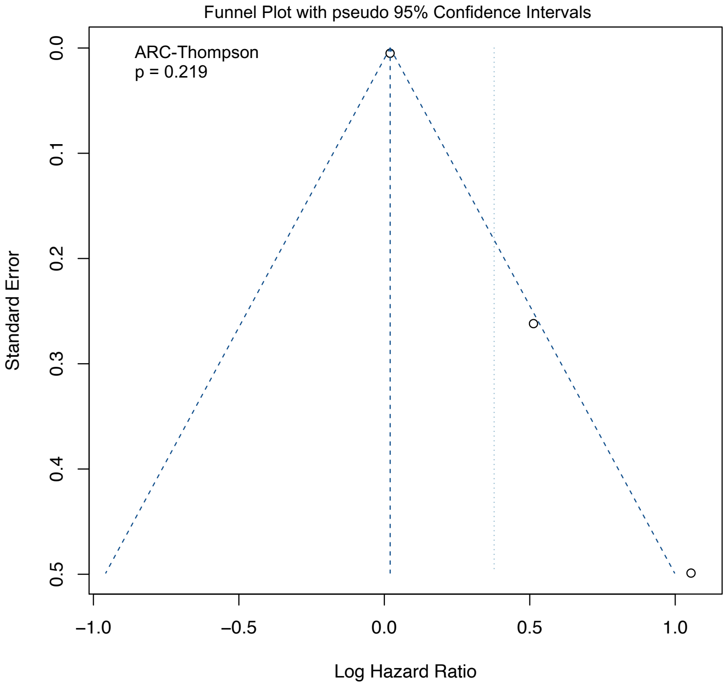

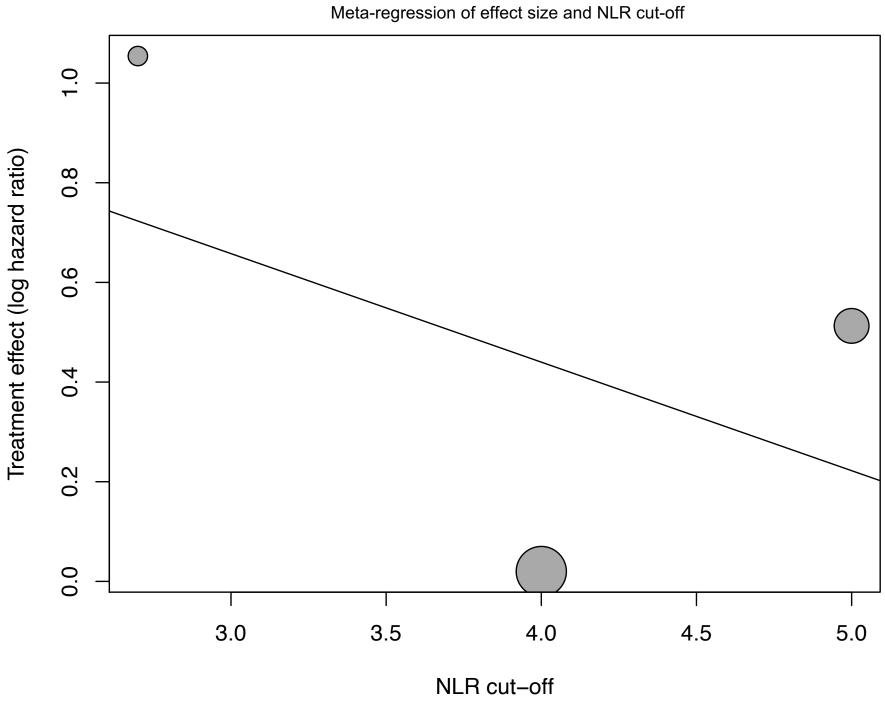

Supplementary Figure 93 - NLR: OS in renal cancer with tyrosine-kinase inhibitors

Study	ES	seES	HR	95%–CI	Weight (fixed)	Weight (random)
Cetin 2013	0.88	0.3025	2.41	[1.33; 4.36]	8.5%	11.9%
Dirican 2013	0.84	0.3878	2.31	[1.08; 4.94]	5.2%	8.4%
Kobayashi 2013	0.96	0.4828	2.62	[1.02; 6.75]	3.4%	5.9%
Kelzman 2014	1.08	0.1981	2.95	[2.00; 4.35]	19.9%	19.0%
Zhang 2015	0.33	0.1568	1.39	[1.02; 1.89]	31.8%	22.9%
Santoni 2015	0.79	0.3078	2.21	[1.21; 4.04]	8.2%	11.6%
Chrom 2016	0.77	0.1843	2.16	[1.51; 3.10]	23.0%	20.2%
**Pooled HR by Fixed Effects**			**2.04**	**[1.72; 2.43]**	**100.0%**	**--**
**Pooled HR by Random Effects**			**2.14**	**[1.66; 2.76]**	**--**	**100.0%**
**Prediction interval**				**[1.11; 4.12]**		

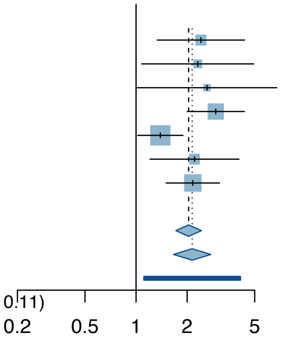

Heterogeneity: $I^2 = 42\%$ [0%; 75%], $\chi^2_6 = 10.29$ ($p = 0.11$)

Tau²= 0.220

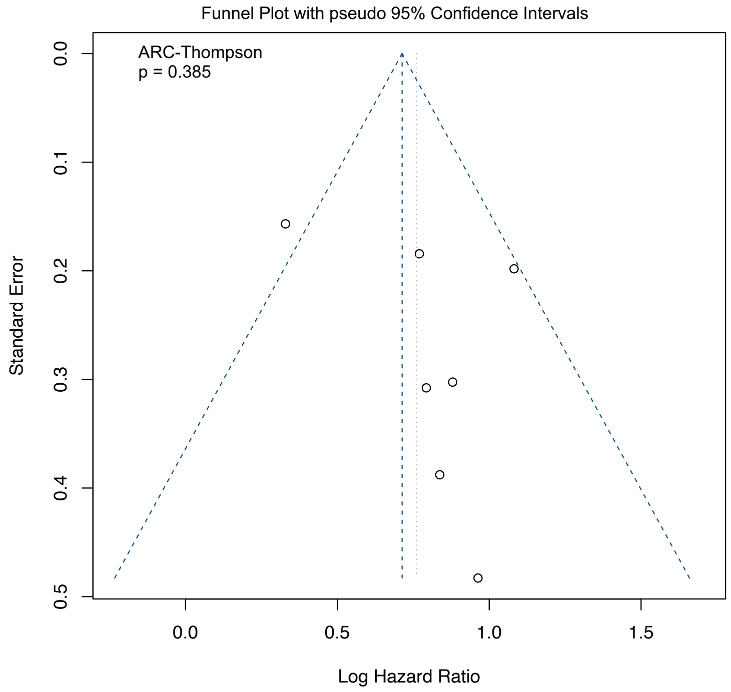

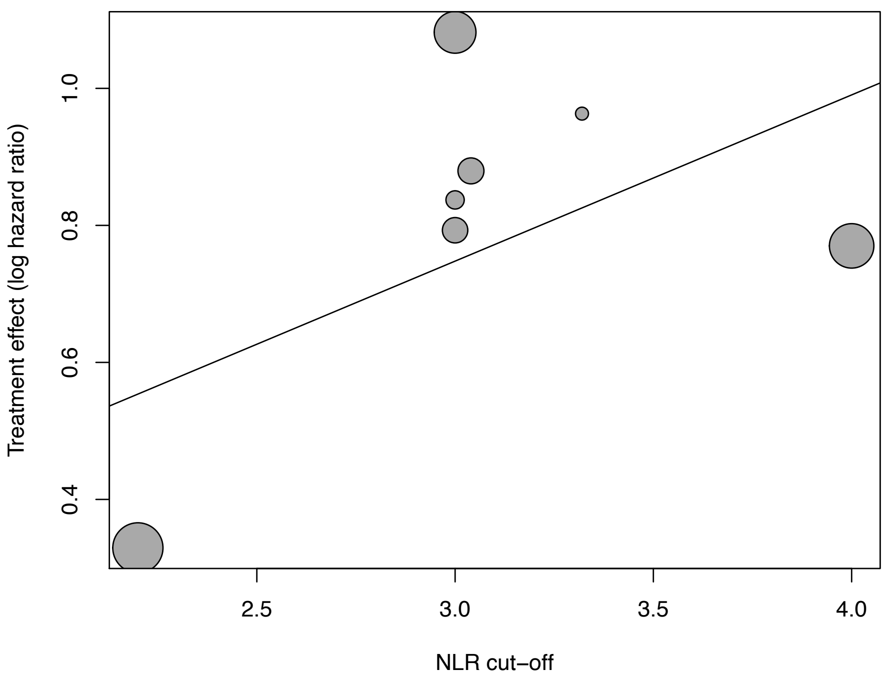

Supplementary Figure 94 - NLR: RFS in renal cancer

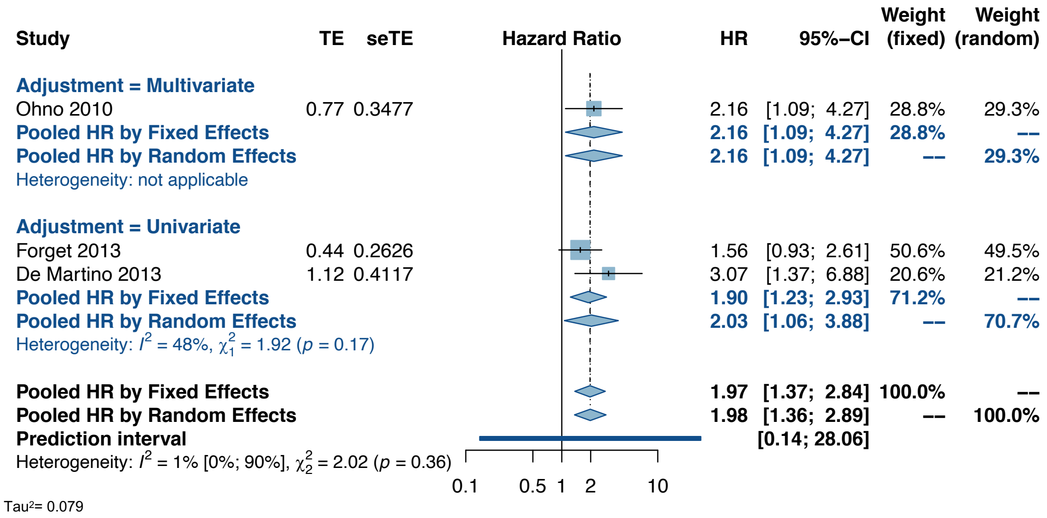

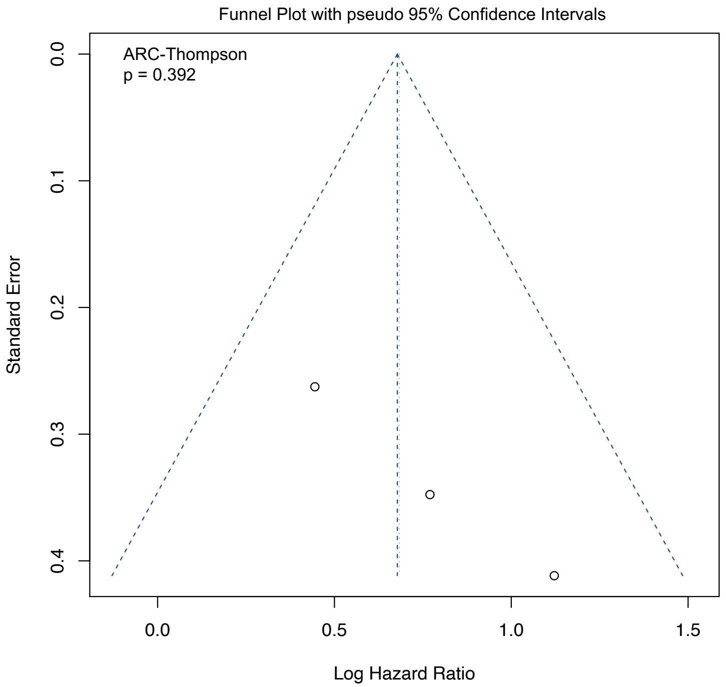

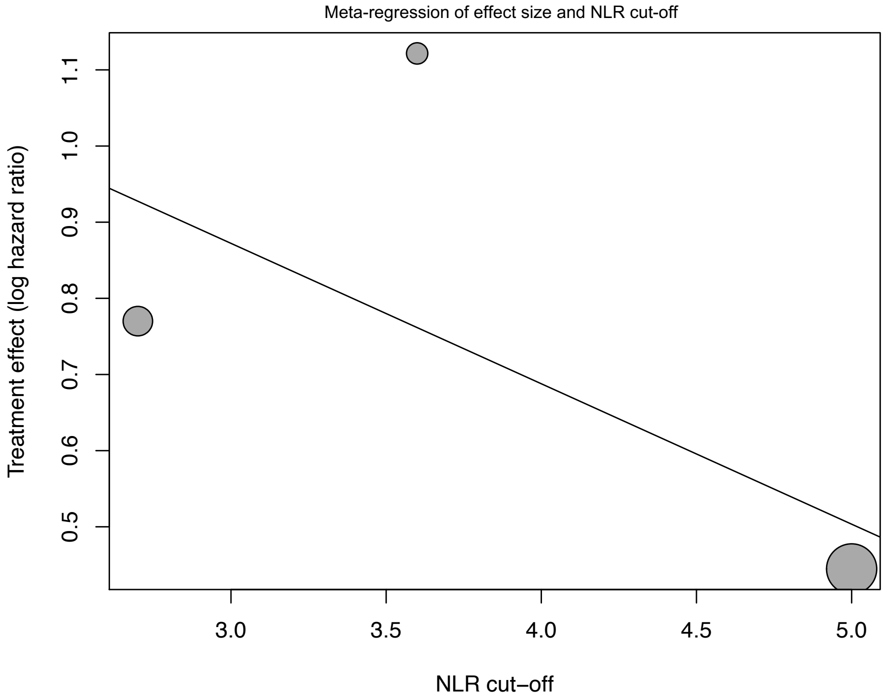

Supplementary Figure 95 - NLR: RFS in localised renal cancer

Study	ES	seES	HR	95%–CI	Weight (fixed)	Weight (random)
Martino 2013	1.12	0.4117	3.07	[1.37; 6.88]	0.0%	11.5%
Forget 2013	0.44	0.2587	1.56	[0.94; 2.59]	0.0%	16.6%
Grivas 2014	0.21	0.1310	1.23	[0.95; 1.59]	0.1%	21.2%
Ohno 2010	0.77	0.3441	2.16	[1.10; 4.24]	0.0%	13.6%
Ohno 2012	1.14	0.3379	3.12	[1.61; 6.05]	0.0%	13.8%
Viers 2014	0.01	0.0050	1.01	[1.00; 1.02]	99.8%	23.4%
**Pooled HR by Fixed Effects**			**1.01**	**[1.00; 1.02]**	**100.0%**	**--**
**Pooled HR by Random Effects**			**1.67**	**[1.13; 2.45]**	**--**	**100.0%**
**Prediction interval**				**[0.48; 5.82]**		

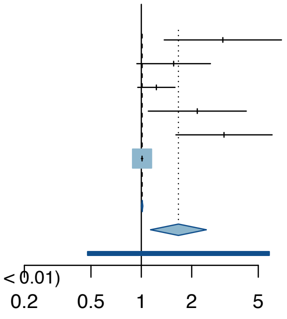

Heterogeneity: $I^2 = 82\%$ [63%; 92%], $\chi^2_5 = 28.36$ ($p < 0.01$)

Tau²= 0.405

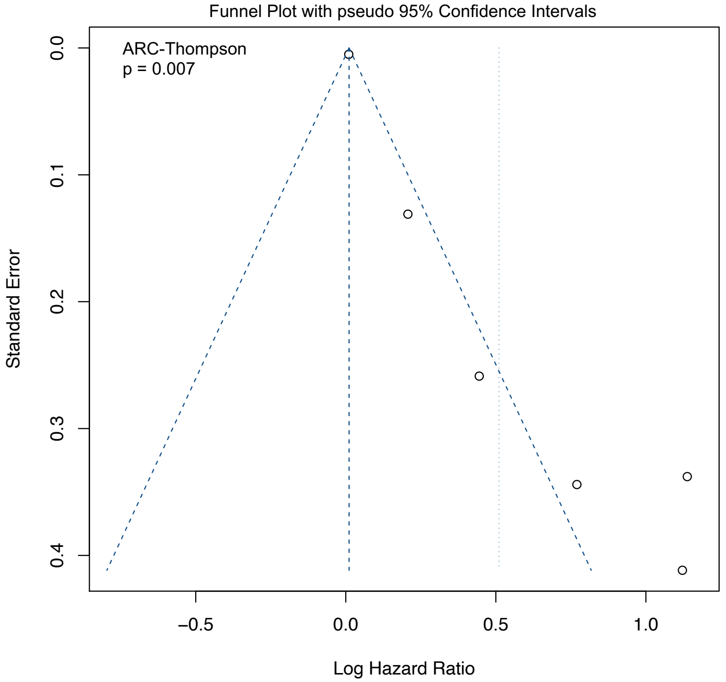

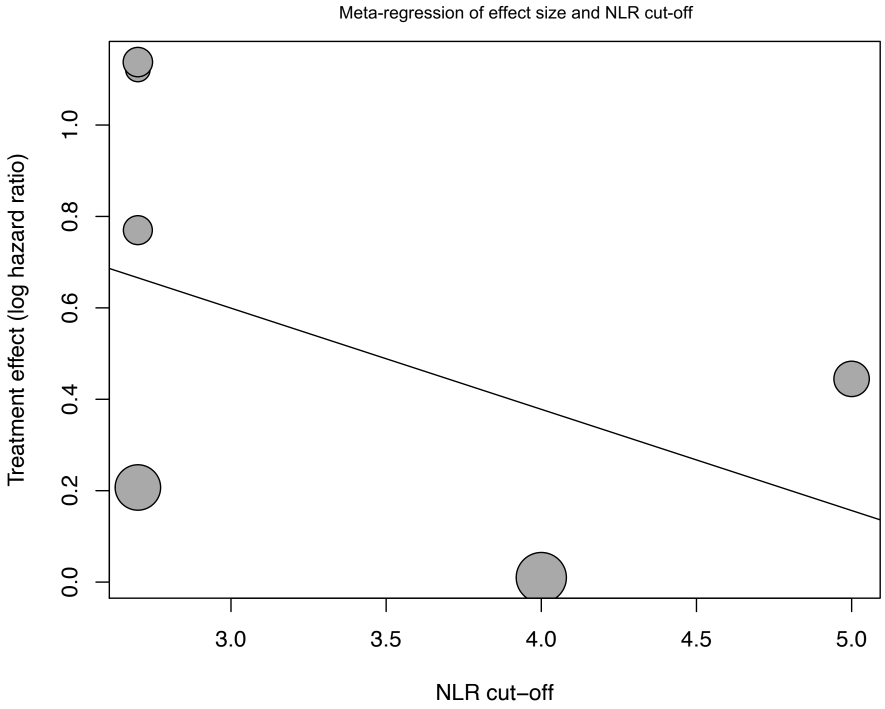

Supplementary Figure 96 - NLR: PFS in renal cancer

Study	ES	seES	HR	95%−CI	Weight (fixed)	Weight (random)
Kobayashi 2013	1.21	0.5135	3.37	[1.23; 9.22]	0.4%	7.2%
Cetin 2013	0.10	0.0355	1.11	[1.04; 1.19]	87.3%	19.1%
Dirican 2013	0.72	0.3586	2.06	[1.02; 4.16]	0.9%	10.6%
Santoni 2014	0.79	0.3078	2.21	[1.21; 4.04]	1.2%	12.1%
Gunduz 2014	1.52	0.7034	4.56	[1.15; 18.10]	0.2%	4.7%
Dana 2014	0.78	0.2536	2.19	[1.33; 3.60]	1.7%	13.7%
Keizman 2014	1.26	0.1936	3.51	[2.40; 5.13]	2.9%	15.6%
Zhang 2015	0.43	0.1435	1.54	[1.16; 2.04]	5.4%	17.0%
**Pooled HR by Fixed Effects**			**1.21**	**[1.13; 1.29]**	**100.0%**	**--**
**Pooled HR by Random Effects**			**2.07**	**[1.47; 2.92]**	**--**	**100.0%**
**Prediction interval**				**[0.71; 6.00]**		

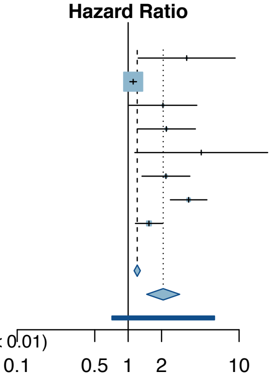

Heterogeneity: $I^2 = 88\%$ [78%; 93%], $\chi^2_7 = 58.02$ ($p < 0.01$)

Tau²= 0.398

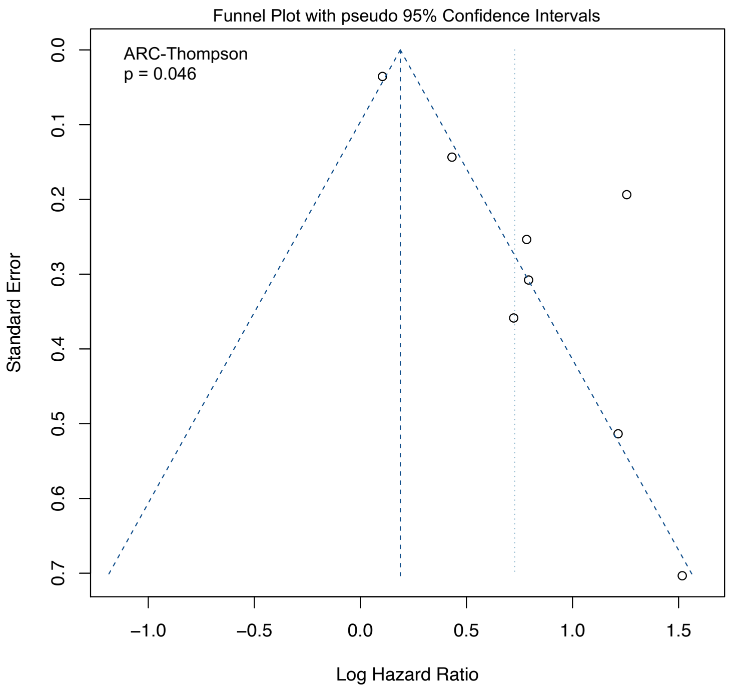

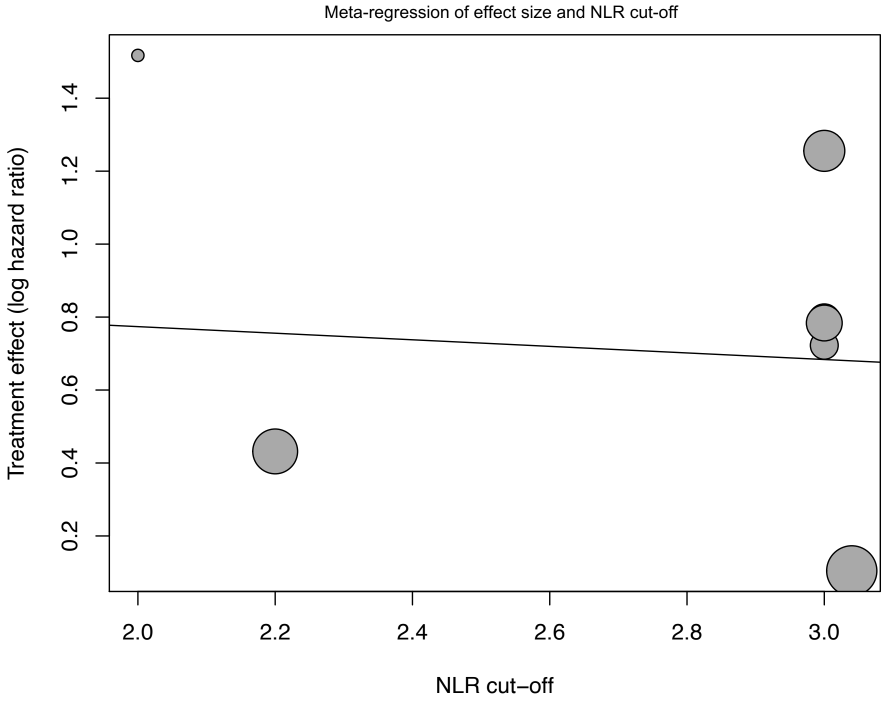

Supplementary Figure 97 - NLR: PFS in advanced renal cancer

Study	ES	seES	HR	95%–CI	Weight (fixed)	Weight (random)
Santoni 2013	0.98	0.3169	2.66	[1.43; 4.95]	33.4%	33.4%
Cetin 2013	1.25	0.3806	3.50	[1.66; 7.38]	23.1%	23.1%
Keizman 2014	1.25	0.2775	3.50	[2.03; 6.03]	43.5%	43.5%
**Pooled HR by Fixed Effects**			**3.19**	**[2.23; 4.57]**	**100.0%**	**--**
**Pooled HR by Random Effects**			**3.19**	**[2.23; 4.57]**	**--**	**100.0%**
**Prediction interval**				**[0.31; 32.69]**		

Heterogeneity: $I^2 = 0\%$ [0%; 58%], $\chi^2_2 = 0.50$ ($p = 0.78$)

Tau²= 0

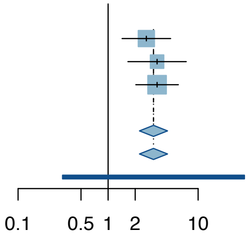

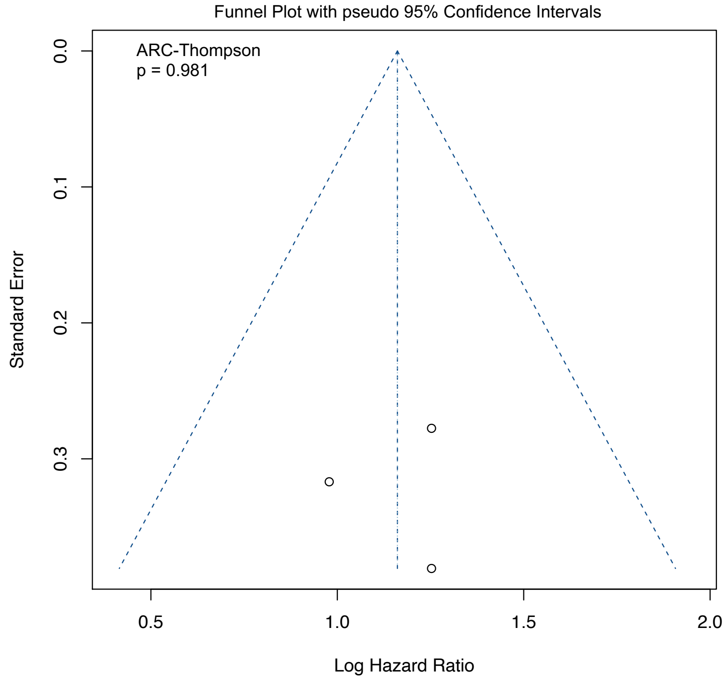

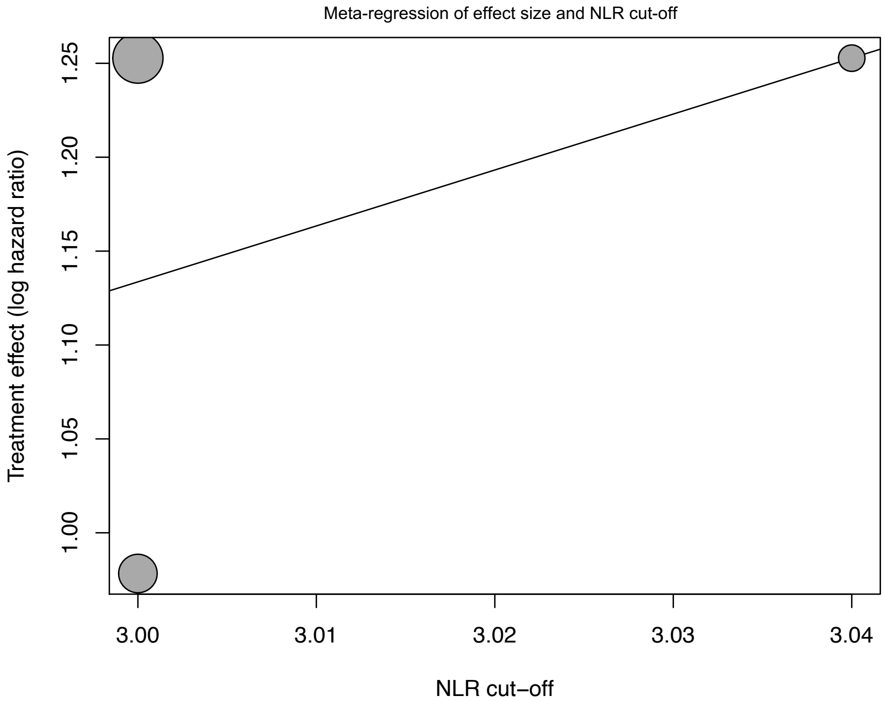

Supplementary Figure 98 - NLR: PFS in renal cancer with tyrosine-kinase inhibitors

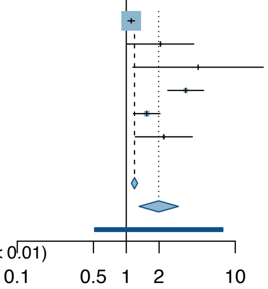

Study	ES	seES	HR	95%−CI	Weight (fixed)	Weight (random)
Cetin 2013	0.10	0.0355	1.11	[1.04; 1.19]	89.2%	23.2%
Dirican 2013	0.72	0.3586	2.06	[1.02; 4.16]	0.9%	14.0%
Gunduz 2014	1.52	0.7028	4.56	[1.15; 18.08]	0.2%	6.6%
Keizman 2014	1.26	0.1936	3.51	[2.40; 5.13]	3.0%	19.5%
Zhang 2015	0.43	0.1435	1.54	[1.16; 2.04]	5.5%	21.1%
Santoni 2015	0.79	0.3078	2.21	[1.21; 4.04]	1.2%	15.6%
**Pooled HR by Fixed Effects**			**1.19**	**[1.11; 1.27]**	**100.0%**	**--**
**Pooled HR by Random Effects**			**1.98**	**[1.31; 3.01]**	**--**	**100.0%**
**Prediction interval**				**[0.51; 7.69]**		

Heterogeneity: $I^2 = 90\%$ [80%; 95%], $\chi^2_5 = 48.32$ ($p < 0.01$)

Tau²= 0.439

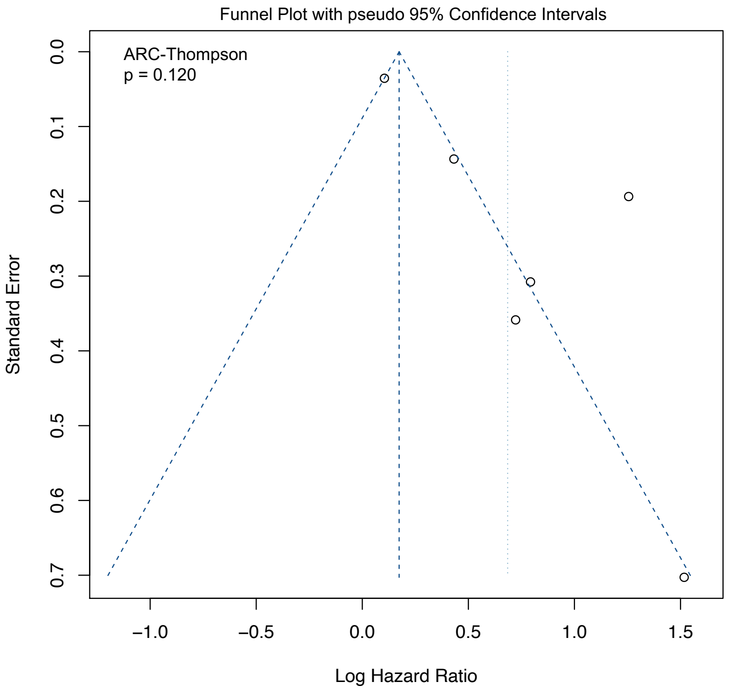

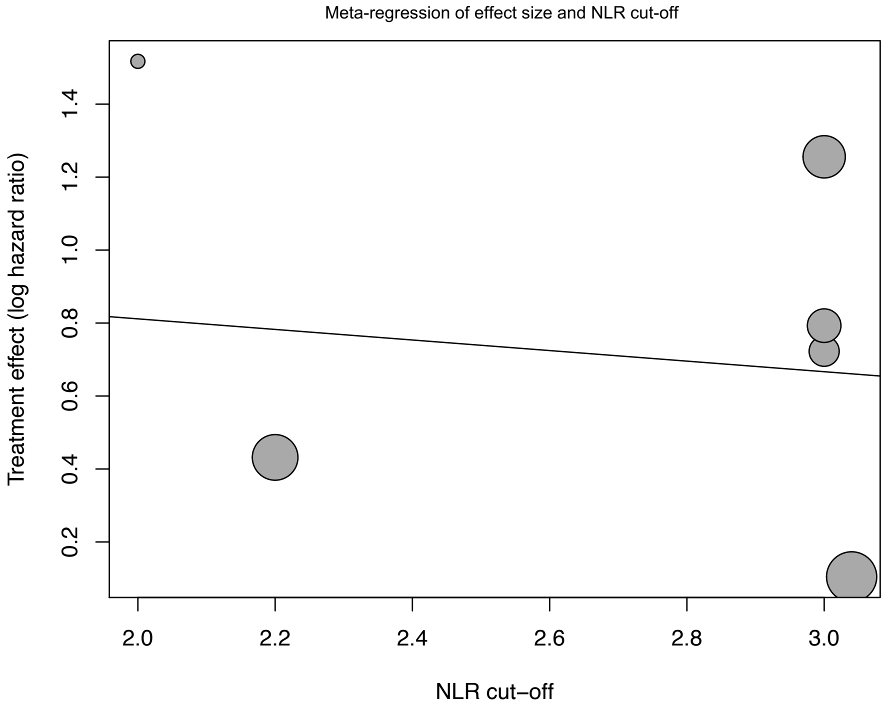

Supplementary Figure 99 - NLR: CSS in renal cancer

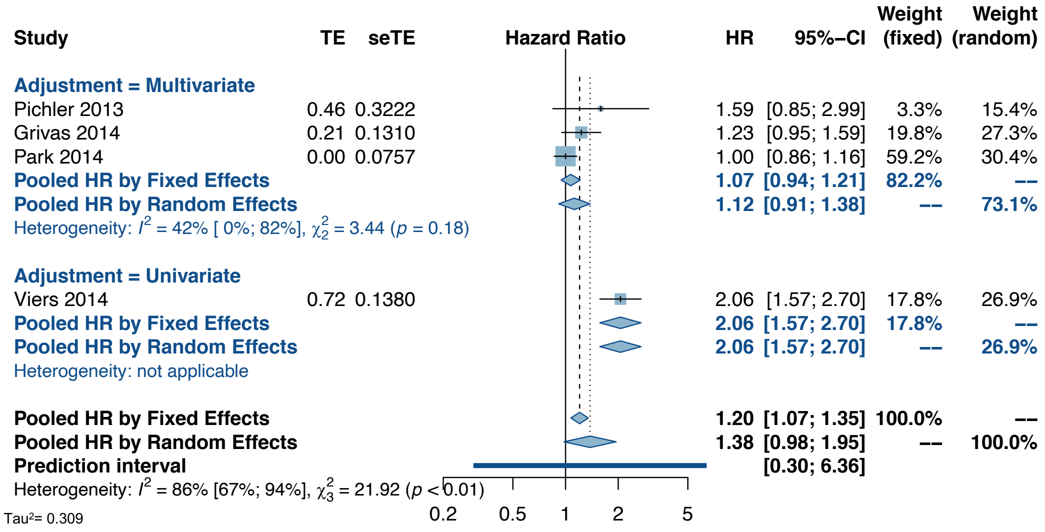

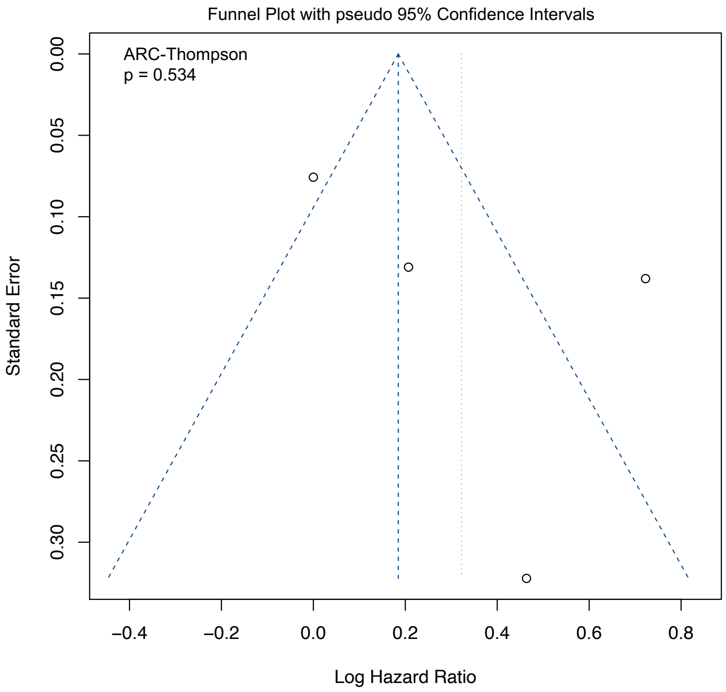

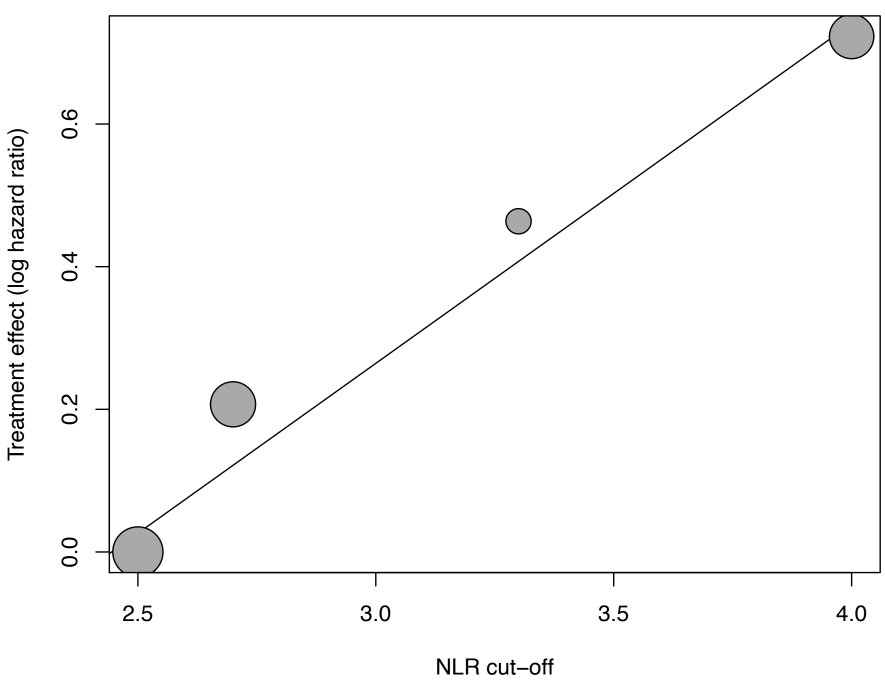

Supplementary Figure 100 - NLR: RFS in urothelial carcinoma

Study	SE	seES	HR	95%−CI	Weight (fixed)	Weight (random)
Azuma 2013	0.75	0.3819	2.11	[1.00; 4.46]	3.6%	11.6%
Lou 2014	0.90	0.3886	2.47	[1.15; 5.29]	3.5%	11.4%
Tanaka 2014	0.32	0.1550	1.38	[1.02; 1.87]	22.0%	24.2%
Song 2016	1.14	0.5216	3.13	[1.13; 8.70]	1.9%	7.6%
Altan 2017	0.61	0.2628	1.84	[1.10; 3.08]	7.7%	17.3%
Vartolomei 2017	0.05	0.0930	1.05	[0.88; 1.26]	61.2%	27.9%
**Pooled HR by Fixed Effects**			**1.26**	**[1.09; 1.45]**	**100.0%**	**--**
**Pooled HR by Random Effects**			**1.61**	**[1.16; 2.22]**	**--**	**100.0%**
**Prediction interval**				**[0.62; 4.14]**		

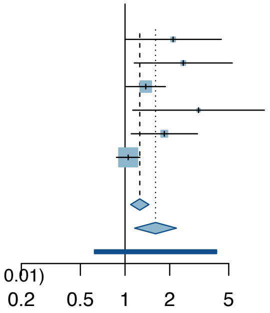

Heterogeneity: I^2 = 65% [15%; 85%], χ^2_5 = 14.13 (p = 0.01)

Tau²= 0.299

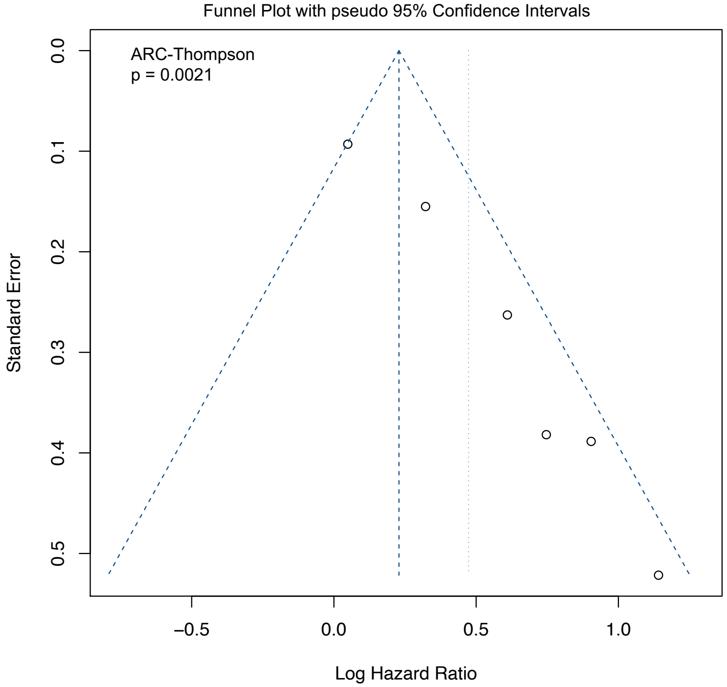

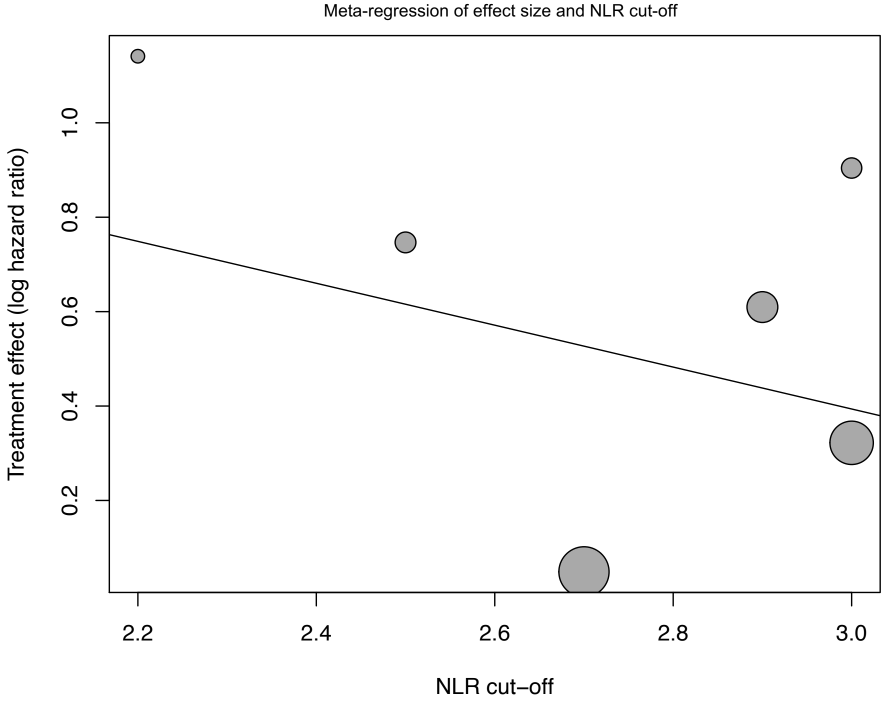

Supplementary Figure 101 - NLR: OS in urinary cancer

Study	ES	seES	HR	95%−CI	Weight (fixed)	Weight (random)
Keizman 2012	1.36	0.3040	3.88	[2.14; 7.04]	3.5%	6.7%
Cetin 2013	0.88	0.3025	2.41	[1.33; 4.36]	3.6%	6.8%
Ohno 2013	1.23	0.3804	3.43	[1.63; 7.23]	2.3%	5.2%
Pichler 2013	0.46	0.1906	1.59	[1.09; 2.31]	9.0%	10.1%
Kobayashi 2013	0.96	0.4828	2.62	[1.02; 6.75]	1.4%	3.7%
Fox 2013	0.35	0.1322	1.42	[1.10; 1.84]	18.7%	12.1%
Forget 2013	0.51	0.2655	1.67	[0.99; 2.81]	4.6%	7.7%
Santoni 2013	0.82	0.3411	2.27	[1.16; 4.43]	2.8%	5.9%
Hatakeyama 2013	0.22	0.0964	1.25	[1.03; 1.51]	35.2%	13.2%
Shafique 2012	0.44	0.2315	1.55	[0.98; 2.44]	6.1%	8.7%
Krane 2013	0.91	0.4563	2.49	[1.02; 6.09]	1.6%	4.0%
Linton 2013	0.17	0.2026	1.19	[0.80; 1.77]	8.0%	9.7%
Dalpiaz 2013	0.91	0.3262	2.48	[1.31; 4.70]	3.1%	6.2%
**Pooled HR by Fixed Effects**			**1.55**	**[1.38; 1.73]**	**100.0%**	**--**
**Pooled HR by Random Effects**			**1.83**	**[1.48; 2.26]**	**--**	**100.0%**
**Prediction interval**				**[0.95; 3.54]**		

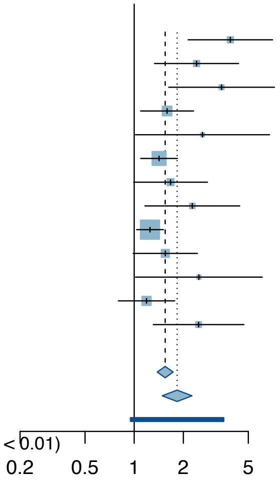

Heterogeneity: $I^2 = 58\%$ [22%; 77%], $\chi^2_{12} = 28.41$ ($p < 0.01$)

Tau²= 0.279

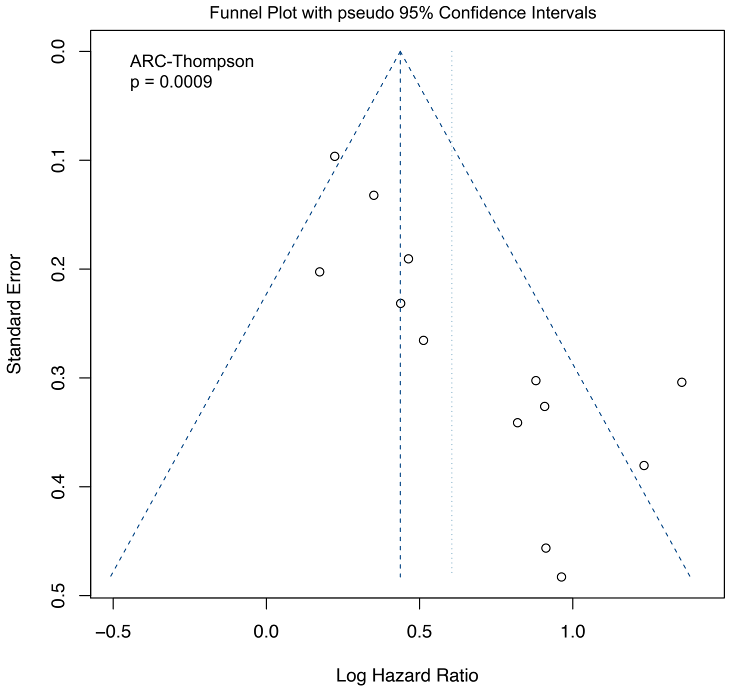

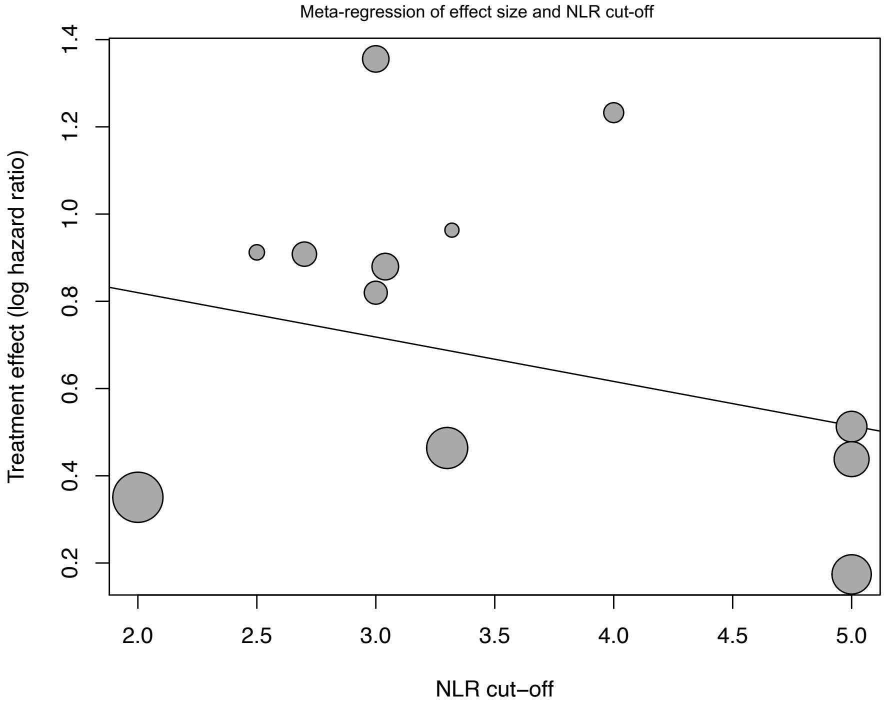

Supplementary Figure 102 - NLR: RFS in upper urinary cancer

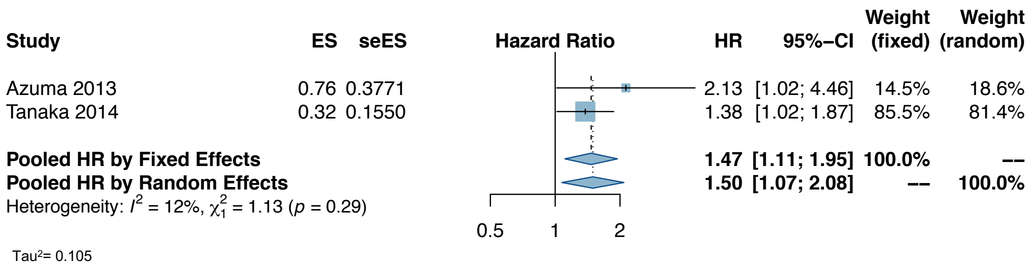

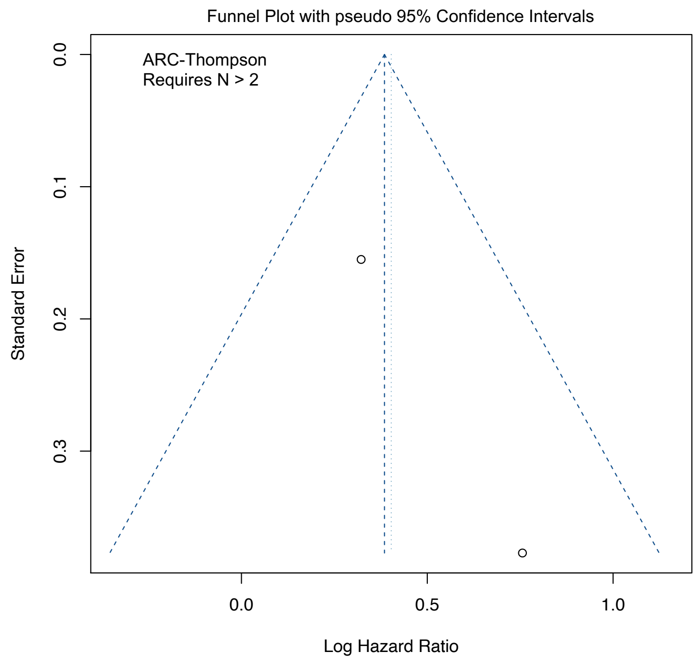

Supplementary Figure 103 - NLR: OS in bladder cancer with metastasis

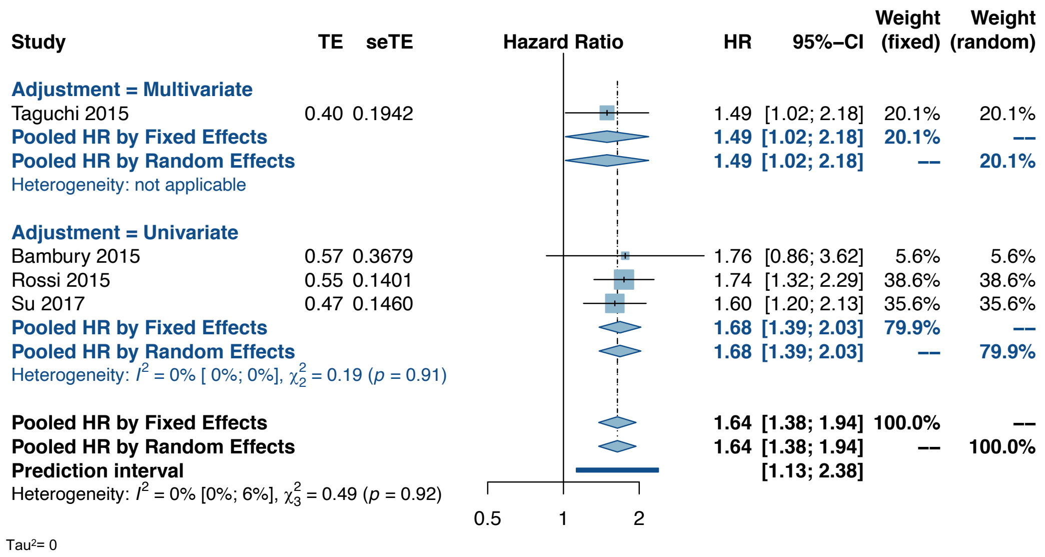

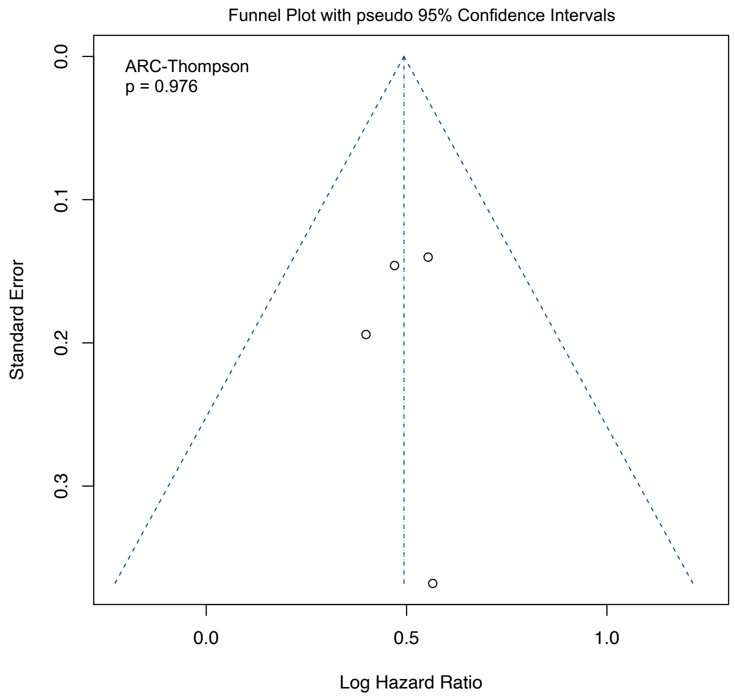

Supplementary Figure 104 - NLR: PFS in bladder and upper urinary cancer

Study	TE	seTE	HR	95%–CI	Weight (fixed)	Weight (random)
**Adjustment = Multivariate**						
Azuma 2013	0.75	0.3819	2.11	[1.00; 4.46]	1.7%	3.3%
Tanaka 2014	0.32	0.1550	1.38	[1.02; 1.87]	10.1%	9.7%
Mano 2014	1.26	0.4973	3.52	[1.33; 9.33]	1.0%	2.1%
Hermanns 2014	0.40	0.1502	1.49	[1.11; 2.00]	10.8%	9.9%
Luo 2014	0.90	0.3886	2.47	[1.15; 5.29]	1.6%	3.2%
Sung 2015	0.53	0.2089	1.70	[1.13; 2.56]	5.6%	7.4%
Song 2016	1.34	0.4786	3.82	[1.50; 9.76]	1.1%	2.3%
Morizawa 2016	0.96	0.4267	2.60	[1.13; 6.00]	1.3%	2.7%
Vartolomei 2016	0.05	0.0930	1.05	[0.88; 1.26]	28.2%	12.8%
Ku 2015	0.70	0.3184	2.02	[1.08; 3.77]	2.4%	4.3%
Ogihara 2016	0.86	0.3579	2.37	[1.18; 4.78]	1.9%	3.6%
Favilla 2016	1.68	1.3354	5.35	[0.39; 73.30]	0.1%	0.3%
Mbeutcha 2016	0.54	0.1989	1.72	[1.16; 2.54]	6.2%	7.8%
**Pooled HR by Fixed Effects**			**1.42**	**[1.27; 1.60]**	**72.0%**	**---**
**Pooled HR by Random Effects**			**1.76**	**[1.42; 2.18]**	**---**	**69.5%**
Heterogeneity: $I^2 = 59\%$ [25%; 78%], $\chi^2_{12} = 29.32$ ($p < 0.01$)						
**Adjustment = Univariate**						
Rossi 2014	0.41	0.1331	1.51	[1.16; 1.96]	13.8%	10.8%
Kim 2015	0.19	0.4244	1.21	[0.53; 2.78]	1.4%	2.8%
Kawahara 2016	0.69	0.4467	2.00	[0.83; 4.80]	1.2%	2.5%
Buisan 2016	0.61	0.3911	1.84	[0.85; 3.96]	1.6%	3.2%
Ohtake 2016	0.50	0.5096	1.65	[0.61; 4.48]	0.9%	2.0%
Bhindi 2015	0.62	0.1637	1.85	[1.34; 2.55]	9.1%	9.3%
**Pooled HR by Fixed Effects**			**1.64**	**[1.36; 1.97]**	**28.0%**	**---**
**Pooled HR by Random Effects**			**1.64**	**[1.36; 1.97]**	**---**	**30.5%**
Heterogeneity: $I^2 = 0\%$ [0%; 26%], $\chi^2_5 = 1.72$ ($p = 0.89$)						
**Pooled HR by Fixed Effects**			**1.48**	**[1.34; 1.63]**	**100.0%**	**---**
**Pooled HR by Random Effects**			**1.68**	**[1.44; 1.95]**	**---**	**100.0%**
**Prediction interval**				**[1.07; 2.62]**		
Heterogeneity: $I^2 = 45\%$ [6%; 68%], $\chi^2_{18} = 32.66$ ($p = 0.02$)						

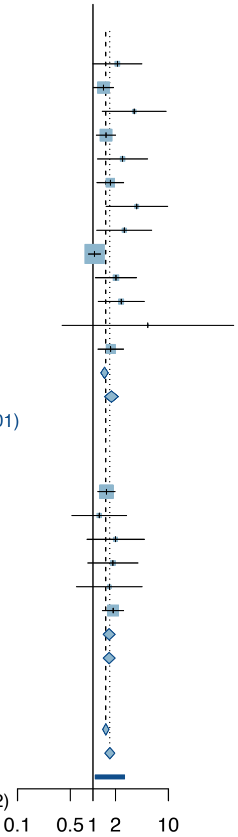

Tau²= 0.197

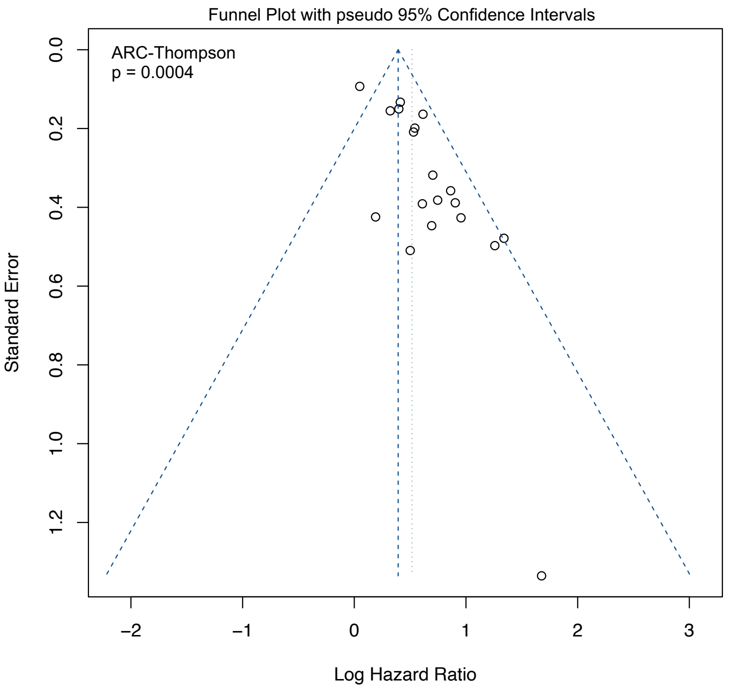

Supplementary Figure 105 - NLR: RFS in bladder cancer

Study	ES	seES	HR	95%–CI	Weight (fixed)	Weight (random)
D'Andrea 2017	0.18	0.0408	1.20	[1.11; 1.30]	61.0%	18.4%
Hermanns 2014	0.40	0.1502	1.49	[1.11; 2.00]	4.5%	11.8%
Morizawa 2016	0.96	0.4267	2.60	[1.13; 6.00]	0.6%	3.2%
Bhindi 2016	0.42	0.1349	1.52	[1.17; 1.98]	5.6%	12.8%
Mano 2015	0.62	0.2601	1.85	[1.11; 3.08]	1.5%	6.7%
Favilla 2016	1.04	0.3599	2.84	[1.40; 5.75]	0.8%	4.2%
Mbeutcha 2016	0.24	0.0950	1.27	[1.05; 1.53]	11.3%	15.4%
Ogihara 2016	0.73	0.1331	2.08	[1.60; 2.70]	5.7%	12.9%
D'Andrea 2017	0.26	0.1059	1.30	[1.06; 1.60]	9.1%	14.7%
**Pooled HR by Fixed Effects**			**1.31**	**[1.23; 1.39]**	**100.0%**	**--**
**Pooled HR by Random Effects**			**1.52**	**[1.29; 1.79]**	**--**	**100.0%**
**Prediction interval**				**[0.93; 2.48]**		

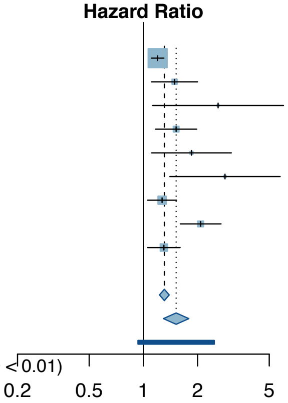

Heterogeneity: $I^2 = 71\%$ [43%; 85%], $\chi^2_8 = 27.70$ ($p < 0.01$)

Tau²= 0.189

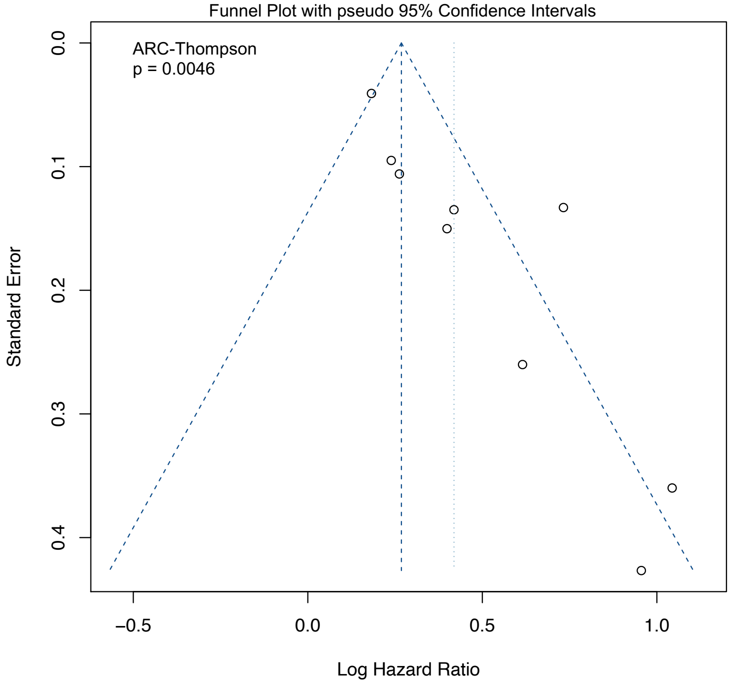

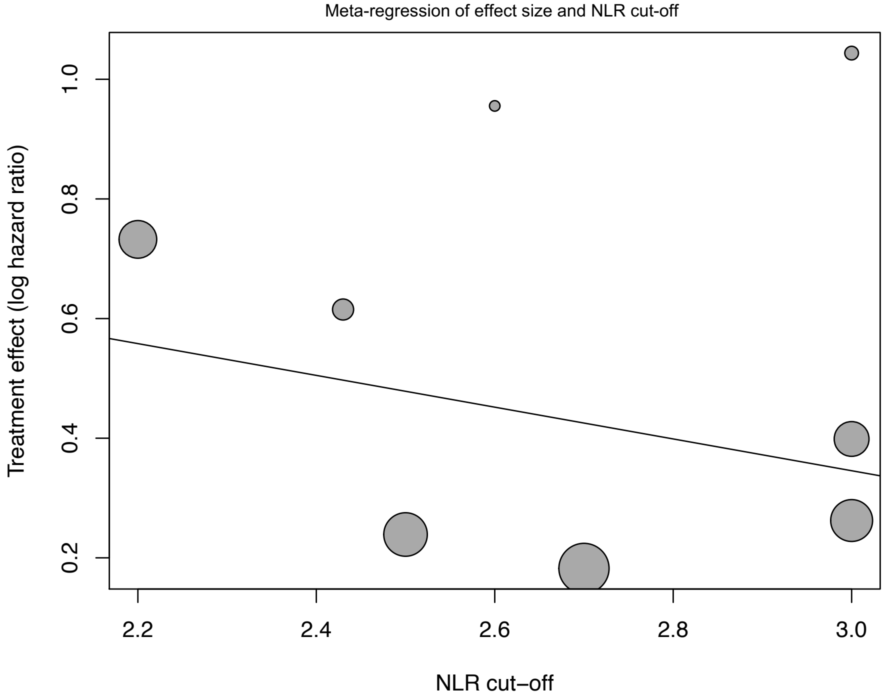

Supplementary Figure 106 - NLR: CSS in bladder and upper urinary cancer

Study	TE	seTE	HR	95%-CI	Weight (fixed)	Weight (random)
**Adjustment = Multivariate**						
Gondo 2011	0.67	0.3212	1.95	[1.04; 3.66]	1.2%	4.1%
Azuma 2013	1.12	0.4096	3.06	[1.37; 6.83]	0.8%	2.9%
Dalpiaz 2013	1.00	0.3976	2.72	[1.25; 5.93]	0.8%	3.0%
Tanaka 2014	0.39	0.1844	1.47	[1.02; 2.11]	3.7%	7.6%
Hermanns 2014	0.63	0.1535	1.88	[1.39; 2.54]	5.3%	8.7%
Luo 2014	1.85	0.6611	6.38	[1.75; 23.31]	0.3%	1.3%
Taguchi 2015	0.39	0.1952	1.48	[1.01; 2.17]	3.3%	7.2%
Cheng 2015	0.31	0.3775	1.36	[0.65; 2.85]	0.9%	3.3%
Ozcan 2015	0.68	0.3062	1.97	[1.08; 3.59]	1.3%	4.4%
Kang 2015	0.15	0.0502	1.16	[1.05; 1.28]	50.0%	12.3%
Morizawa 2016	0.96	0.3536	2.60	[1.30; 5.20]	1.0%	3.6%
Vartolomei 2016	0.07	0.1032	1.07	[0.87; 1.31]	11.8%	10.6%
**Pooled HR by Fixed Effects**			**1.27**	**[1.18; 1.38]**	**80.4%**	**--**
**Pooled HR by Random Effects**			**1.66**	**[1.34; 2.06]**	**--**	**68.7%**
Heterogeneity: $I^2 = 69\%$ [45%; 83%], $\chi^2_{11} = 35.99$ ($p < 0.01$)						
**Adjustment = Univariate**						
Viers 2014	0.39	0.1118	1.47	[1.18; 1.83]	10.1%	10.2%
Sung 2015	0.71	0.1812	2.04	[1.43; 2.91]	3.8%	7.7%
Kim 2015	0.17	0.4261	1.18	[0.51; 2.72]	0.7%	2.7%
Buisan 2016	0.63	0.4201	1.87	[0.82; 4.26]	0.7%	2.8%
Bhindi 2015	0.54	0.1729	1.71	[1.22; 2.40]	4.2%	8.0%
**Pooled HR by Fixed Effects**			**1.62**	**[1.39; 1.90]**	**19.6%**	**--**
**Pooled HR by Random Effects**			**1.63**	**[1.38; 1.93]**	**--**	**31.3%**
Heterogeneity: $I^2 = 0\%$ [0%; 74%], $\chi^2_4 = 3.14$ ($p = 0.53$)						
**Pooled HR by Fixed Effects**			**1.34**	**[1.25; 1.43]**	**100.0%**	**--**
**Pooled HR by Random Effects**			**1.62**	**[1.39; 1.89]**	**--**	**100.0%**
**Prediction interval**				**[0.99; 2.66]**		
Heterogeneity: $I^2 = 66\%$ [42%; 79%], $\chi^2_{16} = 46.40$ ($p < 0.01$)						

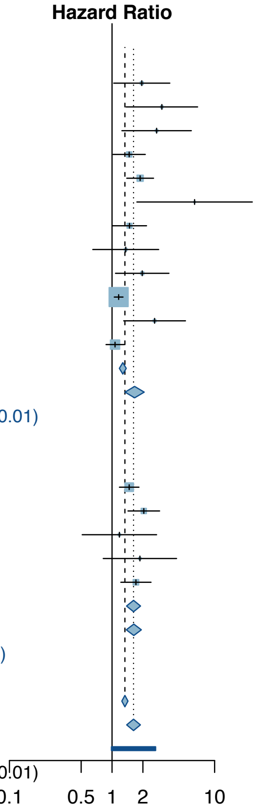

Tau²= 0.219

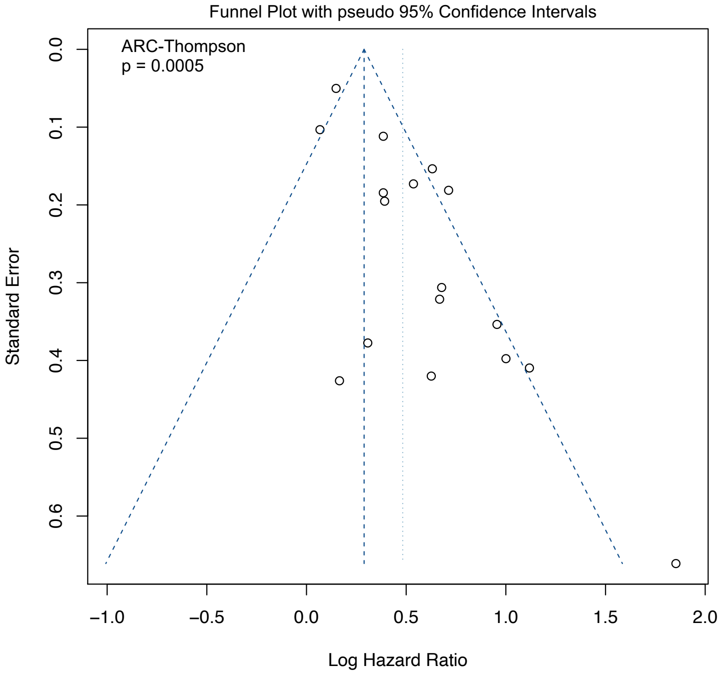

Supplementary Figure 107 - NLR: PFS in castration resistant prostate cancer

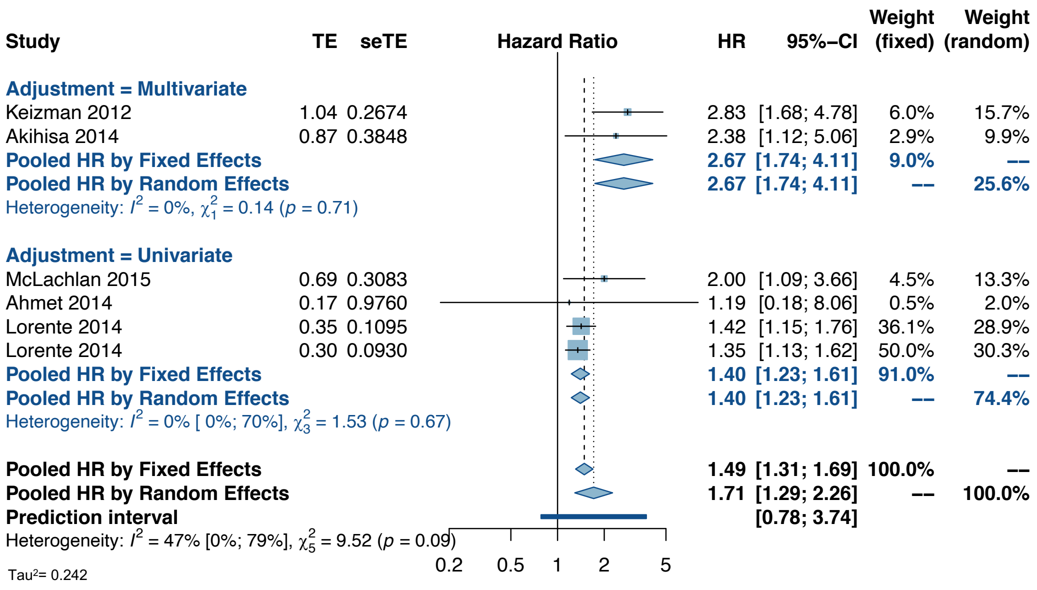

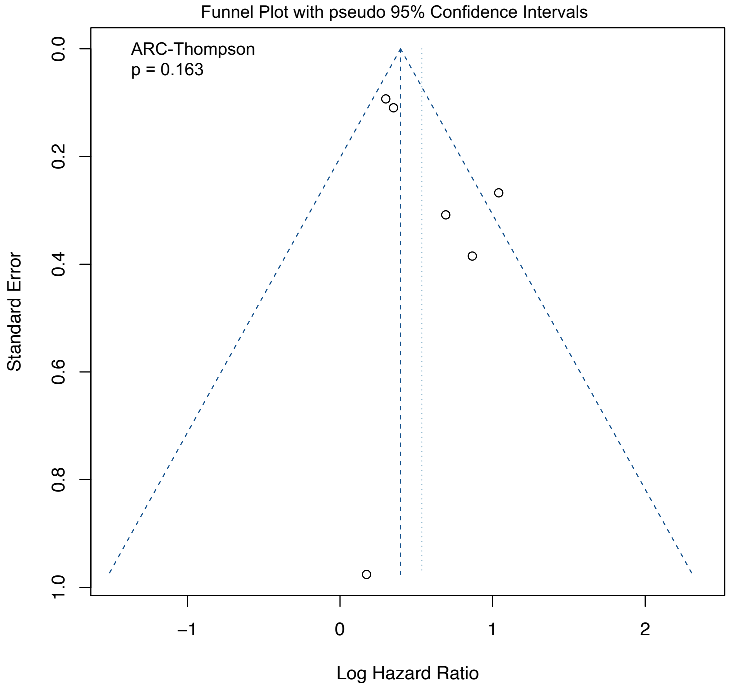

Supplementary Figure 108 - NLR: OS in castration resistant prostate cancer

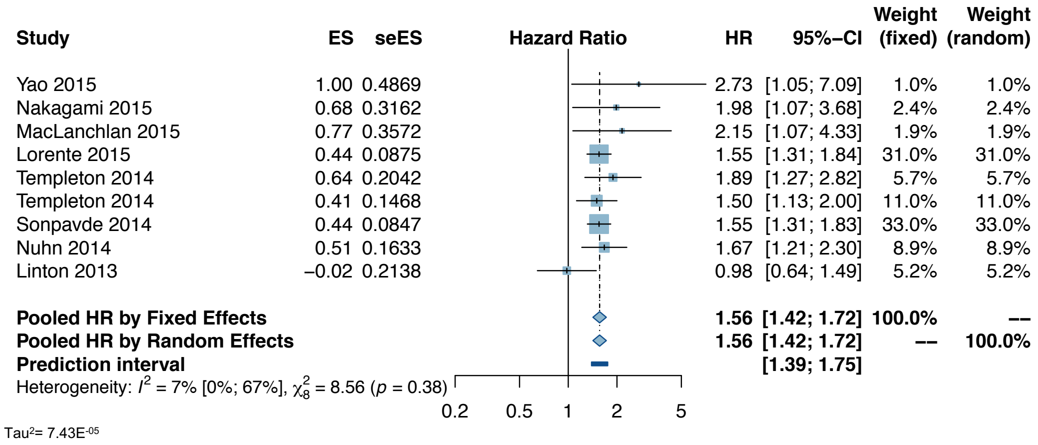

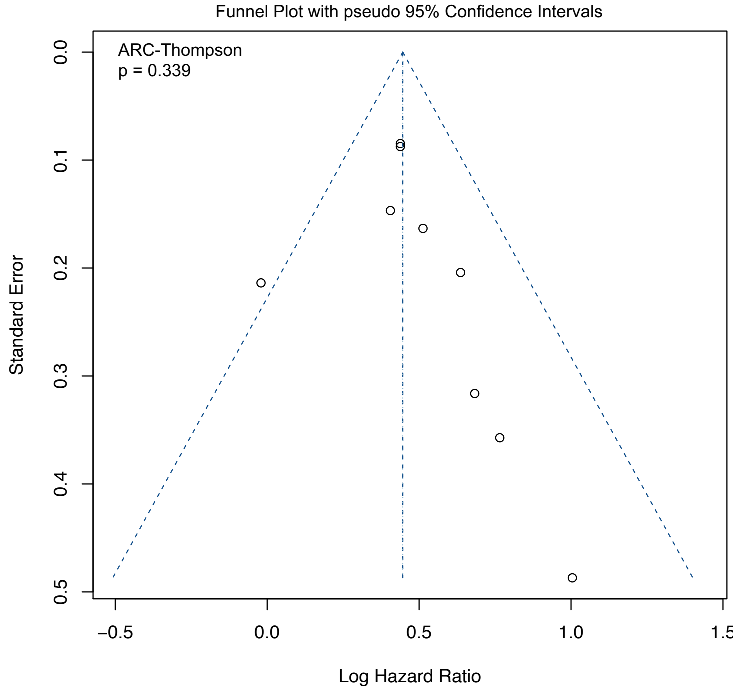

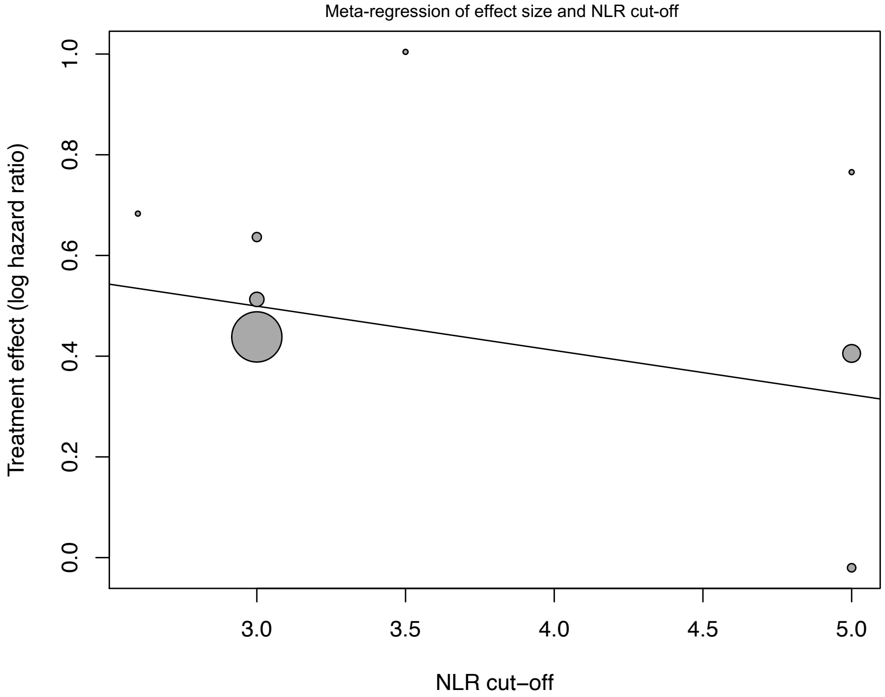

Supplementary Figure 109 - NLR: OS in localised prostate cancer

Study	ES	seES	Hazard Ratio	HR	95%–CI	Weight (fixed)	Weight (random)
Sharma 2015	0.11	0.0601		1.12	[1.00; 1.26]	96.4%	60.9%
Langenlehner 2015	0.77	0.3131		2.16	[1.17; 3.99]	3.6%	39.1%
**Pooled HR by Fixed Effects**				**1.15**	**[1.02; 1.29]**	**100.0%**	**––**
**Pooled HR by Random Effects**				**1.45**	**[0.77; 2.71]**	**––**	**100.0%**

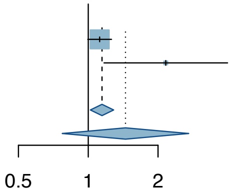

Heterogeneity: $I^2 = 76\%$, $\chi^2_1 = 4.24$ ($p = 0.04$)

Tau²= 0.406

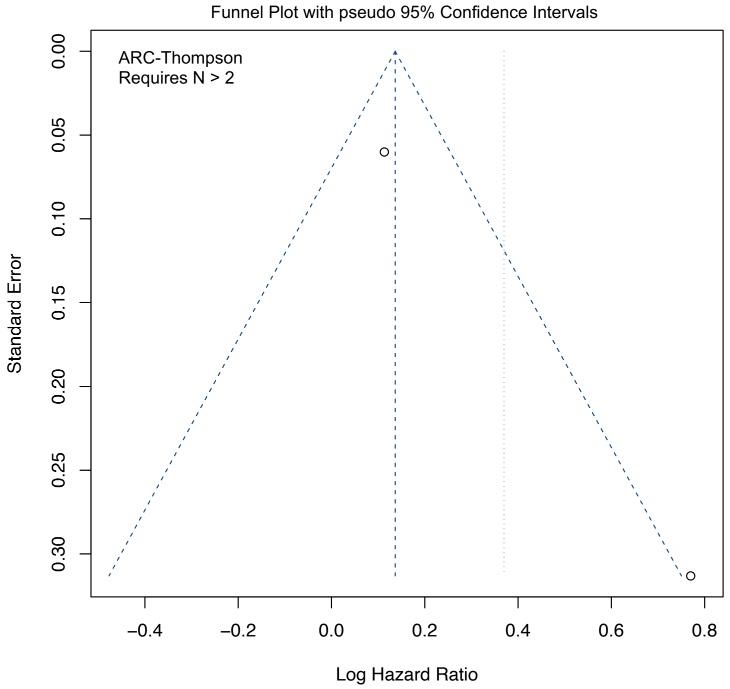

Supplementary Figure 110 - NLR: RFS in non muscle-invasive bladder cancer

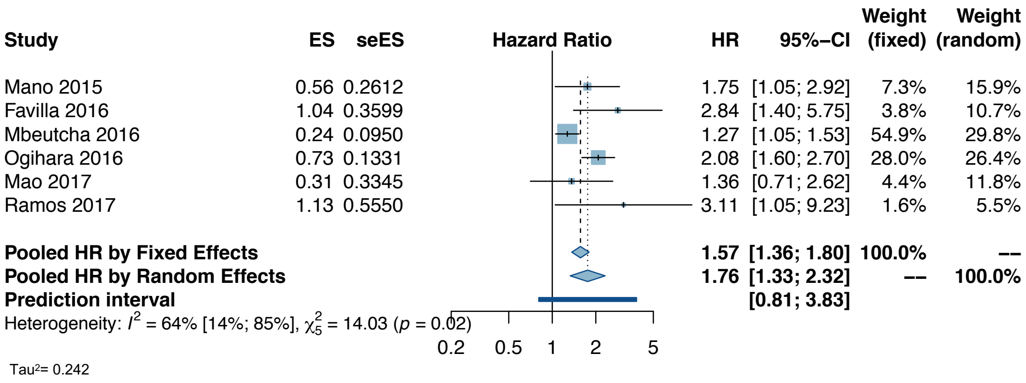

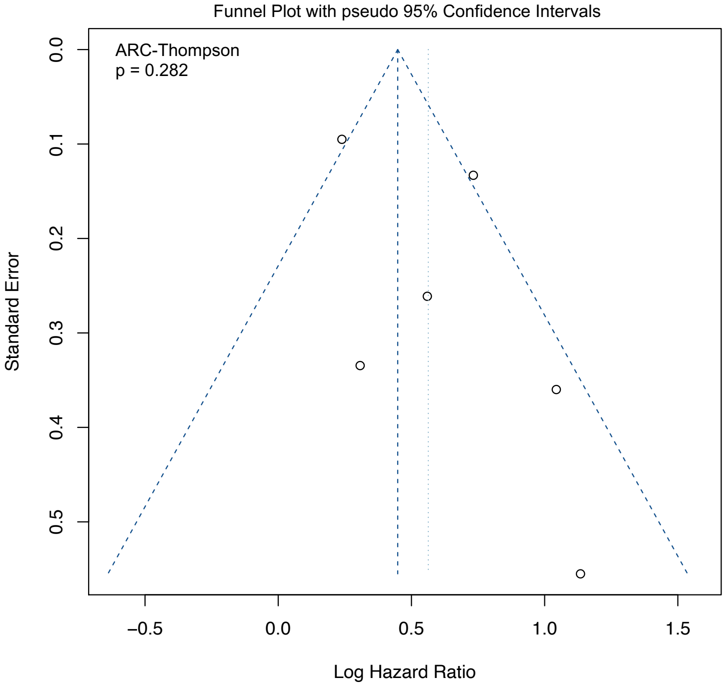

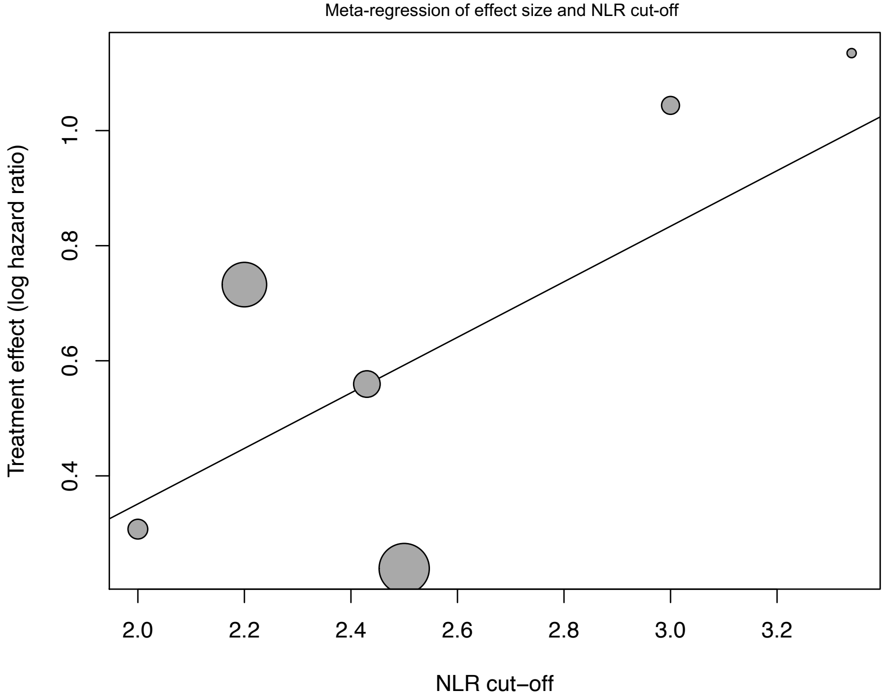

Supplementary Figure 111 - NLR: PFS in non muscle-invasive bladder cancer

Study	ES	seES	HR	95%-CI	Weight (fixed)	Weight (random)
Mano 2015	1.26	0.4973	3.52	[1.33; 9.33]	9.2%	11.7%
Favilla 2016	1.68	1.3382	5.35	[0.39; 73.70]	1.3%	1.8%
Mbeutcha 2016	0.54	0.1989	1.72	[1.16; 2.54]	57.4%	47.0%
Ogihara 2016	0.86	0.3579	2.37	[1.18; 4.78]	17.7%	20.6%
Mao 2017	0.76	0.5284	2.13	[0.76; 6.00]	8.1%	10.5%
Ramos 2017	1.51	0.5993	4.51	[1.39; 14.60]	6.3%	8.4%
**Pooled HR by Fixed Effects**			**2.13**	**[1.59; 2.87]**	**100.0%**	**--**
**Pooled HR by Random Effects**			**2.26**	**[1.59; 3.22]**	**--**	**100.0%**
**Prediction interval**				**[1.13; 4.52]**		

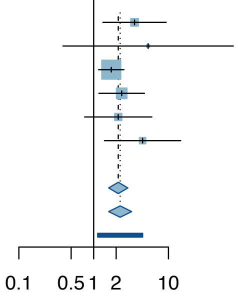

Heterogeneity: $I^2 = 0\%$ [0%; 71%], $\chi^2_5 = 4.30$ ($p = 0.51$)

Tau²= 0.172

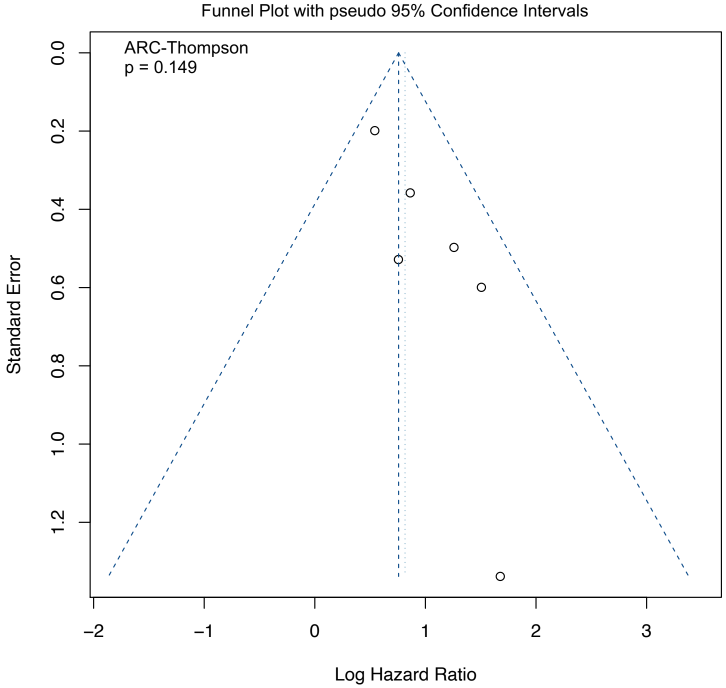

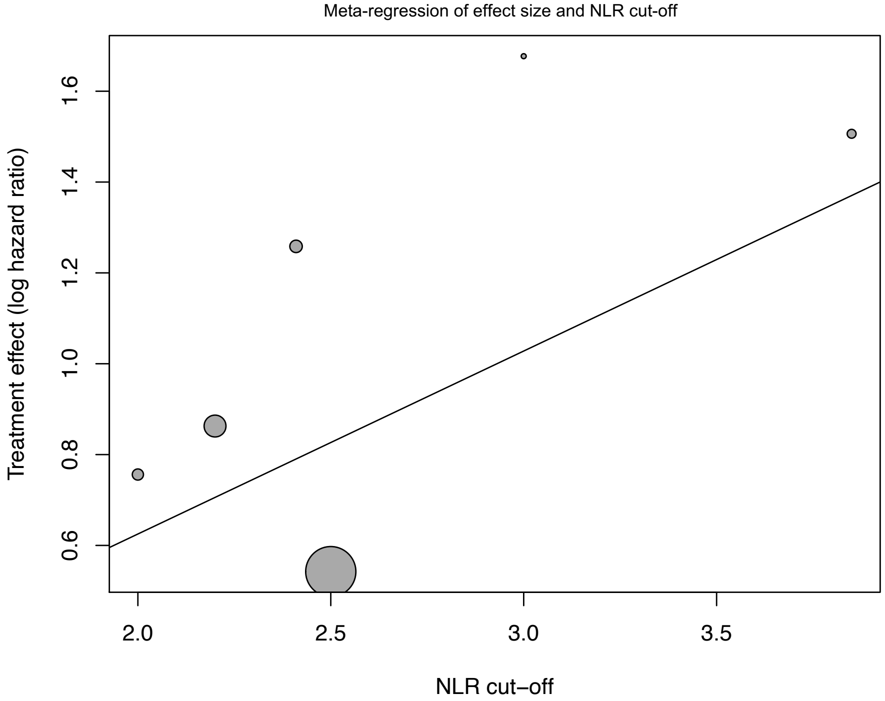

Supplementary Figure 112 - NLR: RFS in high risk non muscle-invasive bladder cancer

Study	ES	seES	HR	95%–CI	Weight (fixed)	Weight (random)
Ozyalcali 2015	1.34	0.4752	3.81	[1.50; 9.67]	10.9%	21.4%
Mbeutcha 2016	0.38	0.1879	1.46	[1.01; 2.11]	69.7%	38.6%
Camtosun 2017	1.59	0.5457	4.90	[1.68; 14.28]	8.3%	18.4%
Martha 2017	0.51	0.4705	1.67	[0.66; 4.20]	11.1%	21.7%
**Pooled HR by Fixed Effects**			**1.82**	**[1.34; 2.47]**	**100.0%**	**--**
**Pooled HR by Random Effects**			**2.31**	**[1.27; 4.18]**	**--**	**100.0%**
**Prediction interval**				**[0.22; 23.88]**		

Heterogeneity: $I^2 = 58\%$ [0%; 86%], $\chi^2_3 = 7.12$ ($p = 0.07$)

Tau²= 0.451

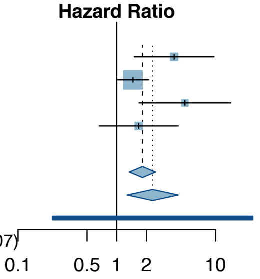

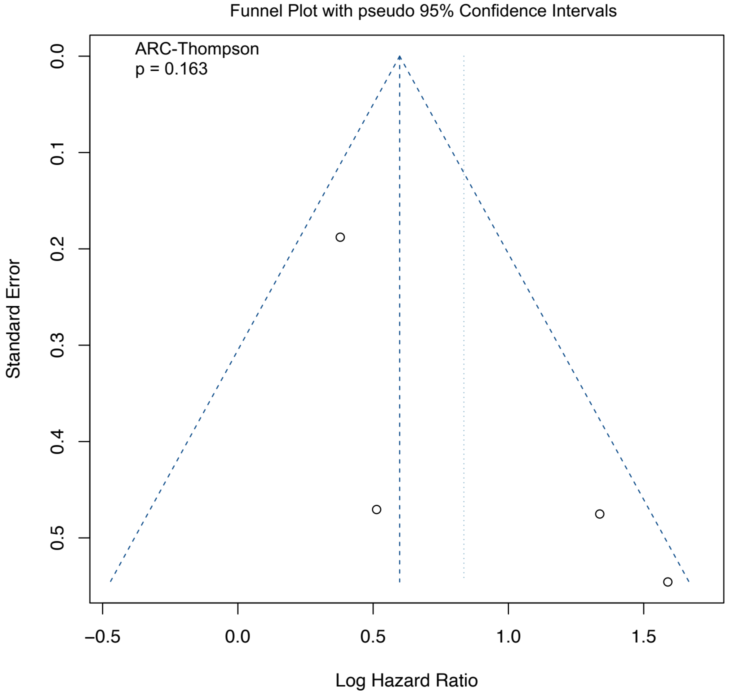

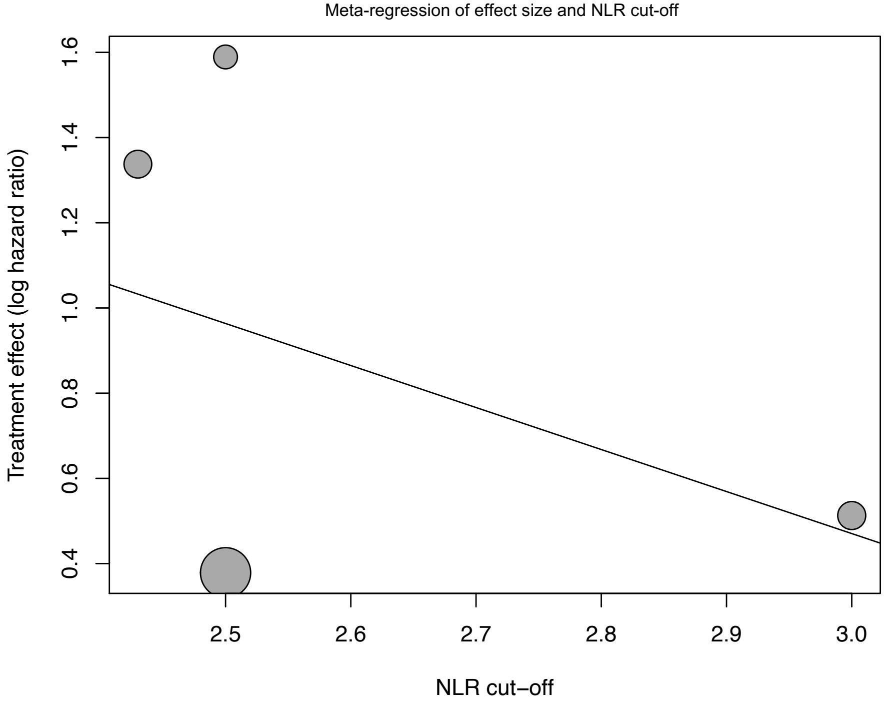

Supplementary Figure 113 - NLR: PFS in high risk non muscle-invasive bladder cancer

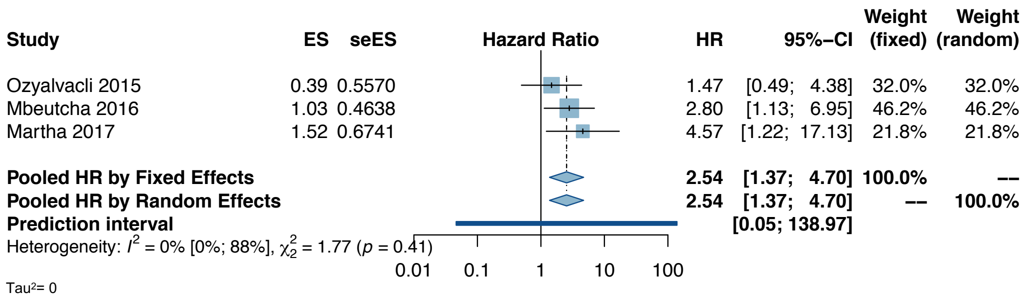

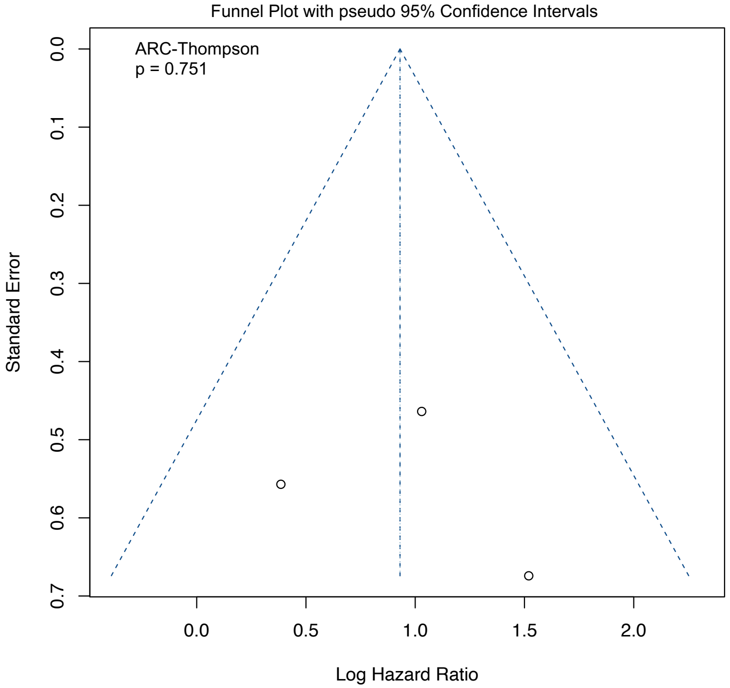

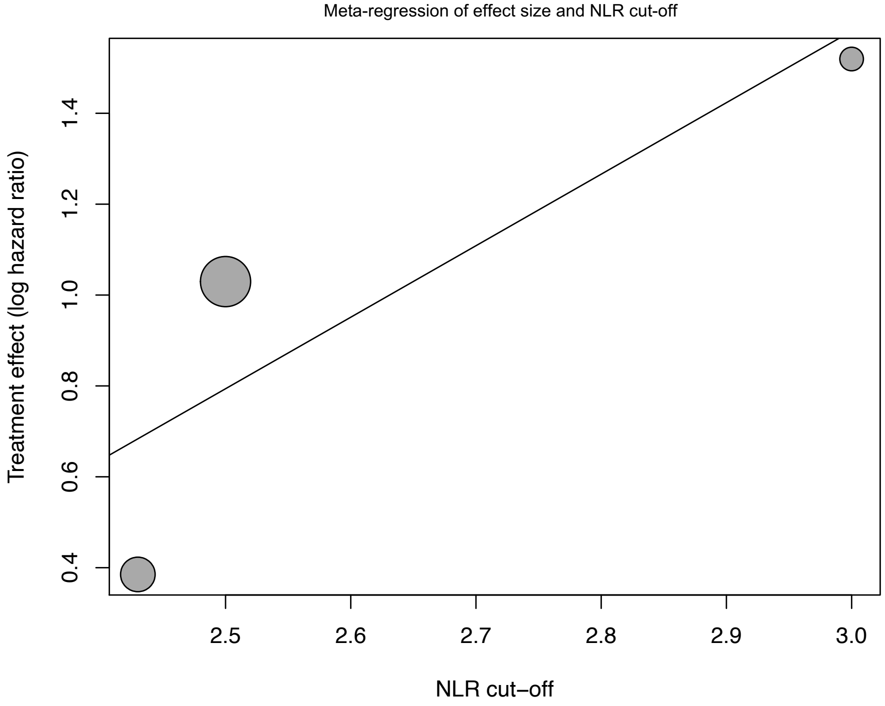

Supplementary Figure 114 - NLR: OS in STS

Study	TE	seTE	Hazard Ratio	HR	95%–CI	Weight (fixed)	Weight (random)
Yanagisawa (1) 2018	0.22	0.1945		1.25	[0.85; 1.83]	3.3%	9.3%
Yanagisawa (2) 2018	0.03	0.0427		1.03	[0.95; 1.12]	68.7%	14.8%
Kobayashi 2018	0.05	0.5183		1.05	[0.38; 2.90]	0.5%	2.7%
Idowu 2012	1.64	0.7213		5.13	[1.25; 21.09]	0.2%	1.6%
Li 2017	0.73	0.2936		2.07	[1.16; 3.68]	1.5%	6.2%
Szkandera 2015	0.47	0.2069		1.60	[1.07; 2.40]	2.9%	8.8%
Nakamura 2017	0.23	0.1251		1.26	[0.99; 1.61]	8.0%	12.1%
Que 2015	0.06	0.3632		1.06	[0.52; 2.16]	1.0%	4.7%
Xia 2016	0.59	0.1468		1.80	[1.35; 2.40]	5.8%	11.2%
Liu 2016	0.74	0.2834		2.10	[1.20; 3.66]	1.6%	6.5%
Jiang 2015	0.91	0.2820		2.48	[1.43; 4.31]	1.6%	6.5%
Liang 2017	0.79	0.2973		2.20	[1.23; 3.94]	1.4%	6.1%
Maretty–Kongstad 2017	0.59	0.1876		1.80	[1.25; 2.60]	3.6%	9.6%
**Pooled HR by Fixed Effects**				**1.18**	**[1.10; 1.27]**	**100.0%**	**--**
**Pooled HR by Random Effects**				**1.56**	**[1.30; 1.88]**	**--**	**100.0%**
**Prediction interval**					**[0.88; 2.77]**		

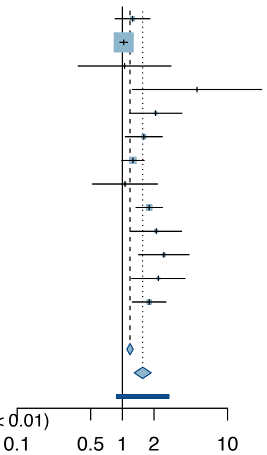

Heterogeneity: $I^2 = 76\%$ [58%; 86%], $\chi^2_{12} = 49.41$ ($p < 0.01$)

Tau²= 0.243

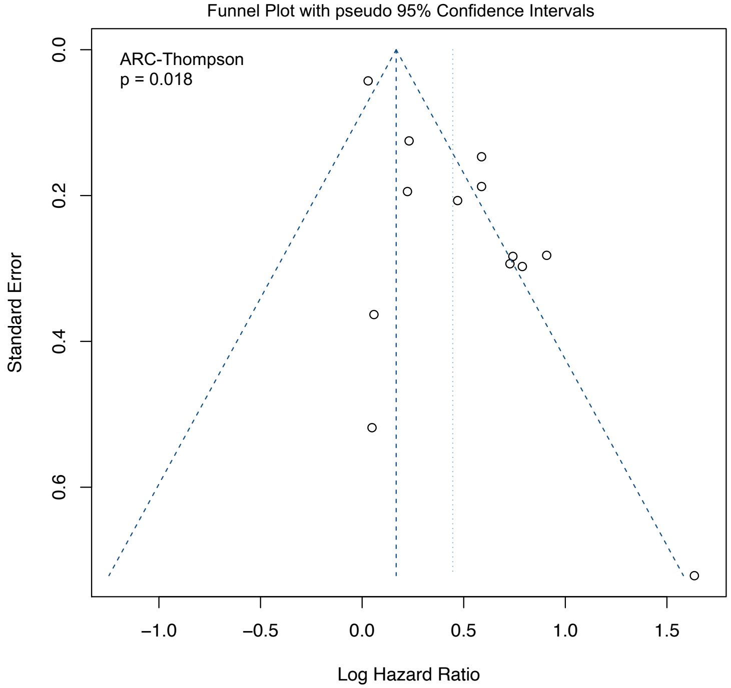

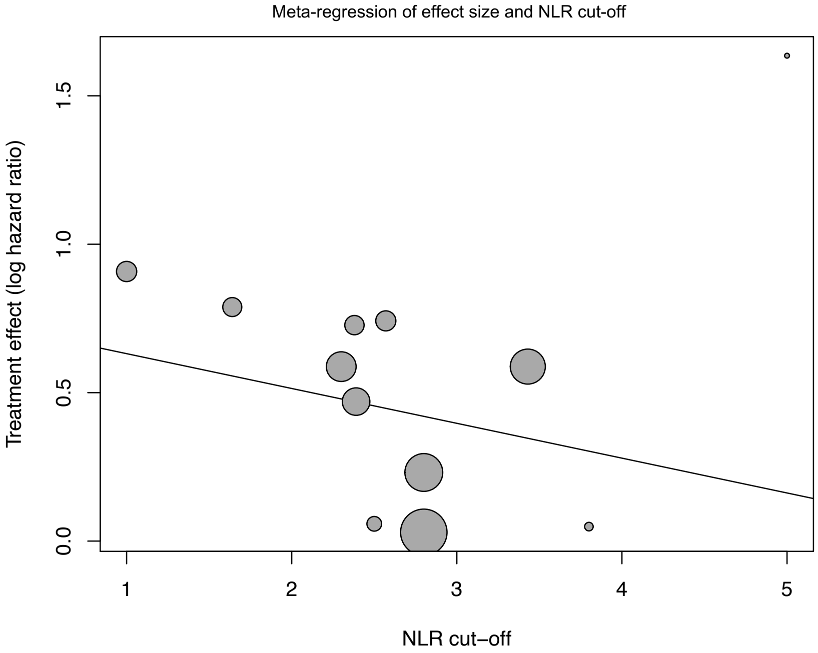

Supplementary Figure 115 - NLR: DFS in STS

Study	TE	seTE	HR	95%−CI	Weight (fixed)	Weight (random)
Chan (L) 2018	0.49	0.1244	1.63	[1.28; 2.08]	59.3%	59.3%
Chen 2019	1.15	0.5042	3.16	[1.18; 8.49]	3.6%	3.6%
Cheng 2019	1.21	0.7318	3.36	[0.80; 14.10]	1.7%	1.7%
Idowu 2012	1.40	0.7316	4.05	[0.97; 16.99]	1.7%	1.7%
Jiang 2015	0.43	0.1990	1.53	[1.04; 2.26]	23.2%	23.2%
Szkandera (V) 2014	0.50	0.4093	1.65	[0.74; 3.68]	5.5%	5.5%
Szkandera (T) 2014	0.82	0.4255	2.28	[0.99; 5.25]	5.1%	5.1%
**Pooled HR by Fixed Effects**			**1.72**	**[1.43; 2.08]**	**100.0%**	**--**
**Pooled HR by Random Effects**			**1.72**	**[1.43; 2.08]**	**--**	**100.0%**
**Prediction interval**				**[1.35; 2.20]**		

Heterogeneity: $I^2 = 0\%$ [0%; 62%], $\chi^2_6 = 4.64$ ($p = 0.59$)

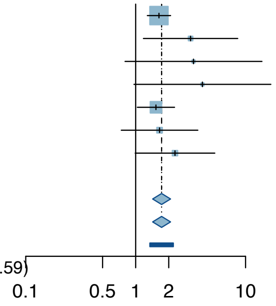

Tau²= 0

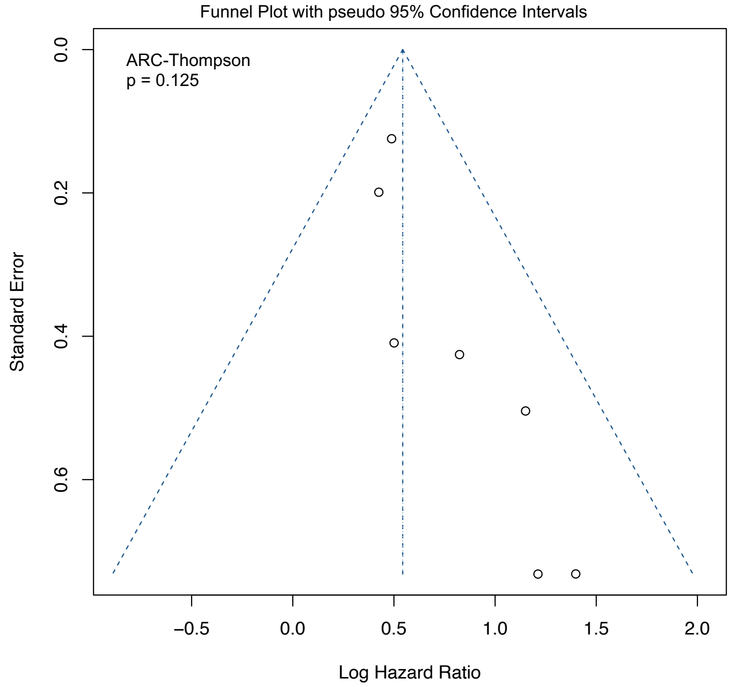

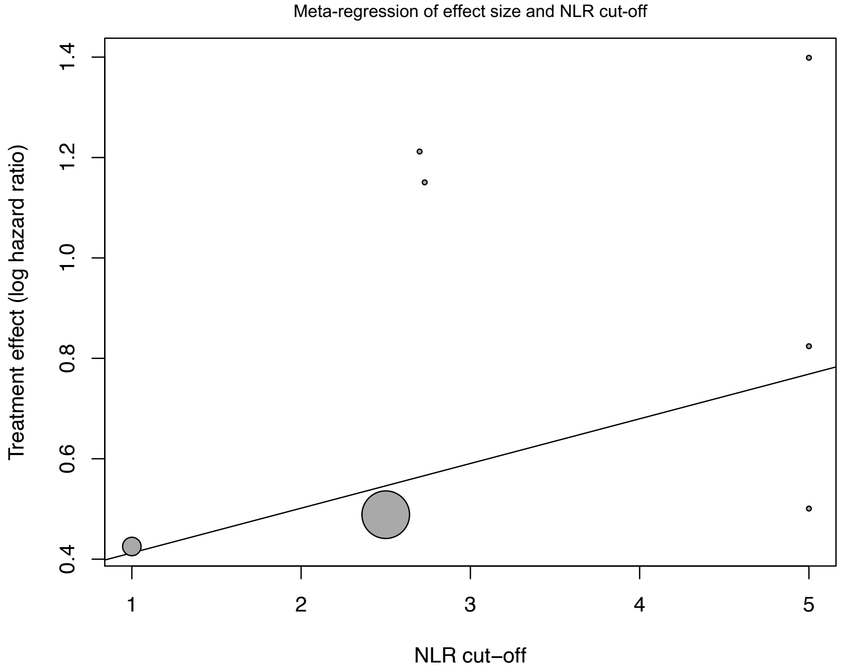

Supplementary Figure 116 - NLR: OS in STS (Synovial Sarcoma)

Study	TE	seTE	HR	95%–CI	Weight (fixed)	Weight (random)
Cheng 2019	1.62	0.7361	5.07	[1.20; 21.46]	2.6%	2.6%
Garcia–Ortega 2017	0.83	0.1253	2.30	[1.80; 2.94]	91.0%	91.0%
Chan	1.09	0.4733	2.97	[1.17; 7.51]	6.4%	6.4%
**Pooled HR by Fixed Effects**			**2.39**	**[1.89; 3.02]**	**100.0%**	**--**
**Pooled HR by Random Effects**			**2.39**	**[1.89; 3.02]**	**--**	**100.0%**
**Prediction interval**				**[0.52; 10.89]**		

Heterogeneity: $I^2 = 0\%$ [0%; 85%], $\chi^2_2 = 1.35$ ($p = 0.51$)

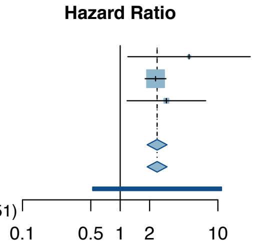

Tau²= 0

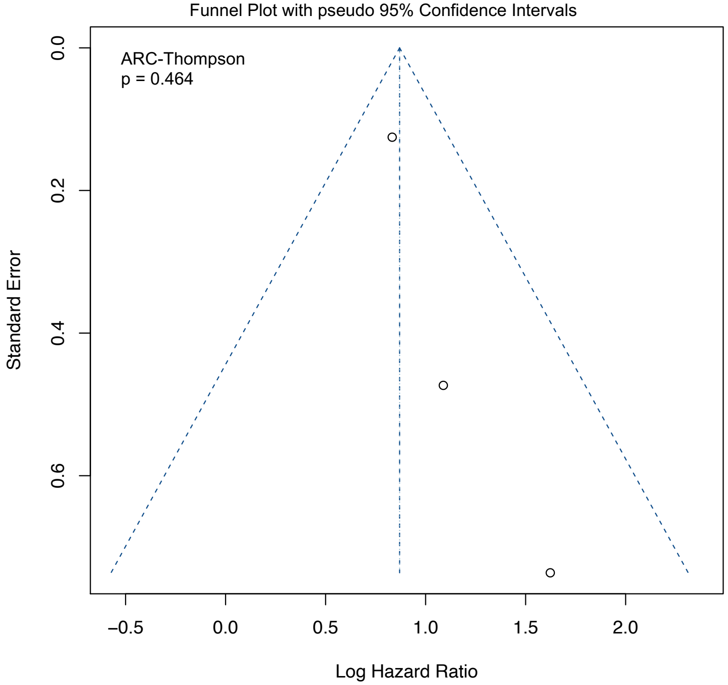

Supplementary Figure 117 - NLR: OS in STS (Liposarcoma)

Study	TE	seTE	HR	95%–CI	Weight (fixed)	Weight (random)
Pantopoulos 2015	1.10	0.4852	3.01	[1.16; 7.79]	26.1%	26.1%
Chan	1.07	0.2883	2.91	[1.65; 5.12]	73.9%	73.9%
**Pooled HR by Fixed Effects**			**2.94**	**[1.81; 4.77]**	**100.0%**	**--**
**Pooled HR by Random Effects**			**2.94**	**[1.81; 4.77]**	**--**	**100.0%**

Heterogeneity: $I^2 = 0\%$, $\chi^2_1 = 0.00$ ($p = 0.95$)

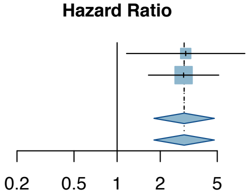

Tau²= 0

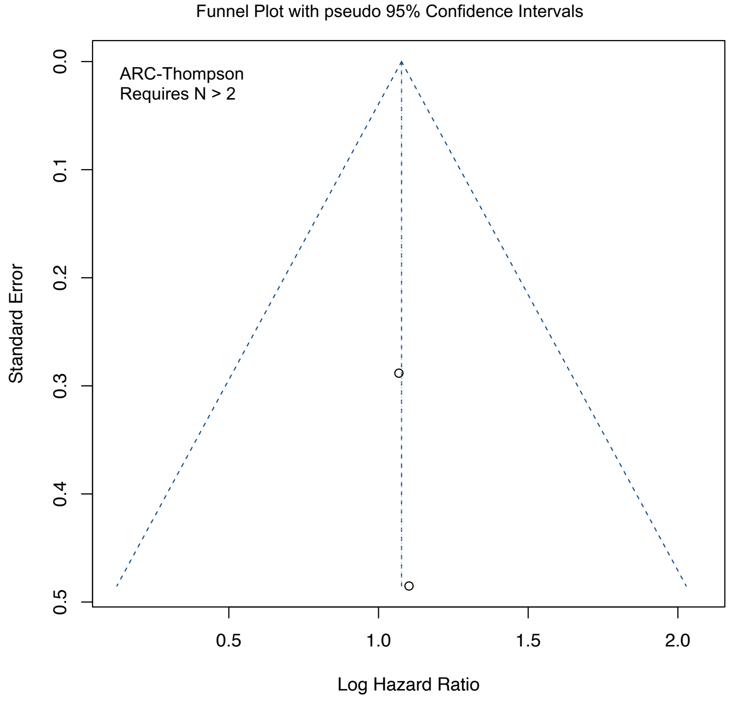

Supplementary Figure 118 - NLR: OS in Multiple Myeloma

Study	TE	seTE	HR	95%−CI	Weight (fixed)	Weight (random)
Kelkitli 2014	0.92	0.3716	2.52	[1.22; 5.22]	0.2%	9.2%
Kim 2016	0.44	0.1914	1.56	[1.07; 2.27]	0.9%	16.6%
Shi 2016	0.75	0.1955	2.12	[1.45; 3.11]	0.9%	16.3%
Li 2016	0.09	0.0184	1.09	[1.05; 1.13]	96.9%	23.2%
Onec 2017	−0.12	0.7743	0.89	[0.20; 4.06]	0.1%	3.1%
Romano 2015	0.80	0.2167	2.23	[1.46; 3.41]	0.7%	15.3%
Wongrakpanich 2016	1.06	0.4056	2.89	[1.31; 6.40]	0.2%	8.3%
Atallah 2013	0.31	0.4104	1.36	[0.61; 3.04]	0.2%	8.1%
**Pooled HR by Fixed Effects**			**1.11**	**[1.07; 1.15]**	**100.0%**	**--**
**Pooled HR by Random Effects**			**1.70**	**[1.28; 2.27]**	**--**	**100.0%**
**Prediction interval**				**[0.75; 3.86]**		

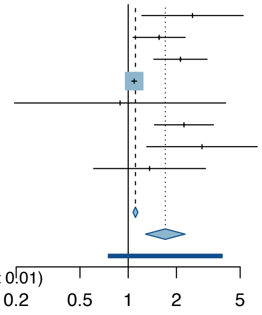

Heterogeneity: $I^2 = 81\%$ [63%; 90%], $\chi^2_7 = 36.22$ ($p < 0.01$)

Tau²= 0.301

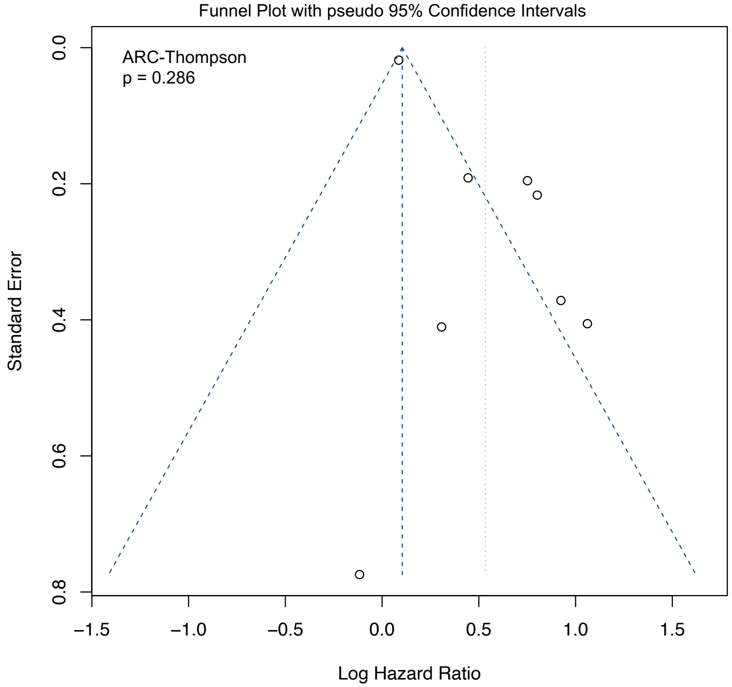

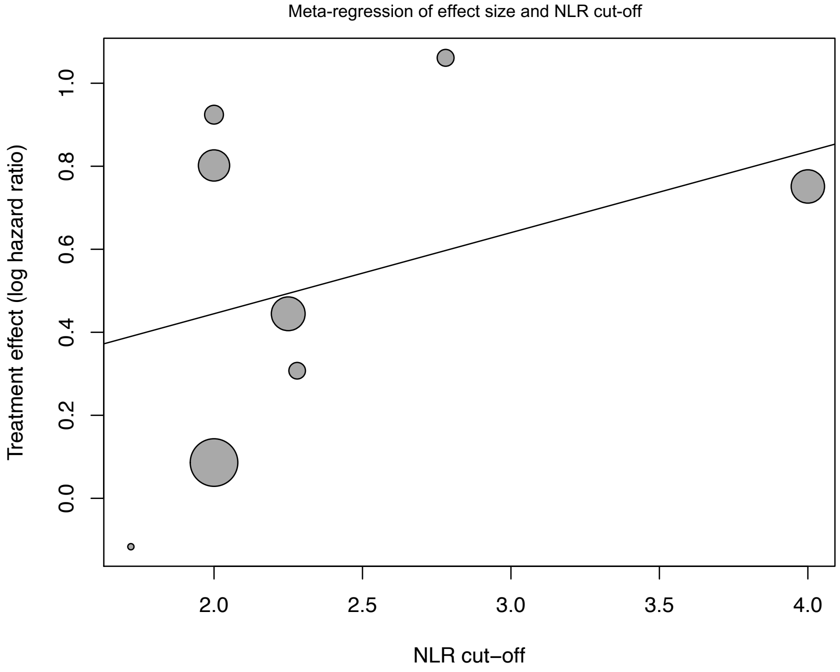

Supplementary Figure 119 - NLR: PFS in Multiple Myeloma

Study	TE	seTE	HR	95%-CI	Weight (fixed)	Weight (random)
Kelkitli 2014	1.25	0.3381	3.50	[1.80; 6.79]	0.5%	20.0%
Shi 2016	0.90	0.2757	2.47	[1.44; 4.24]	0.7%	22.5%
Li 2016	0.07	0.0233	1.07	[1.02; 1.12]	96.4%	30.0%
Romano 2015	0.36	0.1468	1.44	[1.08; 1.92]	2.4%	27.5%
**Pooled HR by Fixed Effects**			**1.09**	**[1.04; 1.14]**	**100.0%**	**--**
**Pooled HR by Random Effects**			**1.78**	**[1.07; 2.96]**	**--**	**100.0%**
**Prediction interval**				**[0.17; 18.19]**		

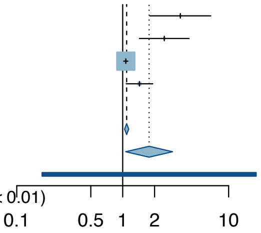

Heterogeneity: I^2 = 88% [72%; 95%], χ^2_3 = 24.94 (p < 0.01)

Tau²= 0.474

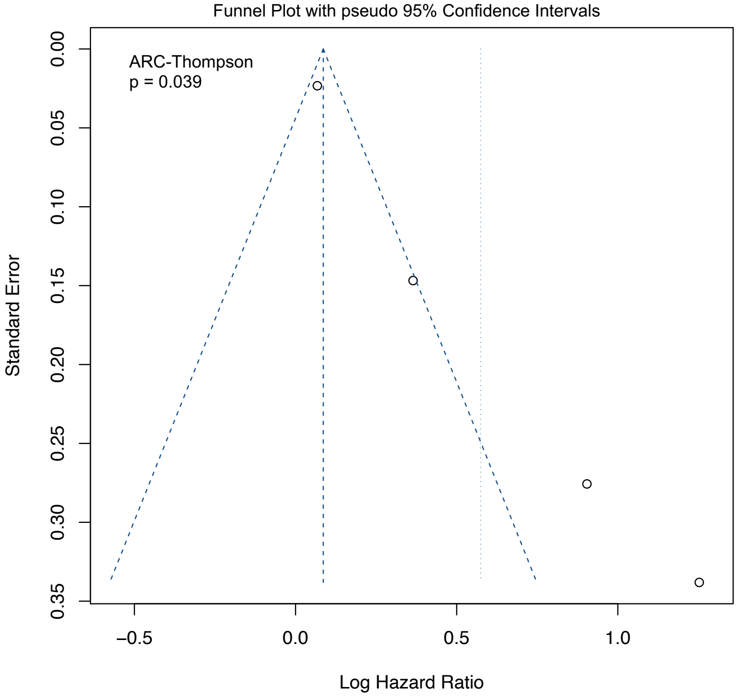

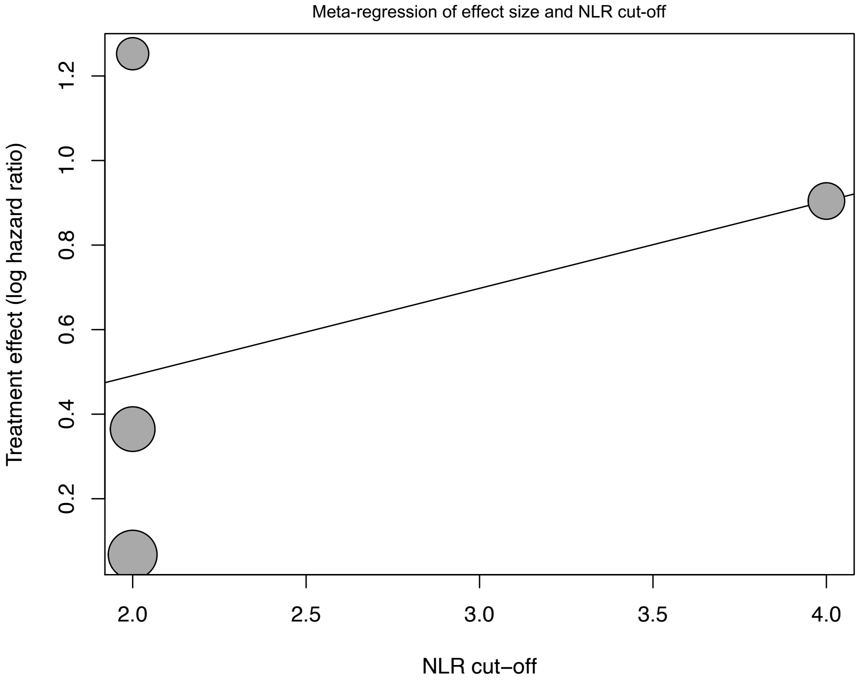

Supplementary Figure 120 - NLR: OS in Nivolumab

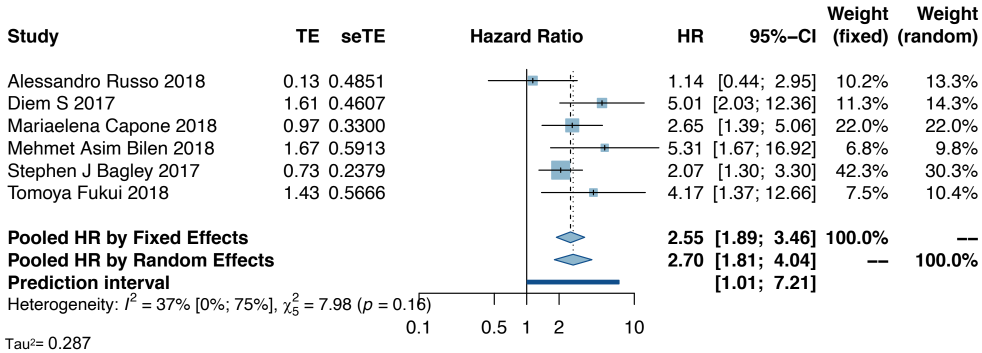

Study	TE	seTE	HR	95%−CI	Weight (fixed)	Weight (random)
Alessandro Russo 2018	0.13	0.4851	1.14	[0.44; 2.95]	10.2%	13.3%
Diem S 2017	1.61	0.4607	5.01	[2.03; 12.36]	11.3%	14.3%
Mariaelena Capone 2018	0.97	0.3300	2.65	[1.39; 5.06]	22.0%	22.0%
Mehmet Asim Bilen 2018	1.67	0.5913	5.31	[1.67; 16.92]	6.8%	9.8%
Stephen J Bagley 2017	0.73	0.2379	2.07	[1.30; 3.30]	42.3%	30.3%
Tomoya Fukui 2018	1.43	0.5666	4.17	[1.37; 12.66]	7.5%	10.4%
**Pooled HR by Fixed Effects**			**2.55**	**[1.89; 3.46]**	**100.0%**	**--**
**Pooled HR by Random Effects**			**2.70**	**[1.81; 4.04]**	**--**	**100.0%**
**Prediction interval**				**[1.01; 7.21]**		

Heterogeneity: I^2 = 37% [0%; 75%], χ^2_5 = 7.98 (p = 0.16)

Tau²= 0.287

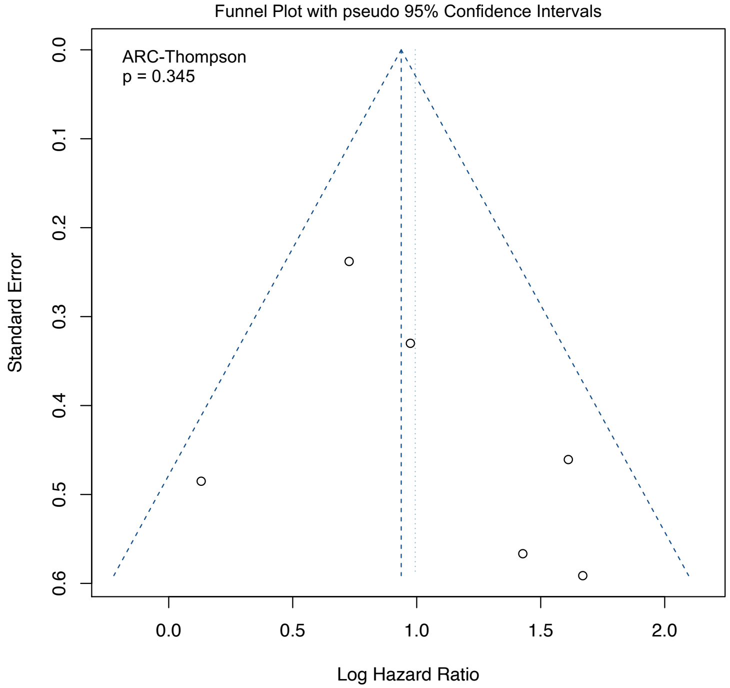

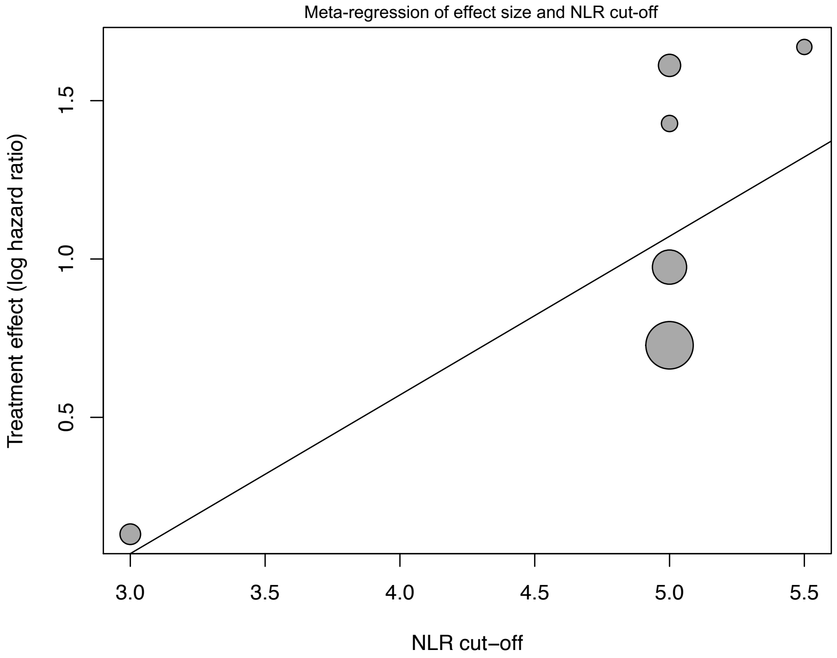

Supplementary Figure 121 - NLR: PFS in Nivolumab

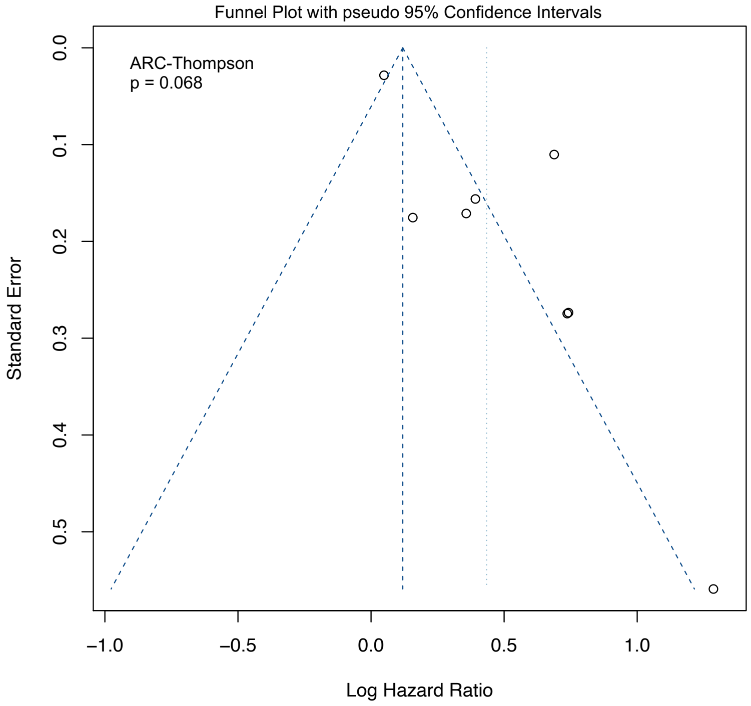

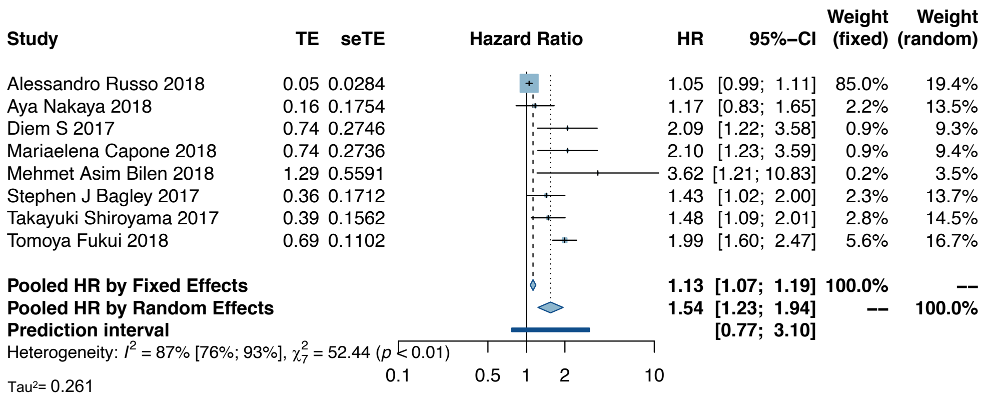

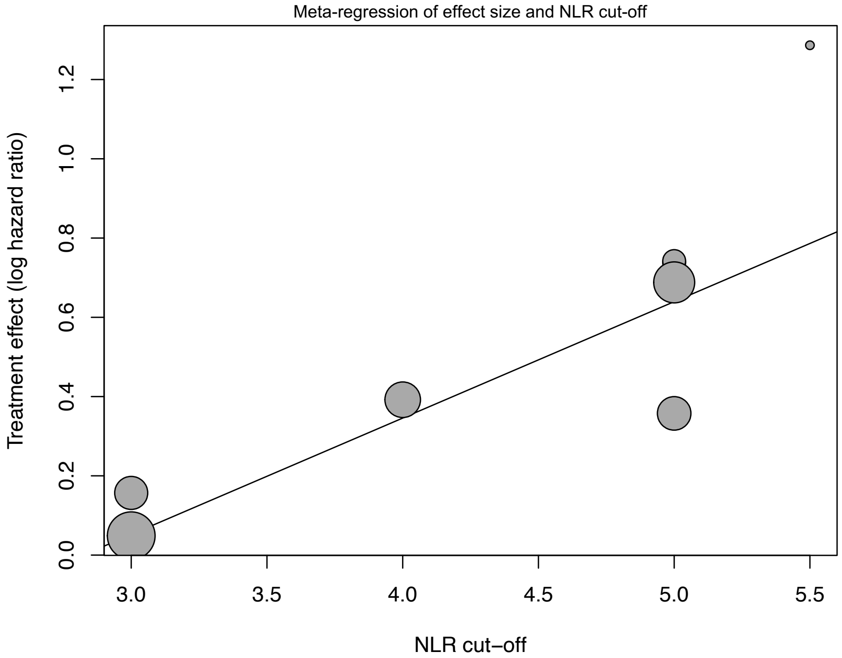

Supplementary Figure 122 - NLR: OS in Ipilimumuab

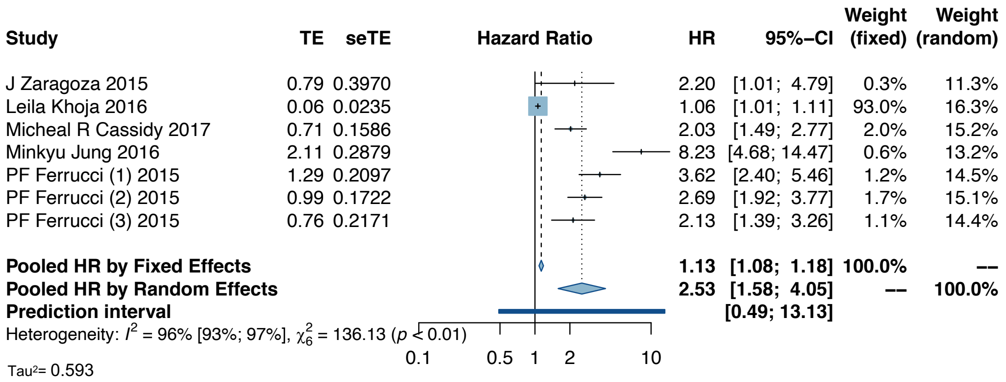

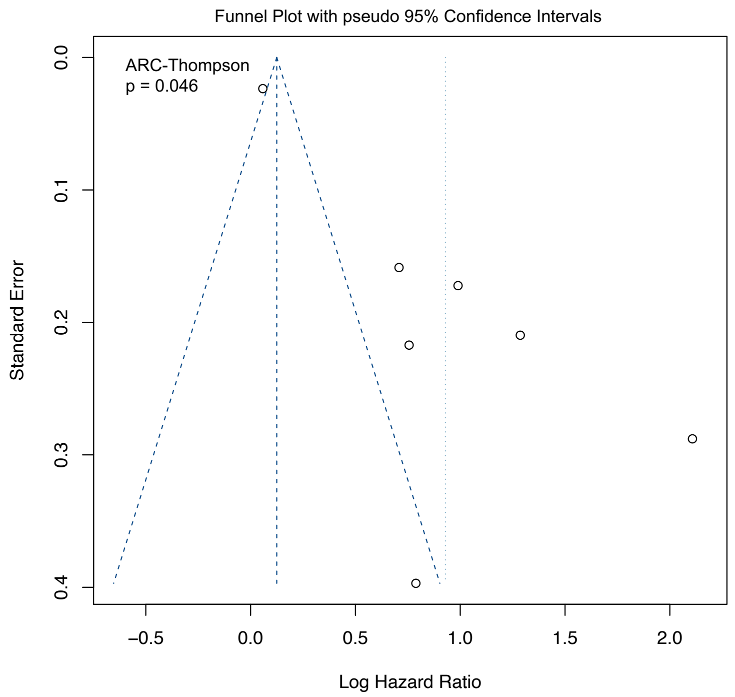

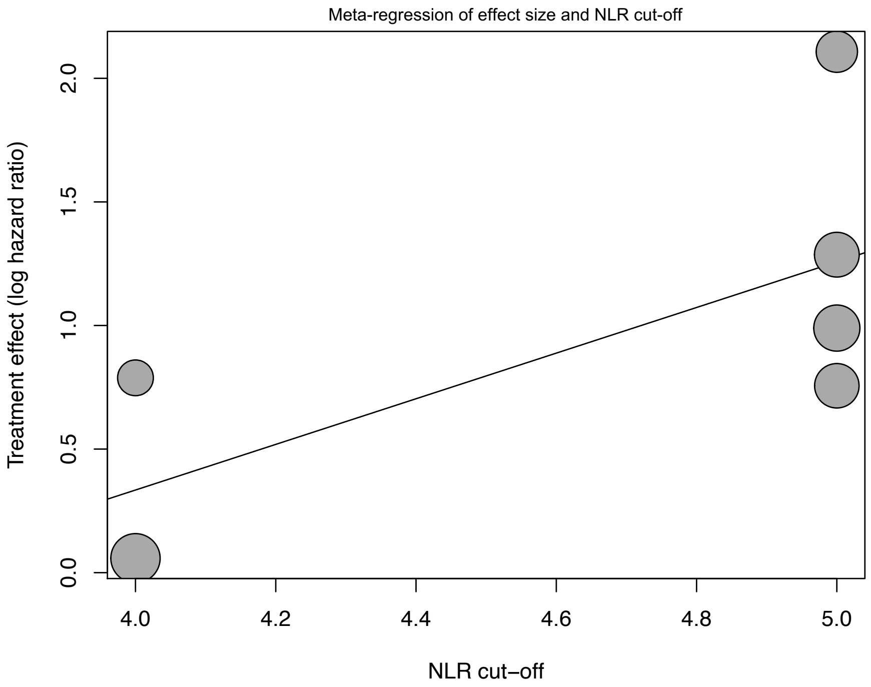

Supplementary Figure 123 - NLR: PFS in Ipilimumuab

Study	TE	seTE	HR	95%-CI	Weight (fixed)	Weight (random)
Micheal R Cassidy 2017	0.01	0.4542	1.01	[0.41; 2.46]	4.0%	16.9%
Minkyu Jung 2016	0.66	0.1112	1.93	[1.55; 2.40]	67.0%	44.2%
PF Ferrucci (1) 2015	1.06	0.1689	2.88	[2.07; 4.01]	29.0%	38.9%
**Pooled HR by Fixed Effects**			**2.11**	**[1.77; 2.52]**	**100.0%**	**--**
**Pooled HR by Random Effects**			**2.02**	**[1.29; 3.18]**	**--**	**100.0%**
**Prediction interval**				**[0.01; 330.06]**		

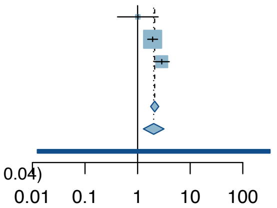

Heterogeneity: I^2 = 70% [0%; 91%], χ^2_2 = 6.67 (p = 0.04)

Tau² = 0.328

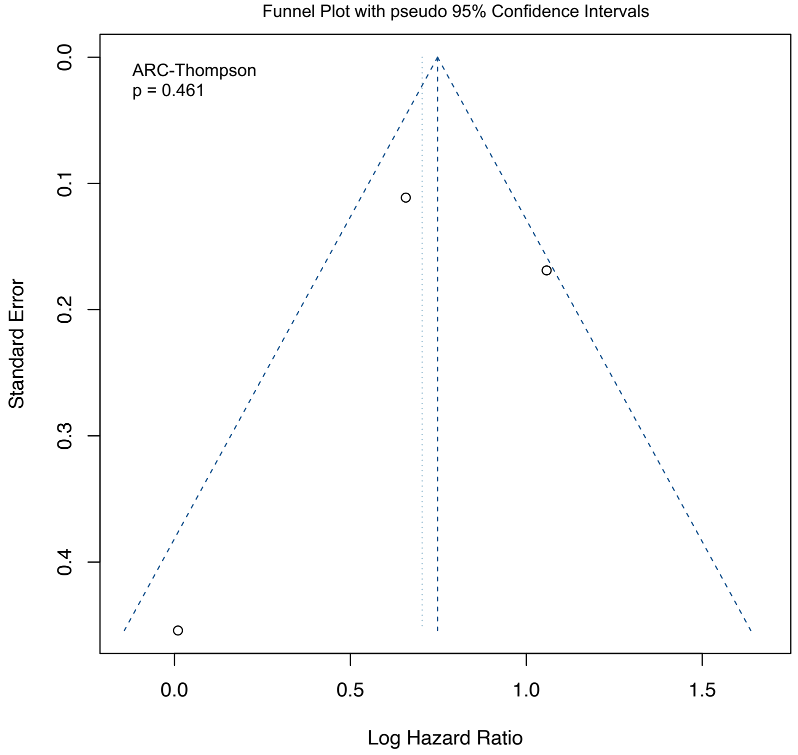

Supplementary Figure 124 - NLR: OS in Oesophageal

Study	TE	seTE	HR	95%-CI	Weight (fixed)	Weight (random)
**Adjustment = Multivariate**						
Miyata 2011	0.26	0.2730	1.30	[0.76; 2.22]	1.0%	3.0%
Sharaiha 2011	0.84	0.2098	2.32	[1.54; 3.50]	1.6%	3.8%
Noble 2013	0.17	0.0451	1.19	[1.09; 1.30]	35.3%	6.0%
Feng 2014	0.29	0.1420	1.34	[1.01; 1.77]	3.6%	4.8%
Yuan 2014	0.94	0.1645	2.55	[1.85; 3.52]	2.7%	4.5%
Yoo 2014	0.75	0.2910	2.12	[1.20; 3.75]	0.8%	2.8%
He 2015	0.25	0.1035	1.29	[1.05; 1.58]	6.7%	5.4%
Han 2015	0.12	0.2023	1.13	[0.76; 1.68]	1.8%	3.9%
Zhangfei 2016	−0.22	0.1339	0.80	[0.62; 1.04]	4.0%	4.9%
Geng 2016	0.17	0.1016	1.18	[0.97; 1.44]	7.0%	5.4%
Zhangpeng 2016	0.17	0.1667	1.19	[0.86; 1.65]	2.6%	4.4%
Miyazaki 2016	0.18	0.1717	1.20	[0.86; 1.68]	2.4%	4.4%
Zhangfan 2016	0.25	0.1625	1.28	[0.93; 1.76]	2.7%	4.5%
Kosumi 2016	0.61	0.2374	1.84	[1.16; 2.93]	1.3%	3.4%
Xiao 2016	0.71	0.2417	2.03	[1.26; 3.26]	1.2%	3.4%
Wan 2016	0.84	0.3265	2.32	[1.22; 4.40]	0.7%	2.5%
Ji 2016	1.25	0.5556	3.50	[1.18; 10.40]	0.2%	1.2%
He 2016	0.31	0.1505	1.37	[1.02; 1.84]	3.2%	4.7%
Toyokawa 2016	0.17	0.3295	1.19	[0.62; 2.27]	0.7%	2.4%
Zhou 2017	0.62	0.1083	1.86	[1.50; 2.30]	6.1%	5.3%
Nakamura 2017	1.28	0.4272	3.61	[1.56; 8.34]	0.4%	1.7%
Gao 2017	0.64	0.0957	1.89	[1.57; 2.28]	7.8%	5.5%
Hirahara 2017	0.49	0.2687	1.63	[0.96; 2.76]	1.0%	3.1%
Yu 2018	−0.45	0.3770	0.64	[0.31; 1.34]	0.5%	2.1%
**Pooled HR by Fixed Effects**			**1.35**	**[1.28; 1.43]**	**95.2%**	**--**
**Pooled HR by Random Effects**			**1.48**	**[1.29; 1.70]**	**--**	**93.1%**
Heterogeneity: $I^2 = 75\%$ [63%; 83%], $\chi^2_{23} = 92.22$ ($p < 0.01$)						
**Adjustment = Univariate**						
Xu 2015	0.41	0.1286	1.50	[1.17; 1.93]	4.3%	5.0%
Kunizaki 2018	1.16	0.4076	3.18	[1.43; 7.07]	0.4%	1.9%
**Pooled HR by Fixed Effects**			**1.61**	**[1.26; 2.04]**	**4.8%**	**--**
**Pooled HR by Random Effects**			**1.98**	**[0.97; 4.02]**	**--**	**6.9%**
Heterogeneity: $I^2 = 68\%$ [0%; 93%], $\chi^2_{1} = 3.09$ ($p = 0.08$)						
**Pooled HR by Fixed Effects**			**1.36**	**[1.30; 1.44]**	**100.0%**	**--**
**Pooled HR by Random Effects**			**1.50**	**[1.32; 1.71]**	**--**	**100.0%**
**Prediction interval**				**[0.85; 2.65]**		

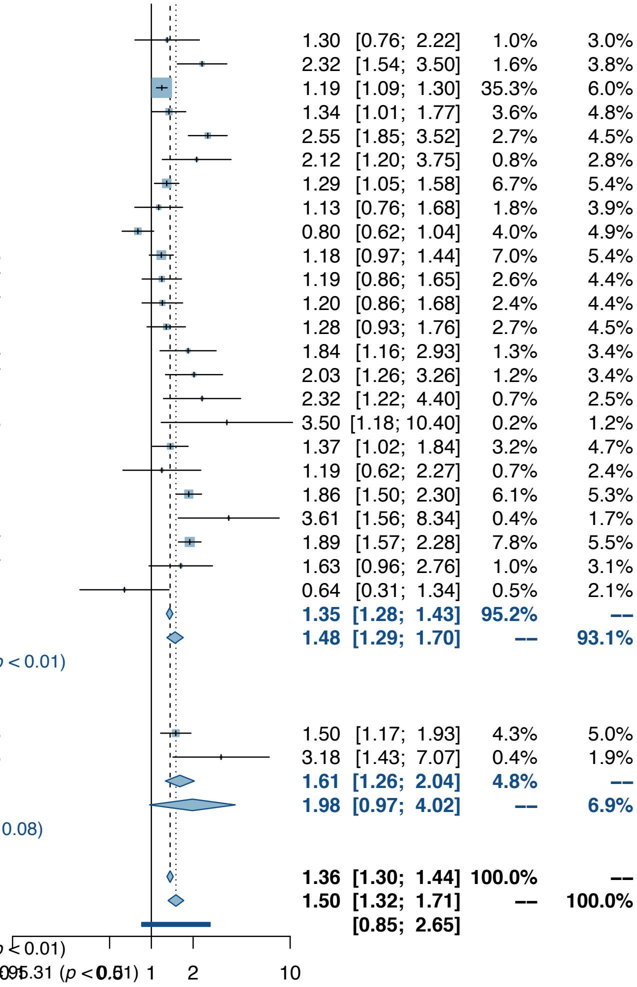

Heterogeneity: $I^2 = 74\%$ [62%; 82%], $\chi^2_{25} = 97.15$ ($p < 0.01$)
Residual heterogeneity: $I^2 = 75\%$ [63%; 83%], $\chi^2_{24} = 95.31$ ($p < 0.01$)

Tau²= 0.267

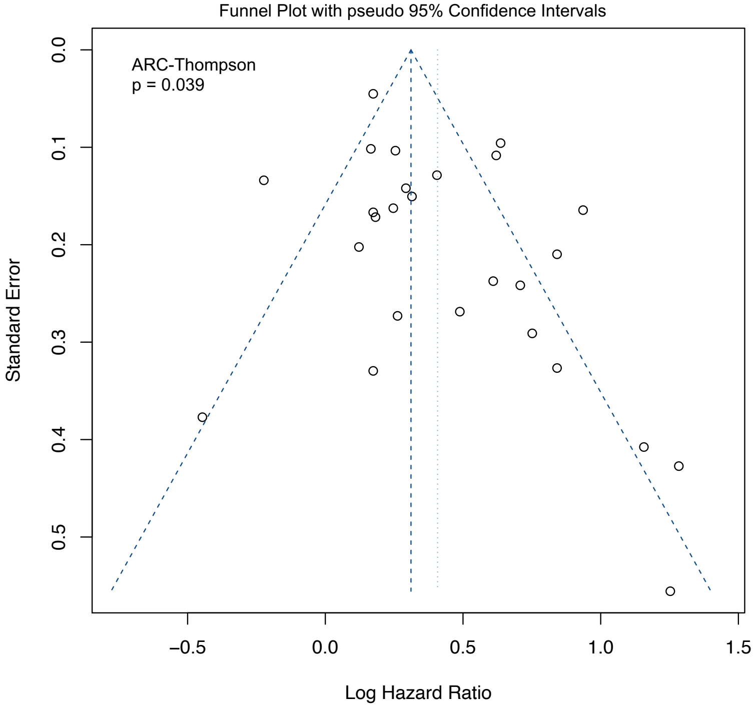

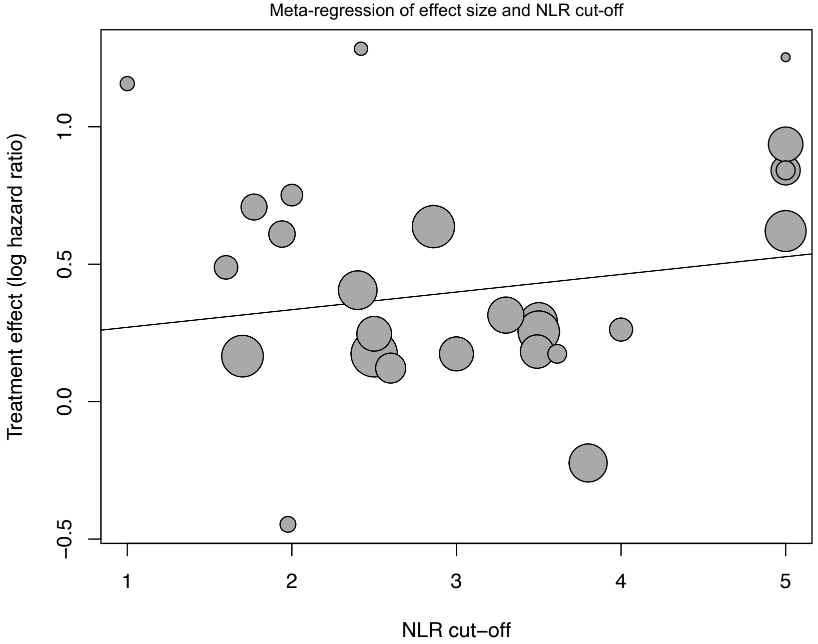

Supplementary Figure 125 - NLR: OS in Oesophageal (Surgery)

Study	TE	seTE	HR	95%-CI	Weight (fixed)	Weight (random)
**Adjustment = Multivariate**						
Miyata 2011	0.26	0.2730	1.30	[0.76; 2.22]	1.2%	3.9%
Sharaiha 2011	0.84	0.2098	2.32	[1.54; 3.50]	2.0%	4.8%
Noble 2013	0.17	0.0451	1.19	[1.09; 1.30]	42.5%	7.2%
Feng 2014	0.29	0.1420	1.34	[1.01; 1.77]	4.3%	6.0%
Yuan 2014	0.94	0.1645	2.55	[1.85; 3.52]	3.2%	5.6%
Han 2015	0.12	0.2023	1.13	[0.76; 1.68]	2.1%	5.0%
Zhangfei 2016	−0.22	0.1339	0.80	[0.62; 1.04]	4.8%	6.1%
Geng 2016	0.17	0.1016	1.18	[0.97; 1.44]	8.4%	6.6%
Miyazaki 2016	0.18	0.1717	1.20	[0.86; 1.68]	2.9%	5.5%
Zhangfan 2016	0.25	0.1625	1.28	[0.93; 1.76]	3.3%	5.6%
Kosumi 2016	0.61	0.2374	1.84	[1.16; 2.93]	1.5%	4.4%
Xiao 2016	0.71	0.2417	2.03	[1.26; 3.26]	1.5%	4.4%
Ji 2016	1.25	0.5556	3.50	[1.18; 10.40]	0.3%	1.6%
He 2016	0.31	0.1505	1.37	[1.02; 1.84]	3.8%	5.8%
Toyokawa 2016	0.17	0.3295	1.19	[0.62; 2.27]	0.8%	3.2%
Nakamura 2017	1.28	0.4272	3.61	[1.56; 8.34]	0.5%	2.3%
Gao 2017	0.64	0.0957	1.89	[1.57; 2.28]	9.4%	6.7%
Hirahara 2017	0.49	0.2687	1.63	[0.96; 2.76]	1.2%	4.0%
Yu 2018	−0.45	0.3770	0.64	[0.31; 1.34]	0.6%	2.7%
**Pooled HR by Fixed Effects**			**1.32**	**[1.24; 1.40]**	**94.3%**	**--**
**Pooled HR by Random Effects**			**1.45**	**[1.24; 1.71]**	**--**	**91.3%**
Heterogeneity: $I^2 = 77\%$ [64%; 85%], $\chi^2_{18} = 76.98$ ($p < 0.01$)						
**Adjustment = Univariate**						
Xu 2015	0.41	0.1286	1.50	[1.17; 1.93]	5.2%	6.2%
Kunizaki 2018	1.16	0.4076	3.18	[1.43; 7.07]	0.5%	2.5%
**Pooled HR by Fixed Effects**			**1.61**	**[1.26; 2.04]**	**5.7%**	**--**
**Pooled HR by Random Effects**			**1.98**	**[0.97; 4.02]**	**--**	**8.7%**
Heterogeneity: $I^2 = 68\%$ [0%; 93%], $\chi^2_1 = 3.09$ ($p = 0.08$)						
**Pooled HR by Fixed Effects**			**1.33**	**[1.26; 1.41]**	**100.0%**	**--**
**Pooled HR by Random Effects**			**1.48**	**[1.27; 1.73]**	**--**	**100.0%**
**Prediction interval**				**[0.79; 2.78]**		

Heterogeneity: $I^2 = 76\%$ [63%; 84%], $\chi^2_{20} = 82.49$ ($p < 0.01$)

Residual heterogeneity: $I^2 = 76\%$ [64%; 85%], $\chi^2_{19} = 80.07$ ($p < 0.01$)

Hazard Ratio axis: 0.1, 0.5, 1, 2, 10

Tau²= 0.289

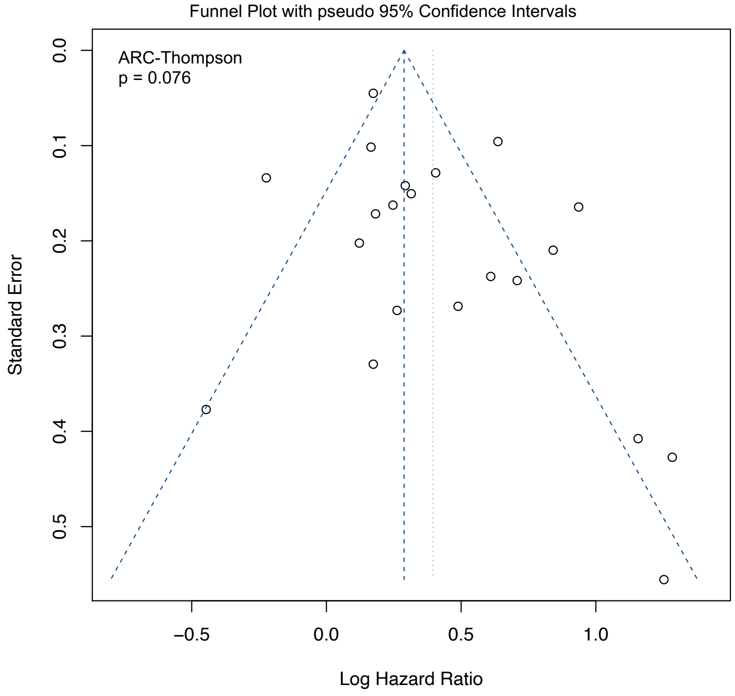

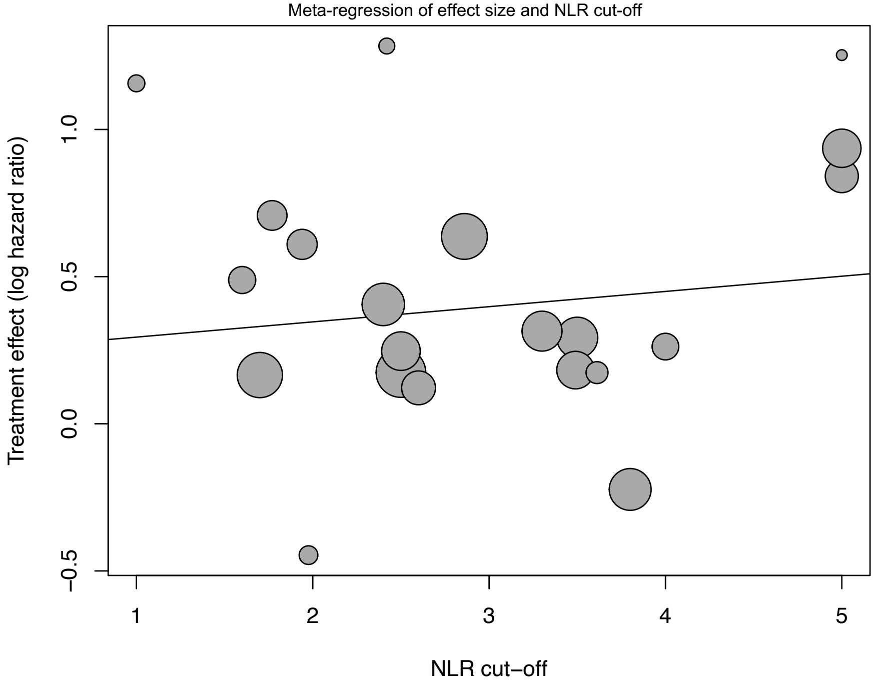

Supplementary Figure 126 - NLR: CSS in Oesophageal

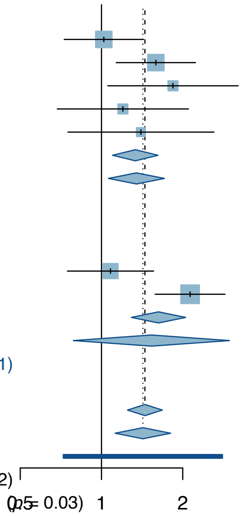

Study	TE	seTE	HR	95%–CI	Weight (fixed)	Weight (random)
**Adjustment = Multivariate**						
Liu 2015	0.02	0.1724	1.02	[0.73; 1.43]	19.4%	17.2%
Duan 2015	0.46	0.1726	1.59	[1.13; 2.23]	19.4%	17.2%
Kosumi 2016	0.61	0.2839	1.84	[1.05; 3.21]	7.2%	10.8%
Xie 2016	0.18	0.2855	1.20	[0.69; 2.10]	7.1%	10.7%
Hirahara 2017	0.34	0.3178	1.40	[0.75; 2.61]	5.7%	9.4%
**Pooled HR by Fixed Effects**			**1.33**	**[1.10; 1.62]**	**58.7%**	**--**
**Pooled HR by Random Effects**			**1.35**	**[1.06; 1.71]**	**--**	**65.2%**
Heterogeneity: $I^2 = 18\%$ [0%; 83%], $\chi^2_4 = 4.9$ ($p = 0.30$)						
**Adjustment = Univariate**						
Dutta 2011	0.08	0.1876	1.08	[0.75; 1.56]	16.4%	16.2%
Zhao 2017	0.76	0.1521	2.13	[1.58; 2.87]	24.9%	18.6%
**Pooled HR by Fixed Effects**			**1.63**	**[1.29; 2.05]**	**41.3%**	**--**
**Pooled HR by Random Effects**			**1.53**	**[0.79; 2.98]**	**--**	**34.8%**
Heterogeneity: $I^2 = 87\%$ [51%; 97%], $\chi^2_1 = 7.91$ ($p < 0.01$)						
**Pooled HR by Fixed Effects**			**1.45**	**[1.25; 1.68]**	**100.0%**	**--**
**Pooled HR by Random Effects**			**1.42**	**[1.12; 1.80]**	**--**	**100.0%**
**Prediction interval**				**[0.72; 2.81]**		
Heterogeneity: $I^2 = 59\%$ [4%; 82%], $\chi^2_6 = 14.46$ ($p = 0.02$)						
Residual heterogeneity: $I^2 = 61\%$ [5%; 84%], $\chi^2_5 = 12.81$ ($p = 0.03$)						

Tau²= 0.235

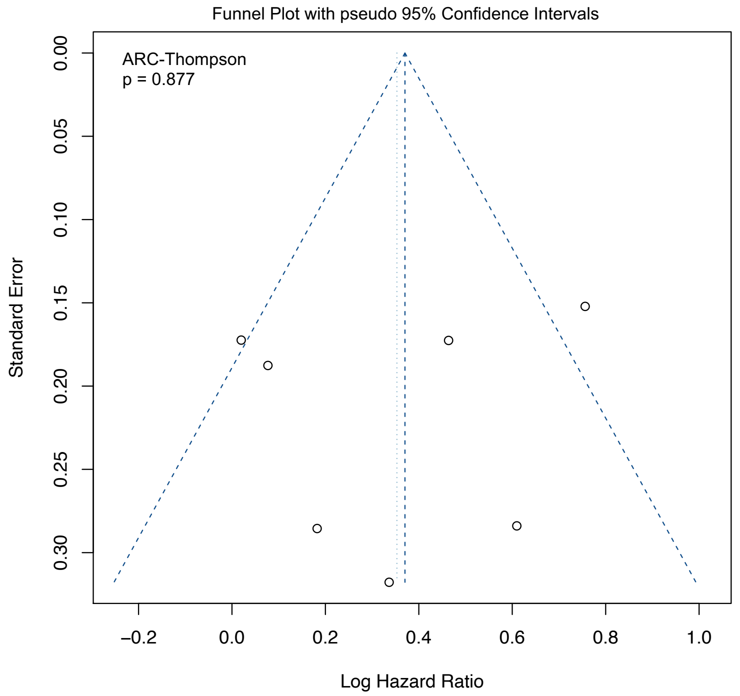

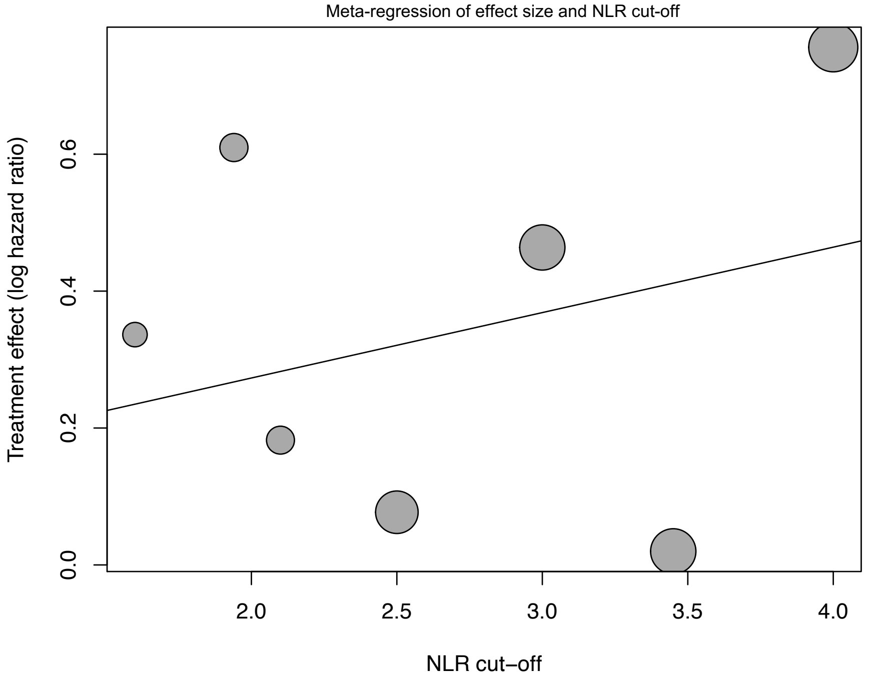

Supplementary Figure 127 - NLR: RFS in Oesophageal

Study	TE	seTE	HR	95%–CI	Weight (fixed)	Weight (random)
Duan 2015	0.42	0.1721	1.52	[1.08; 2.13]	55.3%	46.7%
Toyokawa 2016	0.09	0.3346	1.09	[0.57; 2.10]	14.6%	20.0%
Xiao 2016	0.80	0.2337	2.22	[1.40; 3.51]	30.0%	33.3%
**Pooled HR by Fixed Effects**			**1.62**	**[1.26; 2.08]**	**100.0%**	**--**
**Pooled HR by Random Effects**			**1.61**	**[1.16; 2.25]**	**--**	**100.0%**
**Prediction interval**				**[0.07; 37.24]**		

Heterogeneity: $I^2 = 40\%$ [0%; 82%], $\chi^2_2 = 3.36$ ($p = 0.19$)

Tau²= 0.179

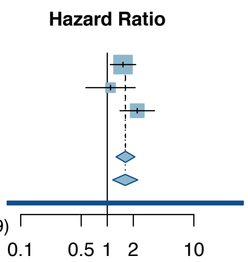

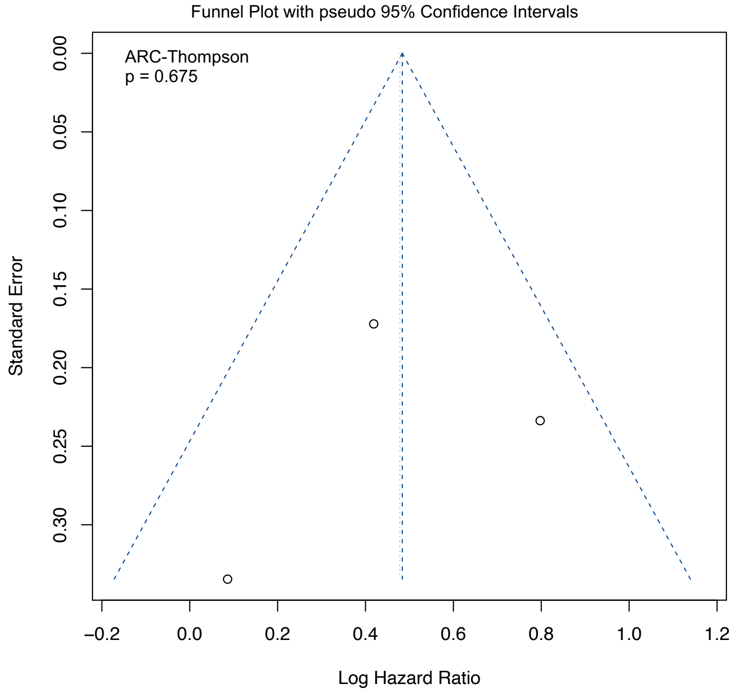

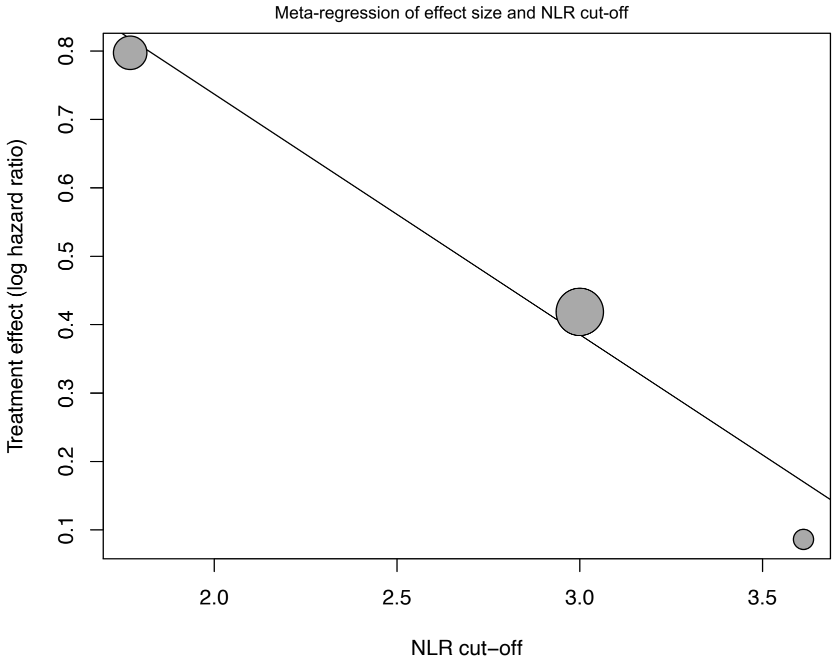

Supplementary Figure 128 - NLR: OS in Oesophageal (DCRT)

Study	TE	seTE	Hazard Ratio	HR	95%–CI	Weight (fixed)	Weight (random)
Grenader T 2016	0.51	0.0712		1.67	[1.45; 1.92]	67.9%	67.9%
Yoo EJ 2014	0.75	0.2923		2.12	[1.20; 3.76]	4.0%	4.0%
Zhou XL 2017	0.62	0.1105		1.86	[1.50; 2.31]	28.1%	28.1%
**Pooled HR by Fixed Effects**				**1.74**	**[1.55; 1.95]**	**100.0%**	**--**
**Pooled HR by Random Effects**				**1.74**	**[1.55; 1.95]**	**--**	**100.0%**
**Prediction interval**					**[0.83; 3.66]**		

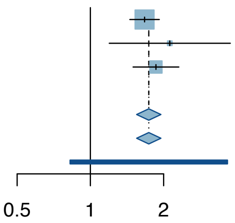

Heterogeneity: $I^2 = 0\%$ [0%; 82%], $\chi^2_2 = 1.15$ ($p = 0.56$)

Tau²= 0

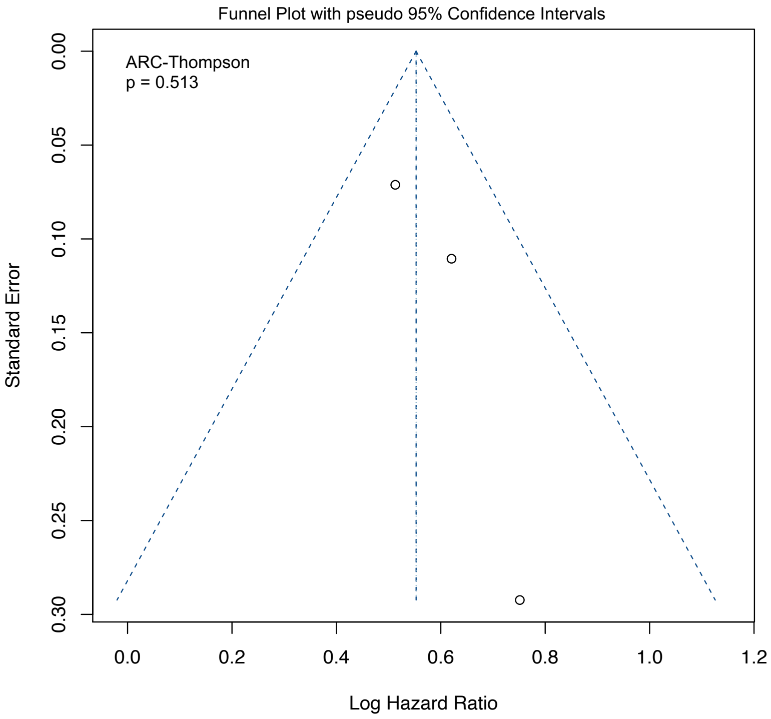

Supplementary Figure 129 - NLR: OS in Oesophageal (Neo+Surgery)

Study	TE	seTE	HR	95%–CI	Weight (fixed)	Weight (random)
Miyata H 2011	0.49	0.2343	1.63	[1.03; 2.58]	9.8%	9.8%
Noble F 2014	0.18	0.1070	1.20	[0.97; 1.48]	47.2%	47.2%
Shao Y 2015	0.23	0.1122	1.26	[1.01; 1.57]	42.9%	42.9%
**Pooled HR by Fixed Effects**			**1.26**	**[1.09; 1.46]**	**100.0%**	**--**
**Pooled HR by Random Effects**			**1.26**	**[1.09; 1.46]**	**--**	**100.0%**
**Prediction interval**				**[0.50; 3.21]**		

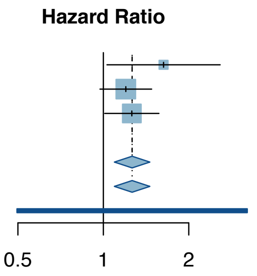

Heterogeneity: $I^2 = 0\%$ [0%; 85%], $\chi^2_2 = 1.41$ ($p = 0.49$)

Tau²= 0.001

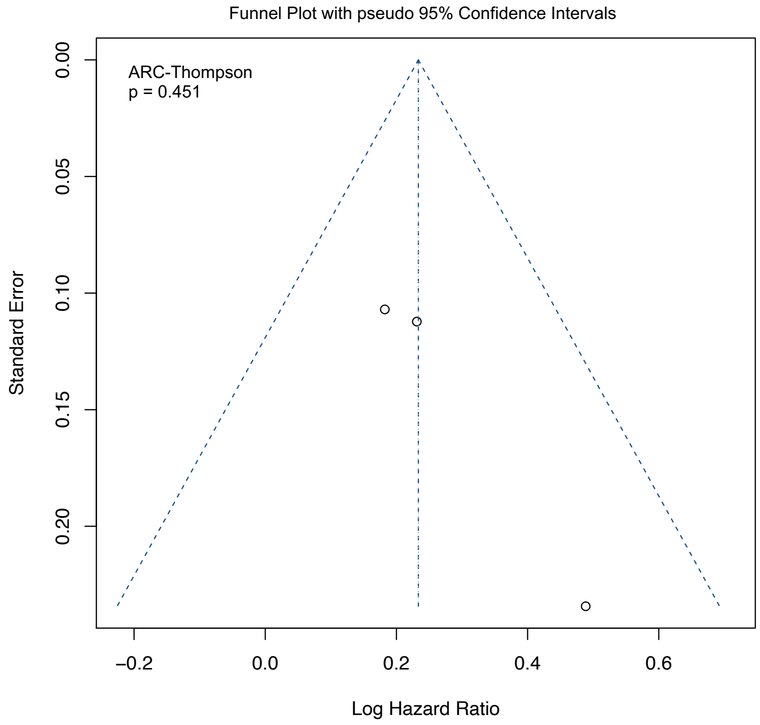

Supplementary Figure 130 - NLR: OS in Oesophageal (No Surgery)

Study	TE	seTE	HR	95%-CI	Weight (fixed)	Weight (random)
Yoo 2014	0.75	0.2910	2.12	[1.20; 3.75]	8.3%	17.2%
Zhangpeng 2016	0.17	0.1667	1.19	[0.86; 1.65]	25.3%	30.0%
Wan 2016	0.84	0.3265	2.32	[1.22; 4.40]	6.6%	14.8%
Zhou 2017	0.62	0.1083	1.86	[1.50; 2.30]	59.9%	38.0%
**Pooled HR by Fixed Effects**			**1.70**	**[1.45; 2.01]**	**100.0%**	**--**
**Pooled HR by Random Effects**			**1.72**	**[1.28; 2.31]**	**--**	**100.0%**
**Prediction interval**				**[0.54; 5.42]**		

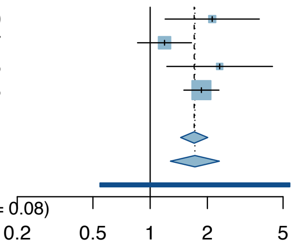

Heterogeneity: $I^2 = 56\%$ [0%; 85%], $\chi^2_3 = 6.75$ ($p = 0.08$)

Tau²= 0.220

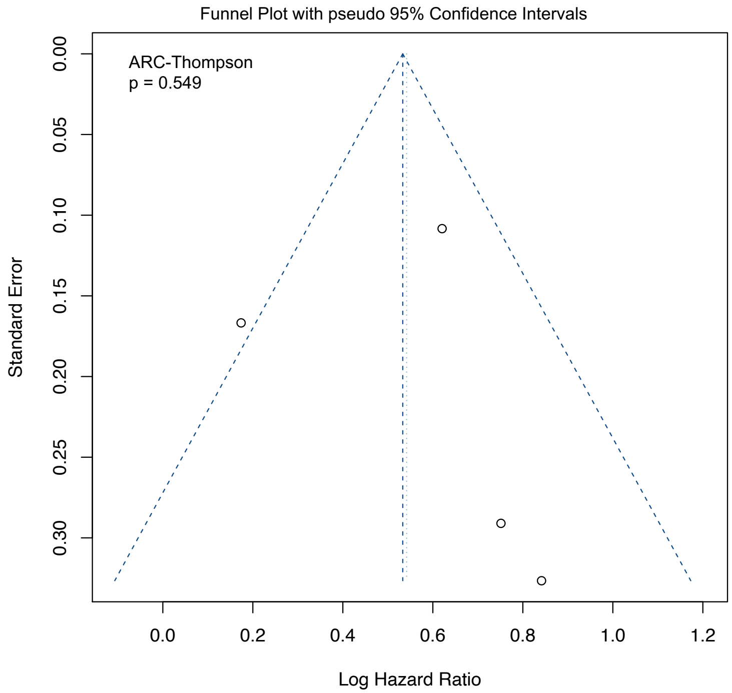

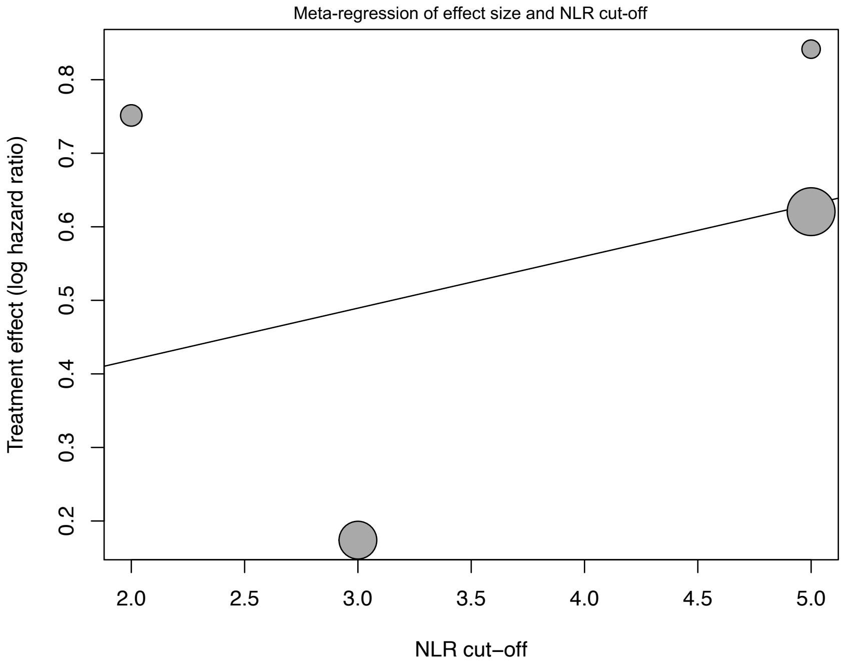

Supplementary Figure 131 - NLR: OS in Oesophageal SCC

Study	TE	seTE	HR	95%-CI	Weight (fixed)	Weight (random)
**Adjustment = Multivariate**						
Feng 2014	0.29	0.1420	1.34	[1.01; 1.77]	6.2%	6.5%
He 2015	0.25	0.1035	1.29	[1.05; 1.58]	11.7%	7.6%
Han 2015	0.12	0.2023	1.13	[0.76; 1.68]	3.0%	5.0%
Zhangfei 2016	−0.22	0.1339	0.80	[0.62; 1.04]	7.0%	6.7%
Geng 2016	0.17	0.1016	1.18	[0.97; 1.44]	12.1%	7.6%
Zhangpeng 2016	0.17	0.1667	1.19	[0.86; 1.65]	4.5%	5.9%
Miyazaki 2016	0.18	0.1717	1.20	[0.86; 1.68]	4.2%	5.7%
Zhangfan 2016	0.25	0.1625	1.28	[0.93; 1.76]	4.7%	6.0%
Kosumi 2016	0.61	0.2374	1.84	[1.16; 2.93]	2.2%	4.2%
Xiao 2016	0.71	0.2417	2.03	[1.26; 3.26]	2.1%	4.2%
Ji 2016	1.25	0.5556	3.50	[1.18; 10.40]	0.4%	1.2%
He 2016	0.31	0.1505	1.37	[1.02; 1.84]	5.5%	6.3%
Toyokawa 2016	0.17	0.3295	1.19	[0.62; 2.27]	1.1%	2.8%
Zhou 2017	0.62	0.1083	1.86	[1.50; 2.30]	10.6%	7.5%
Gao 2017	0.64	0.0957	1.89	[1.57; 2.28]	13.6%	7.8%
Hirahara 2017	0.49	0.2687	1.63	[0.96; 2.76]	1.7%	3.7%
Yu 2018	−0.45	0.3770	0.64	[0.31; 1.34]	0.9%	2.3%
**Pooled HR by Fixed Effects**			**1.38**	**[1.28; 1.48]**	**91.7%**	**--**
**Pooled HR by Random Effects**			**1.35**	**[1.18; 1.55]**	**--**	**91.0%**
Heterogeneity: $I^2 = 69\%$ [49%; 81%], $\chi^2_{16} = 52.03$ ($p < 0.01$)						
**Adjustment = Univariate**						
Xu 2015	0.41	0.1286	1.50	[1.17; 1.93]	7.5%	6.9%
Kunizaki 2018	1.16	0.4076	3.18	[1.43; 7.07]	0.8%	2.1%
**Pooled HR by Fixed Effects**			**1.61**	**[1.26; 2.04]**	**8.3%**	**--**
**Pooled HR by Random Effects**			**1.98**	**[0.97; 4.02]**	**--**	**9.0%**
Heterogeneity: $I^2 = 68\%$ [0%; 93%], $\chi^2_{1} = 3.09$ ($p = 0.08$)						
**Pooled HR by Fixed Effects**			**1.39**	**[1.30; 1.49]**	**100.0%**	**--**
**Pooled HR by Random Effects**			**1.39**	**[1.22; 1.58]**	**--**	**100.0%**
**Prediction interval**				**[0.86; 2.24]**		

Heterogeneity: $I^2 = 68\%$ [49%; 80%], $\chi^2_{18} = 56.55$ ($p < 0.01$)

Residual heterogeneity: $I^2 = 69\%$ [50%; 81%], $\chi^2_{17} = 55.12$ ($p < 0.01$)

Hazard Ratio axis: 0.1 0.5 1 2 10

Tau²= 0.217

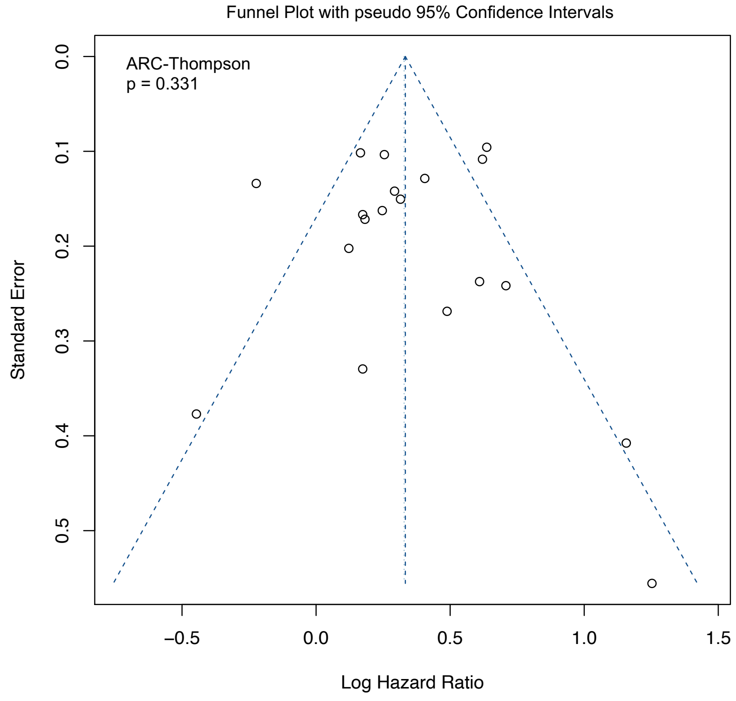

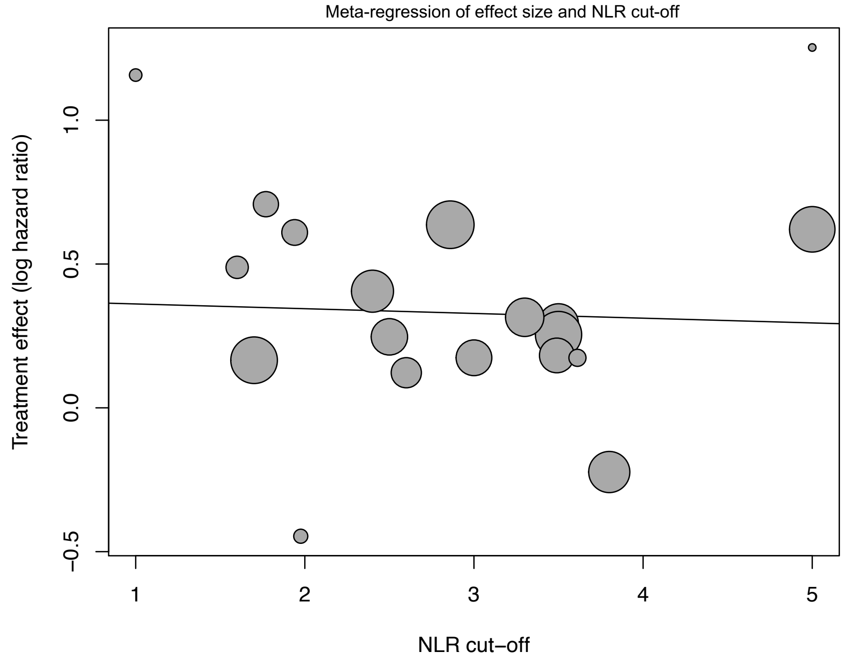

Supplementary Figure 132 - NLR: OS in Oesophageal (Surgery+/-Chemo)

Study	TE	seTE	HR	95%-CI	Weight (fixed)	Weight (random)
Arigami T 2015	1.35	0.2078	3.86	[2.57; 5.80]	7.2%	7.9%
Duan H 2015	0.46	0.1749	1.59	[1.13; 2.24]	10.2%	8.6%
Feng JF 2014	0.29	0.1420	1.34	[1.01; 1.77]	15.5%	9.3%
Han LH 2015	0.12	0.2054	1.13	[0.76; 1.69]	7.4%	7.9%
He YF 2017	0.31	0.1505	1.37	[1.02; 1.84]	13.8%	9.1%
Hirahara N 2015	0.15	0.3242	1.16	[0.61; 2.19]	3.0%	5.6%
Ji WH 2016	1.25	0.5547	3.50	[1.18; 10.38]	1.0%	2.9%
Jung J 2016	0.71	0.2069	2.04	[1.36; 3.06]	7.3%	7.9%
Kosumi K 2016	0.61	0.2304	1.84	[1.17; 2.89]	5.9%	7.4%
Nakamura K 2017	1.28	0.4345	3.61	[1.54; 8.46]	1.7%	4.0%
Sharaiha R 2011	0.84	0.2127	2.32	[1.53; 3.52]	6.9%	7.7%
Toyokawa T 2016	0.17	0.3295	1.19	[0.62; 2.27]	2.9%	5.5%
Xie X 2016	0.18	0.1837	1.20	[0.84; 1.72]	9.3%	8.4%
Yuan D 2014	0.94	0.1981	2.55	[1.73; 3.76]	8.0%	8.1%
**Pooled HR by Fixed Effects**			**1.69**	**[1.51; 1.88]**	**100.0%**	**--**
**Pooled HR by Random Effects**			**1.77**	**[1.43; 2.20]**	**--**	**100.0%**
**Prediction interval**				**[0.83; 3.78]**		

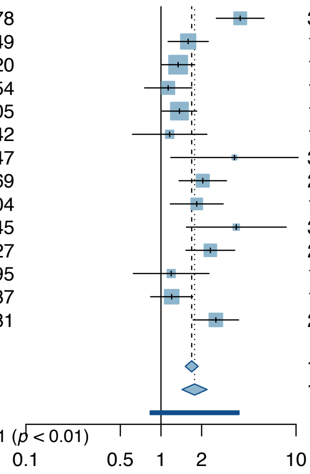

Heterogeneity: $I^2 = 69\%$ [47%; 82%], $\chi^2_{13} = 42.61$ ($p < 0.01$)

Tau²= 0.330

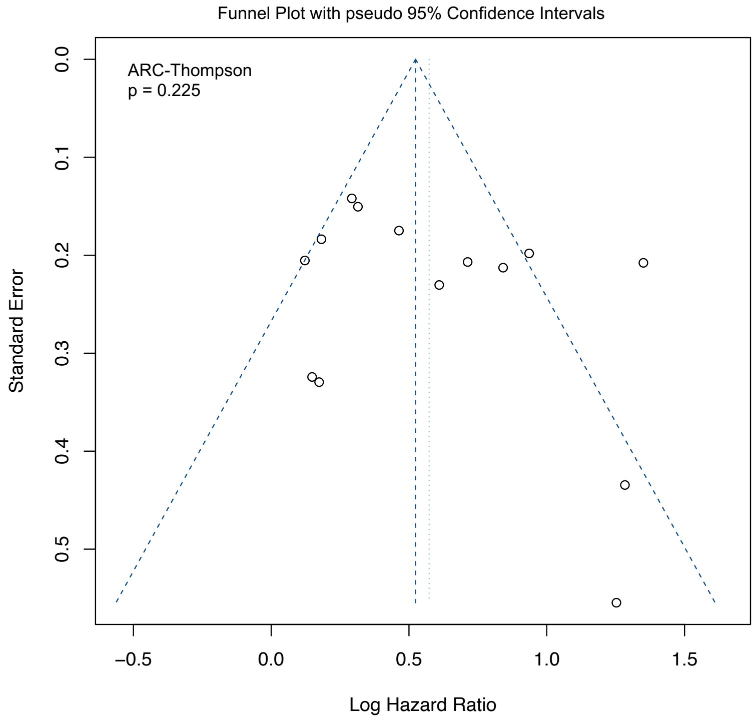

Supplementary Figure 133 - NLR: DFS in Oesophageal

Study	TE	seTE	HR	95%-CI	Weight (fixed)	Weight (random)
Sharaiha 2011	0.82	0.2318	2.26	[1.43; 3.56]	3.0%	9.6%
Noble 2013	0.07	0.0542	1.07	[0.96; 1.19]	55.5%	12.7%
Yuan 2014	1.01	0.1435	2.74	[2.07; 3.63]	7.9%	11.4%
Han 2015	0.21	0.1971	1.23	[0.84; 1.81]	4.2%	10.3%
Zhangfei 2016	−0.13	0.1230	0.88	[0.69; 1.12]	10.8%	11.7%
Zhangfan 2016	0.22	0.1599	1.25	[0.91; 1.71]	6.4%	11.1%
Wan 2016	0.87	0.3273	2.38	[1.25; 4.52]	1.5%	7.6%
Ikeguchi 2016	0.89	0.4065	2.43	[1.10; 5.39]	1.0%	6.2%
He 2016	0.25	0.1438	1.29	[0.97; 1.71]	7.9%	11.4%
Nakamura 2017	1.02	0.3062	2.76	[1.51; 5.03]	1.7%	8.0%
**Pooled HR by Fixed Effects**			**1.24**	**[1.14; 1.34]**	**100.0%**	**--**
**Pooled HR by Random Effects**			**1.59**	**[1.21; 2.10]**	**--**	**100.0%**
**Prediction interval**				**[0.61; 4.17]**		

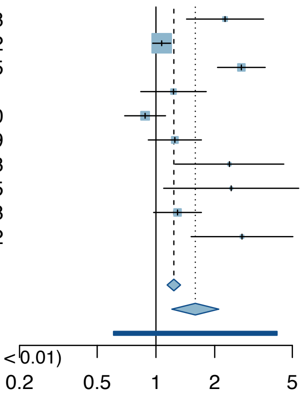

Heterogeneity: I^2 = 86% [77%; 92%], χ^2_9 = 65.99 (p < 0.01)

Tau^2= 0.394

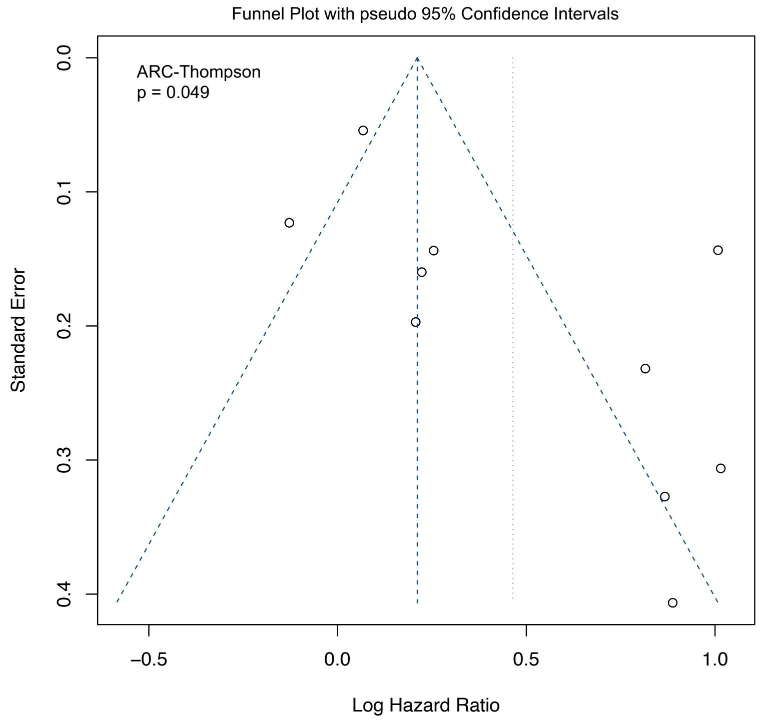

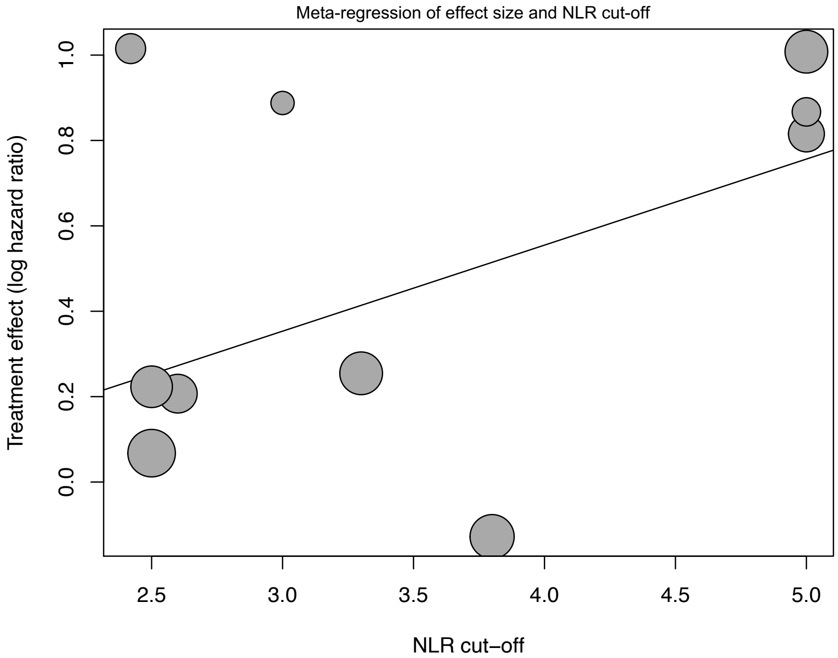

Supplementary Figure 134 - NLR: PFS in Oesophageal

Study	TE	seTE	HR	95%-CI	Weight (fixed)	Weight (random)
Yoo 2014	0.59	0.2741	1.80	[1.05; 3.08]	12.8%	13.9%
Zhangpeng 2016	0.17	0.1617	1.18	[0.86; 1.62]	36.9%	36.8%
Ji 2016	0.52	0.4928	1.69	[0.64; 4.44]	4.0%	4.4%
Zhou 2017	0.43	0.1443	1.53	[1.15; 2.03]	46.3%	44.9%
**Pooled HR by Fixed Effects**			**1.43**	**[1.18; 1.73]**	**100.0%**	**--**
**Pooled HR by Random Effects**			**1.43**	**[1.16; 1.75]**	**--**	**100.0%**
**Prediction interval**				**[0.85; 2.39]**		

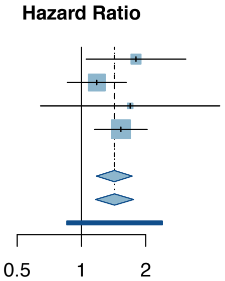

Heterogeneity: $I^2 = 0\%$ [0%; 81%], $\chi^2_3 = 2.45$ ($p = 0.48$)

Tau²= 0.058

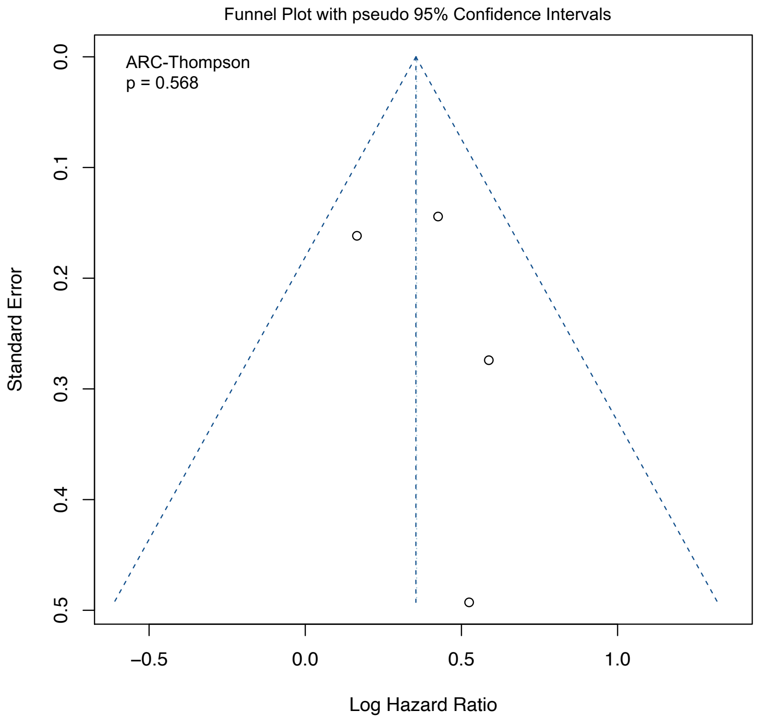

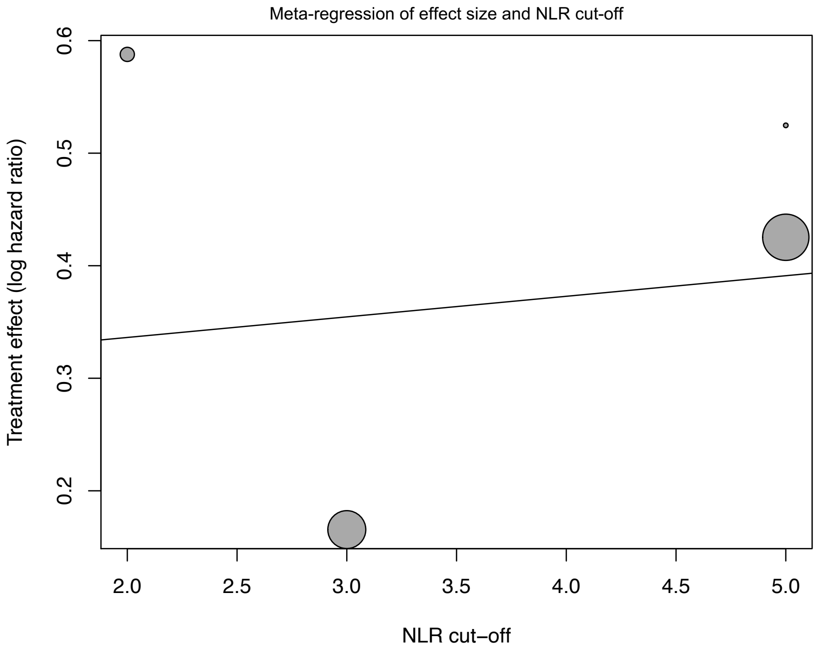

Supplementary Figure 135 - NLR: DFS in Colorectal (SR)

Study	TE	seTE	HR	95%−CI	Weight (fixed)	Weight (random)
Hung HY 2011	0.22	0.1873	1.24	[0.86; 1.79]	22.3%	16.4%
Malietzis G 2014	0.88	0.3874	2.41	[1.13; 5.15]	5.2%	8.1%
Seong MK 2015	0.99	0.4296	2.68	[1.15; 6.22]	4.2%	7.0%
Pine JK 2015	0.78	0.2928	2.18	[1.23; 3.87]	9.1%	11.3%
Shin JS 2015	1.65	0.6706	5.22	[1.40; 19.43]	1.7%	3.5%
Li H 2016	0.62	0.2353	1.86	[1.17; 2.95]	14.2%	13.9%
Pedrazzani C 2017	0.20	0.2340	1.22	[0.77; 1.93]	14.3%	13.9%
Murphy C 2017	−0.11	0.3305	0.90	[0.47; 1.72]	7.2%	9.8%
Wei Y 2018	0.04	0.1902	1.04	[0.72; 1.51]	21.7%	16.2%
**Pooled HR by Fixed Effects**			**1.42**	**[1.20; 1.69]**	**100.0%**	**--**
**Pooled HR by Random Effects**			**1.54**	**[1.18; 2.00]**	**--**	**100.0%**
**Prediction interval**				**[0.74; 3.18]**		

Heterogeneity: I^2 = 52% [0%; 78%], χ^2_8 = 16.81 (p = 0.03)

Tau²= 0.276

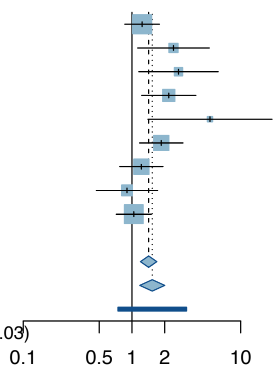

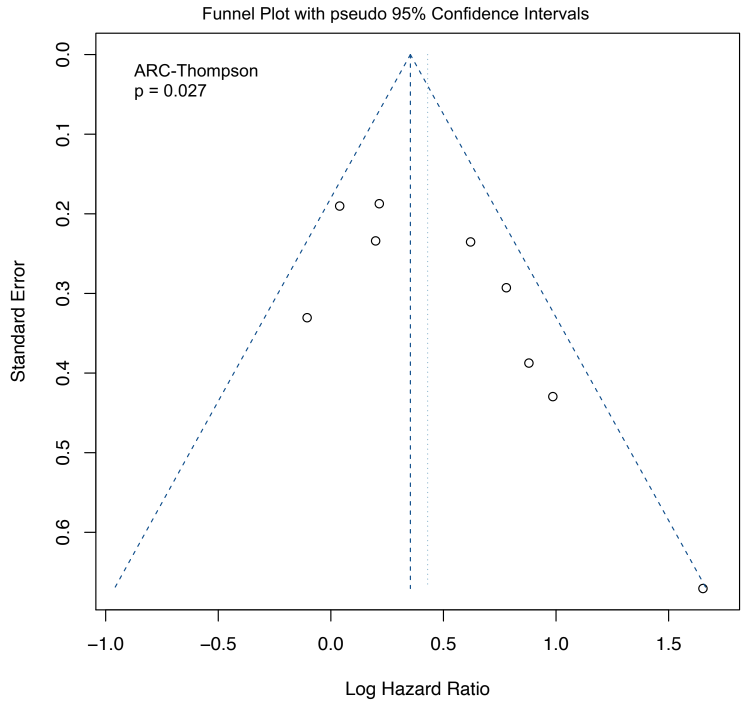

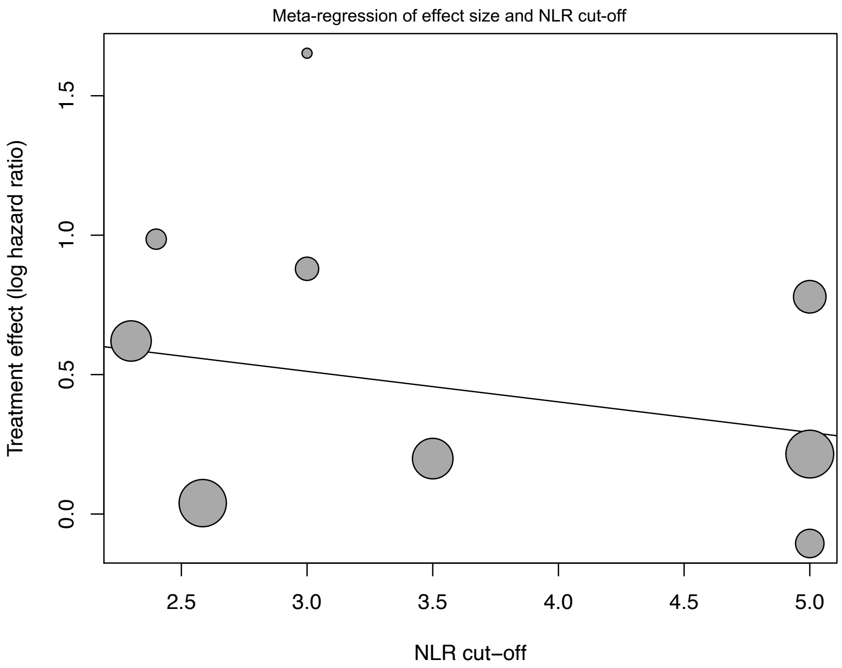

Supplementary Figure 136 - NLR: OS in Colorectal (SR)

Study	TE	seTE	HR	95%–CI	Weight (fixed)	Weight (random)
Hung HY 2011	0.25	0.1700	1.29	[0.92; 1.80]	14.5%	12.0%
Ying HQ 2014	1.00	0.2306	2.73	[1.74; 4.29]	7.9%	9.3%
Malietzis G 2014	0.21	0.2219	1.23	[0.80; 1.90]	8.5%	9.7%
Pine JK 2015	0.60	0.1681	1.82	[1.31; 2.53]	14.8%	12.1%
Nagasaki T 2015	1.22	0.4853	3.38	[1.31; 8.75]	1.8%	3.5%
Li H 2016	0.62	0.2363	1.85	[1.16; 2.94]	7.5%	9.1%
Ishizuka M 2016	0.59	0.1983	1.81	[1.23; 2.67]	10.6%	10.7%
Pedrazzani C 2017	0.14	0.1490	1.15	[0.86; 1.54]	18.8%	13.0%
Murphy C 2017	0.82	0.2735	2.27	[1.33; 3.88]	5.6%	7.8%
Borazan E 2017	1.12	0.5313	3.05	[1.08; 8.64]	1.5%	3.0%
Wei Y 2018	0.17	0.2211	1.18	[0.77; 1.82]	8.6%	9.7%
**Pooled HR by Fixed Effects**			**1.58**	**[1.39; 1.79]**	**100.0%**	**--**
**Pooled HR by Random Effects**			**1.67**	**[1.37; 2.03]**	**--**	**100.0%**
**Prediction interval**				**[0.93; 2.99]**		

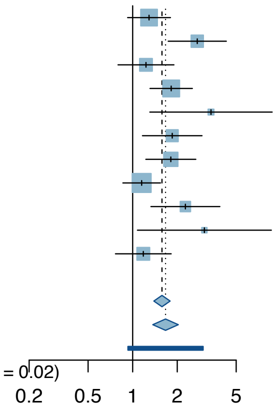

Heterogeneity: I^2 = 54% [10%; 77%], χ^2_{10} = 21.98 (p = 0.02)

Tau²= 0.238

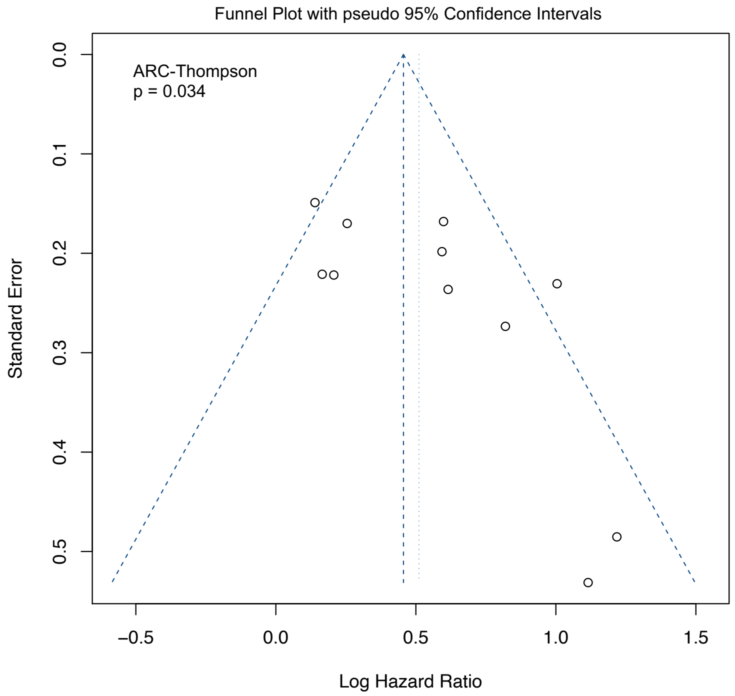

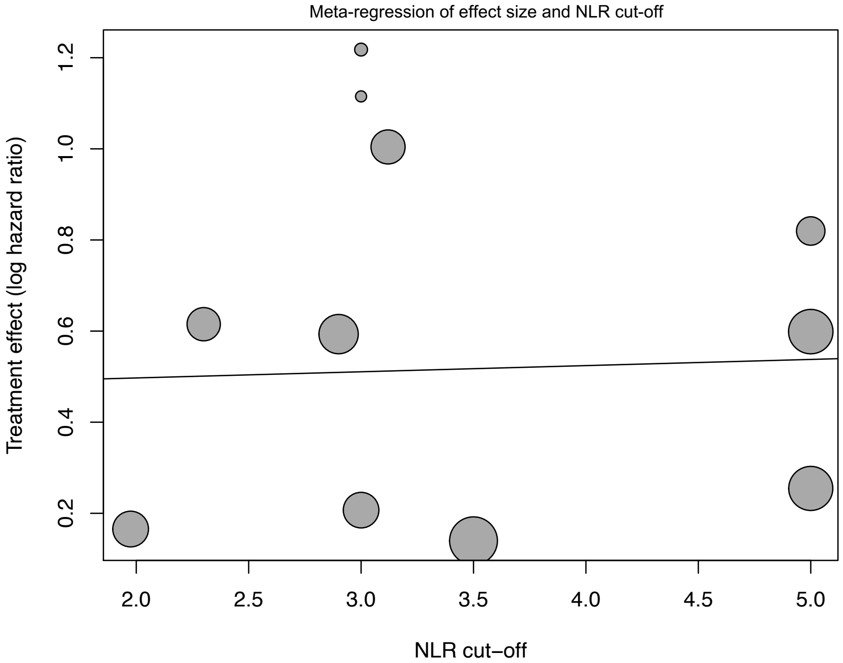

Supplementary Figure 137 - NLR: RFS in Colorectal (SR)

Study	TE	seTE	HR	95%−CI	Weight (fixed)	Weight (random)
Ding PR 2010	1.34	0.5824	3.80	[1.21; 11.90]	7.6%	7.6%
Mallappa S 2013	0.59	0.2696	1.81	[1.07; 3.07]	35.6%	35.6%
Ying HQ 2014	0.92	0.2136	2.52	[1.66; 3.83]	56.7%	56.7%
**Pooled HR by Fixed Effects**			**2.31**	**[1.69; 3.17]**	**100.0%**	**--**
**Pooled HR by Random Effects**			**2.31**	**[1.69; 3.17]**	**--**	**100.0%**
**Prediction interval**				**[0.30; 17.85]**		

Heterogeneity: $I^2 = 0\%$ [0%; 88%], $\chi^2_2 = 1.72$ ($p = 0.42$)

$Tau^2 = 0$

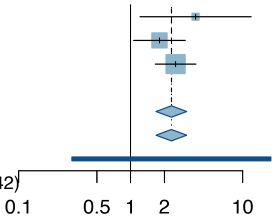

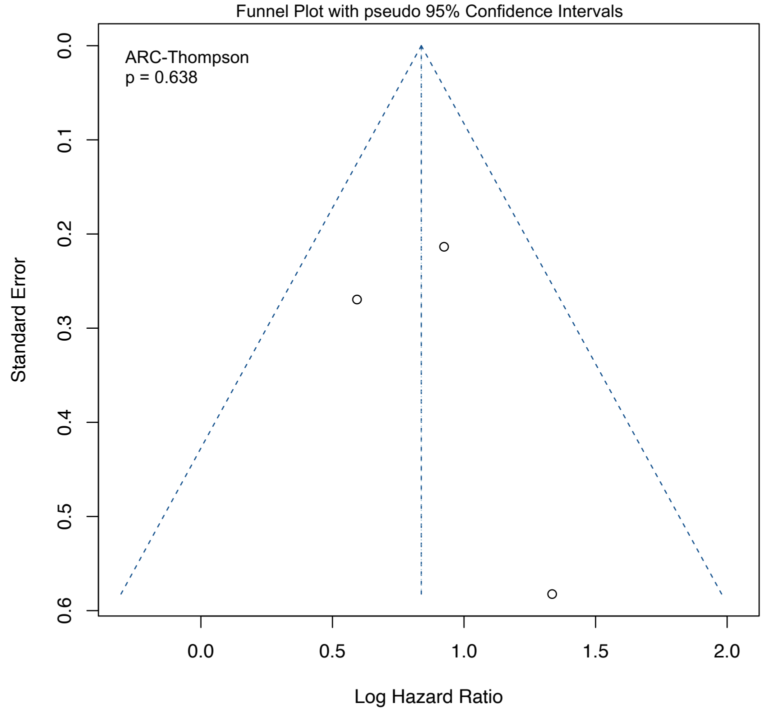

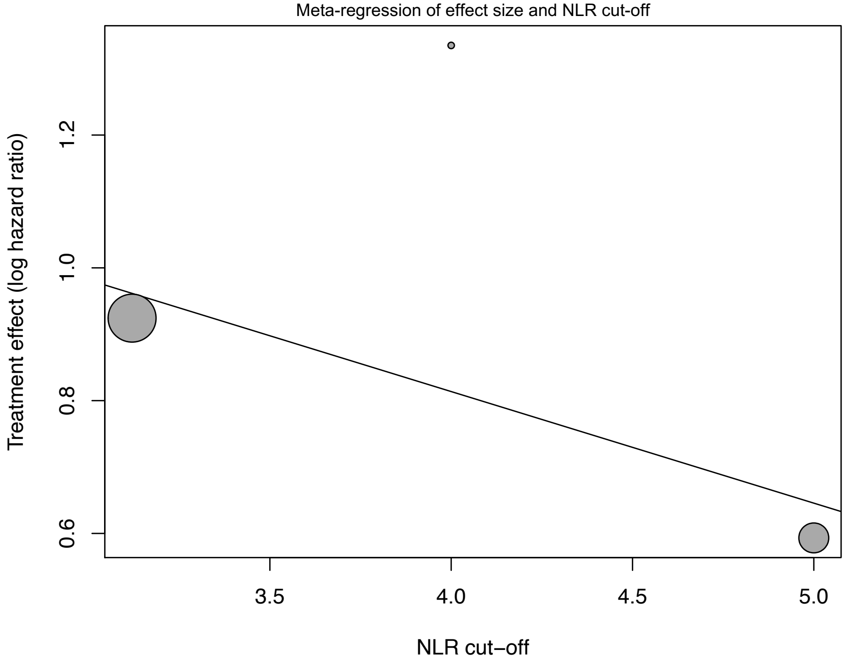

Supplementary Figure 138 - NLR: OS in Endometrial carcinoma

Study	TE	seTE	HR	95%−CI	Weight (fixed)	Weight (random)
Aoyama 2019	0.80	0.7636	2.23	[0.50; 9.96]	2.3%	2.3%
Cömert 2018	0.53	0.4852	1.70	[0.66; 4.40]	5.8%	5.8%
Cummings 2015	0.60	0.1859	1.82	[1.26; 2.62]	39.6%	39.6%
Ding 2017	1.36	0.4693	3.91	[1.56; 9.81]	6.2%	6.2%
Eo 2016	1.24	0.5426	3.47	[1.20; 10.05]	4.6%	4.6%
Haruma 2015	1.20	0.5385	3.32	[1.16; 9.54]	4.7%	4.7%
Li 2015	0.83	0.6218	2.30	[0.68; 7.78]	3.5%	3.5%
Matsuo 2015	0.78	0.3007	2.18	[1.21; 3.93]	15.1%	15.1%
Takahashi 2015	0.90	0.2757	2.47	[1.44; 4.24]	18.0%	18.0%
**Pooled HR by Fixed Effects**			**2.22**	**[1.76; 2.79]**	**100.0%**	**--**
**Pooled HR by Random Effects**			**2.22**	**[1.76; 2.79]**	**--**	**100.0%**
**Prediction interval**				**[1.68; 2.92]**		

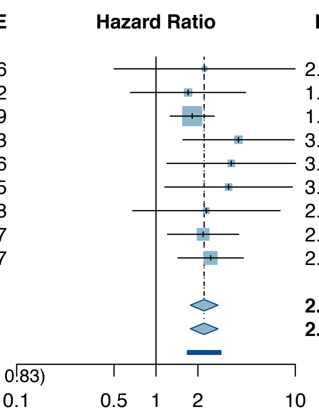

Heterogeneity: $I^2 = 0\%$ [0%; 34%], $\chi^2_8 = 4.29$ ($p = 0.83$)

Tau²= 0

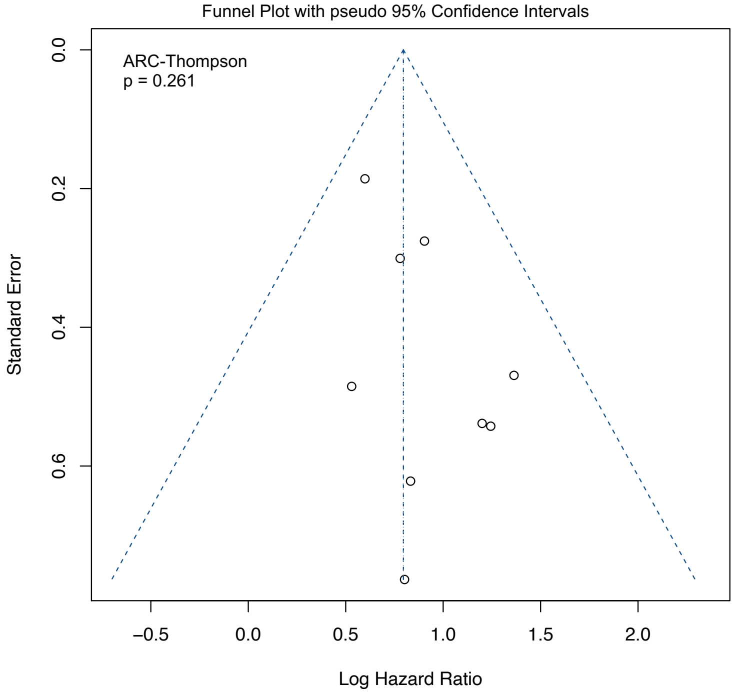

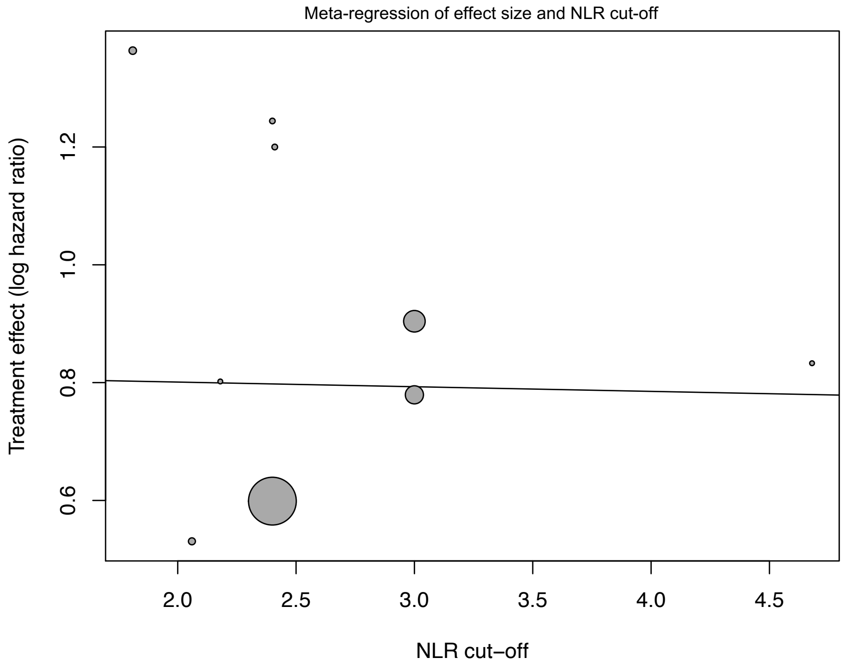

Supplementary Figure 139 - NLR: PFS in Endometrial carcinoma

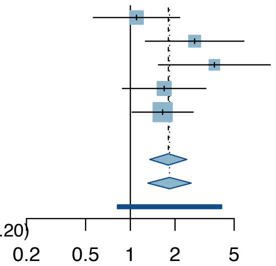

Study	TE	seTE	HR	95%–CI	Weight (fixed)	Weight (random)
Cömert 2018	0.10	0.3419	1.10	[0.56; 2.15]	18.4%	19.4%
Ding 2017	1.00	0.3900	2.71	[1.26; 5.82]	14.1%	15.8%
Eo 2016	1.30	0.4425	3.68	[1.55; 8.76]	11.0%	12.8%
Haruma 2015	0.52	0.3305	1.69	[0.88; 3.23]	19.7%	20.5%
Matsuo 2015	0.50	0.2417	1.65	[1.03; 2.65]	36.8%	31.6%
**Pooled HR by Fixed Effects**			**1.80**	**[1.35; 2.40]**	**100.0%**	**--**
**Pooled HR by Random Effects**			**1.84**	**[1.31; 2.57]**	**--**	**100.0%**
**Prediction interval**				**[0.82; 4.12]**		

Heterogeneity: $I^2 = 33\%$ [0%; 74%], $\chi^2_4 = 5.95$ ($p = 0.20$)

Tau²= 0.187

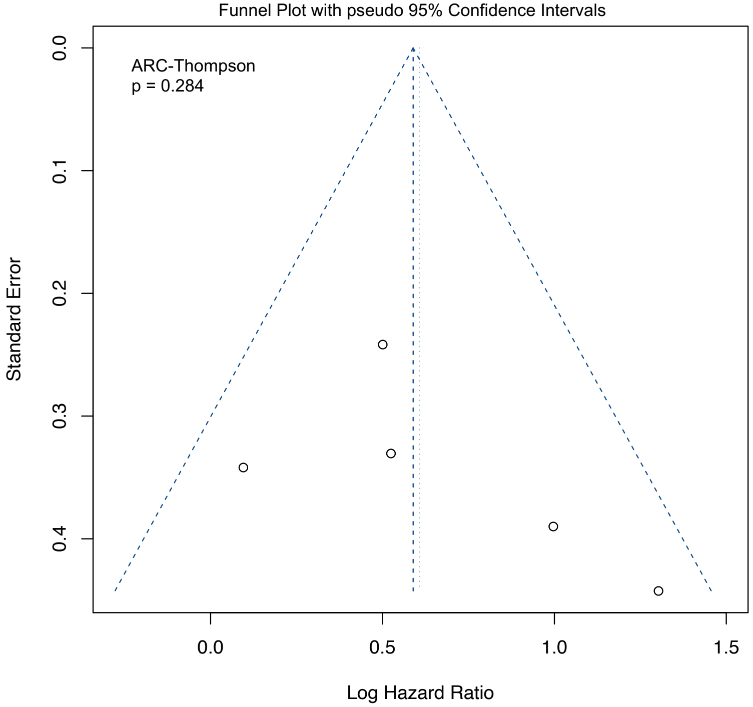

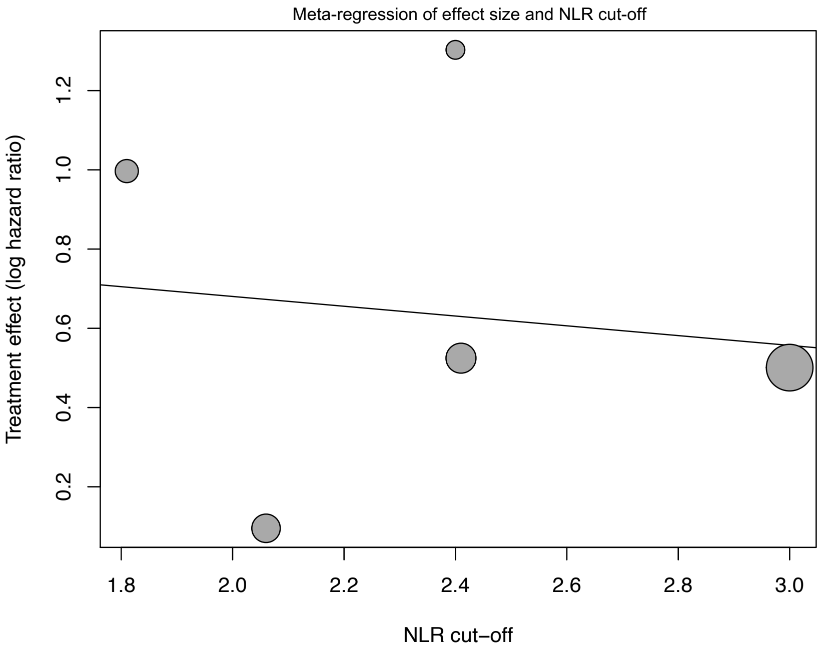

Supplementary Figure 140 - NLR: DFS in Breast (Triple Negative and Her2 Positive)

Study	TE	seTE	HR	95%-CI	Weight (fixed)	Weight (random)
Allan 2016	0.68	0.4659	1.97	[0.79; 4.91]	0.7%	1.5%
Asano 2015	0.03	0.1602	1.03	[0.75; 1.41]	6.2%	5.8%
Bozkurt 2015	1.70	0.6232	5.46	[1.61; 18.52]	0.4%	0.9%
Chae 2018	0.52	0.2169	1.68	[1.10; 2.57]	3.4%	4.4%
Chen 2016	0.45	0.2058	1.57	[1.05; 2.35]	3.8%	4.6%
Cho 2018	0.22	0.4335	1.24	[0.53; 2.90]	0.8%	1.7%
Cihan 2014	−0.22	0.2707	0.80	[0.47; 1.36]	2.2%	3.4%
Dirican 2015	0.38	0.1732	1.46	[1.04; 2.05]	5.3%	5.5%
Ferroni 2018	0.82	0.2484	2.28	[1.40; 3.71]	2.6%	3.8%
Forget 2014	0.69	0.2748	1.99	[1.16; 3.41]	2.1%	3.3%
Hernandez 2017	0.38	0.2641	1.46	[0.87; 2.45]	2.3%	3.5%
Hong 2016	0.63	0.2446	1.87	[1.16; 3.02]	2.7%	3.8%
Jia 2015	0.41	0.1391	1.50	[1.14; 1.97]	8.3%	6.5%
Koh 2014	1.35	0.4390	3.87	[1.64; 9.15]	0.8%	1.6%
Krenn−Pilko 2016	0.53	0.2265	1.70	[1.09; 2.65]	3.1%	4.2%
Lee 2018	0.55	0.3753	1.73	[0.83; 3.61]	1.1%	2.1%
Liu 2016	0.64	0.1447	1.89	[1.42; 2.51]	7.6%	6.3%
Mando 2018	0.43	0.2156	1.54	[1.01; 2.35]	3.4%	4.4%
Nakano 2014	0.69	0.3822	2.00	[0.95; 4.23]	1.1%	2.1%
Orditura 2016	0.97	0.3936	2.64	[1.22; 5.71]	1.0%	2.0%
Pistelli 2014	1.64	0.7827	5.15	[1.11; 23.88]	0.3%	0.6%
Qiu 2018	0.76	0.1210	2.13	[1.68; 2.70]	10.9%	7.0%
Takeuchi 2017	0.71	0.4444	2.03	[0.85; 4.85]	0.8%	1.6%
Templeton 2018	0.13	0.1192	1.14	[0.90; 1.44]	11.2%	7.1%
Ulas 2015	0.30	0.4057	1.35	[0.61; 2.99]	1.0%	1.9%
Yao 2014	0.18	0.1035	1.20	[0.98; 1.47]	14.9%	7.5%
Zhang 2016	0.36	0.2936	1.44	[0.81; 2.56]	1.9%	3.0%
**Pooled HR by Fixed Effects**			**1.52**	**[1.41; 1.64]**	**100.0%**	**--**
**Pooled HR by Random Effects**			**1.59**	**[1.41; 1.80]**	**--**	**100.0%**
**Prediction interval**				**[1.04; 2.45]**		

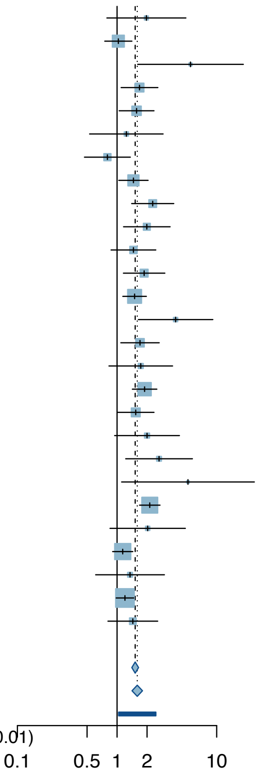

Heterogeneity: $I^2 = 50\%$ [23%; 68%], $\chi^2_{26} = 52.37$ ($p < 0.01$)

Tau²= 0.200

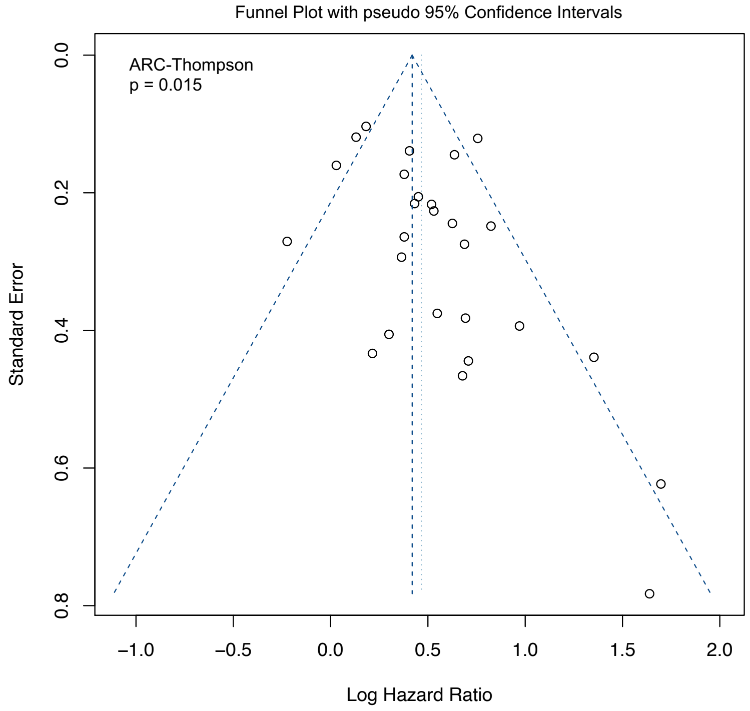

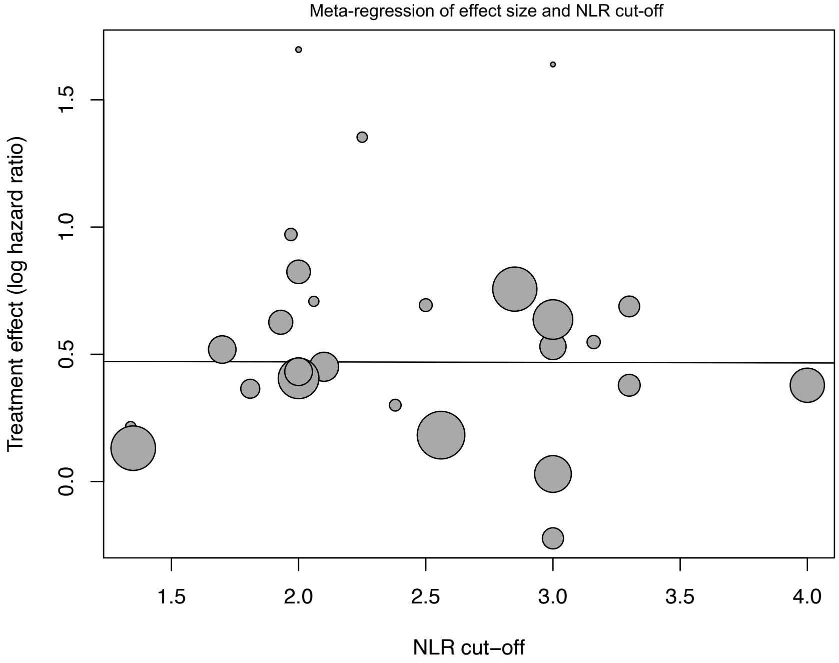

Supplementary Figure 141 - NLR: OS in Breast (Triple Negative and Her2 Positive)

Study	TE	seTE	HR	95%–CI	Weight (fixed)	Weight (random)
Allan 2016	0.59	0.4567	1.81	[0.74; 4.43]	0.8%	2.4%
Asano 2015	0.01	0.1593	1.01	[0.74; 1.38]	6.7%	5.3%
Azab 2013	1.28	0.2674	3.60	[2.13; 6.08]	2.4%	4.1%
Bozkurt 2015	1.05	0.5158	2.86	[1.04; 7.86]	0.6%	2.0%
Cihan 2014	−0.30	0.3838	0.74	[0.35; 1.57]	1.2%	2.9%
Dirican 2015	0.65	0.1915	1.91	[1.31; 2.78]	4.6%	5.0%
Ferroni 2018	1.22	0.5787	3.39	[1.09; 10.54]	0.5%	1.7%
Forget 2014	0.85	0.4254	2.35	[1.02; 5.41]	0.9%	2.6%
Hernandez 2017	0.17	0.2641	1.18	[0.70; 1.98]	2.4%	4.1%
Hong 2016	0.04	0.0897	1.04	[0.87; 1.24]	21.0%	6.0%
Iwase 2017	1.08	0.4189	2.93	[1.29; 6.66]	1.0%	2.7%
Jia 2015	0.49	0.2141	1.63	[1.07; 2.48]	3.7%	4.7%
Koh 2014	3.21	1.0665	24.87	[3.08; 201.13]	0.1%	0.6%
Koh 2015	0.31	0.1220	1.37	[1.08; 1.74]	11.4%	5.7%
Krenn−Pilko 2016	0.43	0.2692	1.54	[0.91; 2.61]	2.3%	4.1%
Lee 2018	1.15	0.4514	3.15	[1.30; 7.63]	0.8%	2.4%
Limori 2018	0.94	0.3762	2.55	[1.22; 5.33]	1.2%	3.0%
Liu 2016	0.85	0.1587	2.33	[1.71; 3.18]	6.7%	5.3%
Miyagawa 2018	0.78	0.2657	2.18	[1.29; 3.67]	2.4%	4.1%
Pistelli 2014	1.82	0.7073	6.16	[1.54; 24.64]	0.3%	1.3%
Qiu 2018	0.99	0.1668	2.69	[1.94; 3.73]	6.1%	5.2%
Takuwa 2018	0.56	0.2416	1.75	[1.09; 2.81]	2.9%	4.4%
Templeton 2018	0.17	0.1113	1.19	[0.96; 1.48]	13.7%	5.8%
Ulas 2015	−0.17	0.5957	0.84	[0.26; 2.70]	0.5%	1.7%
Vernieri 2018	0.67	0.3283	1.96	[1.03; 3.73]	1.6%	3.4%
Wariss 2017	0.03	0.3015	1.03	[0.57; 1.86]	1.9%	3.7%
Yao 2014	1.29	0.4195	3.63	[1.60; 8.26]	1.0%	2.7%
Zhang 2016	0.21	0.3740	1.23	[0.59; 2.56]	1.2%	3.0%
**Pooled HR by Fixed Effects**			**1.50**	**[1.38; 1.62]**	**100.0%**	**--**
**Pooled HR by Random Effects**			**1.78**	**[1.49; 2.12]**	**--**	**100.0%**
**Prediction interval**				**[0.84; 3.76]**		

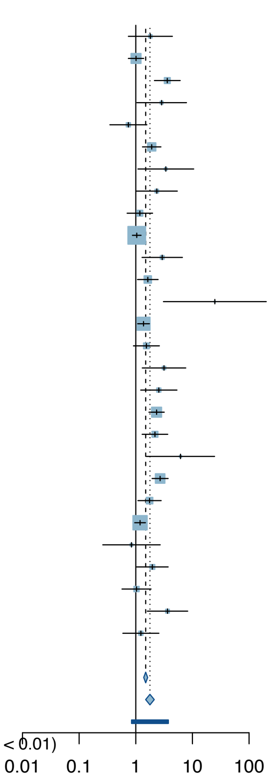

Heterogeneity: $I^2 = 72\%$ [60%; 81%], $\chi^2_{27} = 97.63$ ($p < 0.01$)

Tau²= 0.353

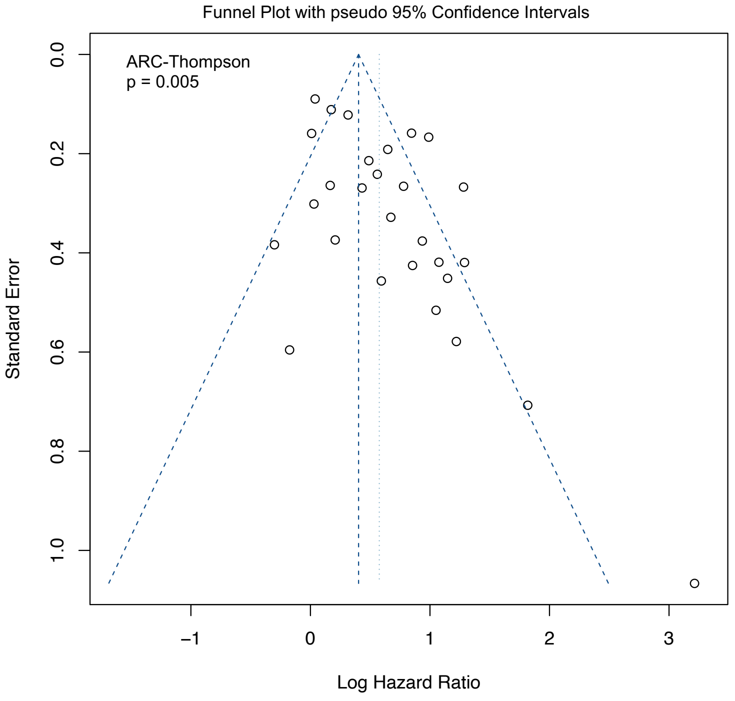

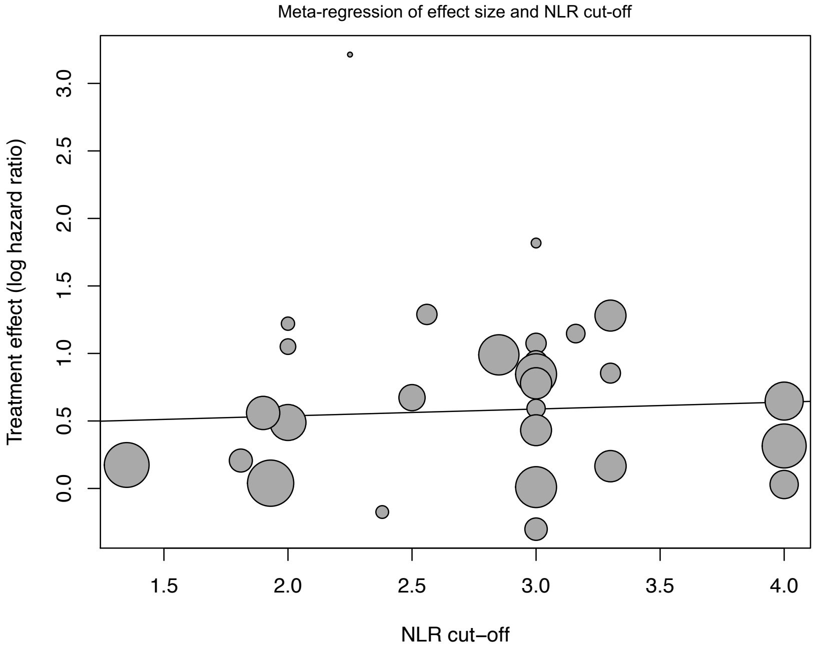

Supplementary Figure 142 - NLR: DFS in Breast (SR)

Study	TE	seTE	HR	95%−CI	Weight (fixed)	Weight (random)
Cihan YB 2014	−0.22	0.2477	0.80	[0.49; 1.30]	0.7%	7.5%
Forget P 2014	0.69	0.2748	1.99	[1.16; 3.41]	0.6%	6.9%
Nakano K 2014	0.69	0.3662	2.00	[0.98; 4.10]	0.3%	5.2%
Suppan C 2015	0.01	0.0247	1.01	[0.96; 1.06]	70.2%	11.8%
Dirican A 2015	0.38	0.1707	1.46	[1.04; 2.04]	1.5%	9.3%
Jia W 2015	0.95	0.3787	2.58	[1.23; 5.42]	0.3%	5.0%
Bozkurt O 2015	1.70	0.6226	5.46	[1.61; 18.50]	0.1%	2.5%
Hong J 2016	0.63	0.2446	1.87	[1.16; 3.02]	0.7%	7.5%
Kim YY 2016	1.37	0.5742	3.93	[1.28; 12.11]	0.1%	2.8%
Takeuchi H 2017	0.71	0.4433	2.03	[0.85; 4.84]	0.2%	4.1%
Marin Hernández C 2018	−1.08	0.7573	0.34	[0.08; 1.50]	0.1%	1.8%
Cho U 2018	0.17	0.0455	1.18	[1.08; 1.29]	20.6%	11.7%
Geng SK 2018	0.49	0.2171	1.64	[1.07; 2.51]	0.9%	8.2%
Lee J 2018	0.55	0.3739	1.73	[0.83; 3.60]	0.3%	5.1%
Qui X 2018	0.76	0.1114	2.13	[1.71; 2.65]	3.4%	10.6%
**Pooled HR by Fixed Effects**			**1.10**	**[1.06; 1.15]**	**100.0%**	**--**
**Pooled HR by Random Effects**			**1.58**	**[1.27; 1.96]**	**--**	**100.0%**
**Prediction interval**				**[0.76; 3.29]**		

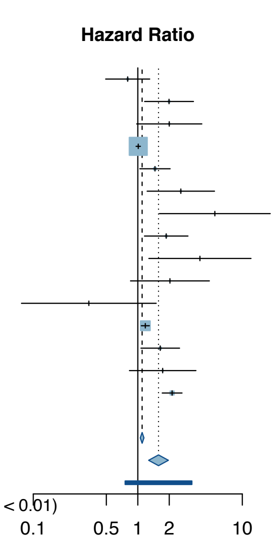

Heterogeneity: $I^2 = 85\%$ [76%; 90%], $\chi^2_{14} = 91.73$ ($p < 0.01$)

Tau²= 0.322

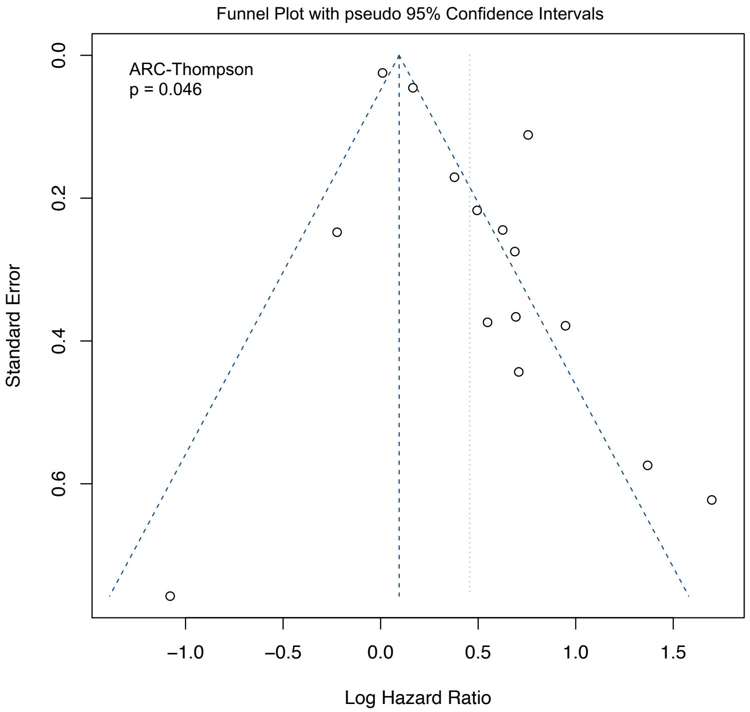

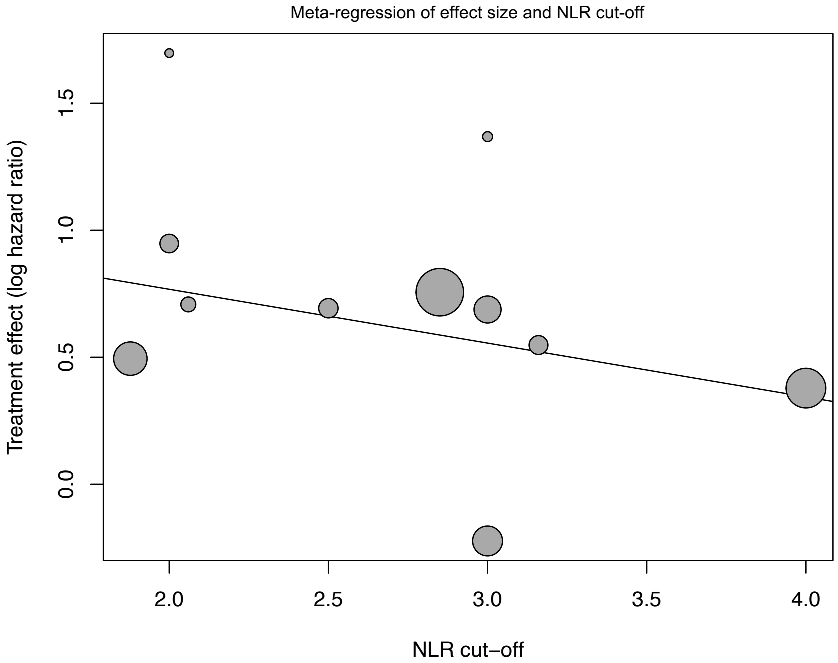

Supplementary Figure 143 - NLR: OS in Breast (SR)

Study	TE	seTE	HR	95%–CI	Weight (fixed)	Weight (random)
Forget P 2013	1.39	0.6500	4.00	[1.12; 14.30]	2.3%	5.6%
Cihan YB 2014	−0.36	0.3536	0.70	[0.35; 1.40]	7.9%	10.8%
Koh YW 2014	3.21	1.0669	24.87	[3.07; 201.29]	0.9%	2.6%
Yao M 2014	1.29	0.4195	3.63	[1.60; 8.26]	5.6%	9.3%
Forget P 2014	0.85	0.4273	2.35	[1.02; 5.43]	5.4%	9.2%
Pistelli M 2015	1.82	0.7077	6.16	[1.54; 24.66]	2.0%	5.0%
Dirican A 2015	0.65	0.1933	1.91	[1.31; 2.79]	26.4%	14.8%
Bozkurt O 2015	1.05	0.5158	2.86	[1.04; 7.86]	3.7%	7.5%
Jia W 2015	1.12	0.5295	3.05	[1.08; 8.61]	3.5%	7.3%
Qui X 2018	0.99	0.1654	2.69	[1.95; 3.72]	36.0%	15.4%
Marin Hernández C 2018	−0.53	0.8690	0.59	[0.11; 3.24]	1.3%	3.7%
Lee J 2018	1.15	0.4426	3.15	[1.32; 7.50]	5.0%	8.8%
**Pooled HR by Fixed Effects**			**2.32**	**[1.91; 2.82]**	**100.0%**	**--**
**Pooled HR by Random Effects**			**2.47**	**[1.71; 3.56]**	**--**	**100.0%**
**Prediction interval**				**[0.84; 7.25]**		

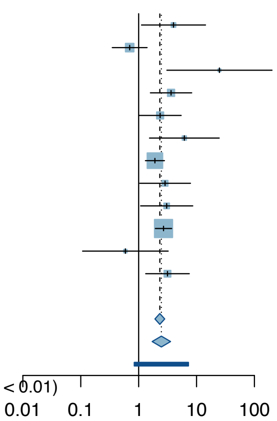

Heterogeneity: I^2 = 57% [17%; 77%], χ^2_{11} = 25.37 (p < 0.01)

Tau²= 0.446

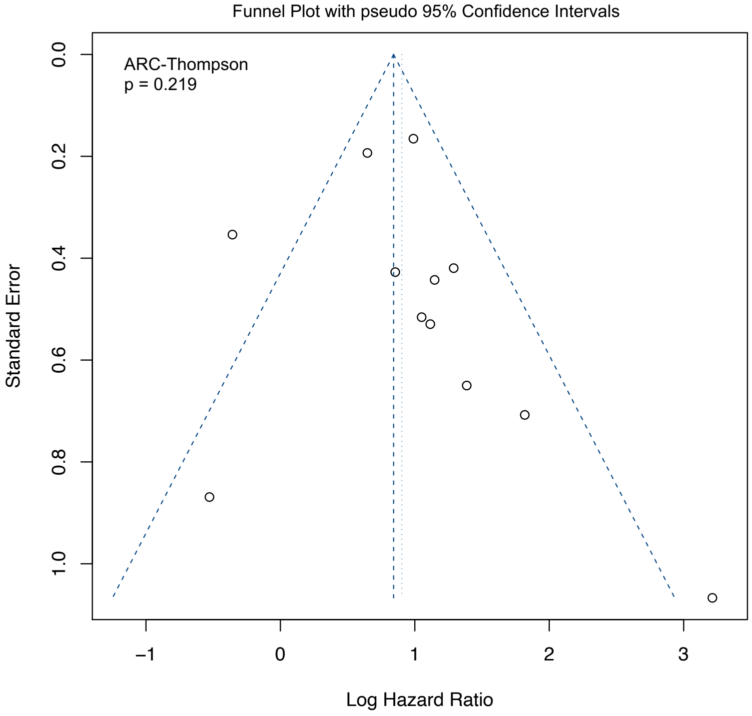

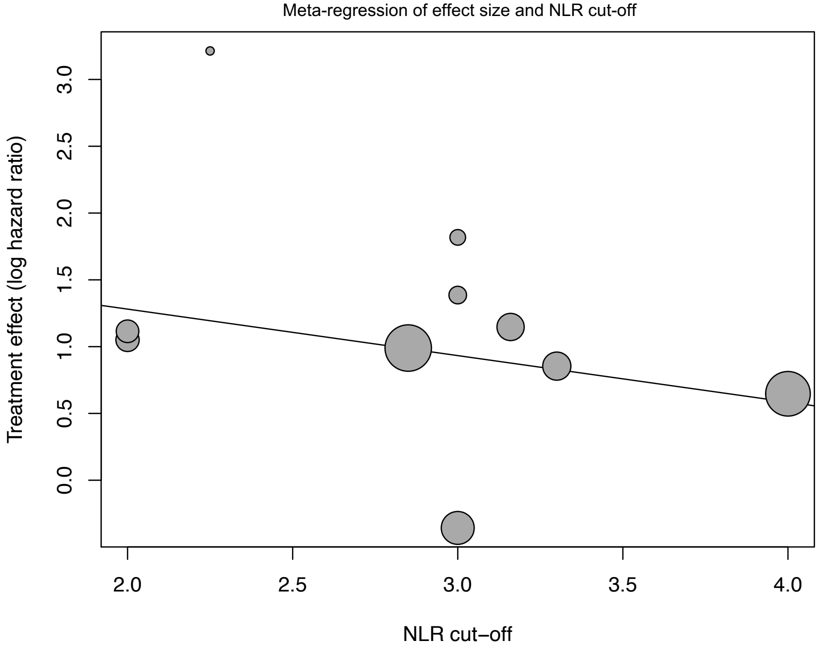

Supplementary Figure 144 - NLR: RFS in Breast (SR)

Study	TE	seTE	Hazard Ratio	HR	95%–CI	Weight (fixed)	Weight (random)
Forget P 2013	1.53	0.7263		4.60	[1.11; 19.10]	26.7%	26.7%
Koh YW 2014	1.35	0.4385		3.87	[1.64; 9.14]	73.3%	73.3%
**Pooled HR by Fixed Effects**				**4.05**	**[1.94; 8.46]**	**100.0%**	**--**
**Pooled HR by Random Effects**				**4.05**	**[1.94; 8.46]**	**--**	**100.0%**

Heterogeneity: $I^2 = 0\%$, $\chi^2_1 = 0.04$ ($p = 0.84$)

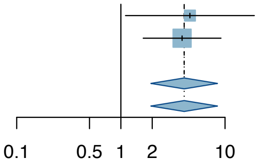

Tau²= 0

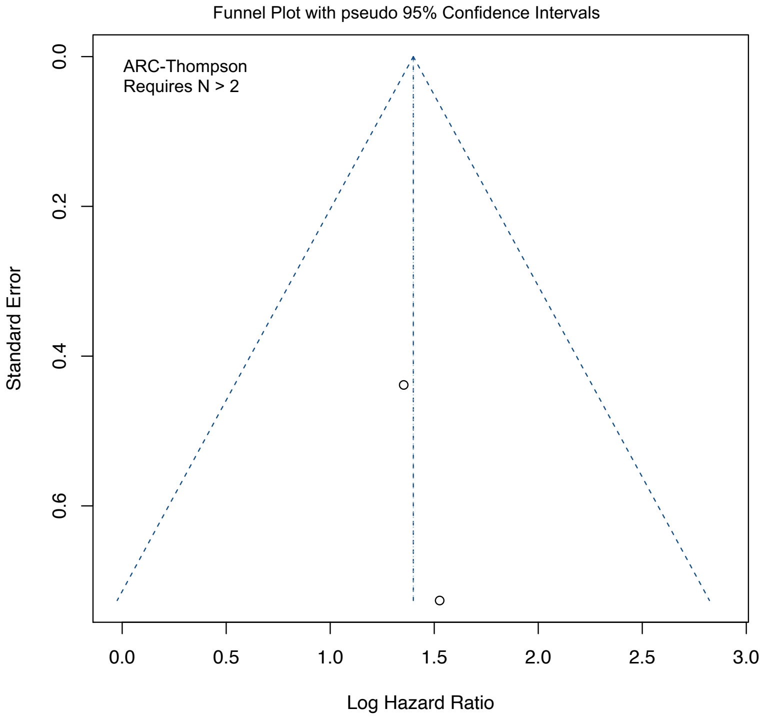

Supplementary Figure 145 - NLR: OS in Hepatocellular (Sorfenib)

Study	TE	seTE	HR	95%–CI	Weight (fixed)	Weight (random)
Afshar 2018	0.28	0.1639	1.32	[0.96; 1.82]	5.8%	11.6%
Bruix 2017	0.76	0.1948	2.13	[1.45; 3.12]	4.1%	10.1%
Casadei Gardini 2016	0.86	0.4040	2.36	[1.07; 5.21]	1.0%	4.0%
Conroy 2017	0.55	0.1683	1.74	[1.25; 2.42]	5.5%	11.4%
Diaz–Beveridge 2018	0.55	0.2214	1.73	[1.12; 2.67]	3.2%	8.9%
Fonseca 2014	0.69	0.2269	2.00	[1.28; 3.12]	3.0%	8.6%
Howell 2017	0.20	0.0479	1.22	[1.11; 1.34]	68.5%	17.8%
Lue 2017	0.54	0.2320	1.72	[1.09; 2.71]	2.9%	8.4%
Personeni 2017	0.46	0.2280	1.58	[1.01; 2.47]	3.0%	8.6%
Wei 2014	1.12	0.3021	3.07	[1.70; 5.55]	1.7%	6.1%
Zheng 2013	1.04	0.3791	2.84	[1.35; 5.97]	1.1%	4.4%
**Pooled HR by Fixed Effects**			**1.38**	**[1.28; 1.49]**	**100.0%**	**--**
**Pooled HR by Random Effects**			**1.73**	**[1.45; 2.07]**	**--**	**100.0%**
**Prediction interval**				**[1.03; 2.91]**		

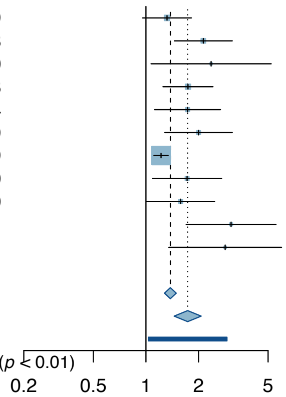

Heterogeneity: I^2 = 68% [39%; 83%], χ^2_{10} = 30.93 (p < 0.01)

Tau²= 0.210

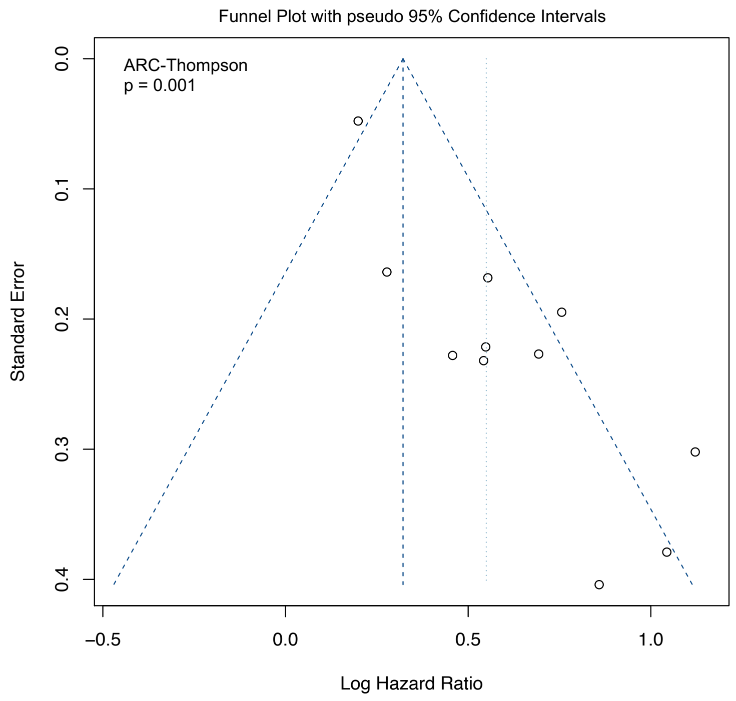

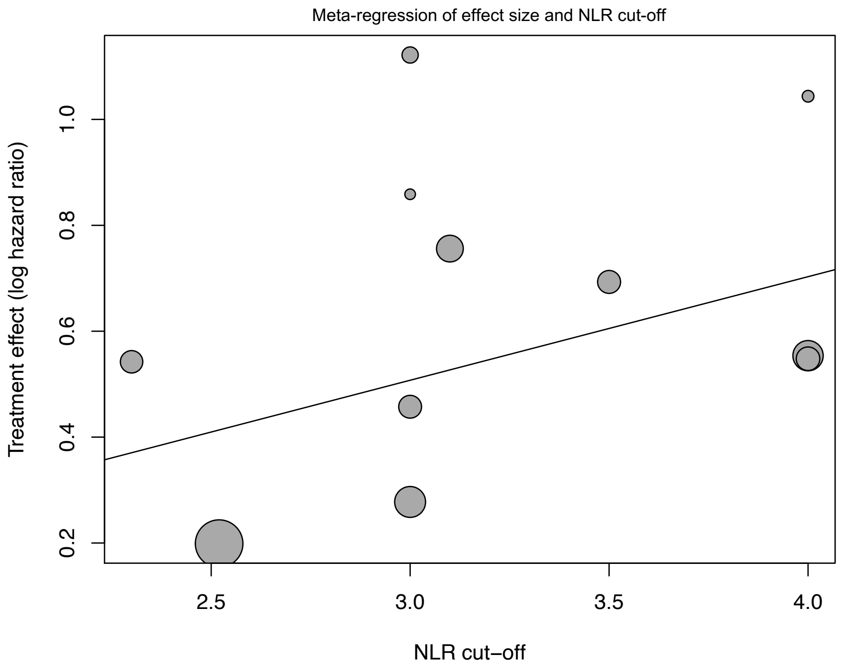

Supplementary Figure 146 - NLR: OS in Hepatocellular (TACE)

Study	TE	seTE	HR	95%−CI	Weight (fixed)	Weight (random)
Chon 2019	0.32	0.1299	1.38	[1.07; 1.78]	10.9%	9.8%
McNally 2013	1.07	0.2493	2.92	[1.79; 4.76]	3.0%	4.4%
Zhang 2014	0.76	0.1891	2.14	[1.48; 3.10]	5.1%	6.5%
Fan 2015	0.81	0.2398	2.25	[1.41; 3.60]	3.2%	4.7%
Huang 2011	0.52	0.1513	1.68	[1.25; 2.26]	8.0%	8.4%
Li 2013	0.54	0.1091	1.72	[1.39; 2.13]	15.5%	11.3%
Sun 2018	0.92	0.2699	2.51	[1.48; 4.26]	2.5%	3.9%
Tian 2016	0.11	0.2670	1.12	[0.66; 1.89]	2.6%	4.0%
Yang 2015	0.61	0.2196	1.84	[1.20; 2.83]	3.8%	5.3%
Zheng 2013	0.87	0.3296	2.39	[1.25; 4.56]	1.7%	2.8%
Zhou 2016	0.38	0.1208	1.46	[1.15; 1.85]	12.6%	10.4%
He 2019	0.91	0.2515	2.48	[1.51; 4.06]	2.9%	4.3%
Liu 2017	0.76	0.0993	2.14	[1.76; 2.60]	18.6%	12.1%
Rebonato 2017	2.44	0.8101	11.47	[2.34; 56.12]	0.3%	0.5%
Xu 2014	0.52	0.2606	1.68	[1.01; 2.80]	2.7%	4.1%
Zou 2017	0.54	0.1678	1.72	[1.24; 2.39]	6.5%	7.5%
**Pooled HR by Fixed Effects**			**1.81**	**[1.67; 1.97]**	**100.0%**	**--**
**Pooled HR by Random Effects**			**1.85**	**[1.64; 2.08]**	**--**	**100.0%**
**Prediction interval**				**[1.33; 2.57]**		

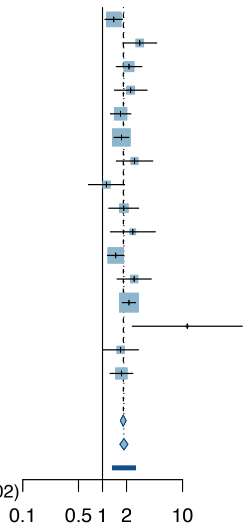

Heterogeneity: I^2 = 47% [6%; 71%], χ^2_{15} = 28.47 (p = 0.02)

Tau²= 0.142

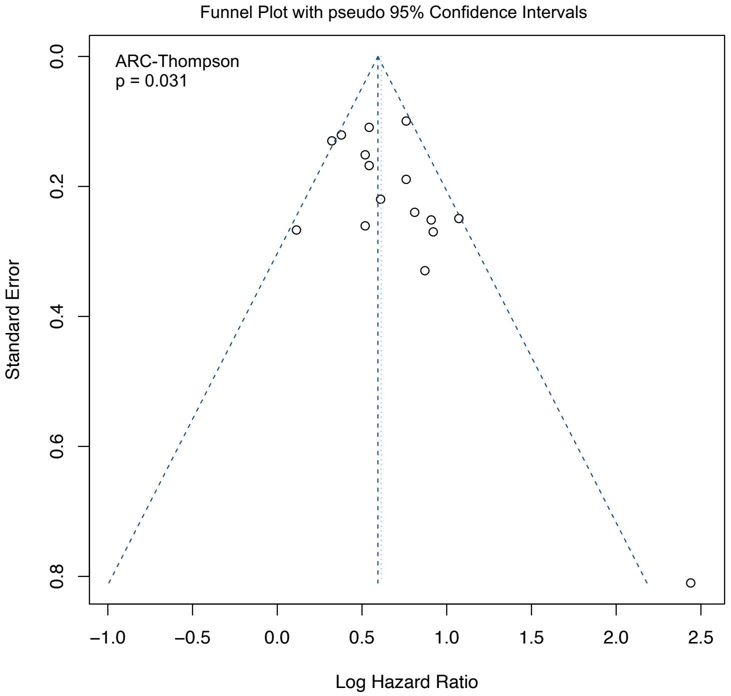

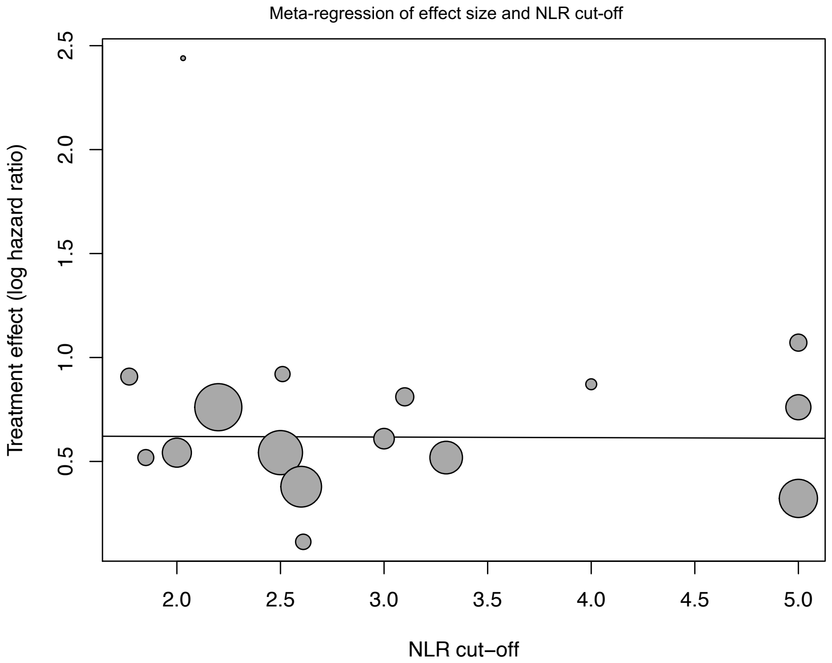

Supplementary Figure 147 - NLR: OS in Glioma

Study	TE	seTE	HR	95%-CI	Weight (fixed)	Weight (random)
**Adjustment = Multivariate**						
Bambury 2013	0.59	0.2612	1.81	[1.08; 3.02]	0.2%	5.4%
Bao 2018	0.57	0.2126	1.76	[1.16; 2.67]	0.3%	6.4%
Gan 2019	0.54	0.2405	1.71	[1.07; 2.74]	0.3%	5.8%
Han 2015	0.05	0.0237	1.05	[1.00; 1.10]	27.6%	10.0%
Kaya 2017	0.88	0.3287	2.41	[1.27; 4.59]	0.1%	4.3%
Lopes 2017	0.00	0.0151	1.00	[0.97; 1.03]	68.3%	10.0%
Matthew 2017	0.00	0.1825	1.00	[0.70; 1.43]	0.5%	7.1%
S.S.Q. 2017	−0.01	0.3050	0.99	[0.54; 1.80]	0.2%	4.6%
Wang 2017	0.54	0.2624	1.71	[1.02; 2.86]	0.2%	5.4%
Wang 2018	0.66	0.3320	1.93	[1.01; 3.70]	0.1%	4.2%
Weng 2018	0.67	0.2265	1.95	[1.25; 3.04]	0.3%	6.1%
Wiencke 2017	0.70	0.3060	2.02	[1.11; 3.68]	0.2%	4.6%
Yang 2019	0.71	0.2423	2.04	[1.27; 3.28]	0.3%	5.8%
**Pooled HR by Fixed Effects**			**1.03**	**[1.00; 1.05]**	**98.6%**	**--**
**Pooled HR by Random Effects**			**1.48**	**[1.22; 1.79]**	**--**	**79.8%**
Heterogeneity: $I^2 = 78\%$ [63%; 87%], $\chi^2_{12} = 54.68$ ($p < 0.01$)						
**Adjustment = Univariate**						
Auezova 2016	0.33	0.1541	1.39	[1.03; 1.88]	0.7%	7.7%
Hao 2019	0.94	0.1691	2.57	[1.84; 3.58]	0.5%	7.4%
Yersal 2018	0.23	0.2797	1.26	[0.73; 2.18]	0.2%	5.1%
**Pooled HR by Fixed Effects**			**1.74**	**[1.42; 2.14]**	**1.4%**	**--**
**Pooled HR by Random Effects**			**1.69**	**[1.08; 2.64]**	**--**	**20.2%**
Heterogeneity: $I^2 = 77\%$ [26%; 93%], $\chi^2_{2} = 8.78$ ($p = 0.01$)						
**Pooled HR by Fixed Effects**			**1.03**	**[1.01; 1.06]**	**100.0%**	**--**
**Pooled HR by Random Effects**			**1.52**	**[1.28; 1.81]**	**--**	**100.0%**
**Prediction interval**				**[0.81; 2.88]**		

Heterogeneity: $I^2 = 83\%$ [74%; 89%], $\chi^2_{15} = 88.11$ ($p < 0.01$)

Residual heterogeneity: $I^2 = 78\%$ [64%; 86%], $\chi^2_{14} = 63.46$ ($p < 0.01$)

Hazard Ratio axis: 0.5, 1, 2

Tau²= 0.283

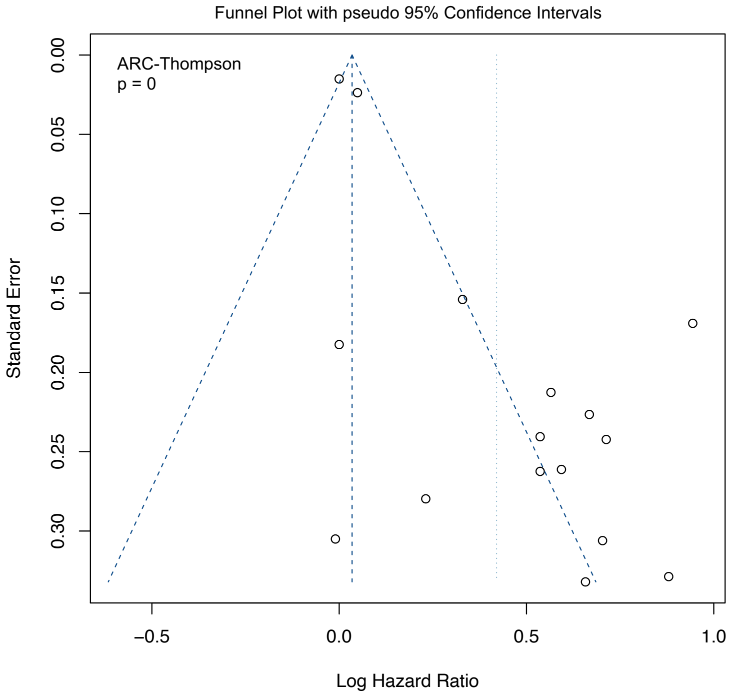

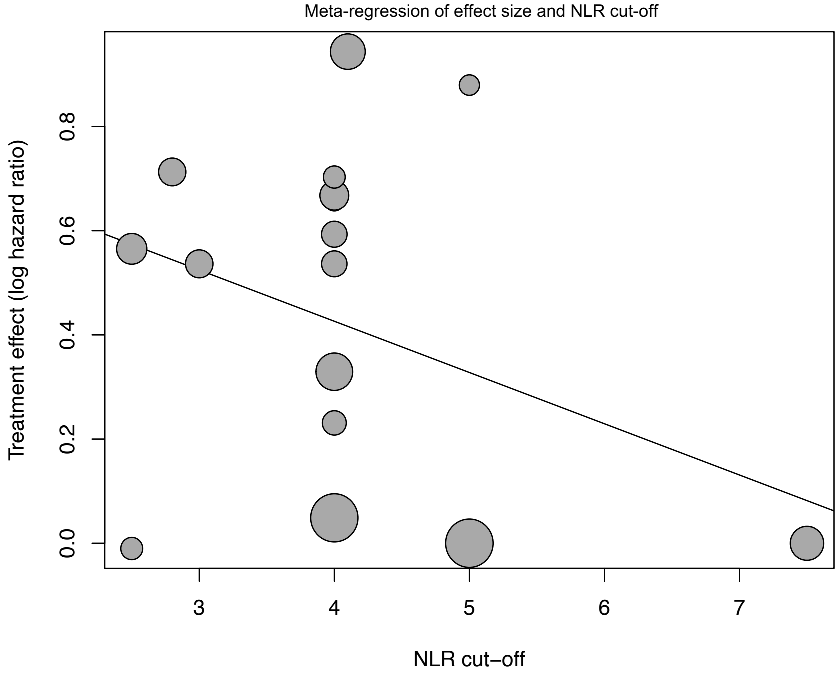

Supplementary Figure 148 - NLR: OS in NSCLC

Study	TE	seTE	HR	95%–CI	Weight (fixed)	Weight (random)
**Adjustment = Multivariate**						
Deng 2017	0.30	0.1439	1.35	[1.02; 1.79]	3.5%	5.7%
Hong 2015	−0.09	0.1150	0.91	[0.73; 1.14]	5.5%	6.5%
Huang 2016	0.51	0.2386	1.66	[1.04; 2.65]	1.3%	3.6%
Ju 2018	0.22	0.0651	1.25	[1.10; 1.42]	17.2%	7.7%
Kasmann 2017	0.72	0.3346	2.05	[1.06; 3.95]	0.6%	2.3%
Li 2019	0.09	0.1697	1.09	[0.78; 1.52]	2.5%	5.1%
Liu 2019	0.96	0.3307	2.62	[1.37; 5.01]	0.7%	2.4%
Lohinai 2019	0.31	0.2217	1.36	[0.88; 2.10]	1.5%	3.9%
Murray 2014	1.77	0.8168	5.89	[1.19; 29.20]	0.1%	0.5%
Sakin 2019	0.82	0.3039	2.26	[1.25; 4.10]	0.8%	2.7%
Suzuki (1) 2018	0.49	0.1317	1.63	[1.26; 2.11]	4.2%	6.0%
Suzuki (2) 2018	0.62	0.2456	1.86	[1.15; 3.01]	1.2%	3.5%
Wang (1) 2017	0.19	0.0559	1.21	[1.08; 1.35]	23.3%	7.9%
Wang (2) 2016	0.54	0.2224	1.72	[1.11; 2.66]	1.5%	3.9%
Wang (3) 2017	0.57	0.1782	1.77	[1.25; 2.51]	2.3%	4.9%
Wang (4) 2014	0.53	0.2454	1.70	[1.05; 2.75]	1.2%	3.5%
Wang (5) 2019	0.32	0.2019	1.38	[0.93; 2.05]	1.8%	4.3%
Xie (1) 2015	0.16	0.0988	1.17	[0.96; 1.42]	7.5%	6.9%
Xie (2) 2015	0.34	0.0649	1.40	[1.23; 1.59]	17.3%	7.7%
Zhang 2017	0.68	0.1620	1.98	[1.44; 2.72]	2.8%	5.3%
**Pooled HR by Fixed Effects**			**1.32**	**[1.25; 1.40]**	**96.7%**	**--**
**Pooled HR by Random Effects**			**1.44**	**[1.29; 1.61]**	**--**	**94.4%**
Heterogeneity: $I^2 = 59\%$ [33%; 75%], $\chi^2_{19} = 46.66$ ($p < 0.01$)						
**Adjustment = Univariate**						
Bernhardt 2018	−0.15	0.1483	0.86	[0.64; 1.15]	3.3%	5.6%
**Pooled HR by Fixed Effects**			**0.86**	**[0.64; 1.15]**	**3.3%**	**--**
**Pooled HR by Random Effects**			**0.86**	**[0.64; 1.15]**	**--**	**5.6%**
Heterogeneity: not applicable						
**Pooled HR by Fixed Effects**			**1.30**	**[1.24; 1.38]**	**100.0%**	**--**
**Pooled HR by Random Effects**			**1.41**	**[1.26; 1.59]**	**--**	**100.0%**
**Prediction interval**				**[0.90; 2.22]**		

Heterogeneity: $I^2 = 64\%$ [42%; 77%], $\chi^2_{20} = 54.83$ ($p < 0.01$)

Residual heterogeneity: $I^2 = 59\%$ [33%; 75%], $\chi^2_{19} = 46.66$ ($p < 0.01$)

Tau²= 0.207

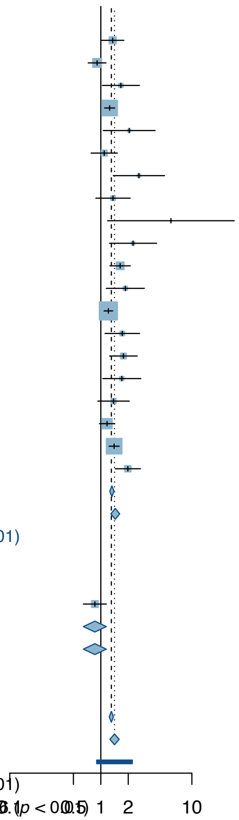

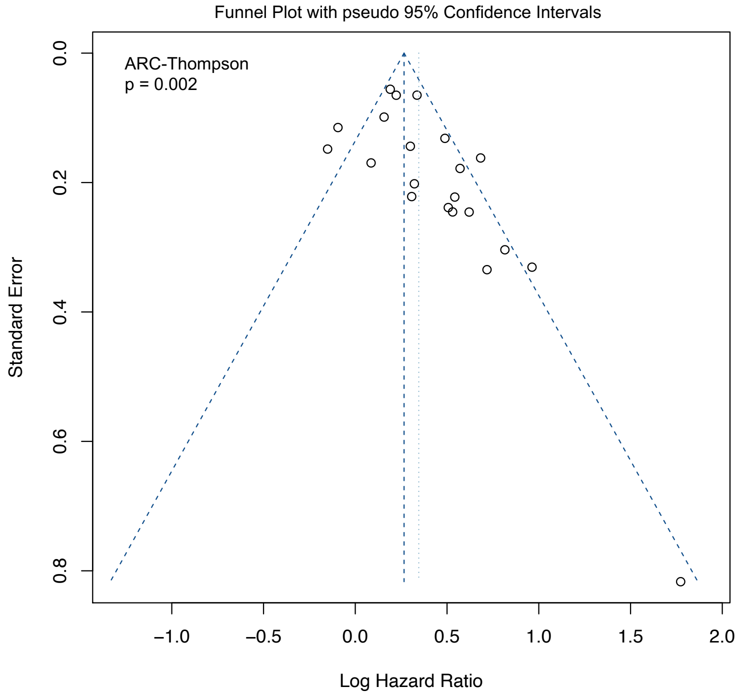

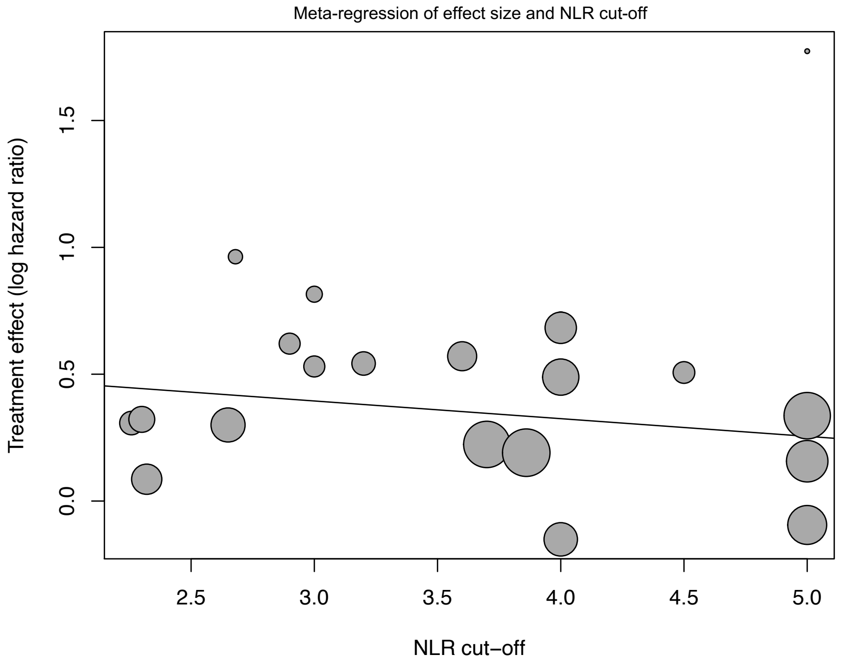

Supplementary Figure 149 - NLR: PFS in NSCLC (Immune Checkpoint Inhibitors)

Study	TE	seTE	HR	95%–CI	Weight (fixed)	Weight (random)
Alessandro Russo 2018	0.05	0.0284	1.05	[0.99; 1.11]	91.5%	29.2%
Aya Nakaya 2018	0.16	0.2338	1.17	[0.74; 1.85]	1.3%	13.2%
Diem S 2017	0.74	0.2746	2.09	[1.22; 3.58]	1.0%	10.9%
Stephen J Bagley 2017	0.36	0.1712	1.43	[1.02; 2.00]	2.5%	17.8%
Takayuki Shiroyama 2017	0.38	0.1631	1.46	[1.06; 2.01]	2.8%	18.5%
Tomoya Fukui 2018	0.69	0.2837	1.99	[1.14; 3.47]	0.9%	10.4%
**Pooled HR by Fixed Effects**			**1.08**	**[1.03; 1.14]**	**100.0%**	**--**
**Pooled HR by Random Effects**			**1.38**	**[1.10; 1.72]**	**--**	**100.0%**
**Prediction interval**				**[0.71; 2.66]**		

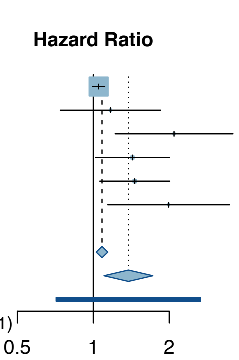

Heterogeneity: $I^2 = 72\%$ [34%; 88%], $\chi^2_5 = 17.62$ ($p < 0.01$)

Tau²= 0.208

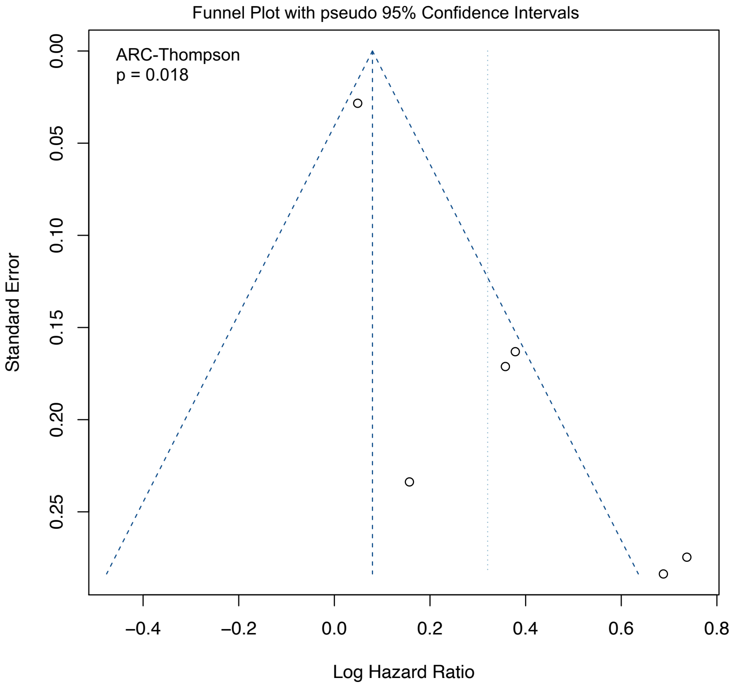

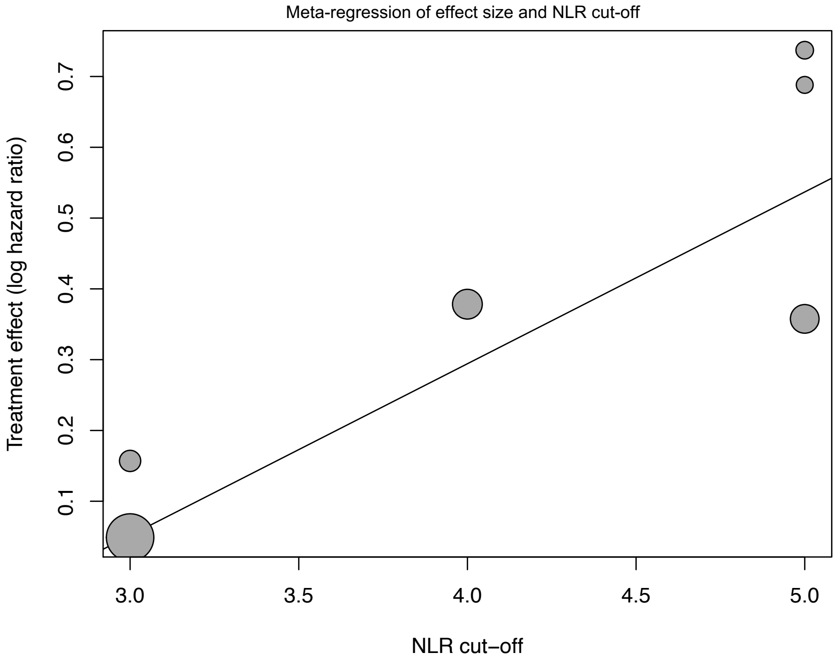

Supplementary Figure 150 - NLR: OS in NSCLC (Chemotherapy)

Study	TE	seTE	HR	95%–CI	Weight (fixed)	Weight (random)
Bozkaya Y 2019	0.29	0.2069	1.34	[0.89; 2.01]	2.1%	12.2%
Guo D 2019	0.67	0.2622	1.95	[1.17; 3.26]	1.3%	11.0%
Yi F 2018	1.33	0.4651	3.77	[1.52; 9.38]	0.4%	7.0%
Minami S 2018	0.31	0.3629	1.37	[0.67; 2.79]	0.7%	8.9%
Minami S2 2018	0.09	0.2502	1.09	[0.67; 1.78]	1.5%	11.3%
Xiong Y 2017	1.71	0.3174	5.54	[2.97; 10.32]	0.9%	9.8%
Shiran I 2017	0.06	0.0326	1.06	[0.99; 1.13]	86.3%	14.8%
Liu ZL 2016	0.52	0.1329	1.68	[1.29; 2.18]	5.2%	13.6%
Yao Y 2013	0.57	0.2423	1.76	[1.09; 2.83]	1.6%	11.4%
**Pooled HR by Fixed Effects**			**1.13**	**[1.07; 1.20]**	**100.0%**	**--**
**Pooled HR by Random Effects**			**1.74**	**[1.24; 2.42]**	**--**	**100.0%**
**Prediction interval**				**[0.57; 5.31]**		

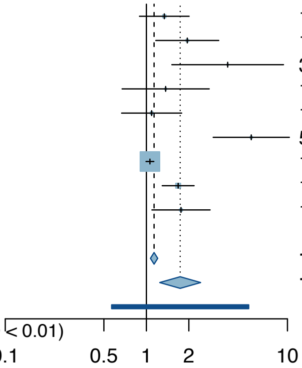

Heterogeneity: $I^2 = 85\%$ [73%; 92%], $\chi^2_8 = 53.18$ ($p < 0.01$)

Tau²= 0.441

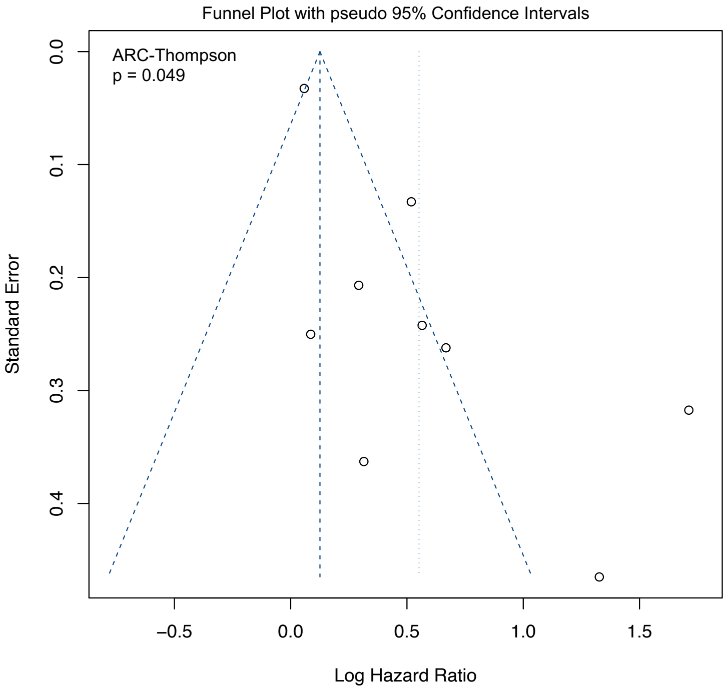

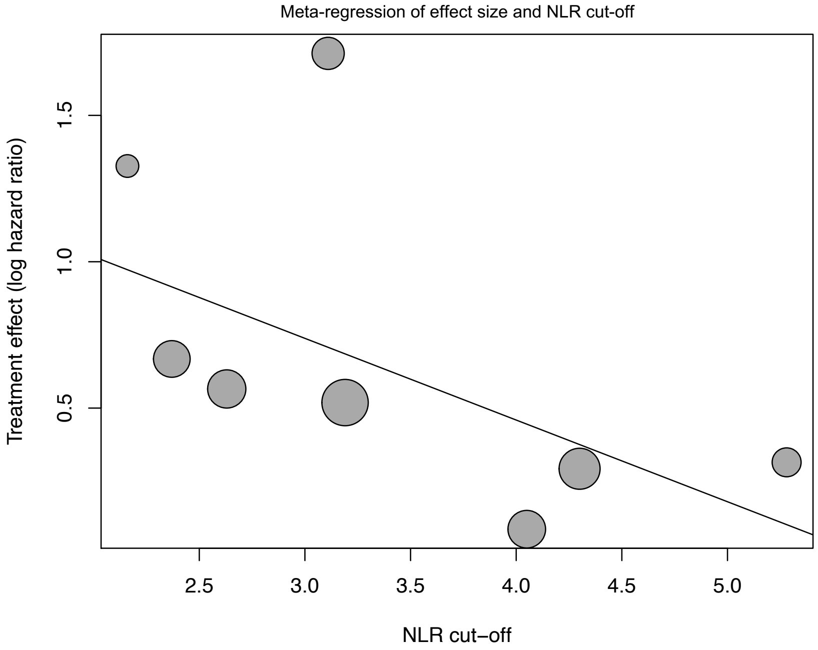

Supplementary Figure 151 - NLR: PFS in NSCLC (Chemotherapy)

Study	TE	seTE	HR	95%−CI	Weight (fixed)	Weight (random)
Guo D 2019	0.90	0.3248	2.46	[1.30; 4.65]	8.3%	10.9%
Xiong Y 2017	0.72	0.2404	2.06	[1.29; 3.30]	15.2%	17.4%
Sim SH 2016	0.63	0.1828	1.88	[1.31; 2.69]	26.2%	25.1%
Liu ZL 2016	0.25	0.1556	1.29	[0.95; 1.75]	36.2%	30.0%
Yao Y 2013	0.59	0.2492	1.81	[1.11; 2.95]	14.1%	16.5%
**Pooled HR by Fixed Effects**			**1.69**	**[1.41; 2.03]**	**100.0%**	**--**
**Pooled HR by Random Effects**			**1.75**	**[1.38; 2.20]**	**--**	**100.0%**
**Prediction interval**				**[0.95; 3.20]**		

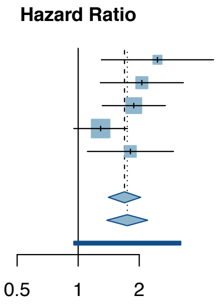

Heterogeneity: $I^2 = 26\%$ [0%; 71%], $\chi^2_4 = 5.44$ ($p = 0.24$)

Tau²= 0.150

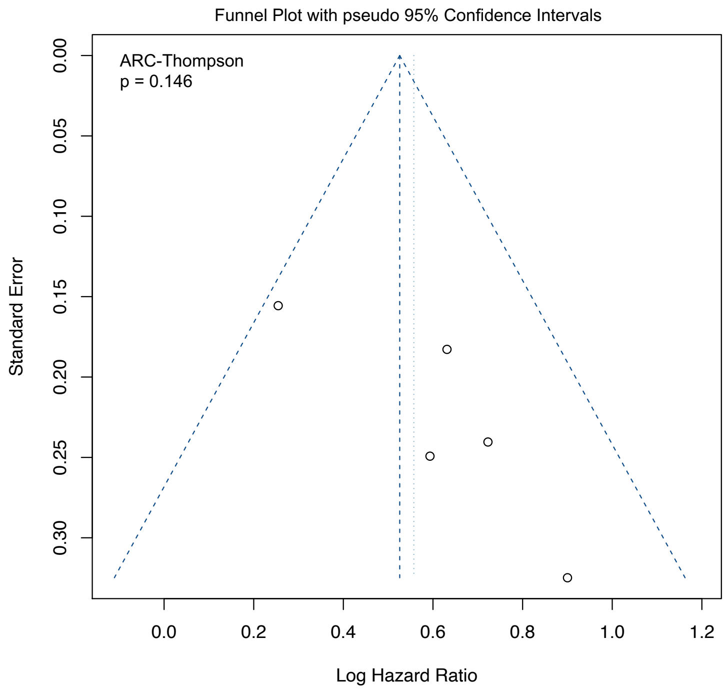

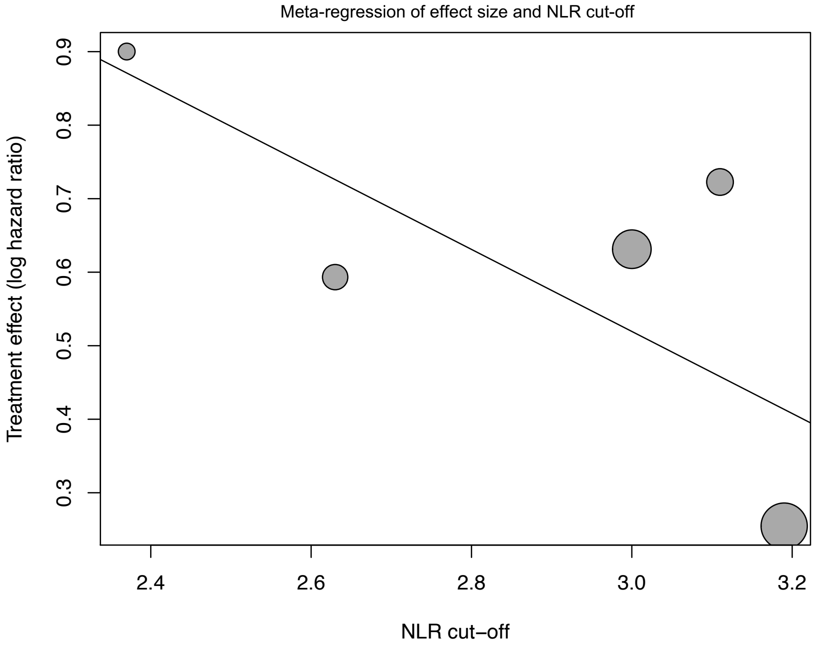

Supplementary Figure 152 - NLR: OS in NSCLC (TT)

Study	TE	seTE	HR	95%−CI	Weight (fixed)	Weight (random)
Aguiar−Bujanda D 2018	1.01	0.4016	2.74	[1.25; 6.02]	0.6%	16.3%
Zhang Y 2018	0.71	0.3201	2.04	[1.09; 3.82]	1.0%	19.9%
Minami S 2017	0.07	0.0323	1.07	[1.00; 1.14]	97.2%	32.3%
Meriggi F 2017	0.99	0.4192	2.70	[1.19; 6.14]	0.6%	15.6%
Chen YM 2016	0.85	0.4111	2.35	[1.05; 5.26]	0.6%	15.9%
**Pooled HR by Fixed Effects**			**1.09**	**[1.03; 1.16]**	**100.0%**	**--**
**Pooled HR by Random Effects**			**1.86**	**[1.18; 2.91]**	**--**	**100.0%**
**Prediction interval**				**[0.43; 8.08]**		

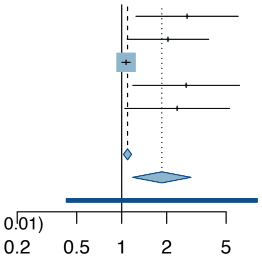

Heterogeneity: $I^2 = 77\%$ [45%; 91%], $\chi^2_4 = 17.59$ ($p < 0.01$)

Tau²= 0.402

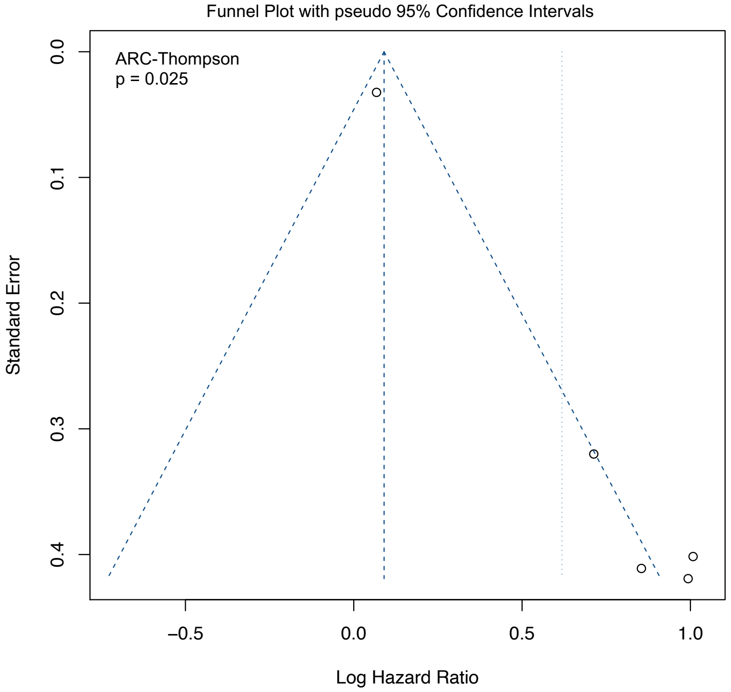

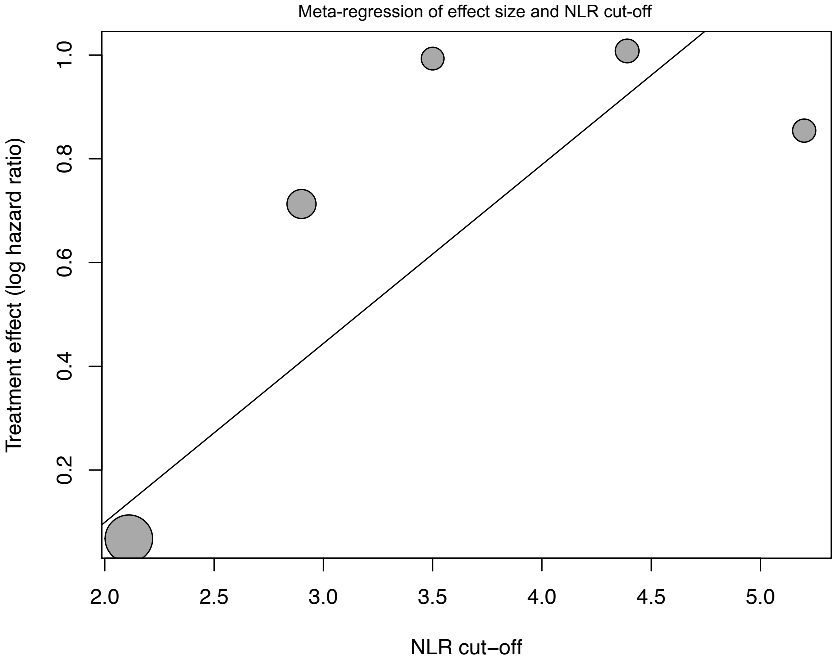

Supplementary Figure 153 - NLR: PFS in NSCLC (TT)

Study	TE	seTE	HR	95%-CI	Weight (fixed)	Weight (random)
Phan TT 2018	0.77	0.3155	2.15	[1.16; 3.99]	1.1%	15.9%
Zhang Y 2018	0.56	0.2647	1.75	[1.04; 2.94]	1.5%	18.7%
Minami S 2017	0.03	0.0335	1.03	[0.96; 1.10]	95.0%	31.8%
Meriggi F 2017	0.82	0.3019	2.28	[1.26; 4.12]	1.2%	16.6%
Sim SH 2016	0.22	0.2948	1.24	[0.70; 2.21]	1.2%	17.0%
**Pooled HR by Fixed Effects**			**1.06**	**[0.99; 1.13]**	**100.0%**	**--**
**Pooled HR by Random Effects**			**1.51**	**[1.06; 2.13]**	**--**	**100.0%**
**Prediction interval**				**[0.48; 4.70]**		

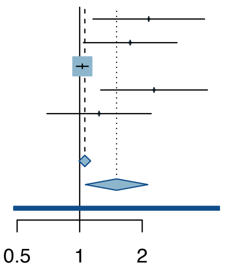

Heterogeneity: $I^2 = 75\%$ [39%; 90%], $\chi^2_4 = 16.06$ ($p < 0.01$)

Tau²= 0.311

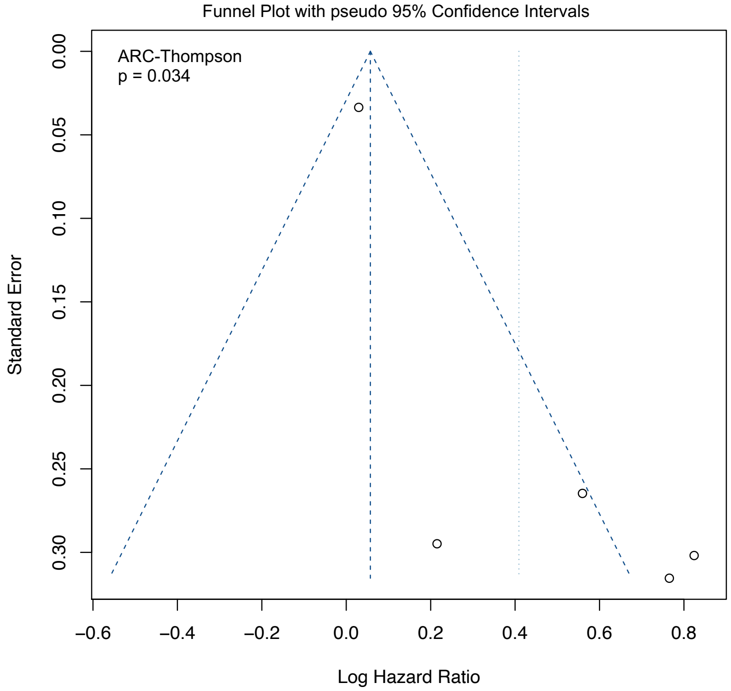

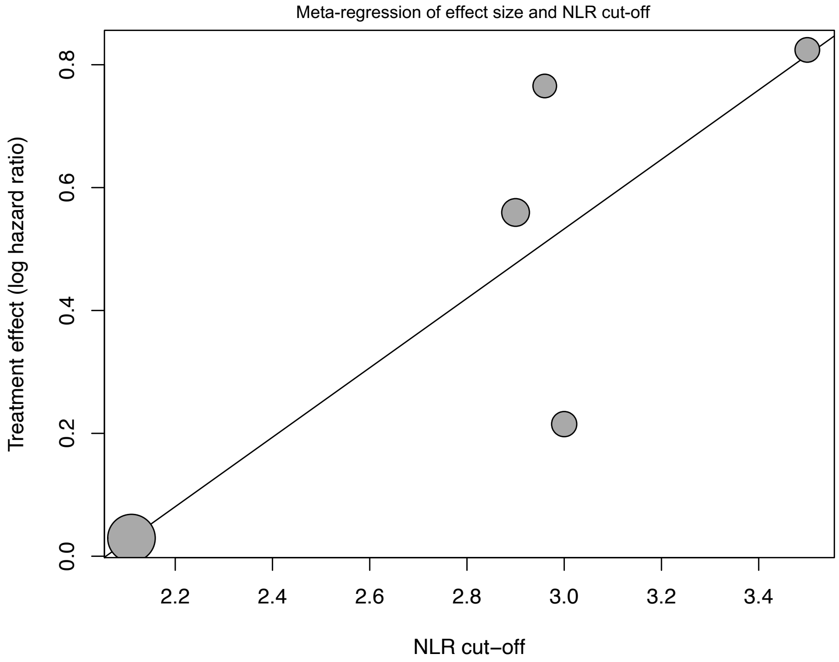

Supplementary Figure 154 - NLR: OS in NSCLC (Nivoumab)

Study	TE	seTE	HR	95%–CI	Weight (fixed)	Weight (random)
Aixa 2017	0.88	0.3953	2.41	[1.11; 5.23]	0.3%	7.2%
Bagley 2017	0.73	0.2379	2.07	[1.30; 3.30]	0.8%	12.4%
Diem 2017	0.96	0.7194	2.60	[0.63; 10.65]	0.1%	2.9%
Kiriu 2018	0.51	0.0821	1.66	[1.41; 1.95]	6.8%	19.3%
Park 2018	0.59	0.2134	1.81	[1.19; 2.75]	1.0%	13.4%
Patil 2017	0.09	0.0229	1.09	[1.04; 1.14]	87.7%	20.7%
Rogado 2017	1.48	0.4285	4.40	[1.90; 10.19]	0.3%	6.5%
Suh 2018	0.41	0.1226	1.51	[1.19; 1.92]	3.1%	17.6%
**Pooled HR by Fixed Effects**			**1.15**	**[1.10; 1.20]**	**100.0%**	**--**
**Pooled HR by Random Effects**			**1.72**	**[1.33; 2.23]**	**--**	**100.0%**
**Prediction interval**				**[0.80; 3.74]**		

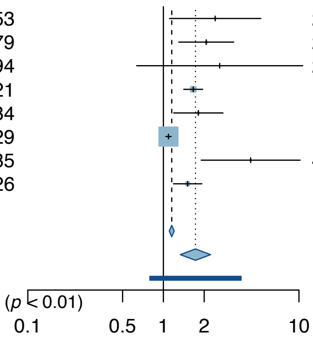

Heterogeneity: $I^2 = 87\%$ [77%; 93%], $\chi^2_7 = 55.59$ ($p < 0.01$)

Tau²= 0.288

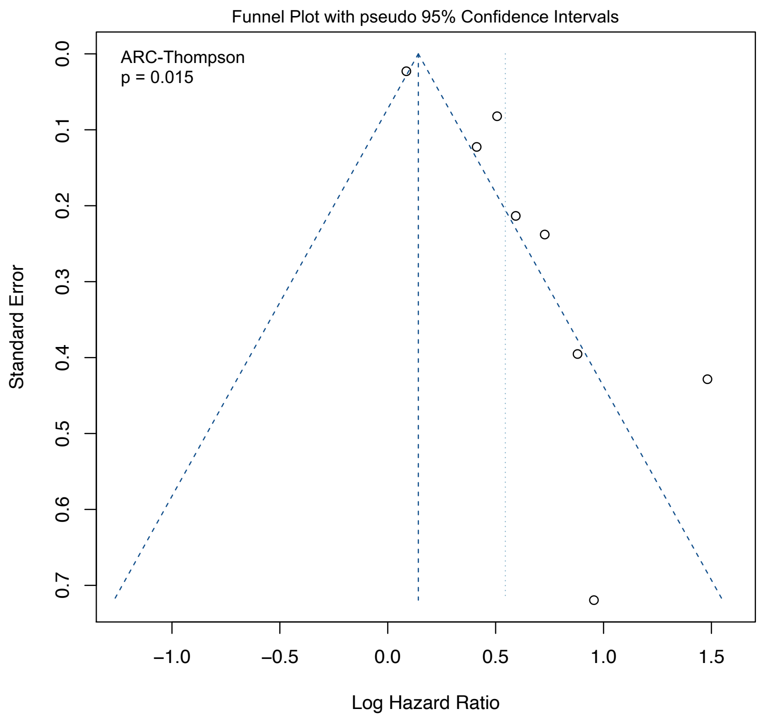

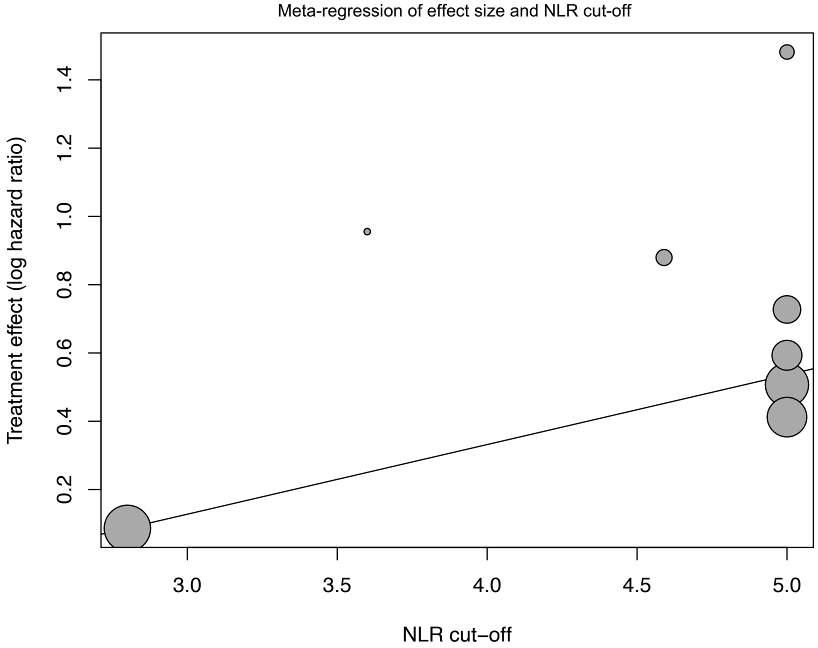

Supplementary Figure 155 - NLR: PFS in NSCLC (Nivoumab)

Study	TE	seTE	HR	95%-CI	Weight (fixed)	Weight (random)
Aixa 2017	0.73	0.3246	2.08	[1.10; 3.93]	0.6%	7.5%
Bagleya 2017	0.36	0.1712	1.43	[1.02; 2.00]	2.2%	10.5%
Diem 2017	0.74	0.2746	2.09	[1.22; 3.58]	0.9%	8.4%
Galetta 2017	0.13	0.0304	1.14	[1.07; 1.21]	70.3%	12.3%
Kiriu 2018	0.57	0.0950	1.76	[1.46; 2.12]	7.2%	11.7%
Nakaya 2018	−0.01	0.0896	0.99	[0.83; 1.18]	8.1%	11.8%
Park 2018	0.52	0.2109	1.68	[1.11; 2.54]	1.5%	9.7%
Rogado 2017	1.90	0.4273	6.70	[2.90; 15.48]	0.4%	5.8%
Shiroyama 2018	0.38	0.1631	1.46	[1.06; 2.01]	2.4%	10.6%
Suh 2018	−0.19	0.1001	0.83	[0.68; 1.01]	6.5%	11.7%
**Pooled HR by Fixed Effects**			**1.18**	**[1.12; 1.24]**	**100.0%**	**--**
**Pooled HR by Random Effects**			**1.51**	**[1.15; 2.00]**	**--**	**100.0%**
**Prediction interval**				**[0.57; 4.04]**		

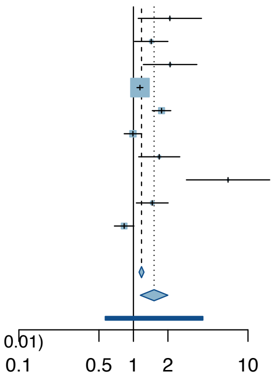

Heterogeneity: I^2 = 86% [76%; 92%], χ^2_9 = 64.86 (p < 0.01)

Tau²= 0.401

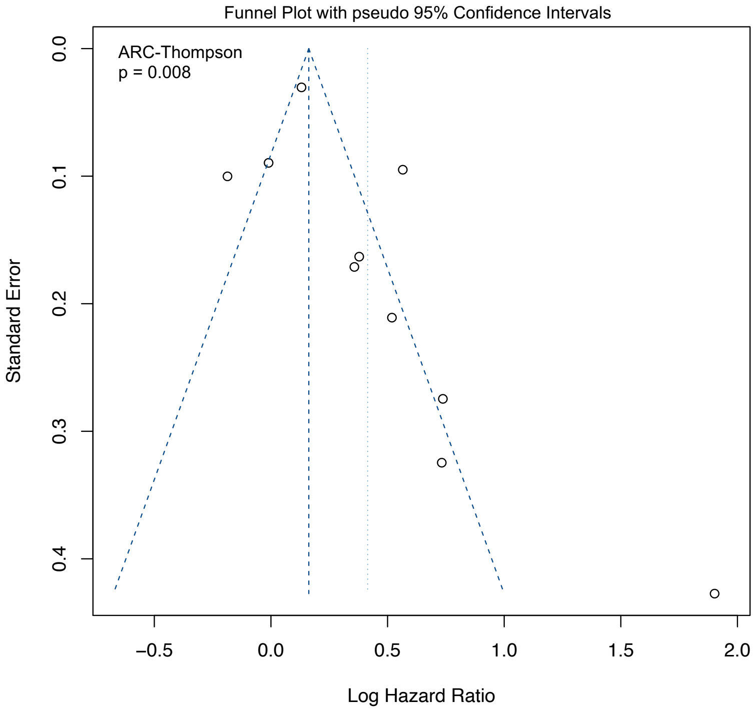

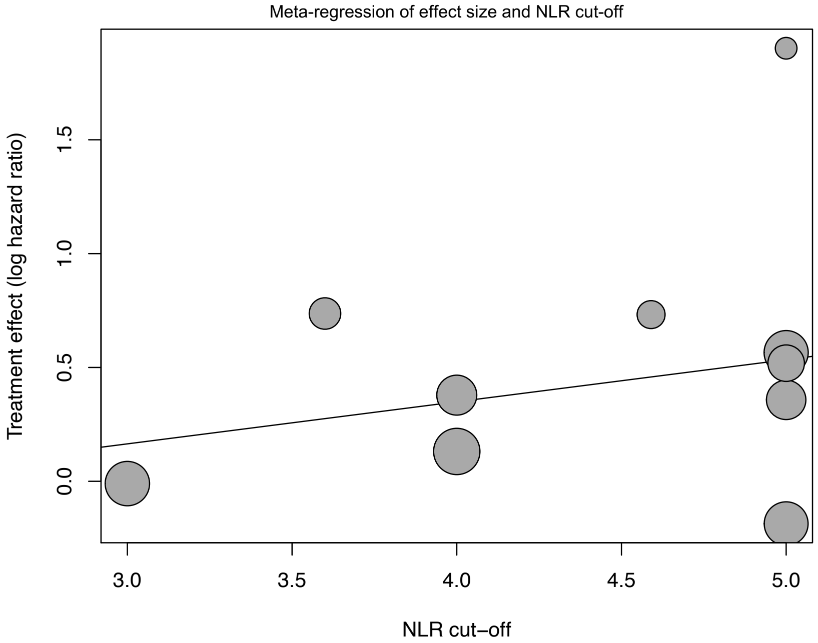

Supplementary Figure 156 - NLR: OS in NSCLC (PD-1 inhibitor)

Study	TE	seTE	HR	95%–CI	Weight (fixed)	Weight (random)
Soyano 2017	0.88	0.3963	2.41	[1.11; 5.24]	4.4%	5.7%
Rogado 2017	1.48	0.3763	4.40	[2.10; 9.20]	4.9%	6.2%
Suh 2018	1.34	0.4468	3.82	[1.59; 9.17]	3.4%	4.7%
Bagley 2017	0.73	0.2379	2.07	[1.30; 3.30]	12.2%	11.7%
Diem 2017	1.61	0.4611	5.01	[2.03; 12.37]	3.2%	4.5%
Park 1 2017	1.11	0.3097	3.03	[1.65; 5.56]	7.2%	8.3%
Park 2 2017	1.24	0.3032	3.45	[1.90; 6.25]	7.5%	8.6%
Shiroyama 2018	0.82	0.2944	2.28	[1.28; 4.06]	7.9%	8.9%
Mezquita 1 2018	0.53	0.1498	1.70	[1.27; 2.28]	30.7%	18.0%
Mezquita 2 2018	0.80	0.3017	2.22	[1.23; 4.01]	7.6%	8.6%
Fukui 2018	1.43	0.5770	4.17	[1.35; 12.92]	2.1%	3.0%
Zer 2018	0.80	0.3432	2.22	[1.13; 4.35]	5.8%	7.2%
Facchinetti 2018	1.17	0.4637	3.22	[1.30; 7.99]	3.2%	4.4%
**Pooled HR by Fixed Effects**			**2.39**	**[2.03; 2.81]**	**100.0%**	**--**
**Pooled HR by Random Effects**			**2.59**	**[2.10; 3.20]**	**--**	**100.0%**
**Prediction interval**				**[1.57; 4.28]**		

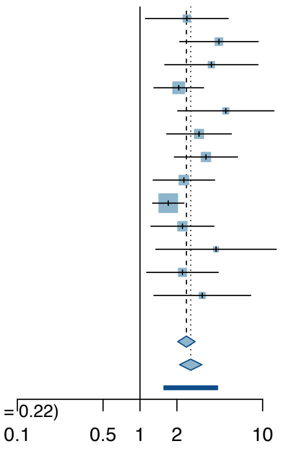

Heterogeneity: $I^2 = 22\%$ [0%; 59%], $\chi^2_{12} = 15.38$ ($p = 0.22$)

Tau²= 0.202

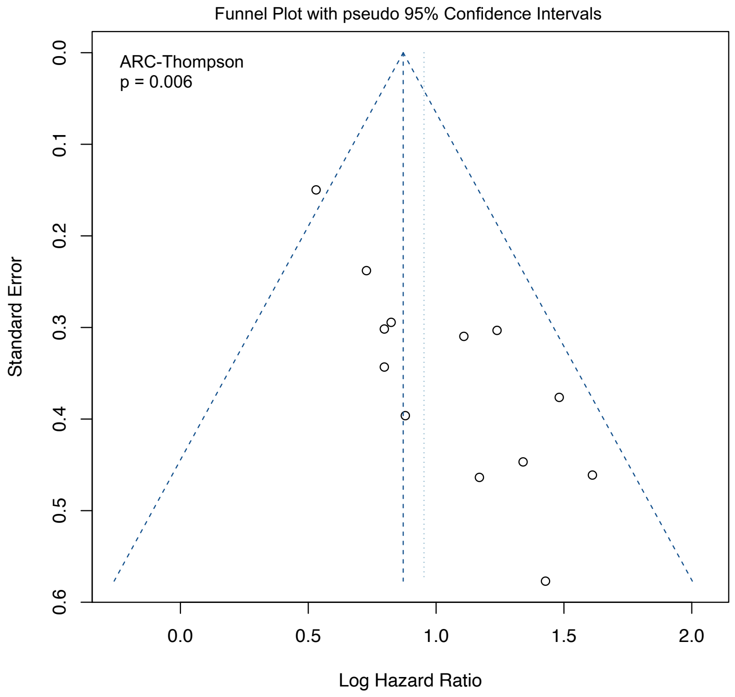

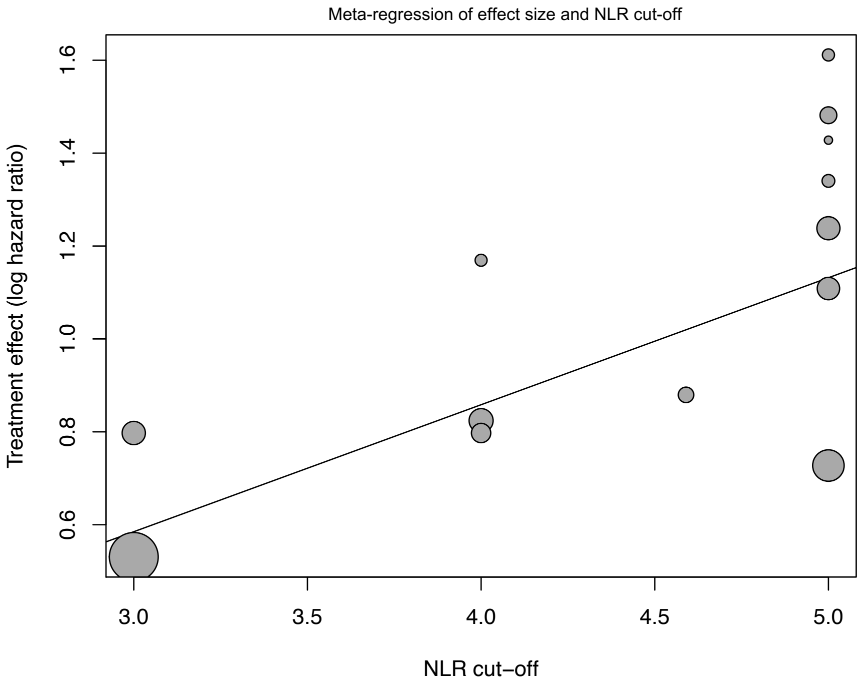

Supplementary Figure 157 - NLR: PFS in NSCLC (PD-1 inhibitor)

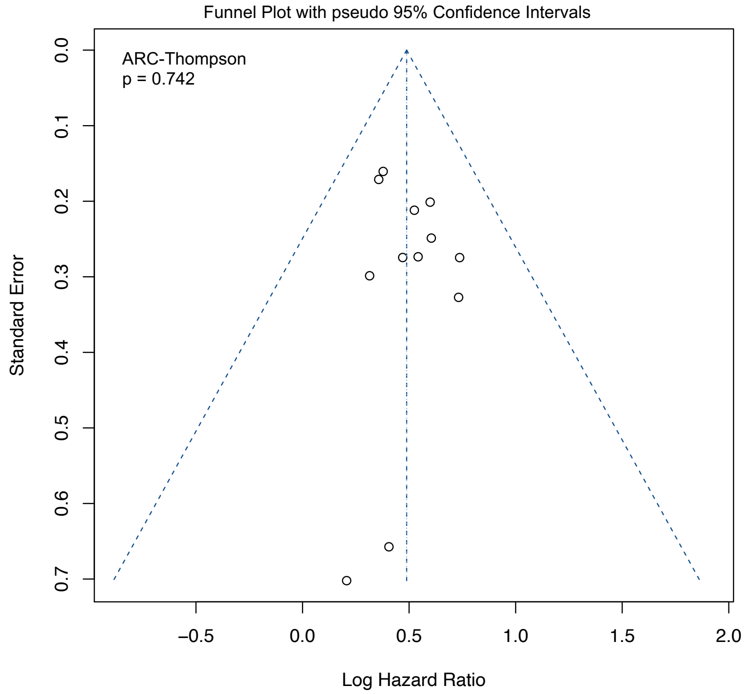

Study	TE	seTE	HR	95%–CI	Weight (fixed)	Weight (random)
Soyano 2017	0.73	0.3272	2.08	[1.10; 3.95]	4.7%	4.7%
Bagley 2017	0.36	0.1712	1.43	[1.02; 2.00]	17.0%	17.0%
Diem 2017	0.74	0.2746	2.09	[1.22; 3.58]	6.6%	6.6%
Park 1 2017	0.52	0.2119	1.69	[1.12; 2.56]	11.1%	11.1%
Park 2 2017	0.60	0.2012	1.82	[1.23; 2.70]	12.3%	12.3%
Nakaya 1 2018	0.47	0.2745	1.60	[0.93; 2.74]	6.6%	6.6%
Nakaya 2 2018	0.31	0.2986	1.37	[0.76; 2.46]	5.6%	5.6%
Shiroyama 2018	0.38	0.1606	1.46	[1.07; 2.00]	19.3%	19.3%
Mezquita 2018	0.60	0.2488	1.83	[1.12; 2.98]	8.0%	8.0%
Zer 2018	0.54	0.2735	1.72	[1.01; 2.94]	6.7%	6.7%
Takeda 1 2018	0.21	0.7021	1.23	[0.31; 4.87]	1.0%	1.0%
Takeda 2 2018	0.41	0.6573	1.50	[0.41; 5.44]	1.2%	1.2%
**Pooled HR by Fixed Effects**			**1.63**	**[1.42; 1.87]**	**100.0%**	**--**
**Pooled HR by Random Effects**			**1.63**	**[1.42; 1.87]**	**--**	**100.0%**
**Prediction interval**				**[1.39; 1.91]**		

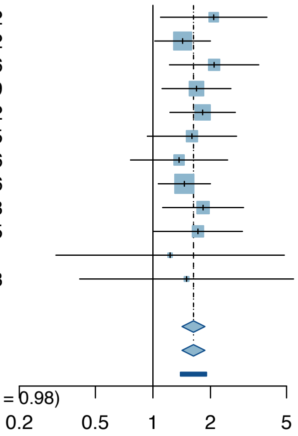

Heterogeneity: I^2 = 0% [<0%; <0%], χ^2_{11} = 3.53 (p = 0.98)

Tau² = 0

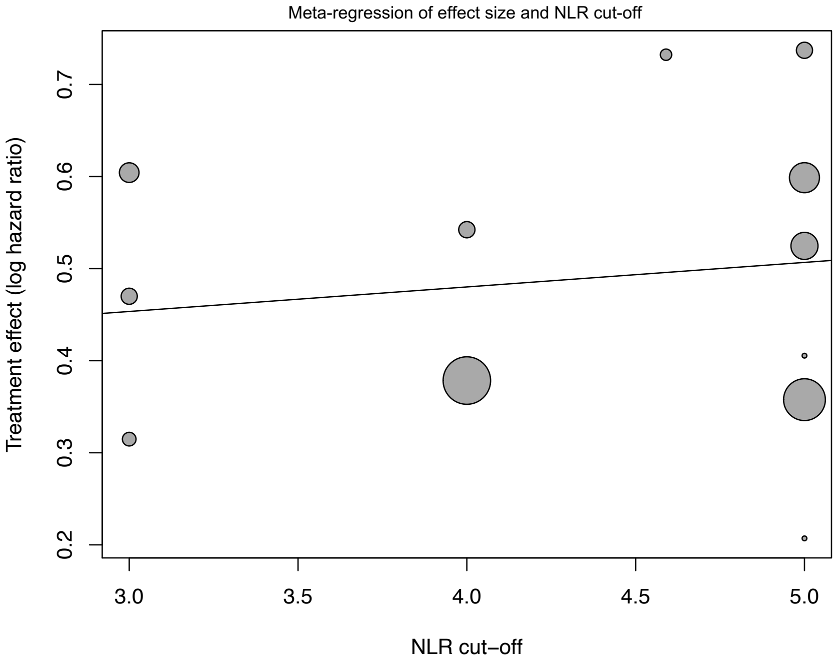

Supplementary Figure 158 - NLR: OS in NSCLC (ST)

Study	TE	seTE	HR	95%–CI	Weight (fixed)	Weight (random)
Bozkaya Y 2019	0.29	0.2069	1.34	[0.89; 2.01]	0.6%	4.9%
Guo D 2019	0.67	0.2622	1.95	[1.17; 3.26]	0.4%	4.4%
Yi F 2018	1.33	0.4651	3.77	[1.52; 9.38]	0.1%	2.7%
Minami S 2018	0.31	0.3629	1.37	[0.67; 2.79]	0.2%	3.5%
Minami S2 2018	0.09	0.2502	1.09	[0.67; 1.78]	0.4%	4.5%
Xiong Y 2017	1.71	0.3174	5.54	[2.97; 10.32]	0.2%	3.9%
Shiran I 2017	0.06	0.0326	1.06	[0.99; 1.13]	23.1%	6.1%
Liu ZL 2016	0.52	0.1329	1.68	[1.29; 2.18]	1.4%	5.6%
Yao Y 2013	0.57	0.2423	1.76	[1.09; 2.83]	0.4%	4.6%
Kiriu T 2019	1.44	0.4646	4.20	[1.69; 10.44]	0.1%	2.7%
Soyano AE 2018	0.63	0.2446	1.87	[1.16; 3.02]	0.4%	4.6%
Naqash AR 2018	1.05	0.3146	2.85	[1.54; 5.28]	0.2%	3.9%
Fukui T 2019	1.43	0.5770	4.17	[1.35; 12.92]	0.1%	2.1%
Facchinetti F 2018	1.17	0.4637	3.22	[1.30; 7.99]	0.1%	2.7%
Diem S 2017	1.61	0.4611	5.01	[2.03; 12.37]	0.1%	2.7%
Bagley SJ 2017	0.73	0.2379	2.07	[1.30; 3.30]	0.4%	4.6%
Mitchell P 2015	0.11	0.0915	1.12	[0.94; 1.34]	2.9%	5.9%
Aguiar–Bujanda D 2018	1.01	0.4016	2.74	[1.25; 6.02]	0.2%	3.2%
Zhang Y 2018	0.71	0.3201	2.04	[1.09; 3.82]	0.2%	3.8%
Minami S 2017	0.07	0.0323	1.07	[1.00; 1.14]	23.5%	6.1%
Meriggi F 2017	0.99	0.4192	2.70	[1.19; 6.14]	0.1%	3.0%
Chen YM 2016	0.85	0.4111	2.35	[1.05; 5.26]	0.1%	3.1%
Berardi R 2016	0.55	0.1662	1.74	[1.26; 2.41]	0.9%	5.3%
Lee Y 2012	0.05	0.0237	1.05	[1.00; 1.10]	43.7%	6.1%
**Pooled HR by Fixed Effects**			**1.10**	**[1.07; 1.14]**	**100.0%**	**--**
**Pooled HR by Random Effects**			**1.87**	**[1.53; 2.29]**	**--**	**100.0%**
**Prediction interval**				**[0.77; 4.52]**		

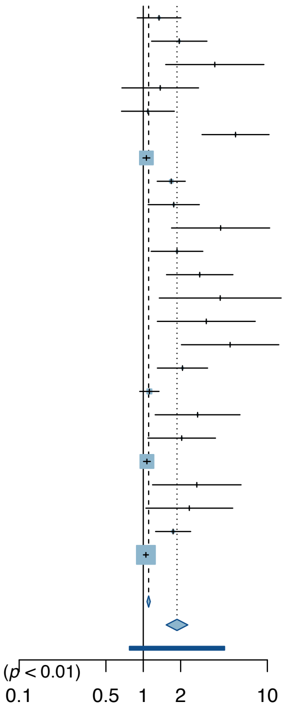

Heterogeneity: $I^2 = 83\%$ [75%; 88%], $\chi^2_{23} = 134.01$ ($p < 0.01$)

Tau²= 0.413

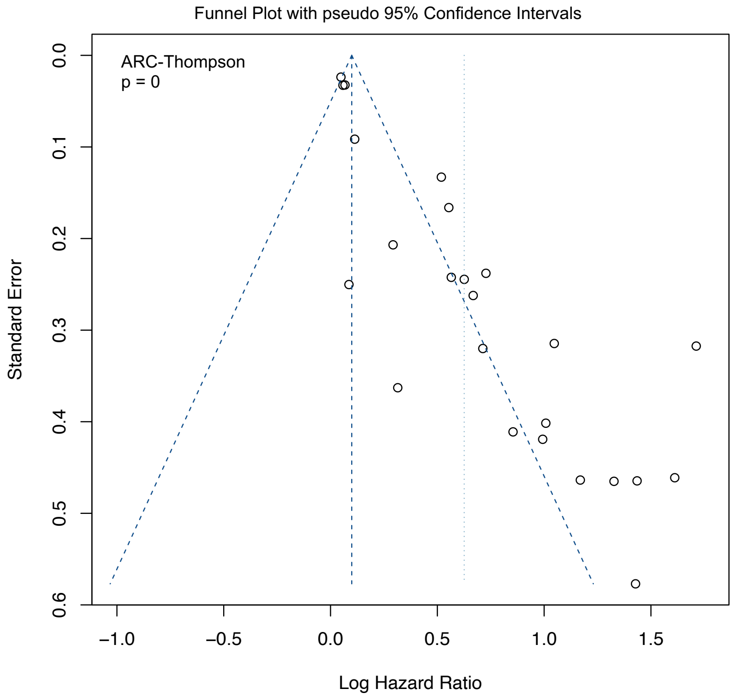

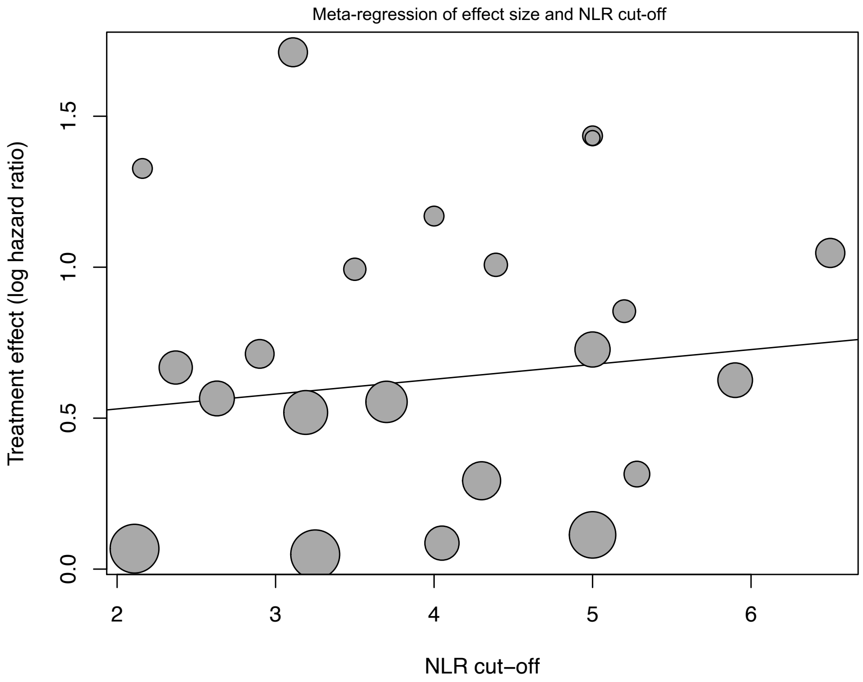

Supplementary Figure 159 - NLR: PFS in NSCLC (ST)

Study	TE	seTE	HR	95%–CI	Weight (fixed)	Weight (random)
Phan TT 2018	0.77	0.3155	2.15	[1.16; 3.99]	0.4%	3.8%
Zhang Y 2018	0.56	0.2647	1.75	[1.04; 2.94]	0.6%	4.7%
Minami S 2017	0.03	0.0335	1.03	[0.96; 1.10]	37.1%	10.8%
Meriggi F 2017	0.82	0.3019	2.28	[1.26; 4.12]	0.5%	4.0%
Sim SH 2016	0.22	0.2948	1.24	[0.70; 2.21]	0.5%	4.1%
Guo D 2019	0.90	0.3248	2.46	[1.30; 4.65]	0.4%	3.7%
Xiong Y 2017	0.72	0.2404	2.06	[1.29; 3.30]	0.7%	5.2%
Sim SH 2016	0.63	0.1828	1.88	[1.31; 2.69]	1.3%	6.7%
Liu ZL 2016	0.25	0.1556	1.29	[0.95; 1.75]	1.7%	7.5%
Yao Y 2013	0.59	0.2492	1.81	[1.11; 2.95]	0.7%	5.0%
Soyano AE 2018	0.48	0.1775	1.61	[1.14; 2.28]	1.3%	6.9%
Shiroyama T 2018	0.30	0.1824	1.35	[0.94; 1.93]	1.3%	6.7%
Diem S 2017	0.74	0.2746	2.09	[1.22; 3.58]	0.6%	4.5%
Bagley SJ 2017	0.36	0.1712	1.43	[1.02; 2.00]	1.4%	7.1%
Berardi R 2016	0.31	0.1315	1.36	[1.05; 1.76]	2.4%	8.3%
Lee Y 2012	0.02	0.0292	1.02	[0.96; 1.08]	49.2%	10.8%
**Pooled HR by Fixed Effects**			**1.09**	**[1.04; 1.13]**	**100.0%**	**--**
**Pooled HR by Random Effects**			**1.50**	**[1.29; 1.74]**	**--**	**100.0%**
**Prediction interval**				**[0.89; 2.51]**		

Heterogeneity: $I^2 = 78\%$ [64%; 86%], $\chi^2_{15} = 66.69$ ($p < 0.01$)

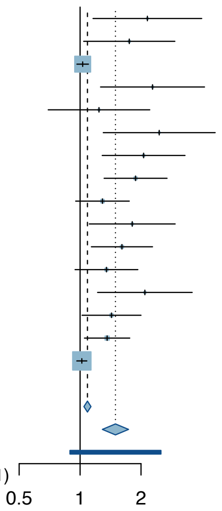

Tau²= 0.229

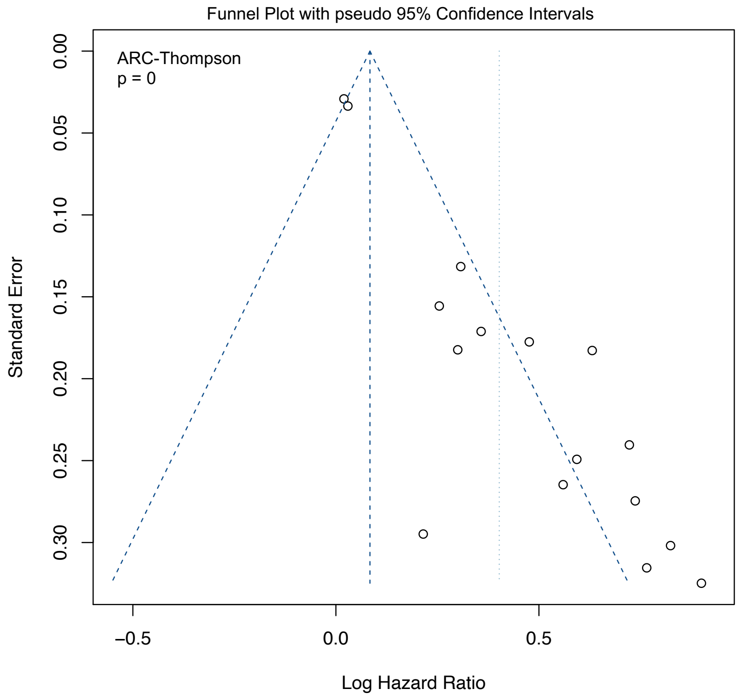

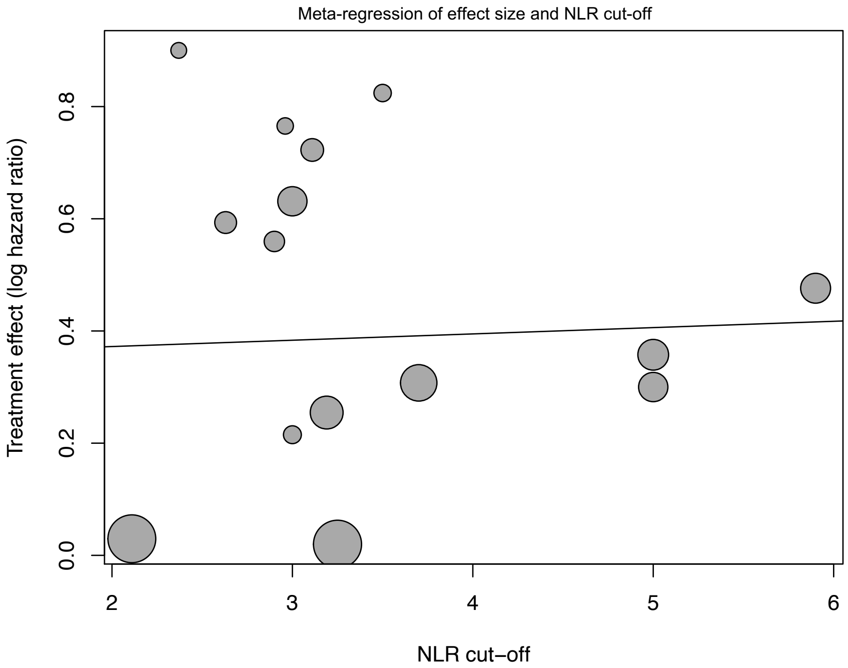

Supplementary Figure 160 - NLR: OS in Head and Neck

Study	TE	seTE	Hazard Ratio	HR	95%–CI	Weight (fixed)	Weight (random)
**Adjustment = Multivariate**							
Bobdey 2016	0.33	0.1465		1.39	[1.04; 1.85]	12.1%	8.5%
Charles 1 2016	1.53	0.6609		4.60	[1.26; 16.80]	0.6%	0.9%
Charles 2 2016	1.29	0.5089		3.64	[1.34; 9.87]	1.0%	1.5%
Chen 2016	0.66	0.2664		1.94	[1.15; 3.27]	3.7%	4.3%
Chua 2016	0.06	0.1737		1.06	[0.75; 1.49]	8.6%	7.2%
Fu 2016	0.27	0.1360		1.31	[1.00; 1.71]	14.1%	9.0%
He 2011	0.45	0.2144		1.57	[1.03; 2.39]	5.7%	5.7%
Sun 2016	0.63	0.3815		1.87	[0.89; 3.95]	1.8%	2.5%
Ikeguchi 2016	1.72	0.7978		5.59	[1.17; 26.68]	0.4%	0.6%
Kano 2016	0.30	0.2460		1.35	[0.83; 2.18]	4.3%	4.8%
Kawakita 2016	0.59	0.2741		1.80	[1.05; 3.08]	3.5%	4.1%
Kim 2016	0.42	0.2699		1.52	[0.90; 2.58]	3.6%	4.2%
Moon 2016	1.17	0.4027		3.22	[1.46; 7.09]	1.6%	2.3%
Ong 2017	0.46	0.2819		1.58	[0.91; 2.75]	3.3%	4.0%
Rachidi 2016	0.87	0.1990		2.39	[1.62; 3.53]	6.6%	6.2%
Rosculet 2017	1.12	0.8228		3.05	[0.61; 15.30]	0.4%	0.6%
Selzer 2015	0.62	0.2512		1.85	[1.13; 3.03]	4.1%	4.7%
Song 2015	0.86	0.2917		2.36	[1.33; 4.18]	3.1%	3.8%
Tu 2015	0.78	0.3006		2.18	[1.21; 3.92]	2.9%	3.6%
Turri–Zanoni 2016	0.77	0.3778		2.17	[1.03; 4.55]	1.8%	2.5%
Wong 2016	1.12	0.5314		3.06	[1.08; 8.67]	0.9%	1.4%
Zeng 2016	0.41	0.1920		1.51	[1.04; 2.20]	7.1%	6.5%
Li 2016	0.14	0.2663		1.15	[0.68; 1.94]	3.7%	4.3%
Ma 2014	2.26	0.9950		9.54	[1.36; 67.04]	0.3%	0.4%
**Pooled HR by Fixed Effects**				**1.62**	**[1.46; 1.79]**	**95.1%**	**--**
**Pooled HR by Random Effects**				**1.71**	**[1.49; 1.96]**	**--**	**93.6%**
Heterogeneity: $I^2 = 35\%$ [0%; 61%], $\chi^2_{23} = 35.52$ ($p = 0.05$)							
**Adjustment = Univariate**							
Wang 2016	0.69	0.3083		1.99	[1.09; 3.65]	2.7%	3.5%
Fang 2013	0.71	0.3453		2.04	[1.04; 4.01]	2.2%	2.9%
**Pooled HR by Fixed Effects**				**2.01**	**[1.28; 3.16]**	**4.9%**	**--**
**Pooled HR by Random Effects**				**2.01**	**[1.28; 3.16]**	**--**	**6.4%**
Heterogeneity: $I^2 = 0\%$, $\chi^2_1 = 0$ ($p = 0.96$)							
**Pooled HR by Fixed Effects**				**1.63**	**[1.48; 1.81]**	**100.0%**	**--**
**Pooled HR by Random Effects**				**1.72**	**[1.51; 1.96]**	**--**	**100.0%**
**Prediction interval**					**[1.18; 2.52]**		
Heterogeneity: $I^2 = 31\%$ [0%; 58%], $\chi^2_{25} = 36.40$ ($p = 0.07$)							
Residual heterogeneity: $I^2 = 32\%$ [0%; 59%], $\chi^2_{24} = 35.52$ ($p = 0.06$)							

Hazard Ratio axis: 0.1 0.5 1 2 10

Tau^2= 0.172

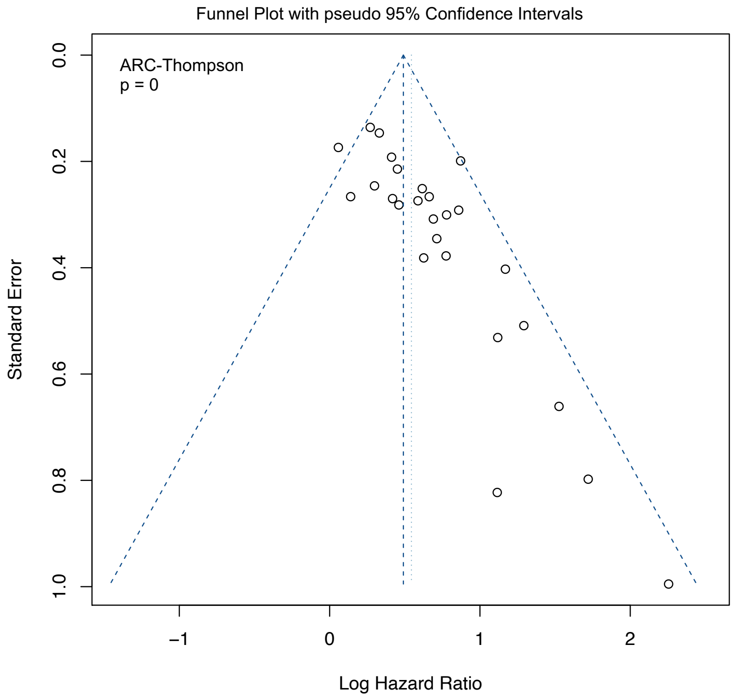

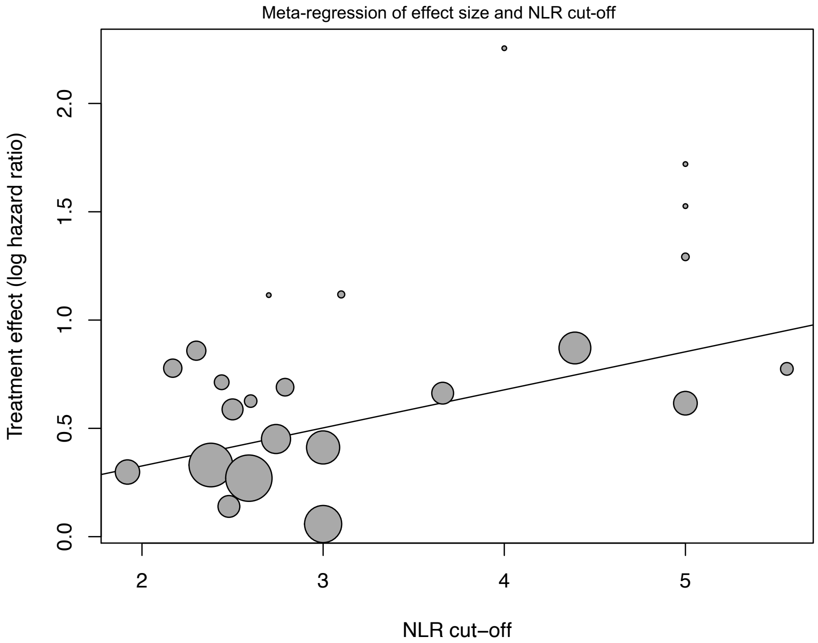

Supplementary Figure 161 - NLR: PFS in Head and neck

Study	TE	seTE	HR	95%–CI	Weight (fixed)	Weight (random)
He 2011	0.52	0.1777	1.68	[1.19; 2.38]	20.9%	19.7%
Sun 2016	0.70	0.2514	2.01	[1.23; 3.29]	10.5%	14.2%
Kawakita 2016	0.00	0.2366	1.00	[0.63; 1.59]	11.8%	15.1%
Kim 2016	0.11	0.1387	1.12	[0.85; 1.47]	34.3%	23.3%
Moon 2016	0.79	0.3407	2.20	[1.13; 4.29]	5.7%	9.6%
Zeng 2016	0.58	0.1982	1.79	[1.21; 2.64]	16.8%	18.0%
**Pooled HR by Fixed Effects**			**1.44**	**[1.23; 1.69]**	**100.0%**	**--**
**Pooled HR by Random Effects**			**1.50**	**[1.18; 1.93]**	**--**	**100.0%**
**Prediction interval**				**[0.74; 3.05]**		

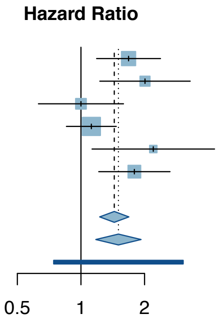

Heterogeneity: $I^2 = 54\%$ [0%; 82%], $\chi^2_5 = 10.92$ ($p = 0.05$)

Tau²= 0.221

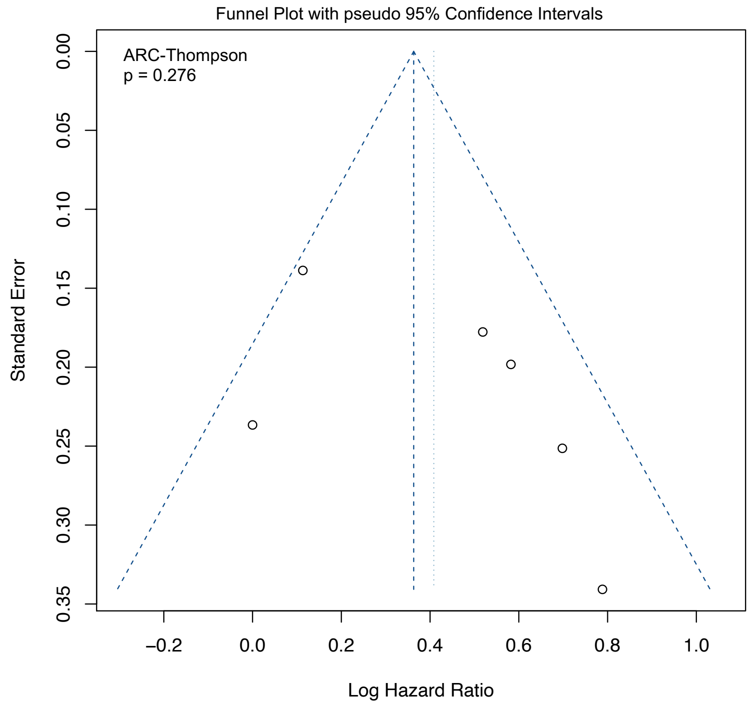

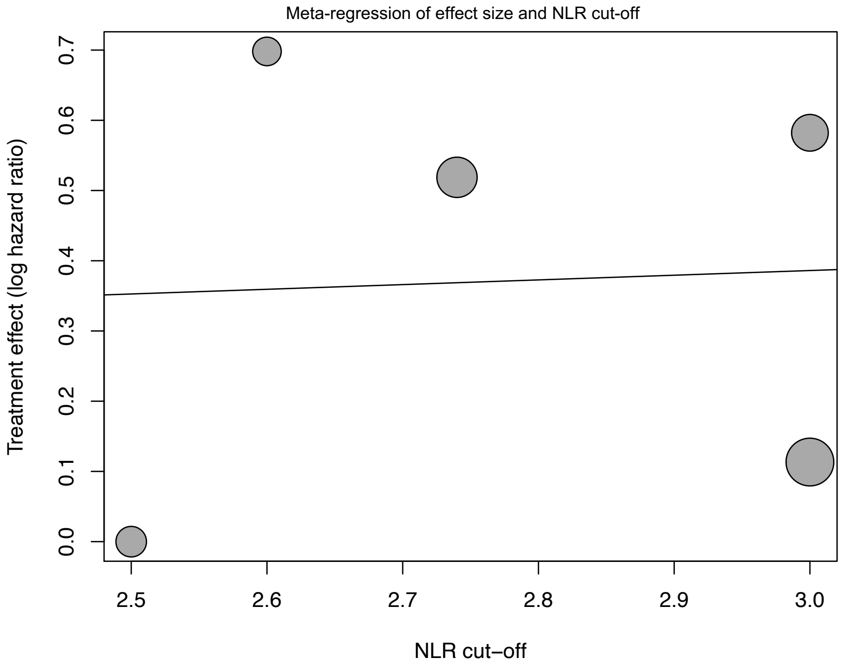

Supplementary Figure 162 - NLR: DFS in Head and neck

Study	TE	seTE	HR	95%–CI	Weight (fixed)	Weight (random)
**Adjustment = Multivariate**						
Charles 1 2016	1.10	0.5266	3.01	[1.07; 8.45]	2.8%	4.5%
Charles 2 2016	0.70	0.4532	2.02	[0.83; 4.91]	3.8%	5.8%
Chua 2016	−0.02	0.1558	0.98	[0.72; 1.33]	32.2%	18.8%
Ong 2017	0.43	0.2423	1.53	[0.95; 2.47]	13.3%	13.1%
Rosculet 2017	0.88	0.6183	2.42	[0.72; 8.13]	2.0%	3.4%
Tu 2015	0.63	0.2812	1.87	[1.08; 3.24]	9.9%	11.1%
Turri–Zanoni 2016	0.94	0.3415	2.56	[1.31; 5.00]	6.7%	8.7%
Wong 2016	0.62	0.3726	1.85	[0.89; 3.84]	5.6%	7.7%
Ma 2014	−0.17	0.6262	0.84	[0.25; 2.88]	2.0%	3.4%
**Pooled HR by Fixed Effects**			**1.43**	**[1.18; 1.74]**	**78.4%**	**--**
**Pooled HR by Random Effects**			**1.64**	**[1.22; 2.21]**	**--**	**76.6%**
Heterogeneity: I^2 = 44% [0%; 74%], χ^2_8 = 14.27 (p = 0.08)						
**Adjustment = Univariate**						
Wang 2016	0.65	0.2814	1.92	[1.11; 3.33]	9.9%	11.1%
Fang 2013	0.54	0.2575	1.72	[1.04; 2.85]	11.8%	12.3%
**Pooled HR by Fixed Effects**			**1.81**	**[1.25; 2.62]**	**21.6%**	**--**
**Pooled HR by Random Effects**			**1.81**	**[1.25; 2.62]**	**--**	**23.4%**
Heterogeneity: I^2 = 0%, χ^2_1 = 0.08 (p = 0.77)						
**Pooled HR by Fixed Effects**			**1.51**	**[1.27; 1.79]**	**100.0%**	**--**
**Pooled HR by Random Effects**			**1.66**	**[1.30; 2.11]**	**--**	**100.0%**
**Prediction interval**				**[0.91; 3.03]**		

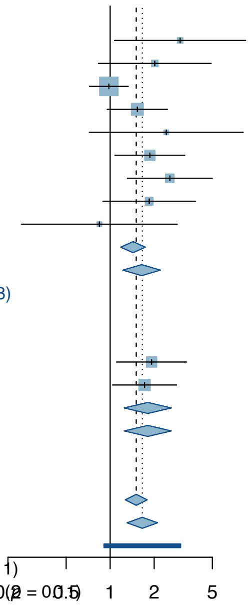

Heterogeneity: I^2 = 36% [0%; 68%], χ^2_{10} = 15.52 (p = 0.11)
Residual heterogeneity: I^2 = 37% [0%; 70%], χ^2_9 = 14.35 (p = 0.15)

Tau²= 0.237

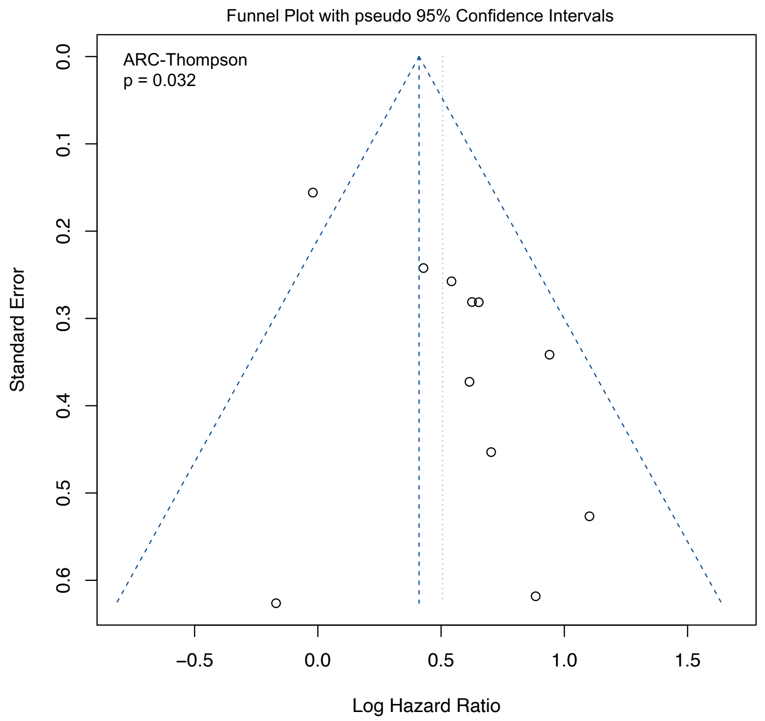

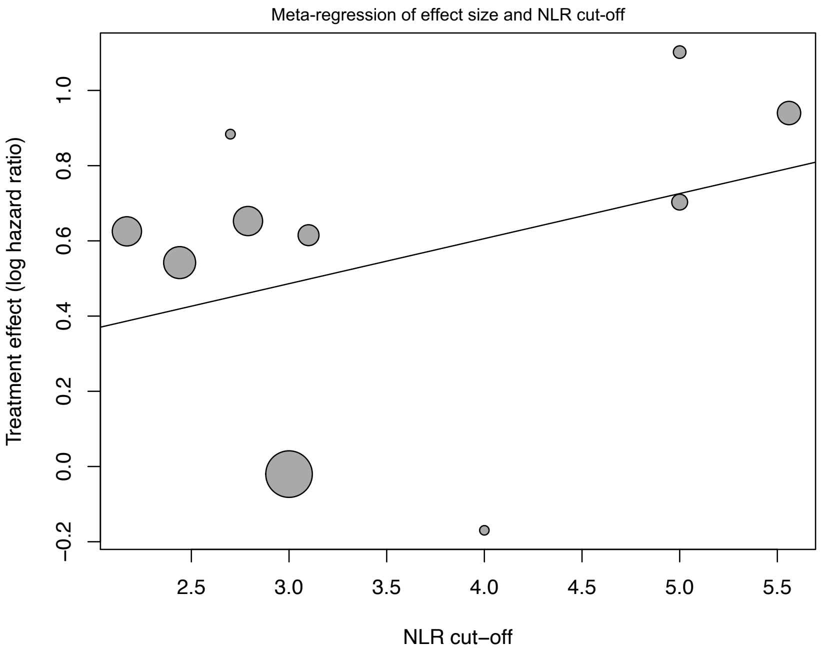

Supplementary Figure 163 - NLR: OS in Head and Neck (SCC)

Study	TE	seTE	HR	95%–CI	Weight (fixed)	Weight (random)
**Adjustment = Multivariate**						
He 2012	0.53	1.2632	1.71	[0.14; 20.30]	0.2%	0.4%
Jin 2014	0.51	0.1351	1.66	[1.28; 2.17]	15.7%	8.2%
Rachidi 2015	0.27	0.3589	1.32	[0.65; 2.66]	2.2%	3.6%
Selzer 2015	0.62	0.2473	1.85	[1.14; 3.01]	4.7%	5.5%
Sun 2015	0.63	0.3801	1.87	[0.89; 3.94]	2.0%	3.3%
Tu 2015	0.78	0.3006	2.18	[1.21; 3.92]	3.2%	4.5%
Charles 1 2016	1.53	0.4300	4.60	[1.98; 10.69]	1.5%	2.8%
Charles 2 2016	1.29	0.5094	3.64	[1.34; 9.88]	1.1%	2.1%
Chua 2016	0.06	0.1717	1.06	[0.76; 1.48]	9.7%	7.3%
Fu 2016	0.28	0.1368	1.32	[1.01; 1.73]	15.3%	8.2%
Ikeguchi 2016	1.72	0.4447	5.59	[2.34; 13.35]	1.4%	2.6%
Kano 2016	0.77	0.1968	2.16	[1.47; 3.18]	7.4%	6.6%
Kim 2016	0.42	0.2496	1.52	[0.93; 2.48]	4.6%	5.4%
Moon 2016	1.17	0.4120	3.22	[1.44; 7.22]	1.7%	3.0%
Nakashima 2016	0.68	0.3716	1.97	[0.95; 4.07]	2.1%	3.4%
Wong 2016	1.06	0.6240	2.88	[0.85; 9.77]	0.7%	1.5%
Zeng 2016	0.41	0.1911	1.51	[1.04; 2.20]	7.8%	6.8%
Turri–Zanoni 2017	0.78	0.3759	2.17	[1.04; 4.54]	2.0%	3.4%
**Pooled HR by Fixed Effects**			**1.67**	**[1.49; 1.88]**	**83.2%**	**--**
**Pooled HR by Random Effects**			**1.86**	**[1.55; 2.24]**	**--**	**78.6%**
Heterogeneity: I^2 = 48% [11%; 70%], χ^2_{17} = 32.83 (p = 0.01)						
**Adjustment = Univariate**						
Song 2015	1.10	0.2280	2.99	[1.91; 4.67]	5.5%	5.9%
Salim 2015	0.65	0.2943	1.92	[1.08; 3.41]	3.3%	4.6%
Millrud 2012	1.69	0.8605	5.44	[1.01; 29.39]	0.4%	0.9%
Fang 2013	0.71	0.3455	2.04	[1.04; 4.01]	2.4%	3.8%
Young 2014	0.58	0.2438	1.79	[1.11; 2.88]	4.8%	5.5%
Haddad 2015	2.11	0.8852	8.27	[1.46; 46.89]	0.4%	0.8%
**Pooled HR by Fixed Effects**			**2.32**	**[1.80; 3.00]**	**16.8%**	**--**
**Pooled HR by Random Effects**			**2.33**	**[1.76; 3.07]**	**--**	**21.4%**
Heterogeneity: I^2 = 16% [0%; 79%], χ^2_5 = 5.98 (p = 0.31)						
**Pooled HR by Fixed Effects**			**1.77**	**[1.59; 1.96]**	**100.0%**	**--**
**Pooled HR by Random Effects**			**1.96**	**[1.66; 2.31]**	**--**	**100.0%**
**Prediction interval**				**[1.11; 3.45]**		

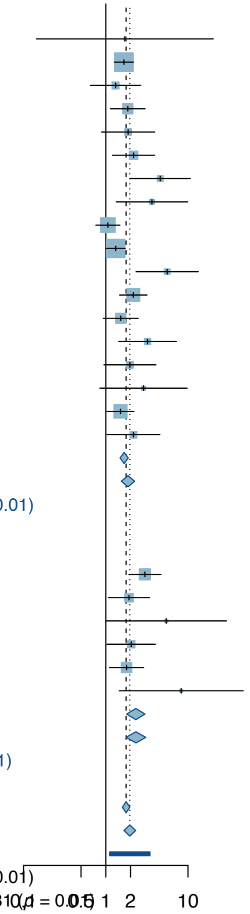

Heterogeneity: I^2 = 48% [16%; 68%], χ^2_{23} = 44.01 ($p < 0.01$)
Residual heterogeneity: I^2 = 43% [7%; 65%], χ^2_{22} = 38.81 (p = 0.01)

Tau²= 0.259

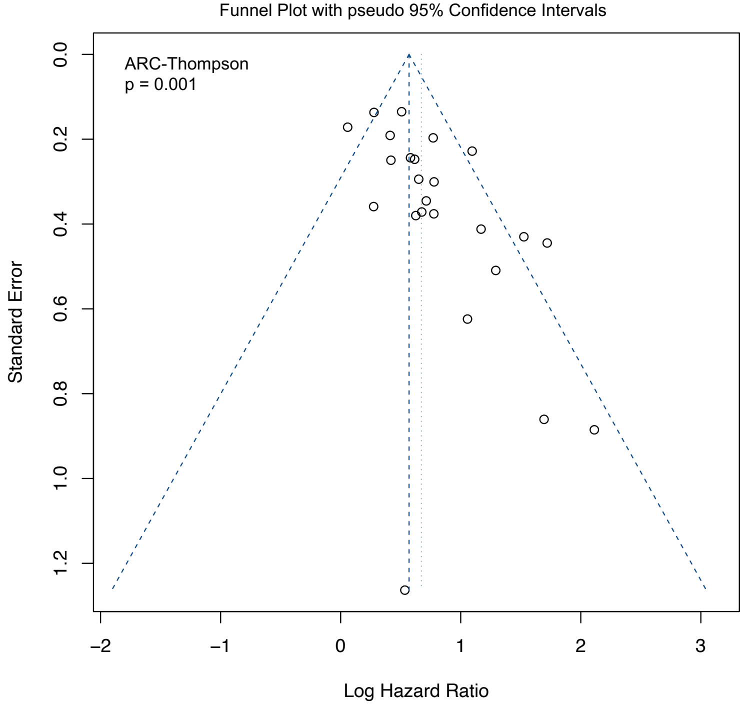

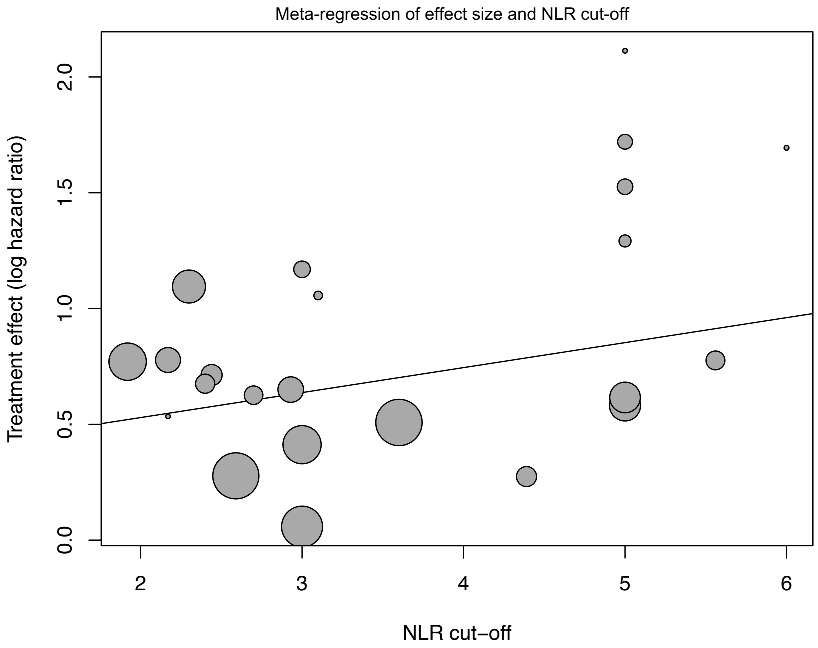

Supplementary Figure 164 - NLR: DFS in Head and Neck (SCC)

Study	TE	seTE	HR	95%-CI	Weight (fixed)	Weight (random)
**Adjustment = Multivariate**						
Tu 2015	0.63	0.2810	1.87	[1.08; 3.24]	5.6%	9.8%
Charles 1 2016	1.10	0.5272	3.01	[1.07; 8.46]	1.6%	5.2%
Charles 2 2016	0.70	0.4535	2.02	[0.83; 4.91]	2.2%	6.3%
Chua 2016	−0.02	0.1531	0.98	[0.73; 1.32]	19.0%	12.9%
Kano 2016	0.68	0.1743	1.98	[1.41; 2.78]	14.6%	12.4%
Kim 2016	0.11	0.1061	1.12	[0.91; 1.38]	39.5%	13.9%
Nakashima 2016	0.70	0.3554	2.01	[1.00; 4.04]	3.5%	8.1%
Wong 2016	1.14	0.6384	3.13	[0.90; 10.95]	1.1%	4.0%
Turri–Zanoni 2017	0.94	0.3407	2.56	[1.31; 4.99]	3.8%	8.4%
**Pooled HR by Fixed Effects**			**1.36**	**[1.19; 1.56]**	**90.9%**	**--**
**Pooled HR by Random Effects**			**1.69**	**[1.26; 2.25]**	**--**	**81.3%**
Heterogeneity: $I^2 = 66\%$ [30%; 83%], $\chi^2_8 = 23.24$ ($p < 0.01$)						
**Adjustment = Univariate**						
Fang 2013	0.54	0.2577	1.72	[1.04; 2.85]	6.7%	10.4%
Rassouli 2013	2.09	0.5334	8.07	[2.84; 22.97]	1.6%	5.2%
Haddad 2015	1.02	0.7413	2.77	[0.65; 11.83]	0.8%	3.2%
**Pooled HR by Fixed Effects**			**2.34**	**[1.52; 3.62]**	**9.1%**	**--**
**Pooled HR by Random Effects**			**3.16**	**[1.18; 8.45]**	**--**	**18.7%**
Heterogeneity: $I^2 = 71\%$ [1%; 91%], $\chi^2_2 = 6.87$ ($p = 0.03$)						
**Pooled HR by Fixed Effects**			**1.43**	**[1.26; 1.63]**	**100.0%**	**--**
**Pooled HR by Random Effects**			**1.90**	**[1.41; 2.54]**	**--**	**100.0%**
**Prediction interval**				[0.75; 4.79]		

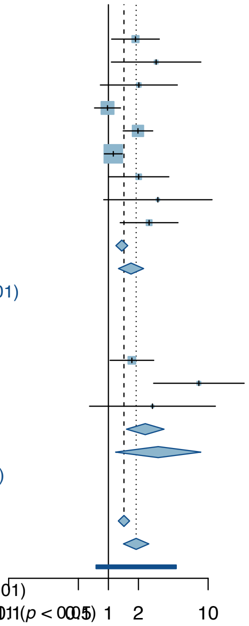

Heterogeneity: $I^2 = 69\%$ [44%; 83%], $\chi^2_{11} = 35.54$ ($p < 0.01$)
Residual heterogeneity: $I^2 = 67\%$ [37%; 82%], $\chi^2_{10} = 30.01$ ($p < 0.05$)

Tau²= 0.387

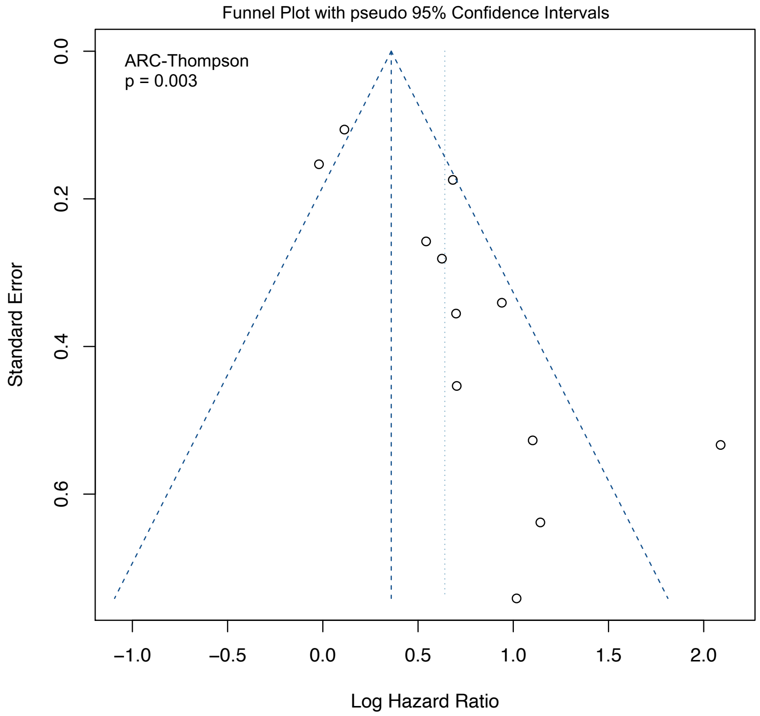

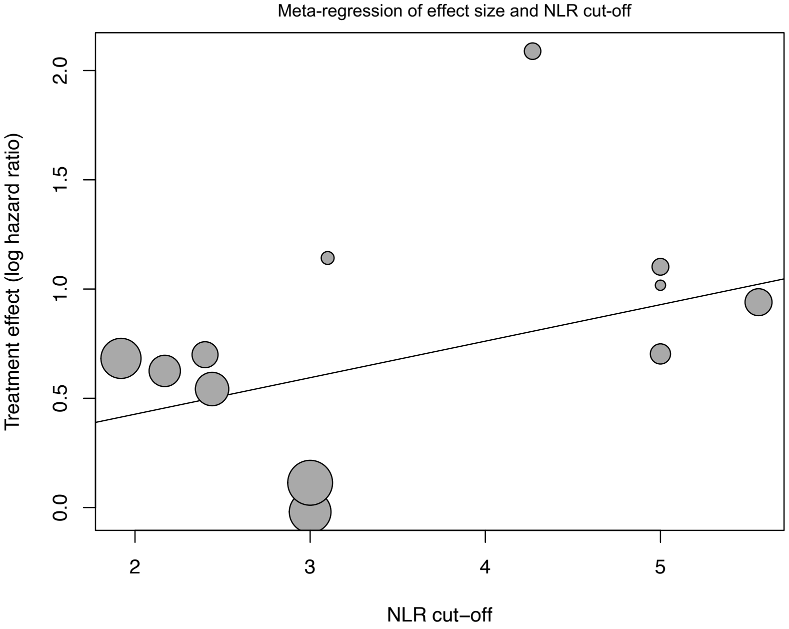

Supplementary Figure 165 - NLR: OS in Head and Neck (No Surgery)

Study	TE	seTE	HR	95%–CI	Weight (fixed)	Weight (random)
Chua 2016	0.06	0.1737	1.06	[0.75; 1.49]	25.6%	20.9%
He 2011	0.45	0.2144	1.57	[1.03; 2.39]	16.8%	16.0%
Sun 2016	0.63	0.3815	1.87	[0.89; 3.95]	5.3%	6.5%
Kano 2016	0.30	0.2460	1.35	[0.83; 2.18]	12.8%	13.2%
Kim 2016	0.42	0.2699	1.52	[0.90; 2.58]	10.6%	11.5%
Moon 2016	1.17	0.4027	3.22	[1.46; 7.09]	4.8%	5.9%
Rosculet 2017	1.12	0.8228	3.05	[0.61; 15.30]	1.1%	1.5%
Selzer 2015	0.62	0.2512	1.85	[1.13; 3.03]	12.2%	12.8%
Li 2016	0.14	0.2663	1.15	[0.68; 1.94]	10.9%	11.7%
**Pooled HR by Fixed Effects**			**1.44**	**[1.21; 1.71]**	**100.0%**	**--**
**Pooled HR by Random Effects**			**1.48**	**[1.21; 1.82]**	**--**	**100.0%**
**Prediction interval**				**[0.97; 2.26]**		

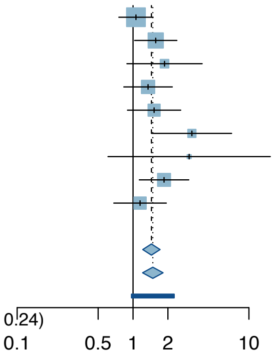

Heterogeneity: I^2 = 23% [0%; 64%], χ^2_8 = 10.40 (p = 0.24)
Tau^2 = 0.[illegible]

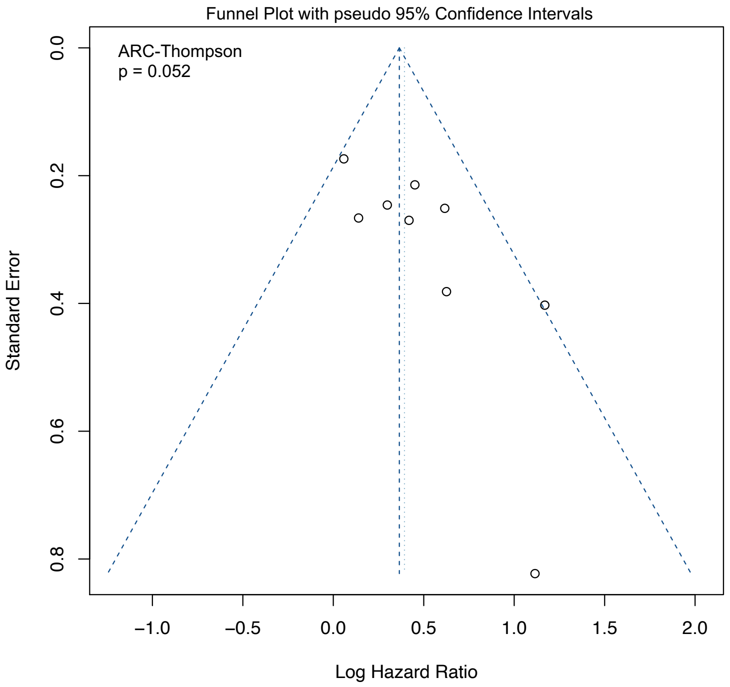

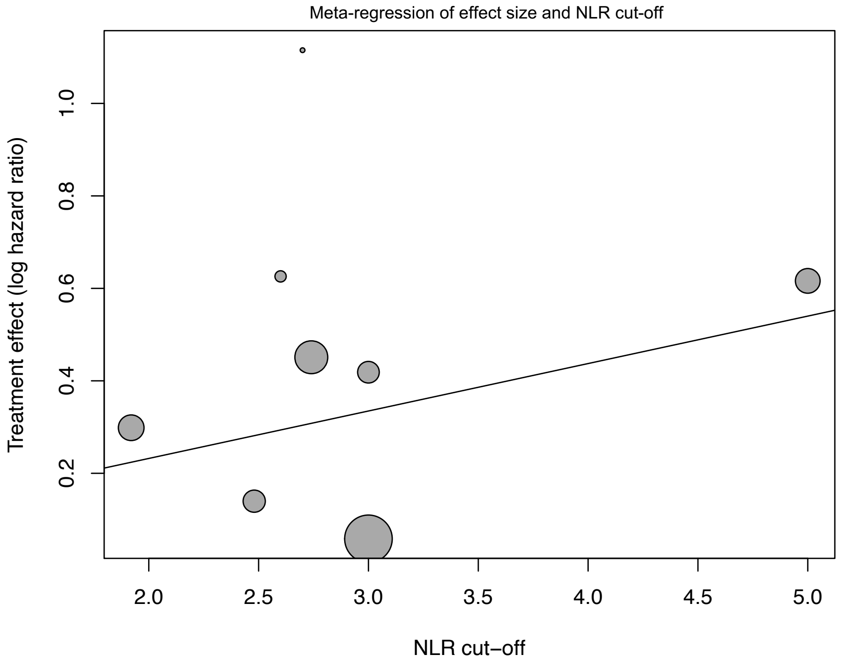

Supplementary Figure 166 - NLR: CSS in Head and Neck (SCC)

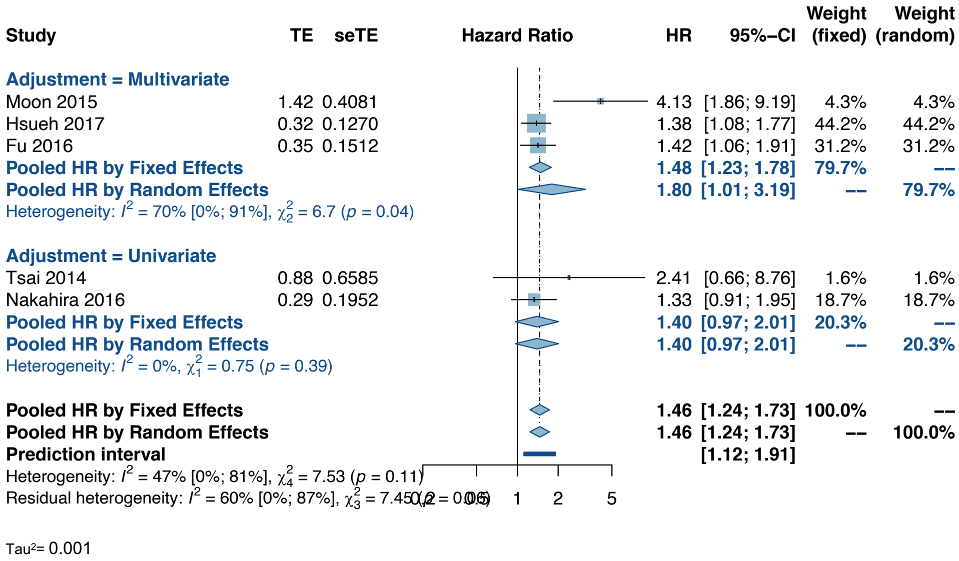

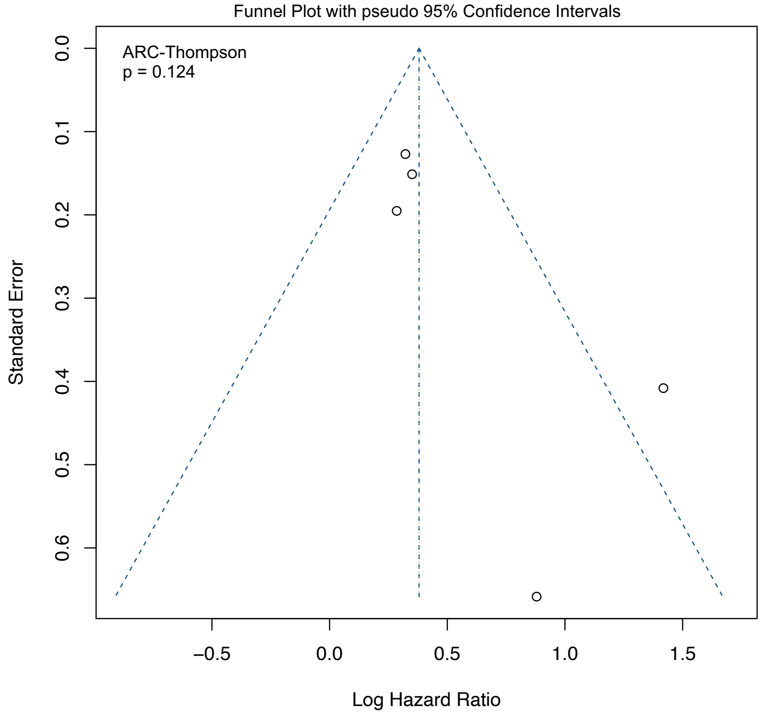

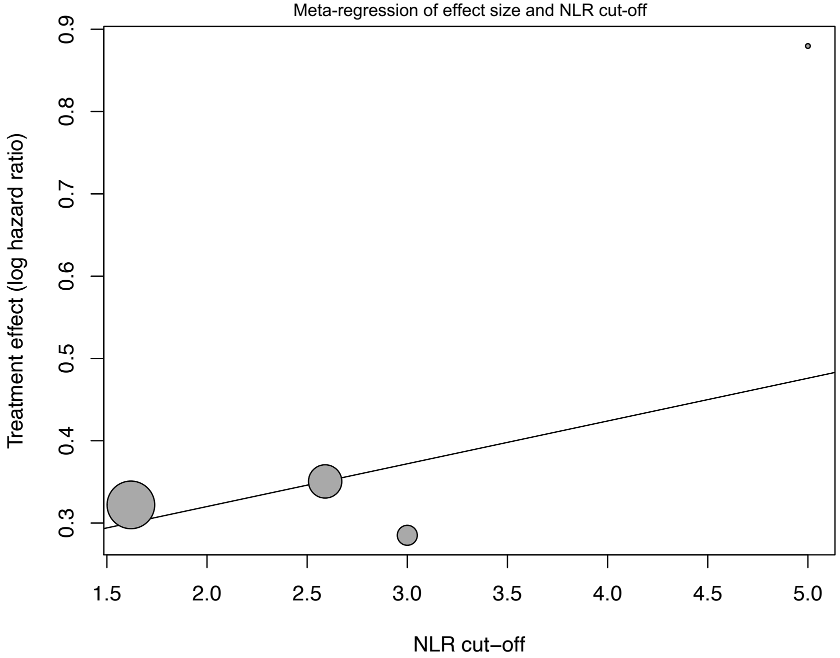

Supplementary Figure 167 - NLR: PFS in Head and Neck (SCC)

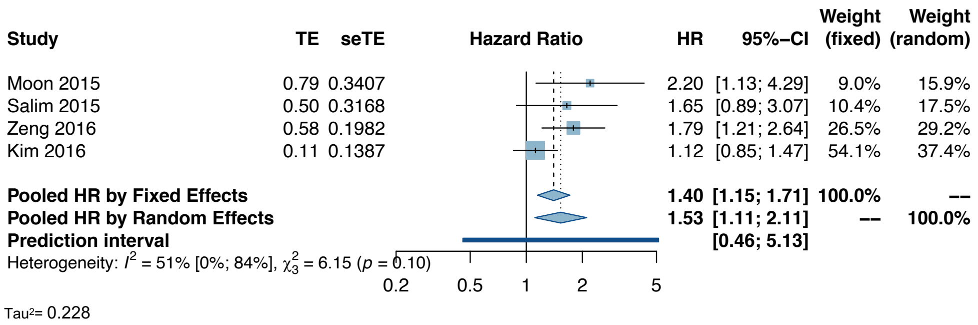

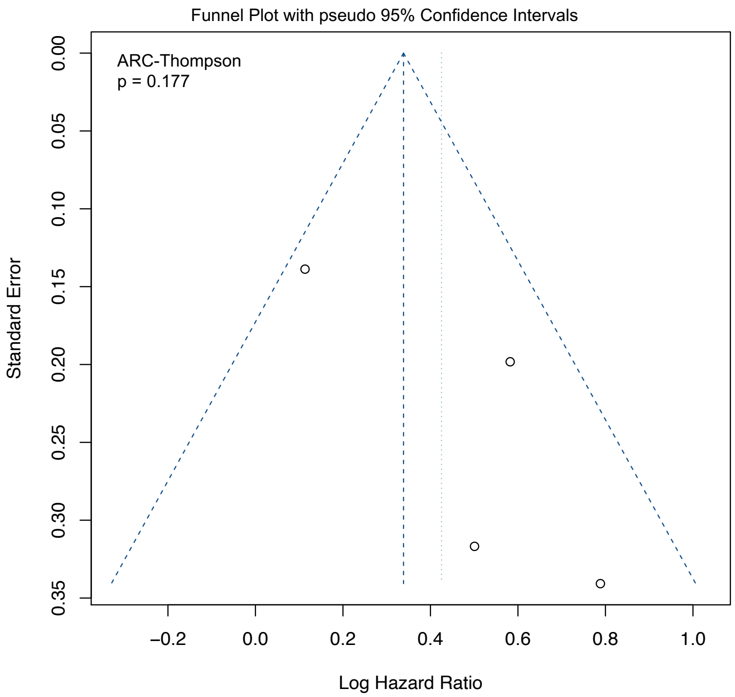

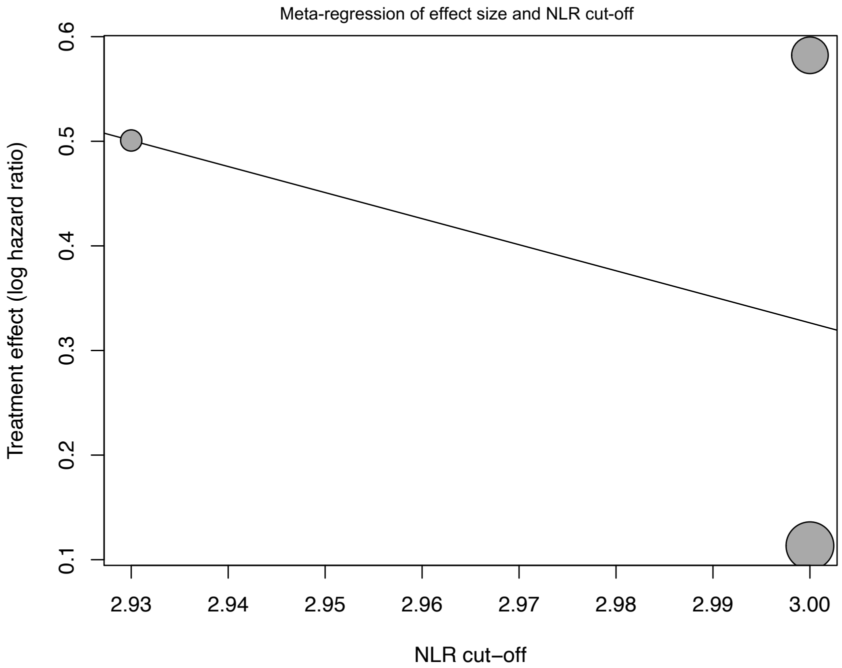

Supplementary Figure 168 - NLR: OS in Head and Neck (SR)

Study	TE	seTE	Hazard Ratio	HR	95%−CI	Weight (fixed)	Weight (random)
Fu 2016	0.27	0.1360		1.31	[1.00; 1.71]	70.3%	46.4%
Song 2015	0.86	0.2917		2.36	[1.33; 4.18]	15.3%	27.3%
Tu 2015	0.78	0.3006		2.18	[1.21; 3.92]	14.4%	26.4%
**Pooled HR by Fixed Effects**				**1.54**	**[1.23; 1.93]**	**100.0%**	**--**
**Pooled HR by Random Effects**				**1.76**	**[1.17; 2.65]**	**--**	**100.0%**
**Prediction interval**					**[0.02; 144.88]**		

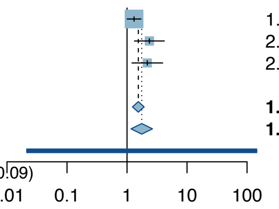

Heterogeneity: $I^2 = 59\%$ [0%; 88%], $\chi^2_2 = 4.88$ ($p = 0.09$)

Tau²= 0.277

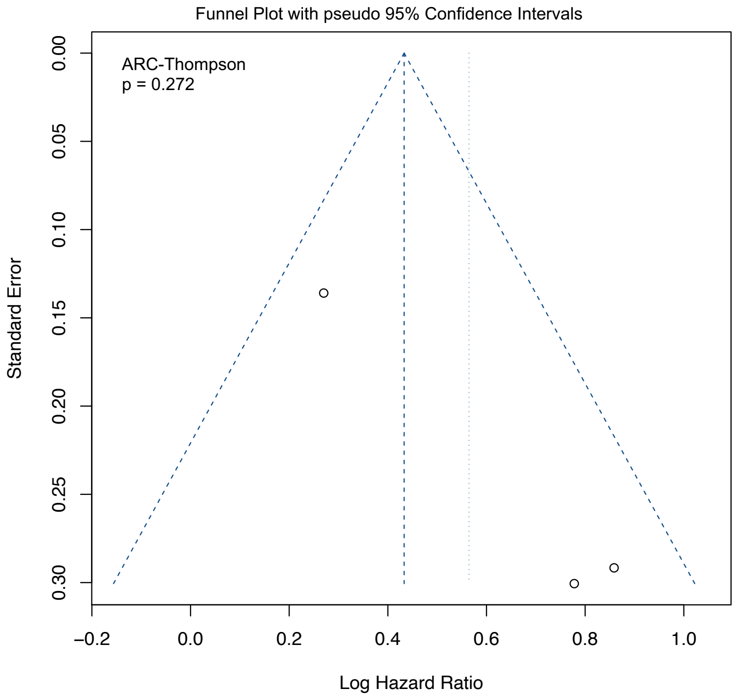

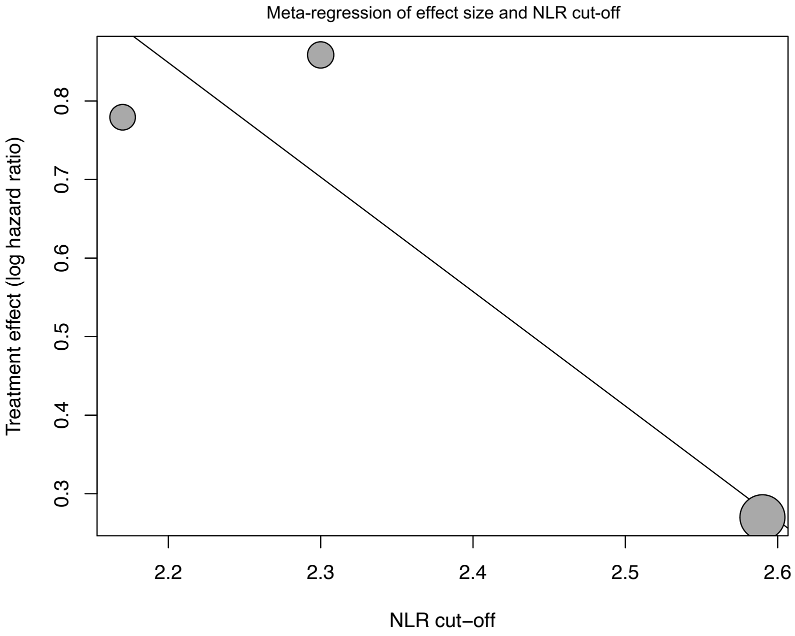

Supplementary Figure 169 - NLR: OS in Hypopharynx

Study	TE	seTE	HR	95%-CI	Weight (fixed)	Weight (random)
**Adjustment = Multivariate**						
Lo 2017	0.93	0.2706	2.53	[1.49; 4.30]	39.5%	39.5%
Ikeguchi 2016	1.72	0.7974	5.59	[1.17; 26.68]	4.6%	4.6%
**Pooled HR by Fixed Effects**			**2.75**	**[1.66; 4.54]**	**44.1%**	**--**
**Pooled HR by Random Effects**			**2.75**	**[1.66; 4.54]**	**--**	**44.1%**
Heterogeneity: $I^2 = 0\%$, $\chi^2_1 = 0.89$ ($p = 0.35$)						
**Adjustment = Univariate**						
Song 2015	1.10	0.2275	2.99	[1.91; 4.67]	55.9%	55.9%
**Pooled HR by Fixed Effects**			**2.99**	**[1.91; 4.67]**	**55.9%**	**--**
**Pooled HR by Random Effects**			**2.99**	**[1.91; 4.67]**	**--**	**55.9%**
Heterogeneity: not applicable						
**Pooled HR by Fixed Effects**			**2.88**	**[2.06; 4.02]**	**100.0%**	**--**
**Pooled HR by Random Effects**			**2.88**	**[2.06; 4.02]**	**--**	**100.0%**
**Prediction interval**				**[0.33; 25.01]**		

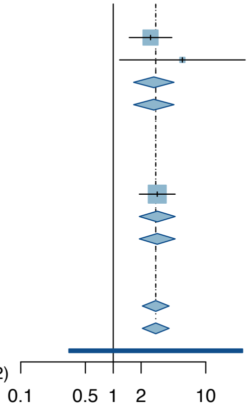

Heterogeneity: $I^2 = 0\%$ [0%; 78%], $\chi^2_2 = 0.95$ ($p = 0.62$)

Residual heterogeneity: $I^2 = 0\%$, $\chi^2_1 = 0.89$ ($p = 0.35$)

Tau²= 0

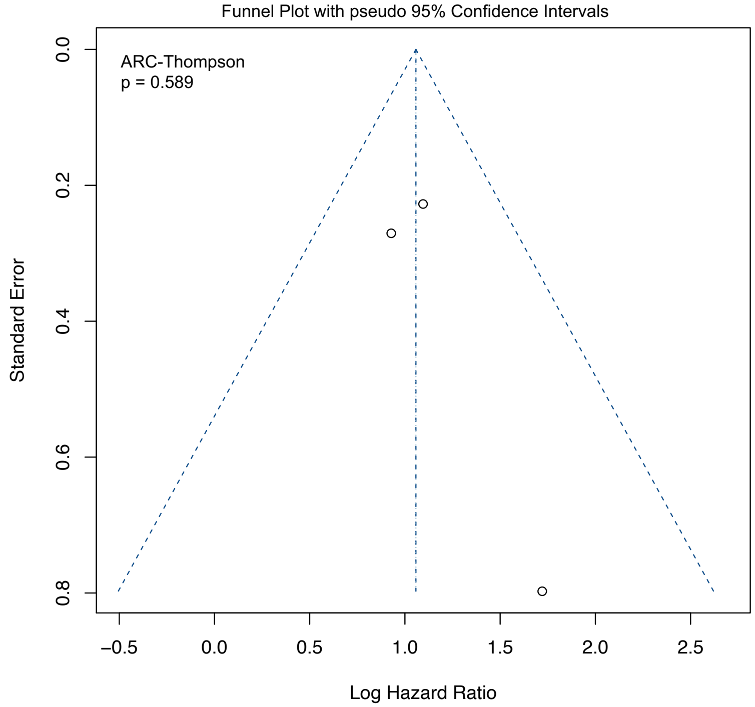

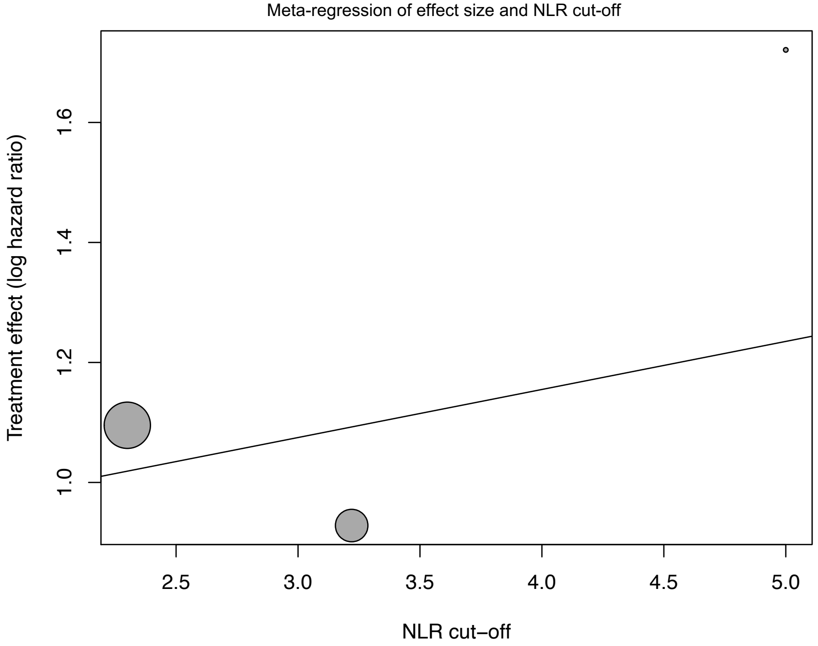

Supplementary Figure 170 - NLR: DFS in Larynx

Study	TE	seTE	HR	95%–CI	Weight (fixed)	Weight (random)
Wong 2016	0.39	0.2205	1.48	[0.96; 2.28]	17.3%	17.7%
Tu 2015	0.63	0.2804	1.87	[1.08; 3.24]	10.7%	11.0%
Hsueh 2017	0.24	0.1082	1.27	[1.03; 1.57]	72.0%	71.3%
**Pooled HR by Fixed Effects**			**1.36**	**[1.14; 1.63]**	**100.0%**	**--**
**Pooled HR by Random Effects**			**1.36**	**[1.13; 1.63]**	**--**	**100.0%**
**Prediction interval**				**[0.40; 4.61]**		

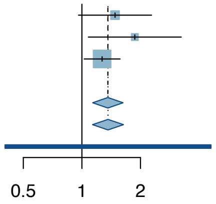

Heterogeneity: $I^2 = 0\%$ [0%; 89%], $\chi^2_2 = 1.84$ ($p = 0.40$)

Tau²= 0.022

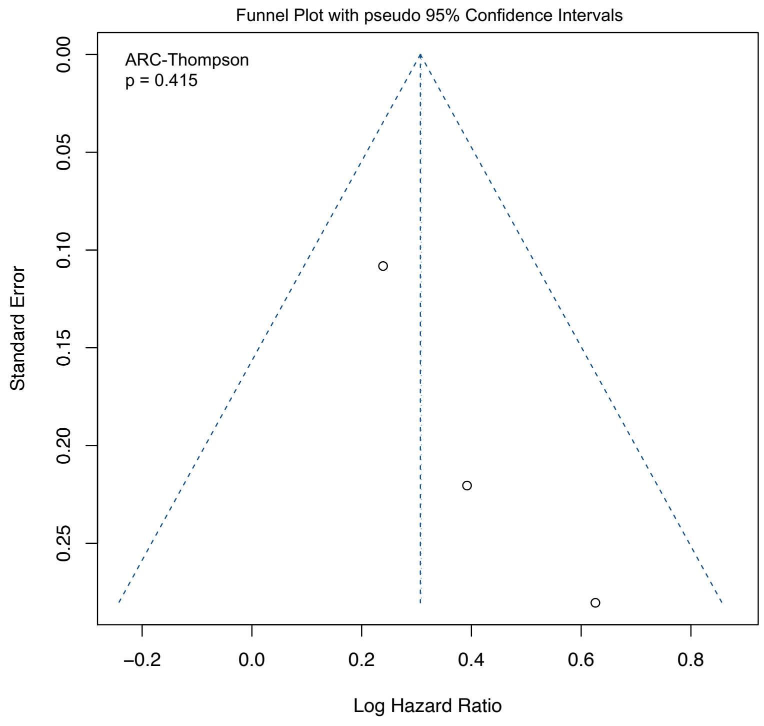

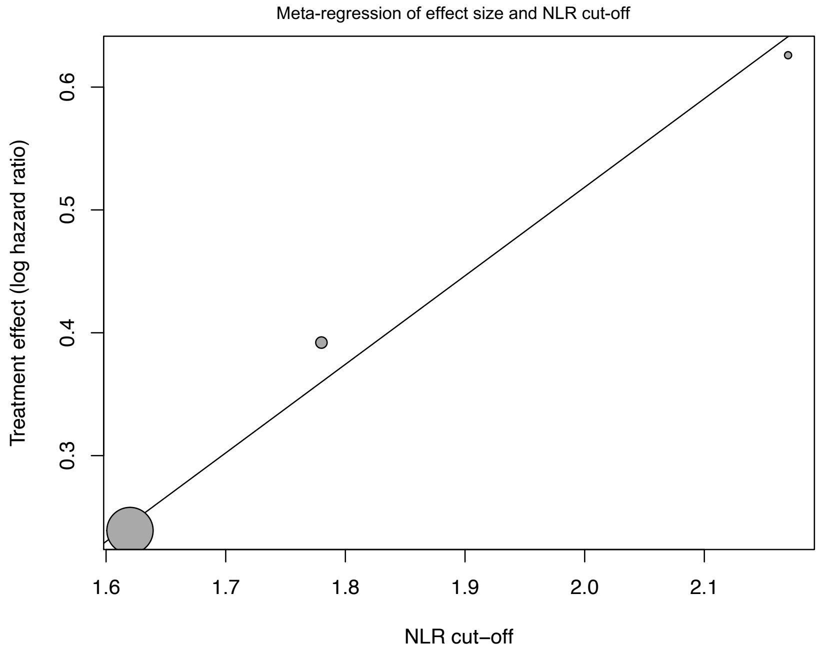

Supplementary Figure 171 - NLR: OS in Nasopharyngeal

Study	TE	seTE	HR	95%−CI	Weight (fixed)	Weight (random)
Ying Jin 2016	0.51	0.1367	1.66	[1.27; 2.17]	17.9%	18.0%
Xin An 2011	0.55	0.2108	1.74	[1.15; 2.63]	7.5%	9.0%
Xiao−Hui Li 2016	0.66	0.3363	1.94	[1.00; 3.75]	3.0%	3.9%
Wei Sun 2016	0.63	0.3802	1.87	[0.89; 3.94]	2.3%	3.1%
Melvin Lee Kiang Chua 2016	0.06	0.1703	1.06	[0.76; 1.48]	11.5%	12.8%
Jian−Rong He 2012	0.45	0.2123	1.57	[1.04; 2.38]	7.4%	8.9%
Jian−Pei Li 2016	0.14	0.2668	1.15	[0.68; 1.94]	4.7%	5.9%
H Chang 2013	0.30	0.0930	1.35	[1.13; 1.62]	38.6%	29.3%
Cui Chen 2014	0.59	0.2503	1.80	[1.10; 2.94]	5.3%	6.7%
Aiying Lu 2017	0.87	0.4236	2.38	[1.04; 5.46]	1.9%	2.5%
**Pooled HR by Fixed Effects**			**1.46**	**[1.30; 1.63]**	**100.0%**	**--**
**Pooled HR by Random Effects**			**1.48**	**[1.29; 1.69]**	**--**	**100.0%**
**Prediction interval**				**[1.15; 1.90]**		

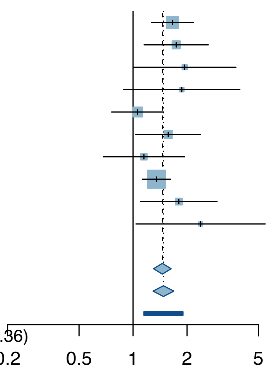

Heterogeneity: $I^2 = 9\%$ [0%; 66%], $\chi^2_9 = 9.90$ ($p = 0.36$)

Tau²= 0.085

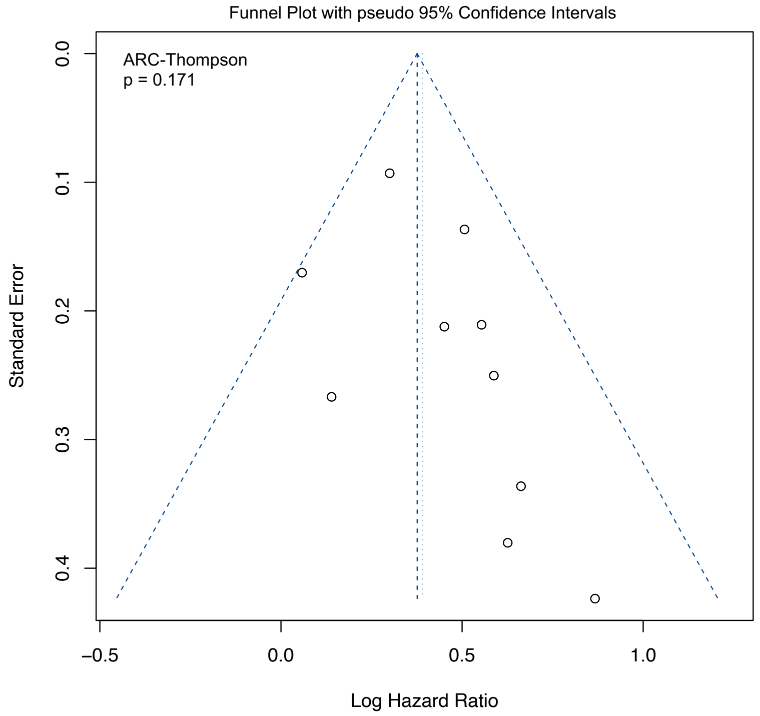

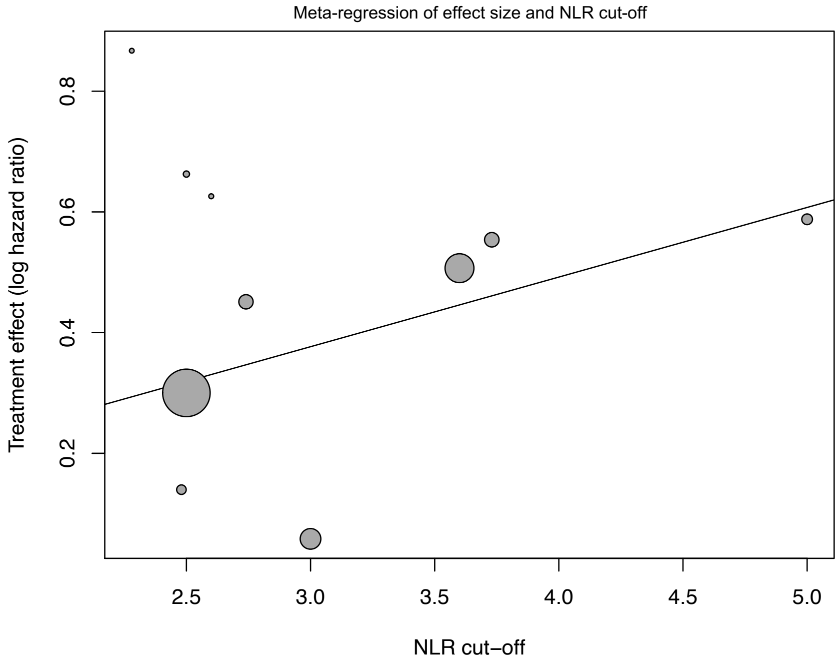

Supplementary Figure 172 - NLR: OS in Oral cavity

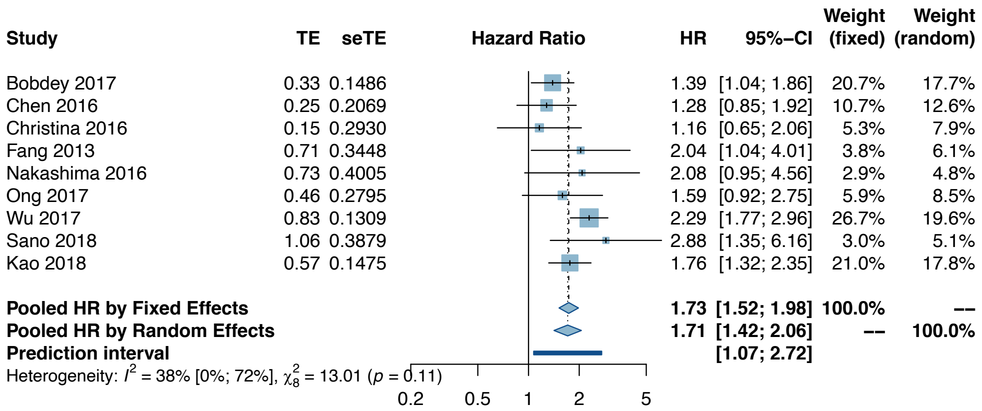

Tau²= 0.172

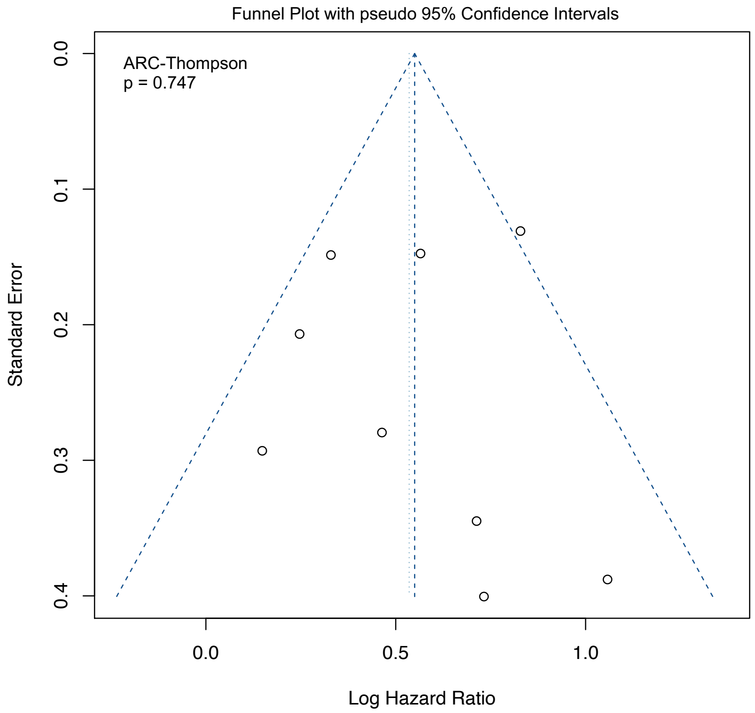

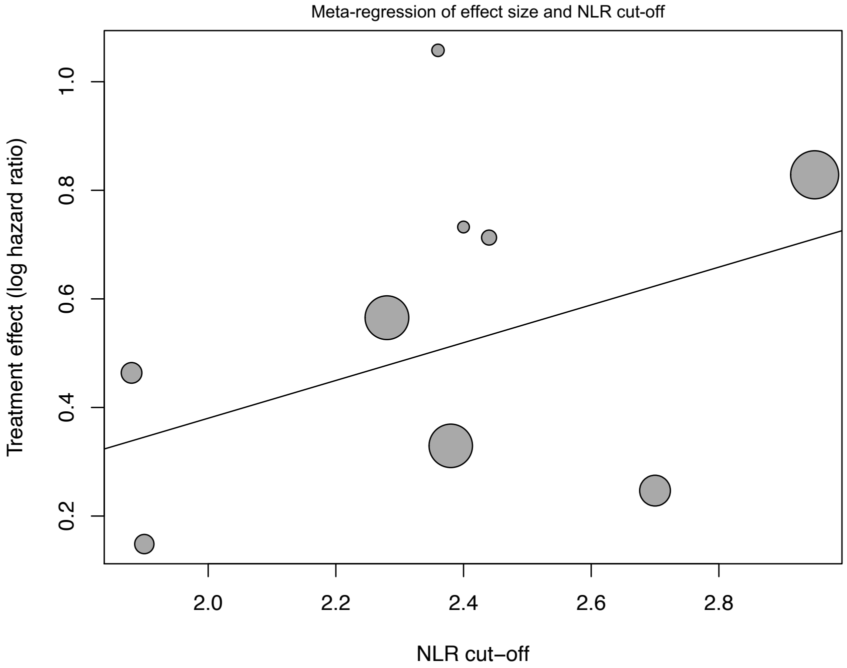

Supplementary Figure 173 - NLR: DFS in Oral cavity

Study	TE	seTE	HR	95%–CI	Weight (fixed)	Weight (random)
Chen 2016	0.23	0.2042	1.26	[0.84; 1.88]	20.4%	20.4%
Fang 2013	0.54	0.2577	1.72	[1.04; 2.85]	12.8%	12.8%
Lee 2017	0.55	0.1767	1.74	[1.23; 2.46]	27.3%	27.3%
Nakashima 2016	0.70	0.3687	2.01	[0.98; 4.14]	6.3%	6.3%
Ong 2017	0.43	0.2444	1.53	[0.95; 2.47]	14.3%	14.3%
Perisanidis 2013	2.34	1.0678	10.37	[1.28; 84.08]	0.7%	0.7%
Tsai 2014	0.88	0.6585	2.41	[0.66; 8.76]	2.0%	2.0%
Wu 2017	0.65	0.3220	1.91	[1.02; 3.59]	8.2%	8.2%
Park 2018	0.97	0.4905	2.65	[1.01; 6.93]	3.5%	3.5%
Sano 2018	1.13	0.4398	3.10	[1.31; 7.34]	4.4%	4.4%
**Pooled HR by Fixed Effects**			**1.72**	**[1.44; 2.07]**	**100.0%**	**--**
**Pooled HR by Random Effects**			**1.72**	**[1.44; 2.07]**	**--**	**100.0%**
**Prediction interval**				**[1.39; 2.13]**		

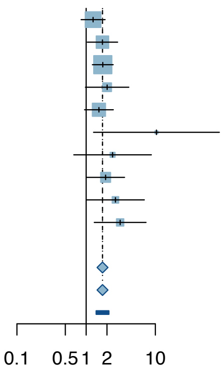

Heterogeneity: $I^2 = 0\%$ [0%; 60%], $\chi^2_9 = 8.51$ ($p = 0.48$)

Tau²= 0.002

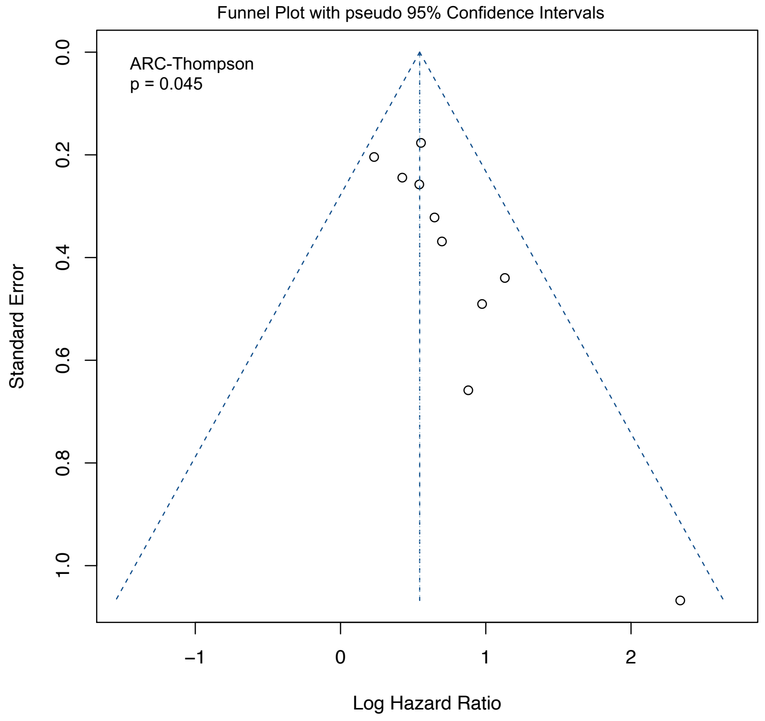

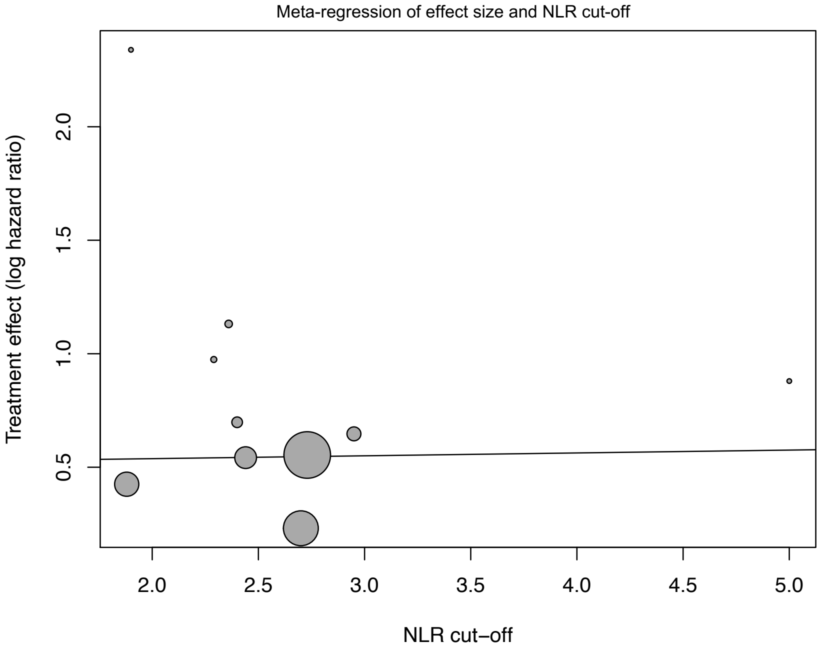

Supplementary Figure 174 - NLR: DFS in Oropharynx

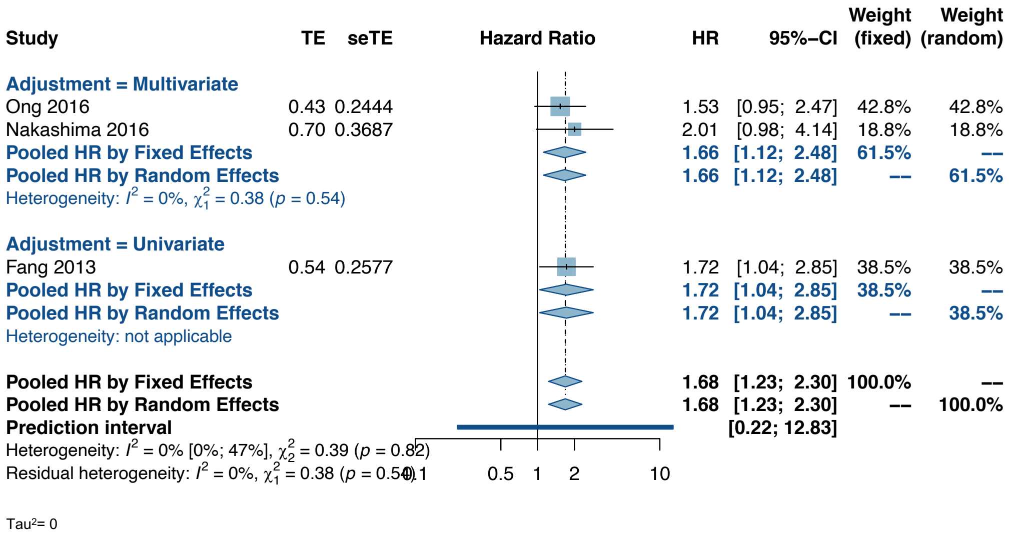

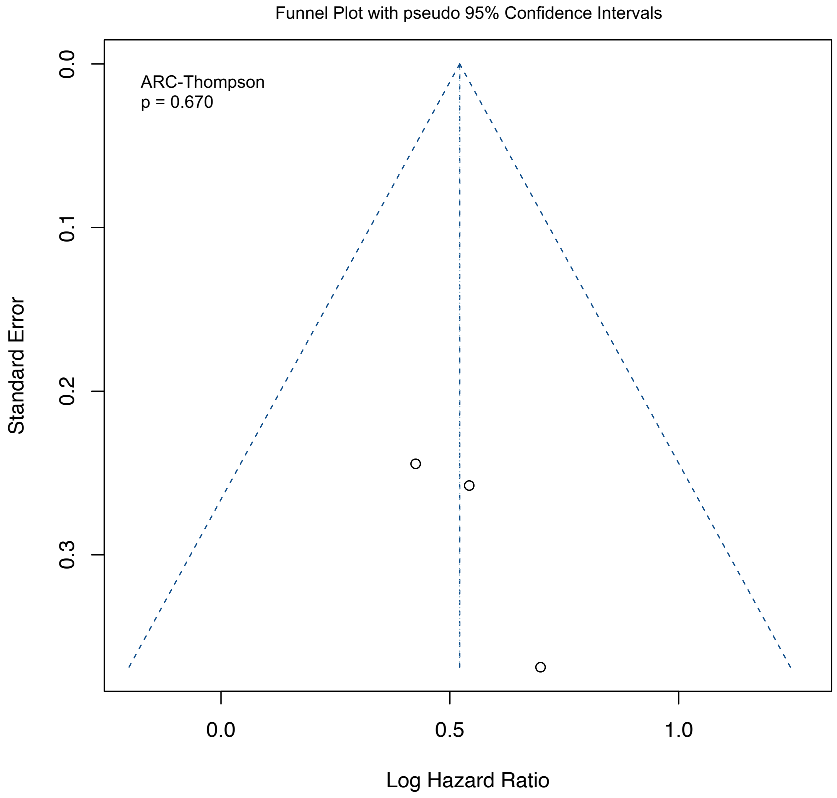

Supplementary Figure 175 - NLR: OS in Melanoma (Immune checkpoint inhibitors)

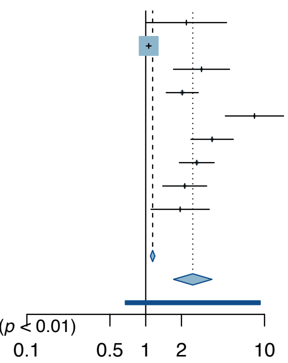

Study	TE	seTE	HR	95%-CI	Weight (fixed)	Weight (random)
J Zaragoza 2015	0.79	0.3970	2.20	[1.01; 4.79]	0.3%	8.5%
Leila Khoja 2016	0.06	0.0235	1.06	[1.01; 1.11]	91.8%	13.4%
Mariaelena Capone 2018	1.08	0.2773	2.95	[1.71; 5.08]	0.7%	10.4%
Micheal R Cassidy 2017	0.71	0.1586	2.03	[1.49; 2.77]	2.0%	12.3%
Minkyu Jung 2016	2.11	0.2879	8.23	[4.68; 14.47]	0.6%	10.2%
PF Ferrucci (1) 2015	1.29	0.2097	3.62	[2.40; 5.46]	1.2%	11.5%
PF Ferrucci (2) 2015	0.99	0.1722	2.69	[1.92; 3.77]	1.7%	12.1%
PF Ferrucci (3) 2015	0.76	0.2171	2.13	[1.39; 3.26]	1.1%	11.4%
Rosner 2018	0.67	0.2881	1.95	[1.11; 3.43]	0.6%	10.2%
**Pooled HR by Fixed Effects**			**1.14**	**[1.09; 1.20]**	**100.0%**	**--**
**Pooled HR by Random Effects**			**2.49**	**[1.72; 3.61]**	**--**	**100.0%**
**Prediction interval**				**[0.67; 9.19]**		

Heterogeneity: $I^2 = 95\%$ [92%; 97%], $\chi^2_8 = 151.41$ ($p < 0.01$)

Tau²= 0.519

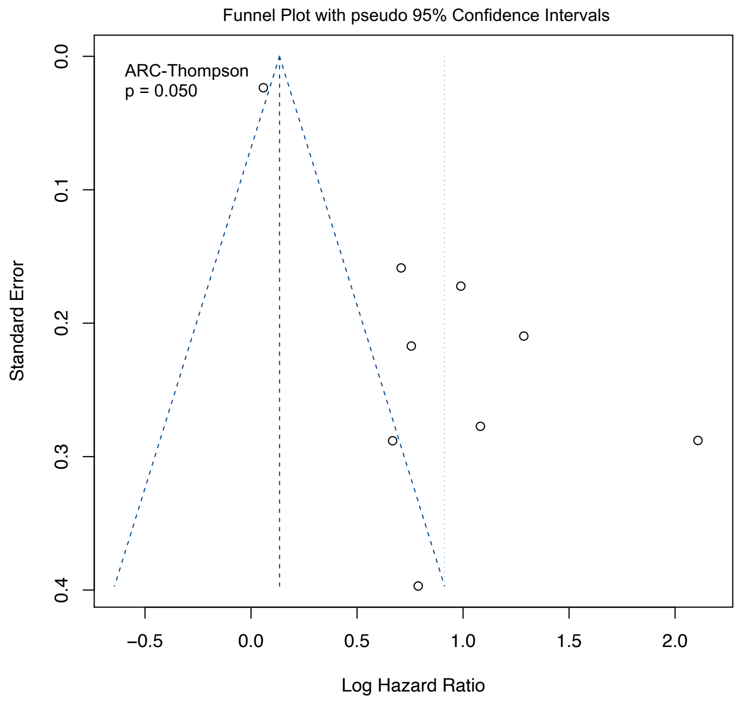

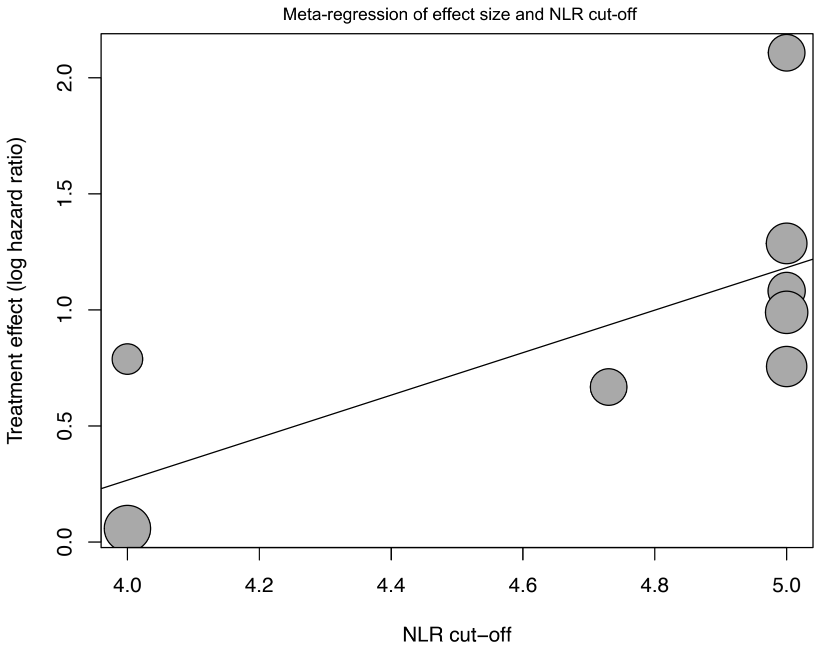

Supplementary Figure 176 - NLR: PFS in Melanoma (Immune checkpoint inhibitors)

Study	TE	seTE	HR	95%-CI	Weight (fixed)	Weight (random)
Mariaelena Capone 2018	0.74	0.2736	2.10	[1.23; 3.59]	7.9%	12.1%
Micheal R Cassidy 2017	0.59	0.1565	1.81	[1.33; 2.46]	24.2%	26.6%
Minkyu Jung 2016	0.66	0.1112	1.93	[1.55; 2.40]	47.9%	37.6%
PF Ferrucci (1) 2015	1.05	0.1724	2.86	[2.04; 4.01]	19.9%	23.7%
**Pooled HR by Fixed Effects**			**2.07**	**[1.78; 2.41]**	**100.0%**	**--**
**Pooled HR by Random Effects**			**2.10**	**[1.71; 2.59]**	**--**	**100.0%**
**Prediction interval**				**[1.02; 4.33]**		

Heterogeneity: $I^2 = 35\%$ [0%; 78%], $\chi^2_3 = 4.65$ ($p = 0.20$)

Tau²= 0.131

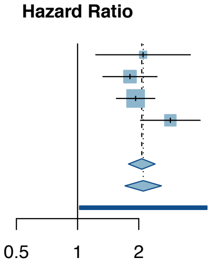

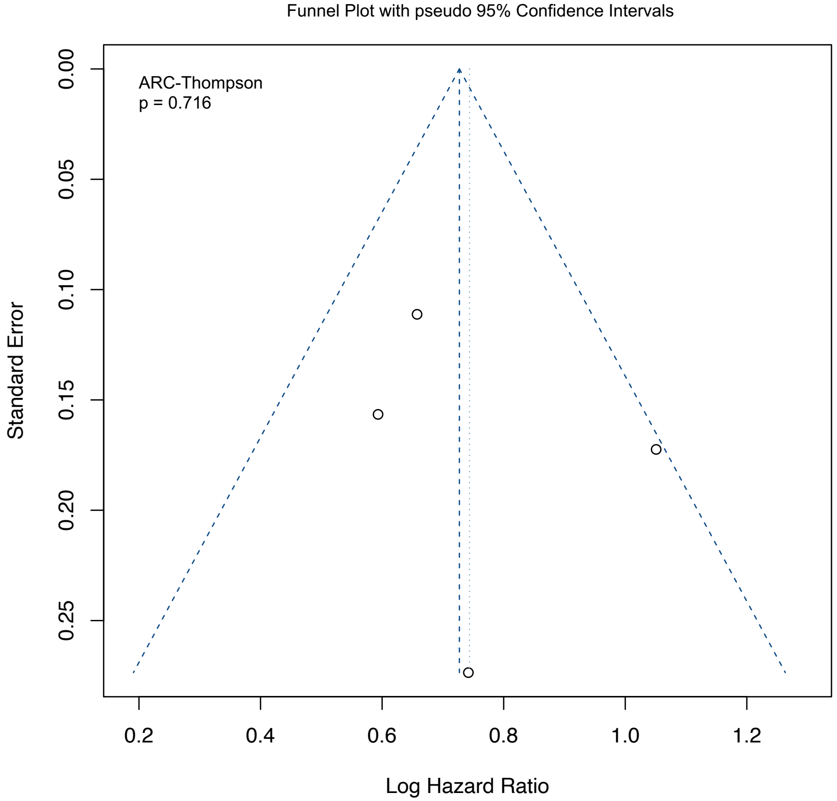

Supplementary Figure 177 - NLR: OS in Urothelial

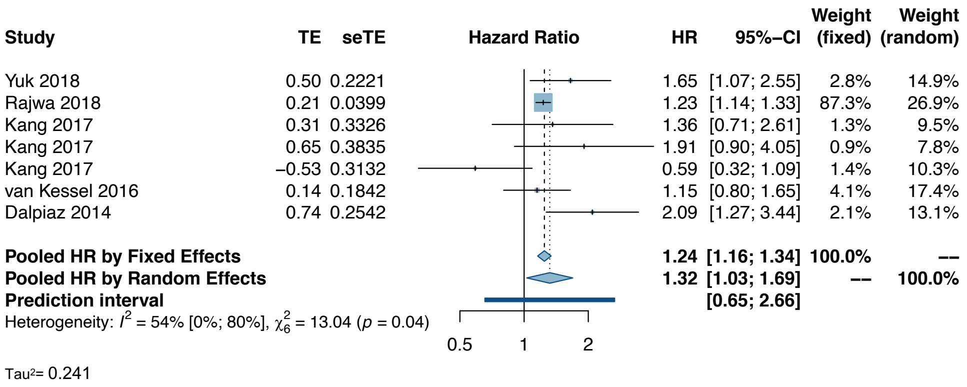

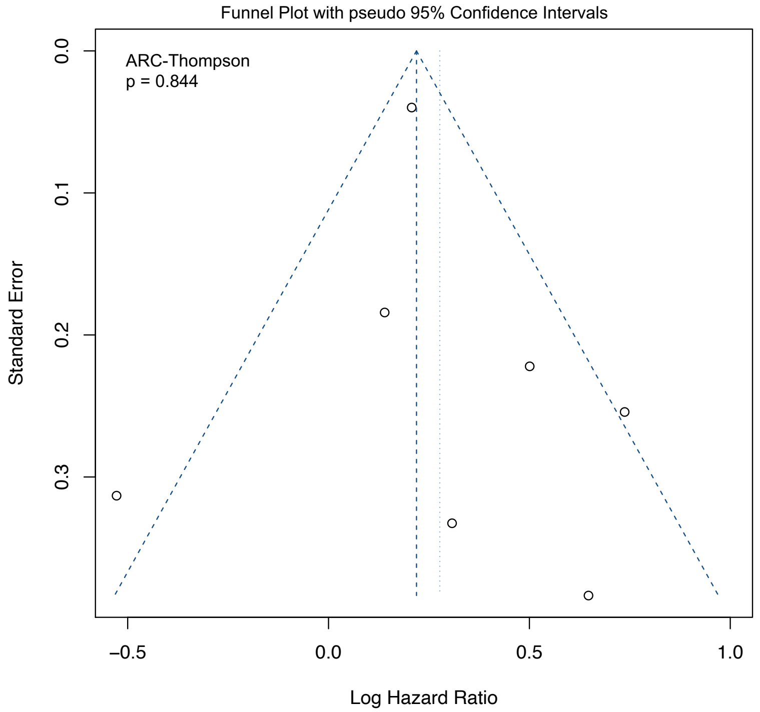

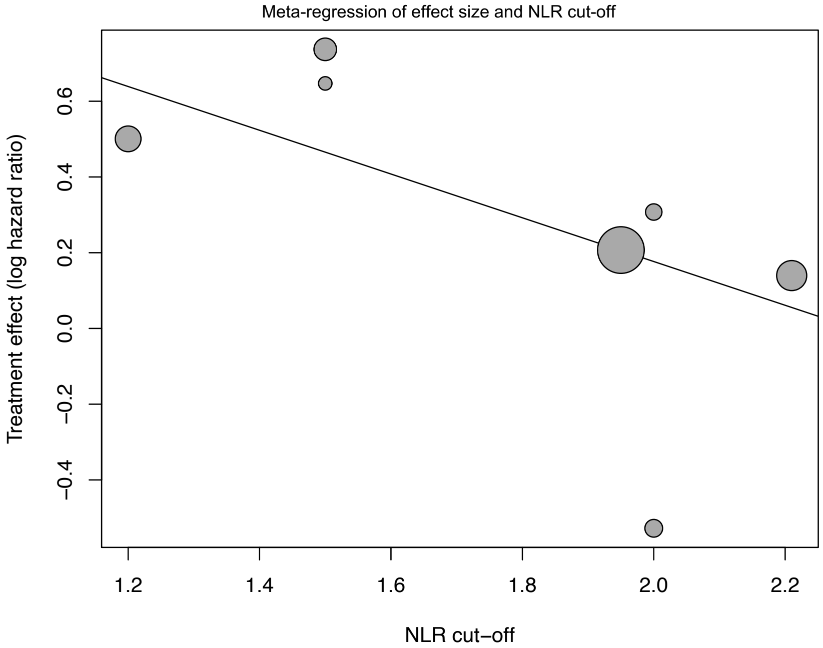

Supplementary Figure 178 - NLR: CSS in UTUC (Nephroureterectomy)

Study	TE	seTE	HR	95%–CI	Weight (fixed)	Weight (random)
Azuma 2013	1.12	0.4096	3.06	[1.37; 6.83]	2.5%	5.0%
Cheng 2016	0.31	0.3775	1.36	[0.65; 2.85]	3.0%	5.6%
Dalpiaz 2013	1.00	0.3976	2.72	[1.25; 5.93]	2.7%	5.2%
Huang J 2017	0.55	0.1959	1.73	[1.18; 2.54]	11.0%	12.4%
Jan H 2019	0.08	0.3945	1.08	[0.50; 2.34]	2.7%	5.3%
Kang 2017	0.39	0.4498	1.47	[0.61; 3.55]	2.1%	4.3%
Kohada 2018	1.18	0.4394	3.25	[1.37; 7.69]	2.2%	4.5%
Kuroda 2019	0.47	0.5008	1.60	[0.60; 4.27]	1.7%	3.6%
Son 2018	0.53	0.2207	1.70	[1.10; 2.62]	8.7%	11.1%
Tanaka 2015	0.54	0.2292	1.71	[1.09; 2.68]	8.0%	10.6%
Vartolomei 2017	0.07	0.1032	1.07	[0.87; 1.31]	39.6%	18.2%
Xu 2019	0.60	0.1632	1.83	[1.33; 2.52]	15.8%	14.3%
**Pooled HR by Fixed Effects**			**1.46**	**[1.29; 1.66]**	**100.0%**	**--**
**Pooled HR by Random Effects**			**1.64**	**[1.34; 2.02]**	**--**	**100.0%**
**Prediction interval**				**[0.96; 2.83]**		

Hazard Ratio (axis): 0.2 0.5 1 2 5

Heterogeneity: I^2 = 51% [5%; 75%], χ^2_{11} = 22.36 (p = 0.02)

Tau² = 0.220

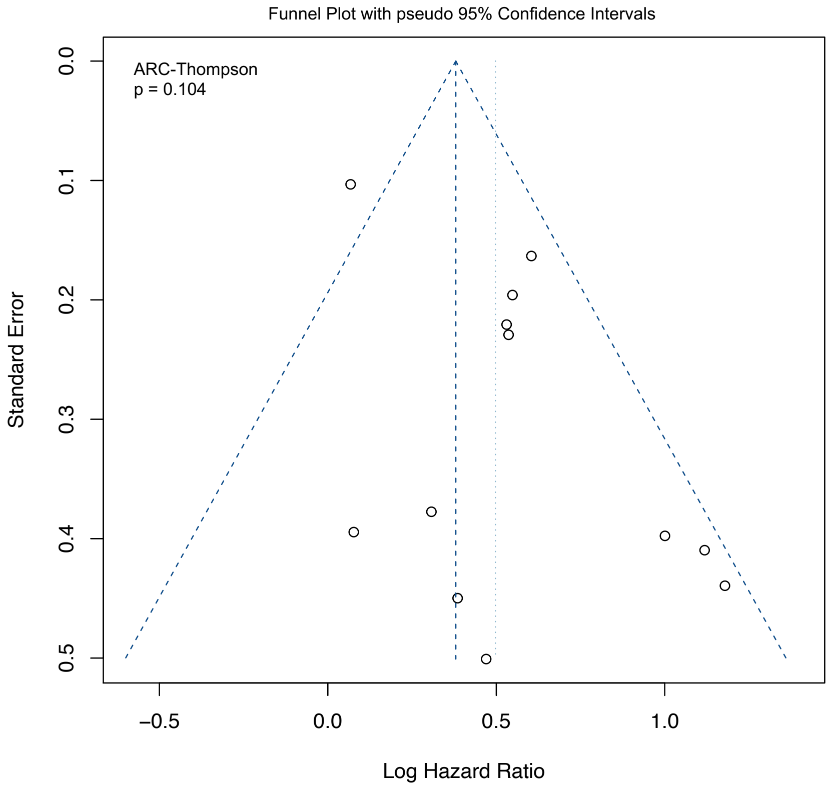

Supplementary Figure 179 - NLR: CSS in UTUC (Radical Cystectomy)

Study	TE	seTE	HR	95%–CI	Weight (fixed)	Weight (random)
Buisan 2016	0.24	0.0641	1.27	[1.12; 1.44]	2.8%	12.7%
D'Andrea 2017	0.18	0.0786	1.20	[1.03; 1.40]	1.9%	11.9%
Gondo 2012	0.67	0.3212	1.95	[1.04; 3.66]	0.1%	3.3%
Hermanns 2014	0.63	0.1535	1.88	[1.39; 2.54]	0.5%	8.1%
Kang 2017	0.15	0.0462	1.16	[1.06; 1.27]	5.4%	13.4%
Lucca 2016	0.19	0.0634	1.21	[1.07; 1.37]	2.9%	12.7%
Miyake 2017	0.41	0.4606	1.50	[0.61; 3.70]	0.1%	1.8%
Ozcan 2015	0.68	0.3062	1.97	[1.08; 3.59]	0.1%	3.5%
Rajwa 2018	0.05	0.0144	1.05	[1.02; 1.08]	55.4%	14.3%
Tan 2017	0.99	0.4120	2.68	[1.20; 6.01]	0.1%	2.2%
Un 2018	0.96	0.4528	2.61	[1.07; 6.34]	0.1%	1.9%
Viers 2014	0.04	0.0193	1.04	[1.00; 1.08]	30.9%	14.2%
**Pooled HR by Fixed Effects**			**1.07**	**[1.05; 1.09]**	**100.0%**	**--**
**Pooled HR by Random Effects**			**1.29**	**[1.13; 1.47]**	**--**	**100.0%**
**Prediction interval**				**[0.85; 1.95]**		

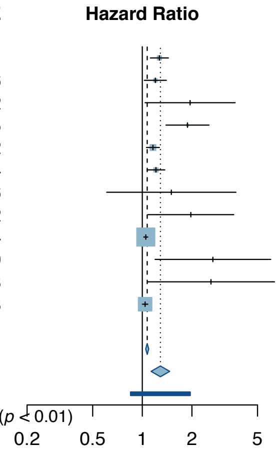

Heterogeneity: $I^2 = 78\%$ [62%; 87%], $\chi^2_{11} = 50.31$ ($p < 0.01$)

Tau²= 0.175

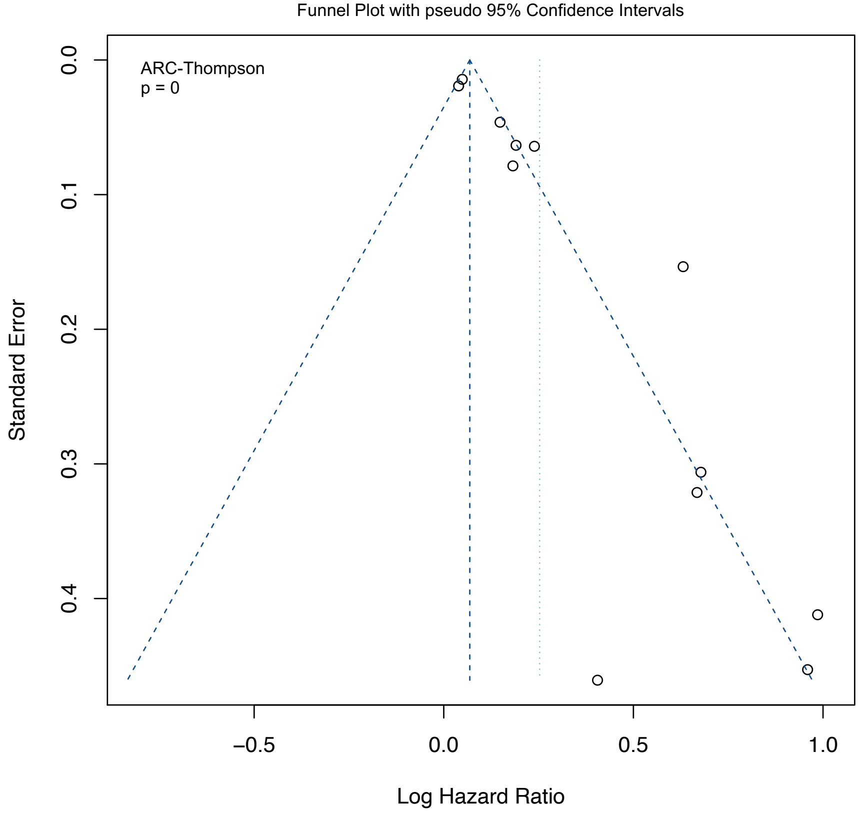

Supplementary Figure 180 - NLR: DFS in Urothelial

Study	TE	seTE	HR	95%-CI	Weight (fixed)	Weight (random)
van Kessel 2016	0.15	0.1890	1.16	[0.80; 1.68]	65.2%	56.3%
Kim 2015	0.65	0.2585	1.91	[1.15; 3.17]	34.8%	43.7%
**Pooled HR by Fixed Effects**			**1.38**	**[1.02; 1.86]**	**100.0%**	**--**
**Pooled HR by Random Effects**			**1.44**	**[0.89; 2.34]**	**--**	**100.0%**

Heterogeneity: $I^2 = 59\%$ [0%; 90%], $\chi^2_1 = 2.43$ ($p = 0.12$)

Hazard Ratio axis: 0.5, 1, 2

Tau²= 0.270

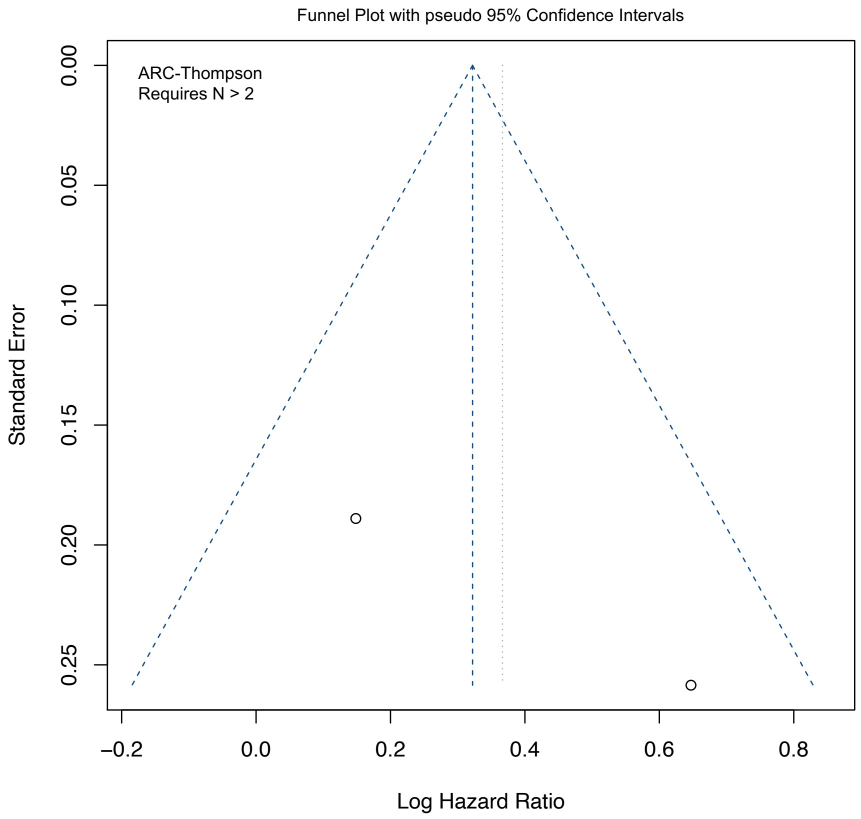

Supplementary Figure 181 - NLR: CSS in Urothelial (Radical Cystectomy)

Study	TE	seTE	Hazard Ratio	HR	95%–CI	Weight (fixed)	Weight (random)
Gondo 2012	0.67	0.3212		1.95	[1.04; 3.66]	0.1%	7.9%
Krane 2013	1.16	0.5464		3.18	[1.09; 9.28]	0.1%	4.1%
Viers 2014	0.04	0.0145		1.04	[1.01; 1.07]	71.8%	15.2%
Ku 2015	0.70	0.3184		2.02	[1.08; 3.77]	0.1%	7.9%
Ozcan 2015	0.68	0.2932		1.97	[1.11; 3.50]	0.2%	8.6%
Nguyen 2016	0.03	0.0289		1.03	[0.97; 1.09]	18.1%	15.1%
Bhindi 2016	0.39	0.1033		1.47	[1.20; 1.80]	1.4%	13.9%
D'Andrea 2017	0.18	0.0447		1.20	[1.10; 1.31]	7.5%	14.9%
Morizawa 2016	0.95	0.1556		2.58	[1.90; 3.50]	0.6%	12.5%
**Pooled HR by Fixed Effects**				**1.06**	**[1.04; 1.09]**	**100.0%**	**--**
**Pooled HR by Random Effects**				**1.53**	**[1.18; 1.97]**	**--**	**100.0%**
**Prediction interval**					**[0.66; 3.56]**		

Heterogeneity: $I^2 = 88\%$ [80%; 93%], $\chi^2_8 = 69.19$ ($p < 0.01$)

Tau²= 0.333

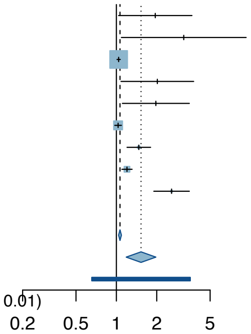

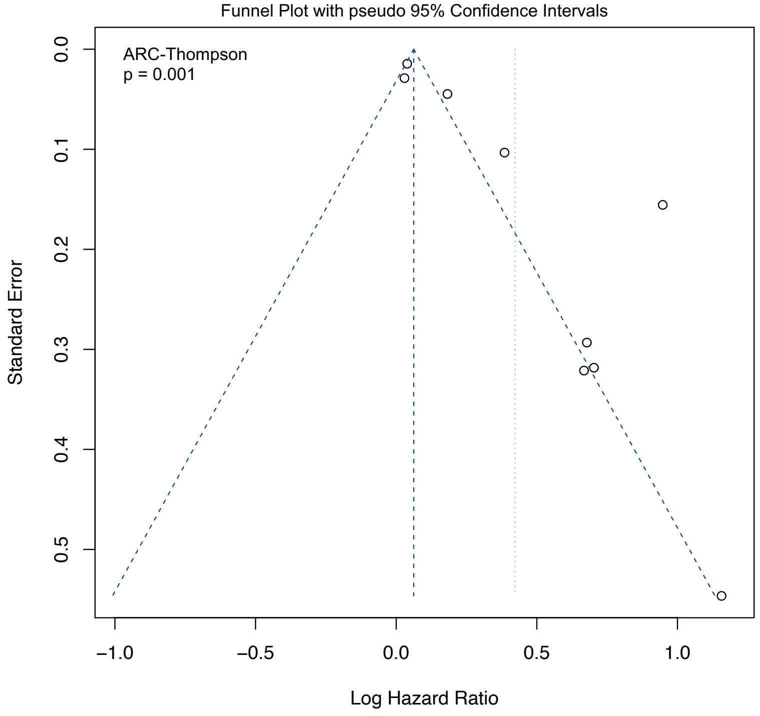

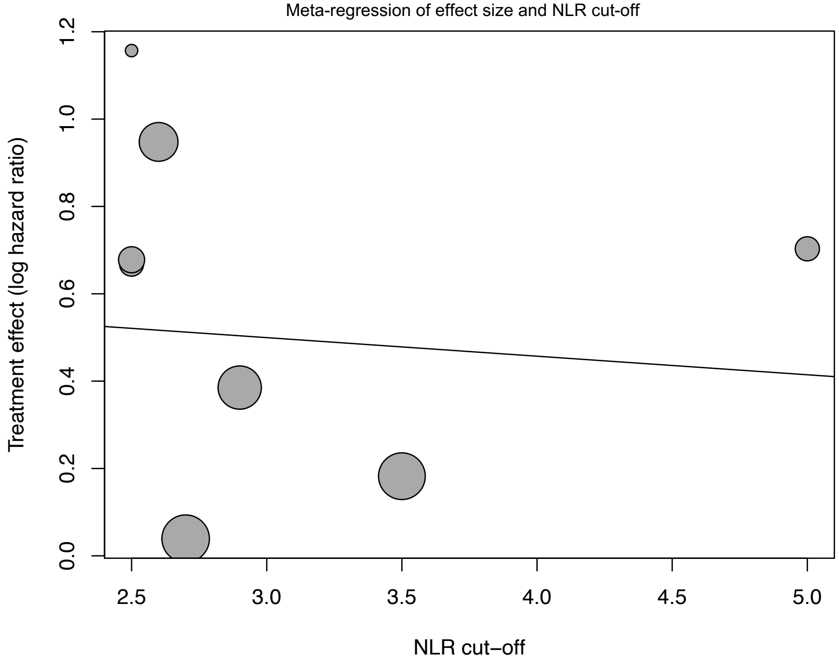

Supplementary Figure 182 - NLR: OS in Urothelial (Radical Cystectomy)

Study	TE	seTE	Hazard Ratio	HR	95%–CI	Weight (fixed)	Weight (random)
Krane 2013	0.91	0.3987		2.49	[1.14; 5.44]	0.0%	6.3%
Viers 2014	0.03	0.0098		1.03	[1.01; 1.05]	77.8%	13.2%
Ku 2015	0.83	0.2664		2.29	[1.36; 3.86]	0.1%	8.9%
Yoshida 2016	0.85	0.1846		2.34	[1.63; 3.36]	0.2%	10.7%
Bhindi 2016	0.44	0.1541		1.56	[1.15; 2.11]	0.3%	11.4%
Morizawa 2016	1.02	0.3536		2.77	[1.39; 5.54]	0.1%	7.1%
Ojerholm 2017	0.04	0.0332		1.04	[0.97; 1.11]	6.8%	13.1%
Nguyen 2016	0.02	0.0292		1.02	[0.96; 1.08]	8.8%	13.2%
D'Andrea 2017	0.10	0.0358		1.10	[1.03; 1.18]	5.8%	13.1%
Kawahara 2016	1.53	0.7051		4.62	[1.16; 18.40]	0.0%	3.0%
**Pooled HR by Fixed Effects**				**1.04**	**[1.02; 1.06]**	**100.0%**	**--**
**Pooled HR by Random Effects**				**1.51**	**[1.15; 1.98]**	**--**	**100.0%**
**Prediction interval**					**[0.60; 3.84]**		

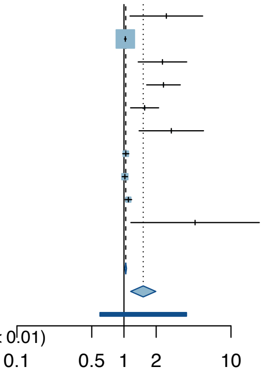

Heterogeneity: I^2 = 84% [72%; 91%], χ^2_9 = 55.80 (p < 0.01)

Tau²= 0.380

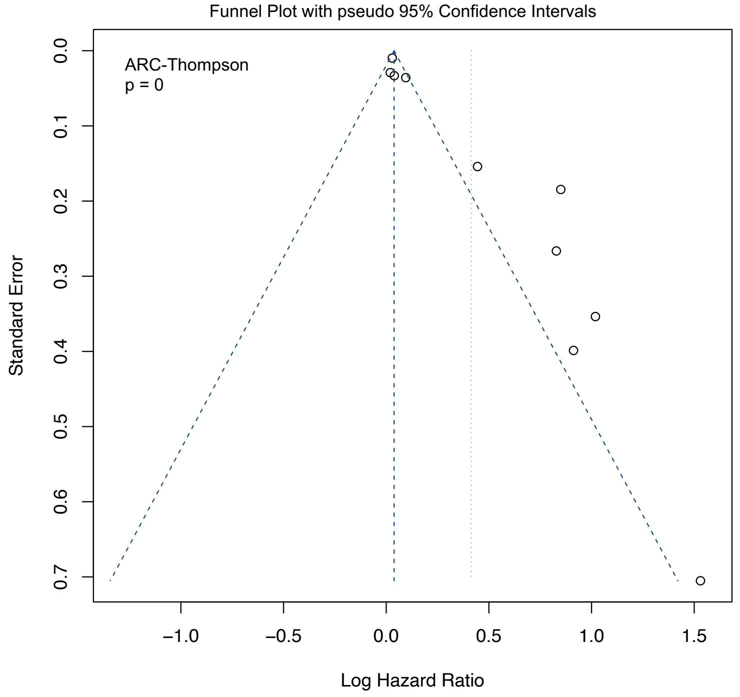

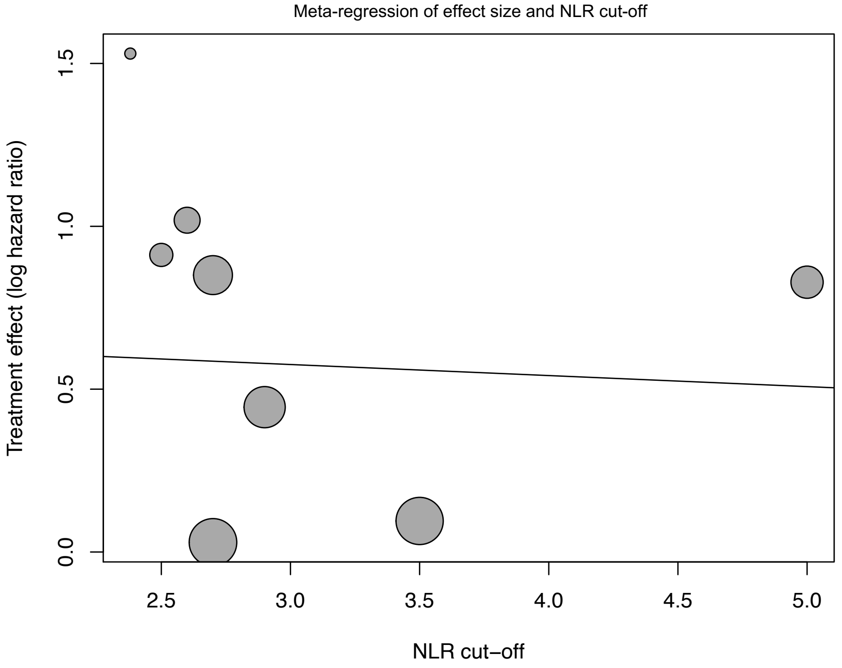

Supplementary Figure 183 - NLR: PFS in Urothelial (Radical Cystectomy)

Study	TE	seTE	HR	95%–CI	Weight (fixed)	Weight (random)
Viers 2014	0.04	0.0193	1.04	[1.00; 1.08]	54.3%	27.8%
Nguyen 2016	0.03	0.0242	1.03	[0.98; 1.08]	34.4%	27.6%
Morizawa 2016	0.96	0.4221	2.61	[1.14; 5.97]	0.1%	3.1%
Bhindi 2016	0.42	0.1349	1.52	[1.17; 1.98]	1.1%	15.5%
D'Andrea 2017	0.18	0.0447	1.20	[1.10; 1.31]	10.1%	25.9%
**Pooled HR by Fixed Effects**			**1.06**	**[1.03; 1.09]**	**100.0%**	**--**
**Pooled HR by Random Effects**			**1.18**	**[1.01; 1.37]**	**--**	**100.0%**
**Prediction interval**				**[0.69; 2.01]**		

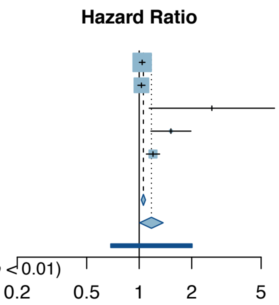

Heterogeneity: $I^2 = 82\%$ [57%; 92%], $\chi^2_4 = 21.73$ ($p < 0.01$)

Tau²= 0.149

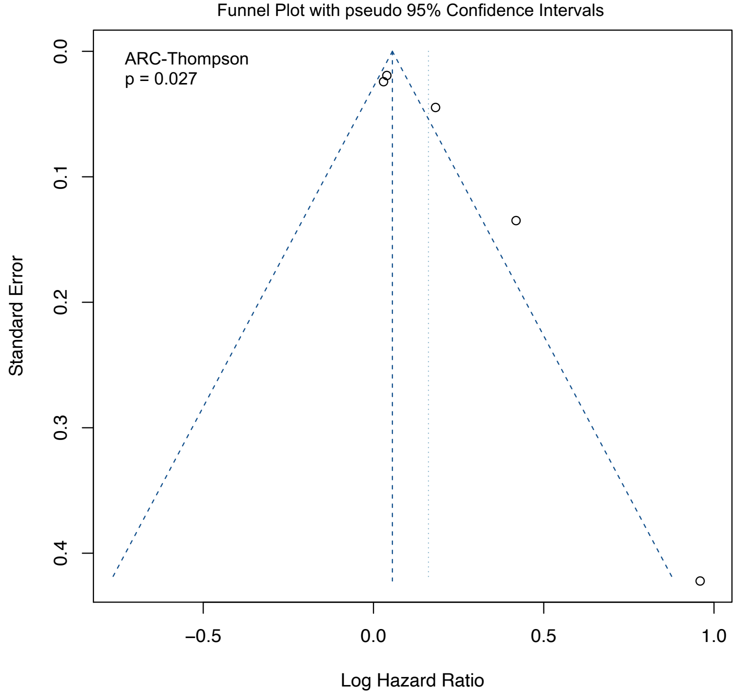

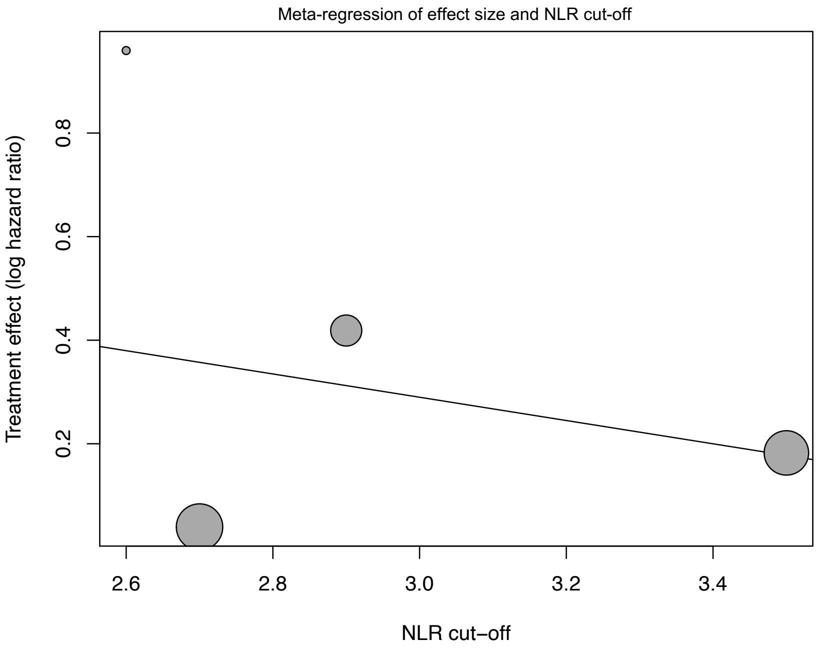

Supplementary Figure 184 - NLR: OS in Urothelial (Chemotherapy)

Study	TE	seTE	HR	95%−CI	Weight (fixed)	Weight (random)
Taguchi 2015	0.40	0.1942	1.49	[1.02; 2.18]	5.5%	9.6%
Rossi 2015	0.55	0.1401	1.74	[1.32; 2.29]	10.6%	15.9%
Sonpavde 2016	0.38	0.1152	1.46	[1.16; 1.83]	15.7%	20.6%
Auvray 2016	0.25	0.0601	1.28	[1.14; 1.44]	57.6%	37.9%
Su 2017	0.46	0.1395	1.59	[1.21; 2.09]	10.7%	16.0%
**Pooled HR by Fixed Effects**			**1.39**	**[1.27; 1.52]**	**100.0%**	**--**
**Pooled HR by Random Effects**			**1.45**	**[1.27; 1.65]**	**--**	**100.0%**
**Prediction interval**				**[1.02; 2.07]**		

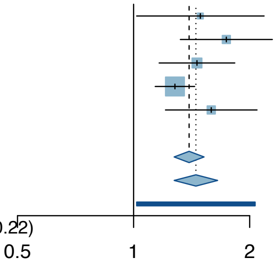

Heterogeneity: $I^2 = 30\%$ [0%; 73%], $\chi^2_4 = 5.69$ ($p = 0.22$)

Tau²= 0.089

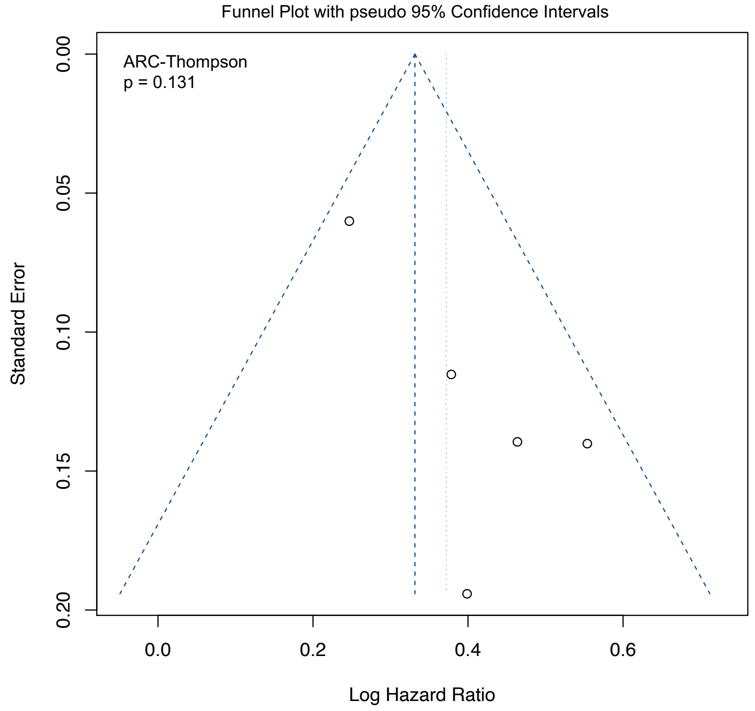

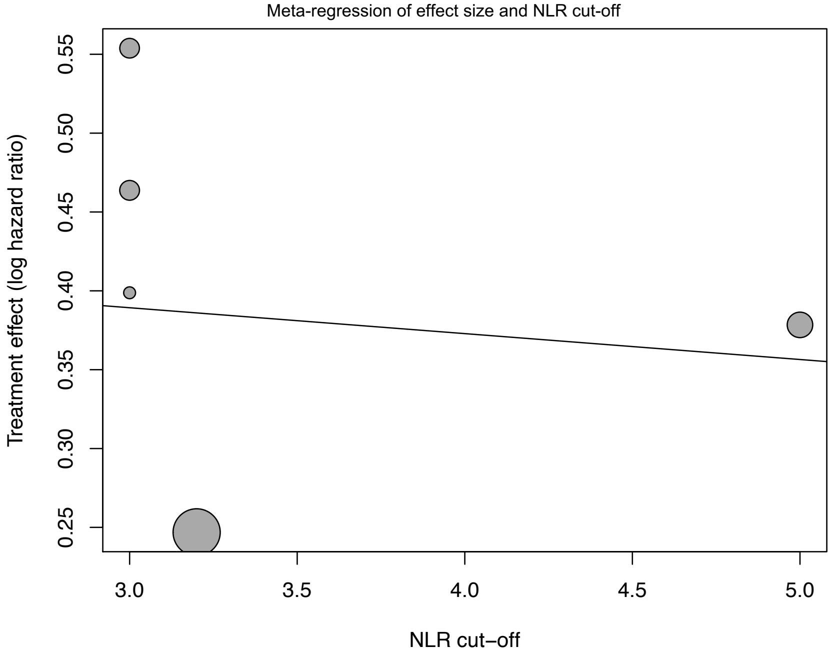

Supplementary Figure 185 **-** NLR: PFS in Urothelial (Chemotherapy)

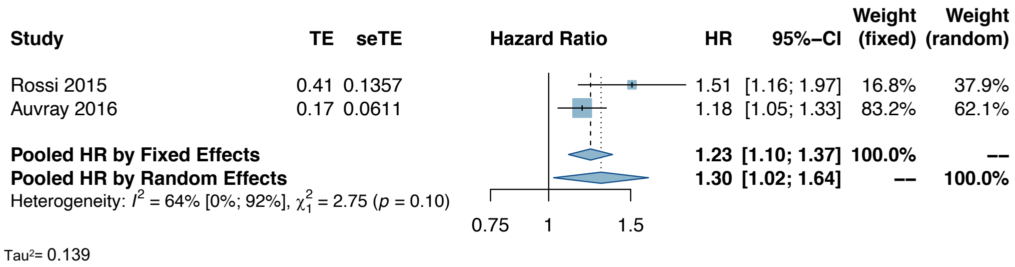

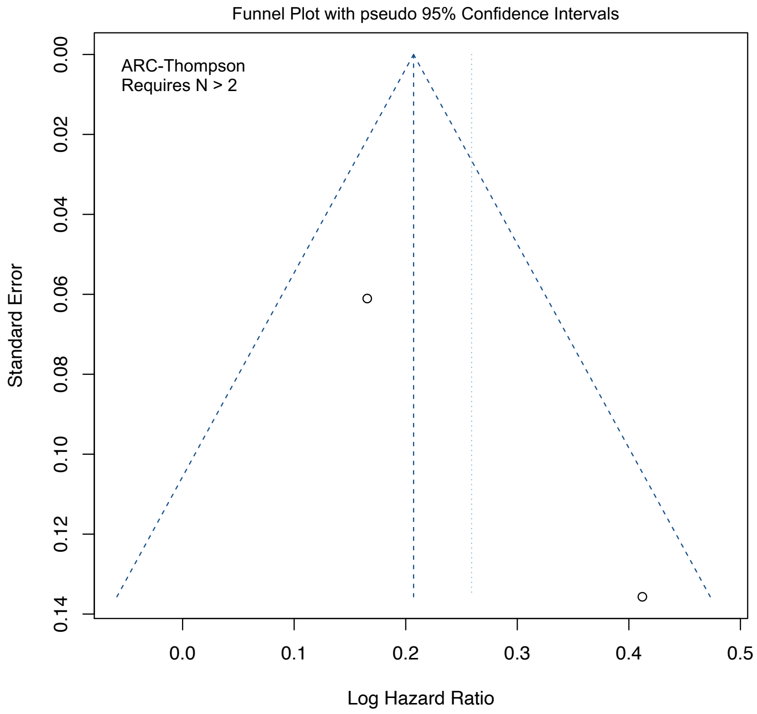

Supplementary Figure 186 - NLR: OS in Urothelial (Nephroureterectomy)

Study	TE	seTE	Hazard Ratio	HR	95%–CI	Weight (fixed)	Weight (random)
Dalpiaz 2014	0.91	0.3262		2.48	[1.31; 4.70]	17.9%	17.9%
Cheng 2016	0.48	0.3037		1.61	[0.89; 2.92]	20.6%	20.6%
Huang 2016	0.46	0.1758		1.58	[1.12; 2.23]	61.5%	61.5%
**Pooled HR by Fixed Effects**				**1.72**	**[1.31; 2.25]**	**100.0%**	**--**
**Pooled HR by Random Effects**				**1.72**	**[1.31; 2.25]**	**--**	**100.0%**
**Prediction interval**					**[0.30; 9.91]**		

Heterogeneity: I^2 = 0% [0%; 86%], χ^2_2 = 1.54 (p = 0.46)

Hazard Ratio axis: 0.2 0.5 1 2 5

Tau²= 0

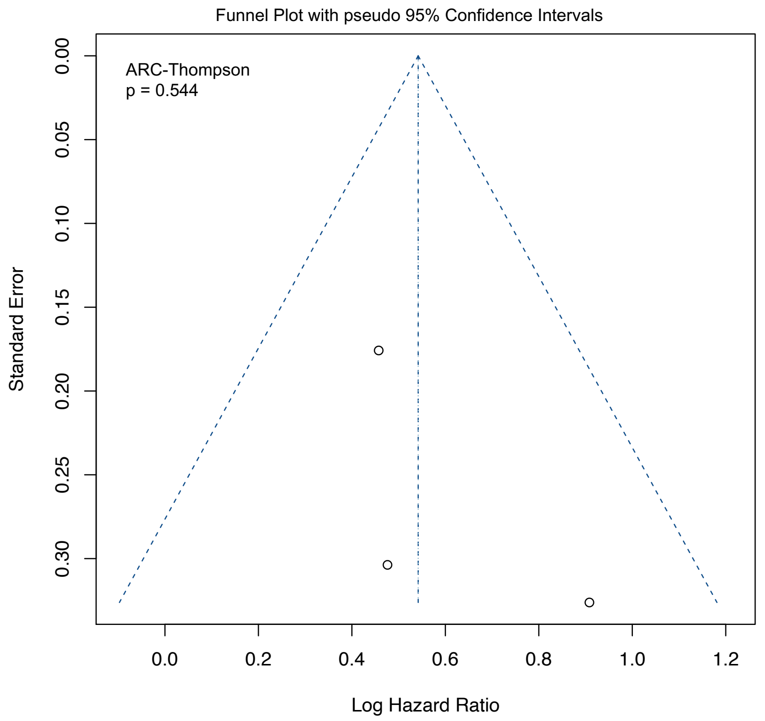

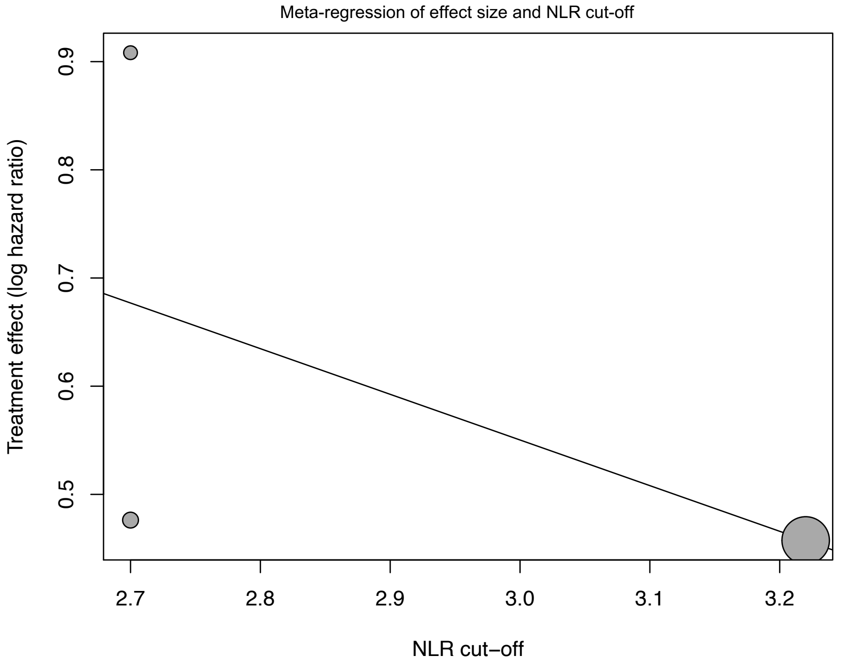

Supplementary Figure 187 - NLR: PFS in Urothelial (Nephroureterectomy)

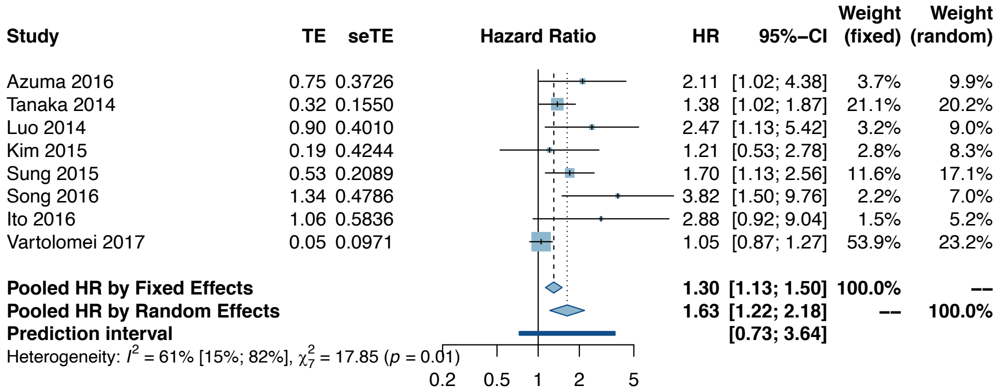

Study	TE	seTE	HR	95%–CI	Weight (fixed)	Weight (random)
Azuma 2016	0.75	0.3726	2.11	[1.02; 4.38]	3.7%	9.9%
Tanaka 2014	0.32	0.1550	1.38	[1.02; 1.87]	21.1%	20.2%
Luo 2014	0.90	0.4010	2.47	[1.13; 5.42]	3.2%	9.0%
Kim 2015	0.19	0.4244	1.21	[0.53; 2.78]	2.8%	8.3%
Sung 2015	0.53	0.2089	1.70	[1.13; 2.56]	11.6%	17.1%
Song 2016	1.34	0.4786	3.82	[1.50; 9.76]	2.2%	7.0%
Ito 2016	1.06	0.5836	2.88	[0.92; 9.04]	1.5%	5.2%
Vartolomei 2017	0.05	0.0971	1.05	[0.87; 1.27]	53.9%	23.2%
**Pooled HR by Fixed Effects**			**1.30**	**[1.13; 1.50]**	**100.0%**	**--**
**Pooled HR by Random Effects**			**1.63**	**[1.22; 2.18]**	**--**	**100.0%**
**Prediction interval**				**[0.73; 3.64]**		

Heterogeneity: I^2 = 61% [15%; 82%], χ^2_7 = 17.85 (p = 0.01)

Tau²= 0.294

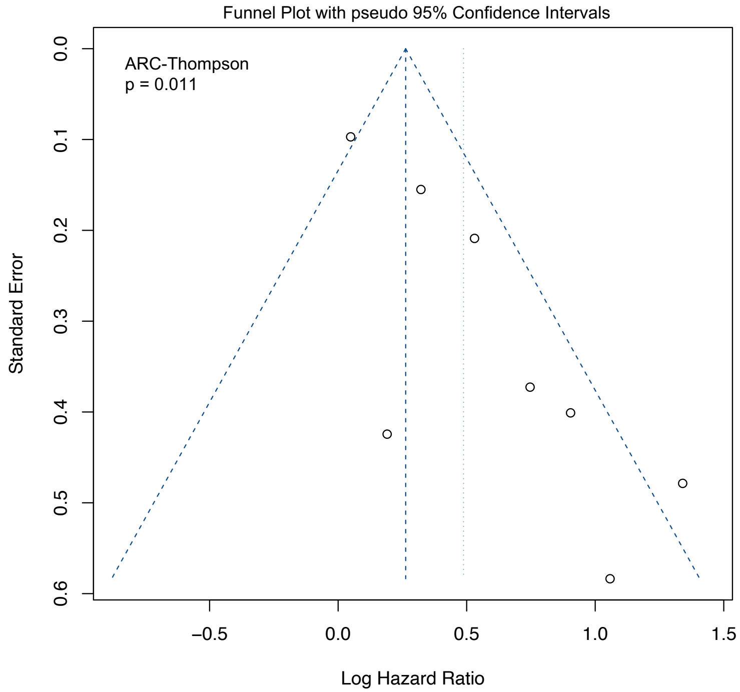

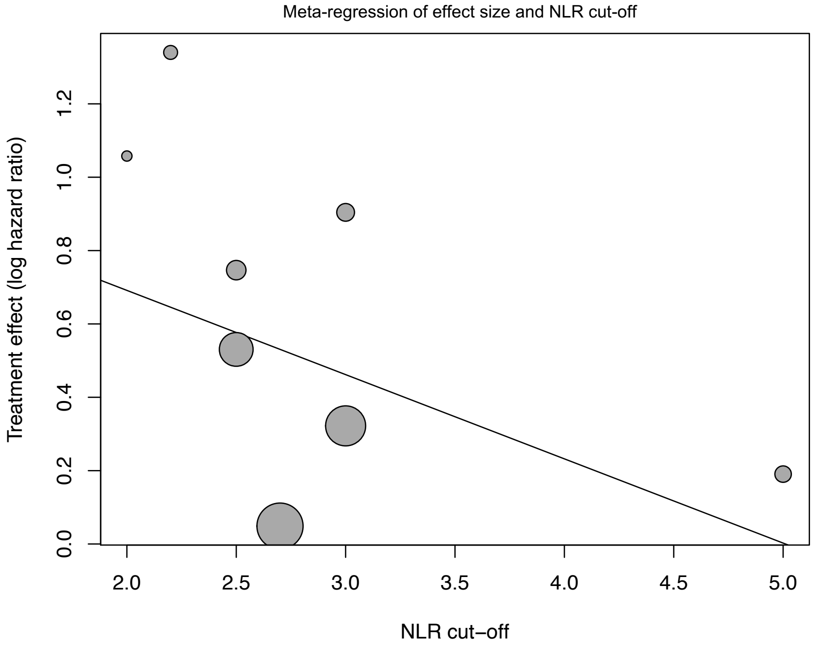

Supplementary Figure 188 - NLR: PFS in Bladder (UTR)

Study	TE	seTE	HR	95%–CI	Weight (fixed)	Weight (random)
Mano 2015	1.26	0.5011	3.52	[1.32; 9.40]	25.6%	25.6%
Ozyalvacli 2015	0.39	0.5582	1.47	[0.49; 4.39]	20.6%	20.6%
Ogihara 2016	0.86	0.3579	2.37	[1.18; 4.78]	50.2%	50.2%
Favilla 2016	1.68	1.3361	5.35	[0.39; 73.39]	3.6%	3.6%
**Pooled HR by Fixed Effects**			**2.45**	**[1.49; 4.02]**	**100.0%**	**--**
**Pooled HR by Random Effects**			**2.45**	**[1.49; 4.02]**	**--**	**100.0%**
**Prediction interval**				**[0.82; 7.28]**		

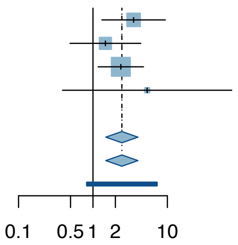

Heterogeneity: $I^2 = 0\%$ [0%; 73%], $\chi^2_3 = 1.71$ ($p = 0.63$)

Tau²= 0

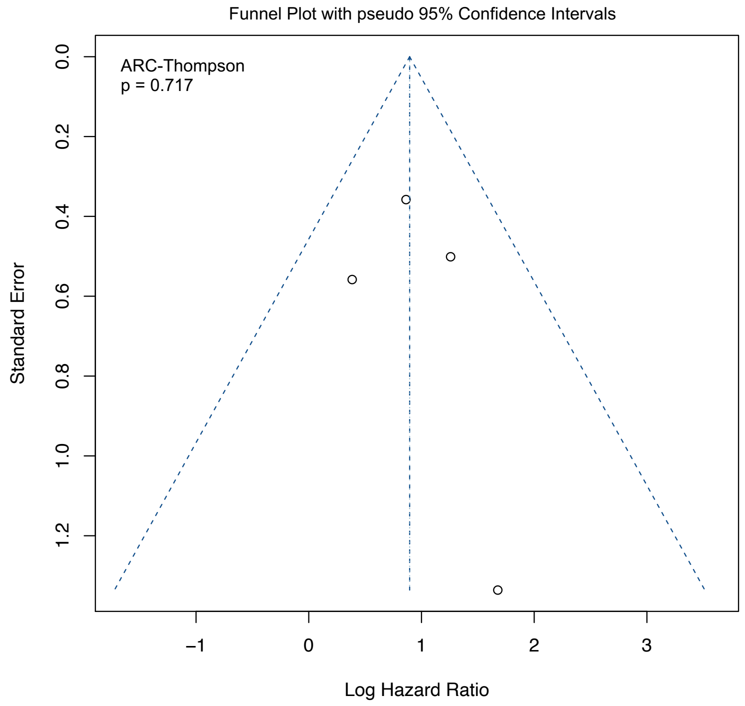

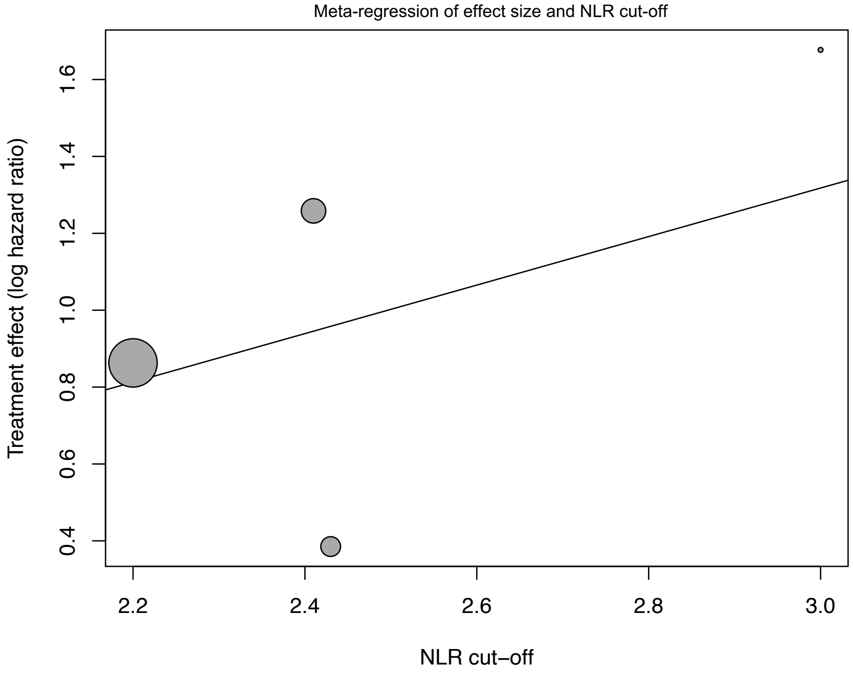

Supplementary Figure 189 - NLR: RFS in Bladder (UTR)

Study	TE	seTE	Hazard Ratio	HR	95%–CI	Weight (fixed)	Weight (random)
Ozyalvacli 2015	1.34	0.4763		3.81	[1.50; 9.69]	5.0%	5.0%
Mano 2015	0.56	0.2612		1.75	[1.05; 2.92]	16.6%	16.6%
Favilla 2016	1.04	0.3260		2.84	[1.50; 5.38]	10.7%	10.7%
Ogihara 2016	0.73	0.1331		2.08	[1.60; 2.70]	63.9%	63.9%
Camtosun 2017	1.59	0.5461		4.90	[1.68; 14.29]	3.8%	3.8%
**Pooled HR by Fixed Effects**				**2.22**	**[1.81; 2.74]**	**100.0%**	**--**
**Pooled HR by Random Effects**				**2.22**	**[1.81; 2.74]**	**--**	**100.0%**
**Prediction interval**					**[1.59; 3.12]**		

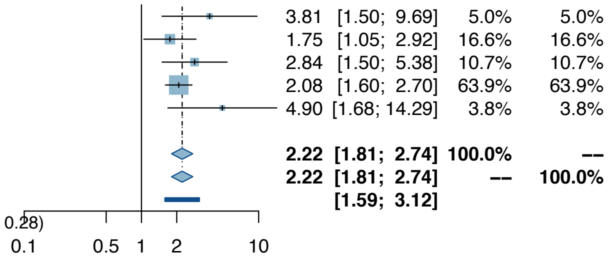

Heterogeneity: I^2 = 20% [0%; 66%], χ_4^2 = 5.03 (p = 0.28)

Tau²= 0.002

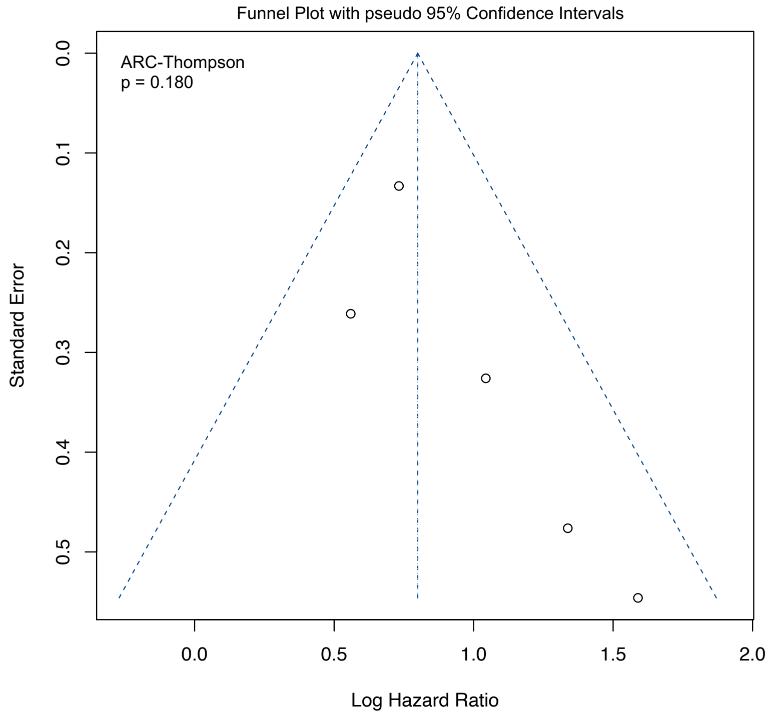

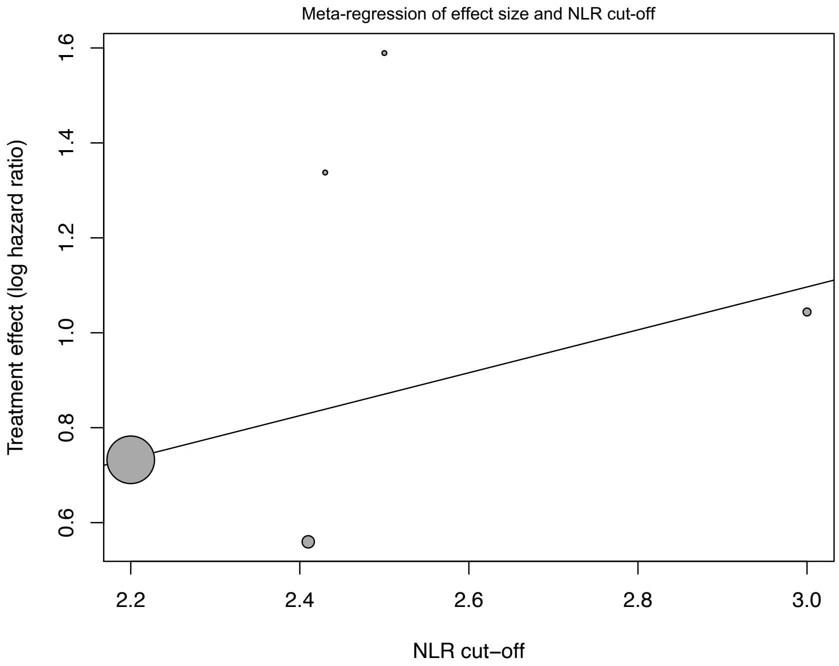

Supplementary Figure 190 - NLR: OS in Bladder

Study	TE	seTE	HR	95%−CI	Weight (fixed)	Weight (random)
D'Andrea A 2016	0.10	0.0444	1.10	[1.01; 1.20]	6.6%	8.8%
Kang MY 2016	0.12	0.0391	1.13	[1.05; 1.22]	8.5%	8.9%
Viers BR 2014	0.03	0.0146	1.03	[1.00; 1.06]	60.6%	9.0%
Tan YG 2017	0.99	0.4120	2.68	[1.20; 6.01]	0.1%	2.6%
Ozcan C 2015	0.59	0.1817	1.80	[1.26; 2.57]	0.4%	6.1%
Zhang GM 2015	0.30	0.3685	1.35	[0.66; 2.78]	0.1%	3.0%
Gondo T 2012	0.67	0.3212	1.95	[1.04; 3.66]	0.1%	3.6%
Lucca I 2016	0.10	0.0482	1.11	[1.01; 1.22]	5.6%	8.7%
Buisan O 2016	0.11	0.0478	1.12	[1.02; 1.23]	5.7%	8.8%
van Kessel EM 2016	0.02	0.3010	1.02	[0.57; 1.84]	0.1%	3.8%
Buisan O 2017	1.02	0.8270	2.78	[0.55; 14.06]	0.0%	0.8%
Krane LS 2013	0.81	0.4362	2.25	[0.96; 5.29]	0.1%	2.3%
Kawahara T 2016	1.53	0.7034	4.62	[1.16; 18.34]	0.0%	1.1%
Ojerholm E 2016	0.02	0.0385	1.02	[0.95; 1.10]	8.8%	8.9%
Hermanns T 2014	0.51	0.1829	1.67	[1.17; 2.39]	0.4%	6.0%
Morizawa T 2016	0.99	0.3344	2.70	[1.40; 5.20]	0.1%	3.4%
Yoshida T 2016	0.85	0.1846	2.34	[1.63; 3.36]	0.4%	6.0%
Hirasawa T 2016	0.26	0.0730	1.30	[1.13; 1.50]	2.4%	8.4%
**Pooled HR by Fixed Effects**			**1.07**	**[1.05; 1.09]**	**100.0%**	**--**
**Pooled HR by Random Effects**			**1.37**	**[1.18; 1.60]**	**--**	**100.0%**
**Prediction interval**				**[0.78; 2.43]**		

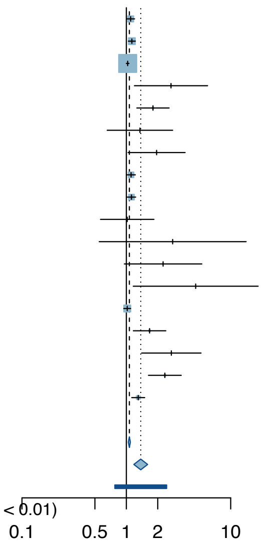

Heterogeneity: I^2 = 78% [65%; 86%], χ^2_{17} = 76.43 (p < 0.01)

Tau²= 0.258

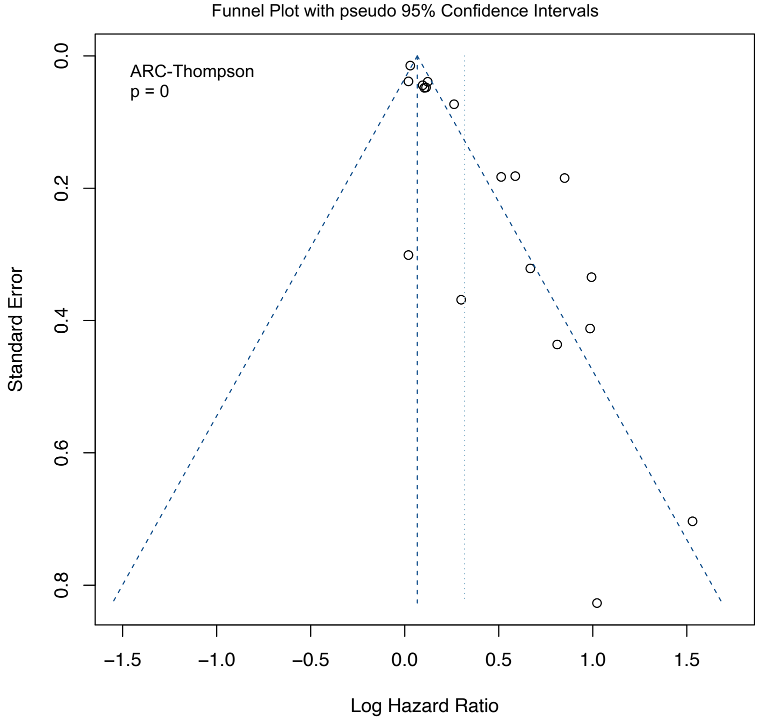

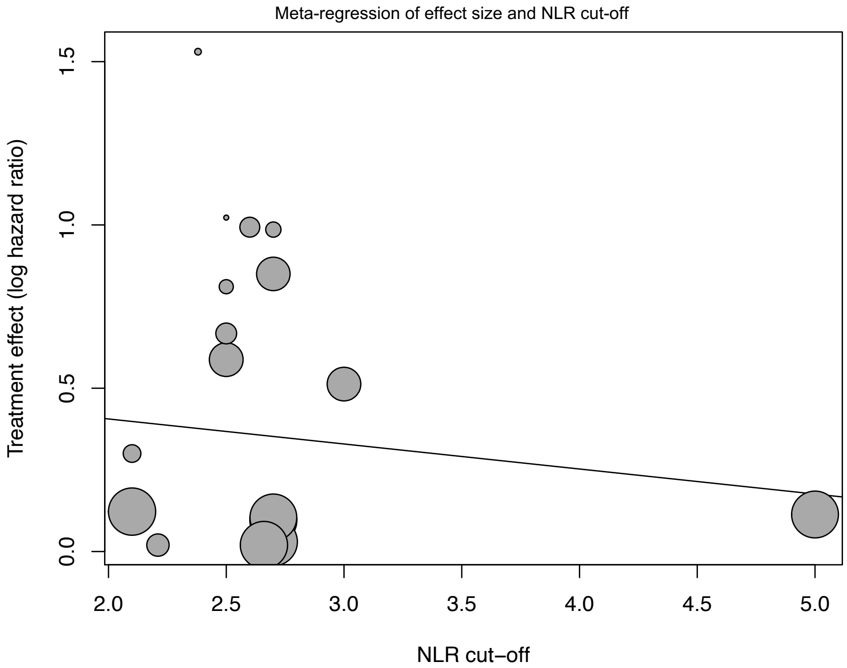

Supplementary Figure 191 - NLR: OS in Bladder (Radical Cystectomy)

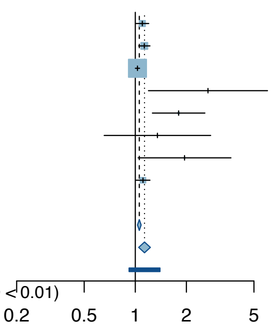

Study	TE	seTE	HR	95%−CI	Weight (fixed)	Weight (random)
D'Andrea A 2016	0.10	0.0444	1.10	[1.01; 1.20]	8.0%	21.4%
Kang MY 2016	0.12	0.0391	1.13	[1.05; 1.22]	10.4%	22.7%
Viers BR 2014	0.03	0.0146	1.03	[1.00; 1.06]	73.9%	27.7%
Tan YG 2017	0.99	0.4120	2.68	[1.20; 6.01]	0.1%	0.9%
Ozcan C 2015	0.59	0.1817	1.80	[1.26; 2.57]	0.5%	4.2%
Zhang GM 2015	0.30	0.3685	1.35	[0.66; 2.78]	0.1%	1.2%
Gondo T 2012	0.67	0.3212	1.95	[1.04; 3.66]	0.2%	1.5%
Lucca I 2016	0.10	0.0482	1.11	[1.01; 1.22]	6.8%	20.4%
**Pooled HR by Fixed Effects**			**1.06**	**[1.03; 1.08]**	**100.0%**	**--**
**Pooled HR by Random Effects**			**1.13**	**[1.05; 1.23]**	**--**	**100.0%**
**Prediction interval**				**[0.92; 1.40]**		

Heterogeneity: $I^2 = 73\%$ [44%; 87%], $\chi^2_7 = 25.63$ ($p < 0.01$)

Tau²= 0.075

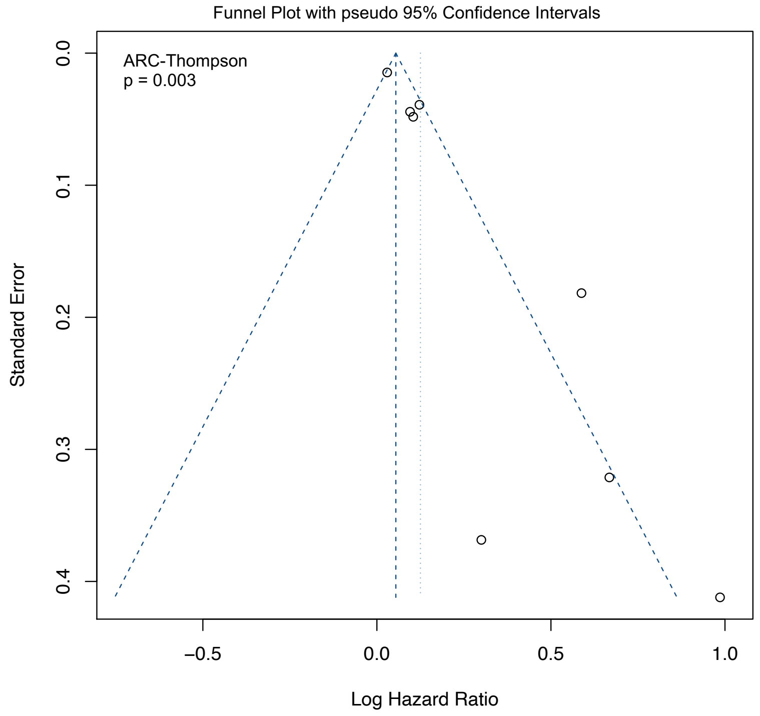

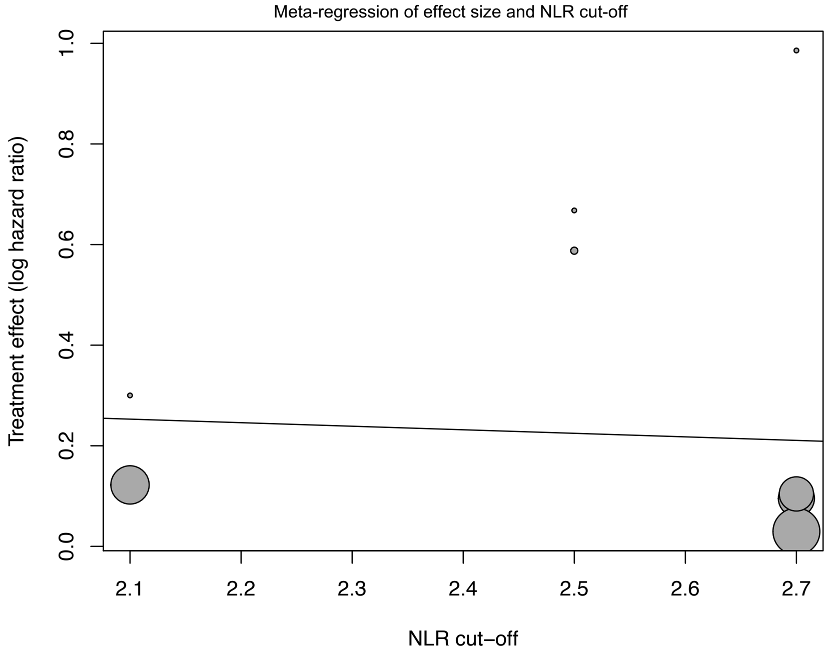

Supplementary Figure 192 - NLR: OS in Bladder (Radical Cystectomy and NAC)

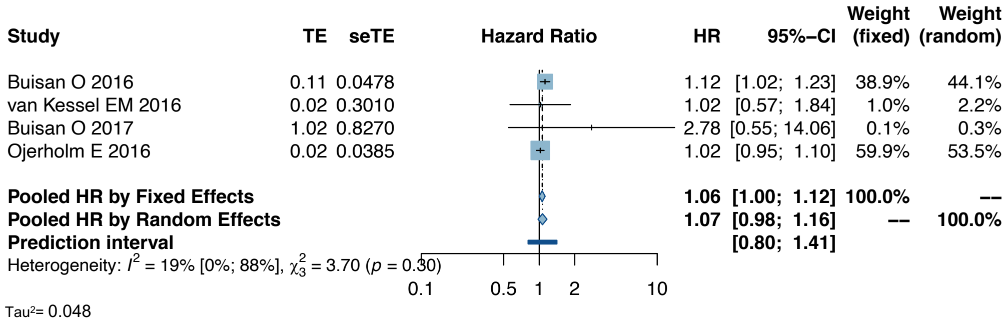

Study	TE	seTE	HR	95%–CI	Weight (fixed)	Weight (random)
Buisan O 2016	0.11	0.0478	1.12	[1.02; 1.23]	38.9%	44.1%
van Kessel EM 2016	0.02	0.3010	1.02	[0.57; 1.84]	1.0%	2.2%
Buisan O 2017	1.02	0.8270	2.78	[0.55; 14.06]	0.1%	0.3%
Ojerholm E 2016	0.02	0.0385	1.02	[0.95; 1.10]	59.9%	53.5%
**Pooled HR by Fixed Effects**			**1.06**	**[1.00; 1.12]**	**100.0%**	**--**
**Pooled HR by Random Effects**			**1.07**	**[0.98; 1.16]**	**--**	**100.0%**
**Prediction interval**				**[0.80; 1.41]**		

Heterogeneity: $I^2 = 19\%$ [0%; 88%], $\chi^2_3 = 3.70$ ($p = 0.30$)

Tau²= 0.048

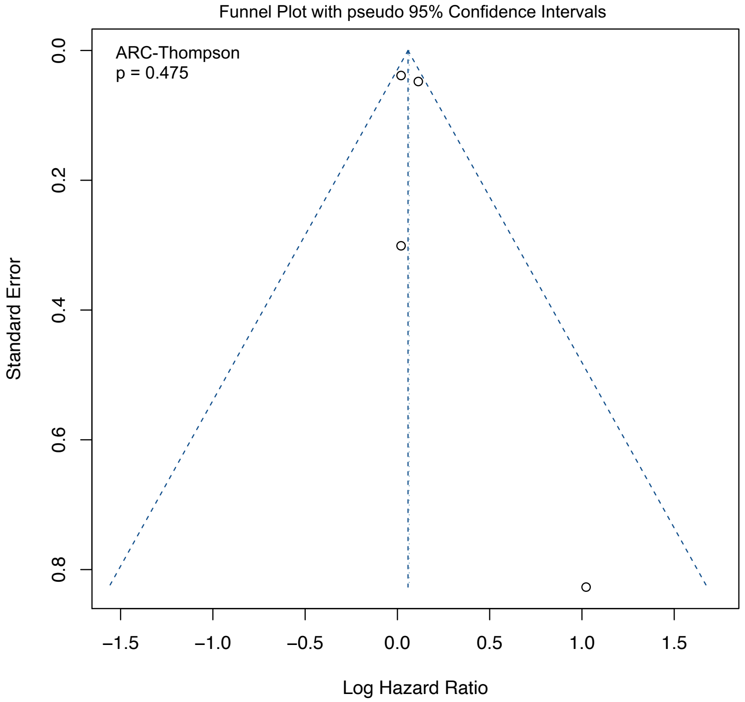

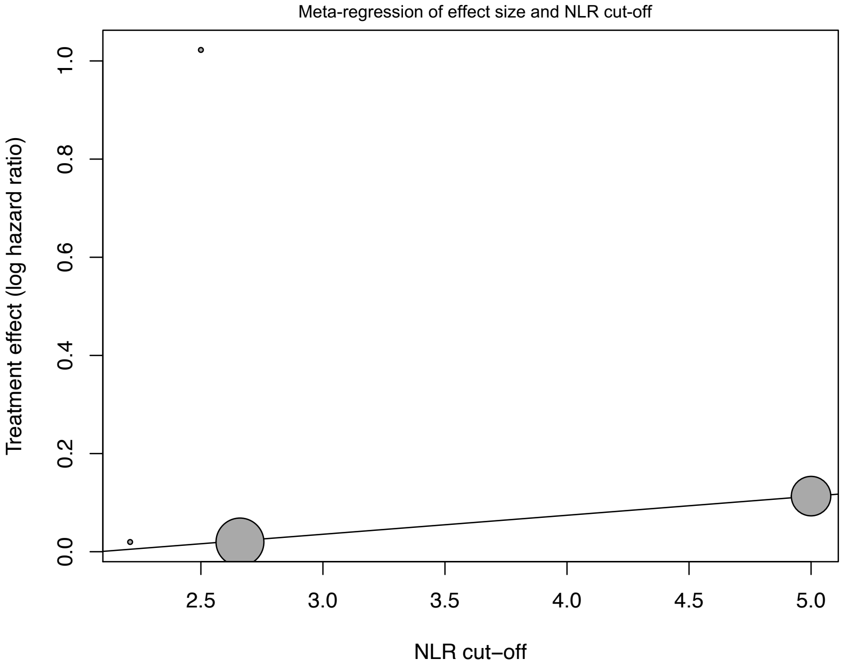

Supplementary Figure 193 - NLR: PFS in Prostate

Study	TE	seTE	HR	95%–CI	Weight (fixed)	Weight (random)
Sun 2017	0.69	0.3275	2.00	[1.05; 3.80]	14.2%	14.4%
Pei 2017	0.60	0.2673	1.83	[1.08; 3.09]	21.3%	19.1%
Mehra 2017	1.25	0.4281	3.50	[1.51; 8.10]	8.3%	9.5%
Buttigliero 2017	0.45	0.3166	1.57	[0.84; 2.92]	15.2%	15.1%
Boegemann 2017	0.10	0.3050	1.10	[0.61; 2.00]	16.3%	16.0%
Yao 2015	0.87	0.3848	2.38	[1.12; 5.06]	10.3%	11.2%
Langsenlehner 2015	1.13	0.3230	3.09	[1.64; 5.82]	14.6%	14.7%
**Pooled HR by Fixed Effects**			**1.95**	**[1.53; 2.48]**	**100.0%**	**--**
**Pooled HR by Random Effects**			**1.97**	**[1.49; 2.62]**	**--**	**100.0%**
**Prediction interval**				**[1.06; 3.69]**		

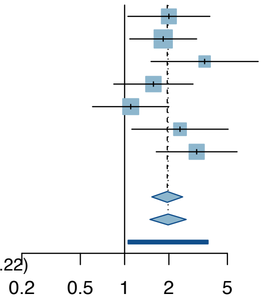

Heterogeneity: $I^2 = 27\%$ [0%; 68%], $\chi^2_6 = 8.22$ ($p = 0.22$)

Tau²= 0.196

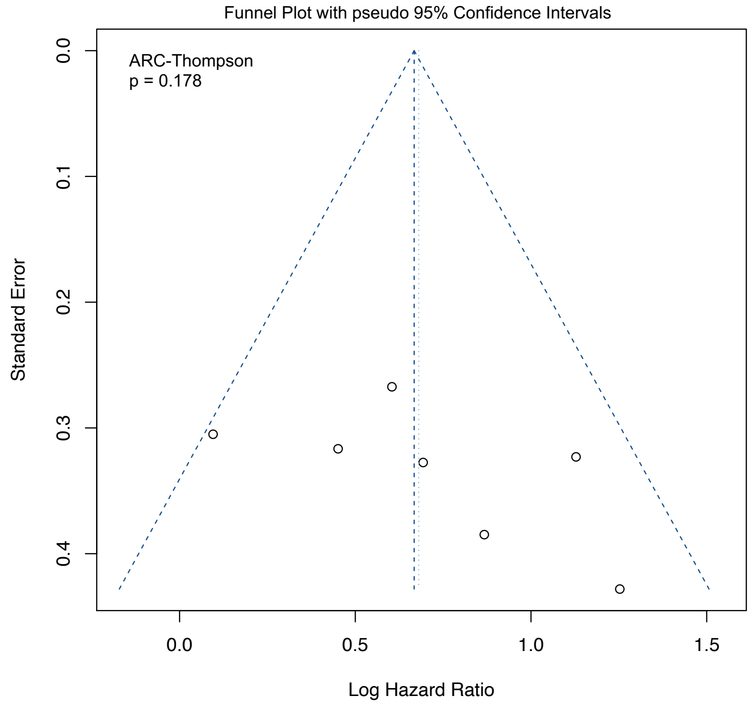

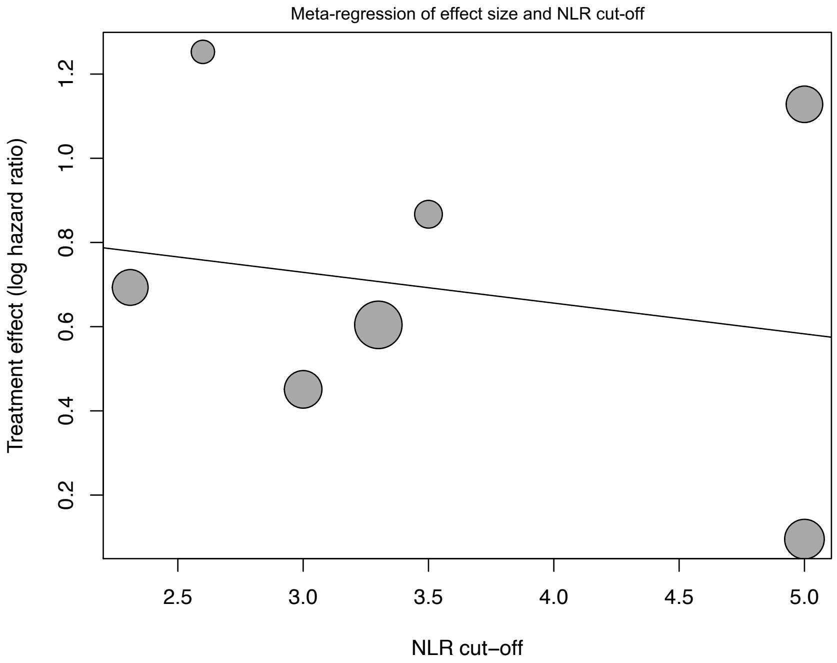

Supplementary Figure 194 - NLR: OS in Prostate CR(Abitaterone)

Study	TE	seTE	HR	95%-CI	Weight (fixed)	Weight (random)
**Adjustment = Multivariate**						
Boegemann 2019	0.17	0.2666	1.18	[0.70; 1.99]	6.2%	9.1%
Schiff 2019	0.54	0.2147	1.72	[1.13; 2.62]	9.6%	11.6%
Onal 2019	1.14	0.3233	3.12	[1.66; 5.88]	4.2%	7.0%
Fan 2017	0.88	0.4323	2.40	[1.03; 5.60]	2.4%	4.5%
Yasui 2017	0.99	0.4353	2.68	[1.14; 6.29]	2.3%	4.4%
Boegemann 2017	0.53	0.2898	1.70	[0.96; 3.00]	5.3%	8.1%
Templeton 2014	0.50	0.1421	1.65	[1.25; 2.18]	22.0%	16.5%
**Pooled HR by Fixed Effects**			**1.75**	**[1.46; 2.10]**	**52.0%**	**---**
**Pooled HR by Random Effects**			**1.75**	**[1.46; 2.10]**	**---**	**61.2%**
Heterogeneity: $I^2 = 15\%$ [0%; 59%], $\chi^2_6 = 7.06$ ($p = 0.32$)						
**Adjustment = Univariate**						
Lolli 2016	0.76	0.1730	2.13	[1.52; 2.99]	14.8%	14.3%
Martinez 2017	0.79	0.3383	2.20	[1.13; 4.27]	3.9%	6.5%
Loubersac 2019	0.19	0.1230	1.21	[0.95; 1.54]	29.3%	18.0%
**Pooled HR by Fixed Effects**			**1.51**	**[1.25; 1.83]**	**48.0%**	**---**
**Pooled HR by Random Effects**			**1.70**	**[1.11; 2.59]**	**---**	**38.8%**
Heterogeneity: $I^2 = 76\%$ [22%; 93%], $\chi^2_2 = 8.43$ ($p = 0.01$)						
**Pooled HR by Fixed Effects**			**1.63**	**[1.43; 1.86]**	**100.0%**	**---**
**Pooled HR by Random Effects**			**1.75**	**[1.44; 2.13]**	**---**	**100.0%**
**Prediction interval**				**[1.03; 2.96]**		

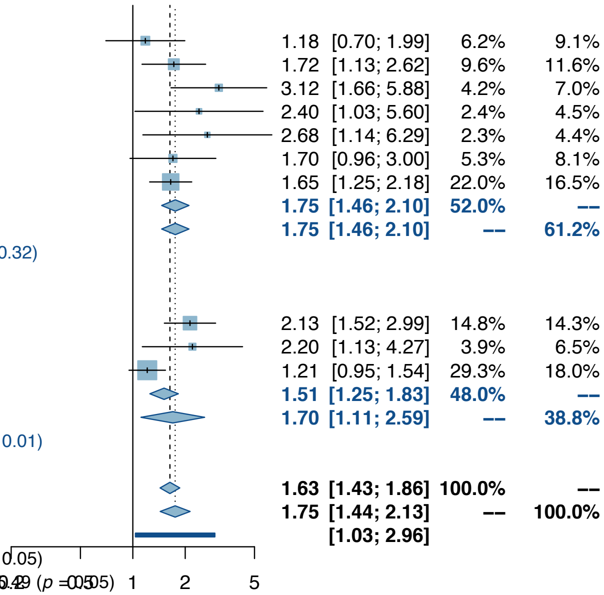

Heterogeneity: $I^2 = 46\%$ [0%; 74%], $\chi^2_9 = 16.74$ ($p = 0.05$)
Residual heterogeneity: $I^2 = 48\%$ [0%; 76%], $\chi^2_8 =$ 1[illegible]9 ($p = 0.05$)

Tau²= 0.204

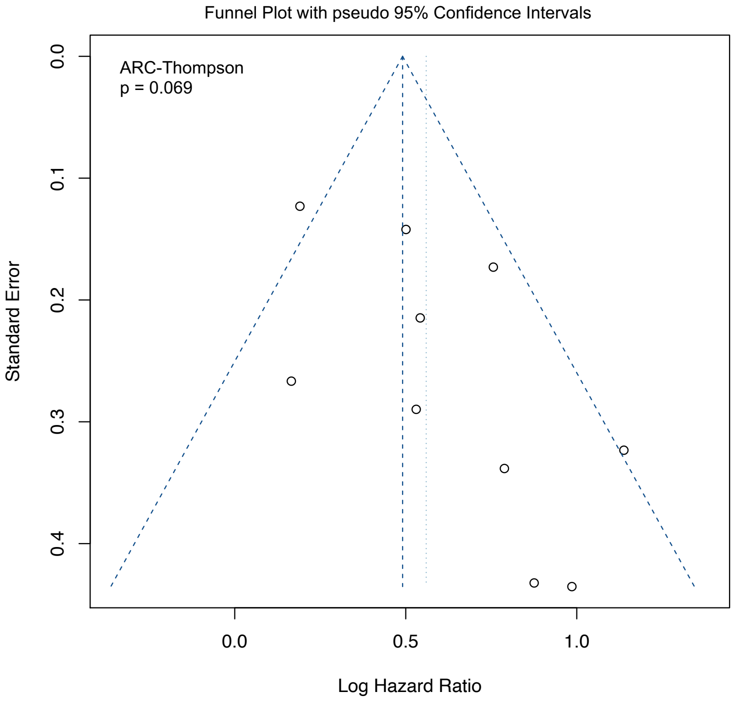

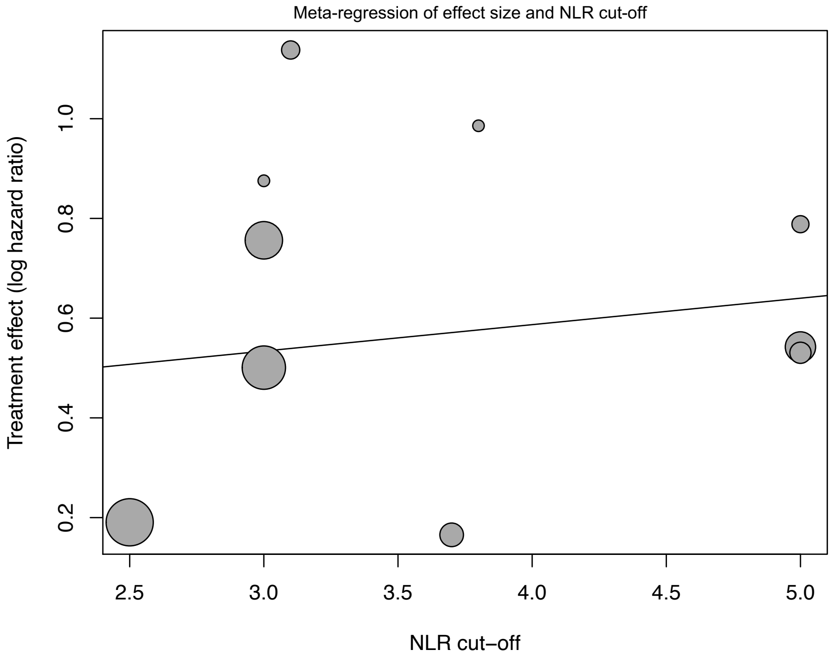

Supplementary Figure 195 - NLR: PFS in Prostate CR (Abiterone)

Study	TE	seTE	Hazard Ratio	HR	95%–CI	Weight (fixed)	Weight (random)
Boegemann 2017	0.10	0.3050		1.10	[0.61; 2.00]	35.6%	45.9%
Onal 2019	0.81	0.2269		2.25	[1.44; 3.51]	64.4%	54.1%
**Pooled HR by Fixed Effects**				**1.74**	**[1.22; 2.49]**	**100.0%**	**--**
**Pooled HR by Random Effects**				**1.62**	**[0.81; 3.26]**	**--**	**100.0%**

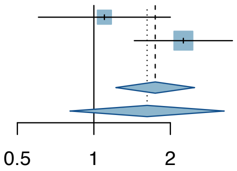

Heterogeneity: $I^2 = 72\%$ [0%; 94%], $\chi^2_1 = 3.54$ ($p = 0.06$)

Tau²= 0.429

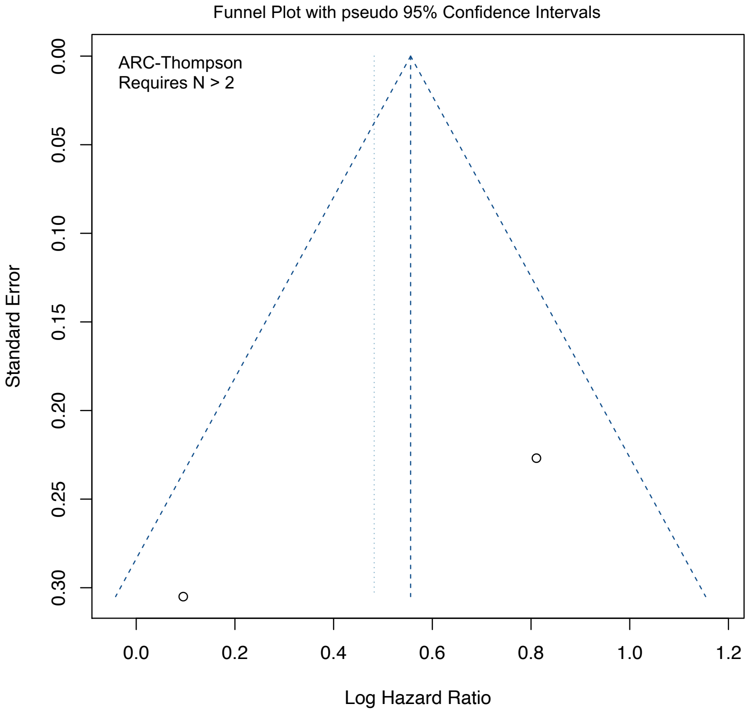

Supplementary Figure 196 - NLR: OS in Prostate CR (Enzalutamide)

Study	TE	seTE	HR	95%−CI	Weight (fixed)	Weight (random)
**Adjustment = Multivariate**						
Choi 2018	0.29	0.0541	1.34	[1.21; 1.49]	51.7%	29.5%
Armstrong 2018	0.37	0.0959	1.45	[1.20; 1.75]	16.5%	23.5%
Conteduca 2016	0.25	0.0803	1.29	[1.10; 1.51]	23.5%	25.8%
**Pooled HR by Fixed Effects**			**1.35**	**[1.24; 1.46]**	**91.7%**	**---**
**Pooled HR by Random Effects**			**1.35**	**[1.24; 1.46]**	**---**	**78.9%**
Heterogeneity: $I^2 = 0\%$ [0%; 77%], $\chi^2_2 = 0.89$ ($p = 0.64$)						
**Adjustment = Univariate**						
Kumano 2019	0.84	0.4110	2.31	[1.03; 5.17]	0.9%	3.8%
Conteduca 2018	0.71	0.1430	2.04	[1.54; 2.70]	7.4%	17.3%
**Pooled HR by Fixed Effects**			**2.07**	**[1.59; 2.69]**	**8.3%**	**---**
**Pooled HR by Random Effects**			**2.07**	**[1.59; 2.69]**	**---**	**21.1%**
Heterogeneity: $I^2 = 0\%$, $\chi^2_1 = 0.08$ ($p = 0.78$)						
**Pooled HR by Fixed Effects**			**1.39**	**[1.29; 1.51]**	**100.0%**	**---**
**Pooled HR by Random Effects**			**1.48**	**[1.26; 1.76]**	**---**	**100.0%**
**Prediction interval**				**[0.86; 2.56]**		

Heterogeneity: $I^2 = 61\%$ [0%; 85%], $\chi^2_4 = 10.23$ ($p = 0.04$)

Residual heterogeneity: $I^2 = 0\%$ [0%; 53%], $\chi^2_3 = 0.92$ ($p = 0.85$)

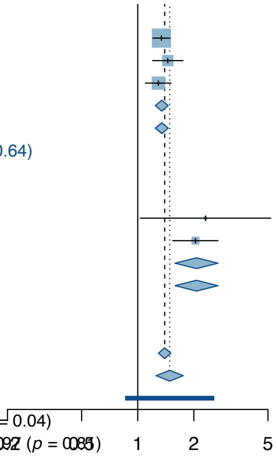

Tau²= 0.148

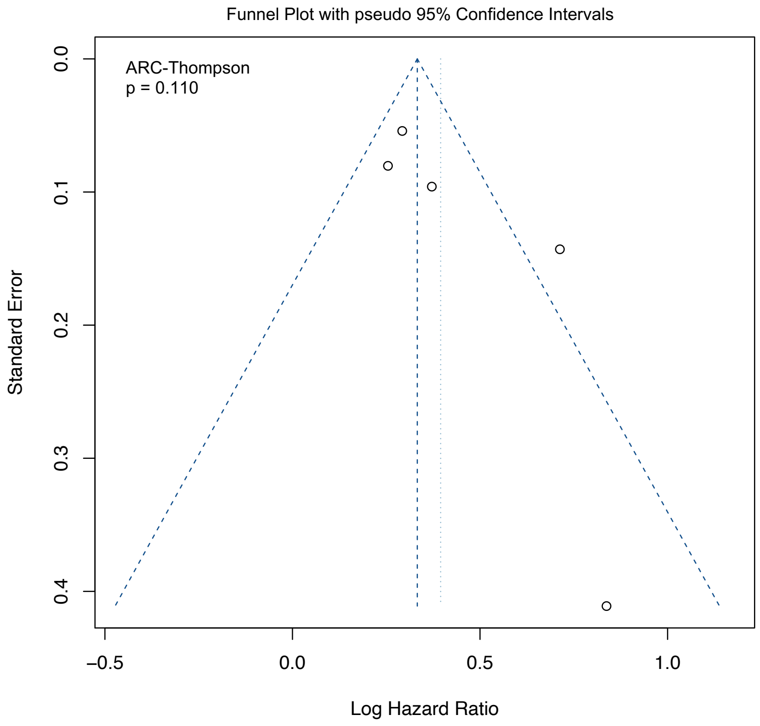

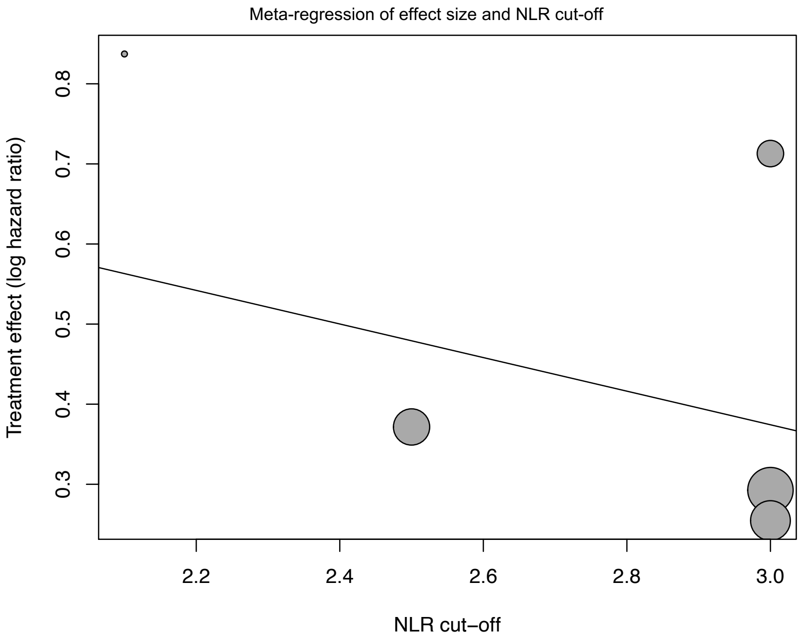

Supplementary Figure 197 - NLR: PFS in Prostate CR (Enzalutamide)

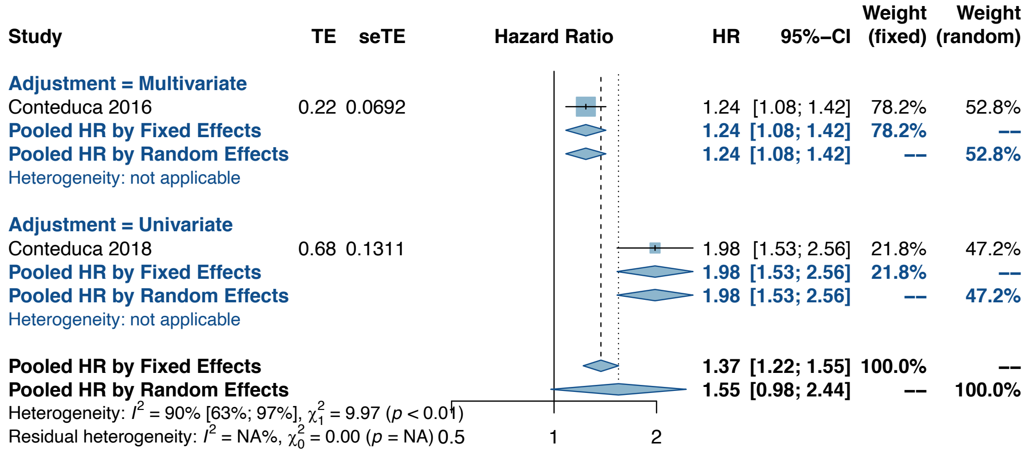

Tau²= 0.314

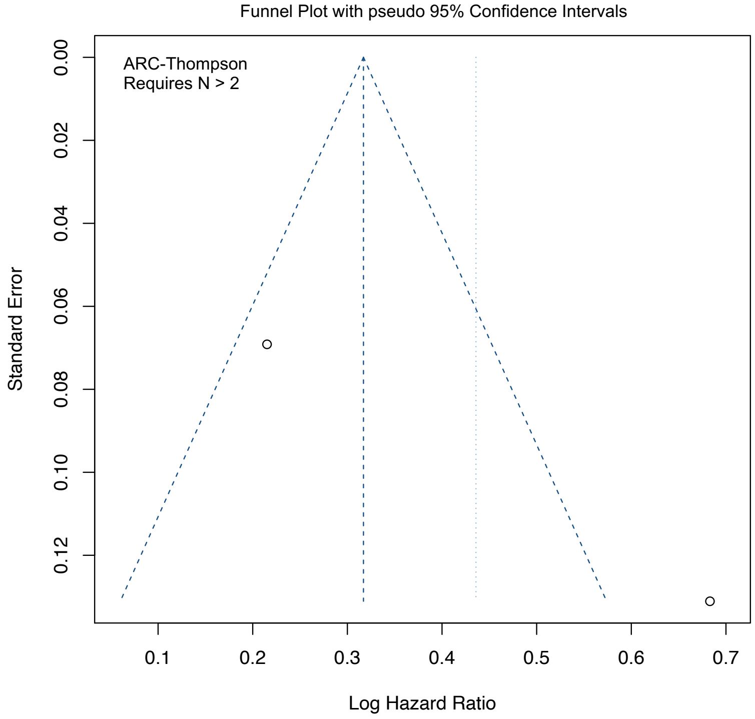

Supplementary Figure 198 - NLR: OS in Prostate (Metastatic)

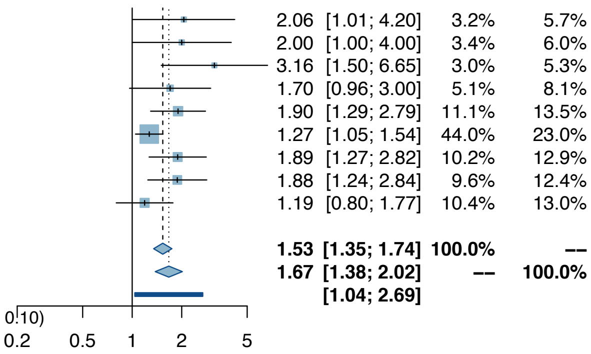

Study	TE	seTE	HR	95%−CI	Weight (fixed)	Weight (random)
Pei 2017	0.72	0.3635	2.06	[1.01; 4.20]	3.2%	5.7%
Mehra 2017	0.69	0.3536	2.00	[1.00; 4.00]	3.4%	6.0%
Buttigliero 2017	1.15	0.3796	3.16	[1.50; 6.65]	3.0%	5.3%
Boegemann 2017	0.53	0.2898	1.70	[0.96; 3.00]	5.1%	8.1%
Lolli 2016	0.64	0.1960	1.90	[1.29; 2.79]	11.1%	13.5%
Lorente 2015	0.24	0.0983	1.27	[1.05; 1.54]	44.0%	23.0%
Templeton 2014	0.64	0.2042	1.89	[1.27; 2.82]	10.2%	12.9%
Nuhn 2014	0.63	0.2105	1.88	[1.24; 2.84]	9.6%	12.4%
Linton 2013	0.17	0.2026	1.19	[0.80; 1.77]	10.4%	13.0%
**Pooled HR by Fixed Effects**			**1.53**	**[1.35; 1.74]**	**100.0%**	**--**
**Pooled HR by Random Effects**			**1.67**	**[1.38; 2.02]**	**--**	**100.0%**
**Prediction interval**				**[1.04; 2.69]**		

Heterogeneity: I^2 = 40% [0%; 73%], χ^2_8 = 13.40 (p = 0.10)

Tau²= 0.176

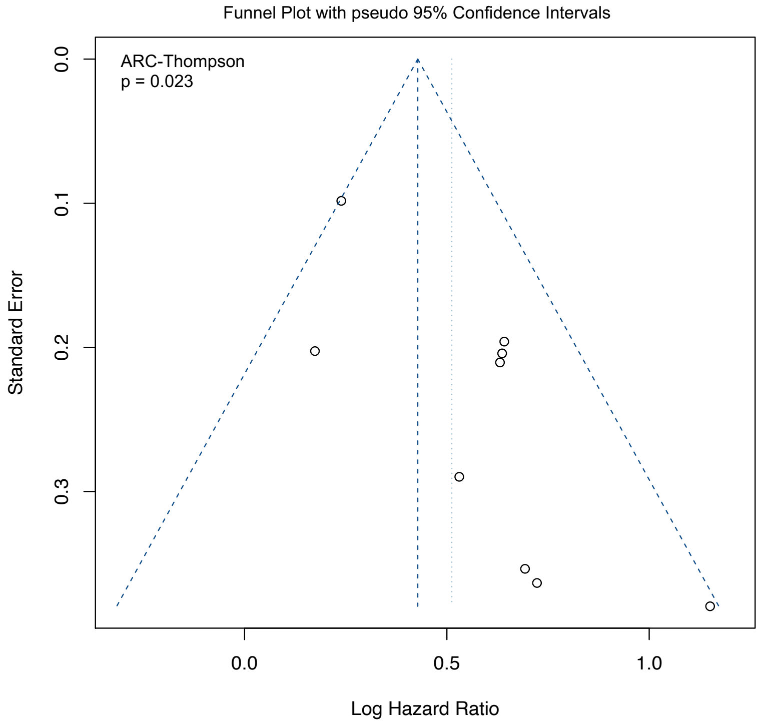

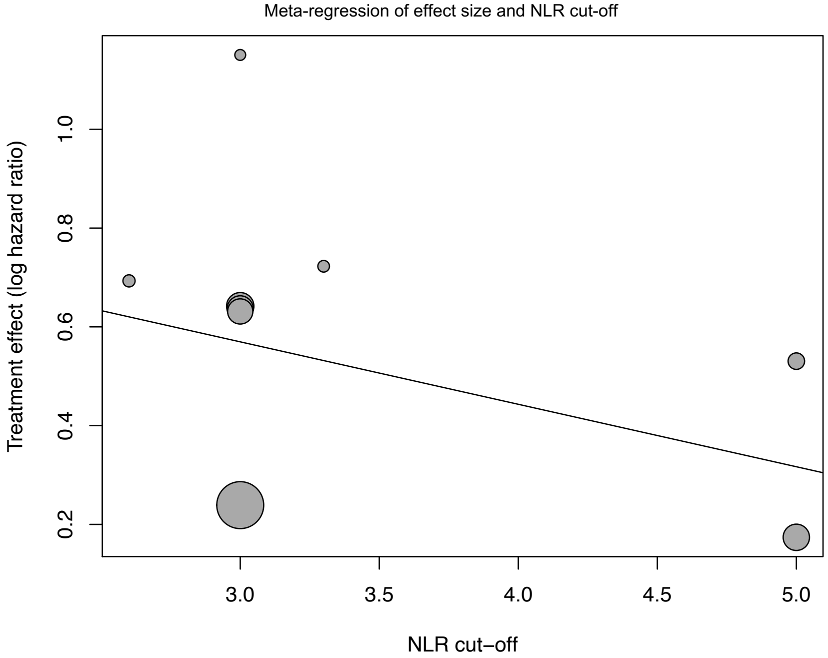

Supplementary Figure 199 - NLR: DFS in Thyroid

Study	TE	seTE	HR	95%–CI	Weight (fixed)	Weight (random)
**Adjustment = Multivariate**						
Kim JY 2014	1.99	1.0659	7.35	[0.91; 59.37]	0.7%	7.4%
Lee KH 2019	1.09	0.5130	2.96	[1.08; 8.09]	3.0%	15.7%
**Pooled HR by Fixed Effects**			**3.51**	**[1.42; 8.69]**	**3.6%**	**--**
**Pooled HR by Random Effects**			**3.51**	**[1.42; 8.69]**	**--**	**23.1%**
Heterogeneity: $I^2 = 0\%$, $\chi^2_1 = 0.59$ ($p = 0.44$)						
**Adjustment = Univariate**						
Lang BH 2014	0.12	0.0945	1.13	[0.94; 1.36]	87.2%	23.5%
Kim JY 2014	2.17	1.0606	8.79	[1.10; 70.27]	0.7%	7.4%
Jiang K 2017	0.31	0.4866	1.36	[0.52; 3.53]	3.3%	16.3%
Lee KH 2019	−0.99	0.6211	0.37	[0.11; 1.25]	2.0%	13.6%
Lee KH 2019	1.19	0.4994	3.28	[1.23; 8.73]	3.1%	16.0%
**Pooled HR by Fixed Effects**			**1.17**	**[0.98; 1.39]**	**96.4%**	**--**
**Pooled HR by Random Effects**			**1.47**	**[0.67; 3.23]**	**--**	**76.9%**
Heterogeneity: $I^2 = 65\%$ [9%; 87%], $\chi^2_4 = 11.54$ ($p = 0.02$)						
**Pooled HR by Fixed Effects**			**1.21**	**[1.02; 1.44]**	**100.0%**	**--**
**Pooled HR by Random Effects**			**1.85**	**[0.93; 3.65]**	**--**	**100.0%**
**Prediction interval**				**[0.24; 14.09]**		
Heterogeneity: $I^2 = 66\%$ [24%; 85%], $\chi^2_6 = 17.61$ ($p < 0.01$)						
Residual heterogeneity: $I^2 = 59\%$ [0%; 83%], $\chi^2_5 = 12.13$ ($p = 0.03$)						

Hazard Ratio axis: 0.1, 0.5, 1, 2, 10

Tau²= 0.710

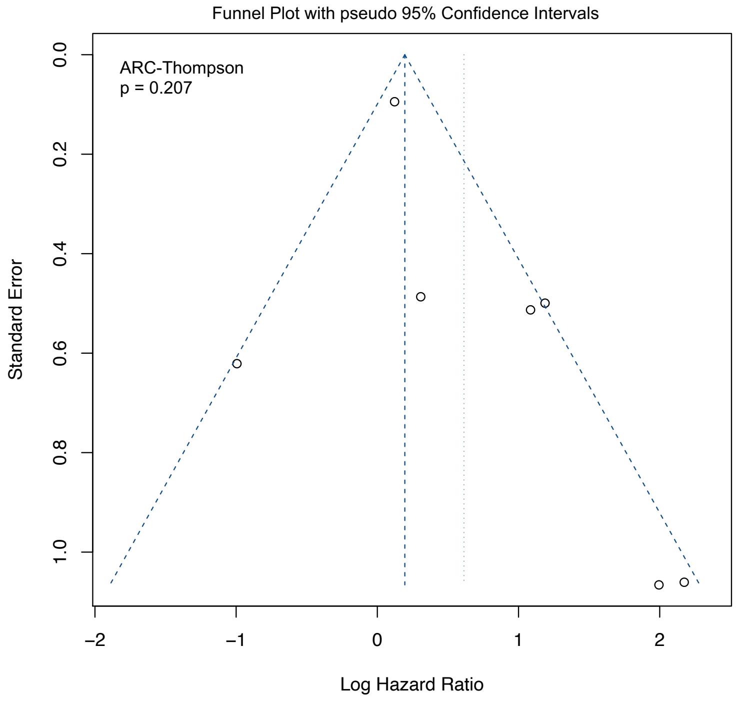

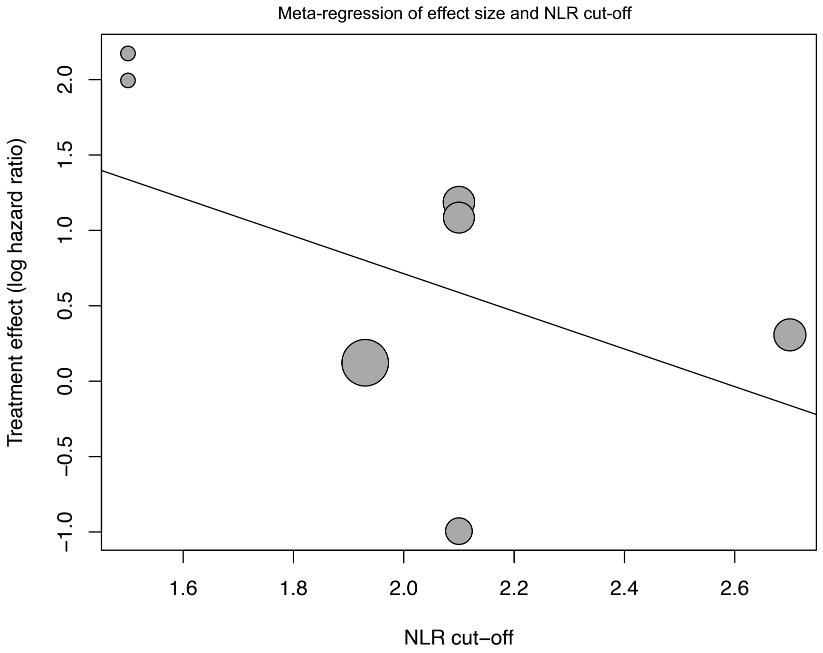

Supplementary Figure 200 - NLR: OS in Ovarian

Study	TE	seTE	HR	95%−CI	Weight (fixed)	Weight (random)
Asher 2011	−0.14	0.2571	0.87	[0.53; 1.44]	0.4%	6.8%
Badora−Rybicka 2016	0.09	0.0575	1.09	[0.97; 1.22]	8.7%	9.2%
Cho 2009	2.13	1.0415	8.42	[1.09; 64.84]	0.0%	1.3%
Eo 2015	0.70	0.2727	2.01	[1.18; 3.43]	0.4%	6.5%
Feng 2016	0.17	0.1181	1.19	[0.94; 1.50]	2.1%	8.7%
Gungorduk 2015	1.16	0.5443	3.20	[1.10; 9.30]	0.1%	3.4%
Kim 2015	0.34	0.1358	1.41	[1.08; 1.84]	1.6%	8.5%
Li 2017	1.24	0.4090	3.45	[1.55; 7.69]	0.2%	4.7%
Miao 2016	0.48	0.1788	1.62	[1.14; 2.30]	0.9%	7.9%
Paik 2016	0.07	0.0187	1.07	[1.03; 1.11]	82.3%	9.4%
Thavaramara 2011	−0.36	0.4218	0.70	[0.31; 1.60]	0.2%	4.6%
Wang 2015	2.15	0.5690	8.57	[2.81; 26.14]	0.1%	3.2%
Wang 2016	1.21	0.4506	3.37	[1.39; 8.15]	0.1%	4.3%
Williams 2014	0.31	0.1278	1.37	[1.07; 1.76]	1.8%	8.6%
Zhang 2014	0.94	0.3808	2.56	[1.21; 5.40]	0.2%	5.1%
Zhang 2015	0.77	0.1737	2.17	[1.54; 3.05]	1.0%	8.0%
**Pooled HR by Fixed Effects**			**1.11**	**[1.07; 1.14]**	**100.0%**	**--**
**Pooled HR by Random Effects**			**1.65**	**[1.29; 2.11]**	**--**	**100.0%**
**Prediction interval**				**[0.65; 4.15]**		

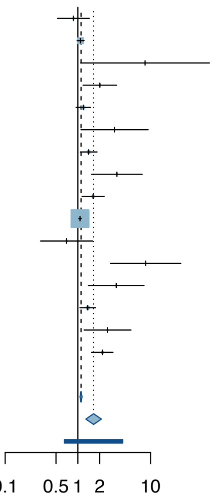

Heterogeneity: $I^2 = 80\%$ [68%; 87%], $\chi^2_{15} = 75.30$ ($p < 0.01$)

Tau²= 0.412

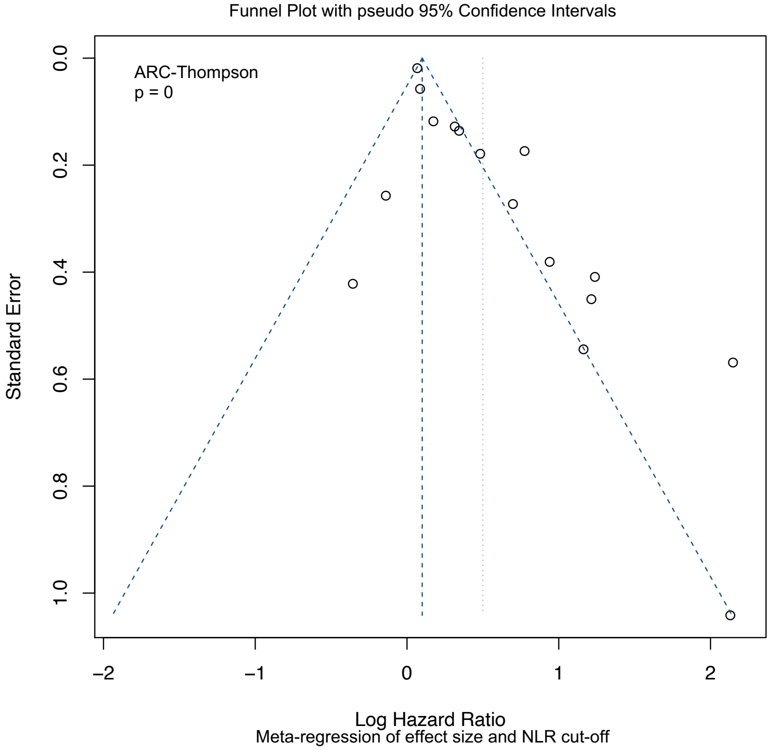

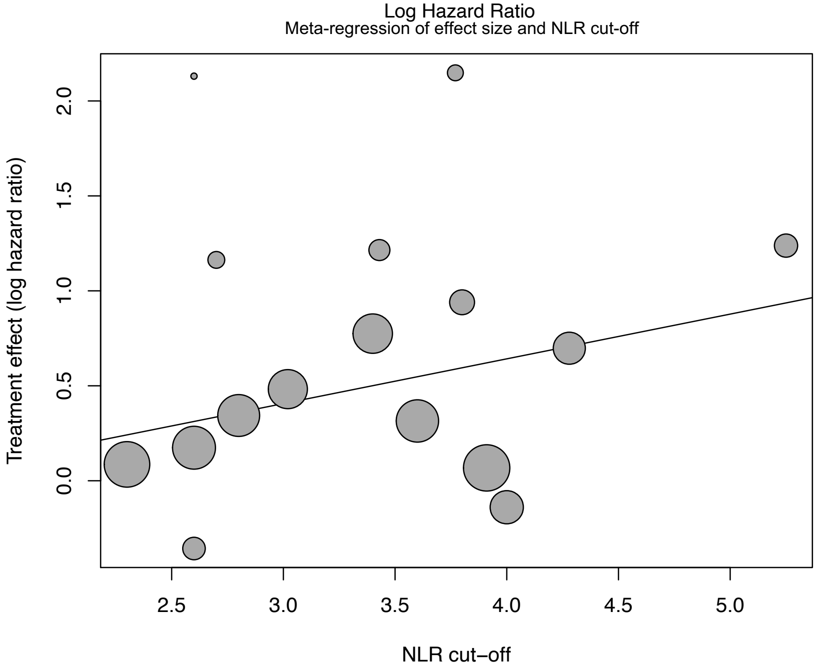

Supplementary Figure 201 - NLR: OS in Hepatocellular (SR)

Study	TE	seTE	HR	95%-CI	Weight (fixed)	Weight (random)
Arai 2017	0.85	0.4027	2.33	[1.06; 5.13]	1.7%	2.9%
Fu 2013	0.36	0.1634	1.43	[1.04; 1.97]	10.5%	11.5%
Goh 2016	0.40	0.2661	1.49	[0.88; 2.51]	4.0%	5.8%
Guo 2009	0.27	0.4652	1.31	[0.53; 3.26]	1.3%	2.2%
Hu 2016	0.52	0.3319	1.68	[0.88; 3.22]	2.5%	4.0%
Ji 2016	0.39	0.1571	1.47	[1.08; 2.00]	11.3%	12.1%
Liao 2014	0.49	0.1545	1.64	[1.21; 2.22]	11.7%	12.3%
Lu 2016	0.26	0.0930	1.30	[1.08; 1.56]	32.4%	19.4%
Mano 2013	1.32	0.6609	3.74	[1.02; 13.66]	0.6%	1.1%
Okamura 2015	0.88	0.2623	2.41	[1.44; 4.03]	4.1%	5.9%
Okamura 2016	0.99	0.2748	2.69	[1.57; 4.61]	3.7%	5.5%
Peng 2014	0.97	0.3389	2.64	[1.36; 5.13]	2.4%	3.9%
Yang 2016	0.29	0.1429	1.33	[1.01; 1.76]	13.7%	13.4%
**Pooled HR by Fixed Effects**			**1.51**	**[1.37; 1.68]**	**100.0%**	**--**
**Pooled HR by Random Effects**			**1.60**	**[1.39; 1.84]**	**--**	**100.0%**
**Prediction interval**				**[1.15; 2.24]**		

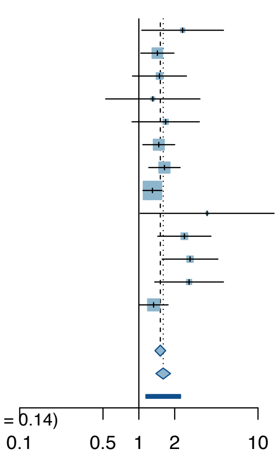

Heterogeneity: $I^2 = 31\%$ [0%; 64%], $\chi^2_{12} = 17.35$ ($p = 0.14$)

Tau²= 0.134

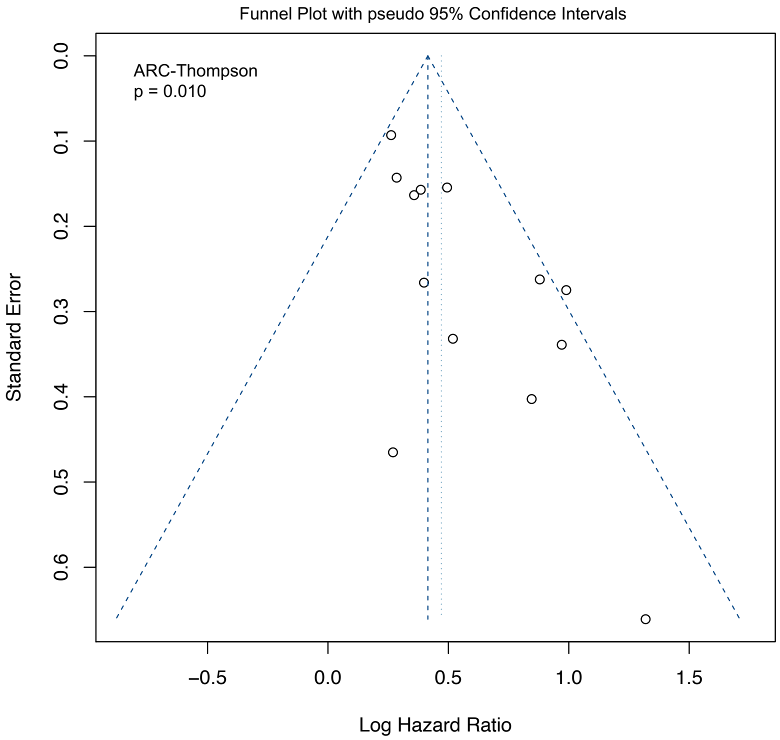

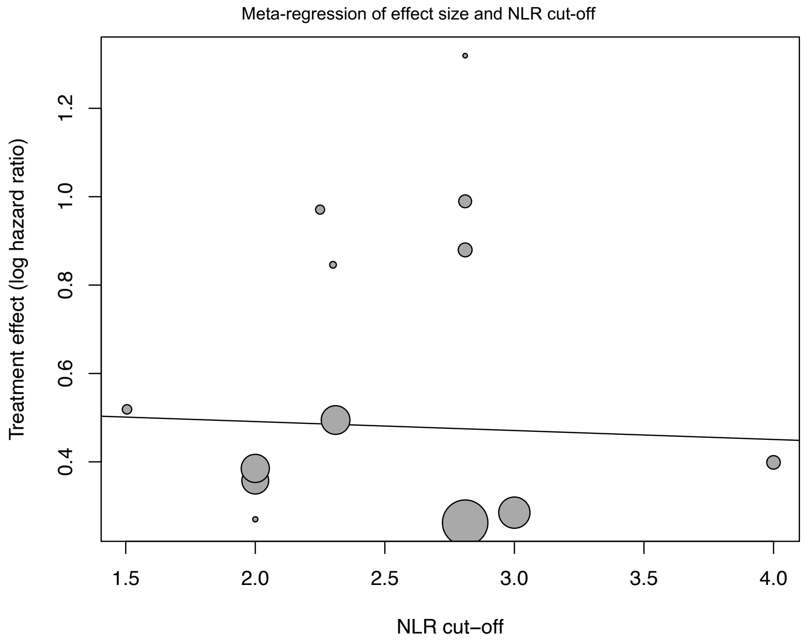

Supplementary Figure 202 - NLR: DFS in Hepatocellular (SR)

Study	TE	seTE	Hazard Ratio	HR	95%–CI	Weight (fixed)	Weight (random)
Fu 2013	0.31	0.1458		1.36	[1.02; 1.81]	14.4%	14.4%
Gomez 2008	0.95	0.4148		2.59	[1.15; 5.84]	1.8%	1.8%
Guo 2009	1.25	0.4043		3.50	[1.58; 7.73]	1.9%	1.9%
Ji 2016	0.34	0.1367		1.40	[1.07; 1.83]	16.4%	16.4%
Liao 2014	0.52	0.1550		1.69	[1.25; 2.29]	12.7%	12.7%
Yang 2016	0.48	0.1282		1.61	[1.25; 2.07]	18.6%	18.6%
Yang 2017	0.32	0.0943		1.38	[1.15; 1.66]	34.4%	34.4%
**Pooled HR by Fixed Effects**				**1.50**	**[1.35; 1.67]**	**100.0%**	**--**
**Pooled HR by Random Effects**				**1.50**	**[1.35; 1.67]**	**--**	**100.0%**
**Prediction interval**					**[1.30; 1.73]**		

Heterogeneity: $I^2 = 30\%$ [0%; 70%], $\chi^2_6 = 8.51$ ($p = 0.20$)

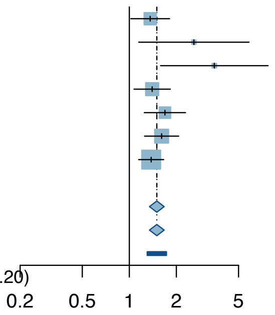

Tau²= 0.001

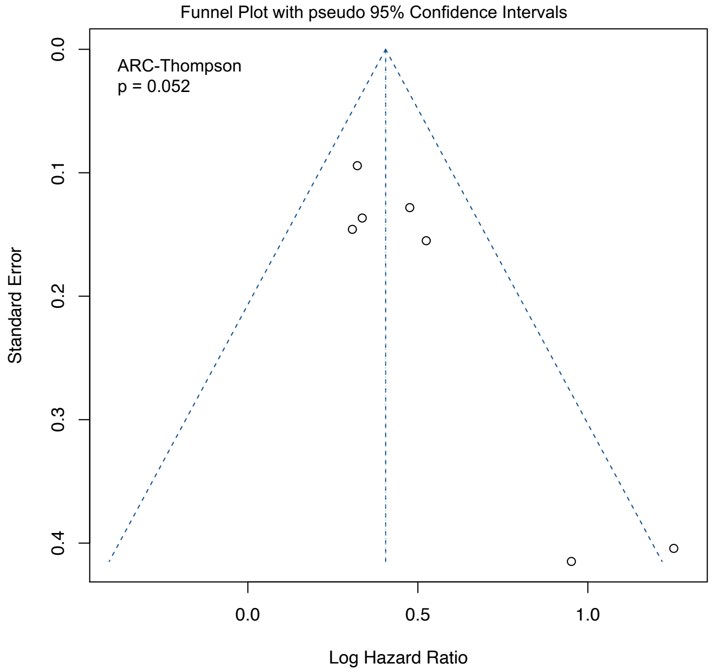

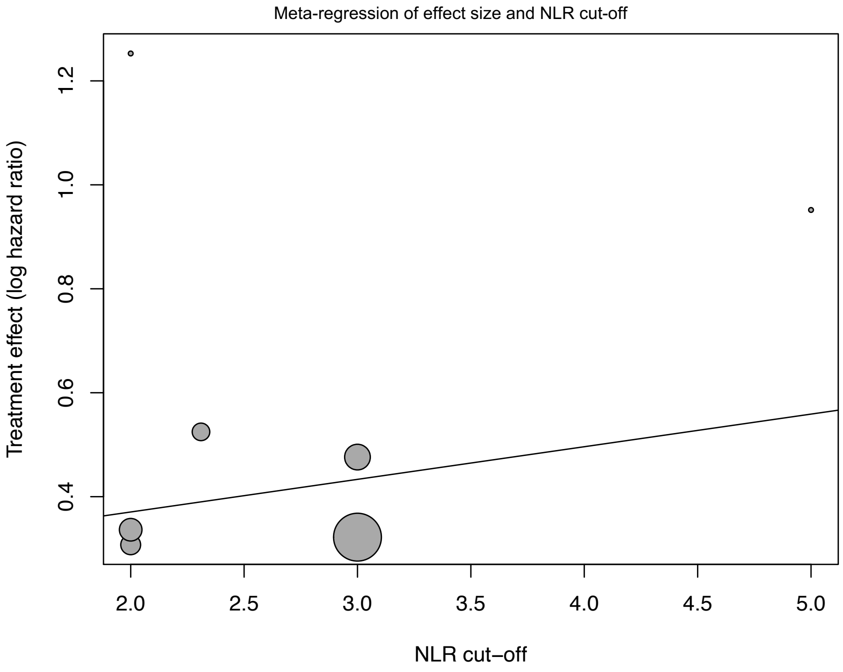

Supplementary Figure 203 - NLR: OS in Prostate

Study	TE	seTE	HR	95%–CI	Weight (fixed)	Weight (random)
Van RJ 1 2015	0.36	0.0882	1.43	[1.20; 1.70]	9.9%	9.1%
Van RJ 2 2015	0.25	0.0770	1.29	[1.11; 1.50]	13.0%	9.6%
Yoshihiro 2015	0.68	0.3162	1.98	[1.07; 3.68]	0.8%	2.7%
Zhang J 2015	1.53	0.4606	4.63	[1.88; 11.42]	0.4%	1.4%
Nuhn P 2014	0.63	0.2105	1.88	[1.24; 2.84]	1.7%	4.7%
Arnoud J 2014	0.64	0.2042	1.89	[1.27; 2.82]	1.8%	4.8%
Lorente 2014	0.44	0.0875	1.55	[1.31; 1.84]	10.1%	9.2%
Bahig 2015	0.10	0.0733	1.10	[0.95; 1.27]	14.3%	9.8%
Akihisa Y 2014	1.00	0.4869	2.73	[1.05; 7.09]	0.3%	1.3%
McLachlan JM 2015	0.77	0.3572	2.15	[1.07; 4.33]	0.6%	2.2%
Linton A 2013	0.17	0.2026	1.19	[0.80; 1.77]	1.9%	4.9%
Sonpavde G 2 2014	0.23	0.1498	1.26	[0.94; 1.69]	3.4%	6.6%
Langsenlehner T 2015	0.77	0.3131	2.16	[1.17; 3.99]	0.8%	2.7%
Sonpavde G 1 2014	0.52	0.0991	1.68	[1.38; 2.04]	7.9%	8.7%
Shfique K 2012	0.45	0.0837	1.57	[1.33; 1.85]	11.0%	9.3%
Sharma 2015	0.11	0.0601	1.12	[1.00; 1.26]	21.3%	10.3%
Pinar A 2015	0.70	0.3143	2.02	[1.09; 3.74]	0.8%	2.7%
**Pooled HR by Fixed Effects**			**1.36**	**[1.28; 1.43]**	**100.0%**	**--**
**Pooled HR by Random Effects**			**1.49**	**[1.33; 1.67]**	**--**	**100.0%**
**Prediction interval**				**[1.01; 2.20]**		

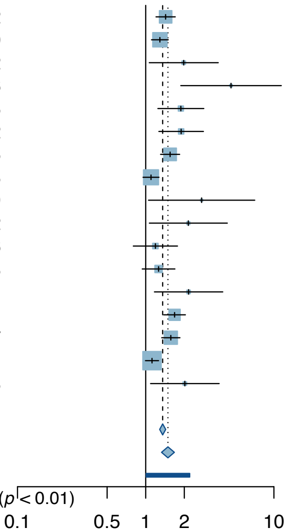

Heterogeneity: I^2 = 69% [48%; 81%], χ^2_{16} = 50.93 (p < 0.01)

Tau²= 0.174

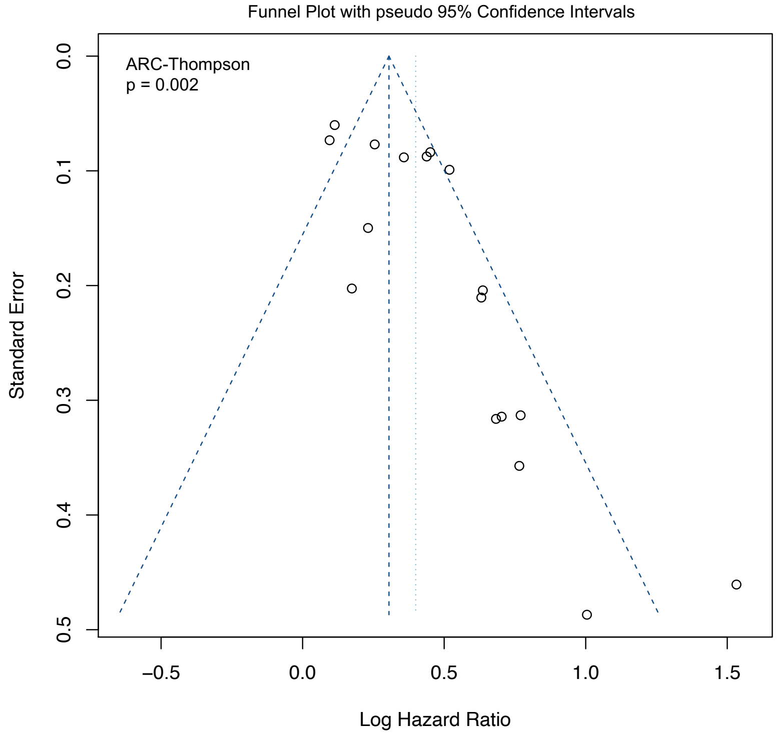

Supplementary Figure 204 - NLR: RFS in Prostate Localised

Study	TE	seTE	HR	95%–CI	Weight (fixed)	Weight (random)
Poyet C 2013	0.06	0.1158	1.06	[0.84; 1.33]	21.6%	17.3%
Young Suk 2015	0.54	0.8305	1.72	[0.34; 8.76]	0.4%	4.8%
Bahig H 2015	0.02	0.0914	1.02	[0.85; 1.22]	34.6%	17.6%
Langenlehner 2015	1.13	0.3230	3.09	[1.64; 5.82]	2.8%	12.8%
Lee H 2015	1.07	0.1344	2.92	[2.24; 3.80]	16.0%	17.0%
Minardi 2015	0.67	0.3057	1.95	[1.07; 3.55]	3.1%	13.2%
Sharma 2015	–0.06	0.1160	0.94	[0.75; 1.18]	21.5%	17.3%
**Pooled HR by Fixed Effects**			**1.26**	**[1.13; 1.40]**	**100.0%**	**––**
**Pooled HR by Random Effects**			**1.56**	**[1.03; 2.36]**	**––**	**100.0%**
**Prediction interval**				**[0.39; 6.20]**		

Heterogeneity: $I^2 = 90\%$ [83%; 95%], $\chi^2_6 = 62.96$ ($p < 0.01$)

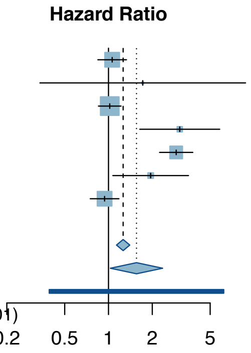

Tau²= 0.494

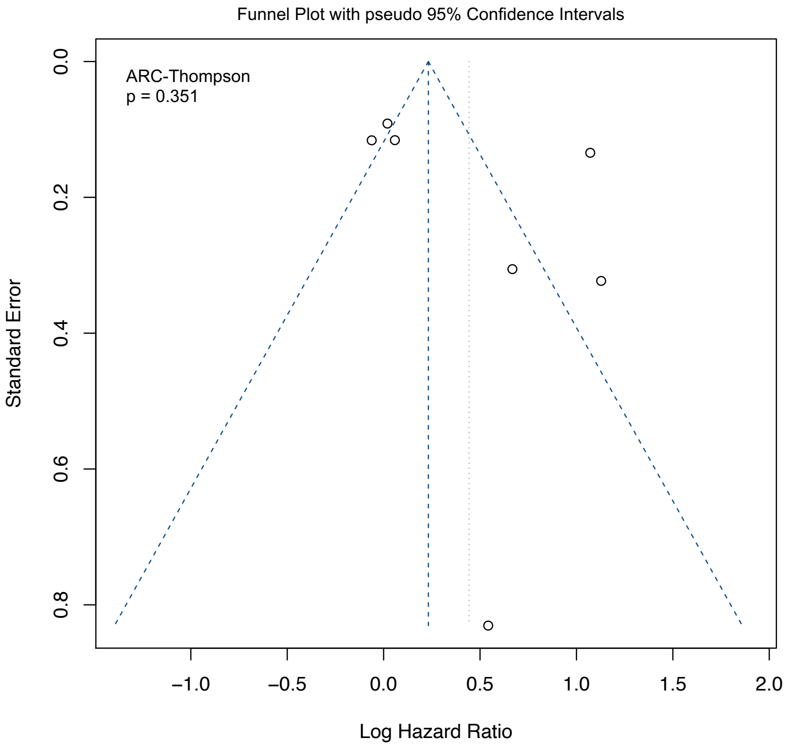

Supplementary Figure 205 - NLR: OS in NSCLC (Immunotherapy)

Study	TE	seTE	HR	95%–CI	Weight (fixed)	Weight (random)
Kiriu T 2019	1.44	0.4646	4.20	[1.69; 10.44]	2.6%	9.6%
Soyano AE 2018	0.63	0.2446	1.87	[1.16; 3.02]	9.2%	15.4%
Naqash AR 2018	1.05	0.3146	2.85	[1.54; 5.28]	5.6%	13.4%
Fukui T 2019	1.43	0.5770	4.17	[1.35; 12.92]	1.7%	7.5%
Facchinetti F 2018	1.17	0.4637	3.22	[1.30; 7.99]	2.6%	9.7%
Diem S 2017	1.61	0.4611	5.01	[2.03; 12.37]	2.6%	9.7%
Bagley SJ 2017	0.73	0.2379	2.07	[1.30; 3.30]	9.8%	15.6%
Mitchell P 2015	0.11	0.0915	1.12	[0.94; 1.34]	66.0%	19.1%
**Pooled HR by Fixed Effects**			**1.48**	**[1.28; 1.72]**	**100.0%**	**--**
**Pooled HR by Random Effects**			**2.43**	**[1.64; 3.59]**	**--**	**100.0%**
**Prediction interval**				**[0.73; 8.08]**		

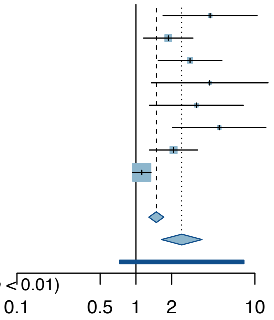

Heterogeneity: $I^2 = 80\%$ [61%; 90%], $\chi^2_7 = 34.59$ ($p < 0.01$)

Tau²= 0.449

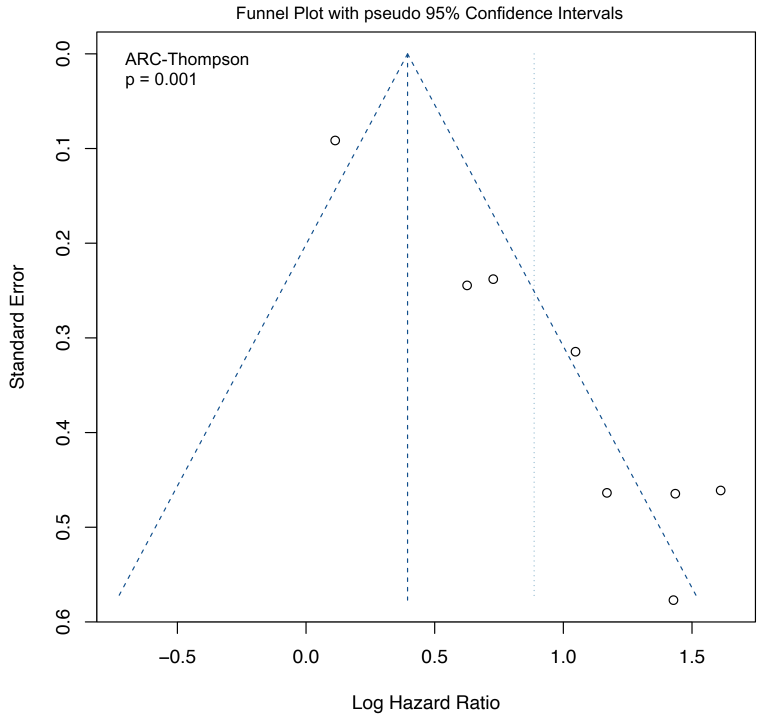

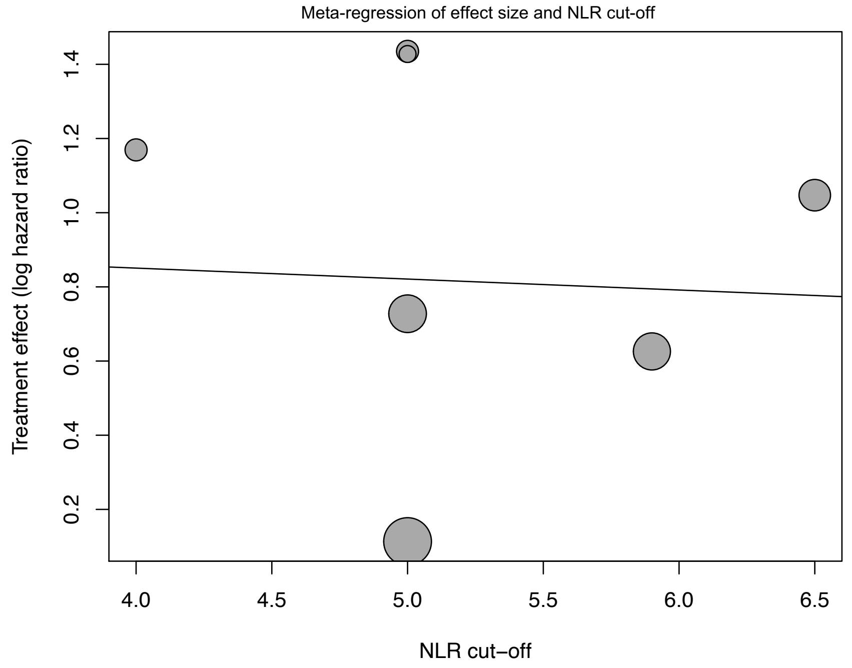

Supplementary references

Kyrgiou, M., Kalliala, I., Markozannes, G., Gunter, M. J., Paraskevaidis, E., Gabra, H., Martin-Hirsch, P., et al. (**2017**). "Adiposity and cancer at major anatomical sites: umbrella review of the literature." **BMJ** 356: j477.

Theodoratou, E., Tzoulaki, I., Zgaga, L. and Ioannidis, J. P. (**2014**). "Vitamin D and multiple health outcomes: umbrella review of systematic reviews and meta-analyses of observational studies and randomised trials." **BMJ** 348: g2035.

Tsilidis, K. K., Kasimis, J. C., Lopez, D. S., Ntzani, E. E. and Ioannidis, J. P. (**2015**). "Type 2 diabetes and cancer: umbrella review of meta-analyses of observational studies." **BMJ** 350: g7607.
