# Supplementary material for: Neutrophil to lymphocyte ratio and cancer prognosis: an umbrella review of systematic reviews and meta-analyses of observational studies
Source: BMC Med. 2020 Nov 20;18:360. doi: 10.1186/s12916-020-01817-1 (PMC7678319; doi:10.1186/s12916-020-01817-1)
Supplement: Supplementary file 3 — Additional file 3. Supplementary Table 1. [file 12916_2020_1817_MOESM3_ESM.pdf]

Supplementary Table 1 *- Description of 204 included meta-analyses*

Author	Year	Biomarker	Cancer site	meta-analysis metric	N	Sample size (HNLR/LNLR)	Fixed Effects			Random Effects			Largest Study			95% Prediction Interval	
							HR	Lower Limit	Upper Limit	HR	Lower Limit	Upper Limit	HR	Lower Limit	Upper Limit	Lower Limit	Upper Limit
Jiang T (1)	2018	NLR	Advanced Cancer with Anti-VEGFR	OS	14	2918	1.45	1.35	1.55	2.02	1.61	2.53	2.29	1.86	2.82	0.88	4.64
Jiang T (1)	2018	NLR	Advanced Cancer (Immunotherapy)	OS	23	4355	1.51	1.41	1.61	1.95	1.67	2.29	2.29	1.86	2.82	1.03	3.71
Jiang T (1)	2018	NLR	Advanced Cancer (Immunotherapy)	PFS	16	2318	1.25	1.19	1.31	1.78	1.47	2.15	1.54	1.17	2.04	0.9	3.54
Mei Z (2)	2017	NLR	All Cancers	OS	65	18262[†]	1.1	1.08	1.12	1.68	1.56	1.82	0.8	0.55	1.16	1.04	2.72
Mei Z (2)	2017	NLR	All Cancers	PFS	23	5607	1.25	1.21	1.3	1.63	1.43	1.86	1.73	1.42	1.88	0.94	2.81
Shen M (3)	2014	IN	All Cancers	OS	16	3335	1.46	1.32	1.6	1.66	1.37	2.02	1.16	0.98	1.38	0.81	3.4
Shen M (3)	2014	IN	All Cancers	CSS	2	437	3.36	2.08	5.41	3.36	2.08	5.41	3.13	1.43	6.83	NA	NA
Paramanathan A (4)	2014	NLR	All Cancers	DFS	20	13065	1.29	1.28	1.31	2.11	1.71	2.6	1.2	1.18	1.22	0.81	5.47
Shen M (3)	2014	PN	All Cancers	OS	2	535	1.26	0.97	1.63	1.66	0.64	4.32	1.06	0.79	1.41	NA	NA
Shen M (3)	2014	SN	All Cancers	OS	2	535	1.07	0.84	1.35	1.1	0.76	1.6	0.93	0.69	1.25	NA	NA
Dolan R (5)	2017	NLR	All Cancers Operable	OS	120	68603 (18667/49936)	1.17	1.16	1.18	1.66	1.55	1.77	1.23	1	1.5	0.97	2.84
Dolan R (5)	2017	NLR	All Cancers Operable	CSS	41	21175 (4617/16558)	1.06	1.05	1.08	1.5	1.35	1.67	1.09	0.94	1.26	0.87	2.58
Tang H (6)	2017	NLR	Biliary Tract	OS	14	3217 (1295/1710)[†]	1.49	1.36	1.63	1.52	1.36	1.7	1.26	1.06	1.5	1.19	1.93
Tang H (6)	2017	NLR	Biliary Tract	RFS	5	637 (303/244)	1.39	1.19	1.62	1.41	1.19	1.68	1.25	0.92	1.7	0.97	2.07
Hu G (7)	2019	NLR	Bladder	OS	18	11945	1.07	1.05	1.09	1.37	1.18	1.6	1.10	1.00	1.20	0.78	2.43
Wu S (8)	2018	NLR	Bladder	RFS	9	8075	1.31	1.23	1.39	1.52	1.29	1.79	1.2	1.1	1.3	0.93	2.48
Wu S (8)	2018	NLR	Bladder (Metastasis)	OS	4	862	1.64	1.38	1.94	1.64	1.38	1.94	1.74	1.32	2.29	1.13	2.38
Hu G (7)	2019	NLR	Bladder (Radical Cystectomy and NAC)	OS	4	447	1.06	1.00	1.12	1.07	0.98	1.16	1.02	0.94	1.10	0.80	1.41
Hu G (7)	2019	NLR	Bladder (Radical Cystectomy)	OS	8	10363	1.06	1.03	1.08	1.13	1.05	1.23	1.10	1.00	1.20	0.92	1.40
Suh J (9)	2019	NLR	Bladder (Urothelial Transurethral Resection)	PFS	4	1040	2.45	1.49	4.02	2.45	1.49	4.02	2.37	1.17	4.78	0.82	7.28
Suh J (9)	2019	NLR	Bladder (Urothelial Transurethral Resection)	RFS	5	1129	2.22	1.81	2.74	2.22	1.81	2.74	2.08	1.60	2.70	1.59	3.12
Li X (10)	2017	NLR	Bladder and Upper Urinary	PFS	19	8182	1.48	1.34	1.63	1.68	1.44	1.95	1.05	0.87	1.26	1.07	2.62
Li X (10)	2017	NLR	Bladder and Upper Urinary	CSS	17	7645	1.34	1.25	1.43	1.62	1.39	1.89	1.07	0.87	1.31	0.99	2.66
Ethier JL (11)	2017	NLR	Breast	OS	13	8015	2.14	1.83	2.5	2.54	1.96	3.3	1.63	1.07	2.48	1.2	3.3
Liu X (12)	2017	NLR	Breast	DFS	12	5523	1.05	1.01	1.1	1.65	1.3	2.1	1.5	1.14	1.97	0.83	3.27
Liu X (12)	2017	NLR	Breast	CSS	4	4186	1.17	1.02	1.34	2.03	1.08	3.82	1.09	0.94	1.26	0.14	29.25
Chen J (13)	2018	NLR	Breast (Metastasis)	OS	2	858	2.81	2.12	3.71	2.81	2.12	3.71	2.7	1.19	6.16	NA	NA

Chen J (13)	2018	NLR	Breast (Metastasis)	DFS	3	1003	2.15	1.53	3.01	2.15	1.53	3.01	2.2	1.29	3.75	0.24	19.27
Chen J (13)	2018	NLR	Breast (No Metastasis)	OS	2	488	2.28	1.73	2.99	1.46	0.37	5.7	0.7	0.4	1.4	NA	NA
Chen J (13)	2018	NLR	Breast (No Metastasis)	DFS	2	466	0.87	0.56	1.35	0.87	0.56	1.35	0.8	0.5	1.3	NA	NA
Duan J (14)	2018	NLR	Breast (SR)	DFS	15	7820	1.10	1.06	1.15	1.58	1.27	1.96	2.58	1.23	5.42	0.76	3.29
Duan J (14)	2018	NLR	Breast (SR)	OS	12	6233	2.32	1.91	2.82	2.47	1.71	3.56	3.05	1.08	8.61	0.84	7.25
Duan J (14)	2018	NLR	Breast (SR)	RFS	2	319	4.05	1.94	8.46	4.05	1.94	8.46	4.60	1.09	19.10		
Guo W (15)	2019	NLR	Breast (Triple Negative and Her2 Positive)	DFS	27	11369	1.52	1.41	1.64	1.59	1.41	1.8	1.50	1.14	1.97	1.04	2.45
Guo W (15)	2019	NLR	Breast (Triple Negative and Her2 Positive)	OS	28	14128	1.50	1.38	1.62	1.78	1.49	2.12	1.03	0.57	1.86	0.84	3.76
Liu X (12)	2017	NLR	Breast (Triple-negative)	OS	4	608	2.58	1.63	4.06	2.58	1.63	4.06	3.05	1.08	8.61	0.95	7.01
Liu X (12)	2017	NLR	Breast (Triple-negative)	DFS	4	461	3.51	1.97	6.25	3.51	1.97	6.25	2.58	1.23	5.42	0.99	12.47
Wu J (16)	2017	NLR	Cervical	OS	12	3661	1.21	1.16	1.26	1.46	1.21	1.76	1.19	1.13	1.25	0.83	2.57
Wu J (16)	2017	NLR	Cervical	PFS	10	2452	1.19	1.14	1.24	1.71	1.32	2.22	1.13	1.08	1.18	0.7	4.18
Zhang J (17)	2017	NLR	Colorectal	DFS	5	2178	1.66	1.31	2.12	1.76	1.27	2.42	1.24	0.86	1.79	0.74	4.17
Zheng DC (18)	2016	NLR	Colorectal	OS	21	7927	1.59	1.45	1.74	1.92	1.58	2.32	1.31	1.09	1.57	0.95	3.88
Zheng DC (18)	2016	NLR	Colorectal	PFS	14	3662	1.71	1.48	1.97	1.91	1.48	2.46	1.95	0.71	5.37	0.86	4.23
Malietzis G (19)	2014	NLR	Colorectal (PC)	DFS	3	515 (148/367)	1.53	1.23	1.9	1.53	1.23	1.9	1.51	1.1	2.06	0.38	6.2
Li H (20)	2019	NLR	Colorectal (SR)	DFS	9	4237	1.42	1.20	1.69	1.54	1.18	2	1.24	0.86	1.79	0.74	3.18
Li H (20)	2019	NLR	Colorectal (SR)	OS	11	4832	1.58	1.39	1.79	1.67	1.37	2.03	1.29	1.07	1.80	0.93	2.99
Li H (20)	2019	NLR	Colorectal (SR)	RFS	3	643	2.31	1.69	3.17	2.31	1.69	3.17	1.81	1.07	3.07	0.30	17.85
Tang H (21)	2016	NLR	CLM	OS	7	1491	2.17	1.83	2.57	2.17	1.83	2.57	2.28	1.65	3.13	1.73	2.72
Tang H (21)	2016	NLR	CLM	RFS	6	1093	1.96	1.63	2.35	1.97	1.48	2.62	2.21	1.7	2.88	0.91	4.23
Tang H (21)	2016	NLR	CLM (Non-surgical)	OS	2	182	3.11	1.83	5.29	3.11	1.83	5.29	2.9	1.51	5.55	NA	NA
Tang H (21)	2016	NLR	CLM (Non-surgical)	RFS	2	190	2.33	1.37	3.95	2.32	1.23	4.38	1.67	0.78	3.57	NA	NA
Tang H (21)	2016	NLR	CLM (SR)	OS	5	1309	2.08	1.73	2.49	2.08	1.73	2.49	2.28	1.65	3.13	1.55	2.79
Tang H (21)	2016	NLR	CLM (SR)	RFS	4	903	1.91	1.58	2.32	1.89	1.34	2.66	2.21	1.7	2.88	0.49	7.25
Ni L (22)	2020	NLR	Endometrial carcinoma	OS	9	2942	2.22	1.76	2.79	2.22	1.76	2.79	1.82	1.27	2.62	1.68	2.92
Ni L (22)	2020	NLR	Endometrial carcinoma	PFS	5	1798	1.80	1.35	2.40	1.84	1.31	2.57	1.65	1.02	2.65	0.82	4.12
Sun J (23)	2016	NLR	Gastric	OS	19	5431	1.98	1.87	2.1	1.98	1.75	2.23	1.52	1.33	1.75	1.39	2.82
Sun J (23)	2016	NLR	Gastric	DFS	7	976	1.58	1.33	1.88	1.58	1.33	1.88	1.65	1.09	2.49	1.26	1.99
Shen M (3)	2014	IN	Gastric	OS	2	388	1.01	0.74	1.39	1.2	0.5	2.89	1.94	1.08	3.5	NA	NA
Zhang X (24)	2014	NLR	Gastric	PFS	2	314	1.54	1.22	1.95	1.59	1.15	2.19	2.48	1.15	1.89	NA	NA
Szor D (25)	2018	NLR	Gastric (SR)	OS	7	3223 (1018/2205)	3.05	2.5	3.66	3.13	1.99	4.92	10.01	6.47	15.48	0.69	14.24
Mellor K (26)	2018	NLR	Gastric (SR)	DFS	3	820 (156/664)	3.14	2.14	4.6	2.73	1.18	6.34	6.03	3.45	10.53	0	58896.33
Zhou Y (27)	2018	NLR	GNT	OS	4	422	3.06	1.97	4.76	3.06	1.97	4.76	3.6	1.33	9.71	1.16	8.08
Zhou Y (27)	2018	NLR	GNT	*RFS*	3	300	3.26	2.01	5.28	3.26	2.01	5.29	2.75	1.57	4.81	0.14	76.19
Luo XF (28)	2018	NLR	Gastrointestinal stromal	OS	2	278	1.13	1.05	1.22	1.74	0.63	4.84	1.12	1.04	1.21	NA	NA

Lei Y (29)	2019	NLR	Glioma	OS	16	2275	1.03	1.01	1.06	1.52	1.28	1.81	1.00	0.70	1.43	0.81	2.88
Ethier JL (30)	2017	NLR	Gynecologic	OS	27	10530	1.24	1.19	1.29	1.72	1.46	2.03	1.3	1.05	1.62	0.89	3.35
Yu Y (31)	2018	NLR	Head and Neck	RFS	4	658	1.34	1.13	1.59	1.63	1.1	2.41	1.81	1.28	2.56	0.34	7.87
Tham T (32)	2018	NLR	Head and Neck	OS	26	6769	1.63	1.48	1.81	1.72	1.51	1.96	1.57	1.04	2.39	1.18	2.52
Tham T (32)	2018	NLR	Head and neck	PFS	6	2173	1.44	1.23	1.69	1.5	1.18	1.93	1.68	1.19	2.38	0.74	3.05
Tham T (32)	2018	NLR	Head and neck	DFS	11	1692	1.51	1.27	1.79	1.66	1.3	2.11	0.98	0.73	1.33	0.91	3.03
Shen M (3)	2014	IN	Head and Neck	OS	3	220	1.69	1.1	2.61	1.69	1.1	2.61	1.98	0.3	4.18	0.1	28.67
Tham T (32)	2018	NLR	Head and Neck (No Surgery)	OS	9	3285	1.44	1.21	1.71	1.48	1.21	1.82	1.57	1.04	2.39	0.97	2.26
Yang L (33)	2019	NLR	Head and Neck (SCC)	CSS	5	1854	1.46	1.24	1.73	1.46	1.24	1.73	1.38	1.07	1.77	1.12	1.91
Yang L (33)	2019	NLR	Head and Neck (SCC)	PFS	4	461	1.40	1.15	1.71	1.53	1.11	2.11	2.20	1.13	4.29	0.46	5.13
Cho J (34)	2018	NLR	Head and Neck (SCC)	OS	24	5650	1.77	1.59	1.96	1.96	1.66	2.31	1.71	0.14	20.30	1.11	3.45
Cho J (34)	2018	NLR	Head and Neck (SCC)	DFS	12	2079	1.43	1.26	1.63	1.9	1.41	2.54	0.98	0.73	1.32	0.75	4.79
Tham T (32)	2018	NLR	Head and Neck (SR)	OS	3	707	1.54	1.23	1.93	1.76	1.17	2.65	1.31	1.00	1.71	0.02	144.88
Min GT (35)	2018	NLR	Hepatocellular	OS	44	10491 (2594/5312)[†]	1.22	1.18	1.25	1.64	1.41	1.9	1.3	1.07	1.56	0.6	3.95
Min GT (35)	2018	NLR	Hepatocellular	DFS	17	3881 (919/1729)[†]	1.23	1.15	1.32	1.82	1.4	2.37	1.38	1.15	1.66	0.67	4.92
Min GT (35)	2018	NLR	Hepatocellular	RFS	16	5037 (1470/3188)[†]	1.13	1.11	1.15	1.85	1.48	2.32	1.32	1.06	1.64	0.8	4.29
Xiao WK (36)	2014	NLR	Hepatocellular (MT)	OS	3	522 (213/309)	1.85	1.4	2.44	1.87	1.39	2.53	1.6	1.12	2.28	0.2	17.22
Xiao WK (36)	2014	NLR	Hepatocellular (RFA)	OS	2	370 (172/198)	1.27	1.14	1.42	1.27	1.12	1.45	1,25	1.11	1.4	NA	NA
Xiao WK (36)	2014	NLR	Hepatocellular (RFA)	DFS	2	1054 (264/790)	1.07	0.82	1.39	1.07	0.82	1.39	1.27	0.83	1.96	NA	NA
Liu L (37)	2019	NLR	Hepatocellular (Sorfenib)	OS	11	2324	1.38	1.28	1.49	1.73	1.45	2.07	2.13	1.46	3.12	1.03	2.91
Wang Y (38)	2018	NLR	Hepatocellular (SR)	RFS	10	3404 (940/2298)[†]	1.64	1.44	1.86	1.71	1.45	2.03	1.32	1.06	1.64	1.16	2.53
Wang Y (38)	2018	NLR	Hepatocellular (SR)	OS	13	4225 (1336/2723)*	1.51	1.37	1.68	1.6	1.39	1.84	1.30	1.07	1.56	1.15	2.24
Wang Y (38)	2018	NLR	Hepatocellular (SR)	DFS	7	1272 (581/691)	1.50	1.35	1.67	1.5	1.35	1.67	1.40	1.08	1.83	1.30	1.73
Li S (39)	2020	NLR	Hepatocellular (TACE)	OS	16	4023	1.81	1.67	1.97	1.85	1.64	2.08	1.38	1.07	1.78	1.33	2.57
Xu ZG (40)	2018	NLR	Hepatocellular (Transplant)	OS	8	1230 (352/878)	1.53	1.28	1.82	2.19	1.4	3.43	1.42	0.88	2.3	0.49	9.71
Xu ZG (40)	2018	NLR	Hepatocellular (Transplant)	RFS	4	83 (251/372)[†]	2.91	2.17	3.91	3.74	2.05	6.83	2.22	1.55	3.18	0.32	43.6
Shen M (3)	2014	IN	Hepatocellular and ICC	OS	4	846	1.79	1.48	2.17	1.8	1.33	2.43	2	1.46	2.75	0.54	5.99
Yang L (33)	2019	NLR	Hypopharynx	OS	3	310	2.88	2.06	4.02	2.88	2.06	4.02	2.99	1.91	4.67	0.33	25.01
Tan Q (41)	2018	NLR	Immunotherapy (Immune checkpoint inhibitors)	OS	18	1863	1.07	1.02	1.12	2.21	1.7	2.88	1.95	1.11	3.43	0.82	5.97
Tan Q (41)	2018	NLR	Immunotherapy (Immune checkpoint inhibitors)	PFS	11	1274	1.06	1.01	1.11	1.75	1.39	2.21	1.46	1.06	2	0.84	3.66

Xie X (42)	2019	NLR	Ipilimumuab	OS	7	798	1.13	1.08	1.18	2.53	1.58	4.05	2.03	1.49	2.77	0.49	13.13
Xie X (42)	2019	NLR	Ipilimumuab	PFS	3	370	2.11	1.77	2.52	2.02	1.29	3.18	1.01	1.33	2.46	0.01	330.06
Mu S (43)	2018	NLR	Large B	OS	10	†	1.62	1.35	1.95	1.86	1.2	2.88	1.14	0.8	1.63	0.44	7.85
Mu S (43)	2018	NLR	Large B	PFS	9	†	1.45	1.21	1.74	1.6	1.1	2.33	1.54	1.06	2.24	0.48	5.36
Yang L (33)	2019	NLR	Larynx	DFS	3	1260	1.36	1.14	1.63	1.36	1.13	1.63	1.27	1.02	1.57	0.40	4.61
Mascarella M (44)	2018	NLR	Larynx	OS	6	1017	1.52	1.26	1.85	1.57	1.25	1.98	1.31	1	1.72	1.01	2.46
Zhao QT (45)	2015	NLR	Lung	OS	21	6942	1.21	1.16	1.25	1.55	1.33	1.8	1.53	1.46	1.78	0.83	2.89
Zhao QT (45)	2015	NLR	Lung	PFS	9	1748	1.24	1.17	1.31	1.38	1.02	1.87	1.48	1.09	2.02	0.49	3.87
Yu Y (46)	2017	NLR	Lung (Surgery)	OS	7	2877	1.2	1.14	1.27	1.49	1.24	1.79	1.53	1.46	1.78	0.85	2.61
Zhan H (47)	2018	NLR	Melanoma	OS	10	4437	1.1	1.06	1.14	1.6	1.26	2.02	1.25	1.02	1.53	0.72	3.54
Ding Y (48)	2018	NLR	Melanoma	PFS	6	752	2.18	1.78	2.68	2.22	1.77	2.78	1.83	0.81	4.13	1.48	3.33
Xie X (42)	2019	NLR	Melanoma (Immune checkpoint inhibitors)	OS	9	1104	1.14	1.09	1.20	2.49	1.72	3.61	1.95	1.11	3.43	0.67	9.19
Xie X (42)	2019	NLR	Melanoma (Immune checkpoint inhibitors)	PFS	4	467	2.07	1.78	2.41	2.1	1.71	2.59	1.81	1.33	2.46	1.02	4.33
Chen N (49)	2017	NLR	MPM	OS	11	1378	1.26	1.17	1.37	1.48	1.14	1.93	1.01	0.75	1.36	0.63	3.5
Zeng Q (50)	2018	NLR	Multiple Myeloma	OS	8	1886	1.11	1.07	1.15	1.7	1.28	2.27	2.12	1.44	3.11	0.75	3.86
Zeng Q (50)	2018	NLR	Multiple Myeloma	PFS	4	1334	1.09	1.04	1.14	1.78	1.07	2.96	2.47	1.44	4.24	0.17	18.19
Yang S (51)	2019	NLR	Nasopharyngeal	OS	10	7031	1.46	1.30	1.63	1.48	1.29	1.69	1.35	1.13	1.62	1.15	1.90
Yin J (52)	2017	NLR	Nasopharyngeal	PFS	5	4130	1.48	1.3	1.69	1.5	1.3	1.73	1.35	1.13	1.62	1.12	1.99
Su L (53)	2017	NLR	Nasopharyngeal	CSS	3	2320	1.42	1.21	1.67	1.44	1.19	1.74	1.35	1.13	1.62	0.33	6.27
Li X (54)	2018	NLR	NAC	OS	18	3282	1.17	1.11	1.23	1.66	1.36	2.04	2.1	1.6	2.76	0.8	3.48
Li X (54)	2018	NLR	NAC	RFS	7	775	1.9	1.5	2.41	2.01	1.47	2.76	3.87	1.64	9.15	0.94	4.33
Li X (54)	2018	NLR	NAC	CSS	5	971	1.44	1.28	1.62	2.17	1.35	3.47	2.5	1.8	3.47	0.48	9.81
Xie X (42)	2019	NLR	Nivolumab	OS	6	442	2.55	1.89	3.46	2.7	1.81	4.04	2.07	1.30	3.30	1.01	7.21
Xie X (42)	2019	NLR	Nivolumab	PFS	8	744	1.09	1.04	1.15	1.5	1.2	1.88	1.48	1.08	2.01	0.77	2.91
Vartolomei MD (55)	2018	NLR	Non muscle Invasive Bladder	RFS	6	2298	1.57	1.36	1.8	1.76	1.33	2.32	1.27	1.05	1.53	0/81	3.83
Vartolomei MD (55)	2018	NLR	Non muscle Invasive Bladder	PFS	6	2298	2.13	1.59	2.87	2.26	1.59	3.22	1.72	1.16	2.54	1.13	4.52
Vartolomei MD (55)	2018	NLR	Non muscle Invasive Bladder (High Risk)	RFS	4	599	1.82	1.34	2.47	2.31	1.27	4.18	1.46	1.01	2.11	0.22	23.88
Vartolomei MD (55)	2018	NLR	Non muscle Invasive Bladder (High Risk)	PFS	3	510	2.54	1.37	4.7	2.54	1.37	4.7	2.8	1.13	6.95	0.05	138.97
Lu Y (56)	2020	NLR	NSCLC	OS	21	5141	1.30	1.24	1.38	1.41	1.26	1.59	0.91	0.72	1.14	0.90	2.22
Gu XB (57)	2015	NLR	NSCLC	PFS	10	2623	1.1	1.04	1.15	1.59	1.29	1.95	1.37	1.05	1.78	0.85	2.97
Shen M (3)	2014	IN	NSCLC	OS	2	967	1.16	1	1.35	1.16	1	1.35	1.16	0.98	1.38	NA	NA
Wang Z (58)	2019	NLR	NSCLC (Chemotherapy)	OS	9	1276	1.13	1.07	1.20	1.74	1.24	2.42	1.68	1.30	2.18	0.57	5.31
Wang Z (58)	2019	NLR	NSCLC (Chemotherapy)	PFS	5	875	1.69	1.41	2.03	1.75	1.38	2.2	1.29	0.95	1.75	0.95	3.20
Xie X (42)	2019	NLR	NSCLC (Immune Checkpoint Inhibitors)	PFS	6	609	1.08	1.03	1.14	1.38	1.1	1.72	1.46	1.06	2.01	0.71	2.66
Wang Z (58)	2019	NLR	NSCLC (Immunotherapy)	OS	8	1396	1.48	1.28	1.72	2.43	1.64	3.59	1.12	0.94	1.34	0.73	8.08
Cao D (59)	2018	NLR	NSCLC (Nivoumab)	OS	8	666	1.15	1.10	1.20	1.72	1.33	2.23	2.07	1.30	3.30	0.80	3.74

Cao D (59)	2018	NLR	NSCLC (Nivoumab)	PFS	10	900	1.18	1.12	1.24	1.51	1.15	2	1.46	1.06	2.01	0.57	4.04
Jiang T (60)	2019	NLR	NSCLC (PD-1 inhibitor)	OS	13	2018	2.39	2.03	2.81	2.59	2.1	3.2	1.70	1.27	2.28	1.57	4.28
Jiang T (60)	2019	NLR	NSCLC (PD-1 inhibitor)	PFS	12	1614	1.63	1.42	1.87	1.63	1.42	1.87	1.83	1.12	2.98	1.39	1.91
Wang Z (58)	2019	NLR	NSCLC (ST)	OS	24	3694	1.10	1.07	1.14	1.87	1.53	2.29	1.12	0.94	1.34	0.77	4.52
Wang Z (58)	2019	NLR	NSCLC (ST)	PFS	16	2679	1.09	1.04	1.13	1.5	1.29	1.74	1.36	1.04	1.76	0.89	2.51
Wang Z (58)	2019	NLR	NSCLC (TT)	OS	5	422	1.09	1.03	1.16	1.86	1.18	2.91	1.07	1.01	1.14	0.43	8.08
Wang Z (58)	2019	NLR	NSCLC (TT)	PFS	5	619	1.06	0.99	1.13	1.51	1.06	2.13	1.24	0.69	2.21	0.48	4.70
Zhang X (61)	2018	NLR	Oesophageal	OS	26	8985	1.36	1.30	1.44	1.5	1.32	1.71	1.89	1.57	2.28	0.85	2.65
Zhang X (61)	2018	NLR	Oesophageal	CSS	7	1885	1.45	1.25	1.68	1.42	1.12	1.8	1.59	1.13	2.23	0.72	2.81
Zhang X (61)	2018	NLR	Oesophageal	RFS	3	677	1.62	1.26	2.08	1.61	1.16	2.25	1.52	1.09	2.13	0.07	37.24
Zhang X (61)	2018	NLR	Oesophageal	DFS	10	2837	1.24	1.14	1.34	1.59	1.21	2.1	1.25	0.90	1.71	0.61	4.17
Zhang X (61)	2018	NLR	Oesophageal	PFS	4	908	1.43	1.18	1.73	1.43	1.16	1.75	1.53	1.31	2.03	0.85	2.39
Pirozzolo G (62)	2019	NLR	Oesophageal (DCRT)	OS	3	1563	1.74	1.55	1.95	1.74	1.55	1.95	1.67	1.45	1.92	0.83	3.66
Pirozzolo G (62)	2019	NLR	Oesophageal (Neo+Surgery)	OS	3	1314	1.26	1.09	1.46	1.26	1.09	1.46	1.26	1.01	1.57	0.50	3.21
Zhang X (61)	2018	NLR	Oesophageal (No Surgery)	OS	4	1046	1.70	1.45	2.01	1.72	1.28	2.31	1.86	1.50	2.30	0.54	5.42
Zhang X (61)	2018	NLR	Oesophageal (Surgery)	OS	22	7119	1.33	1.26	1.41	1.48	1.27	1.73	1.89	1.57	2.28	0.79	2.78
Pirozzolo G (62)	2019	NLR	Oesophageal (Surgery+/-Chemo)	OS	14	3580	1.69	1.51	1.88	1.77	1.43	2.2	1.34	1.02	1.77	0.83	3.78
Zhang X (61)	2018	NLR	Oesophageal SCC	OS	18	7403	1.39	1.30	1.49	1.39	1.22	1.58	1.89	1.57	2.28	0.86	2.24
Yang Y (63)	2018	NLR	Oral cavity	OS	9	2373	1.73	1.52	1.98	1.71	1.42	2.06	1.76	1.32	2.35	1.07	2.72
Yang Y (63)	2018	NLR	Oral cavity	DFS	10	1920	1.72	1.44	2.07	1.72	1.44	2.07	1.74	1.23	2.46	1.39	2.13
Wang Y (64)	2018	NLR	Oral SCC	OS	6	1360	1.56	1.28	1.9	1.56	1.27	1.91	1.39	1.04	1.86	1.17	2.09
Wang Y (64)	2018	NLR	Oral SCC	DFS	6	1161	1.94	1.47	12.55	1.94	1.47	12.55	1.74	1.23	2.46	1.31	2.86
Yang L (33)	2019	NLR	Oropharynx	DFS	3	x	1.68	1.23	2.30	1.68	1.23	2.3	1.72	1.04	2.85	0.22	12.83
Zhou Q (65)	2017	NLR	Ovarian	PFS	12	3884	1.08	1.05	1.11	1.58	1.26	1.98	1.25	1.05	1.48	0.72	3.43
Zhou Q (65)	2017	NLR	Ovarian	OS	16	4910	1.11	1.07	1.14	1.65	1.29	2.11	1.19	0.94	1.5	0.65	4.15
Zhou Y (66)	2018	NLR	Pancreatic	OS	43	NA†	1.35	1.3	1.41	1.81	1.59	2.05	1.24	1.01	1.52	0.89	3.68
Zhou Y (66)	2018	NLR	Pancreatic	DFS	8	1236	1.58	1.31	1.91	1.66	1.16	2.38	0.87	0.57	1.35	0.54	5.07
Yang JJ (67)	2015	NLR	Pancreatic	CSS	3	845 (387/458)	1.43	1.21	1.69	1.66	1.09	2.53	1.24	1.01	1.51	0.01	224.5
Mowbray N (68)	2018	NLR	Pancreatic (SR)	OS	8	1519	1.72	1.48	2	1.72	1.48	2	1.66	1.12	2.46	1.42	2.08
Zhou Y (69)	2018	NLR	Pancreatic Neuroendocrine Tumour	OS	2	223	4.21	1.95	9.13	4.21	1.95	9.13	3.6	133	9.71	NA	NA
Zhou Y (69)	2018	NLR	Pancreatic Neuroendocrine Tumour	RFS	2	223	5.37	2.14	13.46	5.37	2.14	13.46	4.52	1.05	19.46	NA	NA
Guo J (70)	2018	NLR	Prostate	PFS	7	1088	1.95	1.53	2.48	1.97	1.49	2.62	3.09	1.64	5.82	1.06	3.69
Cao J (71)	2016	NLR	Prostate	OS	16	15298	1.36	1.28	1.43	1.49	1.33	1.67	1.12	1.00	1.26	1.01	2.20
Guo J (70)	2018	NLR	Prostate (Metastatic)	OS	8	2152	1.53	1.35	1.74	1.67	1.38	2.02	1.27	1.05	1.54	1.04	2.69
Cao J (71)	2016	NLR	Prostate CR	PFS	6	1629	1.49	1.31	1.69	1.71	1.29	2.26	1.42	1.15	1.76	0.78	3.74
Yin X (72)	2016	NLR	Prostate CR	OS	9	2765	1.56	1.42	1.72	1.56	1.42	1.72	1.55	1.32	1.83	1.39	1.75
Yin X (72)	2016	NLR	Prostate Localised	OS	2	8765	1.15	1.02	1.29	1.45	0.77	2.71	1.12	1	1.26	NA	NA
Guan Y (73)	2020	NLR	Prostate CR (Abitaterone)	OS	10	1626	1.63	1.43	1.86	1.75	1.44	2.13	1.21	0.96	1.54	1.03	2.96
Guan Y (73)	2020	NLR	Prostate CR (Abitaterone)	PFS	2	198	1.74	1.22	2.49	1.62	0.81	3.26	2.25	1.44	3.51	NA	NA

Guan Y (73)	2020	NLR	Prostate CR (Enzalutamide)	OS	5	1518	1.39	1.29	1.51	1.48	1.26	1.76	1.45	1.22	1.75	0.86	2.56
Guan Y (73)	2020	NLR	Prostate CR (Enzalutamide)	PFS	2	427	1.37	1.22	1.55	1.55	0.98	2.44	1.98	1.54	2.56	NA	NA
Cao J (71)	2016	NLR	Prostate Localised	RFS	7	11745	1.26	1.13	1.40	1.56	1.03	2.36	1.02	0.85	1.22	0.3913 04919	6.2017552 01
Dong YW (74)	2016	NLR	Rectal	OS	6	857	14	3.65	53.75	14	3.65	53.75	29.4	3.7	6317	2.08	94.14
Dong YW (74)	2016	NLR	Rectal	DFS	3	403	4.27	1.93	9.45	4.27	1.93	9.45	3.91	2.32	9.15	0.02	731.37
Dong YW (74)	2016	NLR	Rectal	RFS	3	433	3.6	1.48	8.78	3.6	1.48	8.78	2.92	1.7	8.74	0.01	1167.53
Na N (75)	2016	NLR	Renal	PFS	8	1115†	1.21	1.13	1.29	2.07	1.47	2.92	1.54	1.17	2.04	0.71	6
Luo Y (76)	2015	NLR	Renal	OS	14	3373	1.79	1.61	2	1.92	1.63	2.27	1.78	1.45	2.19	1.23	3.01
Luo Y (76)	2015	NLR	Renal	RFS	3	700	1.97	1.37	2.84	1.98	1.36	2.89	3.07	1.37	6.88	0.14	28.06
Luo Y (76)	2015	NLR	Renal	CSS	4	1728	1.2	1.07	1.35	1.38	0.98	1.95	2.06	1.57	2.7	0.3	6.36
Shen M (3)	2014	IN	Renal	OS	2	206	2.71	1.9	3.86	2.71	1.9	3.86	3.1	1.9	5	NA	NA
Semeniuk-Wojtas A (77)	2018	NLR	Renal (TKI)	OS	7	NA†	2.04	1.72	2.43	2.14	1.66	2.76	1.39	1.02	1.89	1.11	4.12
Semeniuk-Wojtas A (77)	2018	NLR	Renal (TKI)	PFS	6	NA†	1.19	1.11	1.27	1.98	1.31	3.01	1.11	1.04	1.19	0.51	7.69
Boissier R (78)	2017	NLR	Renal Advanced	OS	7	1304 (331/337)†	1.55	1.36	1.76	1.91	1.44	2.53	1.91	1.35	2.69	0.81	4.47
Boissier R (78)	2017	NLR	Renal Advanced	PFS	3	306 (143/163)	3.19	2.23	4.57	3.19	2.23	4.57	3.5	2.03	6.03	0.31	32.69
Boissier R (78)	2017	NLR	Renal Localised	OS	3	1168 (403/651)†	1.02	1.01	1.03	1.46	0.85	2.5	1.02	1.01	1.03	0	683.18
Boissier R (78)	2017	NLR	Renal Localised	RFS	6	1962 (487/817)†	1.01	1	1.02	1.67	1.13	2.45	1.01	1	1.02	0.48	5.82
Li LQ (79)	2020	NLR	STS	DFS	7	1239	1.72	1.43	2.08	1.72	1.43	2.08	1.63	1.28	2.08	1.35	2.20
Liu G (80)	2018	NLR	STS	OS	13	2658*	1.18	1.10	1.27	1.56	1.3	1.88	1.80	1.25	2.60	0.88	2.77
Li LQ (79)	2020	NLR	STS (Synovial Sarcoma)	OS	3	NA†	2.39	1.89	3.02	2.39	1.89	3.02	2.97	1.17	7.51	0.52	10.89
Li LQ (79)	2020	NLR	STS (Liposarcoma)	OS	2	NA†	2.94	1.81	4.77	2.94	1.81	4.77	2.91	1.65	5.12		
Feng J (81)	2020	NLR	Thyroid	DFS	7	6343	1.21	1.02	1.44	1.85	0.93	3.65	2.96	1.08	8.09	0.24	14.09
Luo Y (76)	2015	NLR	Upper Urinary	RFS	2	802	1.47	1.11	1.95	1.5	1.07	2.08	1.38	1.02	1.87	NA	NA
Wei Y (82)	2014	NLR	Urinary	OS	13	3391	1.55	1.38	1.73	1.83	1.48	2.26	1.19	0.8	1.77	0.95	3.54
Su S (83)	2019	NLR	Urothelial	OS	7	2554	1.24	1.16	1.34	1.32	1.03	1.69	1.91	0.35	4.05	0.65	2.66
Su S (83)	2019	NLR	Urothelial	DFS	2	400	1.38	1.02	1.86	1.44	0.89	2.34	1.91	1.15	3.17		
Vartolomei MD (84)	2018	NLR	Urothelial	RFS	6	3563	1.26	1.09	1.45	1.61	1.16	2.22	1.05	0.87	1.26	0.62	4.14
Suh J (9)	2019	NLR	Urothelial (Chemotherapy)	OS	5	1664	1.39	1.27	1.52	1.45	1.27	1.65	1.46	1.17	1.83	1.02	2.07
Suh J (9)	2019	NLR	Urothelial (Chemotherapy)	PFS	2	500	1.23	1.10	1.37	1.3	1.02	1.64	1.51	1.16	1.97		
Suh J (9)	2019	NLR	Urothelial (Nephroureterectomy)	OS	3	1103	1.72	1.31	2.25	1.72	1.31	2.25	1.58	1.12	2.23	0.30	9.91
Suh J (9)	2019	NLR	Urothelial (Nephroureterectomy)	PFS	8	4411	1.30	1.13	1.50	1.63	1.22	2.18	1.05	0.87	1.27	0.73	3.64
Suh J (9)	2019	NLR	Urothelial (Radical Cystectomy)	CSS	9	6974	1.06	1.04	1.09	1.53	1.18	1.97	1.20	1.10	1.31	0.66	3.56
Suh J (9)	2019	NLR	Urothelial (Radical Cystectomy)	OS	10	7049	1.04	1.02	1.06	1.51	1.15	1.98	1.10	1.02	1.18	0.60	3.84
Suh J (9)	2019	NLR	Urothelial (Radical Cystectomy)	PFS	5	5935	1.06	1.03	1.09	1.18	1.01	1.37	1.20	1.10	1.31	0.69	2.01
Mori K (85)	2020	NLR	UTUC (Nephroureterectomy)	CSS	12	6350	1.46	1.29	1.66	1.64	1.34	2.02	1.07	0.87	1.31	0.96	2.83
Mori K (86)	2020	NLR	UTUC (Radical Cystectomy)	CSS	12	11158	1.07	1.05	1.09	1.29	1.13	1.47	1.20	1.20	1.40	0.85	1.95

(NA) Not Applicable, (HR), Hazard ratio, (NLR) Neutrophil to Lymphocyte Ratio, (IN) Intratumoural Neutrophils, (PN) Peritumoural Neutrophils, (SN) Stromal Neutrophils, (OS) Overall Survival, (DFS) Disease-Free Survival, (PFS) Progression-Free Survival, (RFS) Reoccurrence-Free Survival, (CSS) Cancer Specific Survival, (VEGFR) Vascular Endothelial Growth Factor Receptor, (PC) Palliative Chemotherapy, (SBR) Surgical Bowel Resection, (SR) Surgical Resection, (CLM) Colorectal Liver Metastasis, (NS) Non-surgical, (HCC) Hepatocellular Carcinoma, (ICC) Intrahepatic Cholangiocarcinoma, (RFA) Radiofrequency Ablation, (TACE) Trans-Arterial Chemoembolization, (MPM) Malignant Pleural Mesothelioma, (NSCLC) Non-Small Cell Lung Cancer, (NHC), Neck and Head Cancer, (CR) Castration Resistant, (TKI) Tyrosine Kinase Inhibitor. *All cancers are defined as a grouping of cancer diagnosis unrelated to site, stage or treatment unless otherwise specified. ⸸review reported incomplete data on sample size.

References:

1. Jiang T, Qiao M, Zhao C, et al. Pretreatment neutrophil-to-lymphocyte ratio is associated with outcome of advanced-stage cancer patients treated with immunotherapy: a meta-analysis. Cancer Immunol Immunother. 2018;67(5):713–27.
2. Mei Z, Shi L, Wang B, et al. Prognostic role of pretreatment blood neutrophil-to-lymphocyte ratio in advanced cancer survivors: A systematic review and meta-analysis of 66 cohort studies. Cancer Treat Rev. 2017;58:1–13.
3. Shen M, Hu P, Donskov F, et al. Tumor-associated neutrophils as a new prognostic factor in cancer: a systematic review and meta-analysis. PLoS One. 2014;9(6):e98259.
4. Paramanathan A, Saxena A, Morris DL. A systematic review and meta-analysis on the impact of pre-operative neutrophil lymphocyte ratio on long term outcomes after curative intent resection of solid tumours. Surg Oncol. 2014;23(1):31–9.
5. Dolan RD, McSorley ST, Horgan PG, et al. The role of the systemic inflammatory response in predicting outcomes in patients with operable cancer: Systematic review and meta-analysis. Sci Rep. 2017;116(1):134–46.
6. Tang H, Lu W, Li B, et al. Prognostic significance of neutrophil-to-lymphocyte ratio in biliary tract cancers: a systematic review and meta-analysis. Oncotarget. 2017;8(22):36857–68.
7. Hu G, Xu F, Zhong K, et al. The prognostic role of preoperative circulating neutrophil-lymphocyte ratio in primary bladder cancer patients undergoing radical cystectomy: a meta-analysis. World J Urol. 2019;37(9):1817–25. doi: https://dx.doi.org/10.1007/s00345-018-2593-z
8. Wu S, Zhao X, Wang Y, et al. Pretreatment Neutrophil-Lymphocyte Ratio as a Predictor in Bladder Cancer and Metastatic or Unresectable Urothelial Carcinoma Patients: a Pooled Analysis of Comparative Studies. Cell Physiol Biochem. 2018;46(4):1352–64.
9. Suh J, Jung JH, Jeong CW, et al. Clinical Significance of Pre-treated Neutrophil-Lymphocyte Ratio in the Management of Urothelial Carcinoma: A Systemic Review and Meta-Analysis. Front Oncol. 2019;9:1365. doi: http://dx.doi.org/10.3389/fonc.2019.01365
10. Li X, Ma X, Tang L, et al. Prognostic value of neutrophil-to-lymphocyte ratio in urothelial carcinoma of the upper urinary tract and bladder: a systematic review and meta-analysis. Oncotarget. 2017;
11. Ethier J-L, Desautels D, Templeton A, et al. Prognostic role of neutrophil-to-lymphocyte ratio in breast cancer: a systematic review and meta-analysis. Breast Cancer Res. 2017;19(1):2.
12. Liu X, Qu JK, Zhang J, et al. Prognostic role of pretreatment neutrophil to lymphocyte ratio in breast cancer patients. Med (United States). 2017;96(45):e8101. doi: 10.1097/MD.0000000000008101
13. Chen J, Pan Y, He B, et al. Meta-analysis of prognostic value of inflammation parameter in breast cancer. J Cancer Res Ther. 2018;14(Supplement):ate of Pubaton: 01 Mar 2018.
14. Duan J, Pan L, Yang M. Preoperative elevated neutrophil-to-lymphocyte ratio (NLR) and derived NLR are associated with poor prognosis in patients with breast cancer: A meta-analysis. Medicine (Baltimore). 2018;97(49):e13340. doi: https://dx.doi.org/10.1097/MD.0000000000013340
15. Guo W, Lu X, Liu Q, et al. Prognostic value of neutrophil-to-lymphocyte ratio and platelet-to-lymphocyte ratio for breast cancer patients: An updated meta-analysis of 17079 individuals. Cancer Med. 2019;8(9):4135–48. doi: https://dx.doi.org/10.1002/cam4.2281
16. Wu J, Chen M, Liang C, et al. Prognostic value of the pretreatment neutrophil-to-lymphocyte ratio in cervical cancer: a meta-analysis and systematic review. Oncotarget. 2017;8(8):13400–12.
17. Zhang J, Zhang H-Y, Li J, et al. The elevated NLR, PLR and PLT may predict the prognosis of patients with colorectal cancer: a systematic review and meta-analysis. Oncotarget. 2017;8(40):68837–46.
18. Zheng DC, Zheng C, Wu J, et al. Neutrophil-lymphocyte ratio predicts the prognosis of patients with colorectal cancer: A meta-analysis. Int J Clin Exp Med. 2015;9(1):78–90.
19. Malietzis G, Giacometti M, Kennedy RH, et al. The emerging role of neutrophil to lymphocyte ratio in determining colorectal cancer treatment outcomes: a systematic review and meta-analysis. Ann Surg Oncol. 2014;21(12):3938–46.
20. Li H, Zhao Y, Zheng F. Prognostic significance of elevated preoperative neutrophil-to-lymphocyte ratio for patients with colorectal cancer undergoing curative surgery: A meta-analysis. Medicine (Baltimore). 2019;98(3):e14126. doi: http://dx.doi.org/10.1097/MD.0000000000014126
21. Tang H, Li B, Zhang A, et al. Prognostic significance of neutrophil-to-lymphocyte ratio in colorectal liver metastasis: A systematic review and meta-analysis. PLoS One. 2016;11(7):e0159447.
22. Ni L, Tao J, Xu J, et al. Prognostic values of pretreatment neutrophil - to - lymphocyte and platelet - to - lymphocyte ratios in endometrial cancer : a systematic review and meta - analysis. 2019;(0123456789). doi: 10.1007/s00404-019-05372-w
23. Sun J, Chen X, Gao P, et al. Can the Neutrophil to Lymphocyte Ratio Be Used to Determine Gastric Cancer Treatment Outcomes? A Systematic Review and Meta-Analysis. Dis Markers. 2016;2016:7862469.
24. Zhang X, Zhang W, Feng L. Prognostic significance of neutrophil lymphocyte ratio in patients with gastric cancer: a meta-analysis. PLoS One. 2014;9(11):e111906.
25. Szor DJ, Dias AR, Pereira MA, et al. Prognostic Role of Neutrophil/Lymphocyte Ratio in Resected Gastric Cancer: A Systematic Review and Meta-analysis. Clinics (Sao Paulo). 2018;73:e360.
26. Mellor KL, Powell AGMT, Lewis WG. Systematic Review and Meta-Analysis of the Prognostic Significance of Neutrophil-Lymphocyte Ratio (NLR) After R0 Gastrectomy for Cancer. J Gastrointest Cancer. 2018;49(3):237–44.

27. Zhou Y, Li D, Lin Y, et al. Pretreatment hematologic markers as prognostic predictors of gastroenteropancreatic neuroendocrine tumors: a systematic review and meta-analysis. Onco Targets Ther. 2018;11:2489–96.
28. Luo X-F, Zhou L-H. Prognostic significance of neutrophil to lymphocyte ratio in patients with gastrointestinal stromal tumors: A meta-analysis. Clin Chim Acta. 2018;477:7–12.
29. Lei Y-Y, Li Y-T, Hu Q-L, et al. Prognostic impact of neutrophil-to-lymphocyte ratio in gliomas: A systematic review and meta-analysis. World J Surg Oncol. 2019;17(1):152. doi: https://dx.doi.org/10.1186/s12957-019-1686-5
30. Ethier J-L, Desautels DN, Templeton AJ, et al. Is the neutrophil-to-lymphocyte ratio prognostic of survival outcomes in gynecologic cancers? A systematic review and meta-analysis. Gynecol Oncol. 2017;145(3):584–94.
31. Yu Y, Wang H, Yan A, et al. Pretreatment neutrophil to lymphocyte ratio in determining the prognosis of head and neck cancer: a meta-analysis. BMC Cancer. 2018;18(1):383.
32. Tham T, Bardash Y, Herman SW, et al. Neutrophil-to-lymphocyte ratio as a prognostic indicator in head and neck cancer: A systematic review and meta-analysis. Head Neck. 2018;(pagination):ate of Pubaton: 2018.
33. Yang L, Huang Y, Zhou L, et al. High pretreatment neutrophil-to-lymphocyte ratio as a predictor of poor survival prognosis in head and neck squamous cell carcinoma: Systematic review and meta-analysis. Head Neck. 2019;41(5):1525–35. doi: https://dx.doi.org/10.1002/hed.25583
34. Cho J-K, Kim MW, Choi IS, et al. Optimal cutoff of pretreatment neutrophil-to-lymphocyte ratio in head and neck cancer patients: a meta-analysis and validation study. BMC Cancer. 2018;18(1):969. doi: https://dx.doi.org/10.1186/s12885-018-4876-6
35. Min G-TT, Li Y-MM, Yao N, et al. The pretreatment neutrophil-lymphocyte ratio may predict prognosis of patients with liver cancer: A systematic review and meta-analysis. Clin Transplant. 2018;32(1):Arte Number: e13151. ate of Pubaton: January 2018.
36. Xiao W-K, Chen D, Li S-Q, et al. Prognostic significance of neutrophil-lymphocyte ratio in hepatocellular carcinoma: a meta-analysis. BMC Cancer. 2014;14:117.
37. Liu L, Gong Y, Zhang Q, et al. Prognostic Roles of Blood Inflammatory Markers in Hepatocellular Carcinoma Patients Taking Sorafenib. A Systematic Review and Meta-Analysis. Front Oncol. 2019;9:1557. doi: https://dx.doi.org/10.3389/fonc.2019.01557
38. Wang Y, Peng C, Cheng Z, et al. The prognostic significance of preoperative neutrophil-lymphocyte ratio in patients with hepatocellular carcinoma receiving hepatectomy: A systematic review and meta-analysis. Int J Surg. 2018;55:73–80.
39. Li S, Feng X, Cao G, et al. Prognostic significance of inflammatory indices in hepatocellular carcinoma treated with transarterial chemoembolization: A systematic review and meta-analysis. PLoS One. 2020;15(3):e0230879. doi: https://dx.doi.org/10.1371/journal.pone.0230879
40. Xu Z-GG, Ye C-JJ, Liu L-XX, et al. The pretransplant neutrophil-lymphocyte ratio as a new prognostic predictor after liver transplantation for hepatocellular cancer: a systematic review and meta-analysis. Biomark Med. 2018;12(2):189–99.
41. Tan Q, Liu S, Liang C, et al. Pretreatment hematological markers predict clinical outcome in cancer patients receiving immune checkpoint inhibitors: A meta-analysis. Thorac Cancer. 2018;
42. Xie X, Liu J, Yang H, et al. Prognostic Value of Baseline Neutrophil-to-Lymphocyte Ratio in Outcome of Immune Checkpoint Inhibitors. Cancer Invest. 2019;37(6):265–74. doi: https://dx.doi.org/10.1080/07357907.2019.1639057
43. Mu S, Ai L, Fan F, et al. Prognostic role of neutrophil-to-lymphocyte ratio in diffuse large B cell lymphoma patients: an updated dose-response meta-analysis. Cancer Cell Int. 2018;18:119.
44. Mascarella MA, Mannard E, Silva SD, et al. Neutrophil-to-lymphocyte ratio in head and neck cancer prognosis: A systematic review and meta-analysis. Head Neck. 2018;40(5):1091–100.
45. Zhao Q-T, Yang Y, Xu S, et al. Prognostic role of neutrophil to lymphocyte ratio in lung cancers: a meta-analysis including 7,054 patients. Onco Targets Ther. 2015;8:2731–8.
46. Yu Y, Qian L, Cui J. Value of neutrophil-to-lymphocyte ratio for predicting lung cancer prognosis: A meta-analysis of 7,219 patients. Mol Clin Oncol. 2017;7(3):498–506.
47. Zhan H, Ma J-Y, Jian Q-C. Prognostic significance of pretreatment neutrophil-to-lymphocyte ratio in melanoma patients: A meta-analysis. Clin Chim Acta. 2018;484:136–40. doi: 10.1016/j.cca.2018.05.055
48. Ding Y, Zhang S, Qiao J. Prognostic value of neutrophil-to-lymphocyte ratio in melanoma: Evidence from a PRISMA-compliant meta-analysis. Medicine (Baltimore). 2018;97(30):e11446.
49. Chen N, Liu S, Huang L, et al. Prognostic significance of neutrophil-to-lymphocyte ratio in patients with malignant pleural mesothelioma: a meta-analysis. Oncotarget. 2017;
50. Zeng Q, Liu Z, Li Q, et al. Prognostic value of neutrophil to lymphocyte ratio and clinicopathological characteristics for multiple myeloma: A meta-analysis. Medicine (Baltimore). 2018;97(41):e12678. doi: https://dx.doi.org/10.1097/MD.0000000000012678
51. Yang S, Zhao K, Ding X, et al. Prognostic Significance of Hematological Markers for Patients with Nasopharyngeal Carcinoma: A Meta-analysis. J Cancer. 2019;10(11):2568–77. doi: https://dx.doi.org/10.7150/jca.26770
52. Yin J, Qin Y, Luo YK, et al. Prognostic value of neutrophil-to-lymphocyte ratio for nasopharyngeal carcinoma. Med (United States). 2017;96(29) (pagination):Arte Number: e7577. ate of Pubaton: 01 Ju 2017.
53. Su L, Zhang M, Zhang W, et al. Pretreatment hematologic markers as prognostic factors in patients with nasopharyngeal carcinoma. Med (United States). 2017;96(11) (pagination):Arte Number: e6364. ate of Pubaton: 01 Mar 2017.
54. Li X, Dai D, Chen B, et al. The value of neutrophil-to-lymphocyte ratio for response and prognostic effect of neoadjuvant chemotherapy in solid tumors: A systematic review and meta-analysis. J Cancer. 2018;9(5):861–71.
55. Vartolomei MD, PoravHodade D, Ferro M, et al. Prognostic role of pretreatment neutrophil-to-lymphocyte ratio (NLR) in patients with non-muscle-invasive bladder cancer (NMIBC): A systematic review and meta-analysis. Urol Oncol Semin Orig Investig. 2018;(pagination):ate of Pubaton: 2018.
56. Lu Y, Jiang J, Yan L, et al. The clinicopathological and prognostic value of the pretreatment neutrophil-to-lymphocyte ratio in small cell lung cancer: A meta-analysis. PLoS One. 2020;15(4):e0230979. doi: https://dx.doi.org/10.1371/journal.pone.0230979
57. Gu X, Tian T, Tian X, et al. Prognostic significance of neutrophil-to-lymphocyte ratio in non-small cell lung cancer: a meta-analysis. Sci Rep. 2015;5:12493. doi: http://dx.doi.org/10.1038/srep12493

58. Wang Z, Zhan P, Lv Y, et al. Prognostic role of pretreatment neutrophil-to-lymphocyte ratio in non-small cell lung cancer patients treated with systemic therapy: A meta-analysis. Transl lung cancer Res. 2019;8(3):214–26. doi: https://dx.doi.org/10.21037/tlcr.2019.06.10
59. Cao D, Xu H, Xu X, et al. A reliable and feasible way to predict the benefits of Nivolumab in patients with non-small cell lung cancer: a pooled analysis of 14 retrospective studies. Oncoimmunology. 2018;(pagination):ate of Pubaton: 2018.
60. Jiang T, Bai Y, Zhou F, et al. Clinical value of neutrophil-to-lymphocyte ratio in patients with non-small-cell lung cancer treated with PD-1/PD-L1 inhibitors. Lung Cancer. 2019;130:76–83. doi: https://dx.doi.org/10.1016/j.lungcan.2019.02.009
61. Zhang X, Wang Y, Zhao L, et al. Prognostic value of platelet-to-lymphocyte ratio in oncologic outcomes of esophageal cancer: A systematic review and meta-analysis. Int J Biol Markers. 2018;33(4):335–44. doi: http://dx.doi.org/10.1177/1724600818766889
62. Pirozzolo G, Gisbertz SS, Castoro C, et al. Neutrophil-to-lymphocyte ratio as prognostic marker in esophageal cancer: A systematic review and meta-analysis. J Thorac Dis. 2019;11(7):3136–45. doi: http://dx.doi.org/10.21037/jtd.2019.07.30
63. Yang Y, Liu R, Ren F, et al. Prognostic and clinicopathological significance of neutrophil-to-lymphocyte ratio in patients with oral cancer. Biosci Rep. 2018;38(6). doi: https://dx.doi.org/10.1042/BSR20181550
64. Wang Y, Wang P, Andrukhov O, et al. Meta-analysis of the prognostic value of the neutrophil-to-lymphocyte ratio in oral squamous cell carcinoma. J Oral Pathol Med. 2018;47(4):353–8.
65. Zhou Q, Hong L, Zuo M-Z, et al. Prognostic significance of neutrophil to lymphocyte ratio in ovarian cancer: evidence from 4,910 patients. Oncotarget. 2017;8(40):68938–49.
66. Zhou Y, Cheng S, Fathy A, et al. Prognostic value of platelet-to-lymphocyte ratio in pancreatic cancer: A comprehensive meta-analysis of 17 cohort studies. Onco Targets Ther. 2018;11:1899–908. doi: http://dx.doi.org/10.2147/OTT.S154162
67. Yang J, Hu Z, Shi W, et al. Prognostic significance of neutrophil to lymphocyte ratio in pancreatic cancer: A meta-analysis. World J Gastroenterol. 2015;21(9):2807–15. doi: http://dx.doi.org/10.3748/wjg.v21.i9.2807
68. Mowbray NG, Griffith D, Hammoda M, et al. A meta-analysis of the utility of the neutrophil-to-lymphocyte ratio in predicting survival after pancreatic cancer resection. HPB. 2018;20(5):379–84.
69. Zhou Y, Li D, Lin Y, et al. Pretreatment hematologic markers as prognostic predictors of gastroenteropancreatic neuroendocrine tumors: A systematic review and meta-analysis. Onco Targets Ther. 2018;11:2489–96.
70. Guo J, Fang J, Huang X, et al. Prognostic role of neutrophil to lymphocyte ratio and platelet to lymphocyte ratio in prostate cancer: A meta-analysis of results from multivariate analysis. Int J Surg. 2018;60:216–23. doi: https://dx.doi.org/10.1016/j.ijsu.2018.11.020
71. Cao J, Zhu X, Zhao X, et al. Neutrophil-to-Lymphocyte Ratio Predicts PSA Response and Prognosis in Prostate Cancer: A Systematic Review and Meta-Analysis. PLoS One. 2016;11(7):e0158770.
72. Yin X, Xiao Y, Li F, et al. Prognostic Role of Neutrophil-to-Lymphocyte Ratio in Prostate Cancer: A Systematic Review and Meta-analysis. Medicine (Baltimore). 2016;95(3):e2544. doi: 10.1097/MD.0000000000002544
73. Guan Y, Xiong H, Feng Y, et al. Revealing the prognostic landscape of neutrophil-to-lymphocyte ratio and platelet-to-lymphocyte ratio in metastatic castration-resistant prostate cancer patients treated with abiraterone or enzalutamide: a meta-analysis. Prostate Cancer Prostatic Dis. 2020;23(2):220–31. doi: https://dx.doi.org/10.1038/s41391-020-0209-3
74. Dong YW, Shi YQ, He LW, et al. Prognostic significance of neutrophil-to-lymphocyte ratio in rectal cancer: A meta-analysis. Onco Targets Ther. 2016;9:3127–34. doi: 10.2147/OTT.S103031
75. Na N, Yao J, Cheng C, et al. Meta-analysis of the efficacy of the pretreatment neutrophil-tolymphocyte ratio as a predictor of prognosis in renal carcinoma patients receiving tyrosine kinase inhibitors. Oncotarget. 2016;7(28):44039–46.
76. Luo Y, She D-L, Xiong H, et al. Pretreatment Neutrophil to Lymphocyte Ratio as a Prognostic Predictor of Urologic Tumors: A Systematic Review and Meta-Analysis. Medicine (Baltimore). 2015;94(40):e1670.
77. Semeniuk-Wojtas A, Lubas A, Stec R, et al. Neutrophil-to-lymphocyte Ratio, Platelet-to-lymphocyte Ratio, and C-reactive Protein as New and Simple Prognostic Factors in Patients With Metastatic Renal Cell Cancer Treated With Tyrosine Kinase Inhibitors: A Systemic Review and Meta-analysis. Clin Genitourin Cancer. 2018;16(3):e693.
78. Boissier R, Campagna J, Branger N, et al. The prognostic value of the neutrophil-lymphocyte ratio in renal oncology: A review. Urol Oncol. 2017;35(4):135–41.
79. Li L, Bai Z, Zhang L, et al. Meta-Analysis of Hematological Biomarkers as Reliable Indicators of Soft Tissue Sarcoma Prognosis. 2020;10(January):1–12. doi: 10.3389/fonc.2020.00030
80. Liu G, Ke L-CC, Sun S-RR. Prognostic value of pretreatment neutrophil-to-lymphocyte ratio in patients with soft tissue sarcoma: A meta-analysis. Medicine (Baltimore). 2018;97(36):e12176.
81. Feng J, Wang Y, Shan G, et al. Clinical and prognostic value of neutrophil-lymphocyte ratio for patients with thyroid cancer: A meta-analysis. Medicine (Baltimore). 2020;99(20):e19686. doi: http://dx.doi.org/10.1097/MD.0000000000019686
82. Wei Y, Jiang Y-Z, Qian W-H. Prognostic role of NLR in urinary cancers: a meta-analysis. PLoS One. 2014;9(3):e92079.
83. Su S, Liu L, Li C, et al. Prognostic role of pretreatment derived neutrophil to lymphocyte ratio in urological cancers: A systematic review and meta-analysis. Int J Surg. 2019;72:146–53. doi: https://dx.doi.org/10.1016/j.ijsu.2019.10.043
84. Vartolomei MD, Kimura S, Ferro M, et al. Is neutrophil-to-lymphocytes ratio a clinical relevant preoperative biomarker in upper tract urothelial carcinoma? A meta-analysis of 4385 patients. World J Urol. 2018;36(7):1019–29.
85. Mori K, Janisch F, Mostafaei H, et al. Prognostic value of preoperative blood-based biomarkers in upper tract urothelial carcinoma treated with nephroureterectomy: A systematic review and meta-analysis. Urol Oncol. 2020;38(5):315–33. doi: http://dx.doi.org/10.1016/j.urolonc.2020.01.015
86. Mori K, Miura N, Mostafaei H, et al. Prognostic value of preoperative hematologic biomarkers in urothelial carcinoma of the bladder treated with radical cystectomy: a systematic review and meta-analysis. Int J Clin Oncol. 2020; doi: https://dx.doi.org/10.1007/s10147-020-01690-1
